# Catalytic asymmetric cationic shifts of aliphatic hydrocarbons

**DOI:** 10.1038/s41586-023-06826-7

**Published:** 2024-01-10

**Authors:** Vijay N. Wakchaure, William DeSnoo, Croix J. Laconsay, Markus Leutzsch, Nobuya Tsuji, Dean J. Tantillo, Benjamin List

**Affiliations:** 1 Max Planck Institut für Kohlenforschung, Mülheim an der Ruhr, Germany; 2 Department of Chemistry, University of California, Davis, Davis, CA USA; 3 Institute for Chemical Reaction Design and Discovery (WPI-ICReDD), Hokkaido University, Sapporo, Japan

**Keywords:** Asymmetric synthesis, Biomimetic synthesis

## Abstract

Asymmetric catalysis is an advanced area of chemical synthesis, but the handling of abundantly available, purely aliphatic hydrocarbons has proven to be challenging. Typically, heteroatoms or aromatic substructures are required in the substrates and reagents to facilitate an efficient interaction with the chiral catalyst. Confined acids have recently been introduced as tools for homogenous asymmetric catalysis, specifically to enable the processing of small unbiased substrates^1^. However, asymmetric reactions in which both substrate and product are purely aliphatic hydrocarbons have not previously been catalysed by such super strong and confined acids. We describe here an imidodiphosphorimidate-catalysed asymmetric Wagner–Meerwein shift of aliphatic alkenyl cycloalkanes to cycloalkenes with excellent regio- and enantioselectivity. Despite their long history and high relevance for chemical synthesis and biosynthesis, Wagner–Meerwein reactions utilizing purely aliphatic hydrocarbons, such as those originally reported by Wagner and Meerwein, had previously eluded asymmetric catalysis.

## Abstract (web summary)

We describe an imidodiphosphorimidate-catalysed asymmetric Wagner–Meerwein shift of aliphatic alkenyl cycloalkanes to cycloalkenes with excellent regio- and enantioselectivity.

## Main

**Fig. 1:**
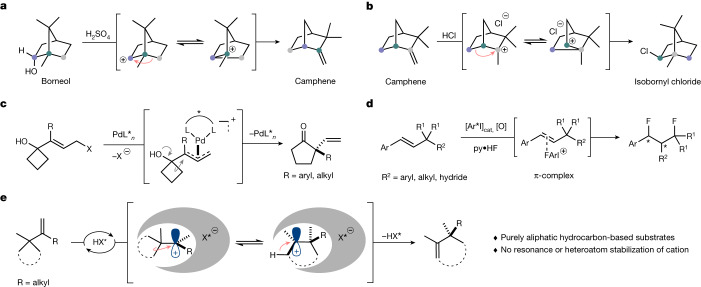
Carbocation rearrangements. **a**, Borneol to camphene rearrangement (Wagner 1899)^46^. **b**, Camphene to isobornyl chloride rearrangement (Meerwein 1922)^47,48^. **c**, Palladium-catalysed semipinacol rearrangement/Wagner–Meerwein shift. **d**, Aryl iodide-catalysed enantioselective 1,3-difluorination. **e**, Catalytic asymmetric Wagner–Meerwein shifts of aliphatic hydrocarbons (this work). HX*, Brønsted acid.

Since Bredig and Fiske^2^ discovered a nonenzymatic, modestly enantioselective cinchona alkaloid-catalysed cyanohydrin synthesis in 1912^3^, asymmetric chemical catalysis has evolved extensively and currently encompasses reactions with transition metals^4–6^, enzymes^7,8^ and organocatalysts^9–11^. However, the catalytic and enantioselective processing of purely aliphatic hydrocarbons is still extremely challenging. Typical substrates and reagents used in asymmetric catalysis feature heteroatoms or aromatic groups, which enhance reactivity and enable enantiodifferentiation by providing functional groups that engage in specific interactions with the catalyst. Indeed, to the best of our knowledge, Pfaltz’s landmark iridium-catalysed asymmetric hydrogenation of a purely alkyl-substituted olefin stands out as the only example in which an only-aliphatic hydrocarbon is catalytically processed to give an enantiopure only-aliphatic hydrocarbon^12–14^. We became interested in advancing hydrocarbon chemistry by investigating cationic shifts as a fundamental class of chemical and biochemical transformations. The most prominent example in this regard is the cationic Wagner–Meerwein rearrangement^15,16^. Catalytic enantioselective Wagner–Meerwein reactions that proceed through purely aliphatic hydrocarbon-based cations, such as those originally reported by Wagner and Meerwein, have not previously been described (Fig. 1a,b)^17–21^.Notable examples of enantioselective cationic shifts were reported by Trost and Yasukata^22^, Trost and Xie^23^ and Jacobsen and coworkers^24,25^, and feature π-allyl palladium intermediates to afford enantioenriched α-vinyl cycloketones (Fig. 1c) or a hypervalent iodine-catalysed, 1,3-difluorinative reaction of β-substituted styrenes (Fig. 1d).

We have recently introduced strong and chiral confined imidodiphosphorimidate (IDPi) Brønsted acids as a new and general tool for asymmetric catalysis^26,27^. IDPi catalysts have activated unbiased olefins in hydroalkoxylations^28^, hydroarylations^29^ and hydrolactonizations^30^ and even enabled control over purely hydrocarbon-based non-classical carbocations^31,32^. Encouraged by these results, we became tempted to challenge our IDPi motif with purely aliphatic hydrocarbon-based substrates toward highly enantioselective Wagner–Meerwein shifts and describe here the results of these investigations (Fig. 1e).

**Fig. 2:**
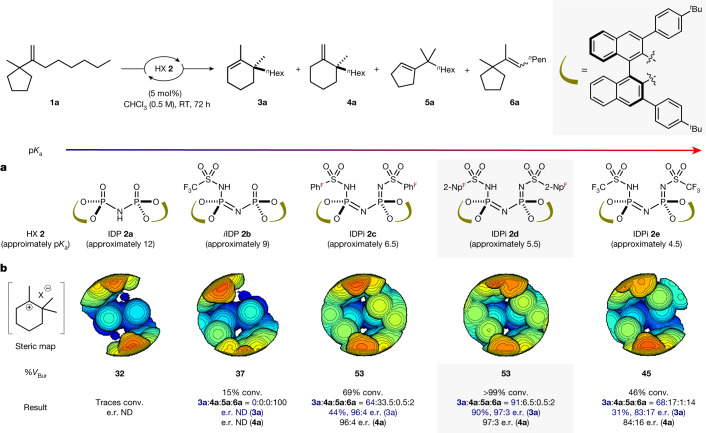
Investigation of acidity, confinement and chiral pocket size of catalyst in the catalytic Wagner–Meerwein shift. Yields and conversion were determined by ^1^H NMR spectroscopy using 1,3,5-trimethoxybenzene as the internal standard. Side product distribution ratios were determined by crude ^1^H NMR spectroscopy. The enantiomeric ratio was determined by gas chromatography analysis (Supplementary Information has details). **a**, Estimated p*K*_a_ values in CH_3_CN based on the literature report for a similar catalyst^27,49^. **b**, Calculated steric map of simplified substrate visualized by SambVca 2.1 (ref. ^50^). The map is viewed from the centre of the substrate and directed toward the active site of each catalyst. The colour indicates depth along the *z* axis; the red zone is closer to the substrate, whereas the blue zone is farther away. For the ion pair of **2b**, one of the C–H bond lengths is fixed (Supplementary Fig. 21). conv., conversion; e.r., enantiomeric ratio; IDP, imidodiphosphoric acid; *i*IDP, iminoimidodiphosphoric acid; ND, not determined; ^*n*^Hex, *n*-hexyl; ^*n*^Pen, *n*-pentyl; 2-Np^F^, 2-perfluoronaphthyl; Ph^F^, pentafluorophenyl; p*K*_a_, negative logarithm of the acid dissociation constant; RT, room temperature; ^*t*^Bu, *tert*-butyl; %*V*_Bur_, percent buried volume.

At the onset of our studies, we subjected olefin **1a** to 5 mol% of various confined chiral Brønsted acid catalysts covering a broad p*K*_a_(negative logarithm of the acid dissociation constant *K*_a_) range at room temperature for 24 h to obtain ring-expanded cycloalkene **3a** (Fig. 2). As expected, weaker acids, such as imidodiphosphoric acid **2a**, failed to give any reactivity. Similarly, iminoimidodiphosphoric acid **2b** gave only poor conversion of substrate **1a** to furnish olefin isomerization product **6a**. By contrast, our highly acidic and confined IDPi catalyst **2c** gave product **3a** in moderate conversion and a high enantiomeric ratio of 96:4. A substantial amount of exoproduct **4a** is also formed in similar enantioselectivity and is slowly converted into the corresponding endoproduct **3a**, already suggesting that deprotonation cannot be enantiodetermining. We further observed trace amounts of product **5a** (less than 0.5%) in which a methyl group has migrated. Our highly acidic and more confined IDPi catalyst apparently suppresses olefin isomerization of the starting material to give hydrocarbon **6a** (less than 2%). Notably, a change in the sulfonamide inner core of the catalyst from pentafluorophenylsulfonyl to perfluoronaphthylsulfonyl (IDPi **2d**) delivered a drastic increase in reactivity and gave full conversion with excellent yield and enantioselectivity. We hypothesized that the high chemoselectivity, enantiocontrol and overall reactivity were enabled by the highly confined microenvironment offered by the IDPi catalysts. To visually assess this confinement effect, buried volumes were calculated using a model ion pair of the catalysts^33,34^ (Fig. 2). A higher percent buried volume, which corresponds to a narrower pocket, may indicate that the carbocation is stabilized within the IDPi anion pocket, possibly compensating for the absence of traditional heteroatom or resonance stabilization. Indeed, consistent with these expectations, the sulfonylimidophosphoryl groups not only modulate the overall acidity but also contribute to the confinement of the catalytic active site. Notably, when compared with IDPi **2d** (53% buried volume), the even more acidic IDPi **2e**, which provides a relatively larger pocket (45% buried volume), afforded product **3a** in only poor yield. At 46% consumption of substrate **1a**, only 31% yield of product **3a** was obtained, with moderate enantioselectivity along with more olefin isomerization product **6a**. These results suggest that a fine-tuned balance between acidity and confinement is crucial to achieve high chemoselectivity, enantiocontrol and overall reactivity. After a brief screening of catalysts and reaction conditions (Supplementary Table 1), we selected catalyst **2d**, CHCl_3_ (4 M) and room temperature for 24 h and found that product **3a** can be obtained in both excellent yield and enantioselectivity (91%, 97:3 enantiomeric ratio).

**Fig. 3:**
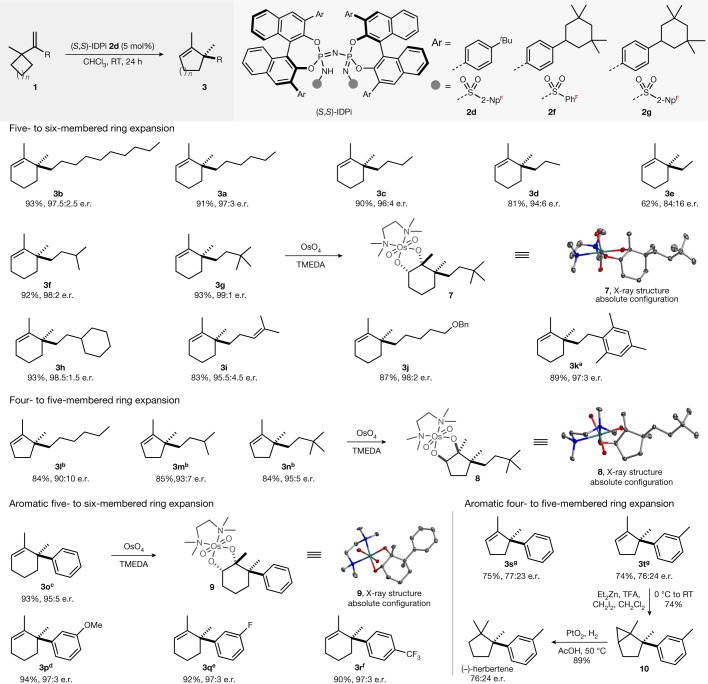
Scope of the catalytic asymmetric Wagner–Meerwein shift. Reactions were performed at 0.25 mmol scale. Isolated yields after chromatographic purification. The enantiomeric ratio was determined by gas chromatography analysis (Supplementary Information has details). ^a^At 50 °C for 24 h. ^b^With catalyst **2f** in *n-*hexane at room temperature for 36 h. ^c^With catalyst **2g** in methyl cyclohexane at 60 °C for 48 h. ^d^With catalyst **2g** in methyl cyclohexane at 60 °C for 72 h. ^e^With catalyst **2g** in methyl cyclohexane at 80 °C for 96 h. ^f^With catalyst **2g** in methyl cyclohexane at 90 °C for 6 days. ^g^With catalyst **2d** in methyl cyclohexane at 50 °C for 24 h. The structure of osmate ester **7** derived from product **3g**, H atoms and the disordered atoms are omitted for clarity. The structure of one of the two independent molecules of osmate ester **8** contained in the unit cell derived from product **3n** and H atoms are omitted for clarity. The structure of osmate ester **9** derived from product **3o**, H atoms, water molecule and the disordered atoms are omitted for clarity (Supplementary Figs. 10–16). Bn, benzyl; TFA, trifluoroacetic acid; TMEDA, *N*,*N*,*N*′,*N*′-tetramethylethylenediamine.

The substrate scope of the Wagner–Meerwein shift was assessed using a variety of alkenyl cycloalkanes that were readily converted into the corresponding ring-expanded cycloalkene products (Fig. 3). Linear and longer alkyl chains attached to the olefin gave products **3a**–**c** in both excellent yields and enantioselectivities. Shortening the alkyl chain to *n*-propyl (**3d**) retained the high enantioselectivity, but moderate enantioselectivity was obtained with an ethyl group (**3e**, 84:16 enantiomeric ratio). The incremental reduction in enantiomeric ratio (**3b** → **3a** → **3c** → **3d** → **3e**) is consistent with either simple steric repulsion or dispersive interactions of the *n*-alkyl groups contributing to the enantioselectivity. To our delight, branched alkyl substituents at the olefin exhibited high yields and excellent enantioselectivities (**3f**–**h**). A substrate bearing an alkene substituent was also tolerated, providing product **3i** with an enantiomeric ratio of 95.5:4.5 in 83% yield. *O*-benzyl and aryl group functionalities at the alkyl chain can be utilized successfully (**3j** and **3k**). We also investigated the analogous four- to five-membered ring expansion using IDPi **2f**, and both linear and branched alkyl substituents at the olefin gave the desired products in high yields and good to high enantioselectivities (**3l**–**n**). Apart from aliphatic hydrocarbons, aryl-substituted substrates also react. For example, five- to six-membered ring expansion with IDPi **2g** provided the corresponding products **3o**–**r** with different stereoelectronic properties in both high yields and enantioselectivities. Aryl-substituted substrates in the four- to five-membered ring expansion reacted with moderate yields and enantioselectivities (**3s** and **3t**). As expected, both the three- and six-membered ring substrates under the optimal condition are unreactive (Supplementary Fig. 1). To illustrate the synthetic utility of this asymmetric transformation, a short enantioselective total synthesis of (−)-herbertene^35^ was developed. First, product **3t** was converted into cyclopropane **10** by a Simmons–Smith reaction in 74% yield, and hydrogenolysis delivered (−)-herbertene in 89% yield. X-ray diffraction analysis of osmate esters derived from ring expansion products **3g**, **3n** and **3o** allowed the unambiguous determination of the absolute configurations of these products^36^. The stereochemistry of all other products has been assigned by analogy.

**Fig. 4:**
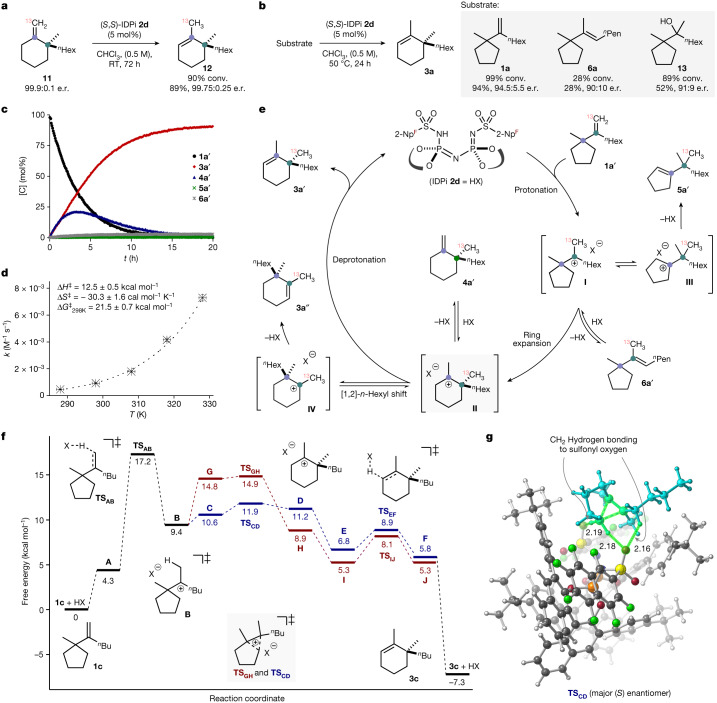
Mechanistic and computational studies. **a**, ^13^C-labelled exocyclic olefin isomerization. **b**, Wagner–Meerwein shift with olefin isomers and corresponding alcohol. Yields and conversion were determined by ^1^H NMR spectroscopy using 1,3,5-trimethoxybenzene as the internal standard. Olefin isomer **6a** contains 3% inseparable side product **5a** (Supplementary Information has details). **c**, Reaction of ^13^C-labelled substrate **1a**′ catalysed by IDPi **2d**. Reaction profile monitored by ^13^C NMR spectroscopy. **d**, Eyring plot obtained for the reaction of IDPi **2d** with substrate **1a**. **e**, A plausible catalytic cycle. **f**, Density functional theory-derived free energy profile (CPCM(CHCl_3_)-ωB97X-D4/def2-TZVPP/CPCM(CHCl_3_)-B3LYP-D3(BJ)/def2-SVP). The red path denotes the path to *R* enantiomer, and the blue path denotes the path to *S*. HX = **2d**. Structures B, C and G are different conformations. B is connected directly to TS_AB_, C is connected directly to TS_CD_ and G is connected directly to TS_GH_ (Supplementary Information has additional details on the conformational landscape of this complex system). **g**, Computed TS_CD_ (major (*S*) enantiomer). Bond lengths shown are in angstrom (Å).

To gain insight into the reaction mechanism, we synthesized enantiopure (greater than 99:1 enantiomeric ratio), ^13^C-labelled exoproduct **11** and subjected it to the reaction conditions (Fig. 4a). Olefin isomerization of olefin **11** with IDPi **2d** gave endoproduct **12** in a greater than 99:1 enantiomeric ratio. Similarly, its enantiomer, ent-**11** (98.3:1.7 enantiomeric ratio), reacted at slightly slower rate (mismatched case) and gave product ent-**12** in 98.4:1.6 enantiomeric ratio (Supplementary Fig. 2). In both cases, we did not observe side products resulting from [1,2]-methyl or [1,2]-*n*-hexyl shifts. Although these results could be interpreted as implying irreversible ring expansion, our computational results indicate that this is not the case (see below). We also subjected the corresponding olefin isomer **6a** and alcohol **13** to the reaction conditions (Fig. 4b). In contrast to substrate **1a**, which afforded the desired product **3a** in excellent yield and enantioselectivity, both substrates **6a** and **13** gave product **3a** in only moderate yield and slightly lower enantioselectivity. In both cases, the substrate is less reactive, and an alternative pathway (for example, the reaction proceeding through protonated alcohol or a concerted pathway that is less enantioselective) might occur. Alternatively, the means by which the carbocation is generated may affect the subsequent rearrangement through a non-equilibrium process (see below). Furthermore, we investigated the reaction progression of ^13^C-labelled substrate **1a**′ with IDPi **2d** by ^13^C nuclear magnetic resonance (NMR) spectroscopy (Fig. 4c). ^13^C NMR data acquired at the beginning of the reaction show the formation of endoproduct **3a**′ and exoproduct **4a**′ in almost equimolar amount. After approximately 2 h, product **4a**′ slowly converts into the thermodynamically favoured product **3a**′. Although the formation of product **3a″** through an [1,2]-*n*-hexyl group migration after the ring expansion was not detected, traces of both a methyl-migrated side product **5a**′ (less than 0.5%) and the olefin isomerization product **6a**′ (less than 2%) were detected. The consumption of starting material **1a**′ shows a characteristic first-order exponential decay, which has been observed for other intramolecular IDPi-catalysed reactions, for which only one substrate molecule is involved in the rate-limiting step of the reaction^30,37^. Additionally, the catalyst concentration remained constant, and no substantial chemical shift changes were observed during the reaction in the ^1^H and ^13^C NMR spectra. The reaction order of catalyst **2d** was determined with variable time normalization analysis^38,39^ from ^1^H NMR concentration profiles and was found to be first order as well (Supplementary Information has further details). Reactions performed at five different temperatures ranging from 15 °C to 55 °C enabled us to determine the thermodynamic parameters of the reaction with the Eyring equation. An enthalpy of activation $\triangle {H}^{\ddagger }$ = 12.5 ± 0.5 kcal mol^−1^, a negative entropy of activation $\triangle {S}^{\ddagger }$ = −30.3 ± 1.6 kcal mol^−1^ K^−1^ and a free energy of activation $\triangle {G}^{\ddagger }$_298K_ = 21.5 ± 0.7 kcal mol^−1^ were determined (Fig. 4d).

On the basis of these experiments, we can propose a plausible reaction mechanism (Fig. 4e). Accordingly, the catalytic cycle is initiated with the protonation of olefin **1a**′ by IDPi **2d** to provide an alkyl-carbocation that is highly confined within the IDPi anion cavity as a contact ion pair (**I**), followed by an enantiodetermining five- to six-membered ring expansion, which affords ion pair **II**. We assume that the protonation of the substrate is the overall rate-limiting step of the reaction as covalent adducts have not been observed by ^31^P NMR spectroscopy during the reaction monitoring. Additionally, the negative activation entropy is in the range of various reported literature values for protonation of olefins^30,40^. Kinetic deprotonation gives an isolable exocyclic product **4a**′ that can reversibly be protonated to regenerate ion pair **II**. Finally, deprotonation from ion pair **II** furnishes the thermodynamically preferred endocyclic product **3a**′ and regenerates catalyst **2d**. Before the ring expansion, ion pair **I** could isomerize to furnish trisubstituted **6a**′ or convert to methyl-migrated ion pair **III**, which upon deprotonation, leads to side product **5a**′. Alternatively, ion pair **II** could undergo a [1,2]-*n*-hexyl shift to provide ion pair **IV**, which upon deprotonation, would lead to side product **3a**″. Similarly, ion pair **II** could undergo a [1,2]-methyl shift followed by deprotonation to provide the side products **14a**′ and **15a**′ (Supplementary Fig. 3). Although these hypothetical side reactions did not occur in the five- to six-membered ring expansion, in the corresponding four- to five-membered ring expansion, we did observe the [1,2]-methyl shift and [1,2]-*n*-hexyl shift side products.

Density functional theory calculations support the proposed mechanism and lead to a model for the origin of enantioselectivity. Our calculations (CPCM(CHCl_3_)-ωB97X-D4/def2-TZVPP//CPCM(CHCl_3_)-B3LYP-D3(BJ)/def2-SVP) (Supplementary Information has details and references) on the conversion of substrate **1c** suggest that the rate-determining step is protonation of the olefin, which has a predicted free energy barrier of 17.2 kcal mol^−1^ (Fig. 4f). The enantioselectivity-determining step is the subsequent five- to six-membered ring expansion, and the origin of enantioselectivity emerges out of a balance of non-covalent interactions within the confined chiral pocket, consistent with an induced-fit model^41,42^. Specifically, favourable dispersion interactions and C–H···O hydrogen bonds seem to play key roles (Fig. 4g; Supplementary Information has additional details)^43,44^. The lowest-energy transition state structures leading to *R* (TS_GH_, red path) and *S* (TS_CD_, blue path) products differ in free energy by 3.0 kcal mol^−1^, which is in reasonable agreement with the experimental enantiomeric ratio of 96:4 (which equates to a difference in free energy barriers ∆∆G^‡^ of approximately 1.9 kcal mol^−1^) (Supplementary Information has further discussion and other levels of theory). Note that although the barrier for the favoured ring expansion step is very small, the overall barrier from predecessor conformer **B** is slightly larger. Although we cannot rule out the inhibition of conformational equilibration owing to the catalyst architecture or non-statistical dynamic effects here (the differences in enantioselectivity in Fig. 4b are at least consistent with such effects), we note that transition structures connected directly to carbocation conformer **B** lead to a prediction of the same sense of enantioselectivity (Supplementary Information has additional discussion). Ring expansion is followed by a facile deprotonation of the cation and energetically downhill dissociation to form the final product **3c**. These results are consistent with an overall mechanism of the IDPi-catalysed rearrangement consisting of the following steps: (1) rate-determining protonation; (2) subsequent enantioselectivity-determining ring expansion; and (3) facile deprotonation to afford the final product after catalyst–substrate dissociation—a mechanistic scenario not unlike that found for terpene synthase enzymes for which the difficult chemical step is carbocation generation and stereoselectivity is controlled by the shape of the carbocation binding site^45^.

The high-acidity and confined chiral microenvironment of our IDPi catalysts enables the handling of purely aliphatic hydrocarbons in catalytic asymmetric Wagner–Meerwein shifts that proceed through unstabilized ‘classical cations’. We believe that the presented approach bears great potential for related reactions of aliphatic hydrocarbons.

## Online content

Any methods, additional references, Nature Portfolio reporting summaries, source data, extended data, supplementary information, acknowledgements, peer review information; details of author contributions and competing interests; and statements of data and code availability are available at 10.1038/s41586-023-06826-7.

### Supplementary information

Supplementary Information Supporting Information.

## Data Availability

We declare that the experimental procedures and analytical data supporting the findings of this study are available in the article and Supplementary Information. Raw and unprocessed nuclear magnetic resonance data are available from the corresponding author on reasonable request. Crystallographic data for compounds **7**, **8** and **9** are available free of charge from the Cambridge Crystallographic Data Centre under deposition numbers CCDC 2248971, CCDC 2248972 and CCDC 2248970. All computed and reported structures in the article and Supplementary Information can be found on ioChem-BD at 10.19061/iochem-bd-6-262.
