# Supplementary material for: Catalytic asymmetric cationic shifts of aliphatic hydrocarbons
Source: Nature. 2024 Jan 10;625(7994):287–92. doi: 10.1038/s41586-023-06826-7 (PMC10781632; doi:10.1038/s41586-023-06826-7)
Supplement: Supplementary file 1 — Supporting Information. [file 41586_2023_6826_MOESM1_ESM.pdf]

nature portfolio

https://doi.org/10.1038/s41586-023-06826-7

Supplementary information

Catalytic asymmetric cationic shifts of aliphatic hydrocarbons

In the format provided by the
authors and unedited

Supporting Information

Catalytic Asymmetric Cationic Shifts of Aliphatic Hydrocarbons

Vijay N. Wakchaure[1], William DeSnoo[2], Croix J. Laconsay[2], Markus Leutzsch[1], Nobuya Tsuji[3], Dean J. Tantillo[2*] & Benjamin List[1,3*]

[1]Max-Planck-Institut für Kohlenforschung, Kaiser-Wilhelm-Platz 1, 45470 Mülheim an der Ruhr, Germany.

[2]Department of Chemistry, University of California, Davis, California 95616, United States.

[3]Institute for Chemical Reaction Design and Discovery (WPI-ICReDD), Hokkaido University, Sapporo 001-0021, Japan.

*e-mail: list@kofo.mpg.de (B.L.); djtantillo@ucdavis.edu (D.J.T)

Table of Contents

General information S2
1. Synthesis of catalysts S3
   1a. Synthesis of *i*IDP catalyst (**2b**) S3
   1b. Synthesis of IDPi catalyst (**2f** and **2g**) S4
2. Substrate synthesis S8
   2a. General procedure 1 (GP1) S9
   2b. General procedure 2 (GP2) S10
   2c. General procedure 3 (GP3) S10
3. Reaction development S25
   3a. General procedure for the Wagner–Meerwein shift (GP4) S27
   3b. Enantioselective total synthesis of (–)-herbertene S35
   3c. ^{13}C Labelled exocyclic olefin isomerization S36
   3d. Isolation of olefin isomerized starting material (**6a**) S37
4. NMR kinetics and mechanistic studies S38
   4a. ^{13}C NMR reaction monitoring S38
   4b. ^{1}H NMR reaction monitoring S39
   4c. Determination of catalyst order S41
   4d. Eyring analysis S43
5. Determination of the absolute configuration of products S47
6. Crystallographic data S49
7. Computational studies S53
8. Computational details on %V_{Bur} S63
9. Copies of NMR spectra S75
10. Copies of GC traces S179
11 References S201

General information

Unless otherwise specified, all reagents were purchased from commercial suppliers and used without further purification. All solvents used in the reactions were distilled from appropriate drying agents prior to use. Dry argon was purchased from Air Liquide with >99.5% purity. Thin layer chromatography (TLC) was performed using silica gel pre-coated polyester sheets (POLYGRAM® SIL G/UV_{254}, 0.2 mm silica gel 60 with fluorescent indicator; Macherey-Nagel) which were visualized by irradiation with UV light (λ = 254nm) and/or $KMnO_4$, phosphomolybdic acid (PMA) stain. Column chromatography was performed on Merck silica gel (60, particle size 0.040–0.063 mm) using technical grade solvents. Nomenclature follows the suggestions proposed by the computer program ChemDraw Professional (20.1.1.125) of PerkinElmer®. All reported yields refer to spectroscopically and chromatographically pure compounds unless otherwise specified. ^{1}H, ^{13}C, ^{19}F, and ^{31}P Nuclear magnetic resonance (NMR) spectra were recorded on a Bruker Avance NEO 600 or Avance III 500 MHz NMR spectrometer in suitable deuterated solvents. Proton chemical shifts are reported in ppm (δ) relative to the solvent resonance employed as the internal standard ($CDCl_3$ δ 7.26 ppm). Data are reported as follows: chemical shift, multiplicity (s = singlet, d = doublet, t = triplet, q = quartet, p = pentet, s = sextet, h = heptet, m = multiplet, br = broad), coupling constants (Hz) and integration. ^{13}C chemical shifts are reported in ppm with the solvent resonance as the internal standard ($CDCl_3$ δ 77.16 ppm). All spectra were recorded at 298 K unless otherwise noted and processed with MestreNova 14.2.3. High resolution mass spectrometry (HRMS) was performed on a Finnigan MAT 95 (EI) or Bruker APEX III FTMS (7 T magnet, ESI). The ionization method and mode of detection employed is indicated for the respective experiment. Specific rotations were determined with an Autopol IV polarimeter (Rudolph Research Analytical) at 589 nm and 25 °C. Data are reported as follows: $[\alpha]_{\lambda}^{temp}$, concentration (*c* in g/100 mL), and solvent. Preparative High Performance Liquid Chromatography (prep-HPLC) was performed on a Shimadzu LC-20AP (SIL-20A HT autosampler, DGU-20A 3R degasser, CTO-20AC column over, SPD-20A UV/Vis detector, FRC-10A fraction collector, CBM-20A controller, LC-20AP pump). All solvents used were HPLC-grade solvents purchased from Sigma-Aldrich. Enantiomeric ratios (er) were determined by Gas chromatography (GC) analyses on an Agilent Technologies 6890N, Agilent Technologies 6890, Agilent Technologies 7890A, Agilent Technologies 7890B (split-mode capillary injection system, flame ionization detector (FID), hydrogen or helium carrier gas) employing a chiral stationary phase column specified in the individual experiment, by comparing the samples with the appropriate racemic mixtures.

1. Synthesis of catalysts

1a. Synthesis of *i*IDP catalysts (2b):

N-((11bS)-4-((2,6-bis(4-(tert-butyl)phenyl)-4-oxidodinaphtho[2,1-d:1',2'-f][1,3,2]dioxaphosphepin-4-yl)amino)-2,6-bis(4-(tert-butyl)phenyl)-4l5-dinaphtho[2,1-d:1',2'-f][1,3,2]dioxaphosphepin-4-ylidene)-1,1,1-trifluoromethanesulfonamide (2b): Synthesis of the *i*IDP catalyst **2b** was adopted from literature[1]. Purification by silica gel column chromatography (eluent: 15–35% Et_2O in *n*-pentane) followed by the acidification in CH_2Cl_2 with pre-acidified Dowex and drying *in vacuo* afforded the desired compound as a white solid (161.0 mg, 64% yield).

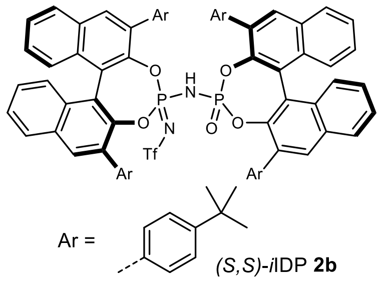

*(S,S)-i*IDP **2b**

^{1}H NMR (501 MHz, $CDCl_3$) δ 8.14–8.03 (m, 4H), 8.01–7.94 (m, 2H), 7.77–7.69 (m, 3H), 7.64 (s, 1H), 7.62–7.50 (m, 6H), 7.48 (s, 1H), 7.40–7.28 (m, 9H), 7.18 (d, *J* = 8.4 Hz, 2H), 6.83 (d, *J* = 8.4 Hz, 2H), 6.67 (d, *J* = 8.4 Hz, 2H), 6.53 (dd, *J* = 8.4, 6.3 Hz, 4H), 1.30 (s, 9H), 1.28 (s, 9H), 1.05 (s, 9H), 0.97 (s, 9H).

^{13}C NMR (126 MHz, $CDCl_3$) δ 151.1, 150.7, 150.5, 150.1, 145.0, 144.9, 144.6, 144.5, 144.35, 144.27, 143.0, 142.9, 134.7, 134.7, 134.2, 134.14, 134.05, 134.0, 133.32, 133.29, 133.22, 133.19, 132.9, 132.8, 132.20, 132.17, 132.06, 132.02, 132.0, 131.7, 131.0, 130.4, 130.31, 130.17, 129.2, 129.1, 128.89, 128.86, 128.8, 128.70, 128.66, 127.7, 127.33, 127.29, 127.04, 127.98, 126.9, 126.8, 126.5, 126.4, 126.0, 125.7, 124.91, 124.88, 124.3, 123.50, 123.48, 123.46, 123.44, 122.40, 122.38, 122.14, 122.12, 34.7, 34.6, 34.5, 34.42, 31.39, 31.3, 31.24, 31.22. (other signals not detected or observed).

^{31}P NMR (203 MHz, $CDCl_3$) δ –1.3 (d, *J* = 111.6 Hz), –7.7 (d, *J* = 111.6 Hz).

^{19}F NMR (471 MHz, $CDCl_3$) δ –79.0.

HRMS *m/z* (ESI): calcd. for $C_{81}H_{72}F_3N_2O_7P_2S$ $[M–H]^-$: 1335.4498; found: 1335.4493.

*Note: IDP **2a** was prepared following the known reported procedure*[2].

1b. Synthesis of IDPi catalysts (2f and 2g):

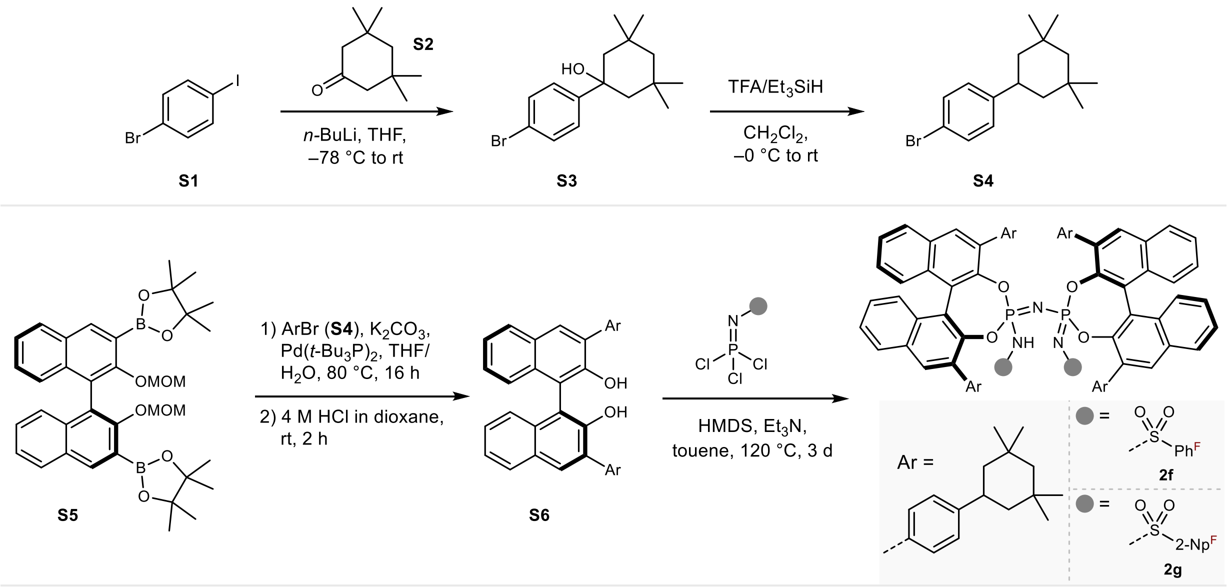

*Note: IDPi **2c**[3], IDPi **2d**[4,5] and IDPi **2e**[6] were prepared following the known reported procedure.*

1-(4-bromophenyl)-3,3,5,5-tetramethylcyclohexan-1-ol (S3): The synthesis is a modified literature procedure for a related compound[7]. *n*-BuLi (7.42 mL of a 2.5 M solution in *n*-hexane, 18.55 mmol, 1.05 equiv.) was slowly added to a solution of 1-bromo-4-iodobenzene (**S1**, 5.00 g, 17.67 mmol, 1.0 equiv.) in THF (75 mL) at –78 °C, and the resulting pale orange suspension was stirred for 1 h at that temperature. 3,3,5,5-tetramethylcyclohexanone (**S2,** 3.35 mL, 19.44 mmol, 1.1 equiv.) was slowly added, the cooling bath was removed and the mixture was allowed to warm to room temperature (rt). After 15 h, the obtained orange-brown suspension was cooled in an ice/water bath, and sat. aq. NH_4Cl (50 mL) was slowly added. The layers were separated and the aq. layer was extracted with Et_2O (3x50 mL), the combined organic layers were washed with sat. aq. NaCl (50 mL), dried over Na_2SO_4, filtered and the solvents removed under reduced pressure. Purification by flash column chromatography on silica (eluent: 2–3% EtOAc in *iso*hexane). Upon evaporation of the solvent under reduced pressure, the corresponding **S3** was obtained as a white solid (4.7 g, 85% yield).

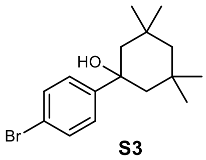

S3

^{1}H NMR (501 MHz, $CDCl_3$) δ 7.47–7.42 (m, 2H), 7.40–7.35 (m, 2H), 1.67–1.54 (m, 4H), 1.49 (dt, J = 13.9, 2.0 Hz, 1H), 1.32 (s, 7H), 1.20 (d, J = 13.8 Hz, 1H), 0.95 (s, 6H).

^{13}C NMR (126 MHz, $CDCl_3$) δ 150.1, 131.3, 126.6, 120.6, 76.3, 51.6, 50.3, 36.6, 31.8, 28.8.

HRMS *m/z* (GC-EI): calcd. for $C_{16}H_{23}OBr$ $[M]^+$: 310.0927; found: 310.0926.

1-bromo-4-(3,3,5,5-tetramethylcyclohexyl)benzene (S4): The synthesis is a modified literature procedure for a related compound[7]. Alcohol **S3** (4.70 g, 15.10 mmol, 1.0 equiv.) was dissolved in anhydrous CH_2Cl_2 (70.0 mL). After cooling down to 0 °C, was added sequentially Et_3SiH (6.03 mL, 37.75 mmol, 2.5 equiv.) and CF_3CO_2H (12.14 mL, 158.55 mmol, 10.5 equiv.). The reaction was stirred further 14 h warming up slowly in the cold bath to rt. The reaction mixture was slowly poured into a stirred sat. aq. $NaHCO_3$ (200 mL) and extracted with CH_2Cl_2 (3x100 mL). The combined CH_2Cl_2 layers dried over Na_2SO_4, filtered and the solvents removed under reduced pressure. Purification by flash column chromatography on silica (eluent: 100% *n*-pentane). Upon evaporation of the solvent under reduced pressure, the corresponding **S4** was obtained as a white solid (4.1 g, 92% yield).

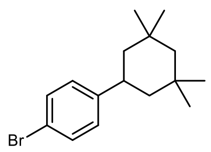

S4

^{1}H NMR (501 MHz, $CDCl_3$) δ 7.40 (d, J = 8.4 Hz, 1H), 7.10 (d, J = 8.4 Hz, 1H), 2.82 (tt, J = 12.7, 3.1 Hz, 1H), 1.60–1.47 (m, 2H), 1.35–1.28 (m, 1H), 1.20 (t, J = 12.8 Hz, 2H), 1.13–1.09 (s, 7H), 0.93 (s, 6H).

^{13}C NMR (126 MHz, $CDCl_3$) δ 146.8, 131.4, 129.0, 119.5, 51.9, 47.5, 36.6, 35.5, 32.1, 27.5.

HRMS *m/z* (GC-EI): calcd. for $C_{16}H_{23}Br$ $[M]^+$: 294.0981; found: 294.0977.

(*S*)-3,3'-bis(4-(3,3,5,5-tetramethylcyclohexyl)phenyl)-[1,1'-binaphthalene]-2,2'-diol (S6): To a 100 mL Schlenk flask under Ar and equipped with a magnetic stirring bar, (*S*)-MOM-BINOL-Bpin ester[8] (**S5,** 2.60 g, 4.15 mmol, 1.0 equiv.) was added followed by 1-bromo-4-(3,3,5,5-tetramethylcyclohexyl)-benzene (**S4,** 3.31 g, 11.21 mmol, 2.7 equiv.) and K_2CO_3 (2.86 g, 20.75 mmol, 5.0 equiv.). The solids were suspended in 50 mL THF/H_2O (4:1, v/v). After degassing the reaction mixture, Pd(*t*-$Bu_3P)_2$ (106 mg, 0.21 mmol, 5 mol%) was added and the reaction mixture was heated to 80 °C and stirred for 16 h. The reaction was then cooled to rt, acidified with 1M HCl and extracted with CH_2Cl_2 (3x50 mL). The combined organic layers were dried over Na_2SO_4, filtered and evaporated under reduced pressure. HCl in dioxane (15 mL, 4 M) was added to the dried crude reaction mixture and stirred at rt for 2 h. Following removal of the solvent under reduced pressure, the compound was directly purified by flash column chromatography on silica (eluent: 3–5% EtOAc in *iso*-hexanes). Upon evaporation of the solvent under reduced pressure, the corresponding BINOL **S6** was obtained as a white solid (2.8 g, 94% yield).

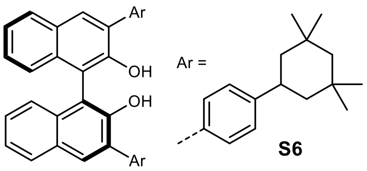

S6

^{1}H NMR (501 MHz, $CDCl_3$) δ 8.02 (s, 2H), 7.92 (d, J = 8.4 Hz, 2H), 7.67 (d, J = 8.3 Hz, 4H), 7.42–7.35 (m, 6H), 7.34–7.27 (m, 2H), 7.25–7.16 (m, 2H), 2.95 (tt, J = 12.7, 3.1 Hz, 2H), 1.71–1.58 (m, 4H), 1.39–1.27 (m, 6H), 1.18–1.13 (s, 14H), 0.97 (s, 12H).

^{13}C NMR (126 MHz, $CDCl_3$) δ 150.3, 147.4, 135.0, 133.0, 131.3, 130.8, 129.7, 129.6, 128.5, 127.4, 127.3, 124.5, 124.3, 112.6, 52.1, 47.6, 36.9, 35.6, 32.1, 27.5.

HRMS *m/z* (ESI): calcd. for $C_{52}H_{57}O_2$ $[M–H]^-$: 713.4363; found: 713.4364.

(*S*,*S*)-IDPi (2f): In a 25 mL flame dried Schlenk flask under Ar, (*S*)-3,3'-bis(4-(3,3,5,5-tetramethylcyclohexyl)phenyl)-[1,1'-binaphthalene]-2,2'-diol **(S6)** (456 mg, 0.63 mmol, 1.0 equiv.) was dissolved in toluene (7 mL), [(perfluorophenyl)sulfonyl]phosphorimidoyl trichloride[3] (256 mg, 0.67 mmol, 1.05 equiv.) and then triethylamine (0.77 g, 1.06 mL, 7.66 mmol, 12.0 equiv.) was added at rt. The mixture was stirred at rt for 1 h and then hexamethyldisilazane (51.5 mg, 66.6 μL, 0.319 mmol, 0.5 equiv.) was added. After being stirred at rt for additional 10 min, the mixture was heated to 120 °C for 3 d. The reaction mixture was cooled to rt, diluted with CH_2Cl_2 and HCl (10% aq.). Two phases were separated and the aqueous layer was washed with CH_2Cl_2 (2x50 mL). The combined organic layer was dried with Na_2SO_4, filtered and concentrated *in vacuo*. Purification by silica gel column chromatography (5–11% Et_2O in *n*-pentane) followed by the acidification in CH_2Cl_2 with pre-acidified Dowex and drying *in vacuo* afforded the desired IDPi catalyst **2f** as a white solid (420 mg, 66% yield).

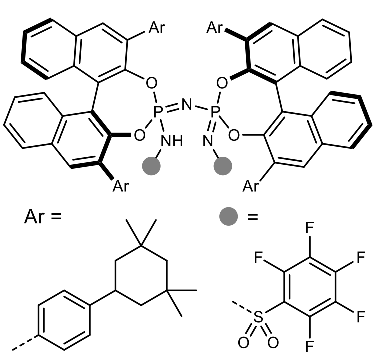

(*S*,*S*)-IDPi **2f**

^{1}H NMR (600 MHz, $CDCl_3$) δ 8.16 (d, *J* = 8.2 Hz, 2H), 8.00 (s, 2H), 7.95 (d, *J* = 8.4 Hz, 2H), 7.86 (s, 2H), 7.70 (t, *J* = 7.5 Hz, 2H), 7.54 (ddd, *J* = 8.2, 5.4, 2.6 Hz, 2H), 7.45 (t, *J* = 7.6 Hz, 2H), 7.37 (d, *J* = 8.6 Hz, 2H), 7.39–7.31 (m, 8H), 7.19 (d, *J* = 8.2 Hz, 4H), 6.81 (d, *J* = 7.8 Hz, 4H), 6.39 (d, *J* = 7.8 Hz, 4H), 6.35 (bs, 1H; OH), 2.80 (tt, *J* = 13.0, 3.1 Hz, 2H), 2.51 (t, *J* = 12.5 Hz, 2H), 1.53–1.46 (m, 4H), 1.25–1.18 (m, 6H), 1.14 (t, *J* = 12.9 Hz, 2H), 1.08–1.03 (m, 4H), 1.02 (s, 6H), 1.00 (s, 6H), 0.95 (d, *J* = 13.1 Hz, 2H), 0.85–0.74 (m, 24H), 0.63 (s, 6H), 0.62 (s, 6H), 0.48 (s, 6H).

^{13}C NMR (151 MHz, $CDCl_3$) δ 147.6, 146.7, 143.94 (dd, *J* = 260.9, 12.2 Hz), 143.93 (t, *J* = 5.1 Hz), 143.84 (d, *J* = 5.7 Hz), 143.1 (dm, *J* = 260.8 Hz), 137.05 (dt, *J* = 254.1, 15.3 Hz), 134.4, 133.9, 133.3, 132.5, 132.5, 131.8, 131.3, 131.0, 130.7, 129.3, 128.9, 128.2, 127.6, 127.4, 126.9, 126.8, 126.7, 126.6, 126.5, 123.4, 122.7, 117.7–117.3 (m), 52.1, 51.8, 48.3, 47.1, 46.8, 45.9, 36.8, 36.3, 35.4, 35.3, 35.22, 35.17, 31.94, 31.93, 31.90, 31.6, 27.44, 27.37, 27.2, 26.9. (other signals not detected or observed).

^{31}P NMR (243 MHz, $CDCl_3$) δ –9.0 (s).

^{19}F NMR (565 MHz, $CDCl_3$) δ –134.8 (d, *J* = 20.6 Hz, 4F), –146.5 (bs, 2F), –159.8 (t, *J* = 20.6 Hz, 4F).

HRMS *m/z* (ESI): calcd. for $C_{121}H_{112}F_{10}N_3O_8P_2S_2$ $[M–H]^{-}$: 1990.72444; found: 1990.7252.

(*S*,*S*)-IDPi (2g): In a 25 mL flame dried Schlenk flask under Ar, (*S*)-3,3'-bis(4-(3,3,5,5-tetramethylcyclohexyl)phenyl)-[1,1'-binaphthalene]-2,2'-diol **(S6)** (0.50 g, 0.69 mmol, 1.0 equiv.) was dissolved in toluene (2 mL), ((perfluoronaphthalen-2-yl)sulfonyl)phosphorimidoyl trichloride (344 mg, 0.73 mmol, 1.05 equiv.)[9] and then triethylamine (0.84 g, 1.17 mL, 8.39 mmol, 12.0 equiv.) was added at rt. The mixture was stirred at rt for 1 h and then hexamethyldisilazane (56.43 mg, 72.91 μL, 0.349 mmol, 0.5 equiv.) was added. After being stirred at rt for additional 10 min, the mixture was heated to 120 °C for 3 d. The reaction mixture was cooled to rt, diluted with CH_2Cl_2 and HCl (10% aq.). Two phases were separated and the aqueous layer was washed with CH_2Cl_2 (2x50 mL). The combined organic layer was dried with Na_2SO_4, filtered and concentrated *in vacuo*. Purification by silica gel column chromatography (5–13% Et_2O in *n*-pentane) followed by the acidification in CH_2Cl_2 with pre-acidified Dowex and drying *in vacuo* afforded the desired IDPi catalyst **2g** as a white solid (510 mg, 67% yield).

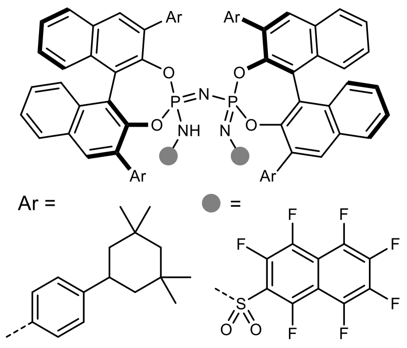

(*S*,*S*)-IDPi **2g**

^{1}H NMR (600 MHz, $CDCl_3$) δ 8.18 (d, *J* = 8.2 Hz, 2H), 7.99 (s, 2H), 7.98 (s, 2H), 7.76 (d, *J* = 8.3 Hz, 2H), 7.72–7.64 (m, 2H), 7.59 (d, *J* = 8.3 Hz, 4H), 7.42–7.33 (m, 8H), 7.12 (d, *J* = 8.6 Hz, 2H), 7.08 (d, *J* = 8.0 Hz, 2H), 7.07–7.03 (m, 2H), 6.96 (d, *J* = 8.1 Hz, 4H), 6.26 (d, *J* = 8.0 Hz, 4H), 5.37 (s, 1H), 2.88 (tt, *J* = 12.6, 2.8 Hz, 2H, 15), 2.40 (tt, *J* = 13.0, 3.1 Hz, 2H, 15'), 1.64–1.54 (m, 4H), 1.29 (t, *J* = 12.8 Hz, 2H), 1.26–1.19 (m, 4H), 1.11 (d, *J* = 13.0 Hz, 2H), 1.07 (d, *J* = 13.7 Hz, 2H), 1.03 (s, 6H), 1.04–1.00 (m, 2H)*, 1.01 (s, 6H,), 0.94 (d, *J* = 12.9 Hz, 2H), 0.85 (s, 6H), 0.83 (s, 6H), 0.80–0.71 (m, 6H), 0.70 (s, 6H), 0.64 (s, 6H), 0.55 (s, 6H), 0.45 (s, 6H). *determined after 2D NMR assigment

^{13}C NMR (151 MHz, $CDCl_3$) δ 149.59 (d, *J* = 272.2 Hz), 148.05, 146.64, 143.85 (t, *J* = 5.6 Hz), 143.66 (t, *J* = 5.1 Hz), 143.35 (dd, *J* = 260.5, 14.2 Hz), 142.08 (dm, *J* = 266.8 Hz), 141.66–139.35 (m), 140.02 (dd, *J* = 256.3, 15.8 Hz), 138.78 (dt, *J* = 256.9, 14.8 Hz), 134.52, 133.58, 133.02, 132.56, 131.72, 131.40, 130.91, 130.87, 130.40, 129.68, 129.48, 128.94, 127.81, 127.79, 127.43, 126.65, 126.55, 126.39, 126.33, 126.21, 125.92, 122.95, 122.73, 120.18 (t, *J* = 15.8 Hz), 111.81 (t, *J* = 10.5 Hz), 107.31 (dd, *J* = 16.4, 9.7 Hz), 52.10, 51.80, 47.81, 46.97, 46.96, 46.21, 36.81, 36.29, 32.00, 31.99, 31.78, 31.57, 27.50, 27.38, 27.15, 26.87.

^{31}P NMR (243 MHz, $CDCl_3$) δ –5.33.

^{19}F NMR (565 MHz, $CDCl_3$) δ –111.48 (dd, *J* = 76, 17 Hz, 2F), –132.56 (d, *J* = 19 Hz, 2F), –141.30 (dt, *J* = 76, 17 Hz, 2F), –144.32 (dt, *J* = 58, 15 Hz, 2F), –146.80 (dt, *J* = 60, 18 Hz, 2F), –150.24 (t, *J* = 18 Hz, 2F), –154.86 (t, *J* = 20 Hz).

HRMS *m/z* (ESI): calcd. for $C_{124}H_{113}F_{14}N_3O_8P_2S_2$ $[M–H]^-$: 2162.7133; found: 2162.7148.

2. Substrate synthesis

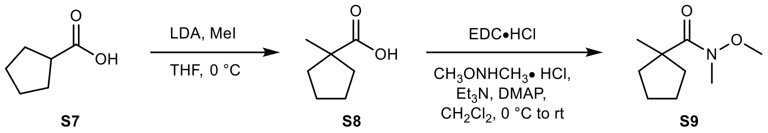

1-methylcyclopentane-1-carboxylic acid (S8): The synthesis is a modified literature procedure for a related compound[10]. To a flame dried 2-neck round-bottom flask, (*i*-Pr)$_2$NH (55.25 mL, 394.24 mmol, 2.25 equiv.) and THF (175 mL) was added under Ar atmosphere. The solution was cooled to 0 °C, and *n*-BuLi (2.5 M in hexane, 154.89 mL, 387.23 mmol, 2.21 equiv.) was slowly added. Stirred for 5 minutes, and the mixture was warmed up to rt slowly. The reaction mixture was stirred for another 15 minutes. Then the mixture was cooled to 0 °C again. A solution of cyclopentanecarboxylic acid (**S7,** 19.05 mL, 175.22 mmol, 1.0 equiv.) in THF (50 mL) was slowly added to the reaction mixture, and the resulting mixture was slowly warmed to rt, then stirred at 45 °C for 1 h. Then the reaction mixture was cooled to 0 °C, and CH_3I (13.63 mL, 219.02 mmol, 1.25 equiv.) was slowly added. The cooling bath was removed and the mixture was allowed to warm to rt. After 15 h, the reaction mixture was cooled in an ice/water bath, and 10% HCl (150 mL) was slowly added. The layers were separated and the aq. layer was extracted with Et_2O (3x100 mL), the combined organic layers were washed with sat. aq. NaCl (100 mL), dried over Na_2SO_4, filtered and the solvents removed under reduced pressure. Crude 1-methylcyclopentane-1-carboxylic acid **S8** was obtained as a dark-amber liquid (22.0 g, 98% yield) and was subsequently used for the next step without further purification.

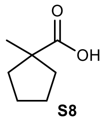

^{1}H NMR (501 MHz, $CDCl_3$) δ 11.59 (s, 1H), 2.19–2.10 (m, 2H), 1.76–1.64 (m, 4H), 1.55–1.45 (m, 2H), 1.27 (s, 3H).

^{13}C NMR (126 MHz, $CDCl_3$) δ 185.3, 49.2, 38.1, 25.4, 24.8.

HRMS *m/z* (GC-EI): calculated for $C_7H_{12}O_2$ $[M]^+$: 128.0832; found: 128.0831.

N-methoxy-N,1-dimethylcyclopentane-1-carboxamide (S9): The synthesis is a modified literature procedure for a related compound[11]. Crude 1-methylcyclopentane-1-carboxylic acid (**S8,** 10.00 g, 78.02 mmol, 1.0 equiv.) was dissolved in CH_2Cl_2 (250 mL). After cooling down to 0 °C, was added sequentially *N*,O-dimethylhydroxylamine hydrochloride (10.27 g, 105.33 mmol, 1.35 equiv.), N-(3-Dimethylaminopropyl)-N′-ethylcarbodiimide hydrochloride (20.19 g, 105.33 mmol, 1.35 equiv.) and 4-(Dimethylamino)pyridine (0.47 g, 3.90 mmol, 5 mol%) under an Ar atmosphere. Then triethylamine (38.06 mL, 273.07 mmol, 3.5 equiv.) was slowly added, so that the temperature was kept below 5 °C. The reaction was stirred further 14 h warming up slowly in the cold bath to rt. Then the reaction mixture cooled to 0 °C, and 1M HCl solution (100 mL) was slowly added. The organic phase was separated and then washed with 1M HCl solution (2x75 mL), water (2x75 mL),

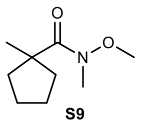

sat. aq. $NaHCO_3$ (2x75 mL), and sat. aq. NaCl (100 mL). The organic layer was dried with Na_2SO_4, filtered and concentrated *in vacuo*. Purification by silica gel flash column chromatography (10–50% Et_2O in *n*-pentane). Upon evaporation of the solvent under reduced pressure, the corresponding Weinreb amide **S9** was obtained as a colorless liquid (12.0 g, 90% yield).

^{1}H NMR (501 MHz, $CDCl_3$) δ 3.67 (s, 3H), 3.18 (s, 3H), 2.15–2.03 (m, 2H), 1.71–1.53 (m, 6H), 1.24 (s, 3H).

^{13}C NMR (126 MHz, $CDCl_3$) δ 179.7, 60.6, 50.1, 37.2, 33.7, 24.5, 24.4.

HRMS *m/z* (ESI): calculated for $C_9H_{18}NO_2$ $[M+H]^+$: 172.1333; found: 172.1332.

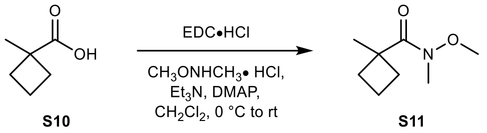

N-methoxy-N,1-dimethylcyclobutane-1-carboxamide (S11): The title compound was prepared

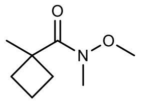

S11

according to the procedure of Weinreb amide (**S9)** from 1-methylcyclobutane-1-carboxylic acid (**S10,** 43.80 mmol, 1.0 equiv.). Purification by silica gel flash column chromatography (10–20% Et_2O in *n*-pentane). Upon evaporation of the solvent under reduced pressure, the corresponding Weinreb amide **S11** was obtained as a colorless liquid (6.15 g, 89% yield).

^{1}H NMR (501 MHz, $CDCl_3$) δ 3.62 (s, 3H), 3.12 (s, 3H), 2.49–2.37 (m, 2H), 2.01–1.88 (m, 1H), 1.83–1.74 (m, 2H), 1.72–1.61 (m, 1H), 1.40 (s, 3H).

^{13}C NMR (126 MHz, $CDCl_3$) δ 179.2, 60.4, 44.0, 32.9, 31.7, 22.9, 14.7.

HRMS *m/z* (GC-EI): calculated for $C_8H_{15}NO_2$ $[M]^+$: 157.1098; found: 157.1097.

2a. General procedure 1 [GP1 (S12a–S12n)]:

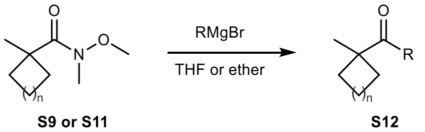

To a solution of Weinreb amide (**S9** or **S11,** 5.0 mmol, 1.0 equiv.) in THF (15 mL) was slowly added Grignard reagent (alkylmagnesium bromide or alkylmagenesium chloride in THF or Et_2O, 10.0 mmol, 2.0 equiv.) at 0 °C under Ar atmosphere. The reaction was stirred further 15 h warming up slowly in the cold bath to rt. Then the reaction mixture was cooled in an ice/water bath, and sat. aq. NH_4Cl solution (50 mL) was slowly added. The layers were separated and the aqueous layer was extracted with diethyl ether (3x20 mL). The combined organic layers were collected and dried over Na_2SO_4, filtered and concentrated under reduced pressure. The crude product was purified by flash column chromatography (eluent: 1–3% Et_2O in *n*-pentane unless noted). Upon evaporation of the solvent under reduced pressure, the corresponding ketone **S12** was obtained as a colorless liquid.

2b. General procedure 2 [GP2 (S12o–S12r)]:

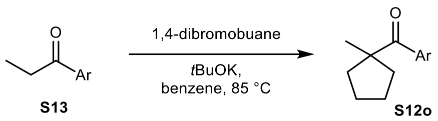

The synthesis is a modified literature procedure for a related compound[12]. A solution of ketone (**S13,** 5.0 mmol, 1.0 equiv.) in benzene (2 mL) and 1,4-Dibromobutane (0.66 mL, 5.5 mmol, 1.1 equiv.) was added sequentially to a magnetically stirred suspension of *t*BuOK (1.40 g, 12.5 mmol, 2.5 equiv.) in benzene (20 mL) under Ar, and the mixture was heated at 85 °C for a period of 16 h. After being cooled to rt, filtered through a pad of Celite, washed with diethyl ether. Followed by removal of solvent under reduced pressure. The crude product was purified by flash column chromatography

2c. General procedure 3 [GP3 (1a–1w)]:

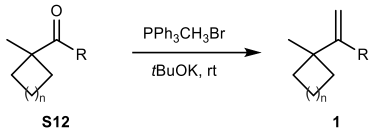

To a solution of triphenylmethylphosphonium bromide (1.50 g, 4.2 mmol, 1.4 equiv.) in THF (20 mL), *t*BuOK (0.40 g, 3.6 mmol, 1.2 equiv.) was added slowly at 0 °C. The reaction mixture was warmed to rt and stirred for 2 h. To the suspension was added corresponding ketone (**S12,** 3.0 mmol, 1.0 equiv.) at rt. The reaction mixture was stirred for 15 h at rt. Filtration through a pad of Celite. Followed by removal of solvent under reduced pressure. The crude product was purified by flash column chromatography (eluent: 100% *n*-pentane unless noted). Upon evaporation of the solvent under reduced pressure, the corresponding olefin **1** was obtained as a colorless liquid.

1-(1-methylcyclopentyl)heptan-1-one (S12a): Prepared according to GP1, 0.75 g, colorless liquid, 77%.

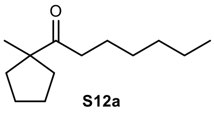

^{1}H NMR (501 MHz, $CDCl_3$) δ 2.46 (t, *J* = 7.4 Hz, 2H), 2.08–1.96 (m, 2H), 1.74–1.52 (m, 6H), 1.43–1.34 (m, 2H), 1.32–1.24 (m, 6H), 1.19 (s, 1H), 0.88 (t, *J* = 7.0 Hz, 3H).

^{13}C NMR (126 MHz, $CDCl_3$) δ 215.4, 55.8, 37.5, 36.4, 31.9, 29.2, 25.2, 24.6, 24.4, 22.7, 14.2.

HRMS *m/z* (GC-EI): calculated for $C_{13}H_{24}O$ $[M]^+$: 196.1821; found: 196.1821.

1-methyl-1-(oct-1-en-2-yl)cyclopentane (1a): Prepared according to GP3, 0.54 g, colorless liquid, 93%.

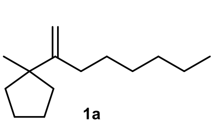

^{1}H NMR (501 MHz, $CDCl_3$) δ 4.84–4.80 (m, 1H), 4.76–4.68 (m, 1H), 2.02 (t, *J* = 8.2 Hz, 2H), 1.71–1.61 (m, 6H), 1.50–1.41 (m, 4H), 1.37–1.27 (m, 6H), 1.04 (s, 3H), 0.93–0.86 (m, 3H).

^{13}C NMR (126 MHz, $CDCl_3$) δ 157.9, 105.8, 48.8, 37.9, 32.4, 32.1, 29.7, 29.3, 26.5, 23.8, 22.8, 14.3.
HRMS *m/z* (GC-CI): calculated for $C_{14}H_{27}$ $[M+H]^+$: 195.2108; found: 195.2107.

1-methyl-1-(oct-1-en-2-yl-1-^{13}*C*)cyclopentane (1a'): Prepared according to GP3, utilizing (methyl-^{13}*C*)triphenylphosphonium iodide[13] (1.70 g, 4.2 mmol, 1.4 equiv.), 0.50 g, colorless liquid, 86%.

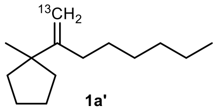

1a'

^{1}H NMR (600 MHz, $CDCl_3$) δ 4.82 (ddt, $^{1}J_{CH}$ = 154.9, J_H = 1.4, 0.8 Hz, 1H), 4.70 (dq, $^{1}J_{CH}$ = 153.5, J_H = 1.4 Hz, 1H), 2.07–1.99 (m, 2H), 1.72–1.60 (m, 6H), 1.51–1.39 (m, 4H), 1.38–1.25 (m, 6H), 1.04 (s, 3H), 0.93–0.86 (m, 3H).
^{13}C NMR (151 MHz, $CDCl_3$) δ 157.9 (d, *J* = 72.3 Hz), 105.8, 48.8 (d, *J* = 1.2 Hz), 37.9 (d, *J* = 2.7 Hz), 32.4 (d, *J* = 1.9 Hz), 32.1, 29.7, 29.3 (d, *J* = 3.4 Hz), 26.5 (d, *J* = 2.5 Hz), 23.7, 22.8, 14.3.
HRMS *m/z* (GC-EI): calculated for $C_{13}H_{26}{}^{13}C$ $[M]^+$: 195.2060; found: 195.2062.

1-(1-methylcyclopentyl)undecan-1-one (S12b): Prepared according to GP1, 0.96 g, colorless liquid, 76%.

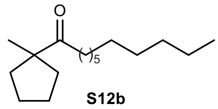
S12b

^{1}H NMR (501 MHz, $CDCl_3$) δ 2.46 (t, *J* = 7.4 Hz, 2H), 2.08–1.97 (m, 2H), 1.73–1.50 (m, 6H), 1.44–1.34 (m, 2H), 1.31–1.22 (m, 14H), 1.18 (s, 3H), 0.88 (t, *J* = 6.9 Hz, 3H).
^{13}C NMR (126 MHz, $CDCl_3$) δ 215.4, 55.8, 37.5, 36.5, 32.0, 29.7, 29.7, 29.6, 29.5, 25.3, 24.7, 24.4, 22.8, 14.3.
HRMS *m/z* (GC-EI): calculated for $C_{17}H_{32}O$ $[M]^+$: 252.2447; found: 252.2447.

1-(dodec-1-en-2-yl)-1-methylcyclopentane (1b): Prepared according to GP3, 0.64 g, colorless liquid, 86%.

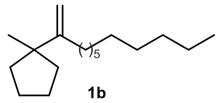
1b

^{1}H NMR (501 MHz, $CDCl_3$) δ 4.82 (s, 1H), 4.73–4.68 (m, 1H), 2.06–1.99 (m, 2H), 1.71–1.60 (m, 6H), 1.50–1.41 (m, 4H), 1.35–1.24 (m, 14H), 1.05 (s, 3H), 0.89 (t, *J* = 6.9 Hz, 3H).
^{13}C NMR (126 MHz, $CDCl_3$) δ 157.8, 105.8, 48.8, 38.0, 37.9, 32.5, 32.1, 30.0, 29.9, 29.8, 29.5, 29.3, 26.5, 23.8, 22.9, 14.3.
HRMS *m/z* (GC-EI): calculated for $C_{18}H_{34}$ $[M]^+$: 250.2656; found: 250.2655.

1-(1-methylcyclopentyl)pentan-1-one (S12c): Prepared according to GP1, used Et_2O as solvent instead of THF, 0.69 g, colorless liquid, 82%.

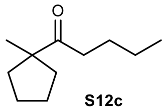
S12c

^{1}H NMR (501 MHz, $CDCl_3$) δ 2.47 (t, *J* = 7.3 Hz, 2H), 2.07–1.96 (m, 2H), 1.72–1.57 (m, 4H), 1.59–1.51 (m, 2H), 1.45–1.35 (m, 2H), 1.34–1.26 (m, 2H), 1.19 (s, 3H), 0.91 (t, *J* = 7.3 Hz, 3H).

^{13}C NMR (126 MHz, $CDCl_3$) δ 215.3, 55.7, 37.2, 36.4, 26.5, 25.2, 24.6, 22.6, 14.1.

HRMS *m/z* (GC-EI): calculated for $C_{11}H_{20}O$ $[M]^+$: 168.1508; found: 168.1508.

1-(hex-1-en-2-yl)-1-methylcyclopentane (1c): Prepared according to GP3, used Et_2O as solvent instead of THF, 0.34 g, colorless liquid, 68%.

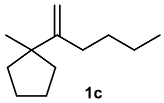

1c

^{1}H NMR (501 MHz, $CDCl_3$) δ 4.83–4.82 (m, 1H), 4.71–4.70 (m, 1H), 2.07–2.00 (m, 2H), 1.71–1.61 (m, 6H), 1.50–1.42 (m, 4H), 1.39–1.32 (m, 2H), 1.05 (s, 3H), 0.93 (t, *J* = 7.3 Hz, 3H).

^{13}C NMR (126 MHz, $CDCl_3$) δ 157.8, 105.9, 48.8, 37.9, 32.1, 31.6, 26.5, 23.8, 23.0, 14.3.

HRMS *m/z* (GC-EI): calculated for $C_{12}H_{22}$ $[M]^+$: 166.1717; found: 162.1716.

1-(1-methylcyclopentyl)butan-1-one (S12d): Prepared according to GP1, used Et_2O as solvent instead of THF, 0.59 g, colorless liquid, 77%.

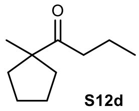

S12d

^{1}H NMR (501 MHz, $CDCl_3$) δ 2.45 (t, *J* = 7.2 Hz, 2H), 2.05–1.95 (m, 2H), 1.71–1.55 (m, 6H), 1.43–1.35 (m, 2H), 1.18 (s, 3H), 0.90 (t, *J* = 7.4 Hz, 3H).

^{13}C NMR (126 MHz, $CDCl_3$) δ 215.2, 55.7, 39.4, 36.4, 25.2, 24.6, 17.7, 14.0.

HRMS *m/z* (GC-EI): calculated for $C_{10}H_{18}O$ $[M]^+$: 154.1352; found: 154.1352.

1-methyl-1-(pent-1-en-2-yl)cyclopentane (1d): Prepared according to GP3, used Et_2O as solvent instead of THF, 0.42 g, colorless liquid, 92%.

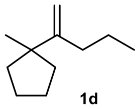

1d

^{1}H NMR (501 MHz, $CDCl_3$) δ 4.83 (s, 1H), 4.70 (q, *J* = 1.5 Hz, 1H), 2.04–1.98 (m, 2H), 1.72–1.61 (m, 6H), 1.56–1.40 (m, 4H), 1.05 (s, 3H), 0.95 (t, *J* = 7.3 Hz, 3H).

^{13}C NMR (126 MHz, $CDCl_3$) δ 157.6, 105.9, 48.7, 37.9, 34.6, 26.5, 23.7, 22.3, 14.4.

HRMS *m/z* (GC-EI): calculated for $C_{11}H_{20}$ $[M]^+$: 152.1558; found: 152.1559.

1-(1-methylcyclopentyl)propan-1-one (S12e): Prepared according to GP1, used Et_2O as solvent instead of THF, 0.60 g, colorless liquid, 86%.

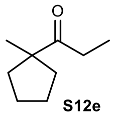

S12e

^{1}H NMR (501 MHz, $CDCl_3$) δ 2.50 (q, *J* = 7.3 Hz, 2H), 2.09–1.97 (m, 2H), 1.73–1.58 (m, 4H), 1.46–1.36 (m, 2H), 1.20 (s, 3H), 1.05 (t, *J* = 7.3 Hz, 3H).

^{13}C NMR (126 MHz, $CDCl_3$) δ 215.9, 55.6, 36.6, 30.5, 25.2, 24.7, 8.6.

HRMS *m/z* (GC-EI): calculated for $C_9H_{16}O$ $[M]^+$: 140.1197; found: 140.1195.

1-(but-1-en-2-yl)-1-methylcyclopentane (1e): Prepared according to GP3, used Et_2O as solvent instead of THF, 0.30 g, colorless liquid, 73%.

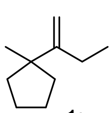

1e

^{1}H NMR (501 MHz, $CDCl_3$) δ 4.82 (s, 1H), 4.70 (s, 1H), 2.08 (q, *J* = 7.4 Hz, 2H), 1.71–1.60 (m, 6H), 1.49–1.38 (m, 2H), 1.10–0.96 (m, 6H).

^{13}C NMR (126 MHz, $CDCl_3$) δ 159.3, 105.1, 48.8, 38.0, 26.5, 24.9, 23.7, 13.4.

HRMS *m/z* (GC-EI): calculated for $C_{10}H_{18}$ $[M]^+$: 138.1403; found: 138.1403.

4-methyl-1-(1-methylcyclopentyl)pentan-1-one (S12f): Prepared according to GP1, 0.77 g, colorless liquid, 85%.

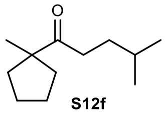

S12f

^{1}H NMR (501 MHz, $CDCl_3$) δ 2.49–2.44 (m, 2H), 2.08–1.96 (m, 2H), 1.72–1.58 (m, 4H), 1.59–1.35 (m, 5H), 1.19 (s, 3H), 0.89 (d, J = 6.5 Hz, 6H).

^{13}C NMR (126 MHz, $CDCl_3$) δ 215.5, 55.9, 36.5, 35.5, 33.4, 27.9, 25.2, 24.7, 22.6.

HRMS *m/z* (GC-EI): calculated for $C_{12}H_{22}O$ $[M]^+$: 182.1666; found: 182.1665.

1-methyl-1-(5-methylhex-1-en-2-yl)cyclopentane (1f): Prepared according to GP3, 0.46 g, colorless liquid, 85%.

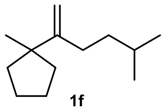

1f

^{1}H NMR (501 MHz, $CDCl_3$) δ 4.82 (s, 1H), 4.70 (q, J = 1.4 Hz, 1H), 2.09–1.99 (m, 2H), 1.72–1.63 (m, 6H), 1.61–1.52 (m, 1H), 1.48–1.41 (m, 2H), 1.39–1.32 (m, 2H), 1.05 (s, 3H), 0.91 (d, J = 6.6 Hz, 6H).

^{13}C NMR (126 MHz, $CDCl_3$) δ 158.1, 105.9, 48.9, 38.8, 38.0, 30.2, 28.4, 26.5, 23.8, 22.9.

HRMS *m/z* (GC-EI): calculated for $C_{13}H_{24}$ $[M]^+$: 180.1873; found: 180.1872.

4,4-dimethyl-1-(1-methylcyclopentyl)pentan-1-one (S12g): Prepared according to GP1, 0.73 g, colorless liquid, 75%.

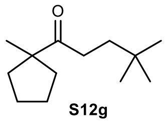

S12g

^{1}H NMR (501 MHz, $CDCl_3$) δ 2.46–2.40 (m, 2H), 2.08–1.96 (m, 2H), 1.75–1.59 (m, 4H), 1.51–1.36 (m, 4H), 1.20 (s, 3H), 0.89 (s, 9H).

^{13}C NMR (126 MHz, $CDCl_3$) δ 215.8, 56.1, 38.4, 36.5, 33.0, 30.2, 29.3, 25.3, 24.7.

HRMS *m/z* (GC-CI): calculated for $C_{13}H_{25}O$ $[M+H]^+$: 197.1897; found: 197.1899.

1-(5,5-dimethylhex-1-en-2-yl)-1-methylcyclopentane (1g): Prepared according to GP3, 0.52 g, colorless liquid, 89%.

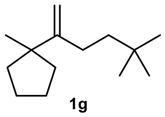

1g

^{1}H NMR (501 MHz, $CDCl_3$) δ 4.82 (s, 1H), 4.70 (q, J = 1.4 Hz, 1H), 2.05–1.95 (m, 2H), 1.71–1.59 (m, 6H), 1.51–1.41 (m, 2H), 1.39–1.32 (m, 2H), 1.05 (s, 3H), 0.90 (s, 9H).

^{13}C NMR (126 MHz, $CDCl_3$) δ 158.6, 106.0, 49.1, 44.2, 38.0, 30.6, 29.5, 27.2, 26.6, 23.8.

HRMS *m/z* (GC-CI): calculated for $C_{14}H_{25}$ $[M–H]^-$: 193.1951; found: 193.1950.

3-cyclohexyl-1-(1-methylcyclopentyl)propan-1-one (S12h): Prepared according to GP1, 0.93 g, colorless liquid, 84%.

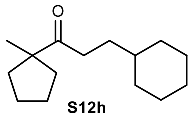

S12h

^{1}H NMR (501 MHz, $CDCl_3$) δ 2.52–2.39 (m, 2H), 2.12–1.94 (m, 2H), 1.77–1.57 (m, 9H), 1.49–1.34 (m, 4H), 1.27–1.10 (m, 7H), 0.96 – 0.81 (m, 2H).

^{13}C NMR (126 MHz, $CDCl_3$) δ 215.7, 55.9, 37.6, 36.5, 35.0, 33.4, 31.9, 26.7, 26.4, 25.2, 24.7.

HRMS *m/z* (GC-EI): calculated for $C_{15}H_{26}O$ $[M]^+$: 222.1981; found: 222.1978.

(3-(1-methylcyclopentyl)but-3-en-1-yl)cyclohexane (1h): Prepared according to GP3, 0.58 g, colorless liquid, 88%.

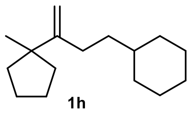

1h

^{1}H NMR (501 MHz, $CDCl_3$) δ 4.81 (s, 1H), 4.69 (q, *J* = 1.5 Hz, 1H), 2.10–1.98 (m, 2H), 1.77–1.61 (m, 11H), 1.47–1.40 (m, 2H), 1.39–1.31 (m, 2H), 1.28–1.10 (m, 4H), 1.04 (s, 3H), 0.97–0.85 (m, 2H).

^{13}C NMR (126 MHz, $CDCl_3$) δ 158.2, 105.8, 48.9, 38.1, 38.0, 37.3, 33.7, 29.7, 26.9, 26.6, 26.5, 23.8.

HRMS *m/z* (GC-EI): calculated for $C_{16}H_{28}$ $[M]^+$: 220.2183; found: 220.2185.

5-methyl-1-(1-methylcyclopentyl)hex-4-en-1-one (S12i): Prepared according to GP1, 0.69 g, colorless liquid, 71%.

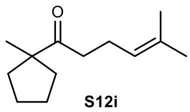

S12i

^{1}H NMR (501 MHz, $CDCl_3$) δ 5.10–5.03 (m, 1H), 2.52–2.45 (m, 2H), 2.29–2.20 (m, 2H), 2.05–1.94 (m, 2H), 1.73–1.55 (m, 10H), 1.43–1.33 (m, 2H), 1.18 (s, 3H).

^{13}C NMR (126 MHz, $CDCl_3$) δ 214.9, 132.6, 123.4, 55.7, 37.6, 36.4, 25.8, 25.3, 24.6, 23.1, 17.8.

HRMS *m/z* (GC-EI): calculated for $C_{13}H_{22}$ $[M]^+$: 194.1664; found: 194.1665.

1-methyl-1-(6-methylhepta-1,5-dien-2-yl)cyclopentane (1i): Prepared according to GP3, 0.50 g, colorless liquid, 86%.

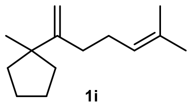

1i

^{1}H NMR (501 MHz, $CDCl_3$) δ 5.21–5.12 (m, 1H), 4.84 (s, 1H), 4.72 (q, *J* = 1.4 Hz, 1H), 2.22–2.11 (m, 2H), 2.09–1.98 (m, 2H), 1.76–1.58 (m, 12H), 1.47–1.39 (m, 2H), 1.05 (s, 3H).

^{13}C NMR (126 MHz, $CDCl_3$) δ 157.4, 131.5, 124.8, 106.1, 48.8, 37.9, 32.4, 27.8, 26.4, 25.8, 23.7, 17.8.

HRMS *m/z* (GC-EI): calculated for $C_{14}H_{24}$ $[M]^+$: 192.1873; found: 192.1872.

6-(benzyloxy)-1-(1-methylcyclopentyl)hexan-1-one (S12j): Prepared according to GP1, eluent: 2–7% Et_2O in *n*-pentane, 0.92 g, colorless liquid, 64%.

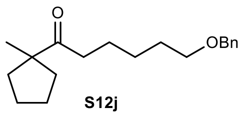

S12j

^{1}H NMR (501 MHz, $CDCl_3$) δ 7.29–7.25 (m, 4H), 7.23–7.18 (m, 1H), 4.43 (s, 2H), 3.40 (t, *J* = 6.6 Hz, 2H), 2.40 (t, *J* = 7.4 Hz, 2H), 2.10–1.84 (m, 2H), 1.72–1.46 (m, 8H), 1.37–1.23 (m, 4H), 1.11 (s, 3H).

^{13}C NMR (126 MHz, $CDCl_3$) δ 215.2, 138.8, 128.5, 127.7, 127.6, 73.0, 70.4, 55.7, 37.4, 36.4, 29.8, 26.1, 25.2, 24.6, 24.2.

HRMS *m/z* (ESI): calculated for $C_{19}H_{29}O$ $[M+H]^+$: 289.2162; found: 289.2162.

[((6-(1-methylcyclopentyl)hept-6-en-1-yl)oxy)methyl]benzene (1j): Prepared according to GP3, eluent: 1–2% Et_2O in *n*-pentane, 0.79 g, colorless liquid, 92%.

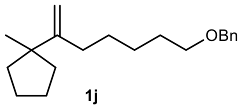

1j

^{1}H NMR (501 MHz, $CDCl_3$) δ 7.37–7.32 (m, 4H), 7.31–7.27 (m, 1H), 4.82 (s, 1H), 4.69 (q, *J* = 1.4 Hz, 1H), 4.51 (s, 2H), 3.48 (t, *J* = 6.6 Hz, 2H), 2.13–1.94 (m, 2H), 1.77–1.61 (m, 8H), 1.57–1.32 (m, 6H), 1.04 (s, 3H).

^{13}C NMR (126 MHz, $CDCl_3$) δ 157.6, 138.9, 128.5, 127.8, 127.6, 106.0, 73.0, 70.6, 48.8, 37.9, 32.3, 29.9, 29.1, 26.5, 26.5, 23.7.

HRMS *m/z* (GC-EI): calculated for $C_{20}H_{30}O$ $[M]^+$: 286.2294; found: 286.2291.

3-mesityl-1-(1-methylcyclopentyl)propan-1-one (S12k): Prepared according to GP1, 0.34 g, colorless liquid, 28%.

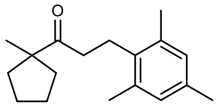

S12k

^{1}H NMR (501 MHz, $CDCl_3$) δ 6.86 (s, 2H), 2.96–2.83 (m, 2H), 2.66–2.57 (m, 2H), 2.29 (s, 6H), 2.27 (s, 3H), 2.11–1.98 (m, 2H), 1.78–1.58 (m, 4H), 1.45–1.42 (m, 2H), 1.21 (s, 3H).

^{13}C NMR (126 MHz, $CDCl_3$) δ 214.7, 136.1, 135.5, 135.2, 129.1, 55.8, 36.8, 36.5, 36.5, 25.3, 24.6, 24.3, 20.9, 19.8.

HRMS *m/z* (GC-EI): calculated for $C_{18}H_{26}O$ $[M]^+$: 258.1975; found: 258.1978.

1,3,5-trimethyl-2-(3-(1-methylcyclopentyl)but-3-en-1-yl)benzene (1k): Prepared according to GP3, eluent: 100% *n*-pentane to 1% Et_2O in *n*-pentane, 0.68 g, colorless liquid, 88%.

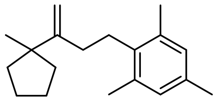

1k

^{1}H NMR (501 MHz, $CDCl_3$) δ 6.85 (s, 2H), 4.94 (s, 1H), 4.89 (q, *J* = 1.3 Hz, 1H), 2.82–2.68 (m, 2H), 2.30 (s, 6H), 2.26 (s, 3H), 2.21–2.10 (m, 2H), 1.74–1.63 (m, 6H), 1.50–1.40 (m, 2H), 1.07 (s, 3H).

^{13}C NMR (126 MHz, $CDCl_3$) δ 157.9, 136.5, 136.1, 135.2, 129.1, 106.2, 49.0, 37.9, 31.6, 29.5, 26.5, 23.8, 20.9, 19.8.

HRMS *m/z* (GC-EI): calculated for $C_{19}H_{28}$ $[M]^+$: 256.2185; found: 256.2185.

1-(1-methylcyclobutyl)heptan-1-one (S12l): Prepared according to GP1, 0.79 g, colorless liquid, 87%.

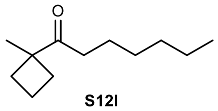

S12l

^{1}H NMR (501 MHz, $CDCl_3$) δ 2.51–2.32 (m, 4H), 2.01–1.88 (m, 1H), 1.82–1.66 (m, 3H), 1.61–1.51 (m, 2H), 1.36 (s, 3H), 1.33–1.21 (m, 6H), 0.88 (t, *J* = 6.8 Hz, 3H).

^{13}**C NMR** (126 MHz, $CDCl_3$) δ 215.0, 50.0, 36.2, 31.8, 30.4, 29.2, 24.0, 23.9, 22.7, 14.6, 14.2.
HRMS *m/z* (GC-CI): calculated for $C_{12}H_{23}$ $[M+H]^+$: 183.1744; found: 183.1743.

1-methyl-1-(oct-1-en-2-yl)cyclobutane (1l): Prepared according to GP3, 0.49 g, colorless liquid, 90%.

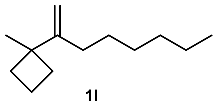

1l

^{1}**H NMR** (501 MHz, $CDCl_3$) δ 4.76–4.62 (m, 2H), 2.20–2.07 (m, 2H), 2.02–1.87 (m, 3H), 1.76–1.63 (m, 3H), 1.48–1.42 (m, 2H), 1.37–1.28 (m, 6H), 1.26 (s, 3H), 0.90 (t, J = 6.8 Hz, 3H).
^{13}**C NMR** (126 MHz, $CDCl_3$) δ 158.7, 104.9, 44.9, 33.1, 32.1, 30.7, 29.6, 28.4, 27.2, 22.8, 15.0, 14.3.
HRMS *m/z* (GC-CI): calculated for $C_{13}H_{25}$ $[M+H]^+$: 181.1948; found: 181.1950.

4-methyl-1-(1-methylcyclobutyl)pentan-1-one (S12m): Prepared according to GP1, 0.67 g, colorless liquid, 80%.

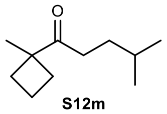

S12m

^{1}**H NMR** (501 MHz, $CDCl_3$) δ 2.41–2.35 (m, 4H), 1.99–1.91 (m, 1H), 1.78–1.68 (m, 3H), 1.57–1.50 (m, 1H), 1.49–1.43 (m, 2H), 1.37 (s, 1H), 0.89 (d, J = 6.5 Hz, 6H).
^{13}**C NMR** (126 MHz, $CDCl_3$) δ 215.1, 50.0, 34.1, 32.9, 30.5, 27.9, 23.9, 22.5, 14.6.
HRMS *m/z* (GC-CI): calculated for $C_{11}H_{21}O$ $[M+H]^+$: 169.1588; found: 169.1586.

1-methyl-1-(5-methylhex-1-en-2-yl)cyclobutane (1m): Prepared according to GP3, 0.41 g, colorless liquid, 83%.

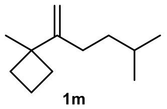

1m

^{1}**H NMR** (501 MHz, $CDCl_3$) δ 4.69–4.65 (m, 2H), 2.15–2.08 (m, 2H), 2.00–1.89 (m, 3H), 1.75–1.63 (m, 3H), 1.55–1.50 (m, 1H), 1.36–1.30 (m, 2H), 1.26 (s, 3H), 0.90 (d, J = 6.6 Hz, 6H).
^{13}**C NMR** (126 MHz, $CDCl_3$) δ 159.0, 104.9, 44.9, 37.8, 33.1, 28.4, 28.3, 27.2, 22.8, 15.0.
HRMS *m/z* (GC-CI): calculated for $C_{12}H_{21}$ $[M–H]^-$: 165.1635; found: 165.1637.

4,4-dimethyl-1-(1-methylcyclobutyl)pentan-1-one (S12n): Prepared according to GP1, 0.83 g, colorless liquid, 92%.

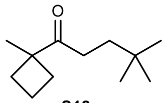

S12n

^{1}**H NMR** (501 MHz, $CDCl_3$) δ 2.43–2.33 (m, 4H), 2.02–1.91 (m, 1H), 1.80–1.68 (m, 3H), 1.49–1.45 (m, 2H), 1.38 (s, 3H), 0.89 (s, 9H).
^{13}**C NMR** (126 MHz, $CDCl_3$) δ 215.4, 50.2, 37.9, 31.7, 30.5, 30.1, 29.3, 24.0, 14.6.
HRMS *m/z* (GC-EI): calculated for $C_{12}H_{22}O$ $[M]^+$: 182.1664; found: 182.1665.

1-(5,5-dimethylhex-1-en-2-yl)-1-methylcyclobutane (1n): Prepared according to GP3, 0.47 g, colorless liquid, 87%.

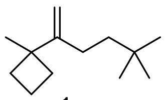

1n

^{1}**H NMR** (501 MHz, $CDCl_3$) δ 4.69–4.67 (m, 2H), 2.17–2.07 (m, 2H), 2.00–1.86 (m, 3H), 1.77–1.63 (m, 3H), 1.39–1.31 (m, 2H), 1.26 (s, 3H), 0.90 (s, 9H).

^{13}C NMR (126 MHz, $CDCl_3$) δ 159.4, 104.9, 45.1, 43.1, 33.1, 30.5, 29.5, 27.3, 25.4, 15.0.

HRMS *m/z* (GC-CI): calculated for $C_{13}H_{23}$ $[M–H]^-$: 179.1794; found: 179.1794.

(1-methylcyclopentyl)(phenyl)methanone (S12o): Prepared according to GP2, eluent: 1–3% EtOAc in *iso*-hexane, 0.54 g, colorless liquid, 55%.

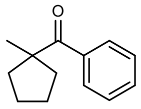

S12o

^{1}H NMR (501 MHz, $CDCl_3$) δ 7.88–7.83 (m, 2H), 7.50–7.46 (m, 1H), 7.44–7.38 (m, 2H), 2.37–2.28 (m, 2H), 1.77–1.62 (m, 6H), 1.42 (s, 3H).

^{13}C NMR (126 MHz, $CDCl_3$) δ 206.7, 136.9, 131.7, 129.1, 128.2, 54.9, 38.1, 27.3, 25.4.

HRMS *m/z* (GC-EI): calculated for $C_{13}H_{16}O$ $[M]^+$: 188.1198; found: 188.1195.

(1-(1-methylcyclopentyl)vinyl)benzene (1o): Prepared according to GP3, 0.46 g, colorless liquid, 83%.

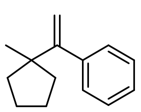

1o

^{1}H NMR (501 MHz, $CDCl_3$) δ 7.37–7.26 (m, 5H), 5.25 (d, *J* = 1.6 Hz, 1H), 4.94 (d, *J* = 1.7 Hz, 1H), 1.91–1.83 (m, 2H), 1.79–1.71 (m, 4H), 1.59–1.50 (m, 2H), 1.21 (s, 3H).

^{13}C NMR (126 MHz, $CDCl_3$) δ 159.0, 144.1, 128.7, 127.6, 126.5, 112.2, 48.4, 38.6, 26.8, 23.5.

HRMS *m/z* (GC-EI): calculated for $C_{14}H_{18}$ $[M]^+$: 186.1404; found: 186.1403.

(3-methoxyphenyl)(1-methylcyclopentyl)methanone (S12p): Prepared according to GP2, eluent: 1–3% Et_2O in *n*-pentane, 0.69 g, colorless liquid, 63%.

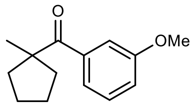

S12p

^{1}H NMR (501 MHz, $CDCl_3$) δ 7.44 (dt, *J* = 7.7, 1.2 Hz, 1H), 7.39 (dd, *J* = 2.7, 1.6 Hz, 1H), 7.32 (t, *J* = 7.9 Hz, 1H), 7.03 (ddd, *J* = 8.2, 2.7, 0.9 Hz, 1H), 3.84 (s, 3H), 2.42–2.24 (m, 2H), 1.82–1.61 (m, 6H), 1.42 (s, 3H).

^{13}C NMR (126 MHz, $CDCl_3$) δ 206.6, 159.6, 138.3, 129.11, 121.4, 117.8, 114.1, 55.5, 54.9, 38.2, 27.3, 25.4.

HRMS *m/z* (GC-EI): calculated for $C_{14}H_{18}O_2$ $[M]^+$: 218.1300; found: 218.1301.

1-methoxy-3-(1-(1-methylcyclopentyl)vinyl)benzene (1p): Prepared according to GP3, eluent: 1–2% Et_2O in *n*-pentane, 0.46 g, colorless liquid, 71%.

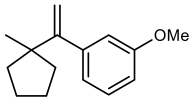

1p

^{1}H NMR (501 MHz, $CDCl_3$) δ 7.19 (t, *J* = 7.9 Hz, 1H), 6.83–6.78 (m, 2H), 6.75 (dd, *J* = 2.6, 1.6 Hz, 1H), 5.16 (d, *J* = 1.6 Hz, 1H), 4.88 (d, *J* = 1.7 Hz, 1H), 3.81 (s, 3H), 1.85–1.75 (m, 2H), 1.72–1.62 (m, 4H), 1.50–1.44 (m, 2H), 1.13 (s, 3H).

^{13}C NMR (126 MHz, $CDCl_3$) δ 158.9, 158.9, 145.5, 128.5, 121.3, 114.8, 112.2, 111.7, 55.3, 48.3, 38.7, 26.9, 23.5.

HRMS *m/z* (GC-ESI): calculated for $C_{15}H_{20}O_1$ $[M+H]^+$: 217.1585; found: 217.1586.

(3-fluorophenyl)(1-methylcyclopentyl)methanone (S12q): Prepared according to GP2, eluent: 1–2% Et_2O in *n*-pentane, 0.65 g, colorless liquid, 63%.

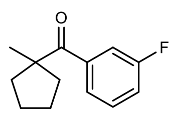

S12q

^{1}H NMR (501 MHz, $CDCl_3$) δ. 7.64 (dt, *J* = 7.8, 1.3 Hz, 1H), 7.54 (ddd, *J* = 9.8, 2.7, 1.6 Hz, 1H), 7.39 (td, *J* = 8.0, 5.7 Hz, 1H), 7.19 (tdd, *J* = 8.2, 2.6, 1.0 Hz, 1H), 2.43–2.24 (m, 2H), 1.77–1.63 (m, 6H), 1.41 (s, 3H).

^{19}F NMR (471 MHz, $CDCl_3$) δ –112.21.

1-fluoro-3-(1-(1-methylcyclopentyl)vinyl)benzene (1q): Prepared according to GP3, 0.53 g, colorless liquid, 87%.

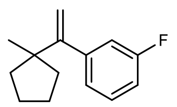

1q

^{1}H NMR (501 MHz, $CDCl_3$) δ 7.23 (td, *J* = 7.9, 6.1 Hz, 1H), 7.00–6.88 (m, 3H), 5.18 (d, *J* = 1.4 Hz, 1H), 4.89 (d, *J* = 1.4 Hz, 1H), 1.85–1.73 (m, 2H), 1.73–1.63 (m, 4H), 1.52–1.38 (m, 2H), 1.13 (s, 3H).

^{13}C NMR (126 MHz, $CDCl_3$) δ 162.2 (d, *J* = 244.9 Hz), 157.9 (d, *J* = 1.6 Hz), 146.3 (d, *J* = 7.4 Hz), 129.0 (d, *J* = 8.4 Hz), 124.5 (d, *J* = 2.9 Hz), 115.7 (d, *J* = 21.3 Hz), 113.3 (d, *J* = 20.9 Hz), 112.8, 48.3, 38.6, 26.8, 23.5, 23.5.

^{19}F NMR (471 MHz, $CDCl_3$) δ –114.37.

HRMS *m/z* (GC-EI): calculated for $C_{14}H_{17}F_1$ $[M]^+$: 204.1307; found: 204.1308.

(1-methylcyclopentyl)(4-(trifluoromethyl)phenyl)methanone (S12r): Prepared according to GP2, eluent: 1–3% Et_2O in *n*-pentane, 0.96 g, colorless liquid, 75%.

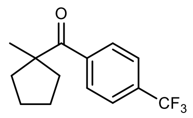

S12r

^{1}H NMR (501 MHz, $CDCl_3$) δ. 7.92 (d, *J* = 8.1 Hz, 2H), 7.68 (d, *J* = 8.3 Hz, 2H), 2.39–2.23 (m, 2H), 1.79–1.63 (m, 6H), 1.41 (s, 3H).

^{19}F NMR (471 MHz, $CDCl_3$) δ –63.06.

1-(1-(1-methylcyclopentyl)vinyl)-4-(trifluoromethyl)benzene (1r): Prepared according to GP3, 0.69 g, colorless liquid, 91%.

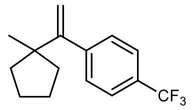

1r

^{1}H NMR (501 MHz, $CDCl_3$) δ 7.54 (d, *J* = 8.0 Hz, 2H), 7.31 (d, *J* = 7.7 Hz, 2H), 5.23 (d, *J* = 1.4 Hz, 1H), 4.89 (d, *J* = 1.3 Hz, 1H), 1.84–1.74 (m, 2H), 1.73–1.63 (m, 4H), 1.60–1.42 (m, 2H), 1.13 (s, 3H).

^{13}C NMR (126 MHz, $CDCl_3$) δ 157.9, 147.8, 129.0, 128.83 (q, *J* = 32.3 Hz), 124.51 (q, *J* = 271.8 Hz), 124.58 (q, *J* = 3.7 Hz), 113.2, 48.3, 38.6, 26.7, 23.5.

^{19}F NMR (471 MHz, $CDCl_3$) δ –62.39.

HRMS *m/z* (GC-EI): calculated for $C_{15}H_{17}F_3$ $[M]^+$: 254.1276; found: 245.1276.

(1-methylcyclobutyl)(phenyl)methanone (S12s): To a solution of bromobenzene (0.94 g, 6.0 mmol, 1.2 equiv.) in THF (20 mL) was slowly added *n*-butyl lithium solution (2.5M in hexane, 2.20 mL, 5.5 mmol, 1.1 equiv.) at –78 °C (dry ice/acetone bath) under Ar atmosphere. After stirred at the same temperature for 1 h, a solution of Weinreb amide **S11** (0.72 g, 5.0 mmol, 1.0 equiv.) in THF (5 mL) was added. After 1 h, the dry ice/acetone bath was removed, and the reaction was stirred at rt for 2 h. Then the reaction mixture was quenched with aq. HCl (1M, 25 mL) and extracted with Et_2O (3x25 mL). The organic layer was separated, dried over dried over Na_2SO_4, filtered and concentrated under reduced pressure. The crude product was purified by flash column chromatography (eluent: 1–3% Et_2O in *n*-pentane). Upon evaporation of the solvent under reduced pressure, ketone **S12s** was obtained as a colorless liquid (0.70 g, 80%).

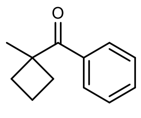
S12s

^{1}H NMR (501 MHz, $CDCl_3$) δ 7.87–7.83 (m, 2H), 7.55–7.48 (m, 1H), 7.45–7.39 (m, 2H), 2.76–2.63 (m, 2H), 2.20–1.98 (m, 3H), 1.90–1.75 (m, 1H), 1.61 (s, 3H).
^{13}C NMR (126 MHz, $CDCl_3$) δ 205.3, 134.4, 132.6, 129.3, 128.5, 49.2, 32.6, 25.9, 15.4.

(1-(1-methylcyclobutyl)vinyl)benzene (1s): Prepared according to GP3, 0.31 g, colorless liquid, 61%.

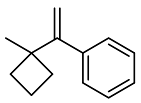
1s

^{1}H NMR (501 MHz, $CDCl_3$) δ 7.28–7.25 (m, 3H), 7.24–7.17 (m, 1H), 5.12 (d, *J* = 1.4 Hz, 1H), 4.99 (d, *J* = 1.4 Hz, 1H), 2.34 (qd, *J* = 9.2, 2.6 Hz, 2H), 2.09–1.95 (m, 1H), 1.92–1.83 (m, 2H), 1.78–1.67 (m, 1H), 1.37 (s, 3H).
^{13}C NMR (126 MHz, $CDCl_3$) δ 158.4, 141.5, 128.0, 127.4, 126.9, 110.7, 44.2, 34.4, 27.9, 15.3.
HRMS *m/z* (GC-EI): calculated for $C_{13}H_{16}$ $[M]^+$: 172.1246; found: 172.1246.

(1-methylcyclobutyl)(m-tolyl)methanone (S12t): The title compound was prepared according to the procedure of ketone **S12s,** using 3-bromotoluene (1.03 g, 6.0 mmol, 1.2 equiv.), *n*-butyl lithium (2.5M in hexane, 2.20 mL, 5.5 mmol, 1.1 equiv.) and Weinreb amide **S11** (0.72 g, 5.0 mmol, 1.0 equiv.). The crude product was purified by flash column chromatography (eluent: 1–3% Et_2O in *n*-pentane). Upon evaporation of the solvent under reduced pressure, ketone **S12t** was obtained as a colorless liquid (0.76 g, 81%).

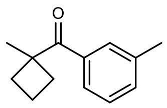
S12t

^{1}H NMR (501 MHz, $CDCl_3$) δ 7.70–7.67 (m, 1H), 7.61 (dt, *J* = 7.0, 1.9 Hz, 1H), 7.36–7.28 (m, 2H), 2.68 (tdd, *J* = 10.4, 8.2, 2.0 Hz, 2H), 2.39 (s, 3H), 2.15–1.99 (m, 3H), 1.85–1.74 (m, 1H), 1.61 (s, 3H).
^{13}C NMR (126 MHz, $CDCl_3$) δ 205.6, 138.3, 134.5, 133.3, 129.8, 128.2, 126.5, 49.3, 32.6, 25.9, 21.6, 15.3.
HRMS *m/z* (GC-EI): calculated for $C_{13}H_{16}O$ $[M]^+$: 188.1196; found: 188.1195.

1-methyl-3-(1-(1-methylcyclobutyl)vinyl)benzene (1t): Prepared according to GP3, 0.46 g, colorless liquid, 83%.

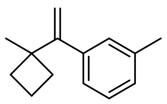

1t

^{1}H NMR (501 MHz, $CDCl_3$) δ 7.18 (t, *J* = 7.6 Hz, 1H), 7.11–7.02 (m, 3H), 5.11 (d, *J* = 1.4 Hz, 1H), 4.99 (d, *J* = 1.4 Hz, 1H), 2.39–2.28 (m, 2H), 2.35 (s, 3H), 2.10–1.97 (m, 1H), 1.93–1.84 (m, 2H), 1.79–1.68 (m, 1H), 1.39 (s, 3H).

^{13}C NMR (126 MHz, $CDCl_3$) δ 158.6, 141.6, 137.5, 128.3, 127.9, 127.7, 124.5, 110.5, 44.3, 34.4, 27.9, 21.7, 15.3.

HRMS *m/z* (GC-EI): calculated for $C_{14}H_{18}$ $[M]^+$: 186.1404; found: 186.1403.

1-(1-methylcyclopropyl)heptan-1-one (S12u): To a solution of 1-methylcyclopropane-1-carboxylic acid (0.50 g, 5.0 mmol, 1.0 equiv.) in THF (15 mL) was slowly added *n*-hexyl lithium (2.3 M in hexane, 3.26 mL, 7.50 mmol, 1.5 equiv.) at –78 °C under Ar atmosphere. The reaction was stirred further 15 h warming up slowly to rt. Then the reaction mixture was cooled in an ice/water bath, and sat. aq. NH_4Cl solution (50 mL) was slowly added. The layers were separated and the aqueous layer was extracted with diethyl ether (3x20 mL). The combined organic layers were collected and dried over Na_2SO_4, filtered and concentrated under reduced pressure. The crude product was purified by flash column chromatography (eluent: 1–3% Et_2O in *n*-pentane). Upon evaporation of the solvent under reduced pressure, ketone **S12u** was obtained as a colorless liquid (0.61 g, 72%).

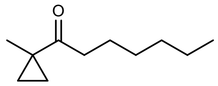

S12u

^{1}H NMR (501 MHz, $CDCl_3$) δ 2.36 (t, *J* = 7.4 Hz, 2H), 1.58–1.49 (m, 2H), 1.34 (s, 3H), 1.32–1.24 (m, 6H), 1.20 (q, *J* = 3.9 Hz, 2H), 0.97–0.83 (m, 3H), 0.73–0.63 (m, 2H).

^{13}C NMR (126 MHz, $CDCl_3$) δ 212.0, 37.9, 31.8, 29.1, 26.6, 24.2, 22.7, 20.0, 18.0, 14.2.

1-methyl-1-(oct-1-en-2-yl)cyclopropane (1u): Prepared according to GP3, 0.39 g, colorless liquid, 78%.

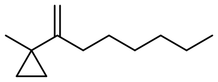

1u

^{1}H NMR (501 MHz, $CDCl_3$) δ 4.79 (d, *J* = 1.8 Hz, 1H), 4.70 (d, *J* = 1.6 Hz, 1H), 2.04–1.97 (m, 2H), 1.49–1.41 (m, 2H), 1.34–1.27 (m, 6H), 1.16 (s, 3H), 1.00–0.82 (m, 3H), 0.71–0.56 (m, 2H), 0.46–0.30 (m, 2H).

^{13}C NMR (126 MHz, $CDCl_3$) δ 153.9, 107.8, 33.7, 32.0, 29.5, 28.6, 24.2, 22.8, 21.2, 14.3, 13.4.

HRMS *m/z* (GC-CI Ammonia): calculated for $C_{12}H_{26}N$ $[M+NH_4]^+$: 184.2058; found: 184.2059.

1-(1-methylcyclohexyl)heptan-1-one (S12v): The title compound was prepared according to the procedure of ketone **S12u,** using 1-methylcyclohexane-1-carboxylic acid (0.71 g, 5.0 mmol, 1.0 equiv.) and *n*-hexyl lithium solution (2.3M in hexane, 3.26 mL, 7.50 mmol, 1.5 equiv.). The crude product was purified by flash column chromatography

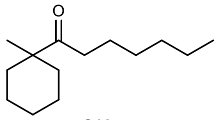

S12v

(eluent: 1–3% Et_2O in *n*-pentane). Upon evaporation of the solvent under reduced pressure, ketone **S12v** was obtained as a colorless liquid (0.75 g, 71%).

^{1}H NMR (501 MHz, $CDCl_3$) δ 2.44 (t, *J* = 7.3 Hz, 2H), 1.99–1.90 (m, 2H), 1.57–1.50 (m, 4H), 1.47–1.39 (m, 1H), 1.38–1.21 (m, 11H), 1.06 (s, 3H), 0.88 (t, *J* = 6.9 Hz, 3H).

^{13}C NMR (126 MHz, $CDCl_3$) δ 216.0, 48.2, 36.5, 34.8, 31.9, 29.2, 26.0, 24.9, 24.0, 23.1, 22.7, 14.2.

HRMS *m/z* (GC-EI): calculated for $C_{14}H_{26}O$ $[M]^+$: 210.1978; found: 210.1978.

1-methyl-1-(oct-1-en-2-yl)cyclohexane (1v): Prepared according to GP3, 0.99 g, colorless liquid, 83%.

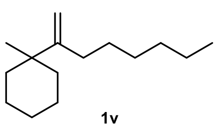

1v

^{1}H NMR (501 MHz, $CDCl_3$) δ 4.86 (s, 1H), 4.80 (s, 1H), 2.02–1.91 (m, 2H), 1.71–1.62 (m, 2H), 1.49–1.26 (m, 16H), 0.97 (s, 3H), 0.94–0.82 (m, 3H).

^{13}C NMR (126 MHz, $CDCl_3$) δ 157.1, 107.1, 39.3, 36.6, 32.1, 31.2, 29.7, 29.3, 27.1, 26.6, 22.9, 22.8, 14.3.

HRMS *m/z* (GC-EI): calculated for $C_{15}H_{28}$ $[M]^+$: 208.2181; found: 208.2185.

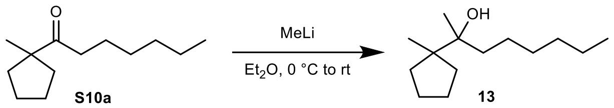

2-(1-methylcyclopentyl)octan-2-ol (13): To a solution of 1-(1-methylcyclopentyl)heptan-1-one (**S10a,** 0.39 g, 2.0 mmol, 1.0 equiv.) in Et_2O (10 mL) was slowly added MeLi (1.6 M in Et_2O, 2.5 mL, 4.0 mmol, 2.0 equiv.) at 0 °C under Ar atmosphere. The reaction was stirred further 12 h warming up slowly in the cold bath to rt. The reaction mixture was cooled in an ice/water bath, and sat. aq. NH_4Cl solution (15 mL) was slowly added. The layers were separated and the aqueous layer was extracted with diethyl ether (3x15 mL). The combined organic layers were collected and dried over Na_2SO_4, filtered and concentrated under reduced pressure. The crude product was purified by flash column chromatography (eluent: 2–6% Et_2O in *n*-pentane). Upon evaporation of the solvent under reduced pressure, the corresponding alcohol **13** was obtained as a colorless liquid (0.30 g, 70%).

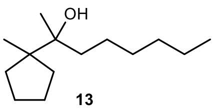

13

^{1}H NMR (501 MHz, $CDCl_3$) δ 1.78–1.70 (m, 2H), 1.66–1.51 (m, 4H), 1.50–1.39 (m, 3H), 1.37–1.24 (m, 7H), 1.24–1.14 (m, 3H), 1.11 (s, 3H), 0.94 (s, 3H), 0.90–0.84 (m, 3H).

^{13}C NMR (126 MHz, $CDCl_3$) δ 76.5, 50.9, 37.6, 34.9, 34.5, 32.1, 30.3, 25.8, 25.6, 24.4, 23.9, 22.8, 22.3, 14.2.

HRMS *m/z* (GC-CI): calculated for $C_{14}H_{27}O$ $[M–H]^-$: 211.2054; found: 211.2056.

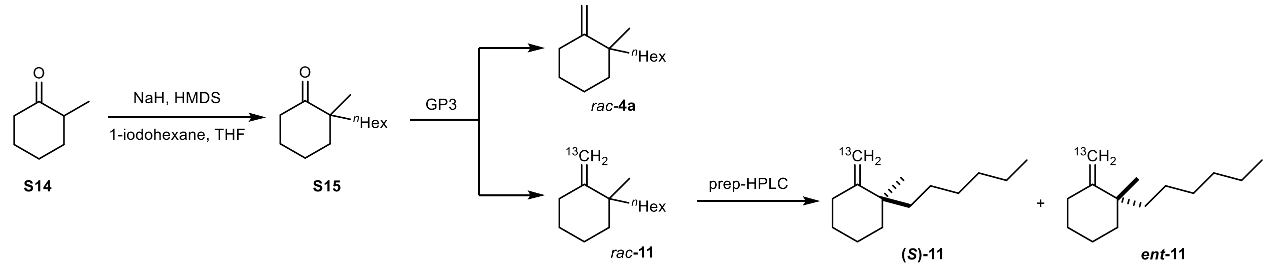

2-hexyl-2-methylcyclohexan-1-one (S15): The synthesis is a modified literature procedure for a related compound[14]. A 100 mL flame dried flask equipped with a stir bar and reflux condenser was charged with sodium hydride (60% dispersion, 0.88 g, 24.0 mmol, 1.1 equiv.) and dry THF (45mL) and cooled to 0 °C under argon. 2-methylcyclohexanone (**S14,** 20.0 mmol, 1.0 equiv.) was slowly added to the solution. The reaction mixture was then refluxed for 1.5 h. Hexamethyldisilazane (0.62 mL, 3.0 mmol, 0.15 equiv.) was added and the reaction mixture further refluxed for 15 min. The reaction mixture was cooled to 0 °C and 1-hexyl iodide (5.90 mL, 40.0 mmol, 2.0 equiv.) added dropwise via syringe. The reaction was stirred further 12 h warming up slowly in the cold bath to rt. The reaction mixture quenched with sat. aq. NH_4Cl solution (50 mL).The layers were separated and the aqueous layer was extracted with diethyl ether (3x25 mL). The combined organic layers were collected and dried over Na_2SO_4, filtered and concentrated under reduced pressure. The crude product was purified by flash column chromatography (eluent: 1–3% Et_2O in *n*-pentane). Upon evaporation of the solvent under reduced pressure, the corresponding ketone **S15** was obtained as a colorless liquid (1.13 g, 29%).

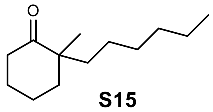
S15

^{1}H NMR (501 MHz, $CDCl_3$) δ 2.44–2.36 (m, 1H), 2.35–2.27 (m, 1H), 1.97–1.87 (m, 1H), 1.85–1.62 (m, 5H), 1.59–1.48 (m, 1H), 1.38 (td, *J* = 13.6, 3.7 Hz, 1H), 1.30–1.23 (m, 7H), 1.05–0.98 (s, 4H), 0.87 (t, *J* = 6.8 Hz, 3H).

^{13}C NMR (126 MHz, $CDCl_3$) δ 216.4, 48.8, 39.6, 39.0, 37.7, 31.8, 30.1, 27.7, 23.8, 22.8, 22.7, 21.2, 14.2.

HRMS *m/z* (GC-EI): calculated for $C_{14}H_{24}O$ $[M]^+$: 196.1820; found: 196.1821.

1-hexyl-1-methyl-2-methylenecyclohexane (*rac*-4a): Prepared according to GP3, 0.58 g, colorless liquid, 83%.

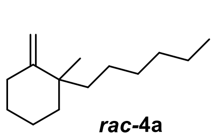
rac-4a

^{1}H NMR (600 MHz, $CDCl_3$) δ 4.69 (dt, *J* = 1.4 Hz, *J* = 1.1 Hz, 1H), 4.57 (d, *J* = 2.1 Hz, 1H), 2.18–2.06 (m, 2H), 1.73–1.68 (m, 1H), 1.67–1.58 (m, 2H), 1.55–1.46 (m, 2H), 1.36–1.22 (m, 8H), 1.21–1.13 (m, 2H), 1.07–1.02 (m, 1H), 1.00 (s, 3H), 0.88 (t, *J* = 7.1 Hz, 3H).

^{13}C NMR (151 MHz, $CDCl_3$) δ 155.8, 106.5, 40.7, 39.5, 37.6, 33.4, 32.1, 30.4, 28.8, 25.8, 24.0, 22.9, 22.2, 14.3.

HRMS *m/z* (GC-CI): calculated for $C_{14}H_{27}$ $[M+H]^+$: 195.2107; found: 195.2107.

(*S*)-1-hexyl-1-methyl-2-(methylene-^{13}C)cyclohexane (11): The title compound was prepared according to the GP3 from compound **S15** (0.59 g, 3.0 mmol, 1.0 equiv.) and (methyl-^{13}*C*)triphenylphosphonium iodide[13] (1.58 g, 3.9 mmol, 1.3 equiv.). Purification by flash column chromatography on silica (eluent: 100% *n*-pentane). Upon evaporation of the solvent under reduced pressure, the corresponding compound **11** was obtained as a colorless liquid (0.52 g, 89% yield). Both enantiomers (*S*)-**11** and ***ent*-11** was separated by using preparative chiral HPLC.

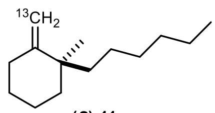

(*S*)-11

^{1}H NMR (600 MHz, $CDCl_3$) δ 4.68 (ddt, $^1J_{CH}$ = 154.0, *J* = 2.0, 1.2 Hz, 1H), 4.56 (dd, $^1J_{CH}$ = 154.7, *J* = 2.0 Hz, 1H), 2.18–2.07 (m, 2H), 1.73–1.67 (m, 1H), 1.67–1.56 (m, 2H), 1.55–1.45 (m, 2H), 1.37–1.12 (m, 10H), 1.08–0.99 (m, 1H), 1.00 (s, 3H), 0.88 (t, *J* = 7.1 Hz, 3H).

^{13}C NMR (151 MHz, $CDCl_3$) δ 155.7 (d, *J* = 72.1 Hz), 106.5, 40.7 (d, *J* = 1.7 Hz), 39.5 (d, *J* = 1.0 Hz), 37.6 (d, *J* = 2.0 Hz), 33.4 (d, *J* = 2.7 Hz), 32.1, 30.4, 28.8 (d, *J* = 2.4 Hz), 25.8 (d, *J* = 4.0 Hz), 24.0, 22.9, 22.2, 14.3.

HRMS *m/z* (GC-EI): calculated for $C_{13}H_{26}{}^{13}C$ $[M]^+$: 195.2061; found: 195.2062.

Preparative HPLC condition: enantiomers were separated by preparative HPLC on a chiral stationary phase; HPLC column: 250 mm Chiralpak IG, solvent system: CH_3CN/H_2O = 75/25, flow rate: 42.5 mL/min., temp.: 25 °C, 205 nm, t_R = 11.05 min (*S*-**11**), t_R = 12.05 min (*ent*-**11**)

Collected solvent (water and acetonitrile mixture) was diluted with sat. aq. NaCl solution (200 mL) and diethyl ether (50 mL). The layers were separated and the aqueous layer was extracted with diethyl ether (3x50 mL). The combined organic layers were collected and dried over Na_2SO_4, filtered and concentrated under reduced pressure to afford (*S*)-**11** with 99.9:0.1 er and ***ent*-11** with 98.3:1.7 er.

GC condition: (30.0 m BGB-176, injection temperature: 220 °C, 100 °C iso 60 min, 20 °C/min, 220 °C iso 10 min, 0.5 bar He): t_R = 47.5 min [(*S*)-**11**], t_R = 48.3 min [***ent*-11**].

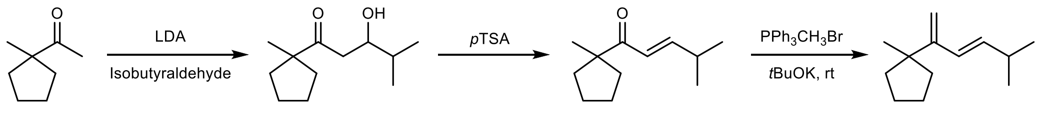

S16 S17 S12w 1w

3-hydroxy-4-methyl-1-(1-methylcyclopentyl)pentan-1-one (S17): To a solution of diisopropylamine (1.46 mL, 10.5 mmol, 1.20 equiv.) in 20 mL of anhydrous THF at –78 °C was added *n*-butyllithium (2.5 M in hexanes, 3.83 mL, 9.6 mmol, 1.10 equiv) under Ar atmosphere. The mixture was stirred for 30 min at 0 °C and recooled to –78 °C. A solution of ketone (**S16,** 1.1 g, 8.72 mmol, 1.0 equiv.) in 5 mL of anhydrous THF was added dropwise over 5 min and the mixture was stirred for 1 h at –78 °C. Isobutyraldehyde (0.95 mL, 10.46 mmol, 1.2 equiv) was added and the reaction mixture was stirred for 10 min and poured into saturated aqueous NH_4Cl. The mixture was extracted with Et_2O (3x25 mL) and the combined organic layers were washed with brine (30 mL),

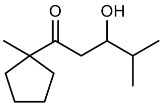

S17

dried over Na_2SO_4, filtered, and concentrated. The residue was purified by flash column chromatography on silica gel (eluent, 5–15% Et_2O in *n*-pentane) to afford **S17** (1.1 g, 64%) as a colorless oil.

^{1}H NMR (501 MHz, CD_2Cl_2) δ 3.78–3.67 (m, 1H), 3.11 (d, *J* = 3.0 Hz, 1H), 2.68 (dd, *J* = 17.3, 2.1 Hz, 1H), 2.48 (dd, *J* = 17.3, 9.8 Hz, 1H), 2.13–1.92 (m, 2H), 1.78–1.59 (m, 5H), 1.45–1.35 (m, 2H), 1.19 (s, 3H), 0.91 (t, *J* = 7.1 Hz, 6H).

^{13}C NMR (126 MHz, $CDCl_3$) δ 217.4, 73.0, 56.3, 41.3, 36.6, 33.6, 25.5, 25.5, 24.6, 18.7, 17.9.

HRMS *m/z* (GC-EI): calculated for $C_{12}H_{22}O_2$ $[M+Na]^+$: 221.1513; found: 221.1511.

(E)-4-methyl-1-(1-methylcyclopentyl)pent-2-en-1-one (S12w): Alcohol (**S17,** 1.15 g, 5.79 mmol, 1.0 equiv.) was dissolved in benzene (10 mL), then p-toluene sulfonic acid (1.27 g, 6.67 mmol, 1.15 equiv.) was added under Ar atmosphere at rt. The reaction mixture was the heated at 50 °C for 5 h. The reaction was cooled to rt and quenched with sat. $NaHCO_3$ (30 mL) and extracted with ether (2x30 mL). The organic layer sequentially washed with brine (30 mL), dried over Na_2SO_4 and concentrated. The residue was purified by flash column chromatography on silica gel (eluent, 1–3% Et_2O in *n*-pentane) to afford ketone **S12w** as a colorless oil (1.0 g, 96%).

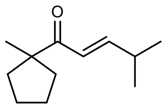

S12w

^{1}H NMR (501 MHz, $CDCl_3$) δ 6.90 (dd, *J* = 15.4, 6.8 Hz, 1H), 6.34 (dd, *J* = 15.4, 1.4 Hz, 1H), 2.45 (dqd, *J* = 13.6, 6.8, 1.4 Hz, 1H), 2.20–1.99 (m, 2H), 1.75–1.56 (m, 4H), 1.46–1.35 (m, 2H), 1.19 (s, 3H), 1.06 (d, *J* = 6.8 Hz, 6H).

HRMS *m/z* (GC-EI): calculated for $C_{12}H_{20}O$ $[M]^+$: 180.1508; found: 180.1508.

(E)-1-methyl-1-(5-methylhexa-1,3-dien-2-yl)cyclopentane (1w): Prepared according to GP3, 0.27 g, colorless liquid, 51%.

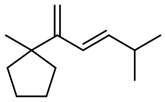

1w

^{1}H NMR (501 MHz, $CDCl_3$) δ 5.98–5.91 (m, 1H), 5.83 (dd, *J* = 15.5, 6.7 Hz, 1H), 4.99 (s, 1H), 4.71 (d, *J* = 1.7 Hz, 1H), 2.33 (dqd, *J* = 13.4, 6.7, 1.2 Hz, 1H), 1.79–1.58 (m, 6H), 1.49–1.37 (m, 2H), 1.08 (s, 3H), 1.02 (d, *J* = 6.8 Hz, 6H).

HRMS *m/z* (GC-EI): calculated for $C_{13}H_{22}$ $[M]^+$: 178.1716; found: 178.1716.

3. Reaction development

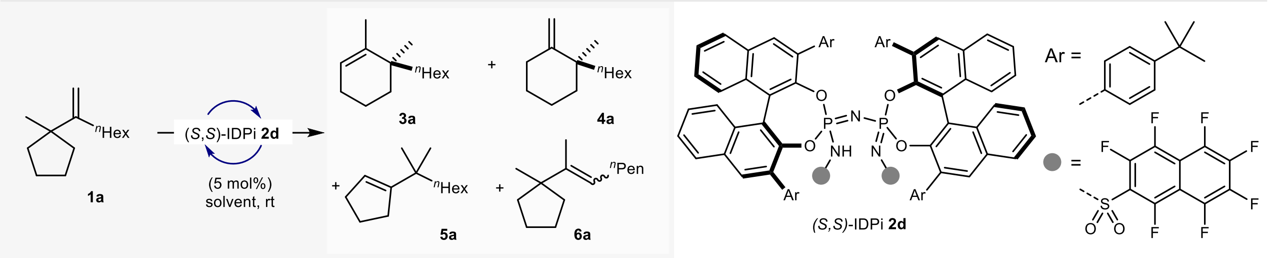

entry	solvent	time (h)	conv. (%)	yield (3a%)	3a:4a:5a:6a	e.r. (3a)	e.r. (4a)
1	Et_2O (0.5 M)	72	52	37	74:21:1:4	95.5:4.5	94.5:5.5
2	Toluene (0.5 M)	72	59	45	77:17:0.5:5.5	96:4	94.5:5.5
3	*n*-Hexane (0.5 M)	72	51	36	71:13:1:15	95.5:4.5	93.5:6.5
4	MeCy (0.5 M)	72	83	69	82:4:1:13	96:4	93:7
5	$CHCl_3$ (0.2 M)	72	71	45	65:34:0.5:1	96.5:3.5	97:3
6	$CHCl_3$ (0.5 M)	72	99	90	91:6.5:0.5:2	97:3	97:3
7[a]	$CHCl_3$ (0.5 M)	72	31	15	47:52:0.5:0.5	96:4	97:3
8[b]	$CHCl_3$ (1 M)	72	99	90	92:5:0.5:2.5	97:3	96:4
9[b]	$CHCl_3$ (4 M)	24	99	93	95:1:0.5:3.5	97:3	N/D

Table S1: Reactions conducted on 0.03 mmol scale. Conv. and yields were determined by ^{1}H NMR spectroscopy using 1,3,5-trimethoxybenzene as internal standard. Side products distribution ratio were determined by crude ^{1}H NMR spectroscopy. The e.r. was determined by GC. N/D = not determined. [a]Used (*S*,*S*)-IDPi **2d** (1 mol%). [b]Reaction conducted on 0.2 mmol scale. ^{n}Pen, *n*-pentyl; ^{n}Hex, *n*-hexyl; MeCy, methyl cyclohexane; conv., conversion.

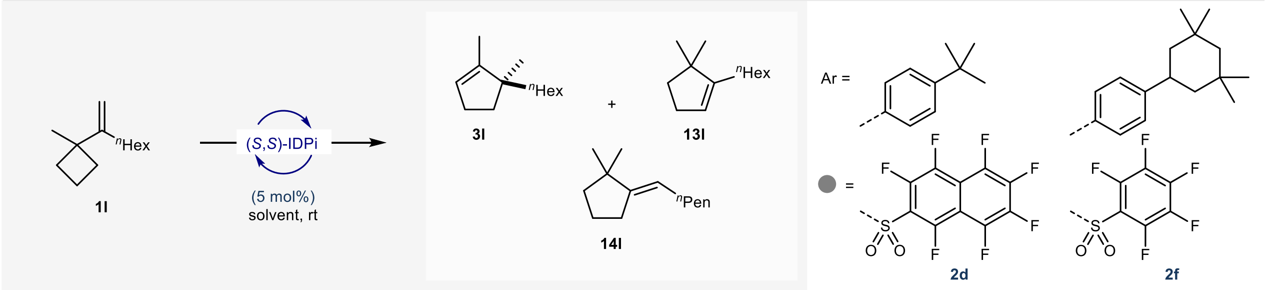

entry	solvent	IDPi	time (h)	conv. (%)	yield (3l%)	3l:13l:14l	e.r. (3l)
1	$CHCl_3$ (0.2 M)	**2d**	48	98	85	86:10:2	81:19
2	$CHCl_3$ (0.5 M)	**2d**	24	99	87	89:9:2	81.5:18.5
3	*n*-Hexane (0.5 M)	**2d**	24	97	92	93:6.5:0.5	82:18
4	$CHCl_3$ (0.5 M)	**2f**	24	94	90	91:7:2	86.5:13.5
5	*n*-Hexane (0.5 M)	**2f**	36	99	94	96:3.5:0.5	90:10
6[a]	*n*-Hexane (4 M)	**2f**	24	99	93	95:4:1	88:12

Table S2: Reactions conducted on 0.03 mmol scale. Conv. and yields were determined by ^{1}H NMR spectroscopy using 1,3,5-trimethoxybenzene as internal standard. Side products distribution ratio were determined by crude ^{1}H NMR spectroscopy. The e.r. was determined by GC analysis. [a]Reaction conducted on 0.20 mmol scale.

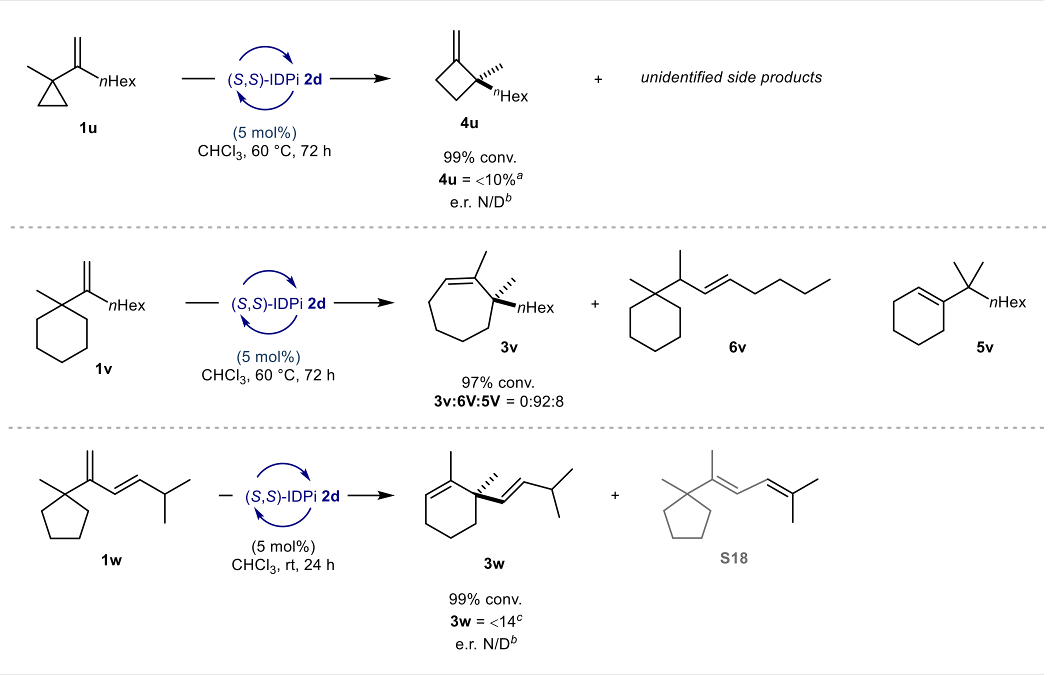

Figure S1: Reactions conducted on 0.03 mmol scale. Conv. and yields were determined by ^{1}H NMR spectroscopy using 1,3,5-trimethoxybenzene as internal standard. [a]Remaining material was unidentified side products. [b]Because of the difficulties to isolate the desired product in pure form, the e.r. was not determined. [c]Major amount of side product **S18** (>80%) was formed along with traces of other unidentified side products.

While the three to four membered ring expansion could occur by releasing ~1.2 kcal/mole strain energy, the six to seven membered ring expansion is energetically uphill by ~6.3 kcal/mol. As expected, we found that under the optimal condition both the three and six membered ring substrates are unreactive. However, at higher temperature, the three membered ring substrate **1u** gave complex reaction mixture with <10% of desired product. The six membered ring substrate **1v** at higher temperature, gave full conversion to the olefin isomerization of staring substrate **6v** and small amounts of the methyl migrated side product **5v** without ring expansion. With optimal condition diene substrate **1w,** gave full conversion to the olefin isomerization of starting substrate **S18** and <14% desired product.

3a. General procedure for the Wagner-Meerwein shift (GP4):

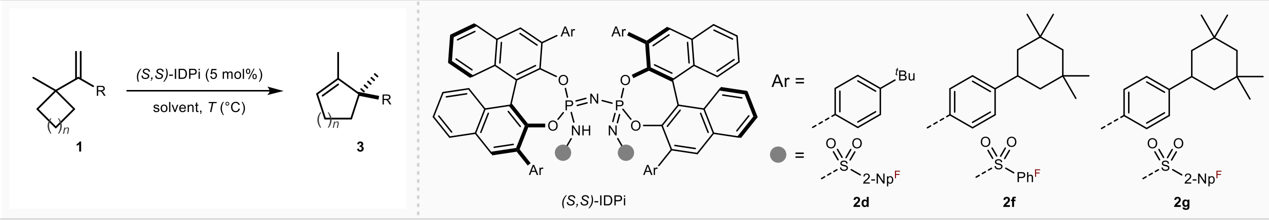

In a 2 mL glass vial, the respective IDPi catalyst (22.96–27.06 mg, 0.0125 mmol, 5 mol%) was measured and it was dissolved in $CHCl_3$ (62.5 µL, 4 M with respect to the olefin substrate). At rt, olefin substrate **1** (0.25 mmol, 1.0 equiv.) was added. The reaction mixture was stirred for indicated time and temperature. The crude reaction mixtures were directly purified *via* flash column chromatography on silver nitrate impregnated silica gel (eluent: 2–16% CH_2Cl_2 in *n*-pentane or 0.5–2% Et_2O in *n*-pentane) to give the ring expansion products **3**.

Note: Thin layer chromatography (TLC) was performed using silver nitrate impregnated silica gel pre-coated glass plate (250µm, 10% $AgNO_3$, Analtech Brand) which were visualized by staining with PMA. Silver nitrate impregnated silica gel was prepared by adding a solution of $AgNO_3$ (10 g, in 10 mL of Millipore water) to a 100 g silica gel (Merck, 60 Å, 230−400 mesh, particle size 0.040−0.063 mm) suspended in 200 mL of Millipore water to make a 10% $AgNO_3$ on silica gel. It was then shaken and water was evaporated under reduced pressure with a rotary evaporator in dark environment at 55 °C. Then further dried in an oven for 48 h at 100 °C.

$CDCl_3$ was allowed to stand over anhydrous K_2CO_3 and 4Å MS to neutralize and dried prior to NMR sample preparation.

(*S*)-6-hexyl-1,6-dimethylcyclohex-1-ene (3a): Prepared according to GP4 using (*S*,*S*)-IDPi **2d** in $CHCl_3$ (4 M) at rt for 24 h, eluent: 2–6% CH_2Cl_2 in *n*-pentane, 44.0 mg, colorless liquid, 90.5%.

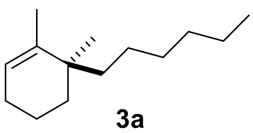

3a

^{1}H NMR (501 MHz, $CDCl_3$) δ 5.37–5.34f (m, 1H), 1.93–1.89 (m, 2fH), 1.64–1.52 (m, 6H), 1.41–1.35 (m, 1H), 1.30–1.21 (m, 9H), 1.11–1.06 (m, 1H), 0.97 (s, 3H), 0.88 (t, *J* = 7.0 Hz, 3H).

^{13}C NMR (126 MHz, $CDCl_3$) δ 140.7, 122.9, 40.2, 37.0, 35.0, 32.1, 30.5, 26.6, 26.1, 24.2, 22.9, 19.6, 19.0, 14.3.

HRMS *m/z* (GC-EI): calculated for $C_{14}H_{26}$ $[M]^+$: 194.2028; found: 194.2029.

The enantiomeric ratio was measured by GC (30.0 m BGB-176, injection temperature: 220 °C, 100 °C iso 60 min, 20 °C/min, 220 °C iso 10 min, 0.5 bar He): t_R = 50.1 min (major), t_R = 51.6 min (minor), e.r. = 97:3. $[\alpha]_D^{25}$ = +6.48 (*c* 0.52, CH_2Cl_2).

(*S*)-6-decyl-1,6-dimethylcyclohex-1-ene (3b): Prepared according to GP4 using (*S*,*S*)-IDPi **2d** in $CHCl_3$ (4 M) at rt for 24 h, eluent: 2–6% CH_2Cl_2 in *n*-pentane, 58.0 mg, colorless liquid, 92.6%.

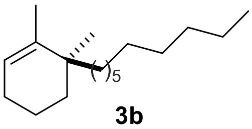

3b

^{1}H NMR (501 MHz, $CDCl_3$) δ 5.38–5.33 (m, 1H), 1.95–1.86 (m, 2H), 1.65–1.51 (m, 6H), 1.43–1.34 (m, 1H), 1.31–1.20 (m, 17H), 1.12–1.04 (m, 1H), 0.97 (s, 3H), 0.88 (t, J = 7.0 Hz, 3H).

^{13}C NMR (126 MHz, $CDCl_3$) δ 140.7, 122.9, 40.2, 37.0, 35.0, 32.1, 30.8, 29.89, 29.9, 29.8, 29.5, 26.6, 26.1, 24.2, 22.9, 19.6, 19.1, 14.3.

HRMS *m/z* (GC-EI): calculated for $C_{18}H_{34}$ $[M]^+$: 250.2653; found: 250.2655.

The enantiomeric ratio was measured by GC (30.0 m BGB-176, injection temperature: 220 °C, 100 °C iso 600 min, 8 °C/min, 240 °C iso 3 min, 0.6 bar H_2): t_R = 501.6 min (major), t_R = 515.2 min (minor), e.r. = 97.5:2.5. $[\alpha]_D^{25}$ = +2.62 (*c* 1.22, CH_2Cl_2).

(*S*)-6-butyl-1,6-dimethylcyclohex-1-ene (3c): Prepared according to GP4 using (*S*,*S*)-IDPi **2d** in $CHCl_3$ (4 M) at rt for 24 h, eluent: 2–6% CH_2Cl_2 in *n*-pentane, 37.5 mg, colorless liquid, 90.1%.

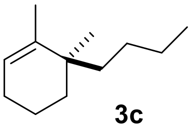

3c

^{1}H NMR (501 MHz, $CDCl_3$) δ 5.38–5.33 (m, 1H), 1.95–1.85 (m, 2H), 1.65–1.53 (m, 6H), 1.44–1.35 (m, 1H), 1.32–1.18 (m, 6H), 0.97 (s, 3H), 0.90 (t, J = 7.1 Hz, 3H).

^{13}C NMR (126 MHz, $CDCl_3$) δ 140.7, 122.9, 39.9, 37.0, 35.0, 26.6, 26.5, 26.1, 23.8, 19.6, 19.0, 14.3.

HRMS *m/z* (GC-EI): calculated for $C_{12}H_{22}$ $[M]^+$: 166.1717; found: 166.1716.

The enantiomeric ratio was measured by GC (30.0 m CycloSil-B, injection temperature: 220 °C, 85 °C iso 43 min, 20 °C/min, 220 °C iso 5 min, 0.5 bar He): t_R = 39.0 min (major), t_R = 40.6 min (minor), e.r. = 96:4. $[\alpha]_D^{25}$ = +14.69 (*c* 0.88, CH_2Cl_2).

(*S*)-1,6-dimethyl-6-propylcyclohex-1-ene (3d): Prepared according to GP4 using (*S*,*S*)-IDPi **2d** in $CHCl_3$ (4 M) at rt for 24 h, eluent: 2–6% CH_2Cl_2 in *n*-pentane, 31.0 mg, colorless liquid, 81.4%.

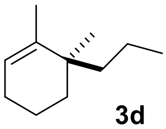

3d

^{1}H NMR (501 MHz, $CDCl_3$) δ 5.39–5.33 (m, 1H), 1.94–1.88 (m, 2H), 1.65–1.52 (m, 6H), 1.42–1.34 (m, 1H), 1.32–1.21 (m, 3H), 1.17–1.05 (m, 1H), 0.97 (s, 3H), 0.88 (t, J = 7.1 Hz, 3H).

^{13}C NMR (126 MHz, $CDCl_3$) δ 140.7, 122.9, 42.7, 37.1, 35.1, 26.5, 26.1, 19.6, 19.0, 17.5, 15.2.

HRMS *m/z* (GC-EI): calculated for $C_{11}H_{20}$ $[M]^+$: 152.1560; found: 152.1559.

The enantiomeric ratio was measured by GC (30.0 m CycloSil-B, injection temperature: 220 °C, 75 °C iso 44 min, 20 °C/min, 220 °C iso 5 min, 0.5 bar He): t_R = 33.6 min (major), t_R = 35.0 min (minor), e.r. = 94:6. $[\alpha]_D^{25}$ = +22.17 (*c* 0.41, CH_2Cl_2).

(*S*)-6-ethyl-1,6-dimethylcyclohex-1-ene (3e): Prepared according to GP4 using (*S*,*S*)-IDPi **2d** in $CHCl_3$

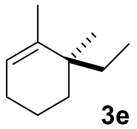

3e

(4 M) at rt for 24 h, eluent: 2–6% CH_2Cl_2 in *n*-pentane, 21.5 mg, colorless liquid, 62.2%. **^{1}H NMR** (501 MHz, $CDCl_3$) δ 5.40–5.35 (m, 1H), 1.98–1.88 (m, 2H), 1.64–1.53 (m, 6H), 1.50–1.41 (m, 1H), 1.33–1.21 (m, 2H), 0.97 (s, 3H), 0.77 (t, *J* = 7.5 Hz, 3H).

^{13}C NMR (126 MHz, $CDCl_3$) δ 140.5, 123.2, 37.1, 34.2, 32.2, 26.1, 19.5, 19.0, 8.6.

HRMS *m/z* (GC-EI): calculated for $C_{10}H_{18}$ $[M]^+$: 138.1404; found: 138.1403.

The enantiomeric ratio was measured by GC (30.0 m Ivadex-1, injection temperature: 220 °C, 30–50 °C (0.5 °C/min) iso 10 min, 20 °C/min, 220 °C iso 5 min, 0.5 bar He): t_R = 40.3 min (minor), t_R = 42.0 min (major), e.r. = 84:16. $[\alpha]_D^{25}$ = +31.74 (*c* 0.46, CH_2Cl_2).

(*R*)-6-isopentyl-1,6-dimethylcyclohex-1-ene (3f): Prepared according to GP4 using (*S*,*S*)-IDPi **2d** in

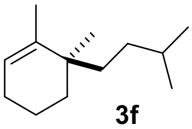

3f

$CHCl_3$ (4 M) at rt for 24 h, eluent: 2–6% CH_2Cl_2 in *n*-pentane, 41.5 mg, colorless liquid, 92.1%.

^{1}H NMR (501 MHz, $CDCl_3$) δ 5.38–5.35 (m, 1H), 1.96–1.87 (m, 2H), 1.66–1.54 (m, 6H), 1.51–1.36 (m, 2H), 1.30–1.19 (m, 2H), 1.18–1.08 (m, 1H), 1.00–0.93 (s, 4H), 0.88 (d, *J* = 6.6 Hz, 6zH).

^{13}C NMR (126 MHz, $CDCl_3$) δ 140.6, 123.0, 37.8, 36.9, 35.0, 33.3, 29.0, 26.7, 26.1, 22.93, 22.90, 19.6, 19.0.

HRMS *m/z* (GC-EI): calculated for $C_{13}H_{24}$ $[M]^+$: 180.1872; found: 180.1872.

The enantiomeric ratio was measured by GC (30.0 m CycloSil-B, injection temperature: 220 °C, 90 °C iso 50 min, 20 °C/min, 220 °C iso 5 min, 0.5 bar He): t_R = 44.2 min (major), t_R = 45.9 min (minor), e.r. = 98:2. $[\alpha]_D^{25}$ = +17.17 (*c* 0.88, CH_2Cl_2).

(*R*)-6-(3,3-dimethylbutyl)-1,6-dimethylcyclohex-1-ene (3g): Prepared according to GP4 using (*S*,*S*)-

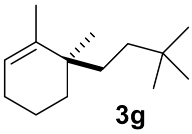

3g

IDPi **2d** in $CHCl_3$ (4 M) at rt for 24 h, eluent: 2–6% CH_2Cl_2 in *n*-pentane, 45.0 mg, colorless liquid, 92.6%.

^{1}H NMR (501 MHz, $CDCl_3$) δ 5.39–5.35 (m, 1H), 1.94–1.88 (m, 2H), 1.66–1.50 (m, 6H), 1.43–1.35 (m, 1H), 1.29–1.10 (m, 3H), 1.01–0.96 (m, 4H), 0.87 (s, 9H).

^{13}C NMR (126 MHz, $CDCl_3$) δ 140.6, 123.1, 37.9, 36.8, 34.8, 34.4, 30.3, 29.6, 26.8, 26.1, 19.6, 19.0.

HRMS *m/z* (GC-EI): calculated for $C_{14}H_{26}$ $[M]^+$: 194.2031; found: 194.2029.

The enantiomeric ratio was measured by GC (30.0 m BGB-176, injection temperature: 220 °C, 100 °C iso 30 min, 20 °C/min, 220 °C iso 5 min, 0.5 bar He): t_R = 25.8 min (major), t_R = 26.4 min (minor), e.r. = 99:1. $[\alpha]_D^{25}$ = +18.3 (*c* 1.33, CH_2Cl_2).

(*R*)-6-(2-cyclohexylethyl)-1,6-dimethylcyclohex-1-ene (3h): Prepared according to GP4 using (*S*,*S*)-IDPi **2d** in $CHCl_3$ (4 M) at rt for 24 h, eluent: 2–6% CH_2Cl_2 in *n*-pentane, 52.0 mg, colorless liquid, 92.9%.

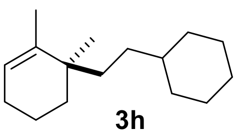

3h

^{1}H NMR (501 MHz, $CDCl_3$) δ 5.38–5.33 (m, 1H), 1.94–1.88 (m, 2H), 1.74–1.52 (m, 11H), 1.47–1.37 (m, 1H), 1.29–1.05 (m, 7H), 1.00–0.93 (m, 4H), 0.92–0.82 (m, 2H).

^{13}C NMR (126 MHz, $CDCl_3$) δ 140.6, 123.0, 38.8, 37.3, 37.0, 34.9, 33.8, 33.7, 31.8, 27.0, 26.7, 26.1, 19.6, 19.0.

HRMS *m/z* (GC-EI): calculated for $C_{14}H_{28}$ $[M]^+$: 220.2186; found: 220.2185.

The enantiomeric ratio was measured by GC (25.0 m Ivadex-1, injection temperature: 220 °C, 80 °C iso 325 min, 8 °C/min, 220 °C iso 3 min, 0.5 bar H_2): t_R = 275.2 min (major), t_R = 282.4 min (minor), e.r. = 98.5:1.5. $[\alpha]_D^{25}$ = +1.42 (*c* 0.56, CH_2Cl_2).

(*R*)-1,6-dimethyl-6-(4-methylpent-3-en-1-yl)cyclohex-1-ene (3i): Prepared according to GP4 using (*S*,*S*)-IDPi **2d** in $CHCl_3$ (4 M) at rt for 24 h, eluent: 1–2% Et_2O in *n*-pentane, 40.0 mg, colorless liquid, 83.2%.

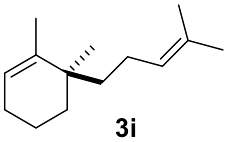

3i

^{1}H NMR (501 MHz, $CDCl_3$) δ 5.41–5.33 (m, 1H), 5.15–5.07 (m, 1H), 1.98–1.88 (m, 3H), 1.84–1.73 (m, 1H), 1.72–1.65 (m, 3H), 1.65–1.52 (m, 9H), 1.46–1.37 (m, 1H), 1.35–1.22 (m, 2H), 0.99 (s, 3H).

^{13}C NMR (126 MHz, $CDCl_3$) δ 140.4, 131.0, 125.4, 123.2, 40.1, 37.1, 34.9, 26.4, 26.1, 25.9, 23.0, 19.6, 19.0, 17.8.

HRMS *m/z* (GC-EI): calculated for $C_{14}H_{24}$ $[M]^+$: 192.1872; found: 192.1872.

The enantiomeric ratio was measured by GC (30.0 m CycloSil-B, injection temperature: 220 °C, 100 °C iso 100 min, 20 °C/min, 220 °C iso 5 min, 0.5 bar He): t_R = 75.3 min (major), t_R = 77.4 min (minor), e.r. = 95.5:4.5. $[\alpha]_D^{25}$ = –17.04 (*c* 0.98, CH_2Cl_2).

(*S*)-[((5-(1,2-dimethylcyclohex-2-en-1-yl)pentyl)oxy)methyl]benzene (3j): Prepared according to GP4 using (*S*,*S*)-IDPi **2d** in $CHCl_3$ (4 M) at rt for 24 h, eluent: 1–2% Et_2O in *n*-pentane, 62.0 mg, colorless liquid, 86.6%.

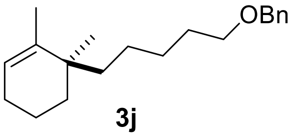

3j

^{1}H NMR (501 MHz, $CDCl_3$) δ 7.36–7.32 (m, 4H), 7.31–7.27 (m, 1H), 5.37–5.35 (m, 1H), 4.51 (s, 1H), 3.47 (t, *J* = 6.6 Hz, 1H), 1.93–1.89 (m, 2H), 1.67–1.52 (m, 8H), 1.45–1.21 (m, 6H), 1.17–1.04 (m, 1H), 0.97 (s, 3H).

^{13}C NMR (126 MHz, $CDCl_3$) δ 140.6, 138.9, 128.5, 127.8, 127.6, 123.0, 73.0, 70.7, 40.1, 37.0, 35.0, 30.0, 27.3, 26.5, 26.1, 24.1, 19.5, 19.0.

HRMS *m/z* (GC-EI): calculated for $C_{20}H_{30}O_1$ $[M]^+$: 286.2289; found: 286.2291.

The enantiomeric ratio was measured by GC (21.5 m Ivadex-5, injection temperature: 220 °C, 120 °C iso 900 min, 8 °C/min, 220 °C iso 3 min, 0.4 bar H_2): t_R = 521.2 min (major), t_R = 541.7 min (minor), e.r. = 98:2. $[\alpha]_D^{25}$ = +1.26 (*c* 0.71, CH_2Cl_2).

(*R*)-2-[2-(1,2-dimethylcyclohex-2-en-1-yl)ethyl]-1,3,5-trimethylbenzene (3k): Prepared according to GP4 using (*S*,*S*)-IDPi **2d** in $CHCl_3$ (4 M) at 50 °C for 24 h, eluent: 0.5–1% Et_2O in *n*-pentane, 57.0 mg, colorless liquid, 88.9%.

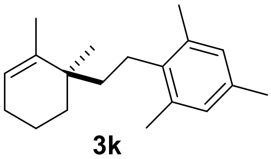
3k

^{1}H NMR (501 MHz, $CDCl_3$) δ 6.84 (s, 1H), 5.42–5.40 (m, 1H), 2.61–2.41 (m, 2H), 2.31 (s, 6H), 2.26 (s, 3H), 2.01–1.97 (m, 2H), 1.87–1.79 (m, 1H), 1.72–1.53 (m, 6H), 1.51–1.46 (m, 1H), 1.44–1.37 (m, 1H), 1.10 (s, 3H).

^{13}C NMR (126 MHz, $CDCl_3$) δ 140.4, 136.8, 136.0, 134.9, 129.0, 123.0, 39.0, 37.1, 34.9, 26.1, 25.9, 24.1, 20.9, 19.8, 19.6, 19.0.

HRMS *m/z* (GC-EI): calculated for $C_{19}H_{28}$ $[M]^+$: 256.2182; found: 256.2185.

The enantiomeric ratio was measured by GC (25.0 m Ivadex-1, injection temperature: 220 °C, 100 °C iso 710 min, 8 °C/min, 220 °C iso 3 min, 0.5 bar H_2): t_R = 662.3 min (major), t_R = 676.9 min (minor), e.r. = 97:3. $[\alpha]_D^{25}$ = –51.65 (*c* 0.57, CH_2Cl_2).

(*S*)-5-hexyl-1,5-dimethylcyclopent-1-ene (3l): Prepared according to GP4 using (*S*,*S*)-IDPi **2f** in *n*-hexane (0.5 M) at rt for 36 h, eluent: 10–16% CH_2Cl_2 in *n*-pentane, 38.0 mg, colorless liquid, 84.3%. *Note: no separation of the side product **13l** and **14l** from **3l** (^{1}H NMR ratio of **3l:13l:14l** = 97:2.5:0.5).*

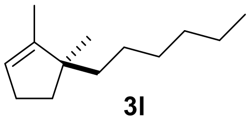
3l

^{1}H NMR (501 MHz, $CDCl_3$) δ 5.26–5.24 (m, 1H), 2.26–2.08 (m, 2H), 1.83–1.77 (m, 1H), 1.62–1.50 (m, 4H), 1.39–1.21 (m, 9H), 1.17–1.06 (m, 1H), 0.97 (s, 3H), 0.88 (t, *J* = 6.9 Hz, 3H).

^{13}C NMR (126 MHz, $CDCl_3$) δ 146.8, 123.6, 49.2, 39.6, 36.7, 32.1, 30.4, 29.7, 25.7, 24.9, 22.9, 14.3, 12.5.

HRMS *m/z* (GC-EI): calculated for $C_{13}H_{24}$ $[M]^+$: 180.1872; found: 180.1872.

The enantiomeric ratio was measured by GC (30.0 m CycloSil-B, injection temperature: 220 °C, 90 °C iso 65 min, 20 °C/min, 220 °C iso 10 min, 0.5 bar He): t_R = 49.0 min (major), t_R = 50.0 min (minor), e.r. = 90:10. $[\alpha]_D^{25}$ = –15.69 (*c* 0.45, CH_2Cl_2).

(*R*)-5-isopentyl-1,5-dimethylcyclopent-1-ene (3m): Prepared according to GP4 using (*S*,*S*)-IDPi **2f** in *n*-hexane (0.5 M) at rt for 36 h, eluent: 10–16% CH_2Cl_2 in *n*-pentane, 35.5 mg, colorless liquid, 85.4%.

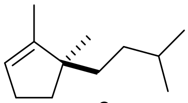
3m

^{1}H NMR (501 MHz, $CDCl_3$) δ 5.26–5.24 (m, 1H), 2.27–2.06 (m, 2H), 1.82–1.77 (m, 1H), 1.63–1.52 (m, 4H), 1.50–1.42 (m, 1H), 1.35–1.23 (m, 2H), 1.19–1.09 (m, 1H), 1.04–0.98 (m, 1H), 0.97 (s, 3H), 0.88 (d, *J* = 2.5 Hz, 3H), 0.87 (d, *J* = 2.5 Hz, 3H).

^{13}C NMR (126 MHz, $CDCl_3$) δ 146.8, 123.6, 49.1, 37.1, 36.6, 34.0, 29.7, 28.9, 25.8, 22.9, 22.9, 12.5.
HRMS *m/z* (GC-EI): calculated for $C_{12}H_{22}$ $[M]^+$: 166.1717; found: 166.1716.
The enantiomeric ratio was measured by GC (30.0 m CycloSil-B, injection temperature: 220 °C, 70 °C iso 65 min, 20 °C/min, 220 °C iso 10 min, 0.5 bar He): t_R = 41.4 min (major), t_R = 42.7 min (minor), e.r. = 93:7. $[\alpha]_D^{25}$ = –14.88 (*c* 0.41, CH_2Cl_2).

(*R*)-5-(3,3-dimethylbutyl)-1,5-dimethylcyclopent-1-ene (3n): Prepared according to GP4 using (*S*,*S*)-IDPi **2f** in *n*-hexane (0.5 M) at rt for 36 h, eluent: 10–16% CH_2Cl_2 in *n*-pentane, 38.0 mg, colorless liquid, 84.3%.

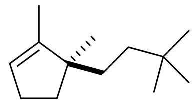
3n

^{1}H NMR (501 MHz, $CDCl_3$) δ 5.27–5.25 (m, 1H), 2.30–2.05 (m, 2H), 1.82–1.75 (m, 1H), 1.60–1.51 (m, 4H), 1.33–1.21 (m, 2H), 1.20–1.10 (m, 1H), 1.96–0.96 (s, 4H), 0.86 (s, 9H).
^{13}C NMR (126 MHz, $CDCl_3$) δ 146.7, 123.7, 49.0, 38.7, 36.4, 33.7, 30.2, 29.7, 29.6, 26.0, 12.5.
HRMS *m/z* (GC-EI): calculated for $C_{13}H_{24}$ $[M]^+$: 180.1872; found: 180.1872.
The enantiomeric ratio was measured by GC (30.0 m CycloSil-B, injection temperature: 220 °C, 70 °C iso 75 min, 20 °C/min, 220 °C iso 10 min, 0.5 bar He): t_R = 56.9 min (major), t_R = 58.8 min (minor), e.r. = 95:5. $[\alpha]_D^{25}$ = –7.3 (*c* 1.35, CH_2Cl_2).

(*S*)-1,6-dimethyl-1,2,3,4-tetrahydro-1,1'-biphenyl (3o): Prepared according to GP4 using (*S*,*S*)-IDPi **2g** in MeCy (4 M) at 60 °C for 48 h, eluent: 5–12% CH_2Cl_2 in *n*-pentane, 43.2 mg, colorless liquid, 93%.

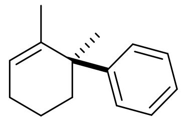
3o

^{1}H NMR (501 MHz, $CDCl_3$) δ 7.35–7.28 (m, 4H), 7.22–7.15 (m, 1H), 5.69–5.63 (m, 1H), 2.15–1.99 (m, 2H), 1.82–1.74 (m, 1H), 1.74–1.64 (m, 1H), 1.55–1.53 (m, 3H), 1.52–1.49 (m, 1H), 1.47 (s, 3H), 1.45–1.36 (m, 1H).
^{13}C NMR (126 MHz, $CDCl_3$) δ 148.8, 138.8, 128.0, 127.2, 125.6, 124.6, 42.7, 41.9, 26.2, 26.0, 20.2, 19.0.
HRMS *m/z* (GC-EI): calculated for $C_{14}H_{18}$ $[M]^+$: 186.1403; found: 186.1403.
The enantiomeric ratio was measured by GC (30.0 m BGB-176, injection temperature: 220 °C, 80–125°C (0.7 °C/min) iso 20 min, 20 °C/min, 220 °C iso 5 min,, 0.5 bar He): t_R = 62.1 min (major), t_R = 63.2 min (minor), e.r. = 95:5. $[\alpha]_D^{25}$ = –87.81 (*c* 0.93, CH_2Cl_2).

(*S*)-3'-methoxy-1,6-dimethyl-1,2,3,4-tetrahydro-1,1'-biphenyl (3p): Prepared according to GP4 using (*S*,*S*)-IDPi **2g** in MeCy (4 M) at 60 °C for 72 h, eluent: 20–30% CH_2Cl_2 in *n*-pentane, 51.0 mg, colorless liquid, 94%.

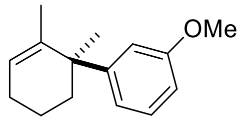

3p

^{1}H NMR (501 MHz, $CDCl_3$) δ 7.31–7.23 (m, 1H), 6.99–6.93 (m, 1H), 6.94–6.90 (m, 1H), 6.77 (ddd, *J* = 8.1, 2.6, 0.9 Hz, 1H), 5.71–5.64 (m, 1H), 3.84 (s, 3H), 2.18–2.01 (m, 2H), 1.80

(ddd, J = 12.9, 7.1, 3.2 Hz, 1H), 1.71 (ddd, J = 13.2, 10.3, 3.1 Hz, 1H), 1.57 (q, J = 2.0 Hz, 3H), 1.55–1.50 (m, 1H), 1.48 (s, 3H), 1.47–1.40 (m, 1H).

^{13}C NMR (126 MHz, $CDCl_3$) δ 159.5, 150.7, 138.7, 128.9, 124.7, 119.7, 114.0, 110.1, 55.3, 42.8, 41.7, 26.2, 26.0, 20.2, 19.0.

HRMS *m/z* (GC-EI): calculated for $C_{15}H_{12}O$ $[M]^+$: 216.1510; found: 216.1508.

The enantiomeric ratio was measured by GC (30.0 m BGB-176, injection temperature: 220 °C, 130 °C iso 90 min, 20 °C/min, 220 °C iso 10 min, 0.5 bar He): t_R = 71.0 min (major), t_R = 71.9 min (minor), e.r. = 97:3. $[\alpha]_D^{25}$ = –87.9 (c 1.69, CH_2Cl_2).

(*S*)-3'-fluoro-1,6-dimethyl-1,2,3,4-tetrahydro-1,1'-biphenyl (3q): Prepared according to GP4 using (*S*,*S*)-IDPi **2g** in MeCy (4 M) at 80 °C for 96 h, eluent: 4–8% CH_2Cl_2 in *n*-pentane, 47.0 mg, colorless liquid, 92%.

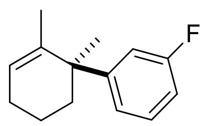

3q

^{1}H NMR (501 MHz, $CDCl_3$) δ 7.33–7.20 (m, 1H), 7.12–7.06 (m, 1H), 7.05–6.98 (m, 1H), 6.95–6.77 (m, 1H), 5.69–5.60 (m, 1H), 2.17–1.96 (m, 2H), 1.80–1.63 (m, 2H), 1.52 (q, J = 1.9 Hz, 3H), 1.51–1.47 (m, 1H), 1.45 (s, 3H), 1.43–1.34 (m, 1H).

^{13}C NMR (126 MHz, $CDCl_3$) δ 163.0 (d, J = 243.8 Hz), 151.8 (d, J = 6.3 Hz), 138.2, 129.3 (d, J = 8.1 Hz), 125.1, 122.7 (d, J = 2.6 Hz), 114.3 (d, J = 21.6 Hz), 112.4 (d, J = 21.1 Hz), 42.8, 41.6, 26.1, 25.9, 20.1, 18.9.

^{19}F NMR (471 MHz, $CDCl_3$) δ –114.07.

HRMS *m/z* (GC-EI): calculated for $C_{14}H_{17}F$ $[M]^+$: 204.1312; found: 204.1308.

The enantiomeric ratio was measured by GC (30.0 m BGB-176, injection temperature: 220 °C, 115 °C iso 55 min, 20 °C/min, 220 °C iso 10 min, 0.5 bar He): t_R = 40.7 min (major), t_R = 41.5 min (minor), e.r. = 97:3. $[\alpha]_D^{25}$ = –90.45 (c 0.64, CH_2Cl_2).

(*S*)-1,6-dimethyl-4'-(trifluoromethyl)-1,2,3,4-tetrahydro-1,1'-biphenyl (3r): Prepared according to GP4 using (*S*,*S*)-IDPi **2g** in MeCy (4 M) at 60 °C for 6 days, eluent: *n*-pentane to 3% CH_2Cl_2 in *n*-pentane, 57.0 mg, colorless liquid, 90%.

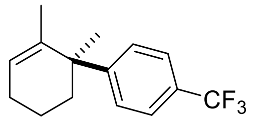

3r

^{1}H NMR (501 MHz, $CDCl_3$) δ 7.55 (d, J = 8.3 Hz, 2H), 7.43 (d, J = 8.0 Hz, 2H), 5.71–5.66 (m, 1H), 2.15–1.98 (m, 2H), 1.84–1.66 (m, 2H), 1.56–1.48 (m, 4H), 1.48 (s, 3H), 1.41–1.31 (m, 1H).

^{13}C NMR (126 MHz, $CDCl_3$) δ 53.1, 137.9, 127.54, 125.36, 128.0 (q, J = 32.4 Hz), 125.0 (q, J = 3.8 Hz), 124.6 (q, J = 272.8 Hz), j42.9, 41.7, 26.0, 25.9, 20.1, 18.8.

^{19}F NMR (471 MHz, $CDCl_3$) δ –62.23.

HRMS *m/z* (GC-EI): calculated for $C_{15}H_{17}F_3$ $[M]^+$: 254.1275; found: 254.1276.

The enantiomeric ratio was measured by GC (30.0 m BGB-176, injection temperature: 220 °C, 130 °C iso 45 min, 20 °C/min, 220 °C iso 10 min, 0.5 bar He): t_R = 25.4 min (major), t_R = 27.2 min (minor), e.r. = 97:3. $[\alpha]_D^{25}$ = –76.33 (*c* 0.62, CH_2Cl_2).

(*S*)-(1,2-dimethylcyclopent-2-en-1-yl)benzene (3s): Prepared according to GP4 using (*S*,*S*)-IDPi **2d** in MeCy (4 M) at 50 °C for 24 h, eluent: 10–15% CH_2Cl_2 in *n*-pentane, 32.5 mg, colorless liquid, 75%.

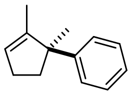

3s

^{1}H NMR (501 MHz, $CDCl_3$) δ 7.35–7.29 (m, 4H), 7.22–7.16 (m, 1H), 5.58–5.50 (m, 1H), 2.41–2.28 (m, 2H), 2.17–2.06 (m, 1H), 2.06–1.97 (m, 1H), 1.51 (q, *J* = 2.0 Hz, 3H), 1.47 (s, 3H).

^{13}C NMR (126 MHz, $CDCl_3$) δ 148.6, 146.8, 128.2, 126.4, 125.6, 125.5, 53.8, 43.4, 30.1, 24.1, 13.2.

HRMS *m/z* (GC-EI): calculated for $C_{13}H_{16}$ $[M]^+$: 172.1244; found: 172.1246.

The enantiomeric ratio was measured by GC (30.0 m CycloSil-B, injection temperature: 220 °C, 110 °C iso 50 min, 20 °C/min, 220 °C iso 10 min, 0.5 bar H_2): t_R = 35.9 min (major), t_R = 37.2 min (minor), e.r. = 77:23.

(*S*)-1-(1,2-dimethylcyclopent-2-en-1-yl)-3-methylbenzene (3t): Prepared according to GP4 using (*S*,*S*)-IDPi **2d** in MeCy (4 M) at 50 °C for 24 h, eluent: 10–15% CH_2Cl_2 in *n*-pentane, 34.5 mg, colorless liquid, 74%.

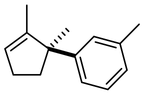

3t

^{1}H NMR (501 MHz, $CDCl_3$) δ 7.20 (t, *J* = 7.9 Hz, 1H), 7.13–7.05 (m, 2H), 7.01 (d, *J* = 7.4 Hz, 1H), 5.54–5.52 (m, 1H), 2.50–2.27 (m, 5H), 2.09 (dt, *J* = 13.8, 6.6 Hz, 1H), 2.00 (dt, *J* = 12.8, 7.2 Hz, 1H), 1.51 (q, *J* = 1.9 Hz, 3H), 1.46 (s, 3H).

^{13}C NMR (126 MHz, $CDCl_3$) δ 148.6, 146.9, 137.6, 128.1, 127.1, 126.4, 125.3, 123.4, 53.7, 43.4, 30.1, 24.2, 21.8, 13.3.

HRMS *m/z* (GC-EI): calculated for $C_{14}H_{18}$ $[M]^+$: 186.1403; found: 186.1403.

The enantiomeric ratio was measured by GC (30.0 m CycloSil-B, injection temperature: 220 °C, 115 °C iso 60 min, 20 °C/min, 220 °C iso 10 min, 0.5 bar H_2): t_R = 44.0 min (major), t_R = 45.2 min (minor), e.r. = 76:24.

3b. Enantioselective total synthesis of (–)-herbertene:

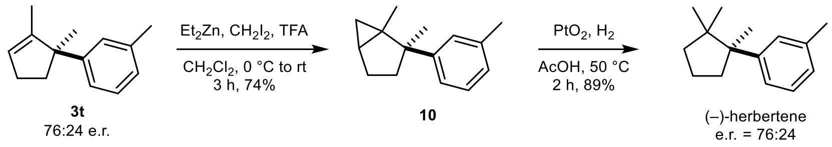

(2S)-1,2-dimethyl-2-(m-tolyl)bicyclo[3.1.0]hexane (10): Trifluroacetic acid (0.30 mL, 4.03 mmol, 5.0

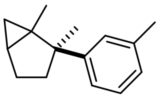

10

equiv.) was added dropwise to a stirred solution of Et_2Zn (1 M *n*-hexane, 4.03 mL, 4.03 mmol, 5.0 equiv.) in CH_2Cl_2 (18 mL) at 0 °C (ice bath) under Ar. After 20 min. of stirring at 0 °C, diiodomethane (0.32 mL, 4.03 mmol, 5.0 equiv.) was added dropwise. After 20 min. of stirring, a solution of **3t** (150.0 mg, 0.80 mmol, 1.0 equiv.) in 2 mL of CH_2Cl_2 was added over 7 min. The resulting mixture was warmed to rt over 3 h while stirring. The reaction mixture was cooled to 0 °C and quenched carefully with saturated aqueous NH_4Cl and extracted with Et_2O (3x20 mL) and the combined organic layer was washed with brine, dried over anhydrous Na_2SO_4, concentrated in vacuo. The crude reaction mixture was purified by silica-gel flash column chromatography (*n*-pentane) to provide the cyclopropane **10** as a colorless liquid (120 mg, 74% yield, ca. 2.3:1 mixture of two isomers).

^{1}H NMR (501 MHz, $CDCl_3$) (major product) δ 7.24–7.15 (m, 3H), 7.06–6.95 (m, 1H), 2.36 (s, 3H), 2.15–1.96 (m, 1H), 1.66 (dd, *J* = 12.7, 8.0 Hz, 1H), 1.52–1.43 (m, 1H), 1.43 (s, 3H), 1.41–1.34 (m, 2H), 0.92 (s, 3H), 0.48 (t, *J* = 4.1 Hz, 1H), 0.27 (ddd, *J* = 8.0, 4.7, 1.1 Hz, 1H).

^{13}C NMR (126 MHz, $CDCl_3$) (major product) δ 148.8, 137.4, 127.9, 127.4, 126.2, 123.9, 47.9, 39.2, 32.9, 27.11, 27.09, 22.3, 22.0, 18.0, 14.5.

^{13}C NMR (126 MHz, $CDCl_3$) (minor product) δ 149.1, 137.4, 127.9, 127.4, 126.1, 123.6, 48.3, 38.1, 29.1, 26.3, 25.6, 23.7, 21.7, 18.3, 15.8.

HRMS *m/z* (APPIpos): calculated for $C_{15}H_{21}$ $[M+H]^+$: 201.1638; found: 201.1637.

Note: without TFA reaction is very sluggish, several equiv. of Et_2Zn and diiodomethane need to be added over time. The leftover starting material can be separated via flash column chromatography on silver nitrate impregnated silica gel. However, traces of silver nitrate form the purified product need to be removed, else next hydrogenolysis of cyclopropane using PtO_2 underwent uncontrolled and full decomposition of product observed at rt.

(–)-herbertene[15]: To a solution of **10** (40 mg, 0.199 mmol, 1.0 equiv.) in acetic acid (1.5 mL) was added

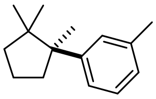

(–)-herbertene

PtO_2 (90.68 mg, 0.399 mmol, 2.0 equiv.). The mixture was stirred under 1 atm hydrogen at 50 °C for 2 h. The reaction mixture was cooled to rt and filtered through Celite pad, washed with Et_2O and the filtrate was evaporated in vacuo. The crude reaction mixture was purified by silica-gel flash column chromatography (*n*-pentane) to provide the (–)-herbertene as a colorless liquid (36 mg, 89%).

Note: Reaction progress was monitored by ^{1}H NMR spectroscopy for every 30 min., longer reaction time can led to a decomposition or over reduction of product.

^{1}H NMR (501 MHz, $CDCl_3$) δ 7.20–7.12 (m, 3H), 7.05–6.96 (m, 1H), 2.51 (dt, *J* = 12.8, 8.9 Hz, 1H), 2.35 (s, 3H), 1.84–1.74 (m, 2H), 1.73–1.64 (m, 2H), 1.60–1.54 (m, H), 1.26 (s, 3H), 1.07 (s, 3H), 0.56 (s, 3H).

^{13}C NMR (126 MHz, $CDCl_3$) δ 147.7, 136.9, 128.0, 127.5, 126.2, 124.3, 50.6, 44.4, 39.9, 36.9, 26.65, 24.59, 24.5, 21.94, 19.86.

The enantiomeric ratio was measured by GC (24.0 m Cyclodextrin-H, injection temperature: 220 °C, 90 °C iso 35 min, 8 °C/min, 180 °C iso 10 min, 0.5 bar H_2): t_R = 29.36 min (minor), t_R = 30.80 min (major), e.r. = 76:24. $[\alpha]_D^{20}$ = –35.6 (*c* 0.99, $CHCl_3$); lit.[15] $[\alpha]_D^{20}$ = –46.5 (c = 1.0, $CHCl_3$).

3c. ^{13}C Labelled exocyclic olefin isomerization:

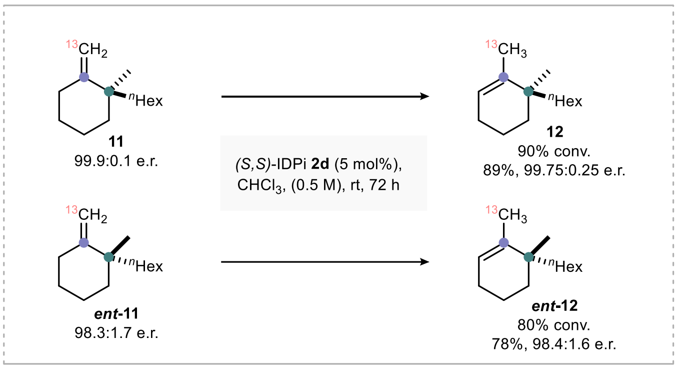

Figure S2: Reactions conducted on 0.03 mmol scale. Conversions and yields were determined by ^{1}H NMR using 1,3,5-trimethoxybenzene as internal standard. The e.r. was determined by GC.

(*S*)-6-hexyl-6-methyl-1-(methyl-^{13}*C*)cyclohex-1-ene (12): Following GP4, using (*S*,*S*)-IDPi **2d** in $CHCl_3$ (0.5 M) at rt for 72 h, 90% conv., 89%, e.r. = 99.75:0.25.

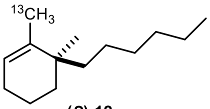

(*S*)-12

^{1}H NMR (600 MHz, $CDCl_3$) δ 5.39–5.32 (m, 1H), 1.94–1.88 (m, 2H), 1.58 (dq, $^{1}J_{CH}$ = 125.0, *J* = 1.8 Hz, 3H), 1.64–1.51 (m, 3H), 1.43–1.34 (m, 1H), 1.34–1.20 (m, 9H), 1.13–1.04 (m, 1H), 0.97 (s, 3H), 0.91–0.85 (m, 3H).

^{13}C NMR (151 MHz, $CDCl_3$) δ 140.7 (d, *J* = 43.6 Hz), 122.9 (d, *J* = 3.0 Hz), 40.2 (d, *J* = 1.4 Hz), 37.0 (d, *J* = 2.2 Hz), 35.0 (d, *J* = 1.5 Hz), 32.1, 30.5, 26.6 (d, *J* = 1.1 Hz), 26.1 (d, *J* = 4.5 Hz), 24.2, 22.9, 19.6, 19.1, 14.3.

HRMS *m/z* (GC-EI): calculated for $C_{13}H_{26}{}^{13}C$ $[M]^+$: 195.2061; found: 195.2062.

3d. Isolation of olefin isomerized starting material (6a):

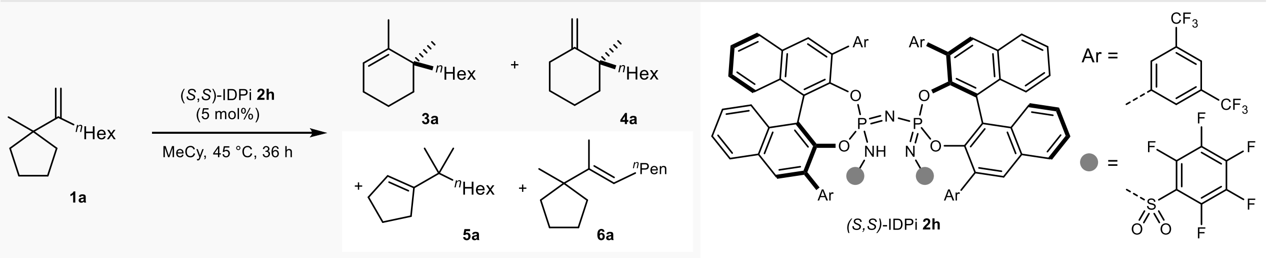

In a 2 mL glass vial, the IDPi **2h** (24.08 mg, 0.0125 mmol, 5 mol%) was measured and it was dissolved in MeCy (125 µL, 2 M). At rt, substrate **1a** (48.59 mg, 0.25 mmol, 1.0 equiv.) was added. The reaction mixture was stirred 45 °C for 36 h. ^{1}H NMR ratio of **3a:4a:5a:6a** = 22:3:2:73. The crude reaction mixtures were directly purified *via* flash column chromatography on silver nitrate impregnated silica gel (eluent: 4–15% CH_2Cl_2 in *n*-pentane) to give the inseparable mixture of isomerized side products **6a** and methyl migrated side product **5a** as a colorless liquid (22.6 mg, 48%, by ^{1}H NMR ratio of **6a:5a** = 97:3).

(E)-1-methyl-1-(oct-2-en-2-yl)cyclopentane (6a):

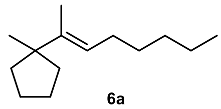
6a

^{1}H NMR (600 MHz, $CDCl_3$) δ 5.16 (tq, *J* = 6.9, 1.3 Hz, 1H), 1.97 (qq, *J* = 7.2, 0.8 Hz, 2H), 1.68–1.62 (m, 4H), 1.61 (q, *J* = 1.3 Hz, 5H), 1.45–1.37 (m, 2H), 1.36–1.21 (m, 6H), 1.01 (s, 3H), 0.89 (t, *J* = 7.1 Hz, 3H).

^{13}C NMR (151 MHz, $CDCl_3$) δ 142.29, 121.71, 48.78, 37.78, 31.79, 29.76, 28.16, 26.23, 23.76, 22.79, 14.27, 13.83.

HRMS *m/z* (GC-EI): calculated for $C_{14}H_{26}$ $[M]^+$: 194.2028; found: 194.2029.

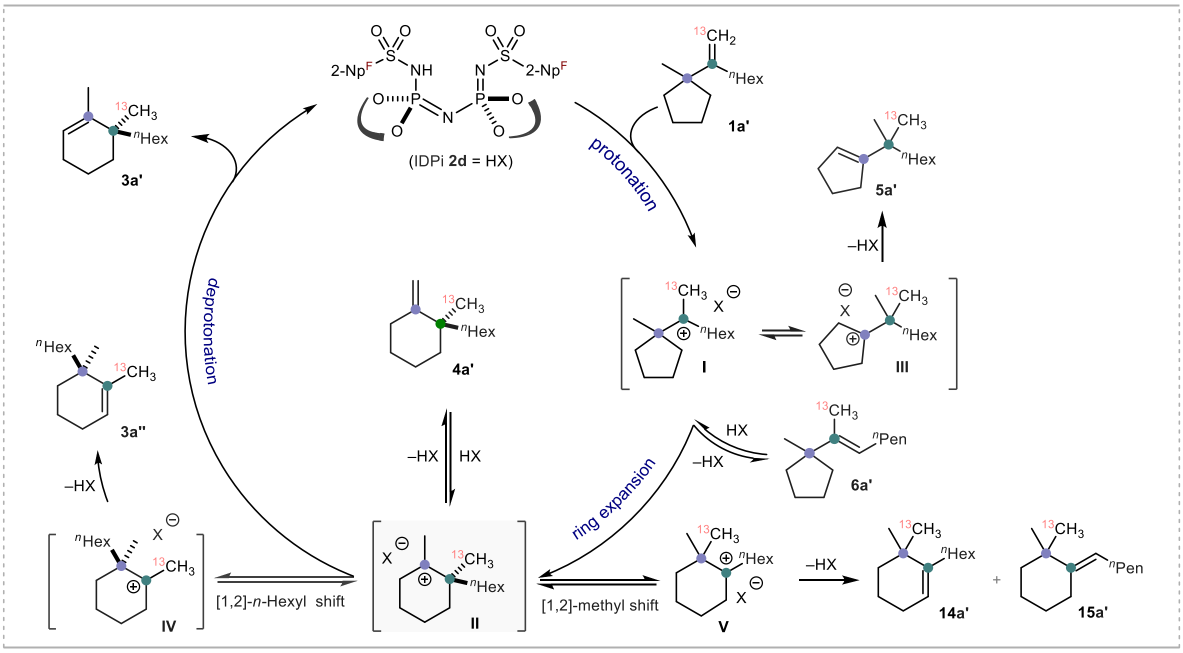

Figure S3: A plausible catalytic cycle.

4. NMR kinetics and mechanistic studies

General NMR data processing

The acquired NMR data was imported with the Reaction Monitoring Plugin into MNOVA 14.3.2 and processed therein (baseline correction, phase correction, integration). Generally, the first acquired NMR spectrum was used as absolute concentration reference by using a characteristic signal of the starting material (**1a** or **1a'**) for the following spectra. At this point, no significant conversion was observed at rt.

Note on the reaction concentration

A realistic reaction concentration used for the initial NMR concentration referencing was based on measuring the total reaction volume at the end of the reactions. As the mass balance based on the NMR integral was found constant throughout the reaction, we concluded that there were no significant volume changes occurring during the reaction and the volume at the end of the reaction can be assumed equal to the reaction. After mixing substrate **1a** or **1a'** (0.25 mmol, 1.0 equiv.), IDPI **2d** and 125 μL $CDCl_3$ a total reaction volume of 195 μL was measured.

4a. ^{13}C NMR reaction monitoring:

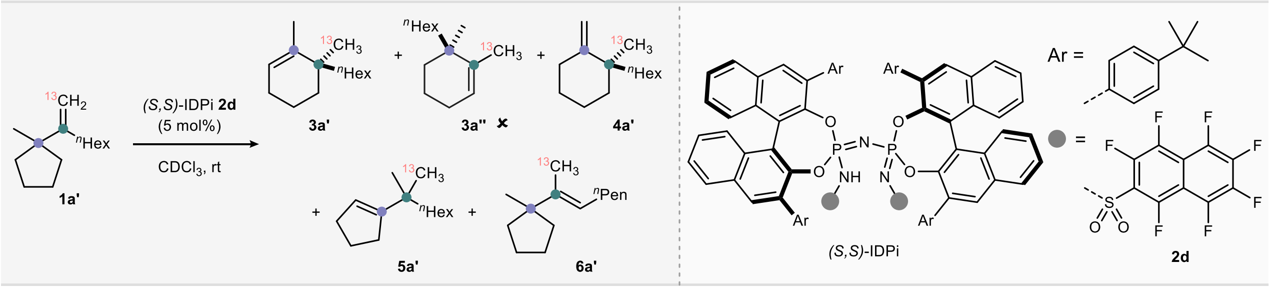

Sample preparation and data acquisition

In an oven-dried (80 °C, overnight) 5 mm heavy wall J. Young NMR tube under Ar, IDPi **2d** (22.95 mg, 5 mol%) dissolved in anhydrous $CDCl_3$ (125 μL) and the substrate (48.83 mg, 0.25 mmol, 1.0 equiv.) was added. The NMR tube was turned upside down and vortexed for 10 s to ensure mixing of all the components. Afterwards the NMR tube was turned once again and the solution was vortexed again to move all the solution back to the bottom of the J. Young NMR tube. Afterwards, the sample was quickly transferred to a Bruker 600 MHz NMR magnet equipped with a TCI cryoprobe and after shimming and tuning & matching single scan $^{13}C[^{1}H]$ NMR spectra (using waltz16 CPD) were acquired every 5 min for a period of approx. 28 h. At this point, the reaction was completed, but it was monitored to an extended time to obtain insides into the stability of the product under reaction conditions (Figure S4).

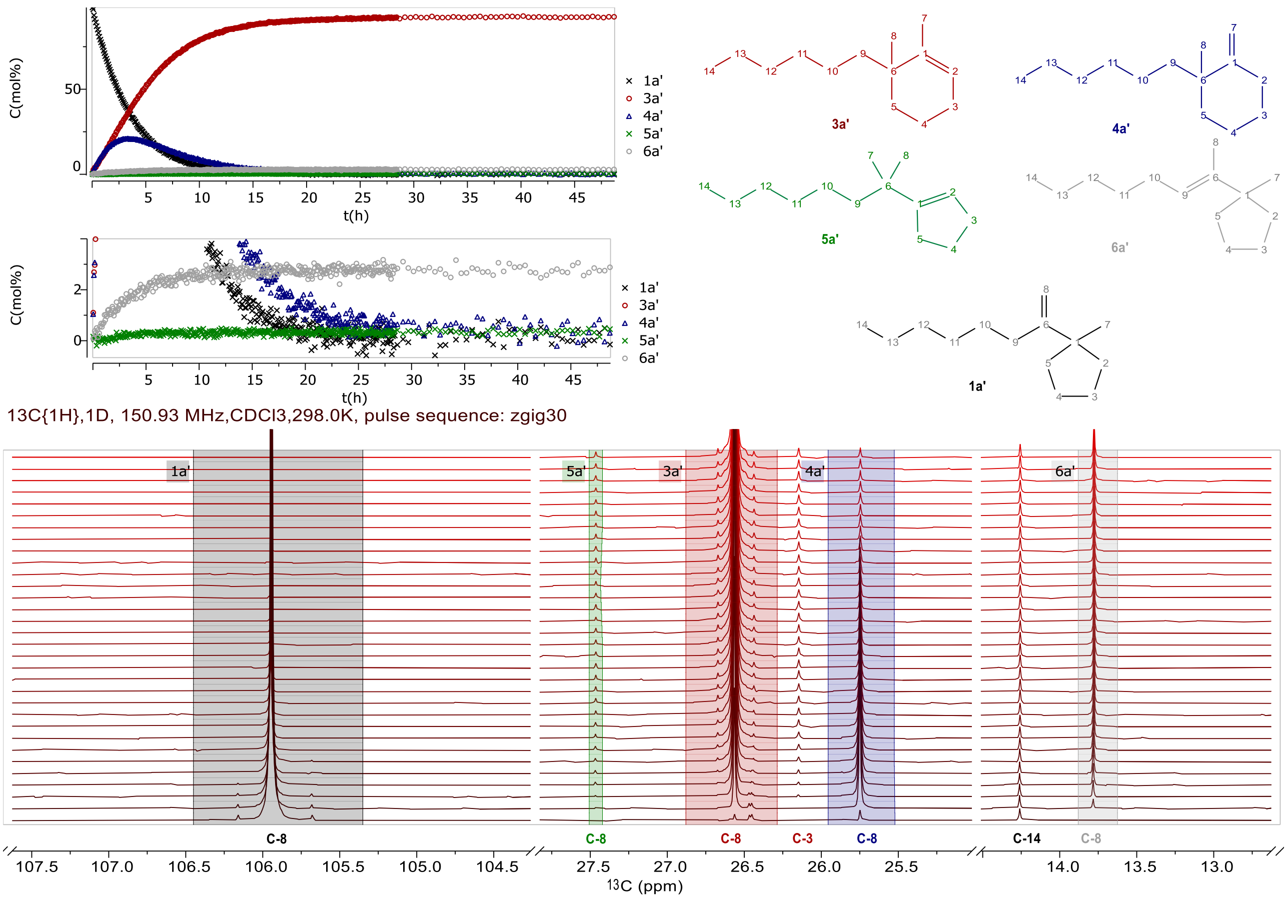

Figure S4: Overview of the single scan ^{13}C NMR reaction monitoring data. The top left shows the relative concentration profiles following the fate of ^{13}C-8 using the initial signal of ^{13}C-8 as concentration reference for all the following spectra. The structures shown on the left show the arbituary numberings used for the atom assignments. The bottom figure shows exerpts of ^{13}C NMR spectra in regions of interest taken at different time points (approx. every 60 min) with the integration regions used to generate the concentration profiles.

4b. ^{1}H NMR reaction monitoring:

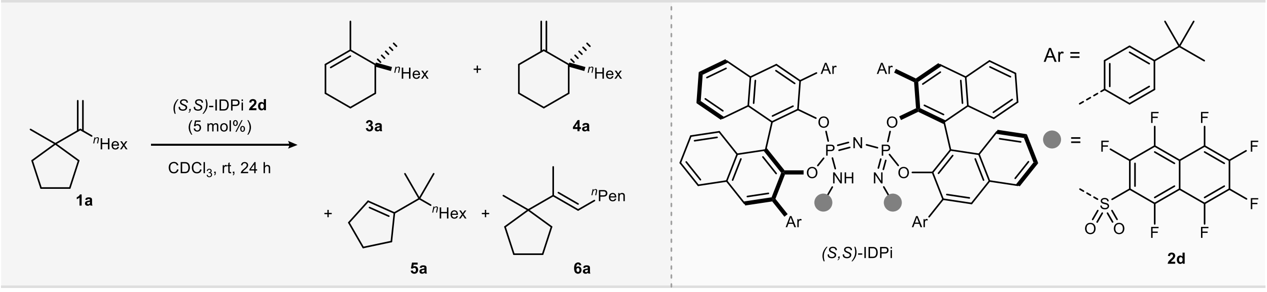

Sample preparation and data acquisition

In an oven-dried (80 °C, overnight) 5 mm heavy wall J. Young NMR tube under Ar, IDPi **2d** (22.95 mg, 5 mol%) dissolved in anhydrous $CDCl_3$ (125 μL) and the substrate (48.83 mg, 0.25 mmol, 1.0 equiv.) was added. The NMR tube was turned upside down and vortexed for 10 s to ensure mixing of all the components. Afterwards the NMR tube was turned once again and the solution was vortexed again to move all the solution back to the bottom of the J. Young NMR tube. Afterwards, the sample was quickly transferred to a Bruker 500 MHz NMR magnet preheated to the reaction temperature and

after shimming and tuning & matching single scan ^{1}H NMR spectra were acquired every 5 min until full conversion was observed.

The following Figure S5 shows ^{1}H NMR spectra taken at different time points during the reaction at 298 K in presence of IDPI **2d** (5 mol%). The reaction profile from this is in qualitative agreement with the previous reaction with the ^{13}C labeled compound **1a'**. The concentration of the free catalyst is found constant during the reaction. Additionally, no significant shifts of the signals were observed. This result is consistent with ^{31}P NMR data acquired during the exact same reaction (Figure S6) and therefore it can be concluded an unbound catalyst species the resting state throughout the reaction and no major catalyst decomposition occurred during the reaction.

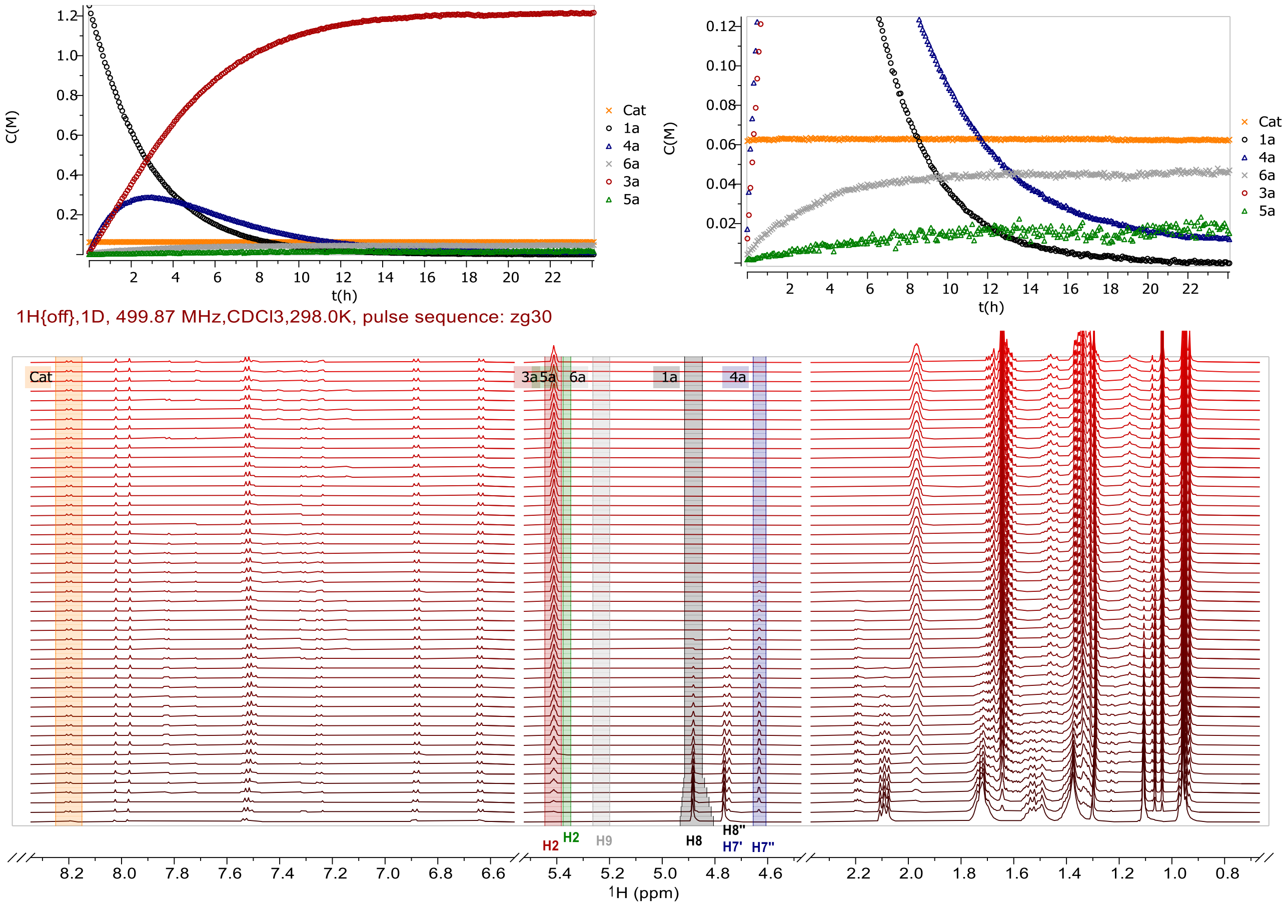

Figure S5: NMR concentration profiles (top) and ^{1}H NMR spectra at different time points (bottom) during the reaction of **1a** (48.83 mg, 0.25 mmol, 1.0 equiv.) in presence of IDPi **2d** (22.95 mg, 5 mol%) in 125 µL $CDCl_3$ showing the integration regions for the generation of the NMR concentration profiles.

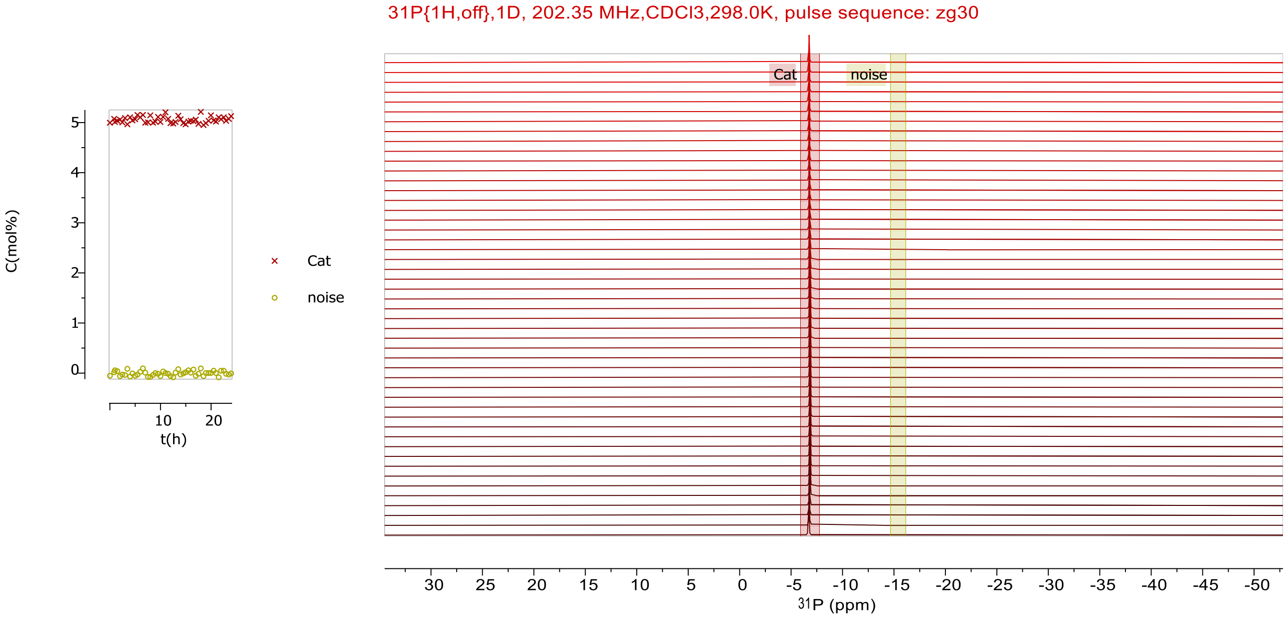

Figure S6: Left: Catalyst concentration profile extracted from ^{31}P NMR reaction data, right: ^{31}P NMR spectra at different time points during the reaction of **1a** (48.83 mg, 0.25 mmol, 1.0 equiv.) in presence of IDPi **2d** (22.95 mg, 5 mol%) in 125 µL $CDCl_3$

4c. Determination of catalyst order:

In order to determine the reaction order of the catalyst with Variable Time Normalization Analysis (VTNA)[16], the reaction of **1a** was monitored at two different concentrations of IDPI **2d** (5 mol% and 2.6 mol%). Figure S7 shows the concentration profiles of **1a**, the kinetic reaction product **4a** and the thermodynamic product **3a** with time scales normalized to a zeroth, first and second reaction order of the catalyst. For all three species, the best overlap of the two reaction profiles were observed when the profiles are normalized to a first order dependence in catalyst concentration. We therefore conclude that a single catalyst molecule is involved in the substrate activation as well in the isomerization from **4a** to **3a**.

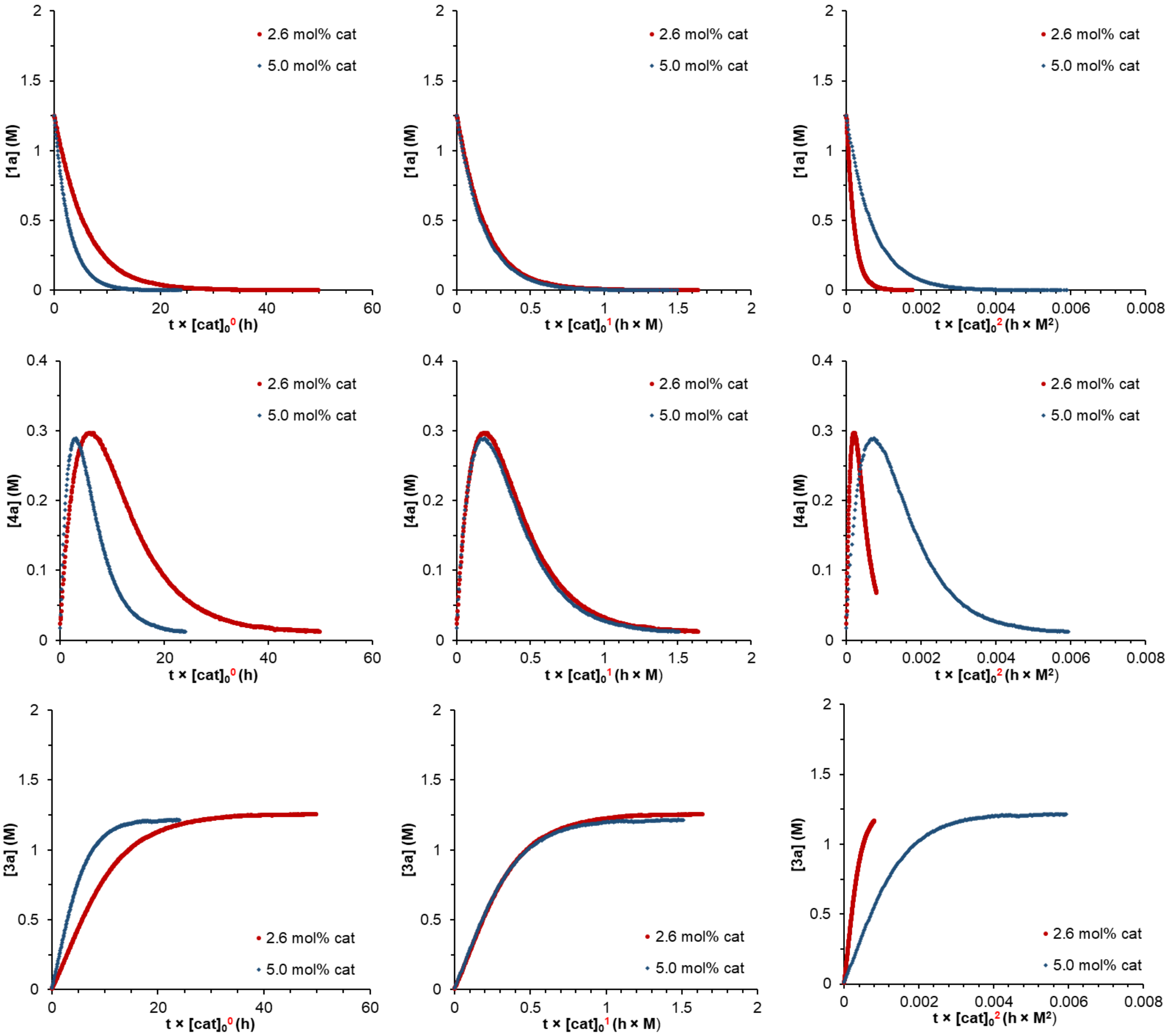

Figure S7: ^{1}H NMR concentrations profiles in $CDCl_3$ at 298K in presence of 2.6 mol% and 5.0 mol% IDPi **2d** of **1a** (first row), **4a** (2nd row) and **3a** (3rd row) with time scales normalized to a zeroth (left), first (middle) and second (right) order in catalyst concentration.

4d. Eyring analysis:

The decay of the substrate **1a** during the reaction can be described with a single exponential decay with no signs of catalyst decomposition or product deactivation. Therefore, the reactions seems suitable to determine the thermodynamic activation parameter from an Eyring analysis.

All of the acquired data suggests that reaction rate of substrate **1a** can be described by the following simple rate law:

$$\frac{d[1\text{a}]}{dt} = k\ [\text{cat}]_0^1[1\text{a}]^1$$

After integration, the concentration [**1a**] at each point of the reaction at time *t* can be described with the following equation:

$$[\mathbf{1a}] = [\mathbf{1a}]_0\ e^{-k_{obs}t}$$

$[1\text{a}]_0$ is the initial concentration of the substrate and $k_{obs} = k\ [\text{cat}]_0^1$.

From concentration profiles of **1a** at different temperatures T k_{obs} values were extracted by non-linear curve fit in Origin 2019b. An overview of the plots and the fitting results at the individual temperatures are shown in Figure S8. A summary of all the extracted values from the NMR data used for the Eyring analysis are shown in Table S4. The physical constants used for calculation are given in Table S3. When plotting k against T, $\Delta H^{\ddagger}$ and $\Delta S^{\ddagger}$ can be determined from non-linear regression by fitting the data to the Eyring equation: $k = \frac{k_bT}{h} e^{\frac{\Delta S^{\ddagger}}{R}} e^{-\frac{\Delta H^{\ddagger}}{RT}}$. An overview of the results from the non-linear fitting is presented in Table S5. This data was then used to derive the parameters $\Delta G_{\mathrm{T}}^{\ddagger}$ (=$\Delta H^{\ddagger}$-$T\Delta S^{\ddagger}$) (Table S6). The Eyring plot including the non-linear fitting result are shown in Figure S9.

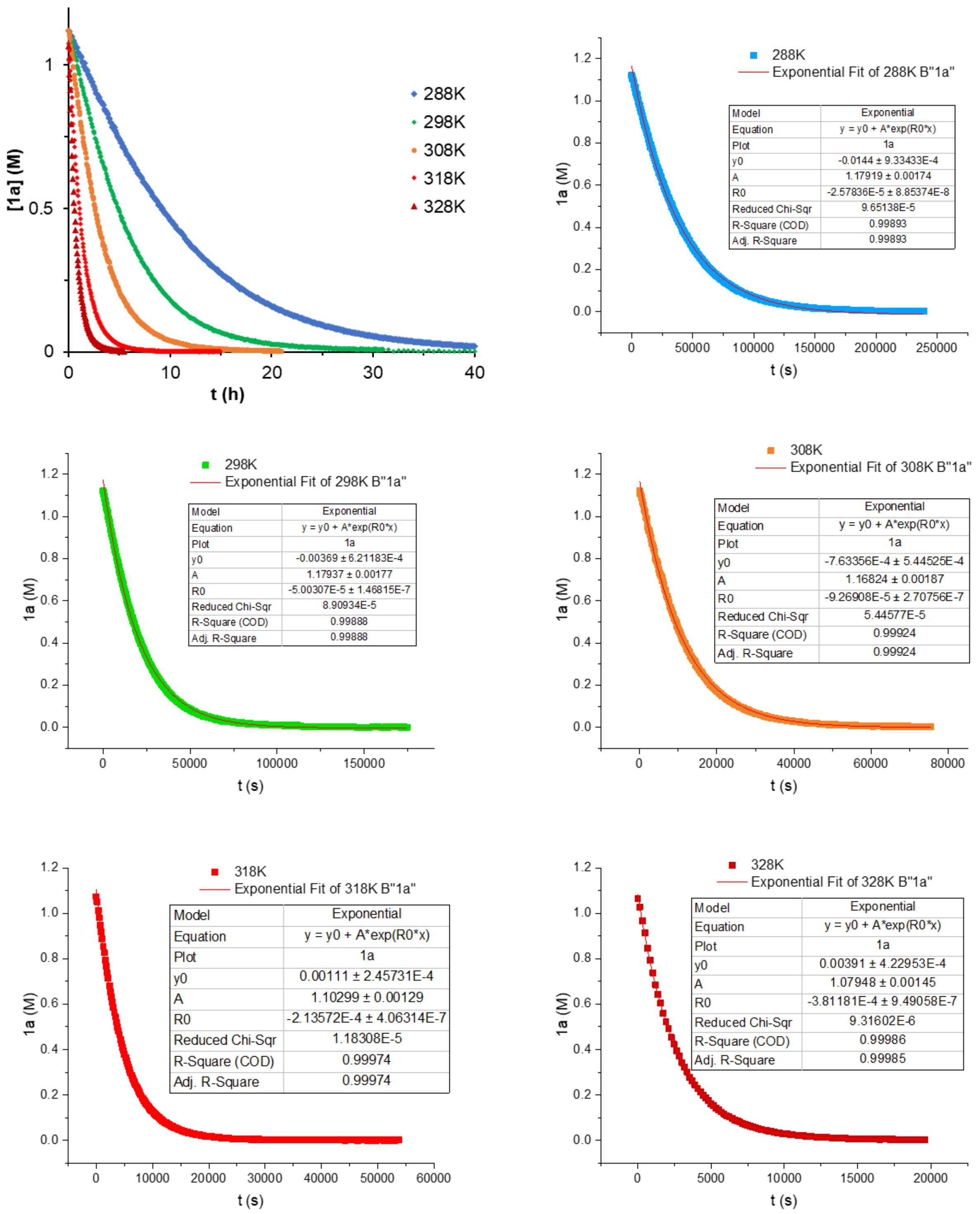

Figure S8: Concentrations graphs showing the decay of **1a** during the reaction at different temperatures from 288 K – 328 K. The insets in the concentration profiles show the result from a non-linear curve fitting in Origin.

	value	unit
R	8.3144598	J K^{-1} mol^{-1}
h	6.62607E-34	J s
k_B	1.38064852E-23	J K^{-1}

Table S3: Physical constants used for the Eyring analysis.

#	T (K)	k_{obs} (s^{-1})	$[cat]_0$ (M)	k ($M^{-1}\times s^{-1}$)
**1**	308	0.00009269 ± 0.00000027	0.0518 ± 3%	0.001789 ± 0.000059
**2**	328	0.0003812 ± 0.0000009	0.0523 ± 3%	0.007288 ± 0.000237
**3**	318	0.0002136 ± 0.0000004	0.0514 ± 3%	0.004157 ± 0.0001326
**4**	288	0.00002578 ± 0.00000009	0.0550 ± 3%	0.0004687 ± 0.0000157
**5**	298	0.00005004 ± 0.00000015	0.0542 ± 3%	0.0009235 ± 0.0000304

Table S4: Overview of all parameters with estimated errors used for the Eyring analysis.

Notes

Description	Nonlinear Curve Fit
User Name	leutzsch
Operation Time	
Iteration Algorithm	Levenberg Marquardt
Model	EyringEquation (User)
Number of Parameters	2
Number of Derived Parameters	0
Number of Datasets	1
Equation	(kb*T/h)*exp(-dH/(R*T))*exp(dS/R)
Report Status	New Analysis Report
Special Input Handling	
Data Filter	No

Input Data

		Dep/Indep	Data	Range	Weight Type	Weight Data
k	T	Indep	[Book1]Sheet1!A"T"	[1*:5*]	No Weighting	
	k	Dep	[Book1]Sheet1!B"k"	[1*:5*]	Instrumental (=1/ei^2)	[Book1]Sheet1!C"k"

Parameters

		Value	Standard Error	t-Value	Prob>|t|	Dependency
k	dH	52422.00171	2257.01546	23.22625	1.74841E-4	0.99822
	dS	-126.85265	6.71423	-18.89311	3.23741E-4	0.99822

Reduced Chi-sqr = 5.83389991989
COD(R^2) = 0.99151756294341
Iterations Performed = 47
Total Iterations in Session = 47
Fit converged. Chi-Sqr tolerance value of 1E-9 was reached.
Standard Error was scaled with square root of reduced Chi-Sqr.
Constants : R=8.31446 kb=1.38065e-023 h=6.62607e-034

Statistics

	k
Number of Points	5
Degrees of Freedom	3
Reduced Chi-Sqr	5.8339
Residual Sum of Squares	17.5017
R-Square (COD)	0.99152
Adj. R-Square	0.98869
Fit Status	Succeeded(100)

Fit Status Code :
100 : Fit converged. Chi-Sqr tolerance value of 1E-9 was reached.

Summary

	dH		dS		Statistics	
	Value	Standard Error	Value	Standard Error	Reduced Chi-Sqr	Adj. R-Square
k	52422.00171	2257.01546	-126.85265	6.71423	5.8339	0.98869

Table S5: Overview of relevant results from non-linear curve fitting of the Eyring data in Origin 2019b.

$\Delta H^‡$	52.4	± 2.3	kJ mol^{-1}
**$\Delta H^‡$**	**12.5**	**± 0.5**	**kcal mol^{-1}**
$\Delta S^‡$	-126.9	± 6.7	J mol^{-1} K^{-1}
**$\Delta S^‡$**	**-30.3**	**± 1.6**	**cal mol^{-1} K^{-1}**
$\Delta G^‡$ (25°C)	90.2	± 3.0	kJ mol^{-1}
**$\Delta G^‡$ (25°C)**	**21.5**	**± 0.7**	**kcal mol^{-1}**

Table S6: Overview of most relevant data obtained from non-linear curve fitting in origin.

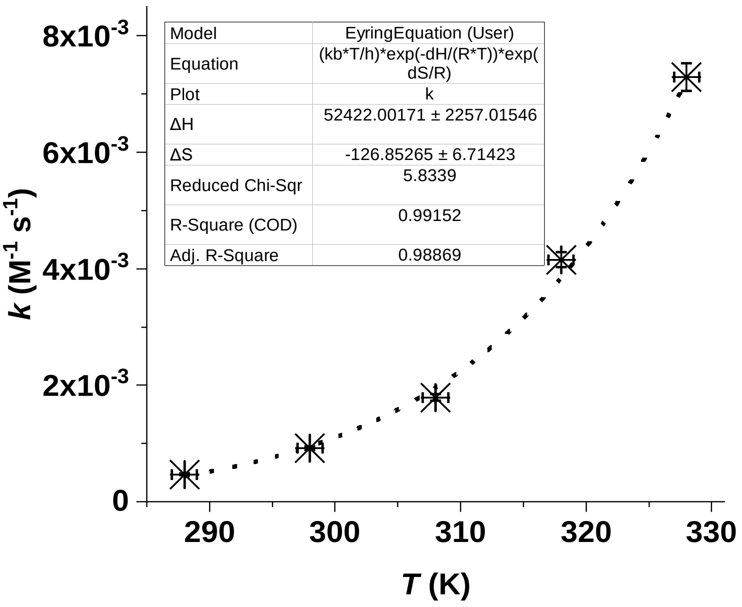

Figure S9: Eyring plot showing the non-linear fitting results of the reaction of **1a** in the presence of IDPi **2d** in $CDCl_3$.

5. Determination of the absolute configuration of products

The absolute configuration of product **3g, 3n** and **3o** was determined by X-ray crystallography after conversion into the corresponding osmate ester[17] and all other compounds were assigned by analogy.

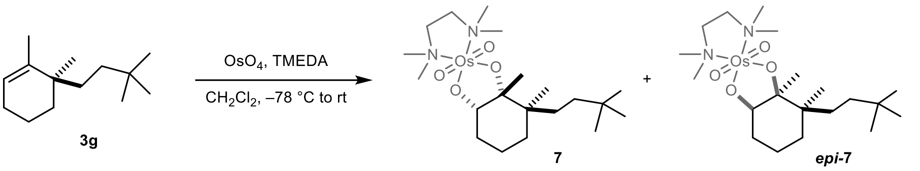

(*R*)-6-(3,3-dimethylbutyl)-1,6-dimethylcyclohex-1-ene osmate ester (7): *N,N,N',N'*-Tetramethylethylenediamine (TMEDA) (42.88 μL, 0.28 mmol, 1.1 equiv.) was added to a stirred solution of compound **3g** (50.53 mg, 0.26 mmol, 1.0 equiv.) in CH_2Cl_2 (5.0 mL) under argon. The resulting solution was cooled to –78 °C before a solution of osmium tetroxide (0.2M in CH_2Cl_2, 1.30 mL, 0.26 mmol, 1.0 equiv.) was added. The mixture was stirred for 30 min at –78 °C before it was allowed to reach rt. Following removal of the solvent under reduced pressure, a brown solid residue was directly purified by flash column chromatography on silica (eluent: 2–3% MeOH in CH_2Cl_2). Upon evaporation of the solvent under reduced pressure, the corresponding inseparable diastereomeric mixture of osmate ester **7** and ***epi*-7** was obtained as a brown solid (104 mg, 71% yield, d.r. = 13:1). Single crystals suitable for X-ray diffraction analysis were obtained by diffusing *n*-pentane into a saturated solution of diastereomeric mixture of osmate ester in THF at rt.

^{1}H NMR (600 MHz, $CDCl_3$) δ 3.76 (dd, *J* = 10.2, 7.0 Hz, 1H), 3.10–3.06 (m, 2H), 3.06–3.02 (m, 2H), 2.79 (s, 3H), 2.78 (s, 3H), 2.78 (s, 3H), 2.77 (s, 3H), 2.04–1.92 (m, 1H), 1.65–1.58 (m, 1H), 1.54–1.45 (m, 2H), 1.43–1.32 (m, 3H), 1.23 (s, 3H), 1.22–1.18 (m, 1H), 1.18–1.07 (m, 2H), 0.99 (s, 3H), 0.90 (s, 9H).

^{13}C NMR (151 MHz, $CDCl_3$) δ 91.8, 89.3, 64.1, 64.0, 51.6, 51.5, 50.9, 50.7, 39.9, 37.6, 32.2, 30.8, 30.3, 29.7, 27.3, 20.7, 19.1, 18.1.

HRMS *m/z* (ESI): calculated for $C_{20}H_{42}N_2NaO_4Os$ $[M+Na]^+$: 589.2656; found: 589.2651.

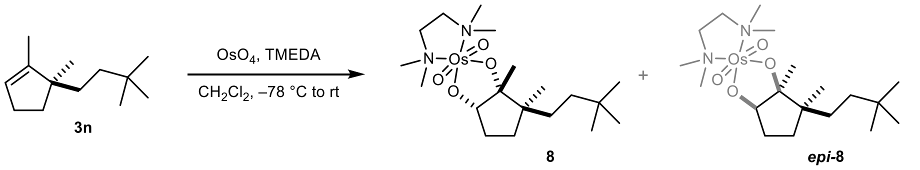

(*R*)-5-(3,3-dimethylbutyl)-1,5-dimethylcyclopent-1-ene osmate ester (8): The title compound was prepared according to the procedure of osmate ester (**7**) from compound **3n** (35.00 mg, 0.19 mmol, 1.0 equiv.). Purification by flash column chromatography on silica (eluent: 2–3% MeOH in CH_2Cl_2). Upon evaporation of the solvent under reduced pressure, the corresponding inseparable diastereomeric mixture of osmate ester **8** and ***epi*-8** was obtained as a brown solid (93 mg, 84% yield, d.r. = 3:1). Single

crystals suitable for X-ray diffraction analysis were obtained by liquid diffusion of saturated solution of diastereomeric mixture of osmate ester in $CDCl_3$ with *n*-pentane in NMR tube at rt.

^{1}H NMR (600 MHz, $CDCl_3$) δ 4.46 (dd, *J* = 9.3, 2.9 Hz, 1H), 3.07–2.97 (m, 2H), 2.95–2.84 (m, 2H), 2.74 (s, 3H), 2.70 (s, 3H), 2.68 (s, 3H), 2.67 (s, 3H), 1.98–1.87 (m, 1H), 1.69–1.60 (m, 2H), 1.46 (dddd, *J* = 13.9, 9.9, 2.9, 1.0 Hz, 1H), 1.24–1.17 (m, 2H), 1.16 (s, 3H), 1.18–1.10 (m, 1H), 0.98 (td, *J* = 13.0, 3.9 Hz, 1H), 0.88 (s, 3H), 0.76 (s, 9H).

^{13}C NMR (151 MHz, $CDCl_3$) δ 100.4, 98.7, 64.2, 64.0, 52.0, 51.4, 51.3, 50.6, 48.6, 38.5, 36.8, 30.4, 29.9, 29.6, 29.4, 24.6, 16.9.

HRMS *m/z* (ESI): calculated for $C_{19}H_{41}N_2O_4Os$ $[M+H]^+$: 553.2678; found: 553.2675.

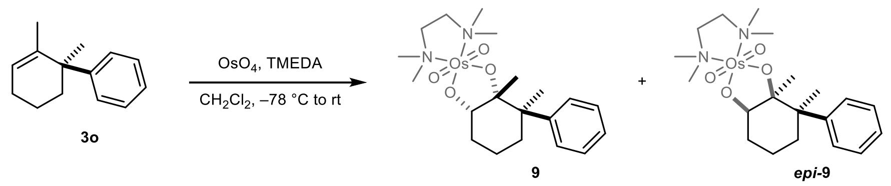

(*S*)-1,6-dimethyl-1,2,3,4-tetrahydro-1,1'-biphenyl osmate ester (9): The title compound was prepared according to the procedure of osmate ester (**7**) from compound **3o** (42.30 mg, 0.23 mmol, 1.0 equiv.). Purification by flash column chromatography on silica (eluent: 2–3% MeOH in CH_2Cl_2). Upon evaporation of the solvent under reduced pressure, the corresponding inseparable diastereomeric mixture of osmate ester **9** and ***epi*-9** was obtained as a brown solid (60 mg, 48% yield, d.r. = 32:1). Single crystals suitable for X-ray diffraction analysis were obtained by diffusing *n*-pentane into a saturated solution of diastereomeric mixture of osmate ester in CH_2Cl_2/Et_2O (1:4) at rt.

^{1}H NMR (600 MHz, $CDCl_3$) δ 7.64 (d, *J* = 7.7 Hz, 2H), 7.30–7.25 (m, 2H), 7.19–7.13 (m, 1H), 4.28 (dd, *J* = 4.6, 2.9 Hz, 1H), 3.30–3.15 (m, 2H), 3.07 (s, 3H), 3.01–2.91 (m, 2H), 2.90 (s, 3H), 2.82 (s, 3H), 2.76 (s, 3H), 2.43 – 2.37 (m, 1H), 2.15–2.04 (m, 1H), 2.02 (td, *J* = 12.6, 3.1 Hz, 1H), 1.91 (ddt, *J* = 14.6, 12.9, 4.8 Hz, 1H), 1.87–1.80 (m, 1H), 1.64–1.61 (m, 1H), 1.60 (s, 3H), 0.90 (s, 3H).

^{13}C NMR (151 MHz, $CDCl_3$) δ 151.2, 127.8, 127.3, 125.1, 98.1, 87.6, 64.4, 64.3, 53.3, 51.6, 50.9, 50.5, 47.8, 37.2, 31.6, 25.2, 24.0, 18.3.

HRMS *m/z* (ESI): calculated for $C_{20}H_{35}N_2O_4Os$ $[M+H]^+$: 559.2210; found: 559.2206.

6. Crystallographic data

X-ray crystal structure analysis of 3g•osmate ester derivate (7)

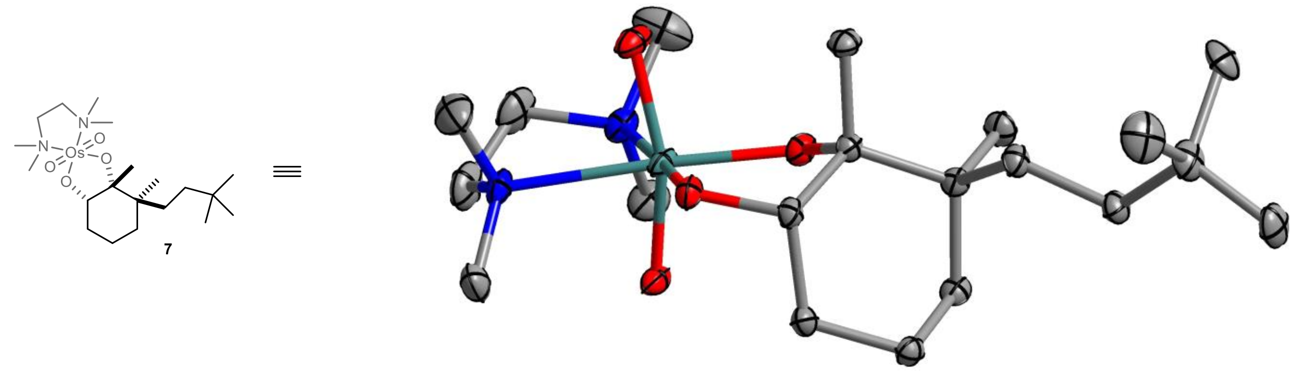

Figure S10: Structure of osmate ester **7** derived from product **3g**, H-atoms and the distorted carbon are omitted for clarity.

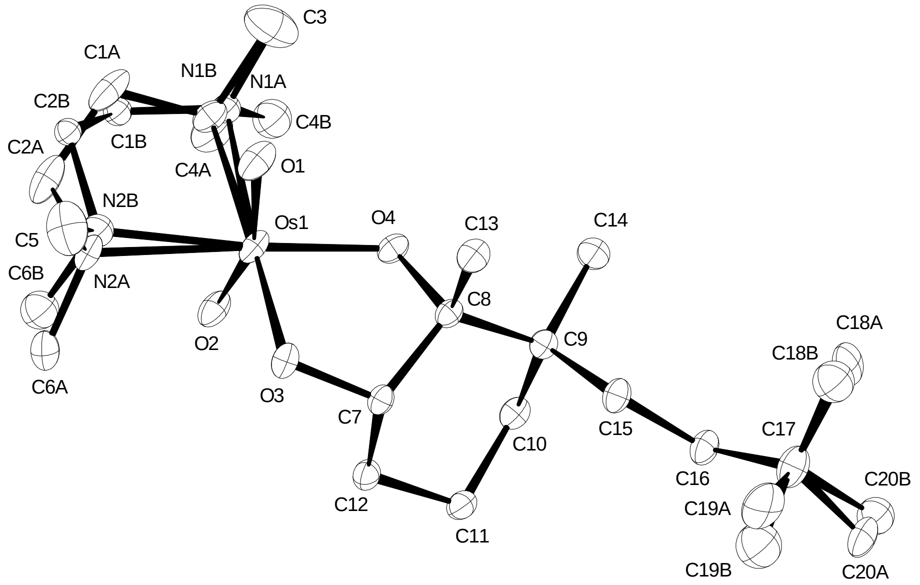

Figure S11: Structure of the osmate ester **7**. Contents of the orthorhombic unit cell with crystallographic numbering scheme. The thermal ellipsoid plot is shown at the 50% probability level, H-atoms omitted for clarity.

X-ray data for compound 7: $C_{20}H_{42}N_2O_4Os$, $M = 564.75$ g · mol^{-1}, yellow prism, crystal dimensions 0.081 x 0.061 x 0.052 mm, orthorhombic, $P2_12_12_1$ (no. 19), T = 100(2) K, a = 10.6851(8), b = 12.2273(9), c = 17.8362(13) Å , V = 2330.3(3) Å^3, Z = 4, ρ = 1.610 Mg · m^{-3}, $\mu(Mo\text{-}K_\alpha)$ = 5.497 mm^{-1}, λ = 0.71073 Å. Gaussian absorption correction (T_{min} = 0.75, T_{max} = 0.81), Bruker-AXS Kappa Mach3 APEX-II with 1\µS X-ray source, 2.019 < θ < 34.397°, 145366 measured reflections, 9746 independent reflections, 9548 reflections with $I > 2\sigma(I)$, R_{int} = 0.0286, absolute structure parameter = –0.0144(14), 298 parameters, residual electron density 1.0 (0.96 Å from Os1) / -1.1 (1.41 Å from N1B) e · Å^{-3}. The structure was solved by *SHELXT* and refined by full-matrix least-squares (*SHELXL*) against F^2 to R_1 = 0.013 [$I > 2\sigma(I)$], wR_2 = 0.027. **CCDC- 2248971.**

X-ray crystal structure analysis of 3n•osmate ester derivate (8)

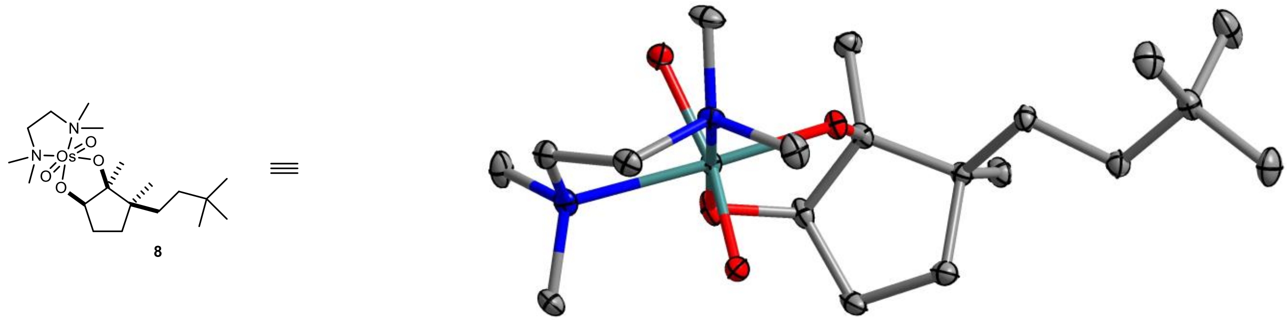

Figure S12: Structure of one of the two independent molecules of osmate ester **8** contained in the unit cell derived from product **3n**, H-atoms omitted for clarity.

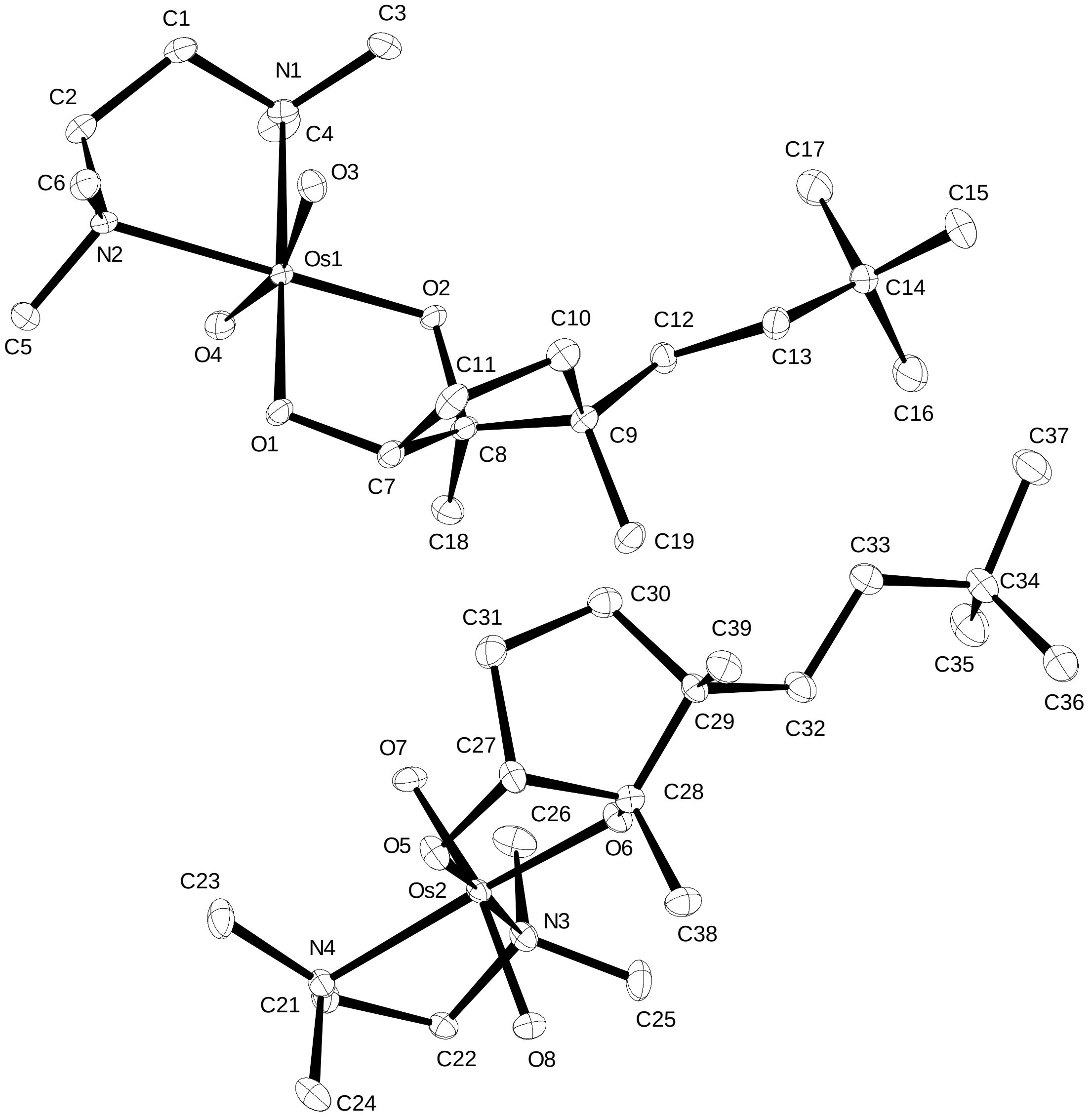

Figure 13: Structure of the osmate ester **8**. Contents of the monoclinic unit cell with crystallographic numbering scheme. The thermal ellipsoid plot is shown at the 50% probability level, H-atoms omitted for clarity.

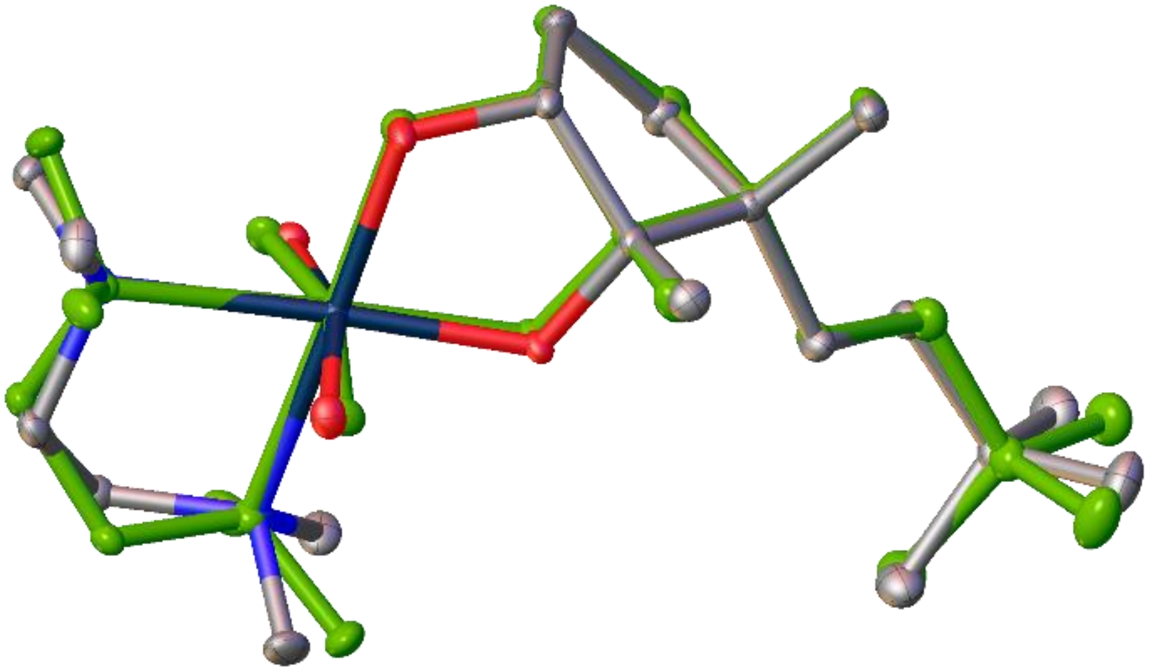

Figure S14: Overlay of the osmium coordination spheres of the two independent molecules of osmate ester **8** in the unit cell, proving that they differ only in conformational detail (molecule 1: C grey, N blue, O red, Os Prussian blue; molecule 2: entirely in green); H-atoms omitted for clarity.

X-ray data for compound 8: $C_{19}H_{40}N_2O_4Os$, *M* = 550.73 g · mol^{-1}, yellow plate, crystal size 0.18 x 0.074 x 0.031, monoclinic *P2₁* (no. 4), *T* = 100(2) K, *a* = 11.4632(15) Å, *b* = 11.0993(14) Å, *c* = 17.277(2) Å , β = 99.150(5)°, *V* = 2170.3(5) Å^3, *Z* = 4, ρ = 1.685 Mg · m^{-3}, $\mu(Mo\text{-}K_\alpha)$ = 5.900 mm^{-1}, λ = 0.71073 Å, Gaussian absorption correction (T_{min} = 0.51, T_{max} = 0.85), Bruker-AXS Kappa Mach3 APEX-II with 1\µS X-ray source, 1.194 < θ < 34.527°, 151761 measured reflections, 18304 independent reflections, 17523reflections with $I > 2\sigma(I)$, R_{int} = 0.0376, absolute structure parameter = –0.005(2), 487 parameters, residual electron density 1.4 (0.68 Å from Os1) / -1.6 (1.40 Å from Os1) e · Å^{-3}. The structure was solved by *SHELXT* and refined by full-matrix least-squares (*SHELXL*) against F^2 to R_1 = R_1 = 0.019 [$I > 2\sigma(I)$], wR_2 = 0.041. **CCDC- 2248972.**

X-ray crystal structure analysis of 3o•osmate ester derivate (9):

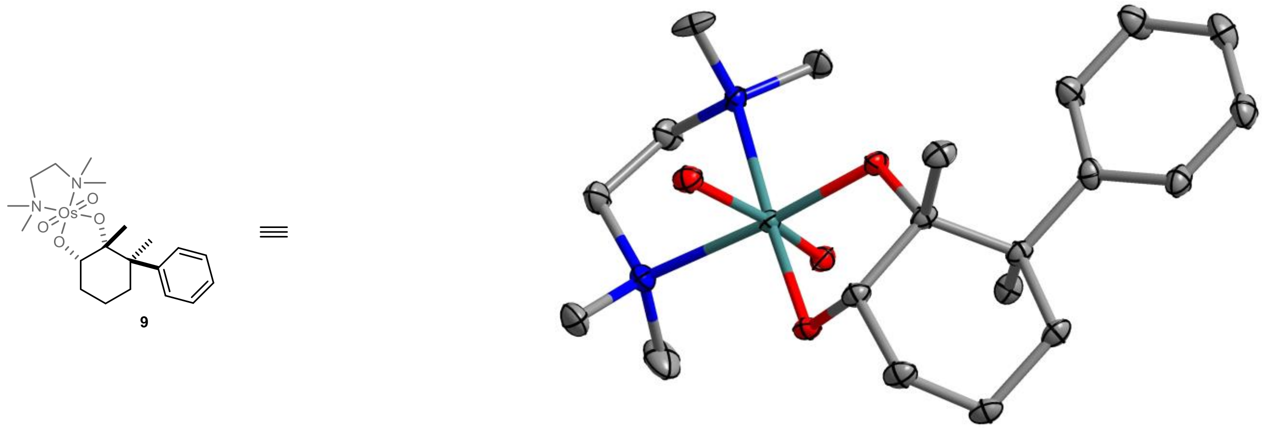

Figure S15: Structure of osmate ester **9** derived from product **3o**, H-atoms, Water molecule and the distorted carbon are omitted for clarity.

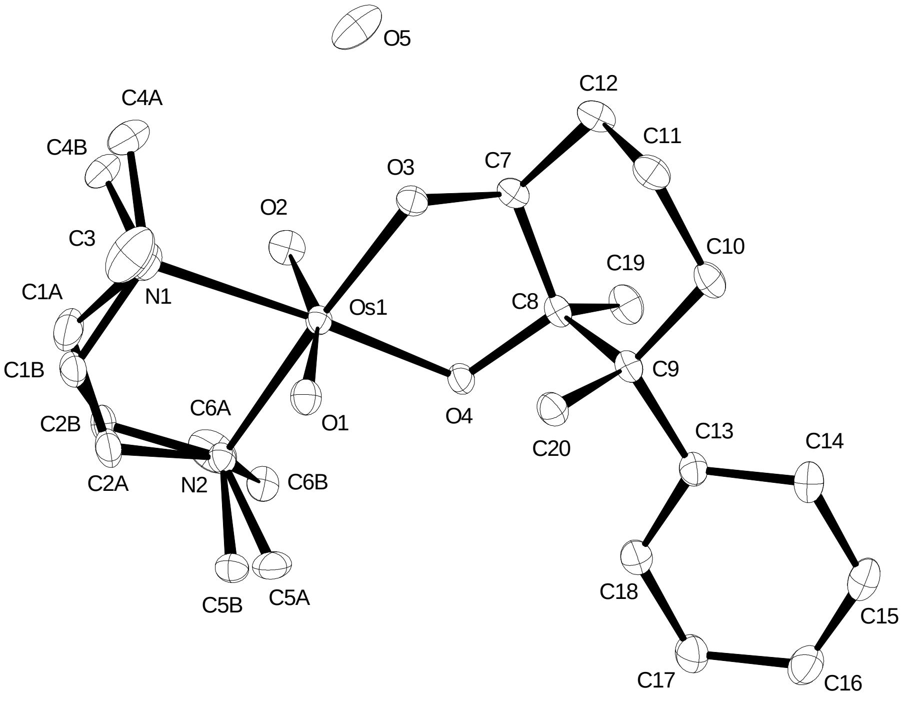

Figure S16: Structure of the osmate ester **9**. Contents of the monoclinic unit cell with crystallographic numbering scheme. The thermal ellipsoid plot is shown at the 50% probability level, H-atoms are omitted for clarity.

X-ray data for compound 9: $C_{20} H_{36} N_2 O_5 Os$, M = 574.71 g · mol^{-1}, yellow plate, crystal size 0.196 x 0.133 x 0.061, monoclinic $P2_1$ (no. 4), T = 100(2) K, a = 7.3868(4)Å, b = 12.2651(7) Å, c = 12.5132(7) Å , β = 105.582(2)°, V = 1092.03(11) Å^3, Z = 2, ρ = 1.748 Mg · m^{-3}, $\mu(Mo\text{-}K_\alpha)$ = 5.871 mm^{-1}, λ = 0.71073 Å, Gaussian absorption correction (T_{min} = 0.55, T_{max} = 0.74), Bruker-AXS Kappa Mach3 APEX-II with 1\μS X-ray source, 1.689 < θ < 33.745°, 41314 measured reflections, 8718 independent reflections, 8549 reflections with $I > 2\sigma(I)$, R_{int} = 0.0173, absolute structure parameter = –0.003(5), 310 parameters, residual electron density 1.6 (0.77 Å from Os1) / -0.9 (0.13 Å from Os1) e · Å^{-3}. The structure was solved by *SHELXT* and refined by full-matrix least-squares (*SHELXL*) against F^2 to R_1 = 0.015 [$I > 2\sigma(I)$], wR_2 = 0.038. **CCDC- 2248970.**

7. Computational studies

An initial, approximate structure for the catalyst was constructed from the crystal structure of a similar IDPi catalyst.[9] This was used as a starting point for conformer and stationary point searches.

Conformational searches were performed on all transition structures following the iMTD-GC procedure using the Conformer-Rotamer Ensemble Sampling Tool (CREST)[18]. Key bonds (breaking or forming) were constrained with a harmonic potential using GFN2-xTB[19]. Metadynamics duration was manually set to 100 ps. Conformers from the first cycle of iMTD-GC were filtered using an isostat with a 0.25 kcal/mol and 10 Å cutoff.[20] Remaining conformers were then optimized with DFT in Gaussian 16[21] unless specified otherwise: we used the B3LYP[22-24] functional, def2-SVP[25] basis set and Grimme's D3 correction[26] with Becke-Johnson damping[27] in a conductor-like polarizable continuum model, C-PCM(chloroform)[28,29]. The remaining conformers were then optimized followed by subsequent single point computations at M06-2X[30], ωB97X-D[31] (G16) and ωB97X-D4[32] (in ORCA)[33], all with the def2-TZVPP basis set, to correct the electronic energies. Calculation of high-level single points on the lower-level optimized geometries is a standard approach and an efficient way to increase the quality of computed energies[34]. A variety of density functional methods were surveyed to confirm that our conclusions were not sensitive to the method used. High-level single points at different levels of theory all favored the *S* TS, with ΔΔG's at M06-2X, ωB97xD, and ωB97x-D4 of 1.4, 2.5, and 3.0 kcal/mol respectively. All functionals include reasonable treatments of dispersion, a major contributor to non-covalent interactions, and have been shown to be reliable functionals in similar previous studies[35]. Thermochemistry analysis was set to a default temperature of 298.15 K. All three functionals include quantum mechanical dispersion, a major contributor to non-covalent interactions, and have been shown to be reliable functionals in similar previous studies[35]. These results revealed that the qualitative trends remained the same.

Conformational searches for the non-covalently bound ring expansion step were performed with a spherical log fermi potential around the system to prevent the substrate from dissociating from the complex in CREST (see below for more details).

Three-dimensional depictions of transition structures were generated using *CYLview*.[36] Noncovalent interaction (NCI)[37] plots were generated using *Multiwfn*[38].

All computed and reported structures in the main text can be found on ioChem-BD at the following DOI: [https://doi.org/10.19061/iochem-bd-6-262][39].

Additional Results:

Interpretation of Reaction Profile

The lowest energy ring expansion conformers (**C** and **G** in Fig. 4f) require the substrate to undergo a conformational change (from **B**) in the anionic pocket, the barrier of which is unclear. Therefore, we also considered the immediate ring expansion directly from the intermediate following the lowest-energy protonation transition structure (i.e., **B**, found by an intrinsic reaction coordinate (IRC) calculation). These ring expansion transition structures would assume that the protonated intermediate does not equilibrate and proceeds to ring expansion before being able to sample other conformer modes in the anionic pocket. These transition structures for *R* and *S* have free energy barriers of 16.9 and 12.1 respectively, still favoring *S* over *R*.

While some elementary steps in the proposed mechanism are uphill in energy, the overall process is predicted to be downhill. We are also assuming here that product release makes the reaction irreversible and thereby prevents equilibration, i.e., that the product ratio should be determined by the relative free energies of competing ring-expansion TSs, not products following them. This contention cannot be proven, since it is notoriously difficult to predict dissociation barriers for solution reactions, but it seems reasonable that dissociation of complex **F** would be faster here than reversion to **B** and conversion to **H** via $\mathbf{TS_{GH}}$ (a predicted overall barrier in the reverse direction of at least 9 kcal/mol; see above).

Hydrogen bonding interactions

To approximate the binding energies (BEs) of hydrogen bonds, electron densities (ρ) at bond critical points (BCPs) between hydrogen bonding atoms were computed at the (CPCM-$CHCl_3$)-B3LYP-D3(BJ)/def2-SVP level. Previous literature demonstrates that a correlation between hydrogen bond binding energy and electron density at BCPs ($BE = -223.08 * \rho + 0.7423$)[40]. Performing this analysis on the four hydrogen bonds with shortest contact distances results in a net total of -11.9 and -8.9 kcal/mol BEs for the *S* and *R* TS conformations, respectively. This result is consistent with the greater hydrogen bonding in the *S* TS relative to *R*.

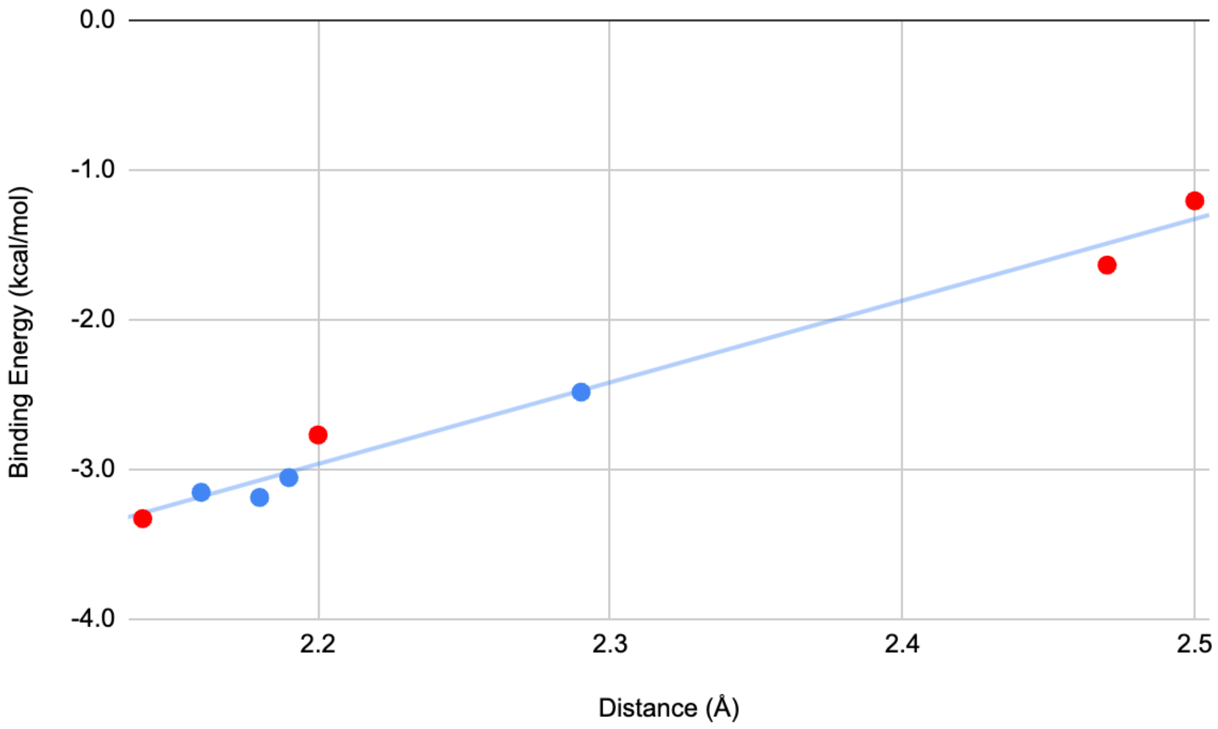

Figure S17: Binding energies computed from the electron density at bond critical points between 4 shortest hydrogen bonds as a function of H•••O distance. The binding energies were computed as (BE = -223.08 * ρ + 0.7423), where ρ is the electron density at the BCP. Blue points are from S TS, red points are from R TS. R^2=0.979.

tert-butyl effects on selectivity

A *tert*-butyl group on the IDPi catalyst appears to engage in weak van der Waals interactions with the alkyl chain of the substrate. However, this interaction is present in both *R* and *S* TSs. We argue that selectivity in part originates from the fact that the *tert*-butyl group prevents the *R* TS from adopting the conformation that maximizes hydrogen bonding that is observed in the *S* TS. For the *R* TS to maximize H-bonding, the alkyl chain of the substrate would have to be oriented directly toward the *tert*-butyl group of the IDPi catalyst, which would likely incur a significant (steric) energy penalty.

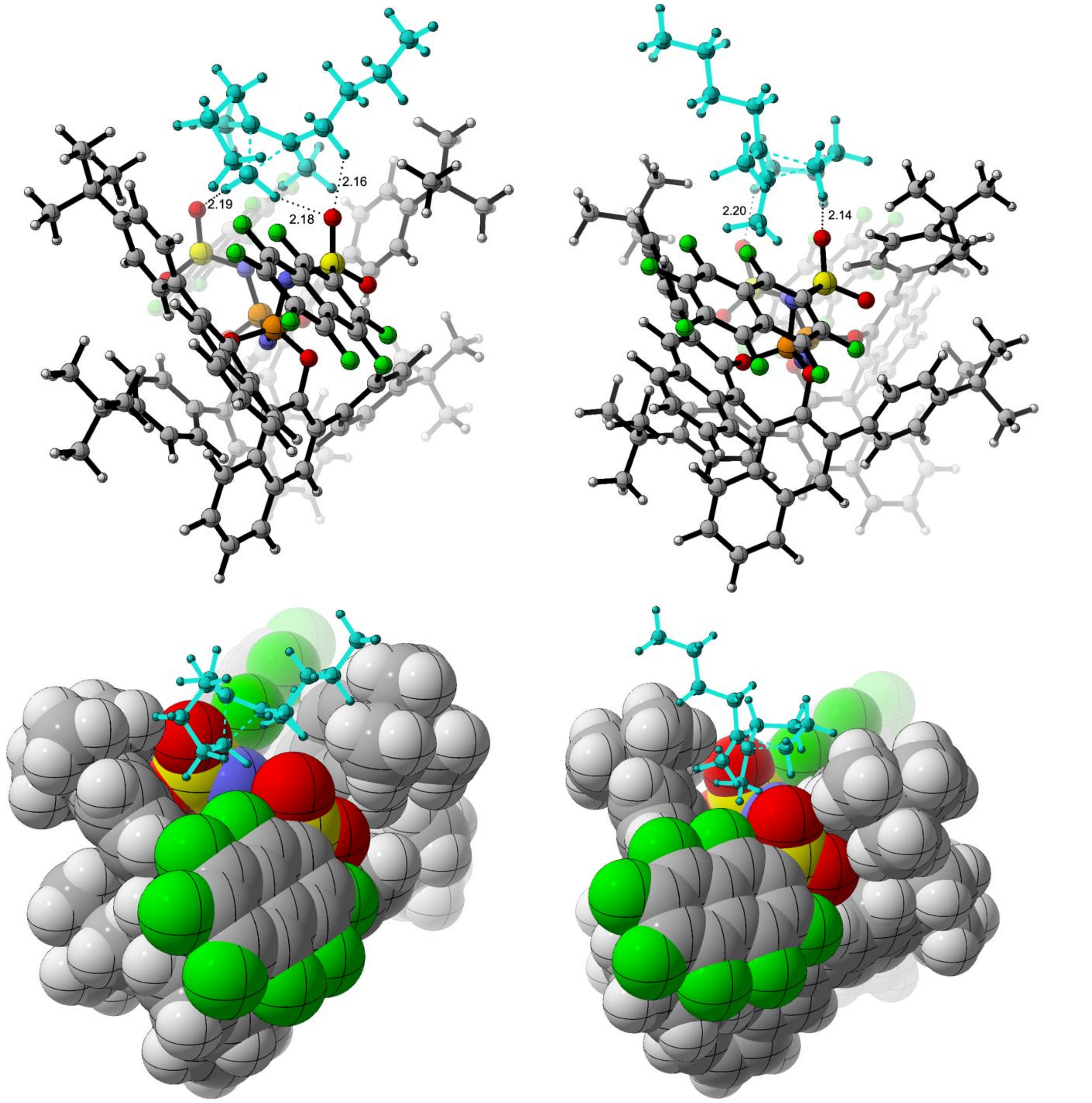

Figure S18: Structures of lowest-energy ring expansion conformers. Ball-and-stick (top) and space-filling models (bottom; substrate is shown in ball-and-stick for clarity). S conformer (left), R conformer (right).

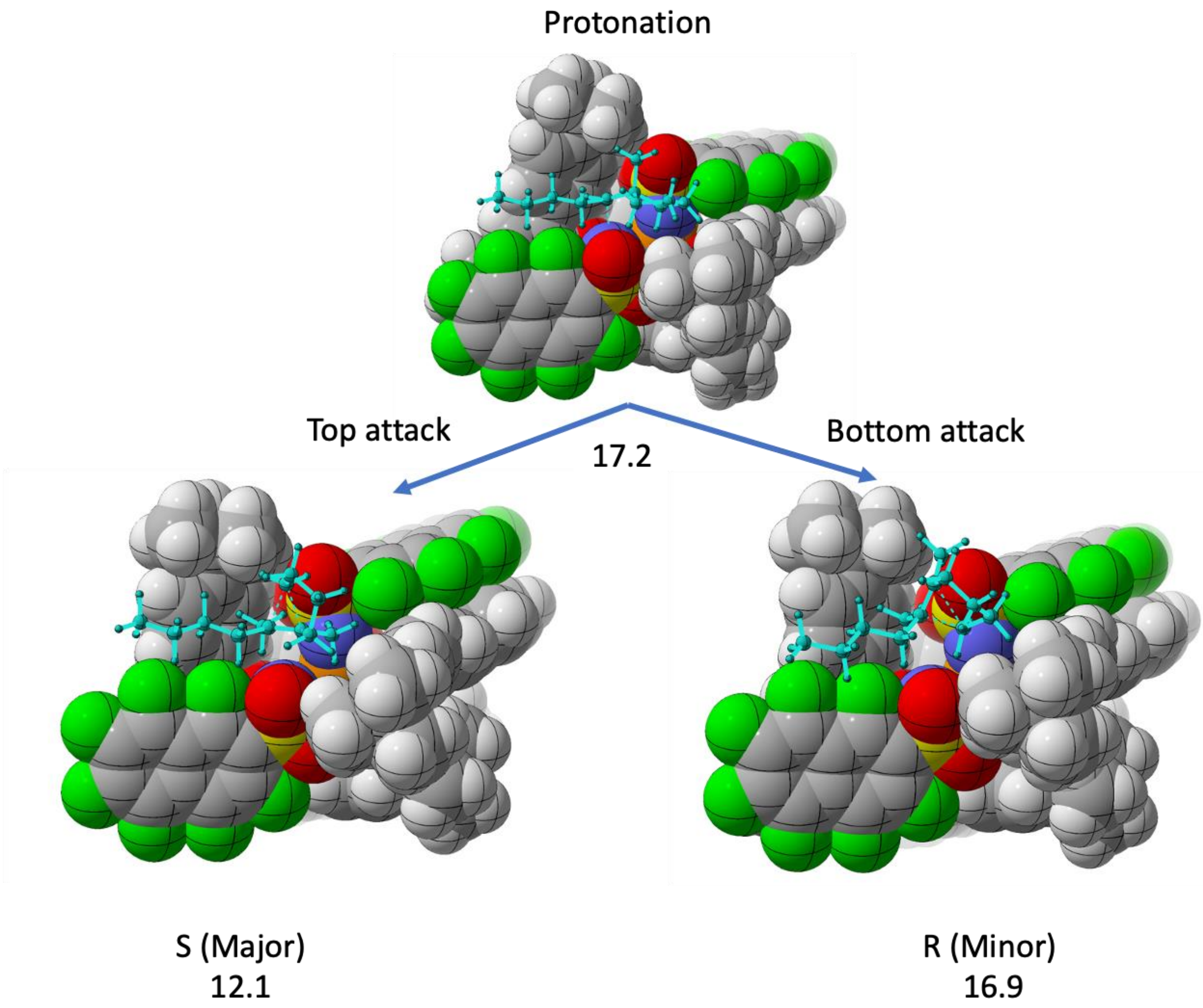

Figure S19: Ring expansion from **B** without significant conformational change. Shown are transition structures. Relative energies are in kcal mol^{-1}.

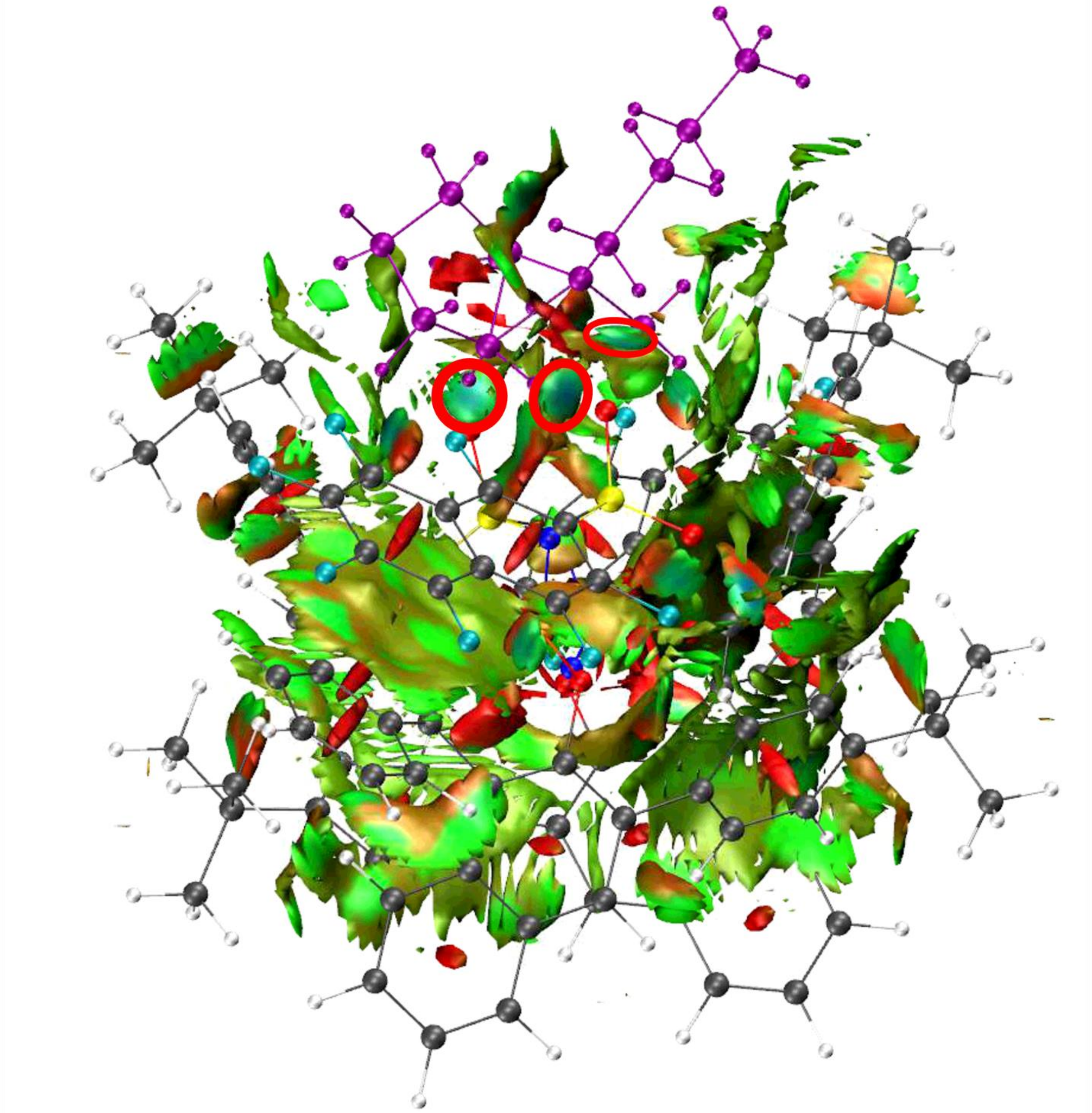

Figure S20: Non-Covalent Interaction plot of the lowest energy S ring expansion conformer. Interactions are colored as strongly attractive (blue), weakly attractive (green), and replusive (red). Key hydrogen bonding interactions between substrate and catalyst are circled in red. Substrate is colored purple for clarity.

Confining potential example syntax:

```
$wall
potential=logfermi
sphere: auto,all
$end
```

Constraints:
TS_AB:
C176, H84: 1.34Å
N40, H84: 1.38Å
TS_CD:

C171, C180: 1.91Å
C180, C179: 1.94Å

TS_GH:
C171, C181: 1.90Å
C171, C181: 1.90Å

TS_EF:
H218, O43: 1.35
H218, C212:1.31
S5, S41: 5.80

TS_IJ:
O43, H218: 1.30Å
C212 ,H218:1.38Å
N4, N40: 3.52Å

Energies of Structures

Table S7: Absolute Electronic (E) and Gibbs Free Energies (G) in Hartrees.

STRUCTURE	E B3LYP	E M06-2X	E WB97XD	E WB97X-D4	G B3LYP	G M062X	G WB97XD	G WB97X-D4
X-H	-7493.336	-7497.756	-7498.184	-7498.649	-7491.966	-7496.386	-7496.815	-7497.279
1C	-470.236	-470.456	-470.555	-470.578	-469.963	-470.183	-470.282	-470.305
A	-7963.609	-7968.225	-7968.766	-7969.245	-7961.940	-7966.556	-7967.097	-7967.576
TS_AB	-7963.594	-7968.205	-7968.746	-7969.222	-7961.928	-7966.538	-7967.080	-7967.556
B	-7963.603	-7968.216	-7968.759	-7969.237	-7961.934	-7966.547	-7967.090	-7967.568
C	-7963.590	-7968.211	-7968.752	-7969.233	-7961.924	-7966.544	-7967.086	-7967.567
TS_CD	-7963.589	-7968.213	-7968.752	-7969.233	-7961.921	-7966.544	-7967.084	-7967.564
D	-7963.598	-7968.213	-7968.756	-7969.235	-7961.928	-7966.543	-7967.086	-7967.566
E	-7963.612	-7968.224	-7968.765	-7969.243	-7961.941	-7966.554	-7967.094	-7967.572
TS_EF	-7963.605	-7968.219	-7968.757	-7969.234	-7961.939	-7966.553	-7967.092	-7967.569
F	-7963.612	-7968.228	-7968.765	-7969.243	-7961.943	-7966.559	-7967.096	-7967.574
G	-7963.588	-7968.209	-7968.748	-7969.229	-7961.919	-7966.540	-7967.080	-7967.560
TS_GH	-7963.588	-7968.211	-7968.749	-7969.229	-7961.919	-7966.542	-7967.080	-7967.560
H	-7963.602	-7968.218	-7968.758	-7969.239	-7961.933	-7966.549	-7967.089	-7967.570
I	-7963.607	-7968.221	-7968.765	-7969.243	-7961.939	-7966.552	-7967.096	-7967.575
TS_IJ	-7963.601	-7968.220	-7968.758	-7969.237	-7961.935	-7966.554	-7967.092	-7967.570
J	-7963.610	-7968.230	-7968.767	-7969.245	-7961.940	-7966.560	-7967.097	-7967.575
3C	-470.251	-470.469	-470.567	-470.590	-469.977	-470.195	-470.293	-470.316
TS_BDCONF	-7963.593	-7968.216	-7968.754	-7969.233	-7961.925	-7966.547	-7967.086	-7967.564
TS_BHCONF	-7963.586	-7968.206	-7968.745	-7969.225	-7961.917	-7966.538	-7967.077	-7967.557
TS_CD2	-7963.588	-7968.212	-7968.751	-7969.231	-7961.920	-7966.543	-7967.082	-7967.563

Intrinsic Reaction Coordinate Diagrams

TS$_{AB}$

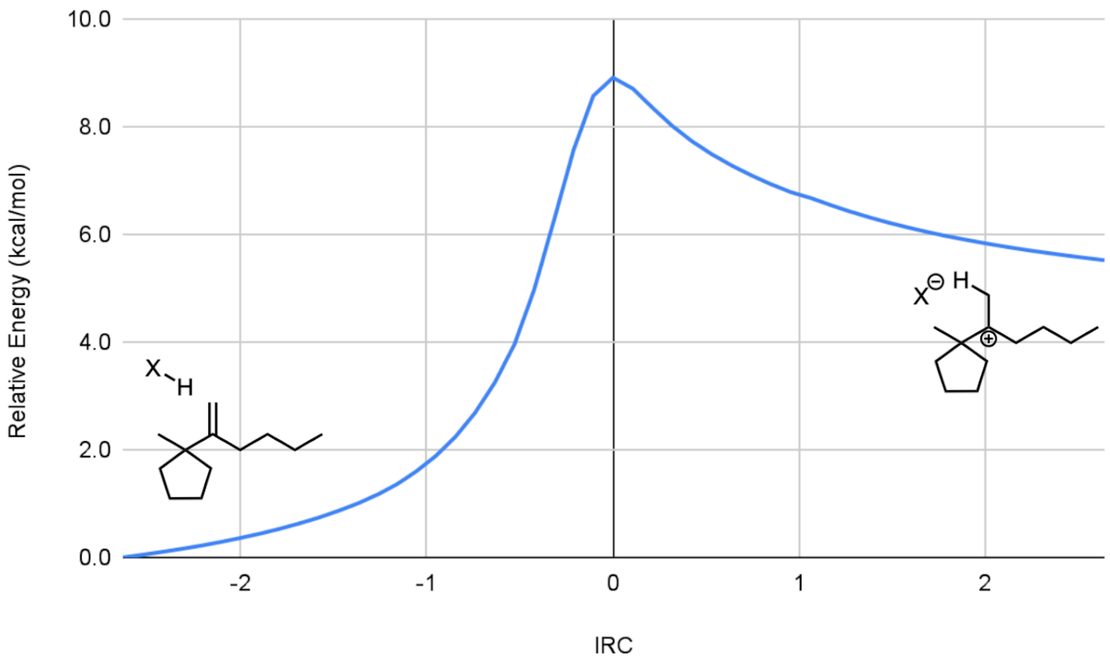

TS$_{CD}$

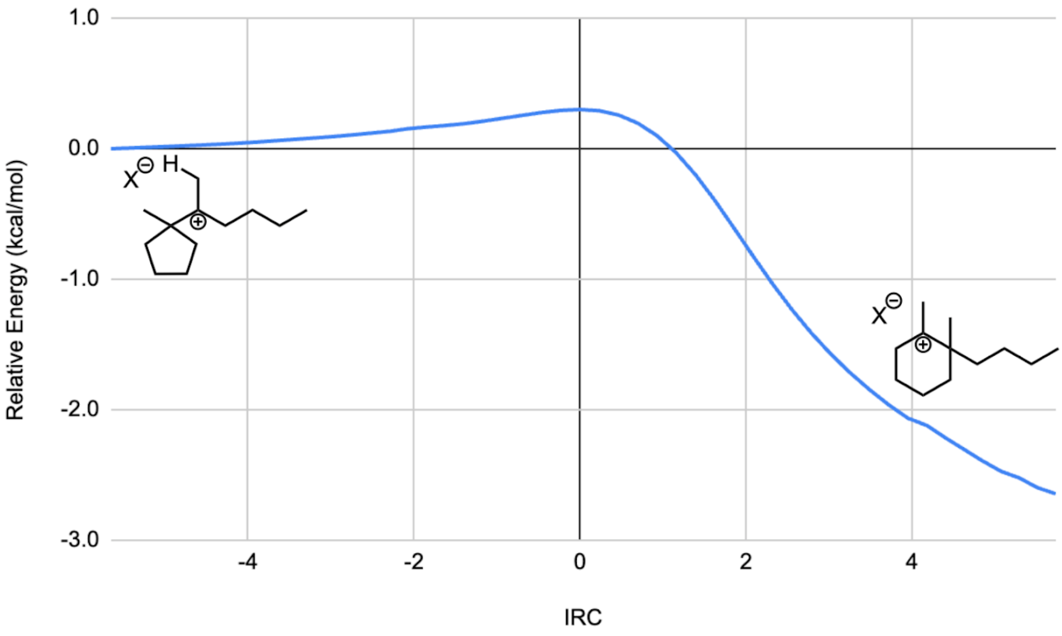

TS$_{GH}$

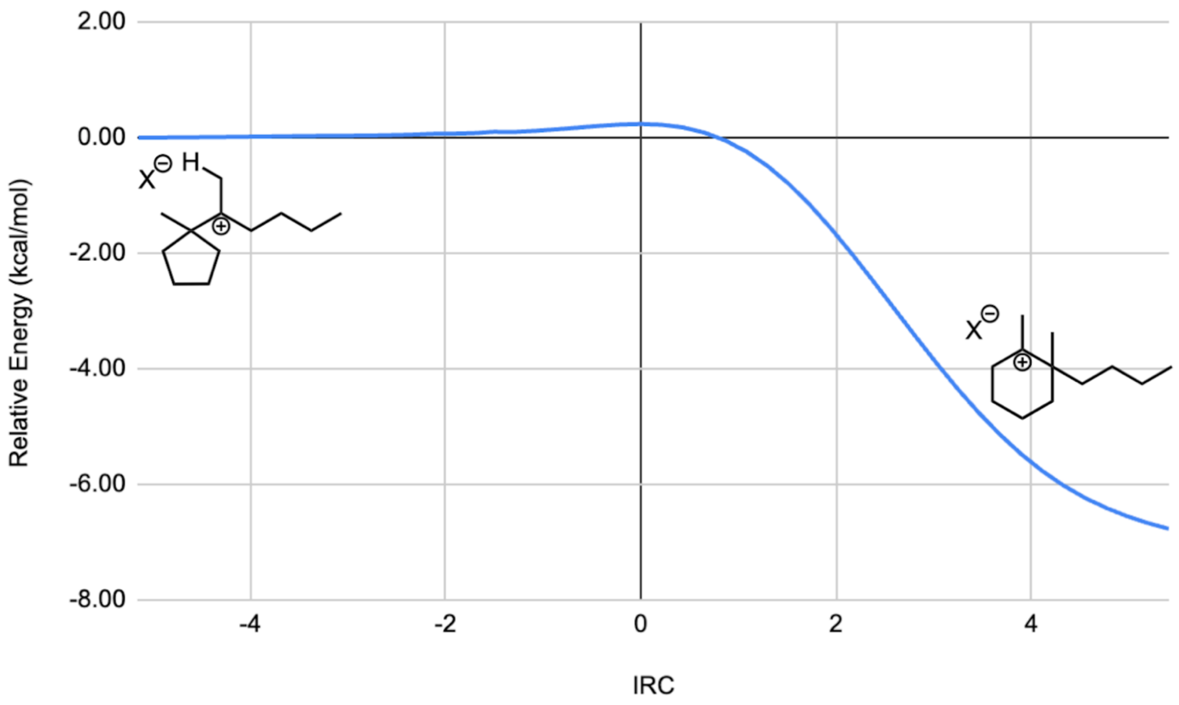
2.00
0.00
-2.00
-4.00
-6.00
-8.00
Relative Energy (kcal/mol)
-4
-2
0
2
4
IRC
X
H
X

TS$_{EF}$

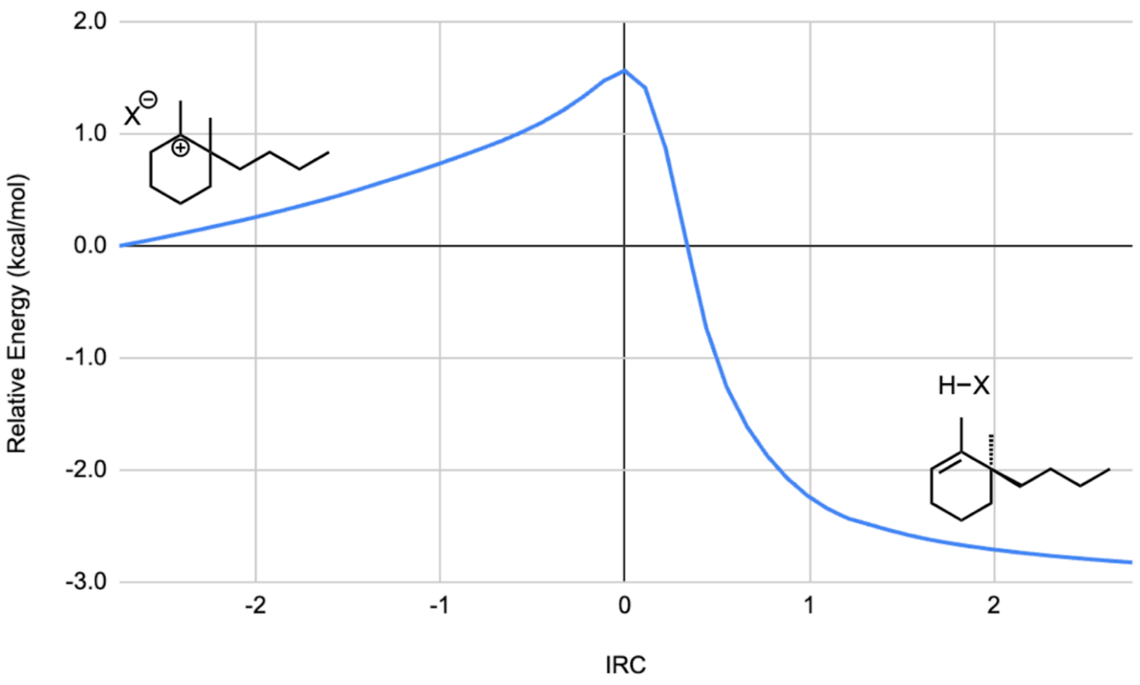
2.0
1.0
0.0
-1.0
-2.0
-3.0
Relative Energy (kcal/mol)
-2
-1
0
1
2
IRC
X
H−X

$\underline{TS_{IJ}}$

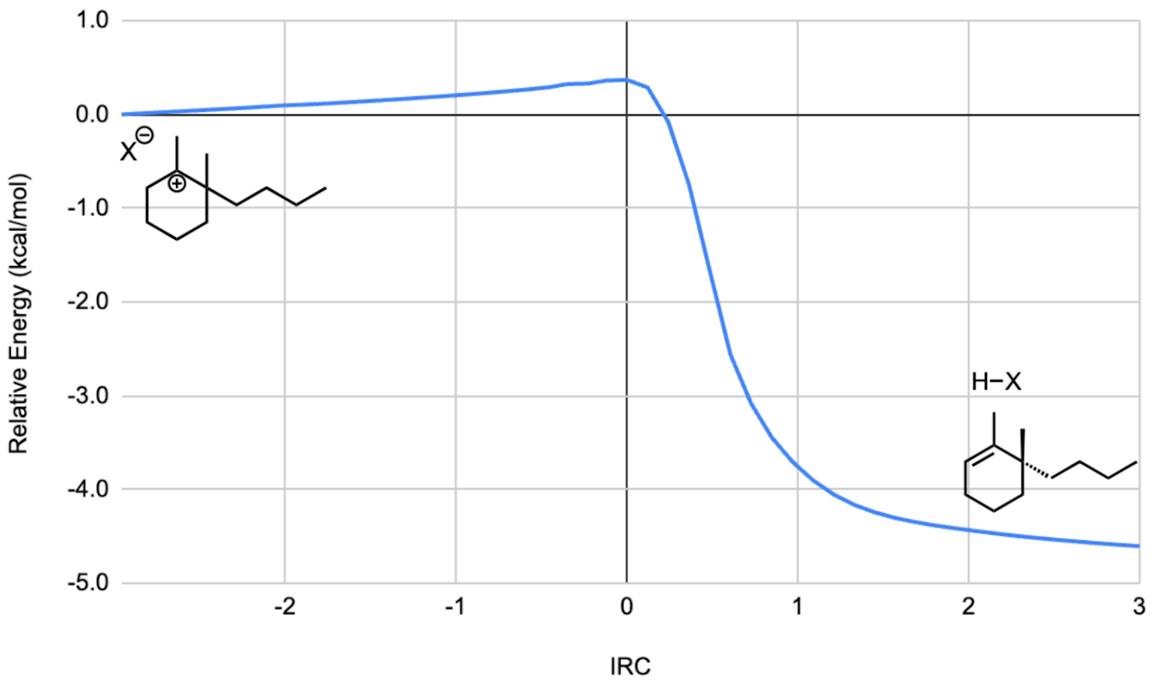

8. Computational details on %V_{Bur}

Method

Possible conformations were explored by the artificial force induced reaction (AFIR) method[41] implemented in the global route reaction mapping (GRRM) program[42]. An exhaustive conformational search has been performed using the artificial force induced reaction (AFIR) method[41], specifically, using SC-AFIR with constraints, implemented in the global route reaction mapping (GRRM) program[42] at GFN2-xTB level of theory[19]. Molecular geometries were optimized by ORCA program version 4.2.1 using B3LYP-D3(BJ)/def2-SVP level of theory[22,25,27]. All single point energy is calculated at B3LYP-D3-(BJ)-CPCM($CHCl_3$)/def2-TZVP level of theory[25]. The RIJCOSX approximation was used as well as the def2/J auxiliary basis set[43] for all atoms. Solvation effect has been accounted by using CPCM (Chloroform) solvation model, as implemented in ORCA. The molecular structures were visualized with the CYLview program.

Results and discussion

The main purpose of this section is to provide an idea of the pocket size of the catalysts for a better discussion. To address this issue, we begin with the conformer sampling of the key ion pair with the simplified substrate (Figure S21A). The most stable 10 conformers were further optimized and evaluated at the above-described DFT level, and the most stable conformers of each ion pair complex were chosen for further visualization (Figure 21B). At this point, as iIDP catalyst **2b** exclusively underwent spontaneous deprotonation upon the geometry optimization at B3LYP-D3(BJ)/def2SVP level of theory, therefore the acidic C-H bond (C165 and H170) length was fixed to 1.09 Å to simulate the corresponding ion pair structures.

For the consistent quantification of the pocket size, %V_{Bur} is used. To define the xyz axes, first the middle point of the substrate is determined by averaging the substrate cation xyz coordinate (Figure 21C). With the central nitrogen defined as the origin of the coordinate, one of the phosphorus atoms defines the x axis, the average point of the substrate defines the z axis, and the y axis is determined automatically. The center of the sphere is set on the z axis 5.0 Å away from the origin, and the sphere radius is set as 5.5 Å to cover the adjacent BINOL-substituents (Figure 21D). Based on the definition above, the %V_{Bur} is visualized by SambVca 2.1 program[44].

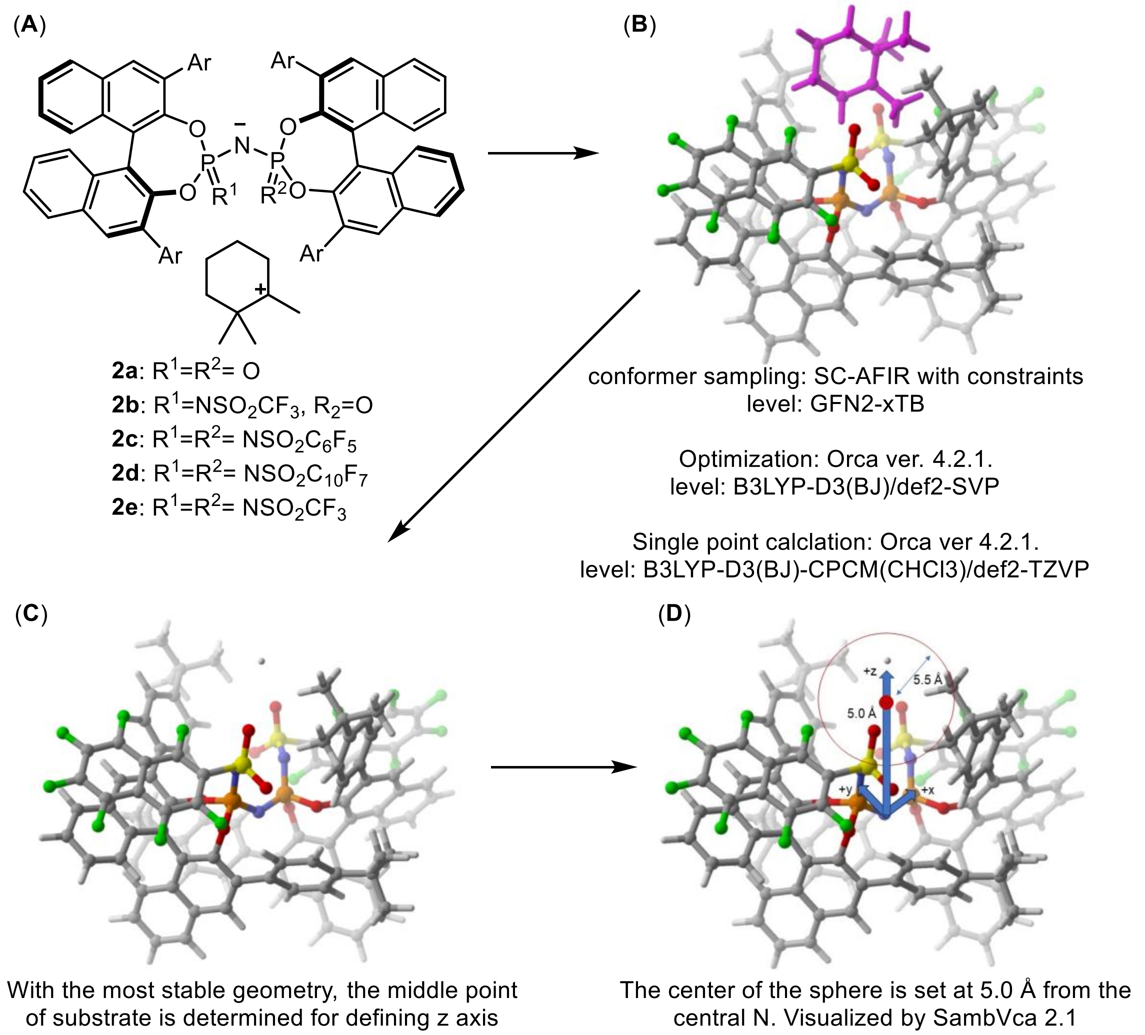

Figure S21: (**A**) Calculated complexes of the simplified substrate and a series of chiral counteranions. (**B**) Description of calculated level of theory in each step and the obtained structure with the example of **2d**. The dummy substrate is depicted in pink for clarity. (**C**) Visualization of the middle point of the dummy substrate with the example of **2d**. (**D**) Definition of sphere with the example of **2d**.

The xyz coordinates used for visualization

2a:

O	0.939134	2.585776	-2.288437
O	-0.751471	1.760075	-0.613668
O	1.724308	-2.131377	-0.369395
O	2.626308	-1.441365	-2.625542
P	1.204966	-1.435787	-1.765666
N	0.963611	0.091962	-1.464123
P	0.029368	1.245407	-1.972966
C	3.776793	2.143958	-2.086571
C	3.605414	2.322798	-3.468756
C	4.427978	1.667041	-4.388144
C	5.449155	0.794024	-3.977016
C	5.581257	0.583456	-2.593301
C	4.770314	1.240874	-1.670732
H	2.831960	3.000462	-3.832251
H	4.264537	1.861974	-5.448890
H	6.336370	-0.105933	-2.211903
H	4.904574	1.035662	-0.608035
C	6.429171	0.128608	-4.960653
C	6.498176	-1.390415	-4.712976
H	5.524663	-1.865675	-4.883155
H	7.226120	-1.853495	-5.397673
H	6.815965	-1.628862	-3.687075
C	6.025931	0.355600	-6.425908
H	6.730701	-0.164954	-7.091790
H	5.020547	-0.039700	-6.635068
H	6.046634	1.422820	-6.696006
C	7.835437	0.728877	-4.741598
H	7.842599	1.810682	-4.950657
H	8.173876	0.579778	-3.704530
H	8.568153	0.245048	-5.408096
C	-3.613294	1.756552	-0.728344
C	-3.281845	0.662591	0.093639
C	-4.120347	-0.445782	0.190145
C	-5.316987	-0.540983	-0.542122
C	-5.619245	0.531664	-1.396714
C	-4.790985	1.653771	-1.486978
H	-2.360149	0.679383	0.671235
H	-3.817559	-1.254779	0.857500
H	-6.521969	0.509226	-2.007073
H	-5.064643	2.462884	-2.168023
C	-6.267981	-1.736618	-0.359412
C	-5.495137	-3.063023	-0.216880
H	-4.878808	-3.268603	-1.105298
H	-4.832304	-3.071151	0.660180
H	-6.206402	-3.895764	-0.094786
C	-7.235187	-1.883605	-1.547810
H	-7.860443	-2.779919	-1.413367
H	-7.914768	-1.023994	-1.645036
H	-6.688063	-1.994266	-2.498511
C	-7.085908	-1.496910	0.930048

H	-6.424123	-1.449703	1.809099
H	-7.638836	-0.545939	0.874704
H	-7.814235	-2.308927	1.092358
C	2.851058	-2.716034	-5.199809
C	2.662598	-1.346285	-5.453404
C	2.080991	-0.904466	-6.645534
C	1.647024	-1.796164	-7.639360
C	1.852001	-3.165502	-7.387623
C	2.433182	-3.613412	-6.202289
H	2.980260	-0.610858	-4.717882
H	1.970281	0.171118	-6.785334
H	1.534591	-3.907813	-8.123636
H	2.538919	-4.687561	-6.036437
C	0.955875	-1.339490	-8.935208
C	-0.439036	-1.997743	-9.035429
H	-0.373265	-3.095282	-9.080085
H	-0.959396	-1.655004	-9.944789
H	-1.067032	-1.733926	-8.170103
C	1.802894	-1.764697	-10.152706
H	1.929767	-2.857190	-10.201086
H	1.317679	-1.437677	-11.087360
H	2.804272	-1.309431	-10.110624
C	0.774914	0.185665	-8.984975
H	1.741709	0.710725	-8.991523
H	0.233836	0.468562	-9.901432
H	0.190983	0.560227	-8.131285
C	1.570764	-0.129339	1.767982
C	1.579632	1.230012	2.128432
C	0.473069	1.835067	2.708901
C	-0.703809	1.116466	2.976654
C	-0.730726	-0.226575	2.566639
C	0.375965	-0.837729	1.965204
H	2.468440	1.830398	1.939365
H	0.543042	2.891928	2.965914
H	-1.622460	-0.832816	2.732064
H	0.315906	-1.885527	1.670416
C	-1.851717	1.807993	3.730704
C	-2.361783	3.024156	2.928930
H	-1.550016	3.727958	2.695388
H	-2.815959	2.710792	1.979544
H	-3.127568	3.569537	3.504747
C	-3.033266	0.860021	3.987930
H	-2.731551	-0.007165	4.595737
H	-3.824976	1.392741	4.538488
H	-3.474418	0.487427	3.051412
C	-1.313456	2.297174	5.096198
H	-2.118143	2.779595	5.676385
H	-0.915059	1.456726	5.687061
H	-0.502898	3.031217	4.971451
C	-1.380341	2.980756	-0.638574

C	1.606718	3.190357	-1.243819
C	2.896689	-1.676890	0.193766
C	3.397441	-2.571030	-2.703564
C	-2.805019	3.003445	-0.735429
C	3.515117	-3.226109	-3.970893
C	3.004770	2.936604	-1.094520
C	2.831603	-0.734092	1.265097
C	-1.169101	7.837436	-0.836048
C	-0.514213	6.626307	-0.752076
C	-1.238926	5.403772	-0.690105
C	-2.669782	5.453373	-0.777367
C	-3.314156	6.719375	-0.855727
C	-2.584589	7.888272	-0.874175
H	-0.591622	8.764129	-0.880241
H	0.575892	6.592975	-0.734789
C	-0.598284	4.127206	-0.568709
C	-3.414148	4.245618	-0.785120
H	-4.406369	6.746184	-0.909196
H	-4.504533	4.307143	-0.823481
C	2.882936	5.442477	3.108934
C	3.572483	4.724308	2.155684
C	2.925053	4.249967	0.979697
C	1.530399	4.525535	0.788662
C	0.847504	5.263820	1.796701
C	1.504537	5.711336	2.924999
H	4.695077	3.306234	0.166991
H	4.631075	4.488072	2.297850
C	3.626762	3.477612	0.017867
C	0.873004	3.979410	-0.365919
H	-0.216572	5.464398	1.681035
H	0.952510	6.266058	3.688008
C	7.768791	-1.501062	0.220580
C	6.607289	-1.991915	-0.339572
C	5.331277	-1.666768	0.202228
C	5.287760	-0.774663	1.323894
C	6.503454	-0.294173	1.886764
C	7.722151	-0.650409	1.352591
H	8.731747	-1.772795	-0.219420
H	6.666582	-2.634503	-1.216277
C	4.090136	-2.139942	-0.347857
C	4.036609	-0.337018	1.822426
H	6.445934	0.377186	2.747891
H	4.029660	0.352505	2.669136
C	6.326400	-6.613711	-1.838586
C	5.766148	-6.060949	-2.969091
C	4.964040	-4.887883	-2.889660
C	4.792660	-4.242977	-1.621701
C	5.346191	-4.863915	-0.467943
C	6.090724	-6.020450	-0.572815
H	4.519235	-4.820044	-4.999200
H	5.922670	-6.523422	-3.947528
H	5.165393	-4.412397	0.508197
H	6.504021	-6.485001	0.325659
C	4.333485	-4.342640	-4.034671
C	4.072312	-3.004449	-1.563873
H	-3.094537	8.852982	-0.934311
H	3.392988	5.797543	4.007781
H	8.646679	-0.267456	1.791476
H	6.933291	-7.519245	-1.912329
C	-1.800676	-1.676913	-5.022254
C	-1.332623	-0.378414	-5.420454
C	-2.351818	0.666890	-5.885706
C	-3.597516	0.644359	-5.005471
C	-4.161411	-0.771444	-4.923082
H	-0.414842	-0.430057	-6.019460
H	-0.984171	0.028441	-4.387570
H	-1.867762	1.654192	-5.857334
H	-2.622841	0.461065	-6.937671
H	-3.335513	1.015496	-4.003473
H	-4.361851	1.326588	-5.410908
H	-5.048843	-0.798348	-4.270963
H	-4.498821	-1.091584	-5.925879
C	-0.862100	-2.787978	-5.045920
H	-1.325647	-3.779971	-5.049374
H	-0.091351	-2.684998	-5.818124
H	-0.324488	-2.688820	-4.052030
C	-3.154242	-1.822012	-4.385878
C	-2.895993	-1.574583	-2.865478
H	-3.852704	-1.724656	-2.352624
H	-2.141594	-2.258757	-2.458835
H	-2.546250	-0.554916	-2.674459
C	-3.750927	-3.230447	-4.551874
H	-4.771151	-3.228999	-4.140684
H	-3.812395	-3.531325	-5.609862
H	-3.176654	-3.987329	-4.001021
O	-0.925418	1.095011	-3.128844
O	0.171117	-2.337720	-2.366005

2b:

O	1.002754	2.968188	-2.367480
O	-0.605625	1.819492	-0.792454
O	1.829806	-1.710893	-0.449779
O	2.710332	-1.380364	-2.786755
S	-0.681329	-3.064069	-1.493254
O	-0.903858	-2.673603	-0.096267
O	-1.868800	-3.510567	-2.254197
N	0.166206	-2.065725	-2.391218
P	1.368523	-1.113427	-1.892026
N	1.224238	0.432308	-1.886924
P	0.172264	1.564879	-2.223349
C	3.826538	2.450437	-2.107840

C	3.639107	2.566230	-3.494390
C	4.419891	1.831702	-4.392072
C	5.418806	0.946778	-3.954086
C	5.584100	0.819734	-2.563942
C	4.811222	1.548698	-1.665228
H	2.877929	3.243580	-3.881586
H	4.240544	1.976670	-5.458603
H	6.329387	0.133244	-2.160109
H	4.961668	1.391800	-0.596508
C	6.343821	0.171513	-4.909869
C	6.304723	-1.335586	-4.590209
H	5.305400	-1.754056	-4.760422
H	7.008125	-1.881762	-5.237858
H	6.583736	-1.542559	-3.547317
C	5.952840	0.363442	-6.382751
H	6.627189	-0.219596	-7.027957
H	4.928520	0.014283	-6.581762
H	6.031032	1.417510	-6.690848
C	7.791236	0.676002	-4.722410
H	7.872374	1.748877	-4.960235
H	8.133110	0.528059	-3.686627
H	8.478348	0.124517	-5.384350
C	-3.479840	1.609604	-0.540198
C	-4.802796	1.518852	-1.008102
C	-5.538189	0.334446	-0.899480
C	-4.985644	-0.825156	-0.332113
C	-3.656019	-0.737580	0.114351
C	-2.921570	0.441227	0.016929
H	-5.270469	2.383587	-1.483553
H	-6.556928	0.326228	-1.288172
H	-3.154476	-1.609549	0.537968
H	-1.901873	0.447934	0.389812
C	-5.753834	-2.147373	-0.191539
C	-5.012466	-3.266669	-0.954178
H	-4.982477	-3.057025	-2.034028
H	-3.971261	-3.387457	-0.625755
H	-5.531862	-4.228650	-0.808782
C	-7.183756	-2.046224	-0.745739
H	-7.694692	-3.015808	-0.637804
H	-7.780156	-1.293326	-0.207489
H	-7.189787	-1.787254	-1.816497
C	-5.839583	-2.522080	1.304265
H	-4.842226	-2.683855	1.739280
H	-6.338066	-1.726980	1.880840
H	-6.419040	-3.451445	1.432946
C	2.652593	-3.009249	-5.104103
C	2.681780	-1.693503	-5.595767
C	2.069840	-1.364140	-6.806393
C	1.375438	-2.315301	-7.570347
C	1.332198	-3.626178	-7.062019
C	1.951444	-3.966144	-5.859523
H	3.189600	-0.913199	-5.031217
H	2.141465	-0.331147	-7.146896
H	0.788339	-4.403101	-7.604197
H	1.865312	-4.986175	-5.477742

C	0.663121	-1.968531	-8.887392
C	-0.842440	-2.289564	-8.763764
H	-1.019987	-3.350857	-8.533177
H	-1.360869	-2.057463	-9.708064
H	-1.311669	-1.690500	-7.971393
C	1.265407	-2.805153	-10.035217
H	1.143237	-3.885571	-9.862269
H	0.768851	-2.558672	-10.988232
H	2.341979	-2.600135	-10.144963
C	0.807442	-0.481499	-9.248764
H	1.860323	-0.199795	-9.402203
H	0.266136	-0.269820	-10.184107
H	0.390734	0.176156	-8.471710
C	1.762445	0.113288	1.761876
C	1.729512	1.437852	2.230807
C	0.599938	1.953128	2.856150
C	-0.551252	1.172573	3.059888
C	-0.526759	-0.136714	2.556553
C	0.599059	-0.658893	1.914935
H	2.596609	2.082046	2.089990
H	0.625859	2.987298	3.195705
H	-1.393958	-0.787788	2.664122
H	0.556014	-1.678112	1.537356
C	-1.743353	1.759854	3.832109
C	-2.297375	2.992813	3.088454
H	-1.518427	3.751087	2.923778
H	-2.702618	2.710089	2.107607
H	-3.109446	3.460364	3.669309
C	-2.879879	0.738448	3.994829
H	-2.546952	-0.150091	4.553625
H	-3.713137	1.191601	4.555034
H	-3.272817	0.403370	3.022717
C	-1.259044	2.189536	5.236687
H	-2.095729	2.604201	5.823978
H	-0.838207	1.333156	5.788039
H	-0.477501	2.962407	5.175531
C	-1.318072	2.984624	-0.610738
C	1.647060	3.473966	-1.256072
C	3.038871	-1.351093	0.106395
C	3.397371	-2.573961	-2.712359
C	-2.746882	2.904984	-0.558985
C	3.373567	-3.408352	-3.869249
C	3.050422	3.246382	-1.120046
C	3.021035	-0.432186	1.196330
C	-1.447716	7.843867	-0.462161
C	-0.708579	6.679928	-0.487067
C	-1.344232	5.408379	-0.453267
C	-2.775959	5.360275	-0.434315
C	-3.508888	6.579379	-0.393973
C	-2.862729	7.796260	-0.402996
H	-0.937235	8.809617	-0.495464
H	0.379835	6.724960	-0.543795
C	-0.611466	4.177191	-0.470720
C	-3.432747	4.104952	-0.452575
H	-4.601183	6.528993	-0.364286

H	-4.521168	4.094051	-0.363565
C	2.872994	5.578518	3.181826
C	3.580467	4.944540	2.183501
C	2.932893	4.470047	1.007222
C	1.518036	4.658784	0.861672
C	0.817044	5.305195	1.919076
C	1.474317	5.755764	3.045769
H	4.731741	3.627908	0.150863
H	4.656586	4.777941	2.287882
C	3.657583	3.766979	0.010600
C	0.873895	4.136396	-0.312622
H	-0.261186	5.433623	1.843180
H	0.906910	6.240877	3.844222
C	7.899096	-1.419716	0.053800
C	6.705685	-1.879773	-0.463478
C	5.458537	-1.472918	0.087260
C	5.472862	-0.545969	1.182236
C	6.720891	-0.100027	1.701171
C	7.910853	-0.525834	1.153110
H	8.840356	-1.749735	-0.392830
H	6.713883	-2.560520	-1.313586
C	4.190788	-1.906424	-0.431964
C	4.250912	-0.065578	1.717341
H	6.713377	0.598582	2.542155
H	4.288604	0.616870	2.569143
C	6.003414	-6.704976	-1.405587
C	5.428024	-6.264484	-2.577163
C	4.748663	-5.015490	-2.634165
C	4.718632	-4.180619	-1.468544
C	5.289900	-4.682578	-0.266334
C	5.911670	-5.913538	-0.234208
H	4.147155	-5.227556	-4.701472
H	5.474490	-6.876437	-3.482084
H	5.220616	-4.083194	0.641422
H	6.337271	-6.284337	0.701453
C	4.087000	-4.592458	-3.814584
C	4.097250	-2.887412	-1.553279
H	-3.438252	8.724615	-0.376362
H	3.384567	5.935122	4.079263
H	8.859596	-0.167081	1.559774
H	6.514214	-7.670096	-1.371532
C	-2.033893	-1.349905	-5.237840
C	-1.123835	-0.277613	-5.595320
C	-1.721538	0.944140	-6.315270
C	-3.047445	1.369739	-5.691265
C	-4.002475	0.182447	-5.598718
H	-0.214682	-0.671960	-6.064298
H	-0.806742	0.099500	-4.623035
H	-0.987066	1.759926	-6.260022
H	-1.862104	0.693453	-7.382451
H	-2.847973	1.788313	-4.693846
H	-3.507976	2.170148	-6.292489
H	-4.941571	0.474400	-5.101770
H	-4.277315	-0.158913	-6.613262
C	-1.535120	-2.718352	-5.153883
H	-2.191832	-3.393677	-5.727070
H	-0.494281	-2.822225	-5.470686
H	-1.640950	-3.067811	-4.101851
C	-3.422639	-1.026710	-4.819861
C	-3.216784	-0.632597	-3.311001
H	-4.207638	-0.345531	-2.937587
H	-2.857618	-1.487350	-2.729646
H	-2.527617	0.206877	-3.178876
C	-4.390079	-2.215759	-4.869513
H	-5.366918	-1.892981	-4.479438
H	-4.539666	-2.578530	-5.899631
H	-4.046427	-3.049972	-4.244091
C	0.353711	-4.633688	-1.404994
F	0.729808	-5.001826	-2.634193
F	-0.372545	-5.608308	-0.866470
F	1.442979	-4.452695	-0.665667
O	-0.754145	1.469178	-3.395561

2c:

O	1.244053	2.998029	-2.307060
O	-0.461404	2.102774	-0.703601
O	1.761532	-1.817811	-0.252015
O	2.742595	-1.298086	-2.511681
S	-0.776324	-2.935158	-1.612886
S	-0.331607	2.049260	-4.726187
O	-1.297585	-2.587556	-0.292462
O	-1.779996	-3.337587	-2.641462
O	1.074099	2.118634	-5.142060
O	-1.276781	1.435512	-5.675982
N	0.158722	-1.844245	-2.276015
N	-0.555993	1.391094	-3.277543
P	1.389873	-1.049920	-1.641011
N	1.312881	0.498225	-1.459496
P	0.388024	1.640768	-2.015978
C	4.011819	2.428493	-1.876855
C	3.875461	2.456197	-3.273708
C	4.673007	1.647911	-4.086127
C	5.633863	0.775752	-3.551408
C	5.728184	0.716722	-2.148648
C	4.939009	1.521068	-1.331018
H	3.145861	3.111722	-3.746695
H	4.526728	1.720470	-5.163751
H	6.431480	0.030213	-1.673788
H	5.031866	1.424651	-0.247062
C	6.574764	-0.069819	-4.426219
C	6.370499	-1.567297	-4.130193
H	5.354152	-1.886122	-4.392919

H	7.072559	-2.174969	-4.722371
H	6.538259	-1.801711	-3.069281
C	6.333291	0.164246	-5.924740
H	7.023734	-0.455340	-6.517092
H	5.310172	-0.111315	-6.222675
H	6.506826	1.214806	-6.206059
C	8.037545	0.305892	-4.103781
H	8.225615	1.373607	-4.300716
H	8.283257	0.101797	-3.050000
H	8.728918	-0.283572	-4.726861
C	-3.298154	1.950872	-0.676565
C	-2.814779	0.859671	0.071953
C	-3.546807	-0.317920	0.186582
C	-4.798230	-0.476634	-0.431051
C	-5.270337	0.605978	-1.193624
C	-4.536307	1.789375	-1.320328
H	-1.856749	0.923006	0.580487
H	-3.110933	-1.131612	0.764261
H	-6.229030	0.537196	-1.708771
H	-4.921753	2.595661	-1.947871
C	-5.622282	-1.752670	-0.193900
C	-4.716803	-3.000402	-0.144309
H	-4.114584	-3.107663	-1.058790
H	-4.010442	-2.972206	0.697623
H	-5.336485	-3.903672	-0.022849
C	-6.685600	-1.960890	-1.286144
H	-7.219178	-2.909206	-1.116743
H	-7.438969	-1.158911	-1.289814
H	-6.233910	-2.004650	-2.289443
C	-6.342682	-1.600018	1.165227
H	-5.615816	-1.482768	1.983715
H	-6.998722	-0.714970	1.167364
H	-6.962966	-2.486508	1.379364
C	2.565134	-2.617061	-5.056527
C	2.593236	-1.233978	-5.309384
C	2.003765	-0.695784	-6.455679
C	1.373819	-1.504871	-7.411178
C	1.362357	-2.890850	-7.163427
C	1.930268	-3.433564	-6.013229
H	3.073248	-0.553725	-4.609402
H	2.020250	0.386804	-6.565698
H	0.868978	-3.565427	-7.867706
H	1.843404	-4.506017	-5.828182
C	0.692865	-0.928267	-8.661892
C	-0.804248	-1.306864	-8.653512
H	-0.953183	-2.398086	-8.632650
H	-1.298673	-0.919019	-9.558715
H	-1.311093	-0.868558	-7.781871
C	1.355153	-1.515651	-9.926479
H	1.259590	-2.612473	-9.968899
H	0.880843	-1.099962	-10.831068
H	2.428027	-1.267094	-9.959354
C	0.796761	0.604253	-8.717404
H	1.843998	0.936960	-8.787955
H	0.270015	0.978930	-9.608966

H	0.341657	1.079780	-7.837050
C	1.929069	0.099288	1.875198
C	2.068896	1.471118	2.147423
C	1.031969	2.205898	2.708900
C	-0.193389	1.604862	3.046294
C	-0.346122	0.246387	2.724641
C	0.684540	-0.494521	2.137902
H	3.002083	1.976245	1.896973
H	1.194861	3.267240	2.895311
H	-1.282074	-0.269441	2.941207
H	0.514864	-1.544917	1.901100
C	-1.284872	2.432870	3.742078
C	-1.890513	3.437838	2.741962
H	-1.112394	4.051865	2.269522
H	-2.438581	2.919946	1.944241
H	-2.596181	4.112103	3.256194
C	-2.414227	1.548518	4.289216
H	-2.033821	0.798366	5.001134
H	-3.152000	2.173243	4.817112
H	-2.946616	1.021997	3.483667
C	-0.665850	3.210388	4.923367
H	-1.451339	3.762653	5.465941
H	-0.175115	2.524746	5.632197
H	0.082596	3.943828	4.587743
C	-1.143800	3.301058	-0.713253
C	1.857408	3.617902	-1.229344
C	3.015099	-1.588037	0.299735
C	3.288592	-2.561796	-2.606014
C	-2.569449	3.245317	-0.730521
C	3.205197	-3.232165	-3.864734
C	3.233194	3.329330	-0.990821
C	3.104503	-0.647565	1.364557
C	-1.170465	8.147755	-0.961367
C	-0.455250	6.969851	-0.907028
C	-1.118572	5.720082	-0.768106
C	-2.552252	5.698995	-0.758161
C	-3.257676	6.933760	-0.797820
C	-2.584758	8.132653	-0.882050
H	-0.642574	9.097296	-1.076989
H	0.634013	6.984686	-0.972749
C	-0.409347	4.480435	-0.658499
C	-3.238666	4.457757	-0.719804
H	-4.350300	6.909396	-0.777575
H	-4.329674	4.467411	-0.679654
C	2.911509	5.979954	3.109387
C	3.638810	5.220917	2.218328
C	3.046700	4.715490	1.026701
C	1.665071	4.995973	0.755501
C	0.943244	5.777259	1.700611
C	1.549933	6.260245	2.842774
H	4.845926	3.712726	0.363977
H	4.684370	4.974430	2.423196
C	3.794349	3.900164	0.137850
C	1.064105	4.409475	-0.413733
H	-0.108044	5.993676	1.524182

H	0.968441	6.852241	3.553701
C	7.849831	-2.084654	0.154707
C	6.611112	-2.438328	-0.339990
C	5.414702	-1.922395	0.230849
C	5.531315	-1.002679	1.325933
C	6.822819	-0.665330	1.819320
C	7.960602	-1.191594	1.248734
H	8.750324	-2.495491	-0.307828
H	6.546060	-3.111947	-1.191559
C	4.103392	-2.227366	-0.275726
C	4.369399	-0.399858	1.869582
H	6.892976	0.034729	2.656061
H	4.484234	0.297270	2.702589
C	5.466651	-7.044304	-1.800644
C	4.945611	-6.416468	-2.909347
C	4.389635	-5.110257	-2.815371
C	4.417168	-4.430051	-1.553366
C	4.922793	-5.123083	-0.419094
C	5.431002	-6.399254	-0.539941
H	3.818003	-5.010204	-4.894874
H	4.938344	-6.917016	-3.880541
H	4.897033	-4.634204	0.555204
H	5.806108	-6.918934	0.344289
C	3.797724	-4.482957	-3.939723
C	3.918058	-3.088164	-1.483539
H	-3.140756	9.072182	-0.919938
H	3.378809	6.359140	4.021266
H	8.944488	-0.911746	1.633296
H	5.885061	-8.049368	-1.884828
C	-2.388066	-2.070321	-4.414652
C	-3.040103	-1.020096	-3.597068
C	-3.924784	-0.184860	-4.576389
C	-4.846411	-1.075092	-5.410781
C	-4.064575	-2.202875	-6.080664
H	-2.296227	-0.358762	-3.132069
H	-3.670845	-1.458554	-2.816776
H	-4.492221	0.530508	-3.965102
H	-3.259941	0.404405	-5.224807
H	-5.648672	-1.497281	-4.783741
H	-5.345898	-0.469051	-6.185133
H	-4.724767	-2.887341	-6.639403
H	-3.349998	-1.783353	-6.808391
C	-1.085972	-1.755636	-4.989604
H	-0.732545	-2.441979	-5.756524
H	-1.106858	-0.712760	-5.353863
H	-0.353152	-1.710920	-4.161905
C	-3.270127	-3.107689	-5.048047
C	-4.257214	-3.788901	-4.086051
H	-5.007123	-4.342557	-4.671914
H	-3.717173	-4.492672	-3.440729
H	-4.790949	-3.086589	-3.436962
C	-2.502297	-4.192885	-5.814907
H	-3.216733	-4.915790	-6.237247
H	-1.917144	-3.782795	-6.649676
H	-1.821536	-4.730223	-5.139531
C	-0.934433	3.750175	-4.489953
C	-0.126835	4.888550	-4.602648
C	-2.281620	3.922445	-4.147609
C	-0.659760	6.161873	-4.369555
C	-2.819434	5.186192	-3.922050
C	-2.007840	6.313869	-4.050546
C	0.274946	-4.396194	-1.396028
C	0.789740	-4.767754	-0.145816
C	0.625682	-5.162540	-2.514803
C	1.599108	-5.900467	-0.022104
C	1.407222	-6.308803	-2.391501
C	1.876774	-6.692248	-1.134776
F	1.157716	4.827172	-4.921271
F	0.112230	7.240811	-4.463214
F	-2.504345	7.526286	-3.850777
F	-4.098567	5.308483	-3.572996
F	-3.093361	2.879880	-4.000879
F	0.560895	-4.076745	0.959567
F	2.107144	-6.227969	1.160372
F	2.607134	-7.787948	-0.998658
F	1.723024	-7.020251	-3.469886
F	0.238159	-4.812943	-3.739416

2d:

O	1.182612	3.012714	-2.409323
O	-0.543031	2.049883	-0.854243
O	1.921651	-1.647333	-0.117266
O	2.804555	-1.296246	-2.440575
S	-0.673512	-2.809964	-1.137438
S	-0.260890	1.797298	-4.870139
O	-0.975455	-2.279183	0.197631
O	-1.820239	-3.267059	-1.941902
O	1.140327	1.899538	-5.292222
O	-1.177022	1.064956	-5.764445
N	0.221981	-1.846748	-2.052768
N	-0.462942	1.234474	-3.384210
P	1.479478	-0.998177	-1.542602
N	1.398600	0.564017	-1.481520
P	0.407794	1.611440	-2.104251
C	3.983644	2.488987	-1.971292
C	3.847857	2.480903	-3.367936
C	4.666955	1.674135	-4.161809
C	5.651206	0.840385	-3.608279
C	5.752071	0.822915	-2.204891
C	4.940883	1.624093	-1.406818
H	3.103066	3.106965	-3.856465

H	4.515318	1.713684	-5.240345
H	6.476198	0.169965	-1.714216
H	5.038627	1.557447	-0.321523
C	6.605735	-0.012573	-4.461565
C	6.435468	-1.504689	-4.115475
H	5.422495	-1.852178	-4.353899
H	7.141819	-2.115635	-4.698908
H	6.622471	-1.701306	-3.050330
C	6.352187	0.170431	-5.965269
H	7.052020	-0.452440	-6.542837
H	5.334054	-0.134021	-6.250038
H	6.503743	1.215638	-6.277311
C	8.062130	0.402600	-4.160062
H	8.226147	1.468295	-4.387406
H	8.319272	0.232312	-3.103032
H	8.761227	-0.189105	-4.772341
C	-3.419232	1.904427	-0.716290
C	-2.893927	0.697709	-0.205619
C	-3.675100	-0.453026	-0.122034
C	-5.023950	-0.467662	-0.517133
C	-5.541388	0.727964	-1.038837
C	-4.758172	1.879112	-1.147007
H	-1.870032	0.643940	0.152383
H	-3.197590	-1.349878	0.275462
H	-6.572405	0.776472	-1.388911
H	-5.196324	2.766993	-1.602664
C	-5.868353	-1.735770	-0.322830
C	-5.213334	-2.935245	-1.040642
H	-5.225096	-2.785390	-2.130648
H	-4.166637	-3.093321	-0.745357
H	-5.777024	-3.857636	-0.824125
C	-7.297507	-1.568168	-0.865274
H	-7.861592	-2.503305	-0.724656
H	-7.844155	-0.769608	-0.341003
H	-7.301408	-1.335418	-1.941868
C	-5.952819	-2.040929	1.189661
H	-4.960218	-2.253556	1.614167
H	-6.383990	-1.189450	1.739133
H	-6.593612	-2.920224	1.368764
C	2.686830	-2.708369	-4.907195
C	2.817249	-1.340722	-5.202332
C	2.319033	-0.803212	-6.387282
C	1.666305	-1.595508	-7.339859
C	1.537480	-2.967445	-7.044901
C	2.027922	-3.512821	-5.857077
H	3.312537	-0.669983	-4.505471
H	2.428006	0.267709	-6.536913
H	1.021418	-3.628370	-7.746754
H	1.867355	-4.572285	-5.641971
C	1.101323	-0.998275	-8.638394
C	-0.420985	-1.232838	-8.686009
H	-0.663030	-2.304750	-8.645843
H	-0.841150	-0.829376	-9.622211
H	-0.922161	-0.722572	-7.849926
C	1.761427	-1.685331	-9.851573
H	1.560962	-2.768540	-9.868234
H	1.369914	-1.256353	-10.788815
H	2.853946	-1.542805	-9.838170
C	1.348776	0.516267	-8.731977
H	2.423266	0.754953	-8.765555
H	0.894402	0.907334	-9.655819
H	0.902622	1.054480	-7.884496
C	2.125597	0.283680	2.000856
C	2.261040	1.625838	2.391785
C	1.214360	2.316723	2.996902
C	-0.025013	1.700070	3.237710
C	-0.168847	0.370938	2.804565
C	0.875327	-0.328624	2.200693
H	3.196780	2.152852	2.201910
H	1.381033	3.357014	3.271572
H	-1.116298	-0.152909	2.938546
H	0.704479	-1.357144	1.892390
C	-1.191039	2.456574	3.891640
C	-2.062383	3.061869	2.770980
H	-1.463975	3.723308	2.126773
H	-2.488655	2.273996	2.133331
H	-2.894847	3.648759	3.194842
C	-2.052119	1.510757	4.752435
H	-1.439025	0.989382	5.505488
H	-2.829808	2.085803	5.280190
H	-2.564486	0.750761	4.144457
C	-0.680322	3.592901	4.794803
H	-1.530080	4.085575	5.294073
H	0.001959	3.210195	5.571181
H	-0.145504	4.362613	4.221065
C	-1.228606	3.242723	-0.803053
C	1.791920	3.628409	-1.321738
C	3.183381	-1.391384	0.391823
C	3.370559	-2.553098	-2.461694
C	-2.657592	3.184837	-0.742327
C	3.279365	-3.297264	-3.678625
C	3.177050	3.374529	-1.093505
C	3.297046	-0.431452	1.439511
C	-1.273704	8.104281	-0.839861
C	-0.557872	6.926209	-0.886254
C	-1.211148	5.669388	-0.759646
C	-2.639278	5.644839	-0.658687
C	-3.344548	6.879216	-0.581974
C	-2.678932	8.083682	-0.656984
H	-0.754163	9.058815	-0.949327
H	0.524539	6.946531	-1.023124
C	-0.497346	4.427168	-0.721052
C	-3.317523	4.400912	-0.629339
H	-4.433548	6.851598	-0.484583
H	-4.401352	4.413069	-0.501688
C	2.825944	6.019069	3.010572
C	3.564268	5.286412	2.106633
C	2.966917	4.747938	0.931863
C	1.568917	4.963988	0.690674
C	0.832140	5.700472	1.659805

C	1.444909	6.224957	2.780153
H	4.790379	3.813513	0.242361
H	4.625230	5.090410	2.286317
C	3.729953	3.963347	0.030385
C	0.978705	4.376037	-0.485211
H	-0.236570	5.849761	1.523086
H	0.851635	6.786797	3.505407
C	8.011865	-1.894162	0.126969
C	6.759541	-2.271584	-0.313637
C	5.579999	-1.733297	0.270943
C	5.726019	-0.773367	1.327269
C	7.031057	-0.413168	1.766248
C	8.152813	-0.956993	1.180025
H	8.899320	-2.321849	-0.345289
H	6.669144	-2.984030	-1.131277
C	4.254665	-2.058923	-0.182007
C	4.577756	-0.167132	1.895613
H	7.125712	0.317699	2.573864
H	4.717809	0.539936	2.715715
C	5.516441	-6.992942	-1.385517
C	4.979757	-6.437403	-2.526519
C	4.442554	-5.120264	-2.517614
C	4.515596	-4.349260	-1.309564
C	5.051492	-4.961301	-0.142812
C	5.533398	-6.253174	-0.177591
H	3.850791	-5.148870	-4.597420
H	4.944154	-7.008092	-3.458107
H	5.065580	-4.398004	0.790532
H	5.921620	-6.714206	0.732735
C	3.845931	-4.560883	-3.677227
C	4.030158	-2.998925	-1.322763
H	-3.235236	9.021900	-0.608391
H	3.299042	6.429174	3.906018
H	9.147038	-0.659400	1.522726
H	5.902662	-8.014335	-1.401033
C	0.386850	-4.275387	-0.886262
C	0.611421	-5.136868	-1.945283
C	1.005477	-4.566426	0.356929
C	1.353896	-6.340298	-1.810468
C	1.771471	-5.702662	0.518483
C	1.928341	-6.642261	-0.526929
C	1.525639	-7.276021	-2.866580
C	2.634632	-7.860752	-0.359538
C	2.175050	-8.473682	-2.654896
C	2.737120	-8.765136	-1.391336
F	0.116139	-4.840922	-3.147837
F	1.061638	-7.023803	-4.091438
F	2.300281	-9.360234	-3.637490
F	3.421944	-9.892069	-1.229300
F	3.227960	-8.150634	0.795012
F	2.352711	-5.910381	1.699346
F	0.899659	-3.764037	1.404019
C	-0.906650	3.495991	-4.718220
C	-2.232730	3.659899	-4.364494
C	-0.092258	4.651741	-4.817297
C	-2.814763	4.930075	-4.125256
F	-2.998354	2.583997	-4.200717
C	-0.610060	5.906104	-4.555136
F	1.188724	4.580372	-5.146099
C	-1.973882	6.092845	-4.224414
C	-4.180347	5.101623	-3.776576
F	0.206128	6.956415	-4.623709
C	-2.542597	7.371426	-3.979891
C	-4.691350	6.351890	-3.515507
F	-4.999101	4.054911	-3.669044
C	-3.869169	7.494525	-3.630714
F	-1.809389	8.479251	-4.057182
F	-5.952102	6.498066	-3.117985
F	-4.384341	8.685162	-3.350730
C	-2.438850	-1.889610	-4.823696
C	-2.942439	-0.906454	-3.862064
C	-4.249163	-0.205422	-4.244001
C	-5.297444	-1.181593	-4.763048
C	-4.729527	-2.016061	-5.906823
H	-2.144781	-0.202099	-3.582641
H	-3.061256	-1.540598	-2.949172
H	-4.613119	0.349959	-3.371979
H	-4.007386	0.539840	-5.016902
H	-5.649365	-1.830600	-3.945524
H	-6.184070	-0.632724	-5.120539
H	-5.455360	-2.771489	-6.249513
H	-4.543097	-1.354787	-6.770208
C	-1.009687	-2.045283	-4.942477
H	-0.662096	-2.902599	-5.519198
H	-0.693763	-1.097962	-5.439844
H	-0.514488	-1.969282	-3.952266
C	-3.400081	-2.750627	-5.584815
C	-3.637847	-3.933180	-4.582937
H	-4.238071	-4.688064	-5.113858
H	-2.693141	-4.387963	-4.253910
H	-4.175766	-3.612039	-3.685119
C	-2.834122	-3.327858	-6.891325
H	-3.603985	-3.961546	-7.357009
H	-2.581698	-2.526739	-7.597448
H	-1.942869	-3.949922	-6.733342

2e:

O	1.241292	2.476546	-1.925947
O	-0.602458	1.797001	-0.345339
O	1.949192	-2.195889	-0.038598
O	2.374666	-1.600201	-2.461717

S	-1.239784	-2.875246	-0.789762
S	-0.482281	1.810072	-4.372367
O	-1.539687	-1.836718	0.202686
O	-2.327688	-3.386813	-1.638743
O	0.926443	1.863436	-4.786549
O	-1.457695	1.244160	-5.317782
N	0.039290	-2.589827	-1.712935
N	-0.739174	1.184280	-2.930424
P	1.216154	-1.549347	-1.328005
N	0.919299	-0.036032	-1.116202
P	0.211794	1.253793	-1.638932
C	4.041120	2.291877	-1.965368
C	3.677466	2.513847	-3.304831
C	4.381240	1.923376	-4.354491
C	5.478493	1.080055	-4.123915
C	5.832537	0.854266	-2.781100
C	5.136134	1.444356	-1.725349
H	2.821928	3.140908	-3.545917
H	4.044434	2.134635	-5.368978
H	6.668518	0.196346	-2.539510
H	5.438603	1.222980	-0.701571
C	6.289707	0.435656	-5.262382
C	6.358417	-1.090300	-5.058896
H	5.361049	-1.543582	-5.129793
H	6.996919	-1.550509	-5.829615
H	6.781983	-1.356687	-4.078961
C	5.675554	0.704177	-6.646939
H	6.274139	0.196956	-7.419730
H	4.646253	0.322349	-6.719211
H	5.667363	1.778344	-6.888243
C	7.721679	1.012956	-5.255231
H	7.711096	2.096434	-5.455743
H	8.222344	0.851098	-4.287655
H	8.331508	0.527706	-6.034664
C	-3.458700	1.990106	-0.746513
C	-3.239348	0.774622	-0.069272
C	-4.169867	-0.259994	-0.131443
C	-5.372604	-0.143100	-0.847482
C	-5.563994	1.047869	-1.566335
C	-4.624279	2.081042	-1.528771
H	-2.336579	0.621452	0.515982
H	-3.932390	-1.177568	0.405609
H	-6.464022	1.193622	-2.165375
H	-4.804928	2.975782	-2.127203
C	-6.406795	-1.280237	-0.796031
C	-5.840159	-2.540385	-1.484564
H	-5.727136	-2.365137	-2.565410
H	-4.857645	-2.832368	-1.086894
H	-6.528666	-3.391992	-1.356041
C	-7.726050	-0.899652	-1.488444
H	-8.448142	-1.726841	-1.397587
H	-8.181241	-0.007221	-1.031196
H	-7.587757	-0.699554	-2.563117
C	-6.715877	-1.613327	0.681222
H	-5.822028	-1.974647	1.210795
H	-7.092776	-0.726737	1.215231
H	-7.483105	-2.403071	0.744936
C	2.696646	-2.564390	-5.080185
C	2.519395	-1.177003	-5.216622
C	1.884471	-0.637211	-6.333297
C	1.389591	-1.446051	-7.366191
C	1.570298	-2.835294	-7.230274
C	2.208579	-3.383374	-6.114505
H	2.884067	-0.494130	-4.452398
H	1.758056	0.441784	-6.358993
H	1.203237	-3.513513	-8.004641
H	2.315196	-4.468318	-6.035940
C	0.704913	-0.842486	-8.601011
C	-0.602005	-1.597939	-8.911274
H	-0.433251	-2.660207	-9.147195
H	-1.104480	-1.142159	-9.779836
H	-1.291701	-1.537519	-8.055217
C	1.665346	-0.958096	-9.803921
H	1.920831	-2.006937	-10.022880
H	1.206796	-0.520594	-10.706493
H	2.604605	-0.419558	-9.602555
C	0.352243	0.640564	-8.384771
H	-0.199656	1.021801	-9.258429
H	-0.275523	0.790725	-7.494089
H	1.252482	1.261359	-8.269000
C	1.936732	-0.152725	2.029252
C	1.917836	1.211547	2.374651
C	0.797753	1.793456	2.953266
C	-0.368625	1.053388	3.213197
C	-0.357092	-0.297859	2.842072
C	0.769999	-0.892343	2.268867
H	2.785950	1.835501	2.165020
H	0.840333	2.853629	3.199921
H	-1.236909	-0.923338	2.989851
H	0.726429	-1.944844	2.008402
C	-1.556110	1.732815	3.911247
C	-2.062796	2.918307	3.064029
H	-1.261203	3.636950	2.841472
H	-2.474639	2.568316	2.107854
H	-2.862798	3.456395	3.598651
C	-2.728974	0.765717	4.135815
H	-2.435578	-0.093561	4.758257
H	-3.548135	1.288035	4.655187
H	-3.130689	0.379860	3.186272
C	-1.078170	2.256085	5.285799
H	-1.910741	2.735637	5.827499
H	-0.689134	1.432215	5.905635
H	-0.274215	3.000255	5.177494
C	-1.153919	3.065259	-0.403009
C	1.901574	3.141359	-0.913590
C	3.167593	-1.660388	0.358626
C	3.325714	-2.591046	-2.616496
C	-2.562596	3.169819	-0.609252
C	3.449584	-3.134854	-3.932627
C	3.318667	2.977496	-0.860796

C	3.172560	-0.715354	1.426997
C	-0.620731	7.893510	-0.529144
C	-0.045149	6.644234	-0.422152
C	-0.843739	5.467514	-0.405903
C	-2.261548	5.607465	-0.569687
C	-2.824582	6.910770	-0.663954
C	-2.026389	8.032554	-0.634293
H	0.013419	8.783075	-0.538860
H	1.038437	6.549366	-0.353902
C	-0.295362	4.147686	-0.265483
C	-3.079225	4.454049	-0.655317
H	-3.908536	7.003342	-0.775683
H	-4.156268	4.593287	-0.767007
C	3.255327	5.429124	3.373346
C	3.934586	4.740383	2.391172
C	3.264379	4.253330	1.233342
C	1.854588	4.488255	1.094377
C	1.185802	5.206648	2.124986
C	1.864983	5.661882	3.236659
H	5.050702	3.432626	0.316353
H	5.004951	4.540463	2.495193
C	3.966281	3.533682	0.228120
C	1.166970	3.928815	-0.037132
H	0.113441	5.379632	2.041424
H	1.322335	6.194943	4.021349
C	7.974678	-1.212792	-0.231251
C	6.780602	-1.756883	-0.657532
C	5.566348	-1.518720	0.044997
C	5.613725	-0.643961	1.182464
C	6.863311	-0.109317	1.605093
C	8.024471	-0.389647	0.920120
H	8.888255	-1.419124	-0.793922
H	6.767089	-2.369703	-1.556015
C	4.290910	-2.064619	-0.348170
C	4.416020	-0.264474	1.836110
H	6.877506	0.543004	2.482041
H	4.481545	0.430434	2.676055
C	6.823518	-6.217065	-2.204038
C	6.105784	-5.694721	-3.258079
C	5.168141	-4.643325	-3.055584
C	5.026037	-4.076082	-1.746950
C	5.746224	-4.668621	-0.673896
C	6.618846	-5.714597	-0.894971
H	4.571416	-4.510853	-5.132380

H	6.237870	-6.090721	-4.268552
H	5.594589	-4.282977	0.334986
H	7.156966	-6.159938	-0.055123
C	4.394743	-4.126449	-4.125347
C	4.171158	-2.933215	-1.562277
H	-2.473825	9.026673	-0.710713
H	3.784547	5.790668	4.258337
H	8.974925	0.032866	1.254999
H	7.534560	-7.029350	-2.373981
C	-2.226238	-2.126170	-4.466684
C	-3.078682	-1.208111	-3.690115
C	-4.070291	-0.490274	-4.657191
C	-4.798349	-1.482016	-5.561168
C	-3.814348	-2.422748	-6.251075
H	-2.476689	-0.467761	-3.149610
H	-3.654181	-1.797548	-2.963516
H	-4.766687	0.085213	-4.030247
H	-3.490062	0.230544	-5.250149
H	-5.535972	-2.063837	-4.984825
H	-5.369371	-0.931847	-6.327910
H	-4.328719	-3.180429	-6.866190
H	-3.168436	-1.843729	-6.932192
C	-0.813931	-1.914145	-4.501118
H	-0.247836	-2.387468	-5.305551
H	-0.531051	-0.877306	-4.306632
H	-0.518013	-2.426276	-3.522016
C	-2.886640	-3.217790	-5.250602
C	-3.734366	-4.144042	-4.348712
H	-4.284766	-4.846918	-4.993567
H	-3.081751	-4.708698	-3.669263
H	-4.455323	-3.602531	-3.728454
C	-1.906779	-4.084826	-6.050971
H	-2.474239	-4.829731	-6.629048
H	-1.306128	-3.496459	-6.757638
H	-1.219110	-4.625228	-5.382183
C	-0.749226	-4.356240	0.258236
F	-0.200778	-5.308018	-0.487308
F	-1.840143	-4.838922	0.850955
F	0.120197	-3.985990	1.198052
C	-0.999223	3.610393	-4.257655
F	-2.264389	3.695877	-3.847068
F	-0.225236	4.301005	-3.422590
F	-0.893360	4.146480	-5.47017

9. Copies of NMR spectra

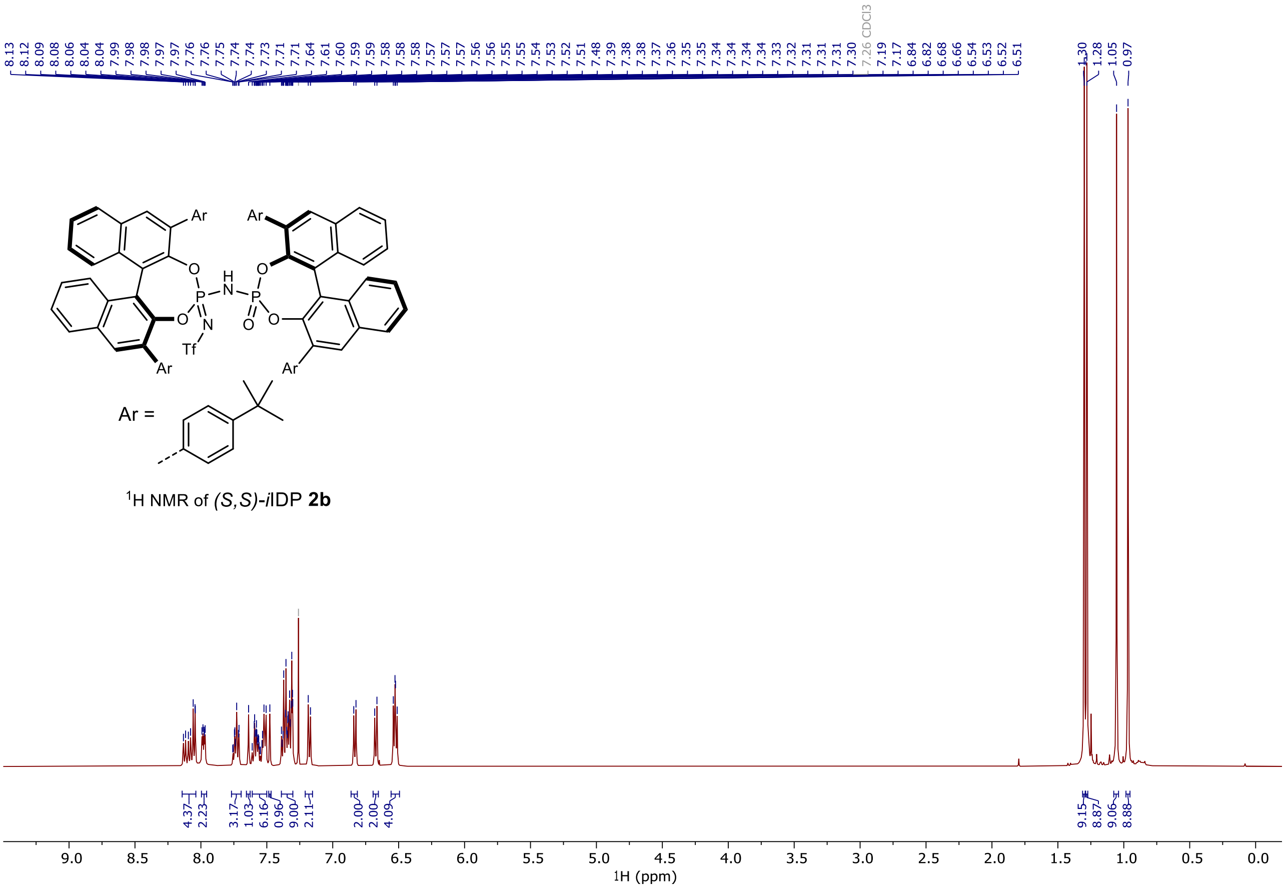

^{1}H NMR of *(S,S)*-*i*IDP **2b**

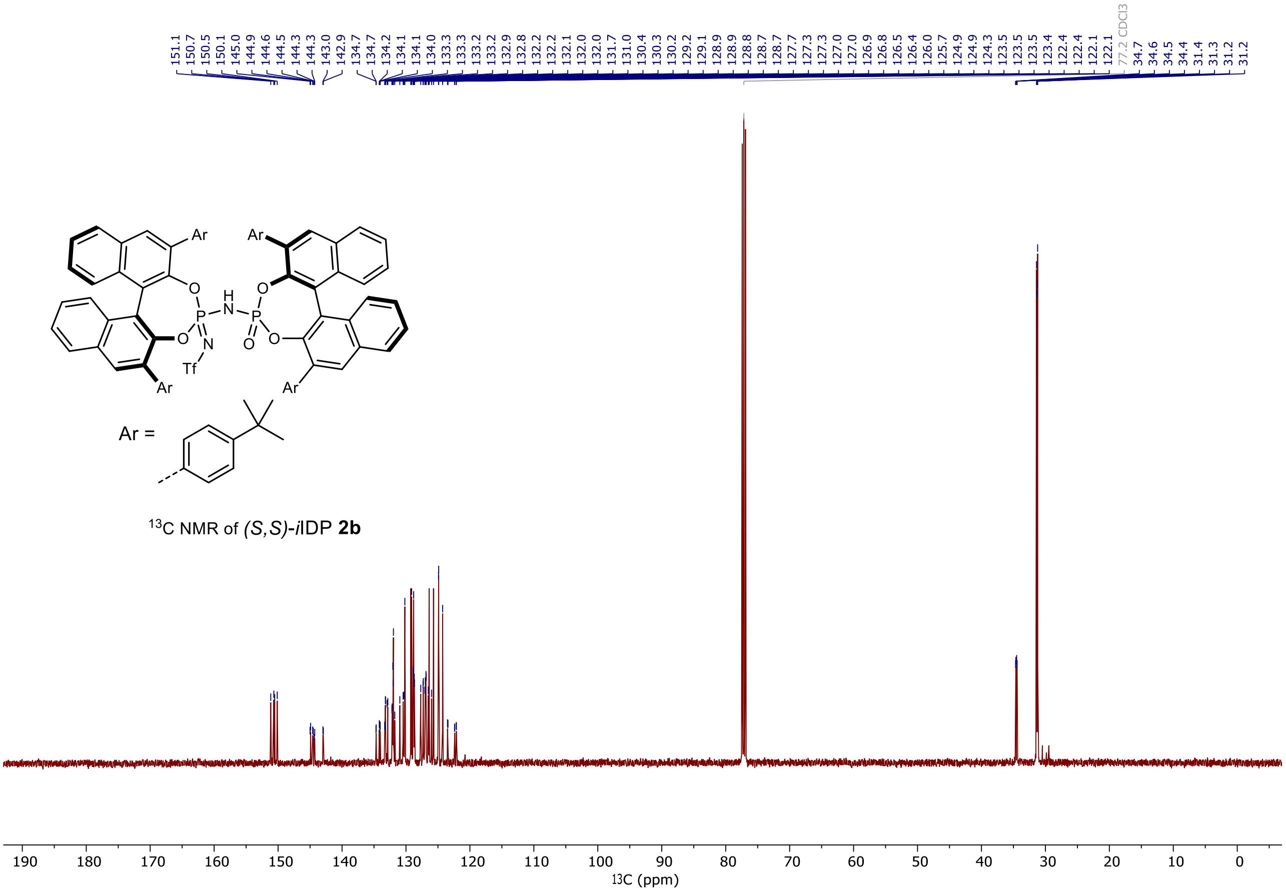

^{13}C NMR of *(S,S)*-*i*IDP **2b**

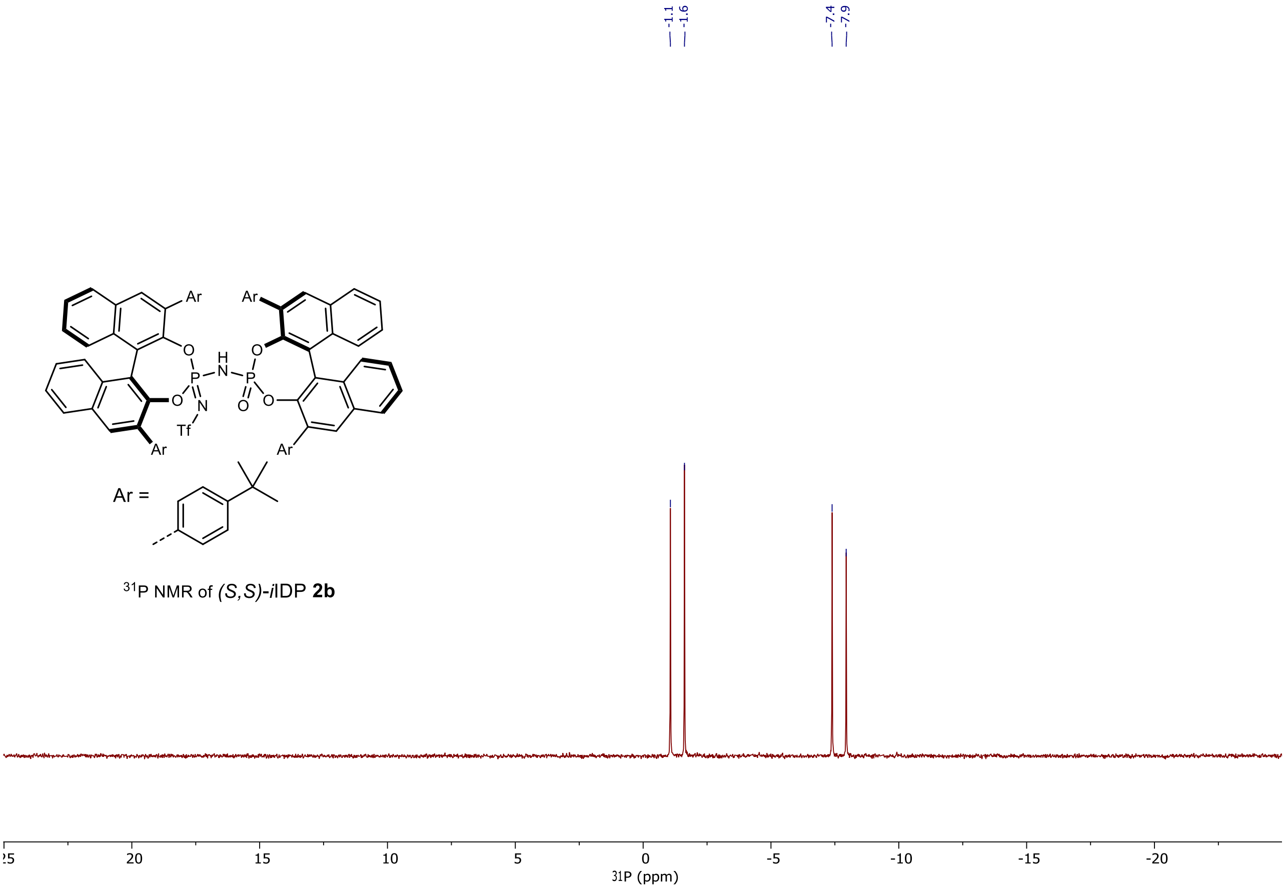

^{31}P NMR of *(S,S)-i*IDP **2b**

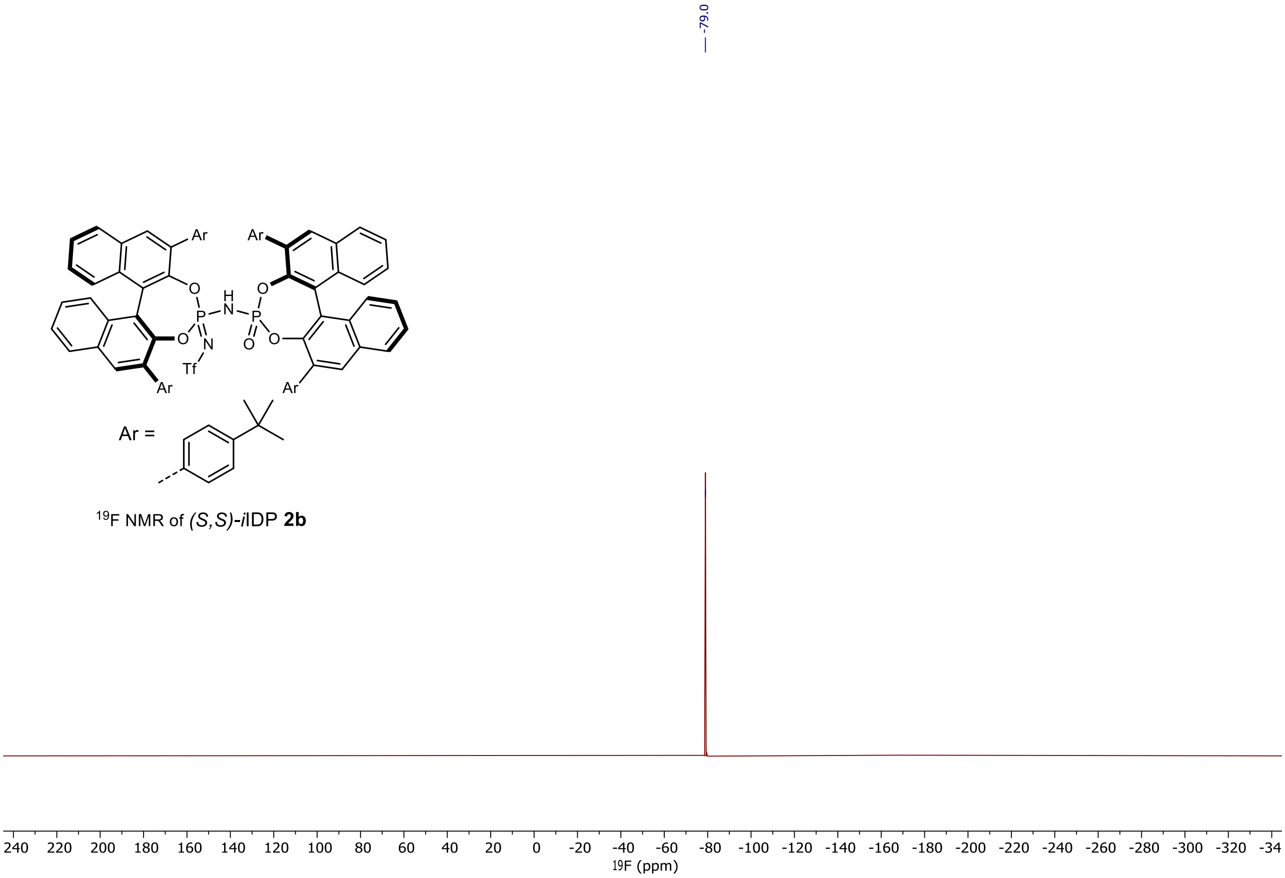

^{19}F NMR of *(S,S)-i*IDP **2b**

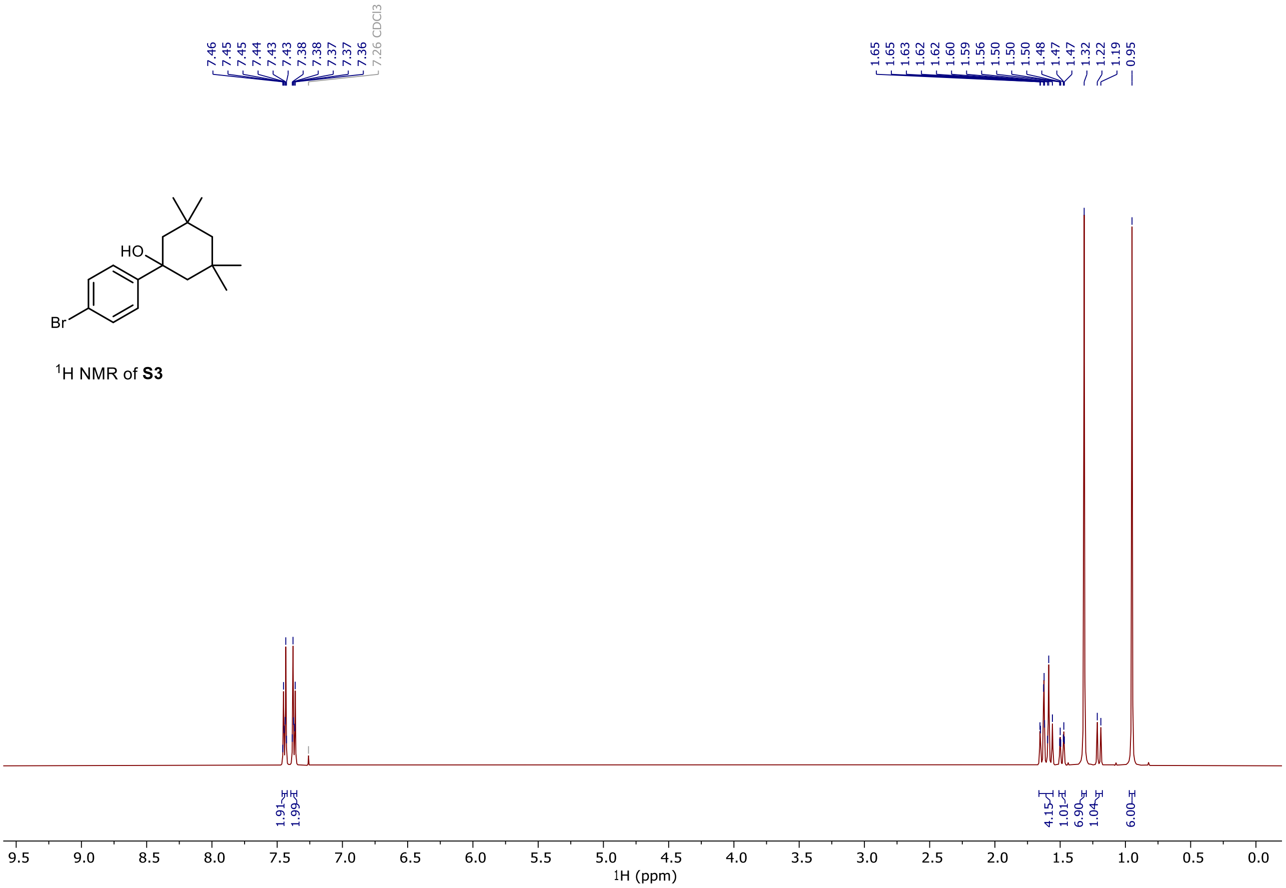
7.46 7.45 7.45 7.44 7.43 7.43 7.38 7.38 7.37 7.37 7.36 7.26 CDCl3
1.65 1.65 1.63 1.62 1.62 1.60 1.59 1.56 1.50 1.50 1.48 1.47 1.47 1.32 1.22 1.19 0.95
HO
Br
^{1}H NMR of **S3**
1.91 1.99
4.15 1.01 6.90 1.04 6.00
1H (ppm)

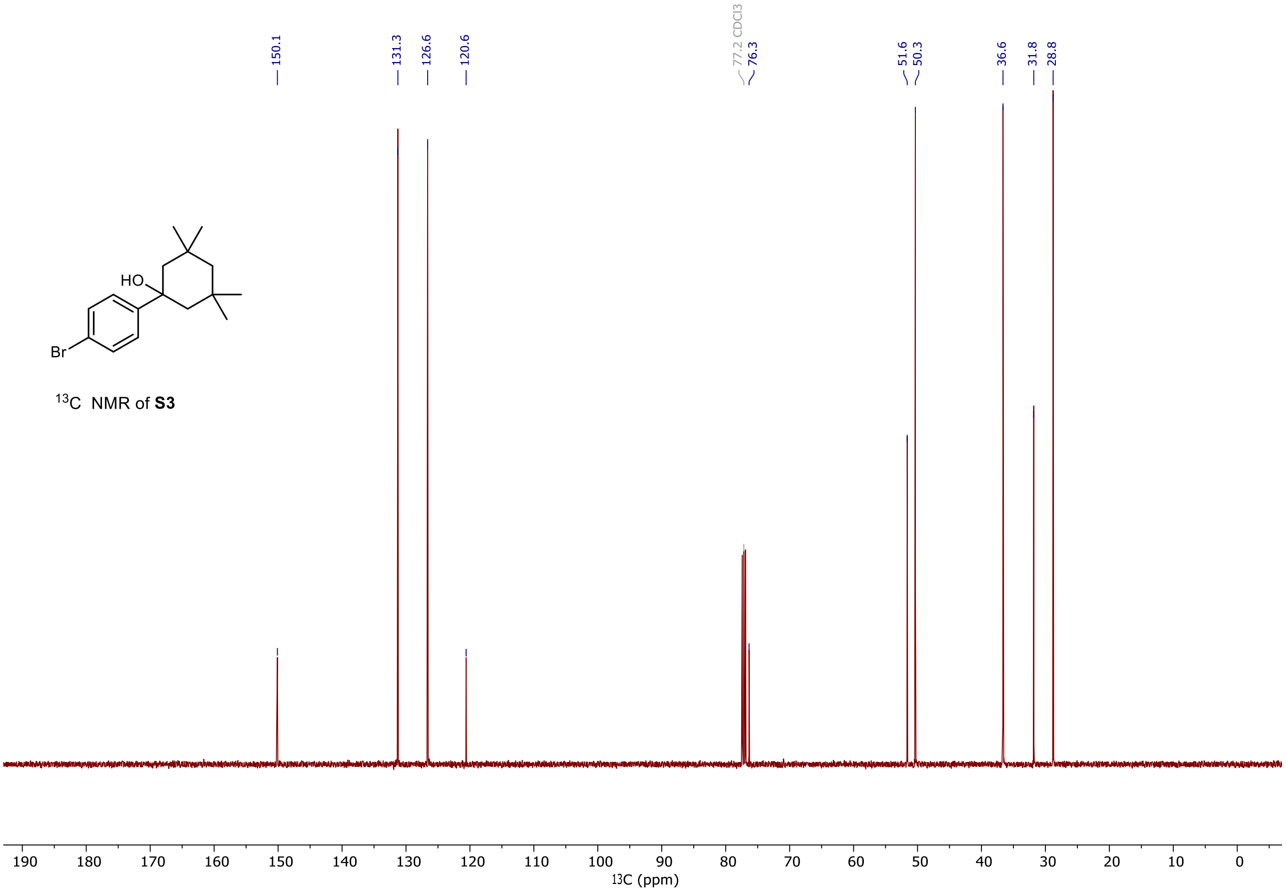
150.1
131.3 126.6 120.6
77.2 CDCl3 76.3
51.6 50.3
36.6 31.8 28.8
HO
Br
^{13}C NMR of **S3**
13C (ppm)

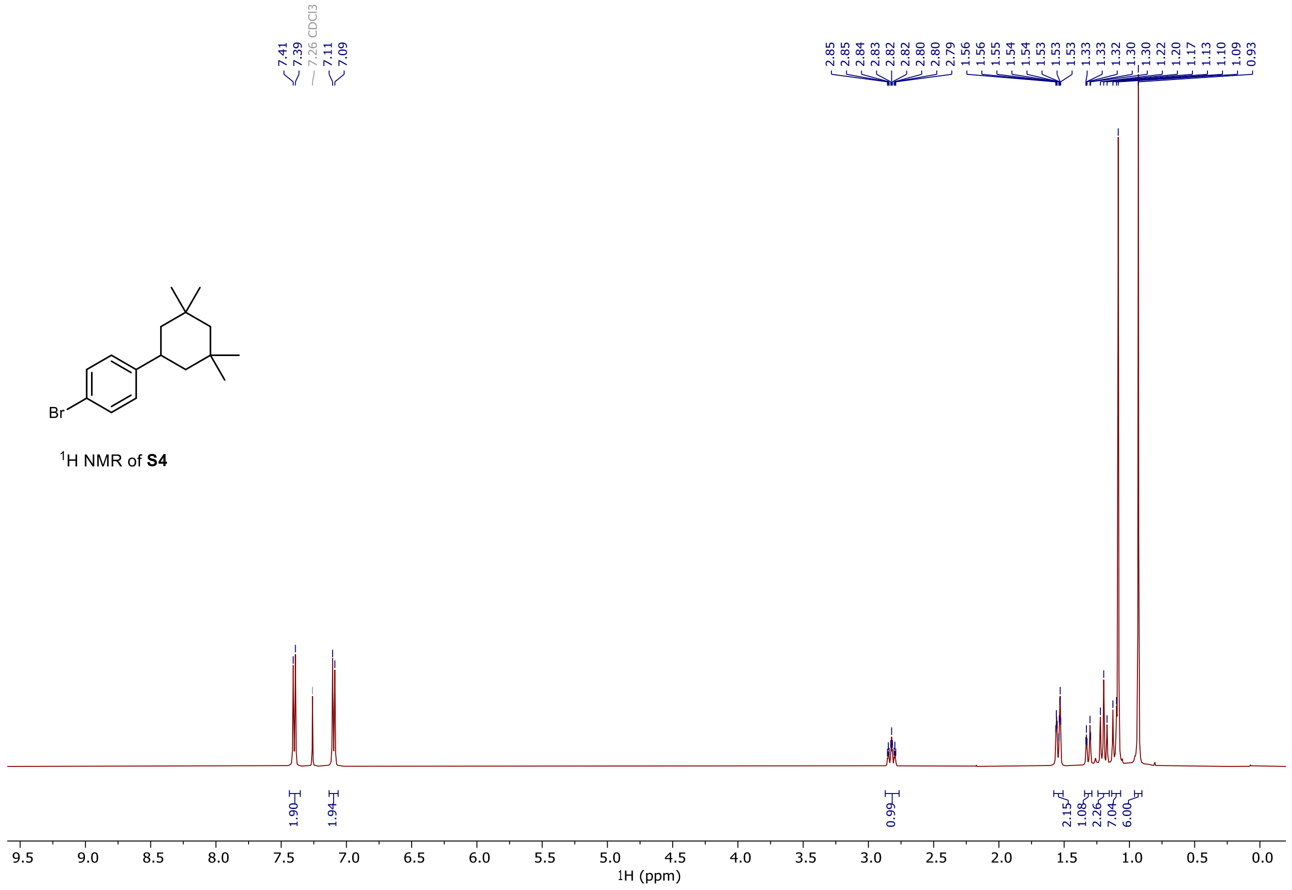

^{1}H NMR of **S4**

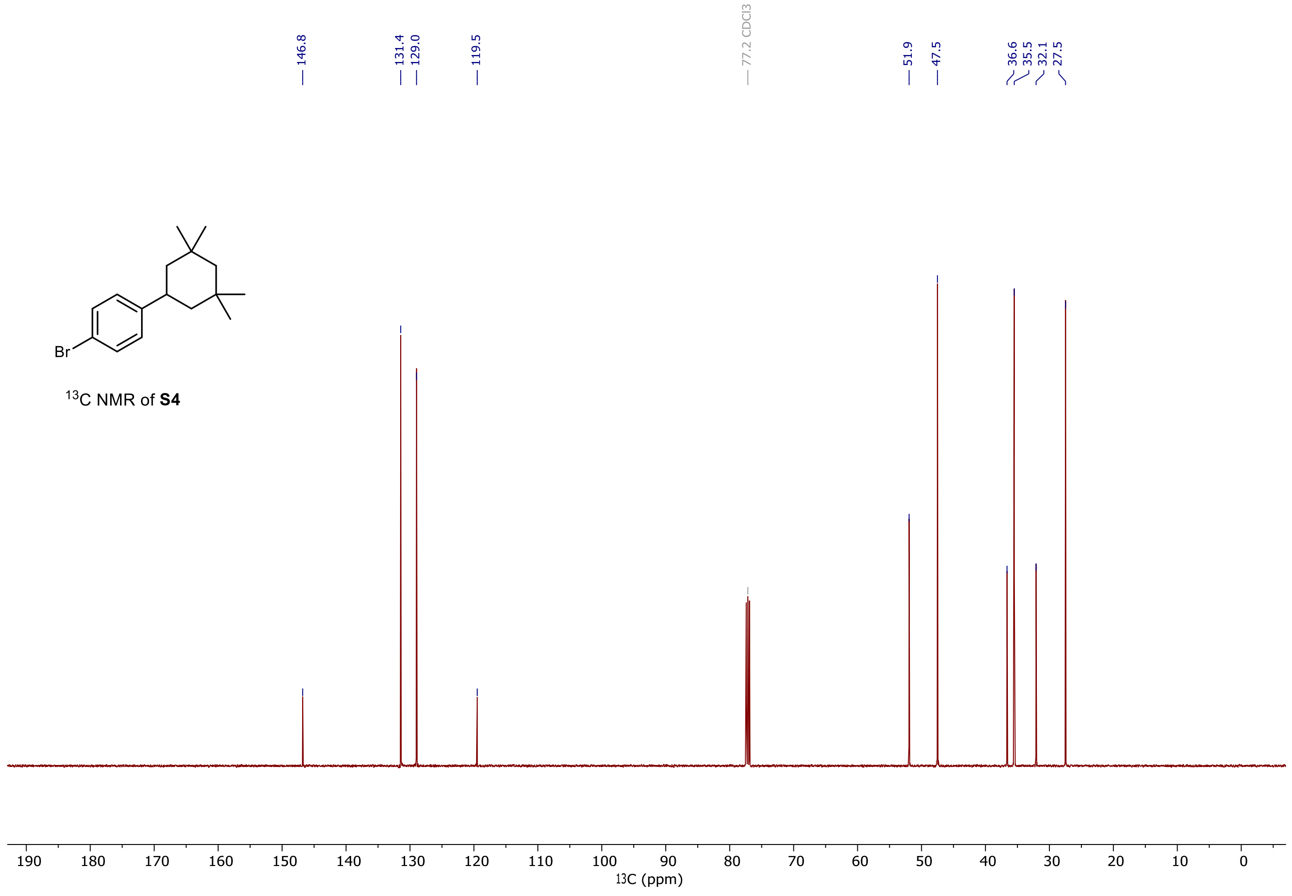

^{13}C NMR of **S4**

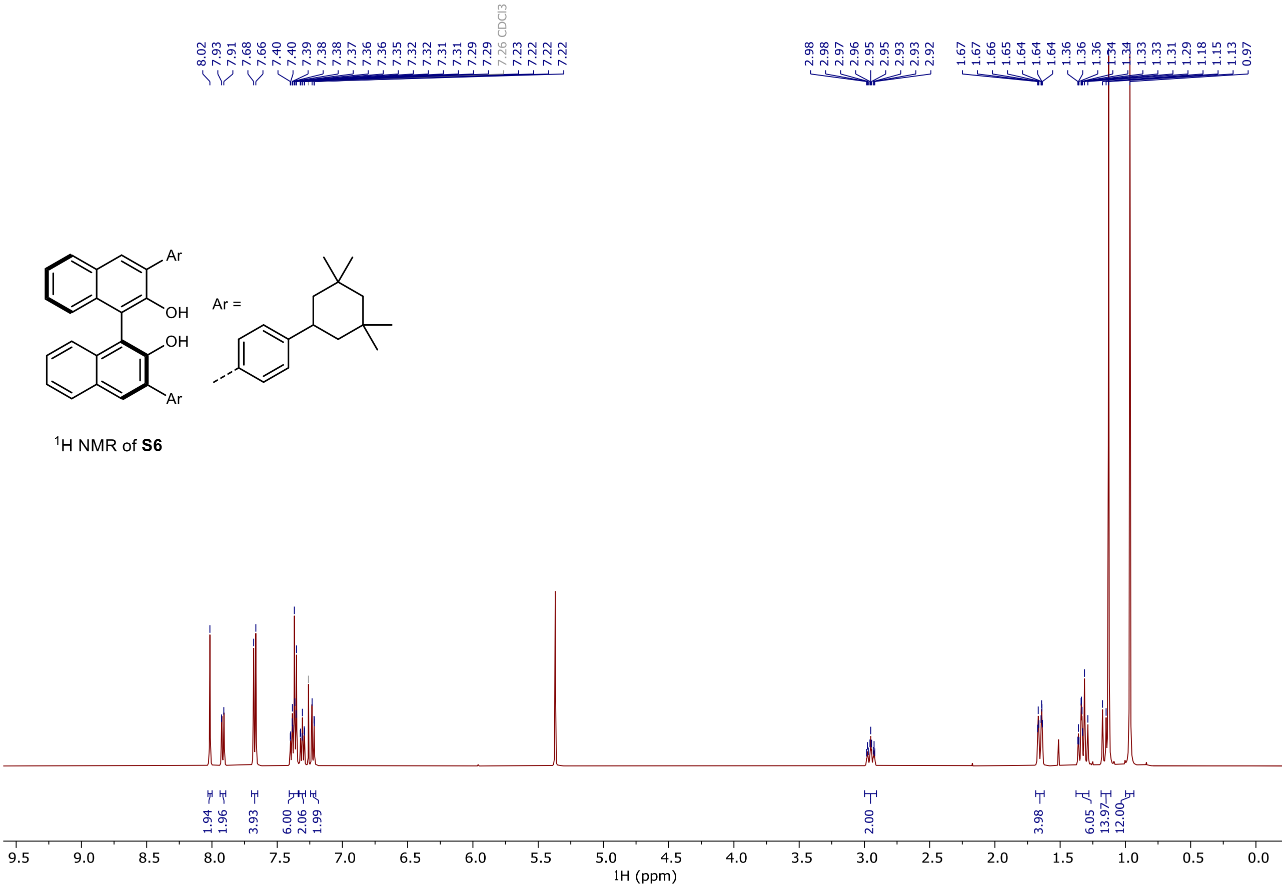
8.02
7.93
7.91
7.68
7.66
7.40
7.39
7.38
7.38
7.37
7.36
7.36
7.35
7.32
7.32
7.31
7.31
7.29
7.29
7.26 CDCl3
7.23
7.22
7.22
7.22
2.98
2.98
2.97
2.96
2.95
2.95
2.93
2.93
2.92
1.67
1.67
1.66
1.65
1.64
1.64
1.64
1.36
1.36
1.34
1.34
1.33
1.33
1.31
1.29
1.18
1.15
1.13
0.97
Ar
OH
OH
Ar
Ar =
^{1}H NMR of **S6**
1.94
1.96
3.93
6.00
2.06
1.99
2.00
3.98
6.05
13.97
12.00
1H (ppm)

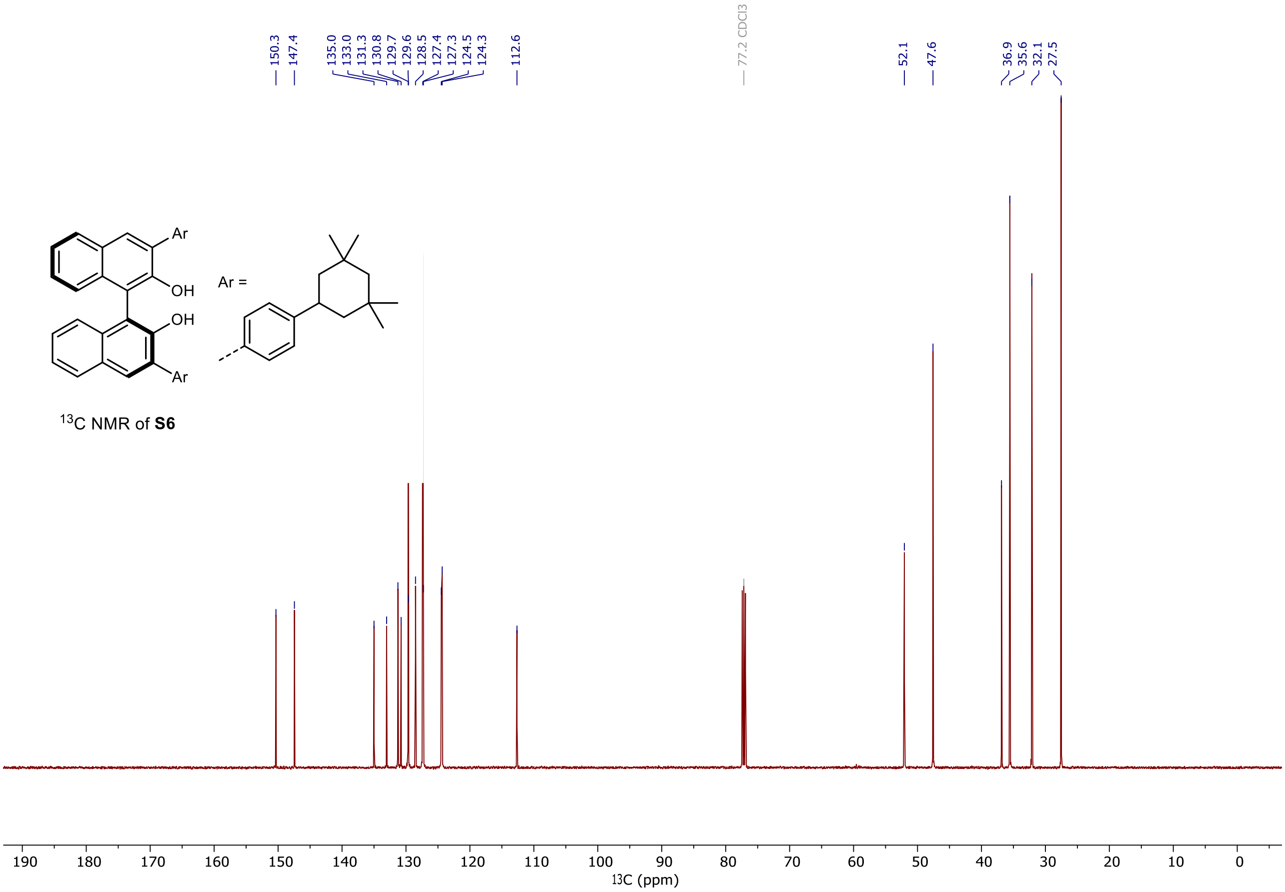
150.3
147.4
135.0
133.0
131.3
130.8
129.7
129.6
128.5
127.4
127.3
124.5
124.3
112.6
77.2 CDCl3
52.1
47.6
36.9
35.6
32.1
27.5
Ar
OH
OH
Ar
Ar =
^{13}C NMR of **S6**
13C (ppm)

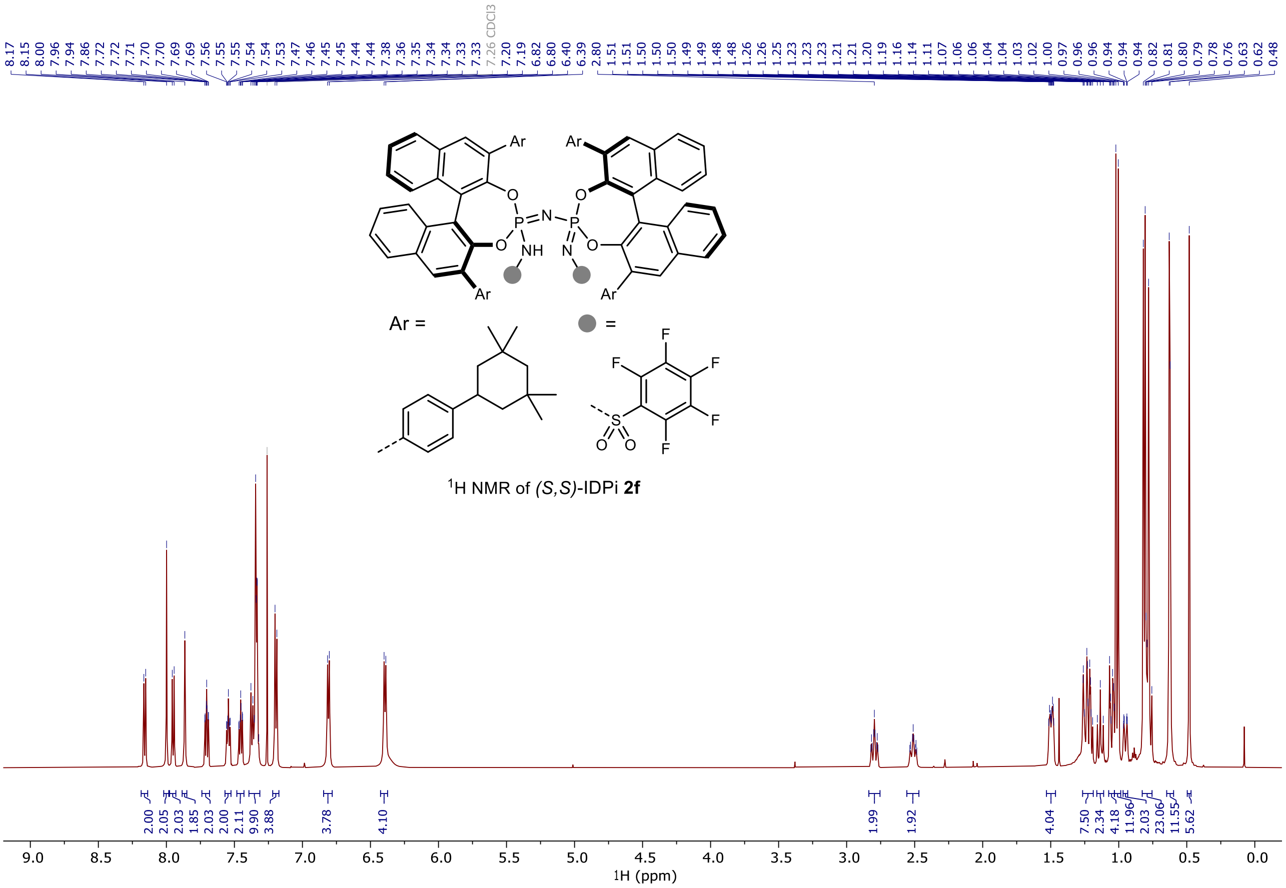

^{1}H NMR of *(S,S)*-IDPi **2f**

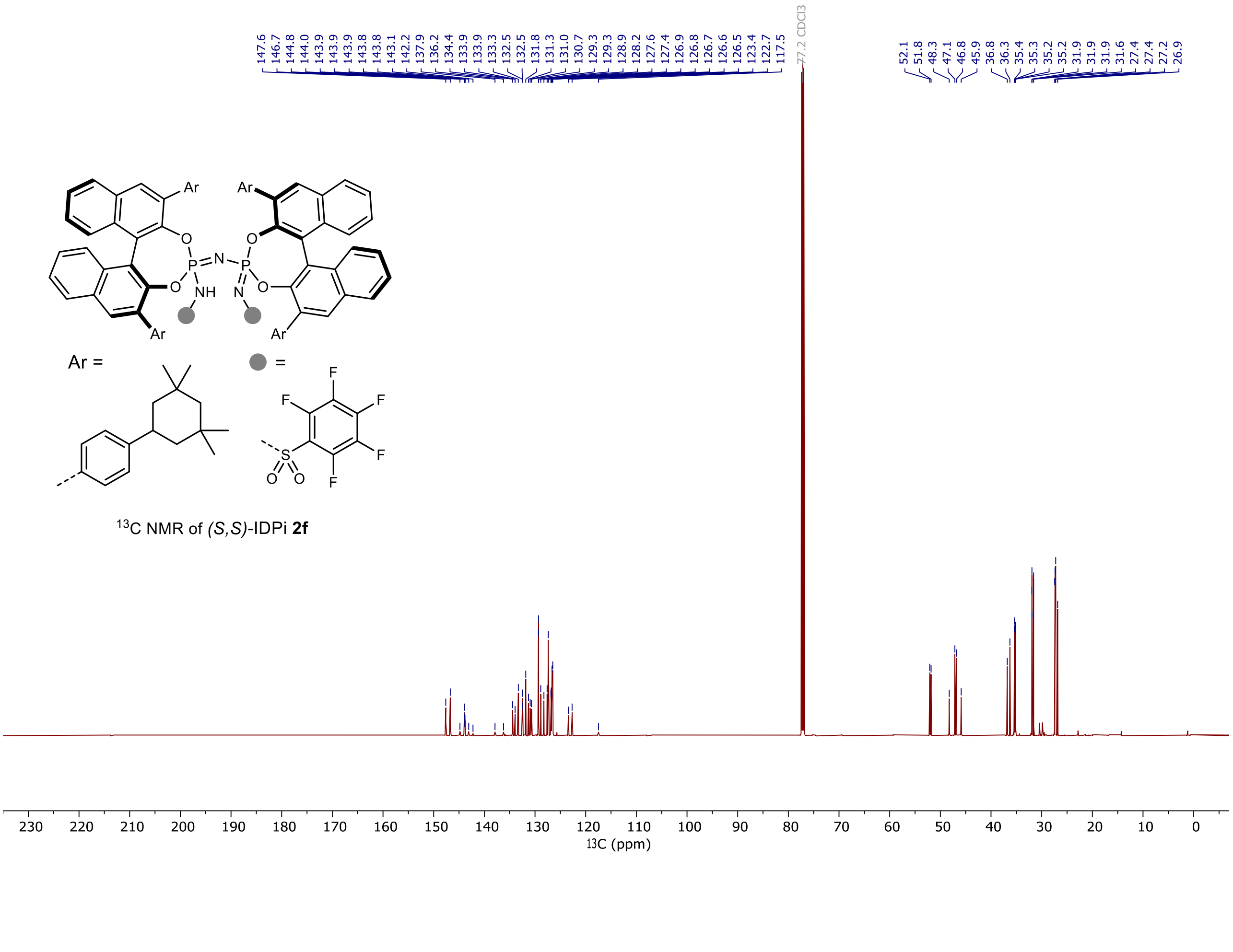

^{13}C NMR of *(S,S)*-IDPi **2f**

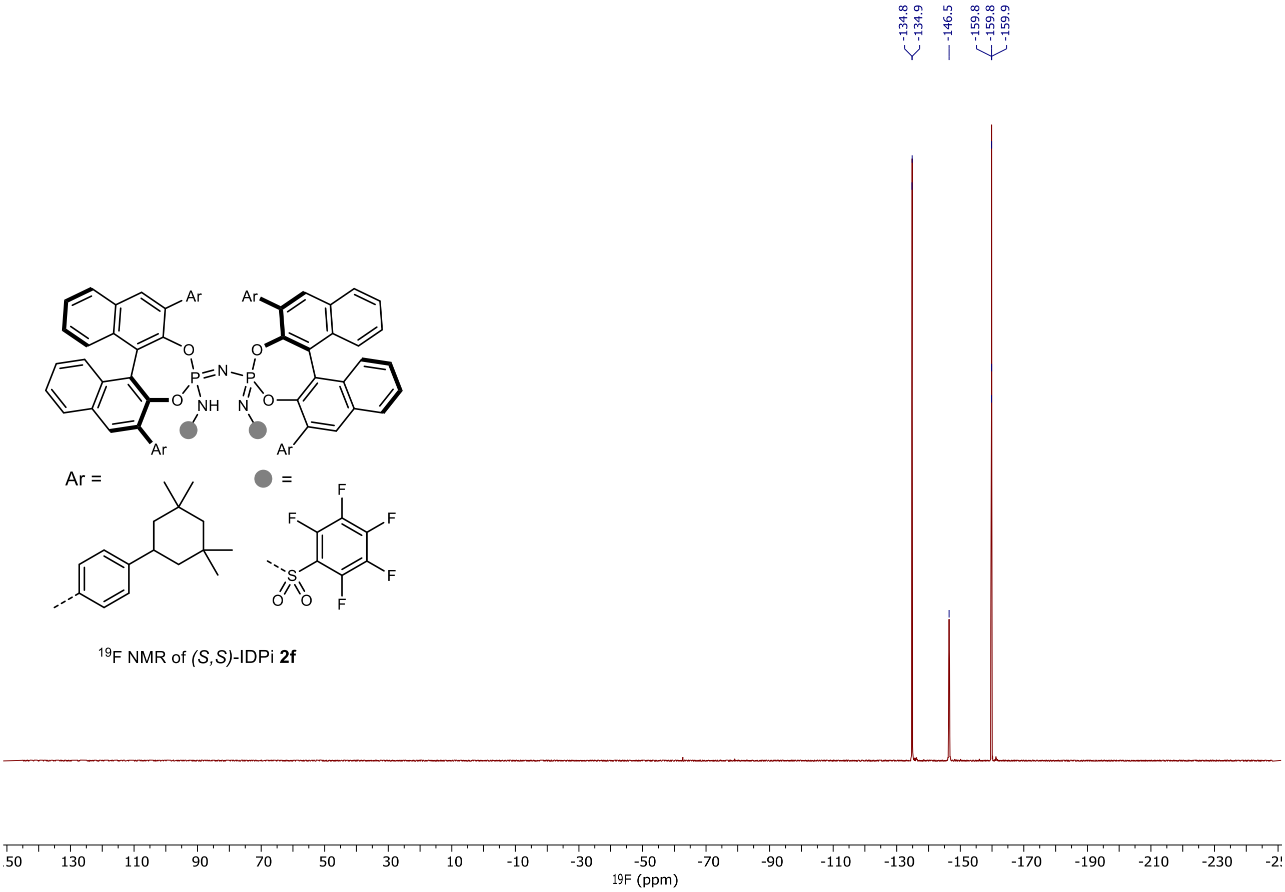
-134.8
-134.9
-146.5
-159.8
-159.8
-159.9
Ar
Ar
O
P
N
NH
O
Ar =
F
S
O
^{19}F NMR of (S,S)-IDPi 2f
19F (ppm)

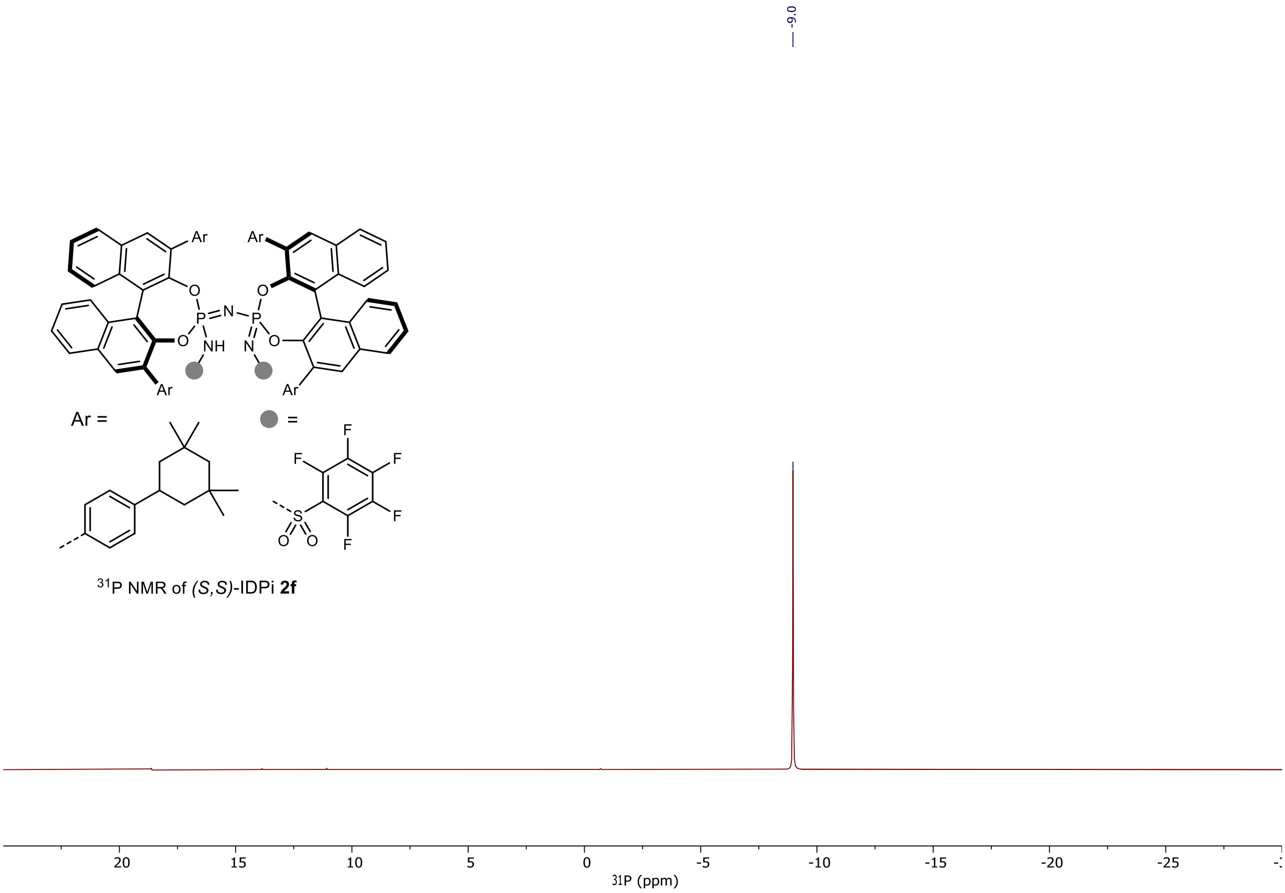
-9.0
Ar
Ar
O
P
N
NH
O
Ar =
F
S
O
^{31}P NMR of (S,S)-IDPi 2f
31P (ppm)

1H{off},1D, 600.20 MHz,CDCl3,298.0K, pulse sequence: zg30

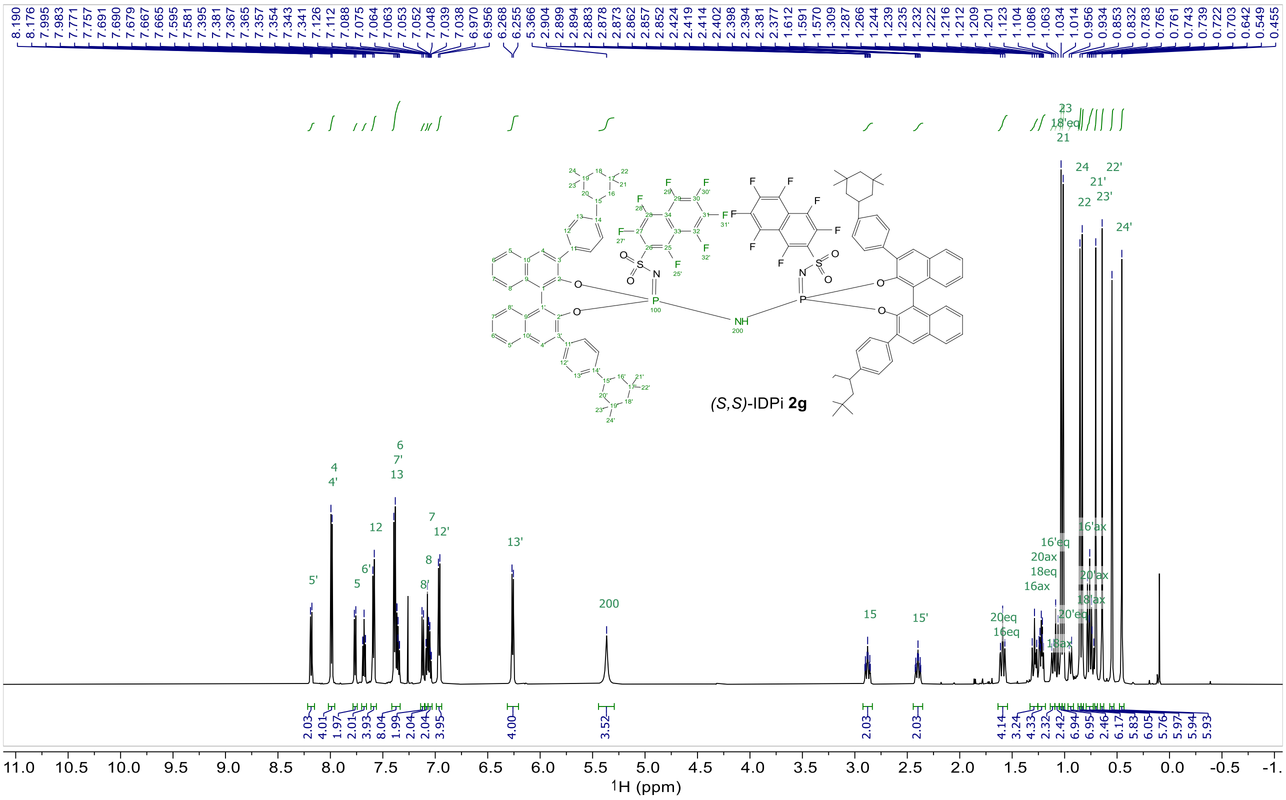

13C{1H},1D, 150.94 MHz,CDCl3,298.0K, pulse sequence: zgpg30

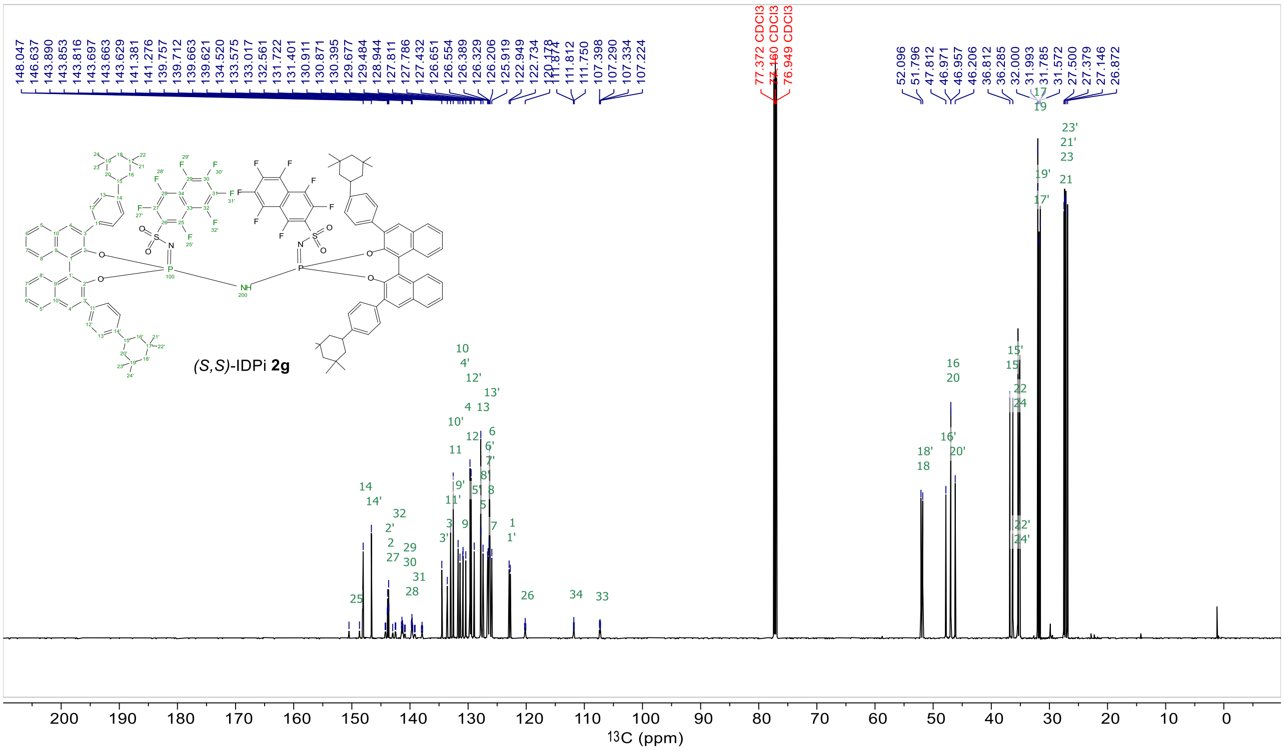

31P{1H},1D, 242.98 MHz,CDCl3,298.0K, pulse sequence: zgpg30

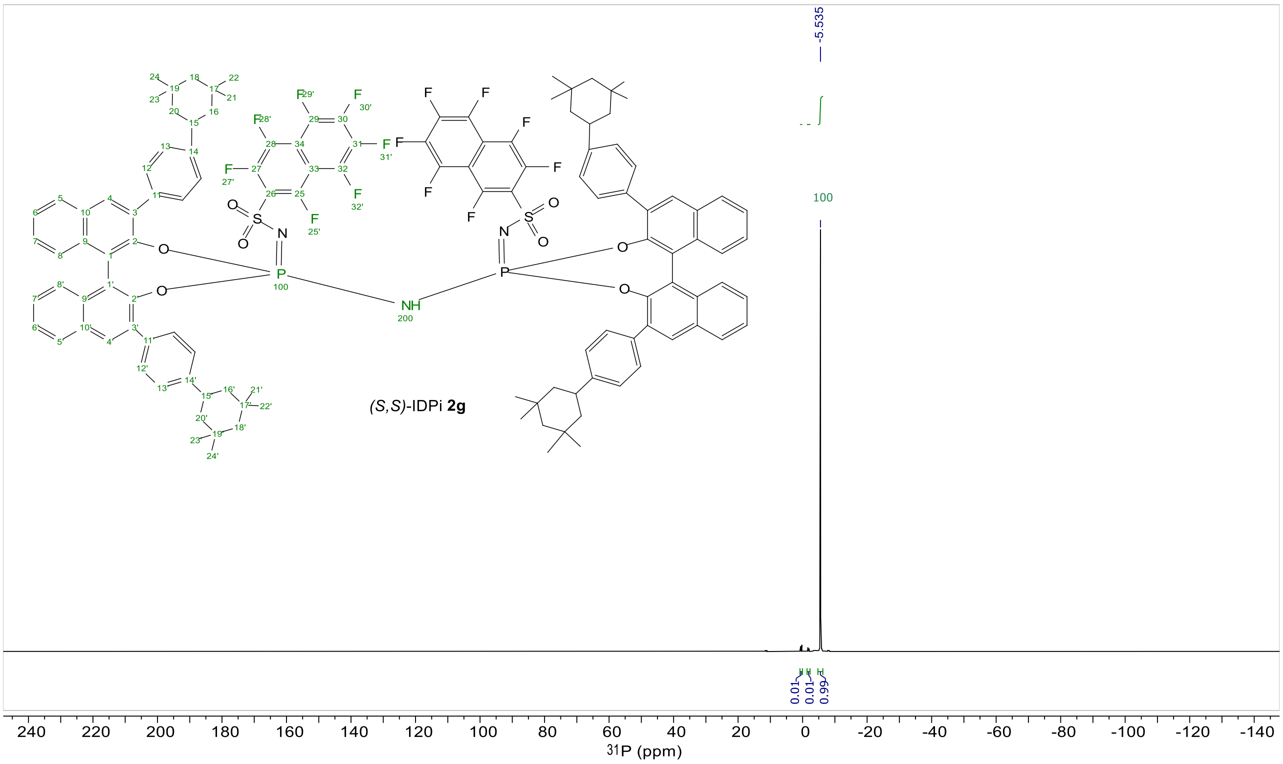

19F{off},1D, 564.72 MHz,CDCl3,298.0K, pulse sequence: zg30

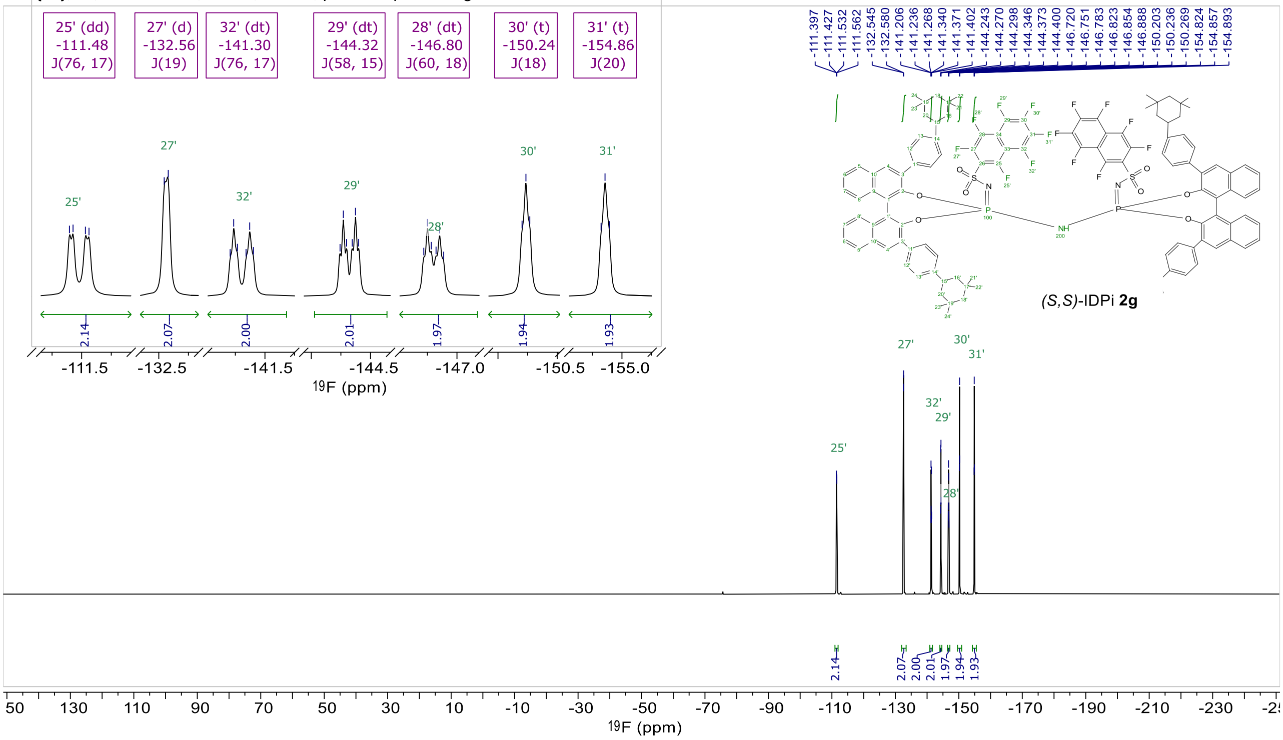

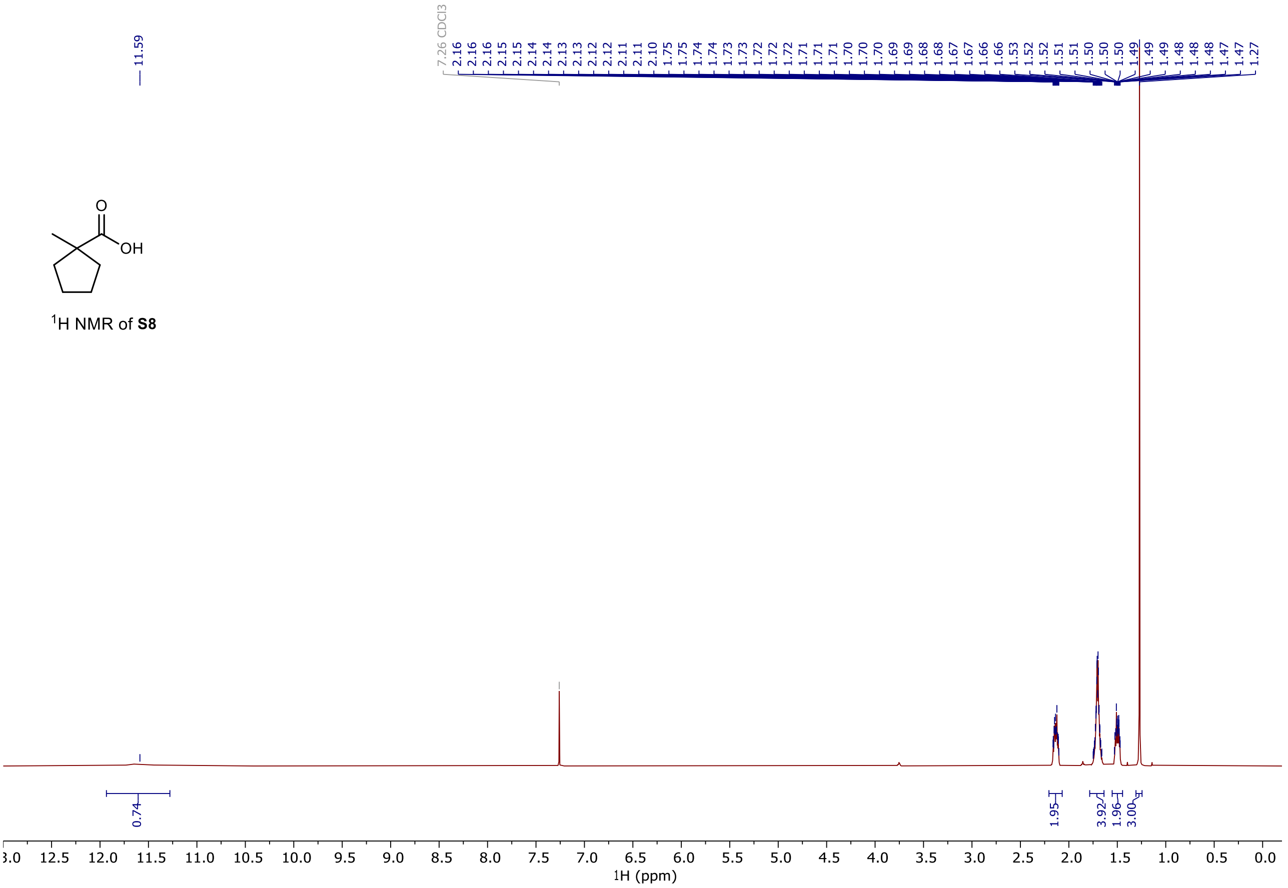

^{1}H NMR of **S8**

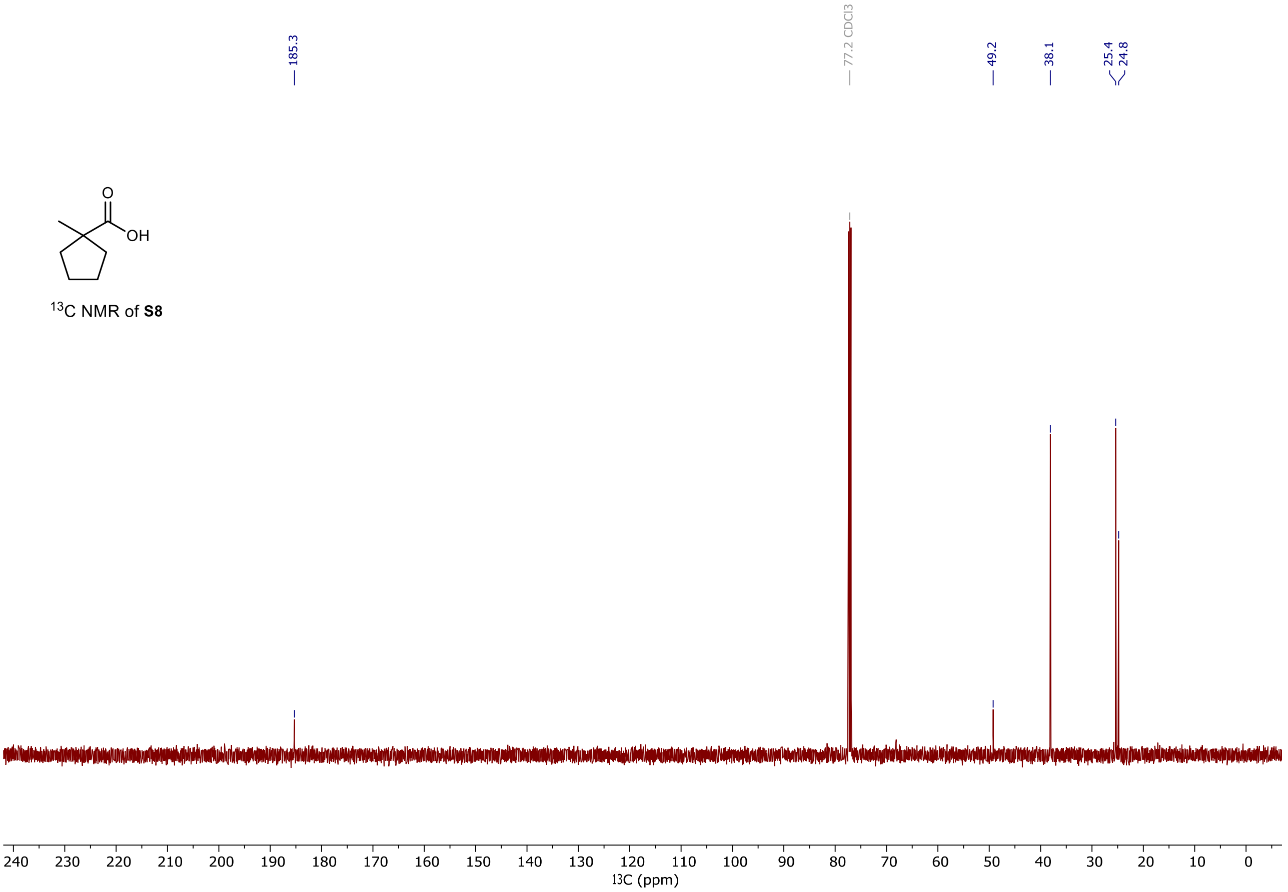

^{13}C NMR of **S8**

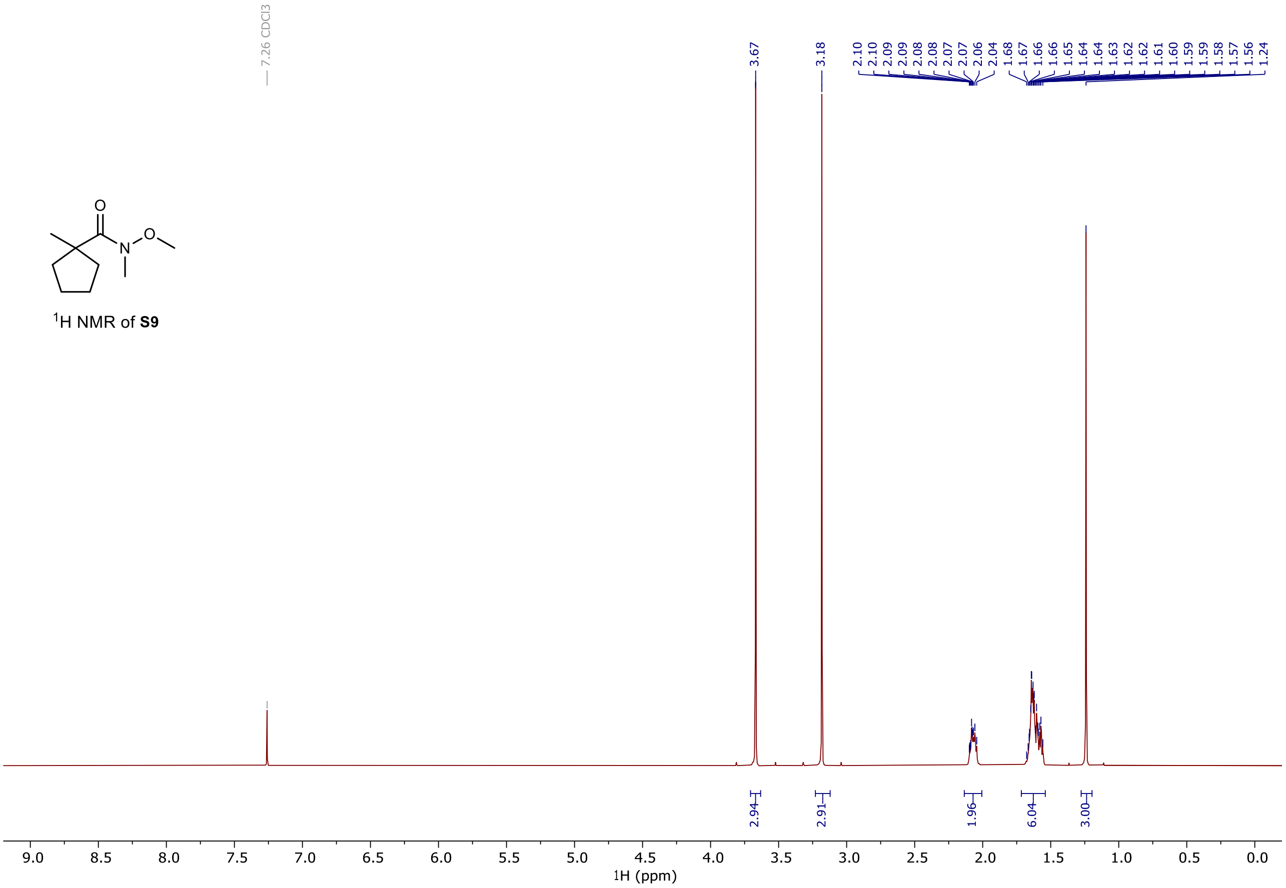

^{1}H NMR of **S9**

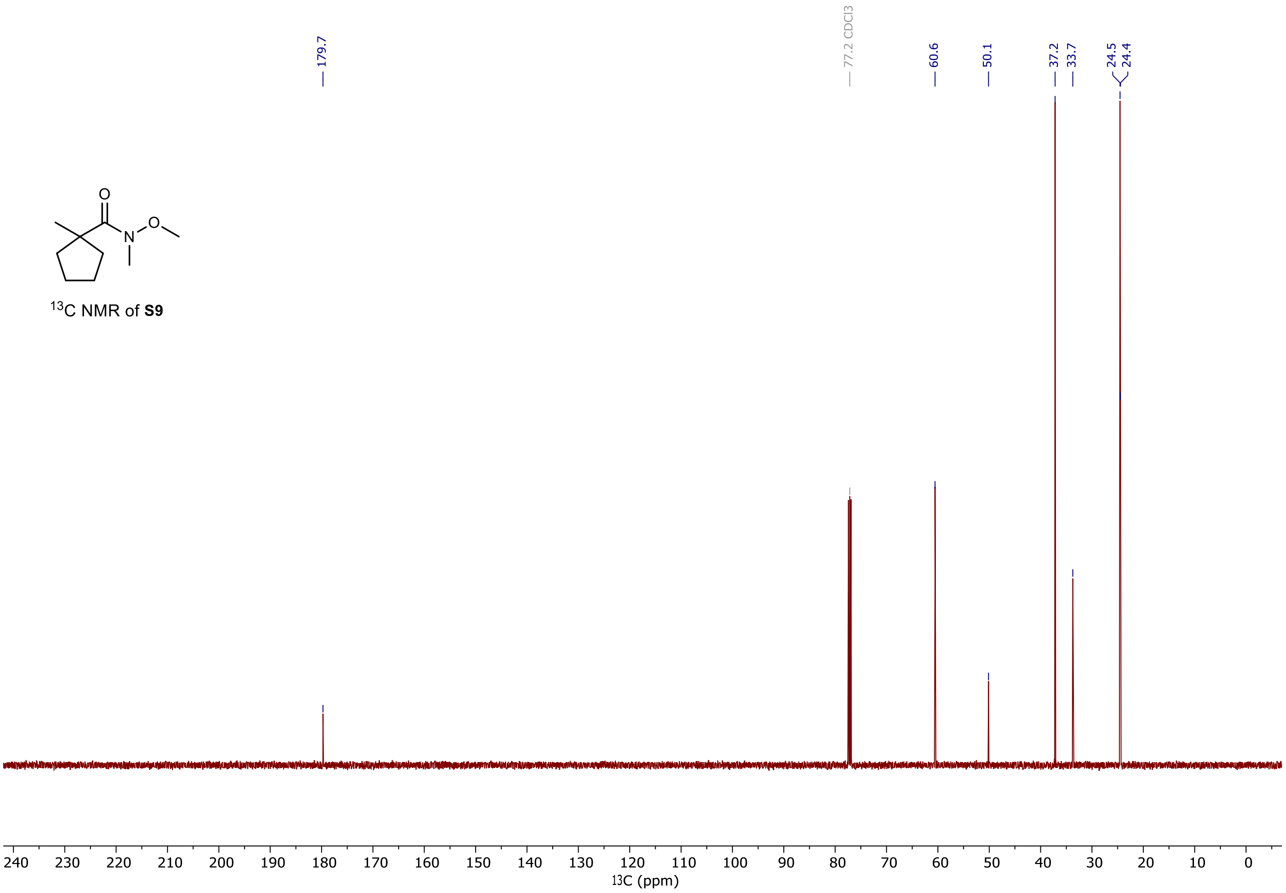

^{13}C NMR of **S9**

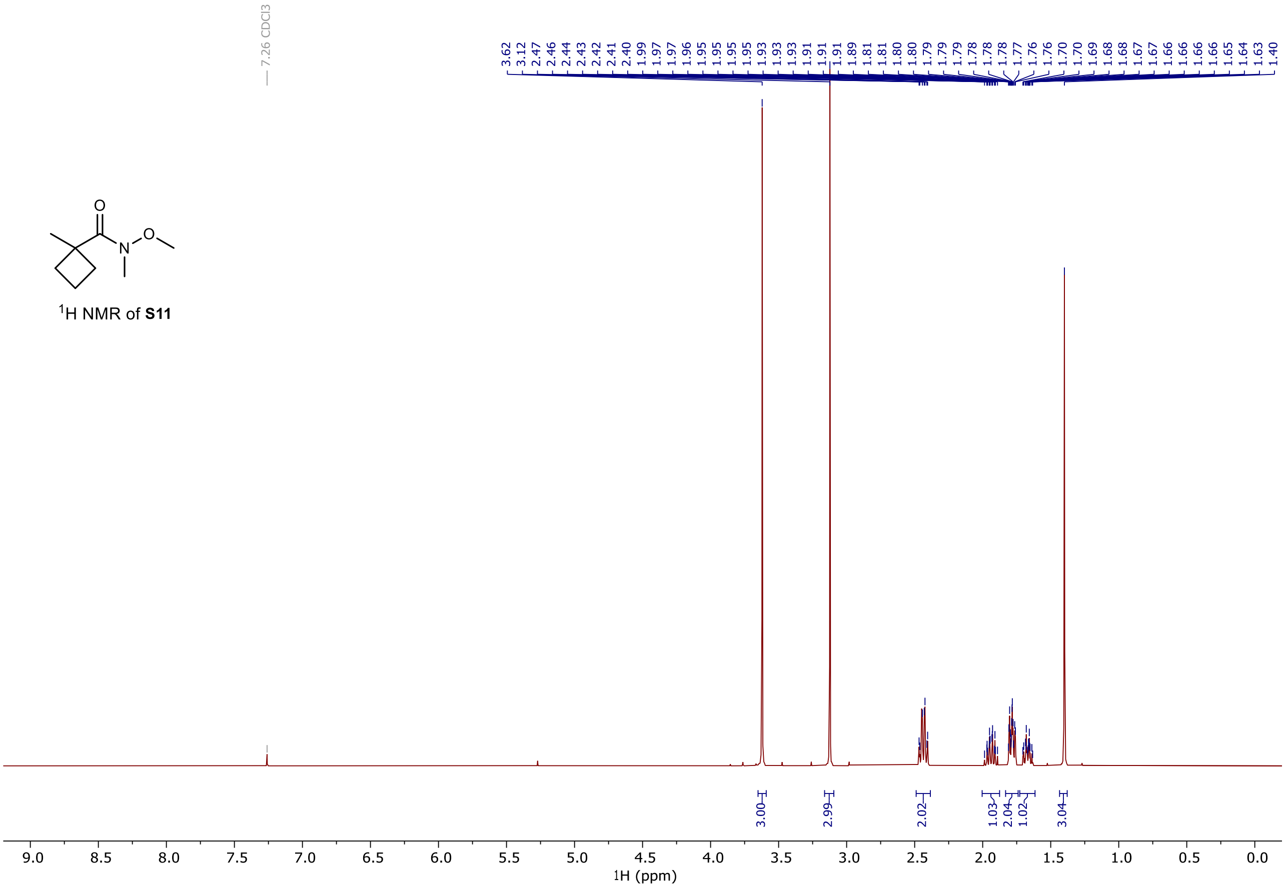
7.26 CDCl3
3.62
3.12
2.47
2.46
2.44
2.43
2.42
2.41
2.40
1.99
1.97
1.97
1.96
1.95
1.95
1.95
1.95
1.93
1.93
1.93
1.91
1.91
1.91
1.89
1.81
1.81
1.80
1.79
1.79
1.79
1.78
1.78
1.78
1.77
1.76
1.76
1.70
1.70
1.69
1.68
1.68
1.67
1.67
1.66
1.66
1.66
1.65
1.64
1.63
1.40
O
N
O
1H NMR of S11
3.00
2.99
2.02
1.03
2.04
1.02
3.04
1H (ppm)

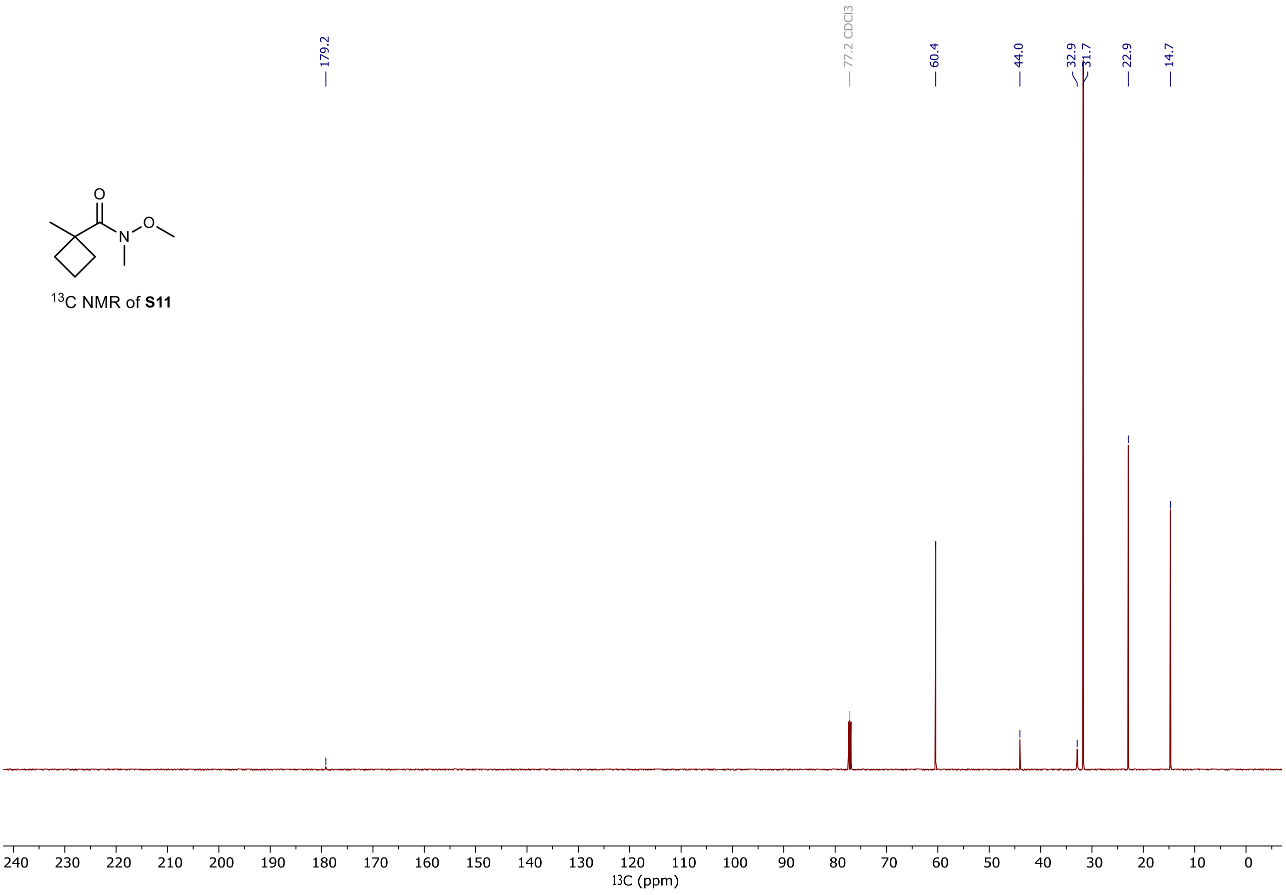
179.2
77.2 CDCl3
60.4
44.0
32.9
31.7
22.9
14.7
O
N
O
13C NMR of S11
13C (ppm)

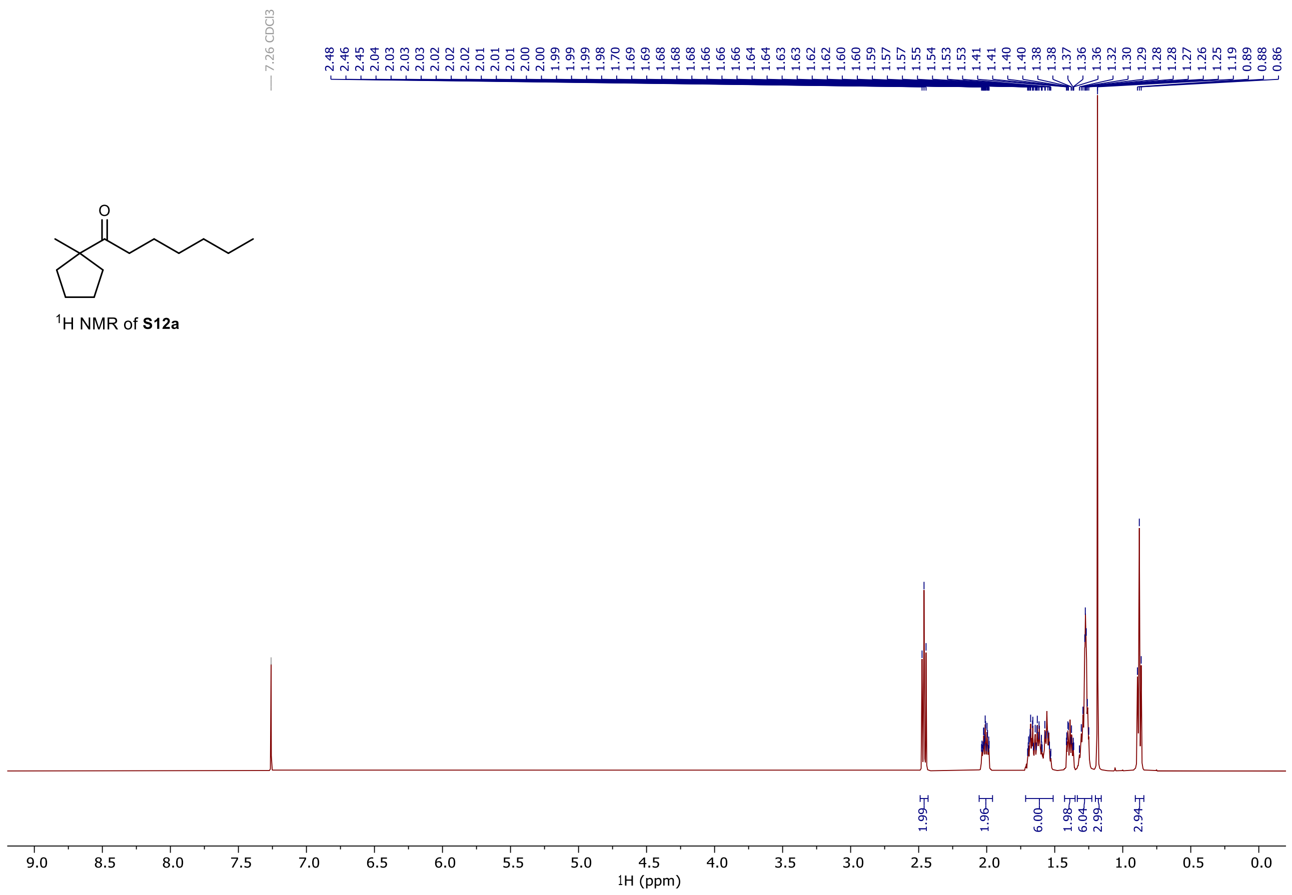
7.26 CDCl3
2.48 2.46 2.45 2.04 2.03 2.03 2.03 2.02 2.02 2.02 2.01 2.01 2.01 2.00 2.00 1.99 1.99 1.99 1.98 1.70 1.69 1.69 1.68 1.68 1.68 1.66 1.66 1.66 1.64 1.64 1.63 1.63 1.62 1.62 1.60 1.60 1.59 1.57 1.57 1.55 1.54 1.53 1.53 1.41 1.41 1.40 1.40 1.38 1.38 1.37 1.36 1.36 1.32 1.30 1.29 1.29 1.28 1.28 1.27 1.26 1.25 1.19 0.89 0.88 0.86
O
1H NMR of S12a
1.99
1.96
6.00
1.98
6.04
2.99
2.94
9.0 8.5 8.0 7.5 7.0 6.5 6.0 5.5 5.0 4.5 4.0 3.5 3.0 2.5 2.0 1.5 1.0 0.5 0.0
1H (ppm)

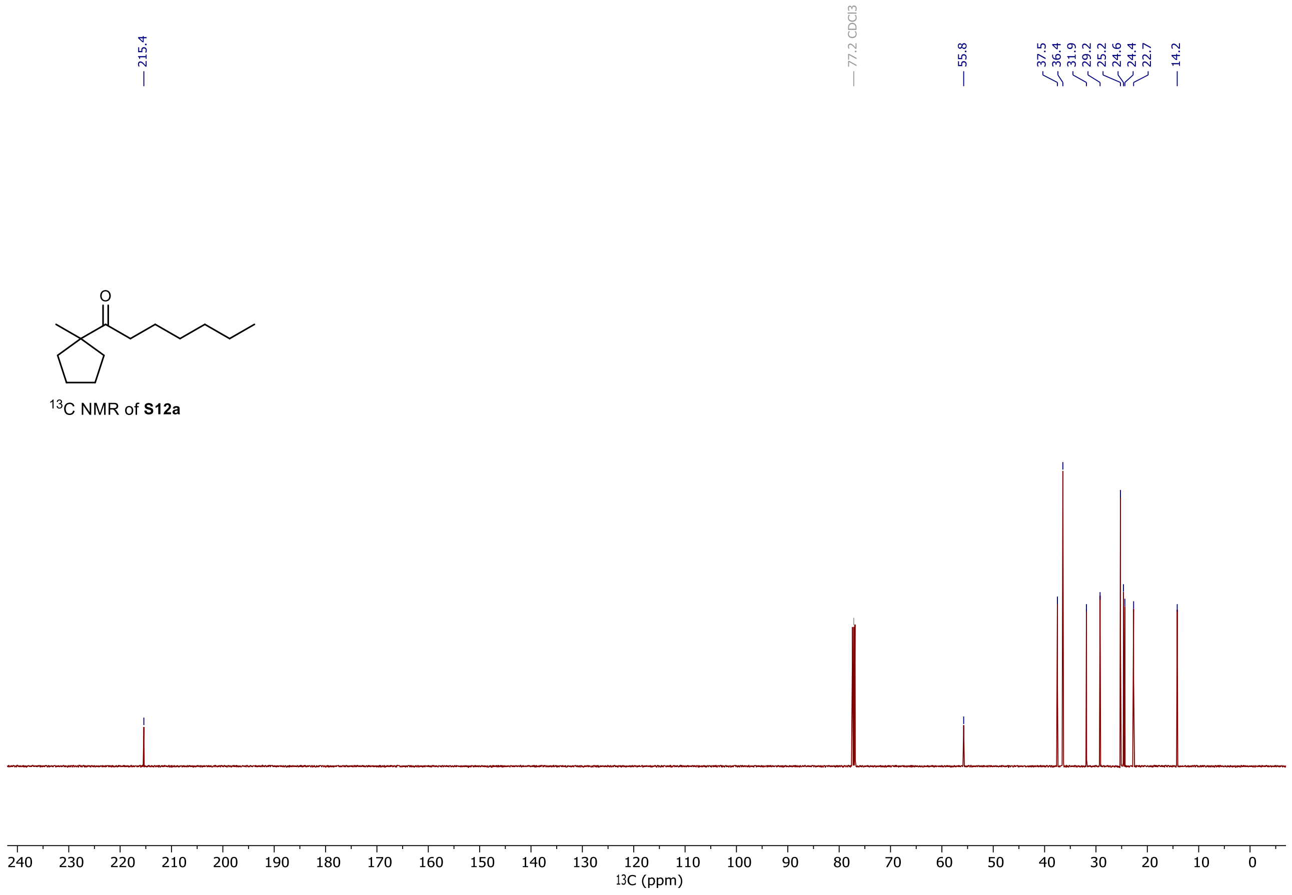
215.4
77.2 CDCl3
55.8
37.5 36.4 31.9 29.2 25.2 24.6 24.4 22.7
14.2
O
13C NMR of S12a
240 230 220 210 200 190 180 170 160 150 140 130 120 110 100 90 80 70 60 50 40 30 20 10 0
13C (ppm)

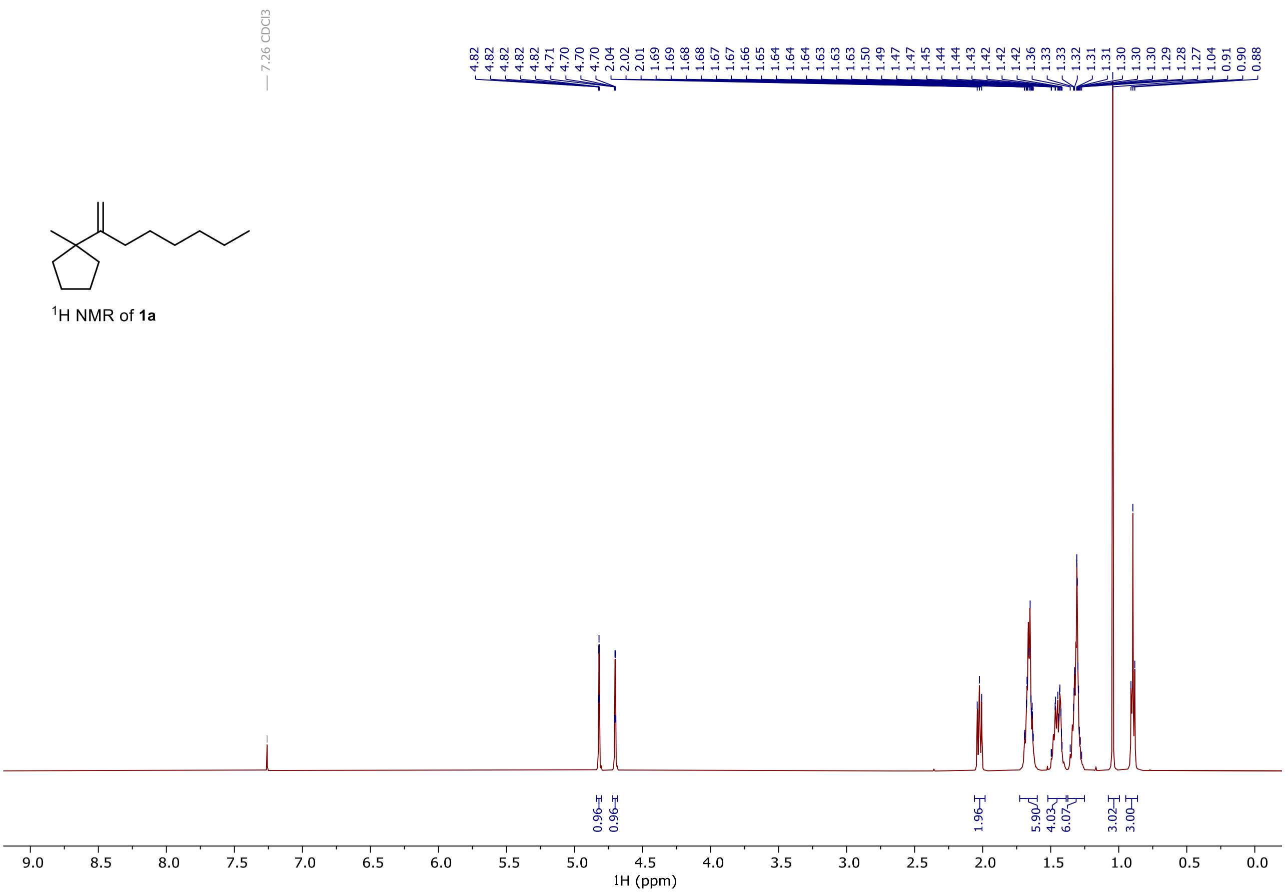

^{1}H NMR of **1a**

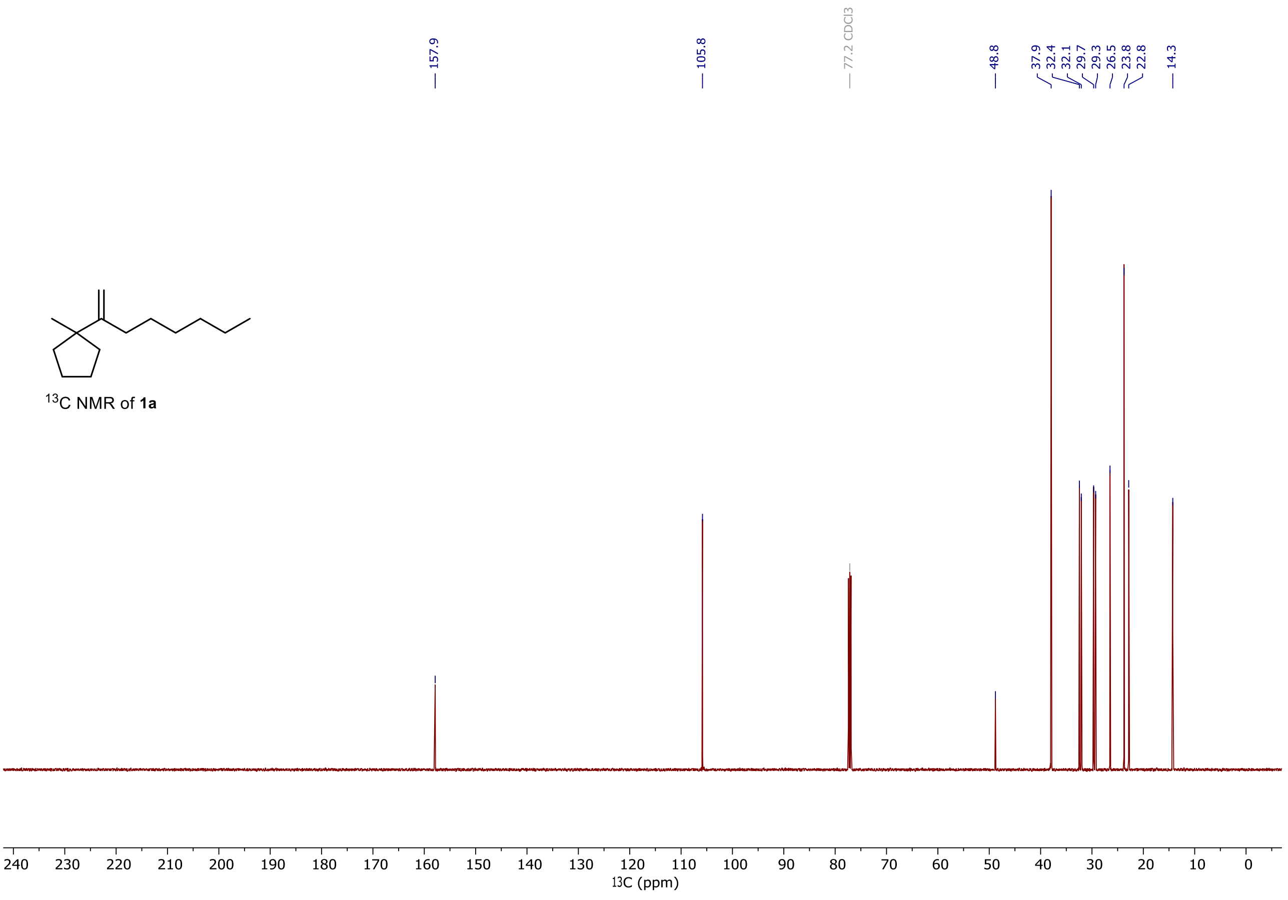

^{13}C NMR of **1a**

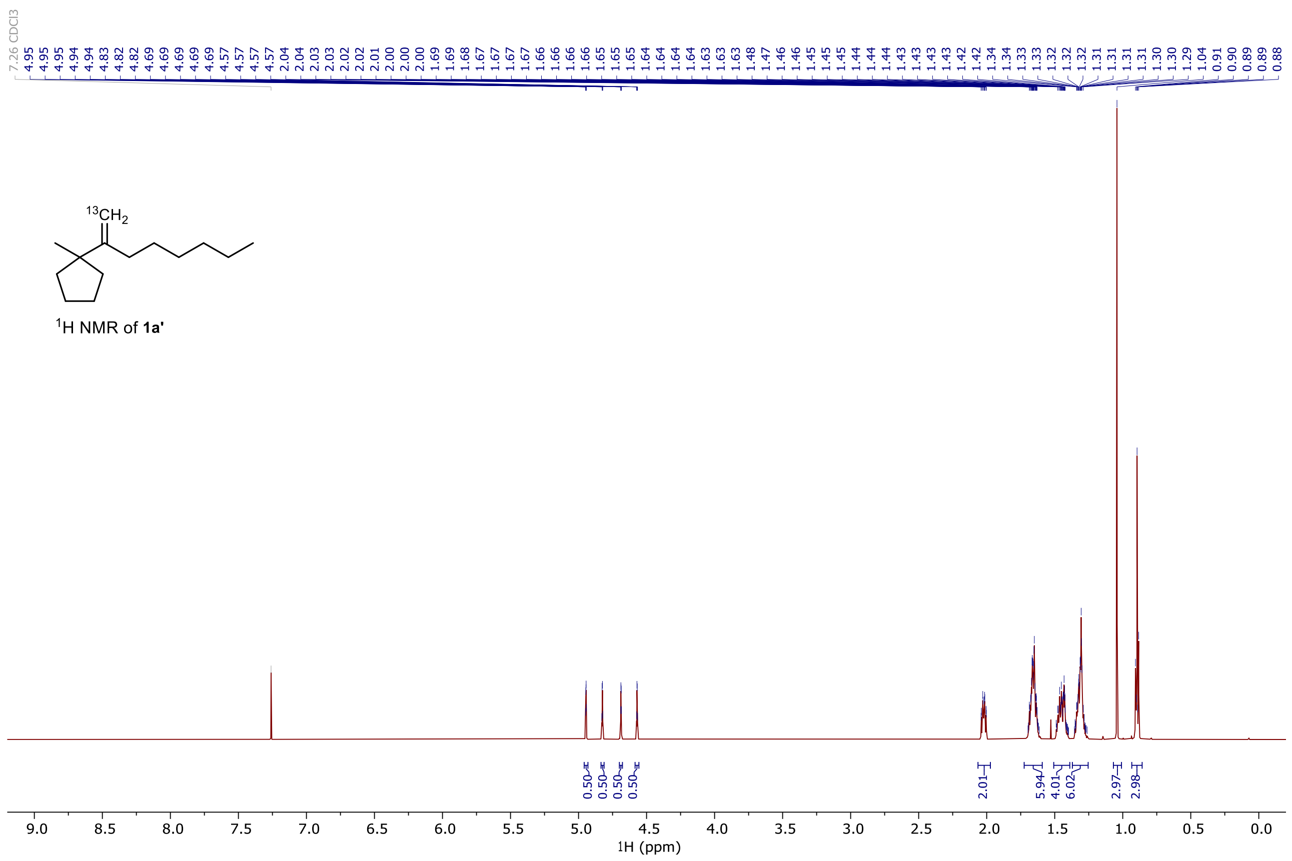

$^{13}CH_2$
^{1}H NMR of **1a'**
1H (ppm)

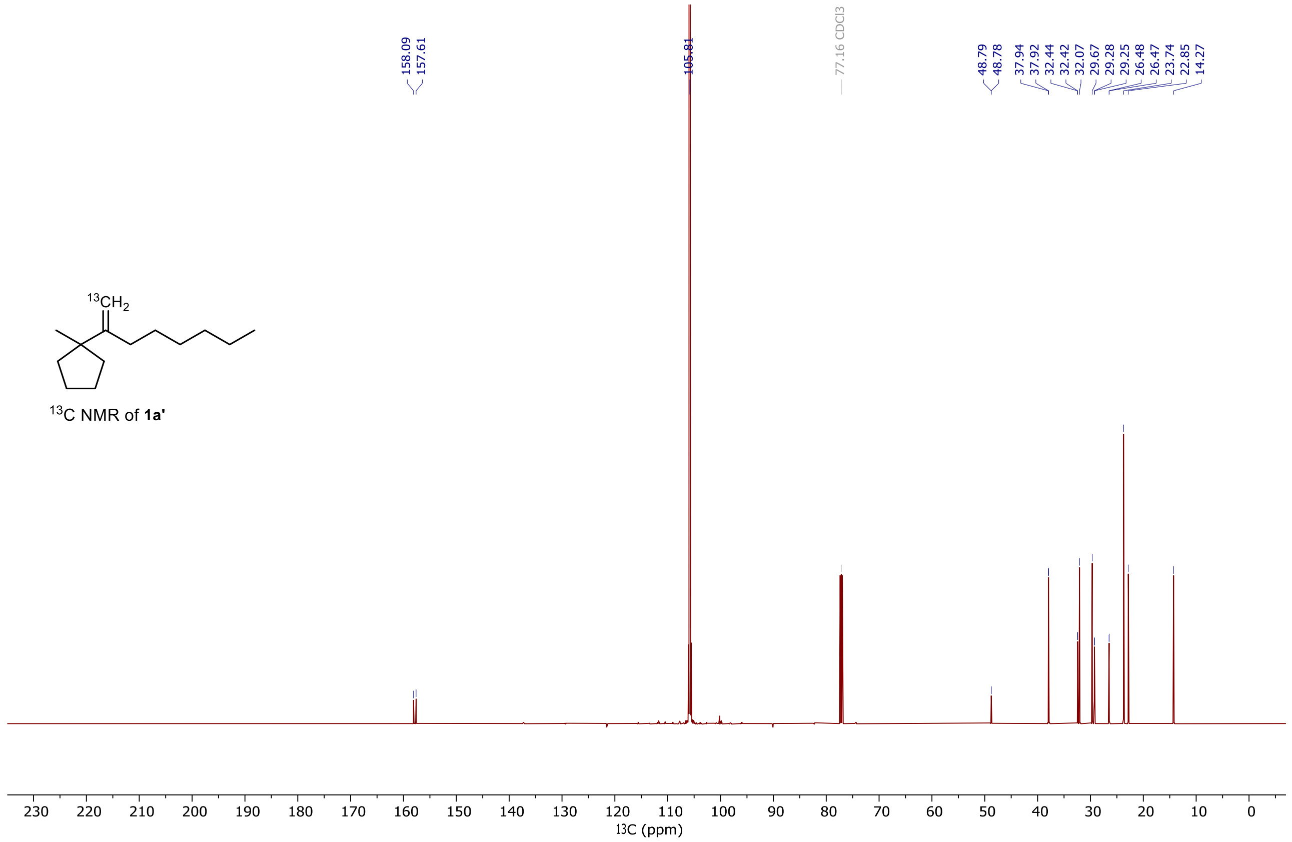

$^{13}CH_2$
^{13}C NMR of **1a'**
13C (ppm)

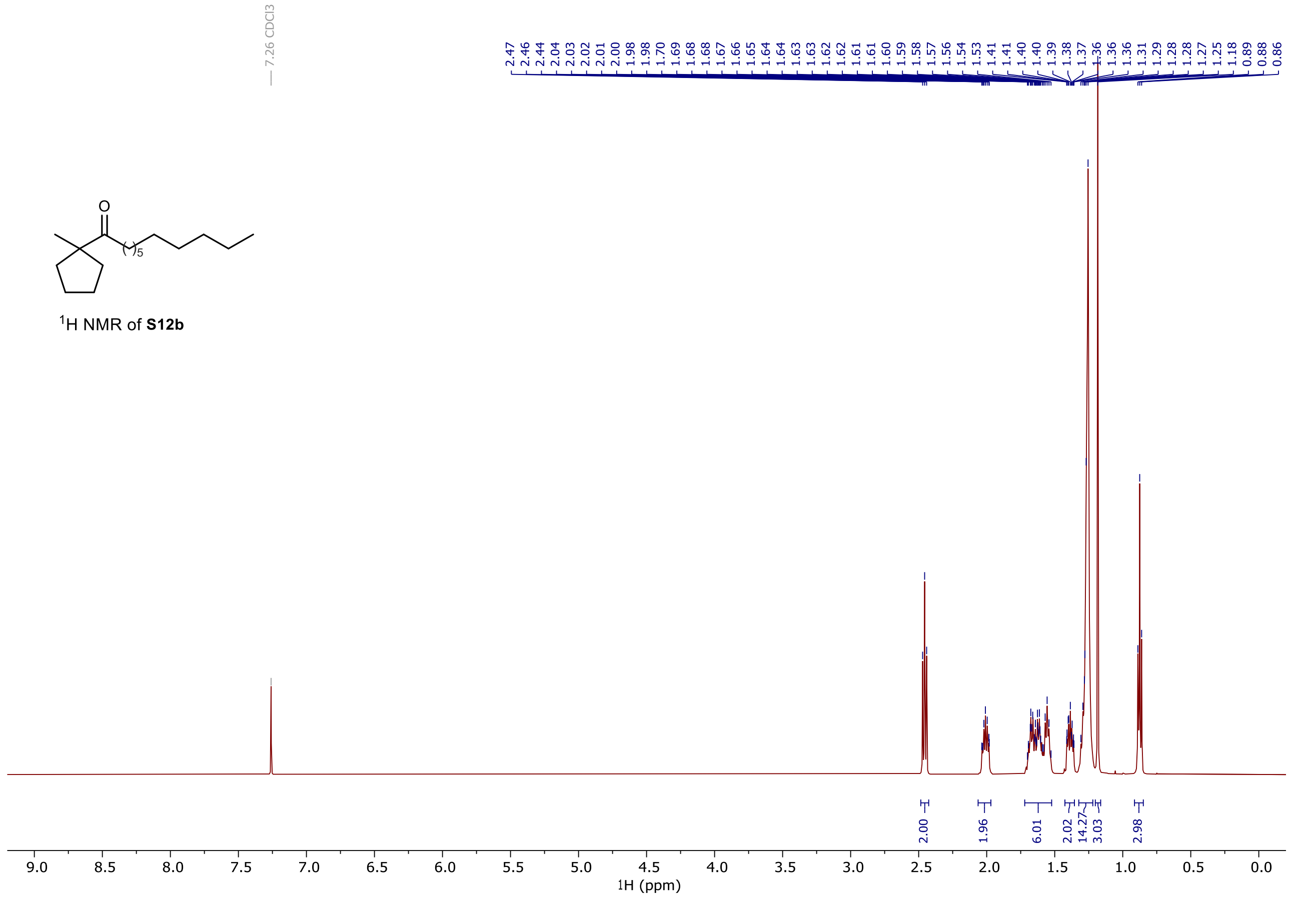
O
5
1H NMR of S12b
7.26 CDCl3
2.47 2.46 2.44 2.04 2.03 2.02 2.01 2.00 1.98 1.98 1.70 1.69 1.68 1.68 1.67 1.66 1.65 1.64 1.63 1.63 1.62 1.62 1.61 1.61 1.60 1.59 1.58 1.57 1.56 1.54 1.53 1.41 1.41 1.40 1.40 1.39 1.38 1.37 1.36 1.36 1.36 1.31 1.29 1.28 1.28 1.27 1.25 1.18 0.89 0.88 0.86
2.00 1.96 6.01 2.02 14.27 3.03 2.98
9.0 8.5 8.0 7.5 7.0 6.5 6.0 5.5 5.0 4.5 4.0 3.5 3.0 2.5 2.0 1.5 1.0 0.5 0.0
1H (ppm)

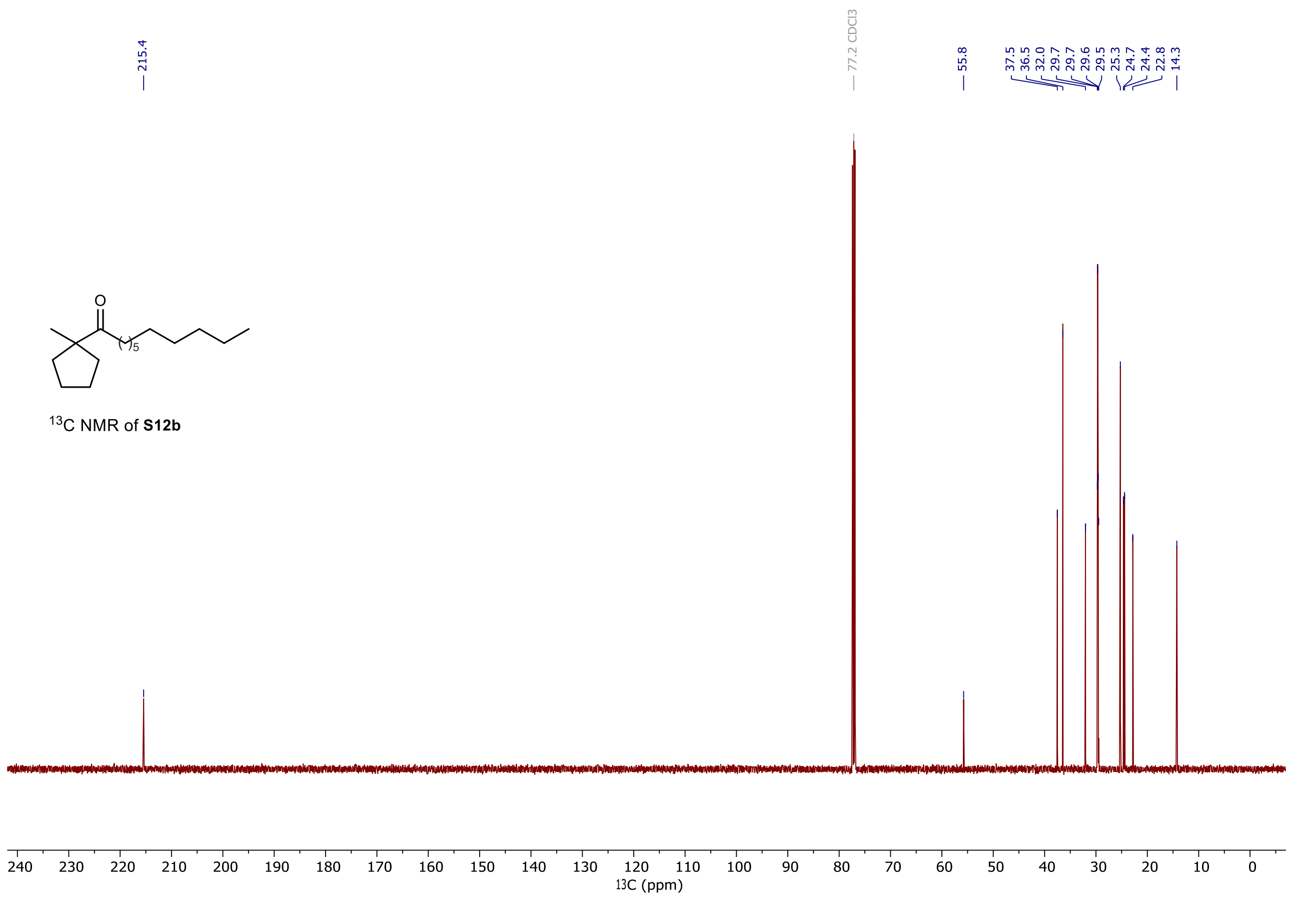
O
5
13C NMR of S12b
215.4
77.2 CDCl3
55.8
37.5 36.5 32.0 29.7 29.7 29.6 29.5 25.3 24.7 24.4 22.8 14.3
240 230 220 210 200 190 180 170 160 150 140 130 120 110 100 90 80 70 60 50 40 30 20 10 0
13C (ppm)

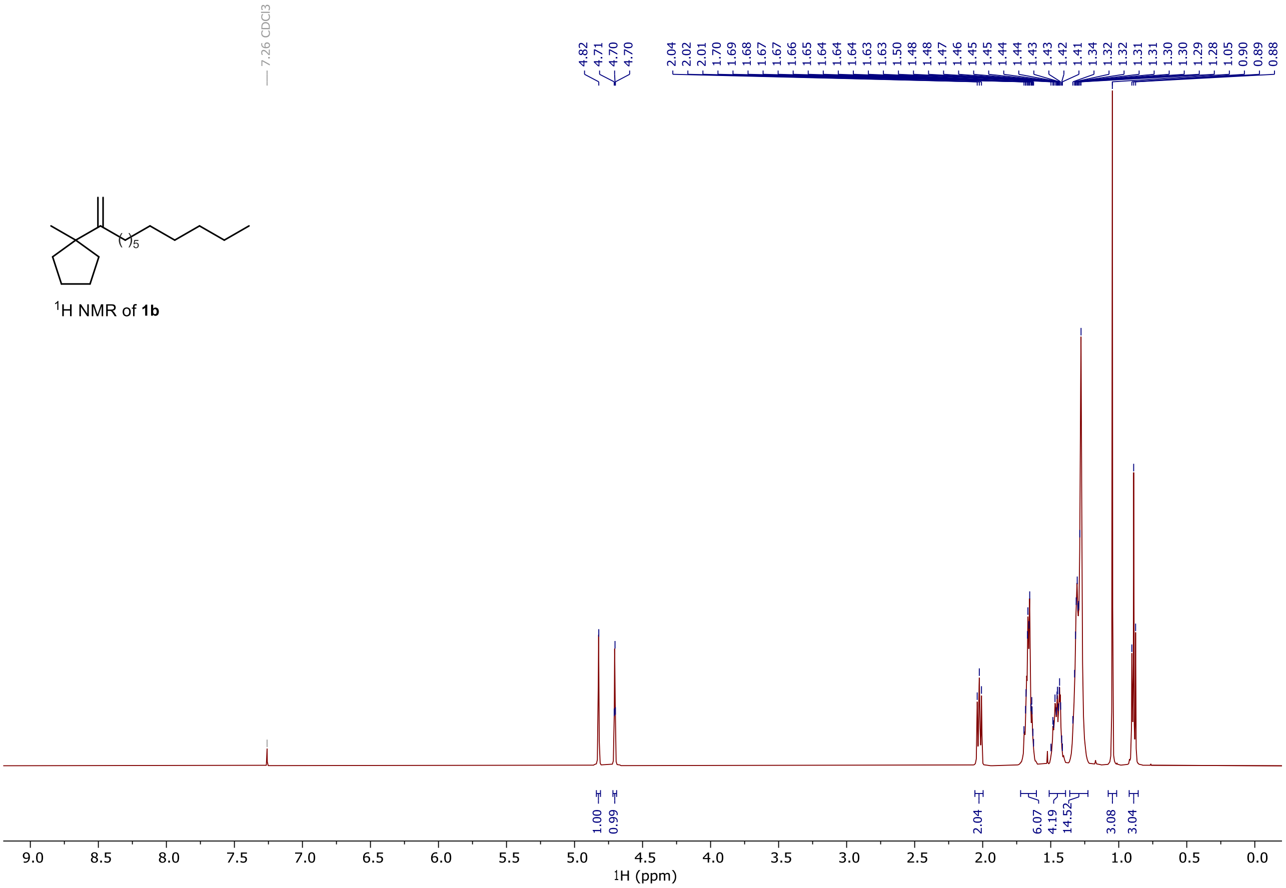

^{1}H NMR of **1b**

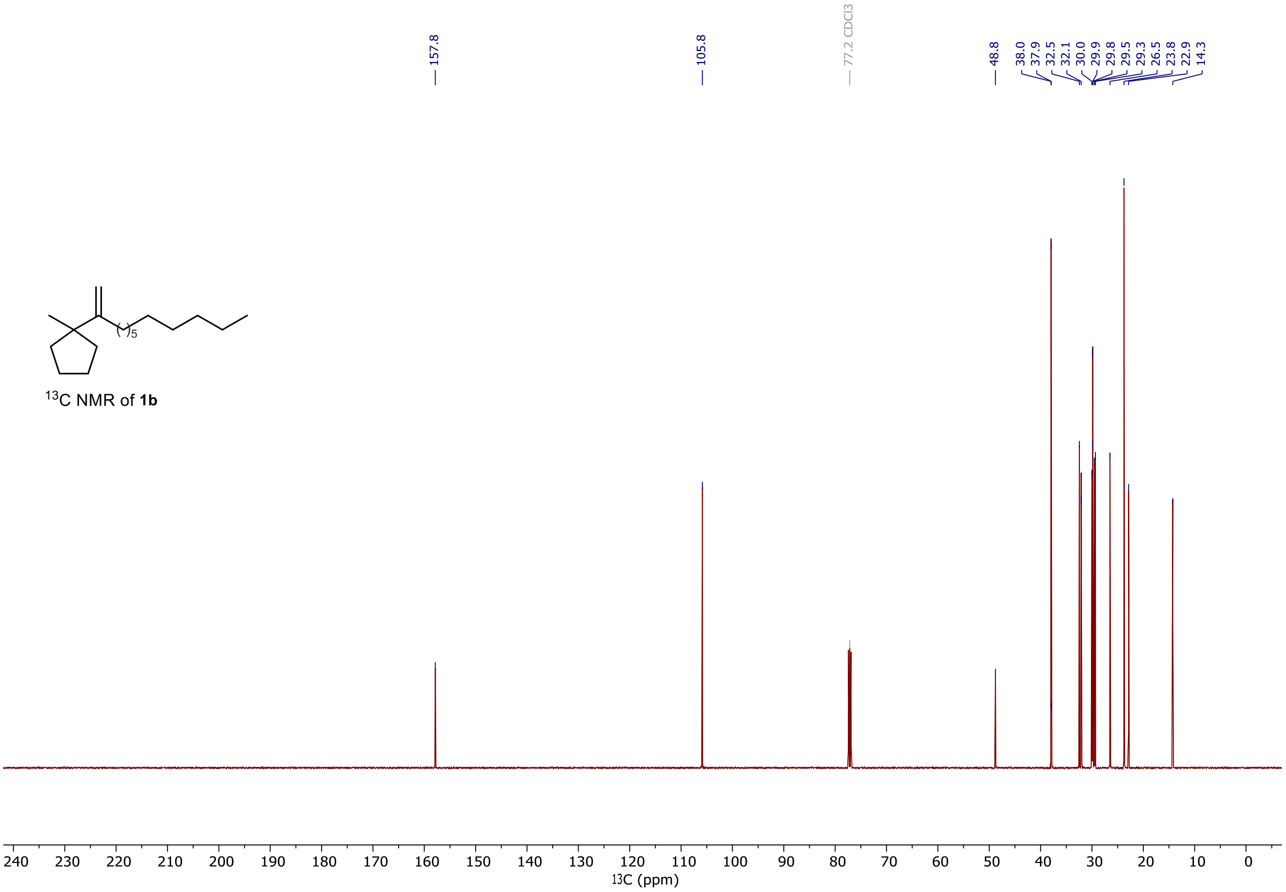

^{13}C NMR of **1b**

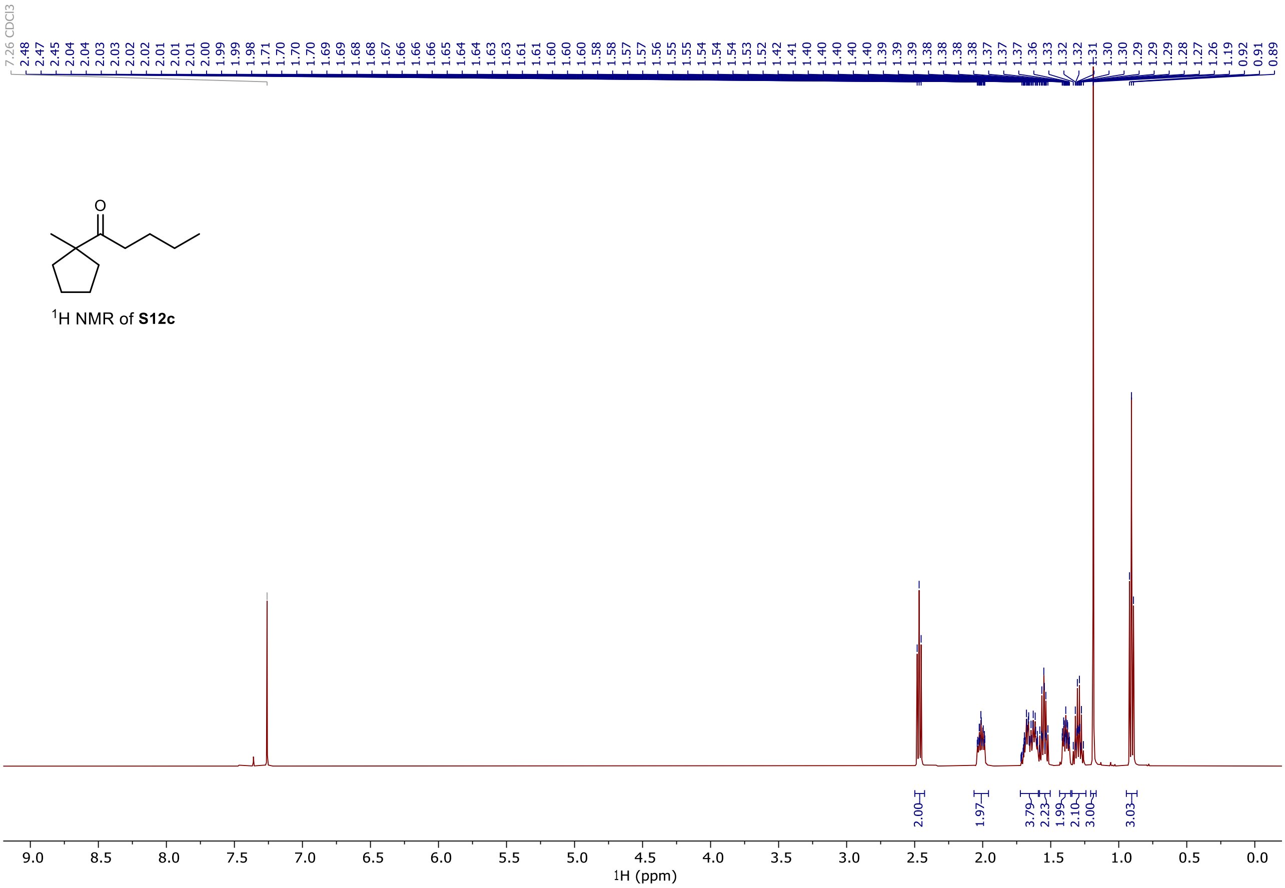

^{1}H NMR of **S12c**

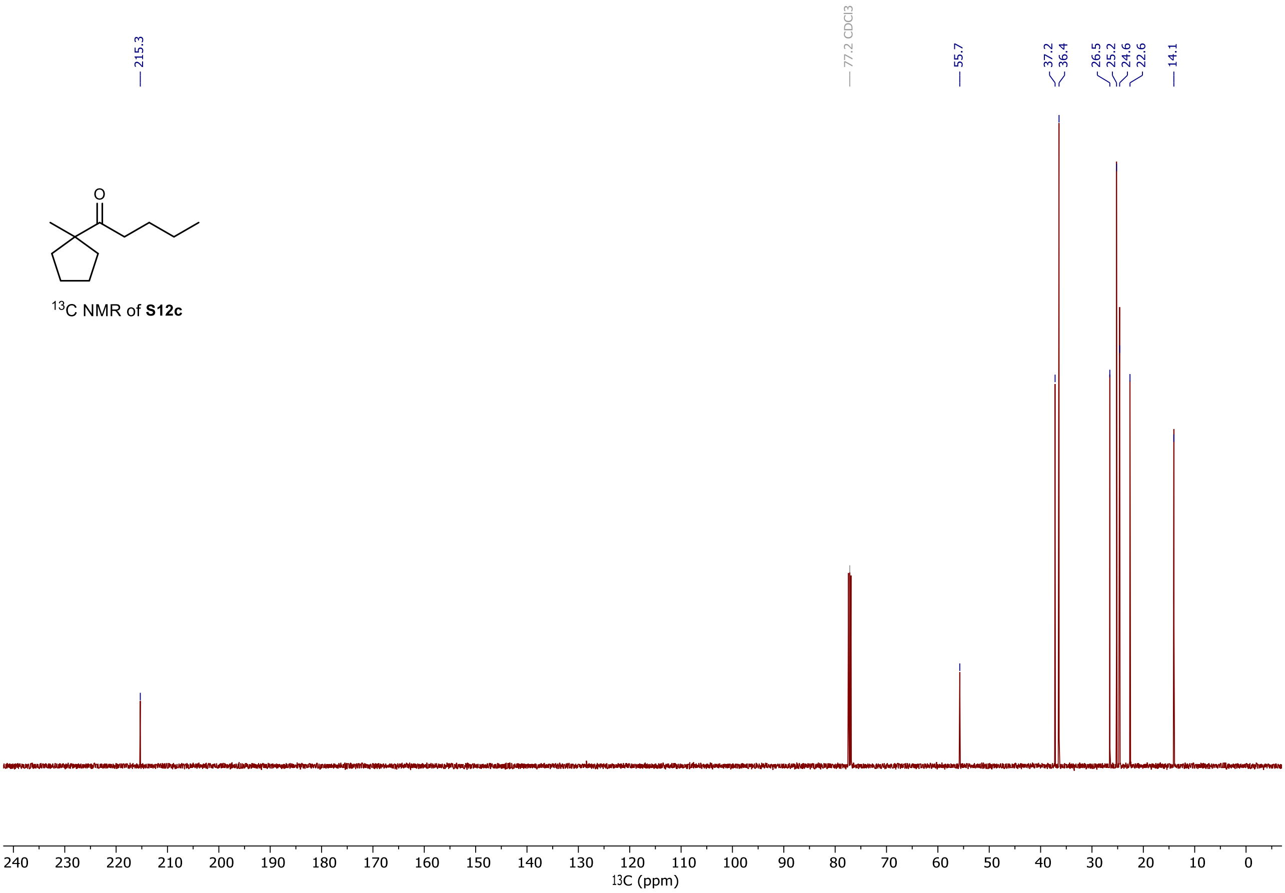

^{13}C NMR of **S12c**

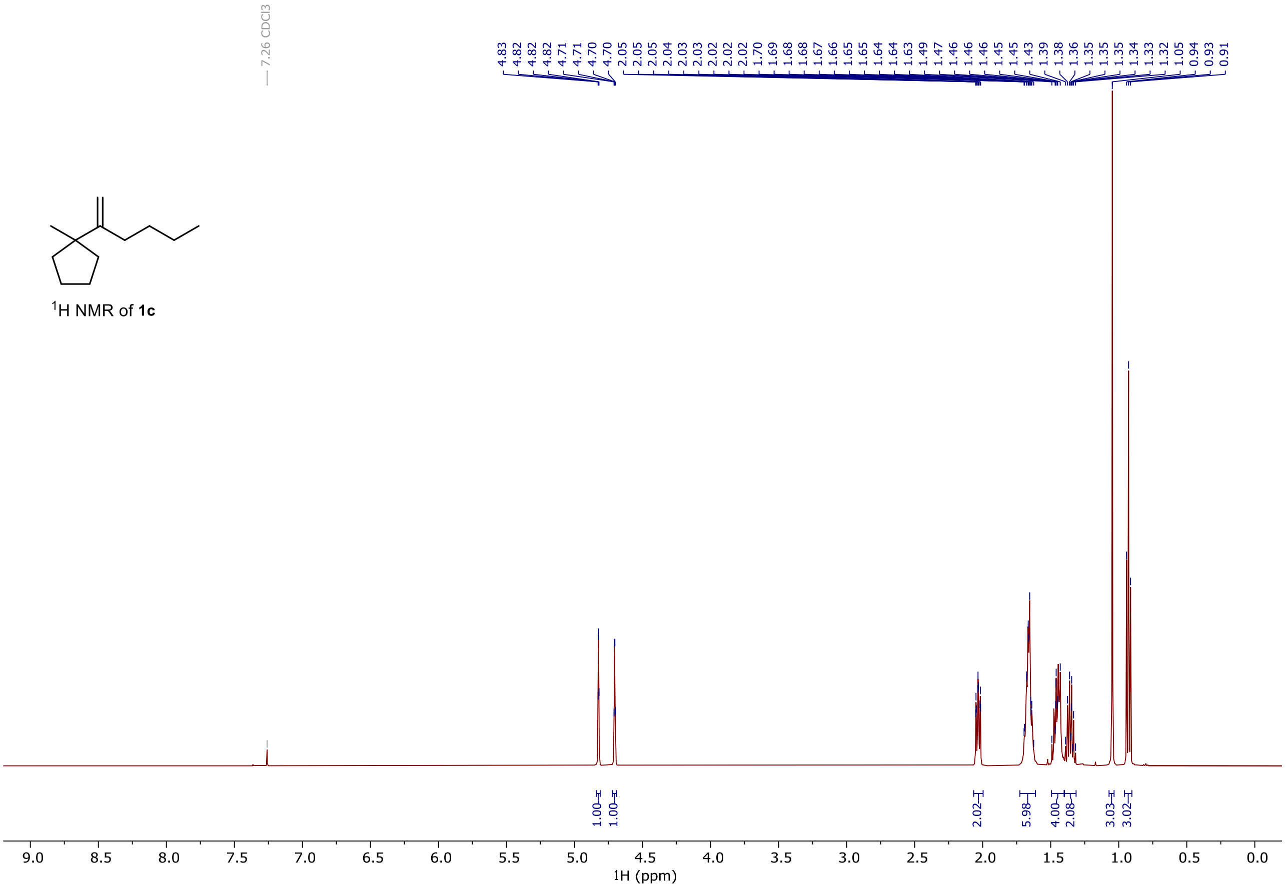

7.26 CDCl3
4.83
4.82
4.82
4.82
4.71
4.71
4.70
4.70
2.05
2.05
2.05
2.04
2.03
2.03
2.02
2.02
2.02
1.70
1.69
1.68
1.68
1.67
1.66
1.65
1.65
1.65
1.64
1.64
1.63
1.49
1.47
1.46
1.46
1.46
1.45
1.45
1.43
1.39
1.38
1.36
1.35
1.35
1.35
1.34
1.33
1.32
1.05
0.94
0.93
0.91
1H NMR of 1c
1.00
1.00
2.02
5.98
4.00
2.08
3.03
3.02
9.0
8.5
8.0
7.5
7.0
6.5
6.0
5.5
5.0
4.5
4.0
3.5
3.0
2.5
2.0
1.5
1.0
0.5
0.0
1H (ppm)

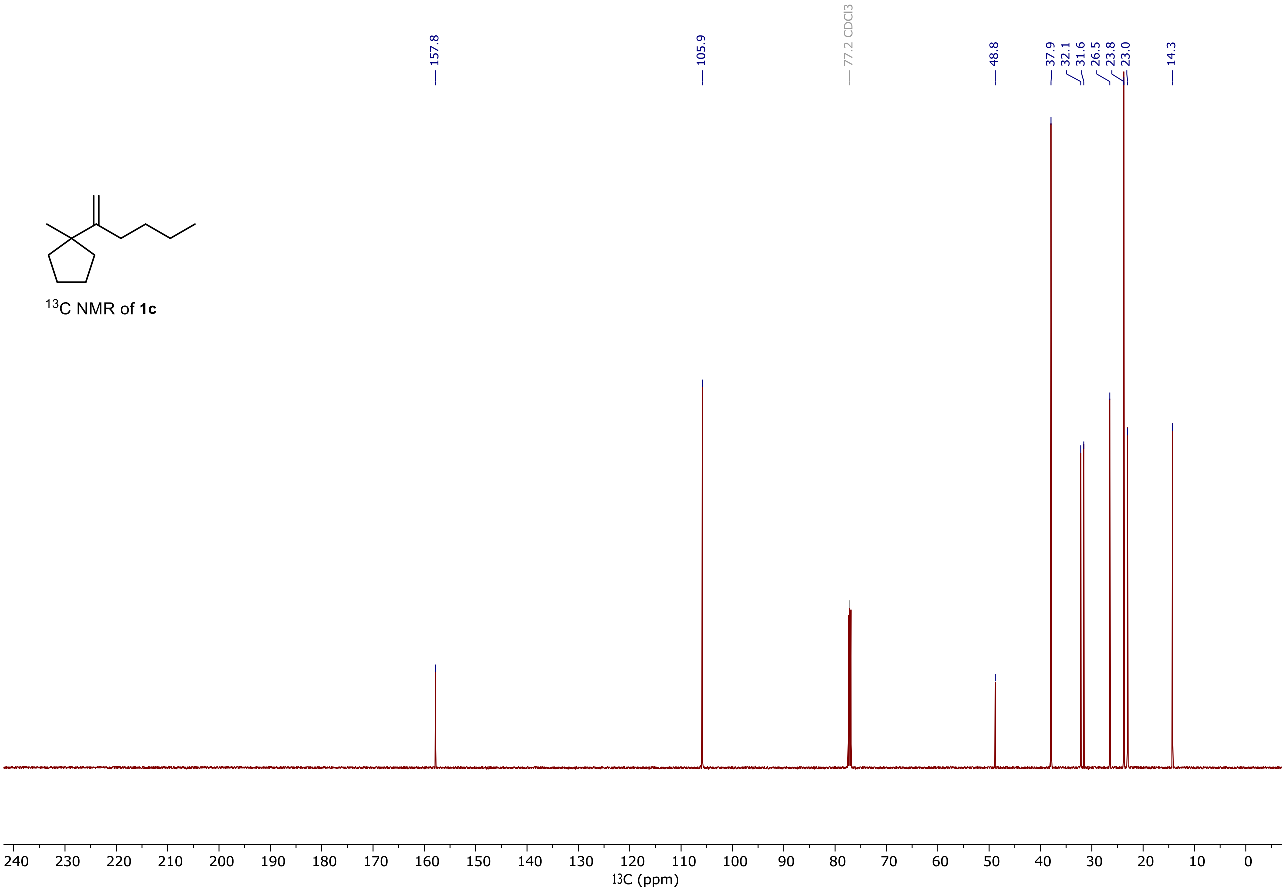

157.8
105.9
77.2 CDCl3
48.8
37.9
32.1
31.6
26.5
23.8
23.0
14.3
13C NMR of 1c
240
230
220
210
200
190
180
170
160
150
140
130
120
110
100
90
80
70
60
50
40
30
20
10
0
13C (ppm)

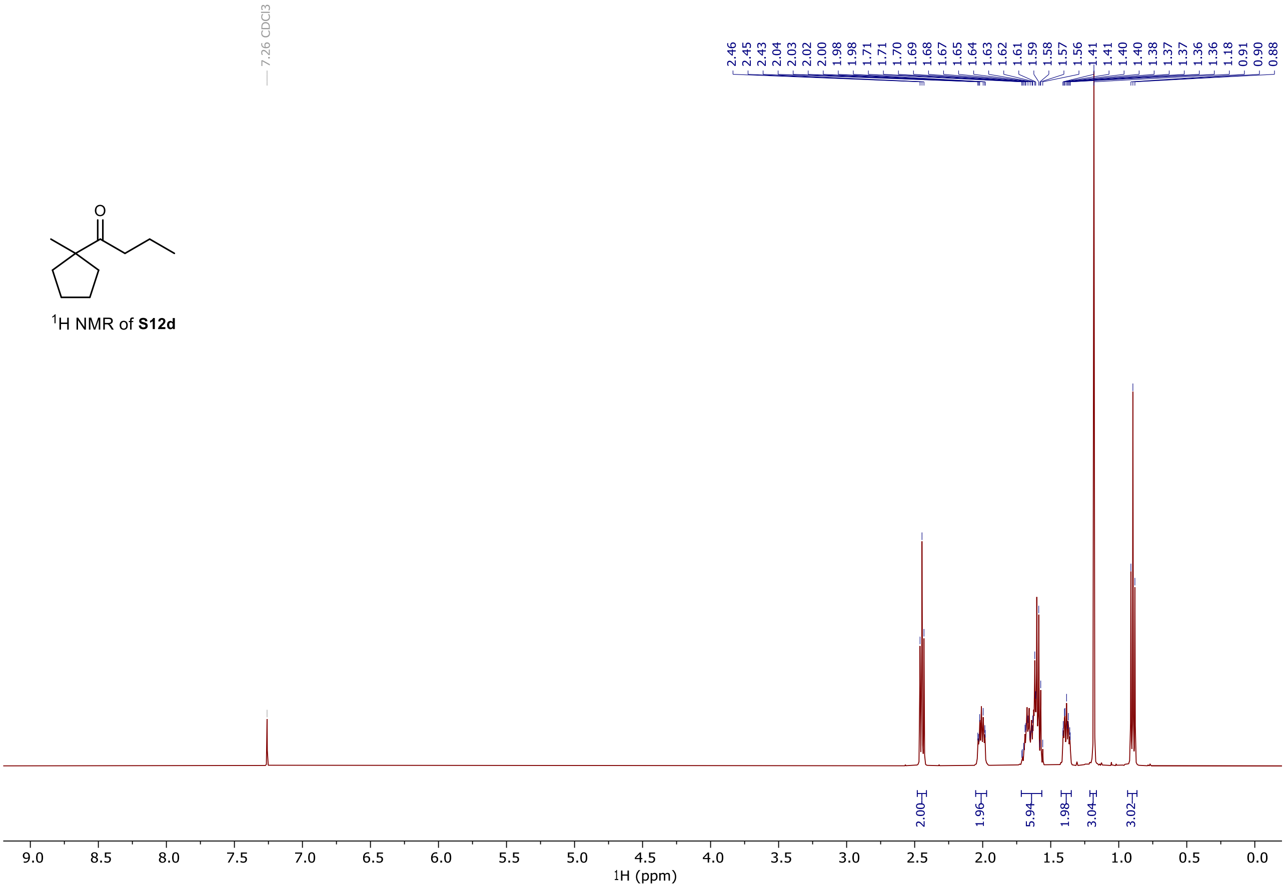

^{1}H NMR of **S12d**

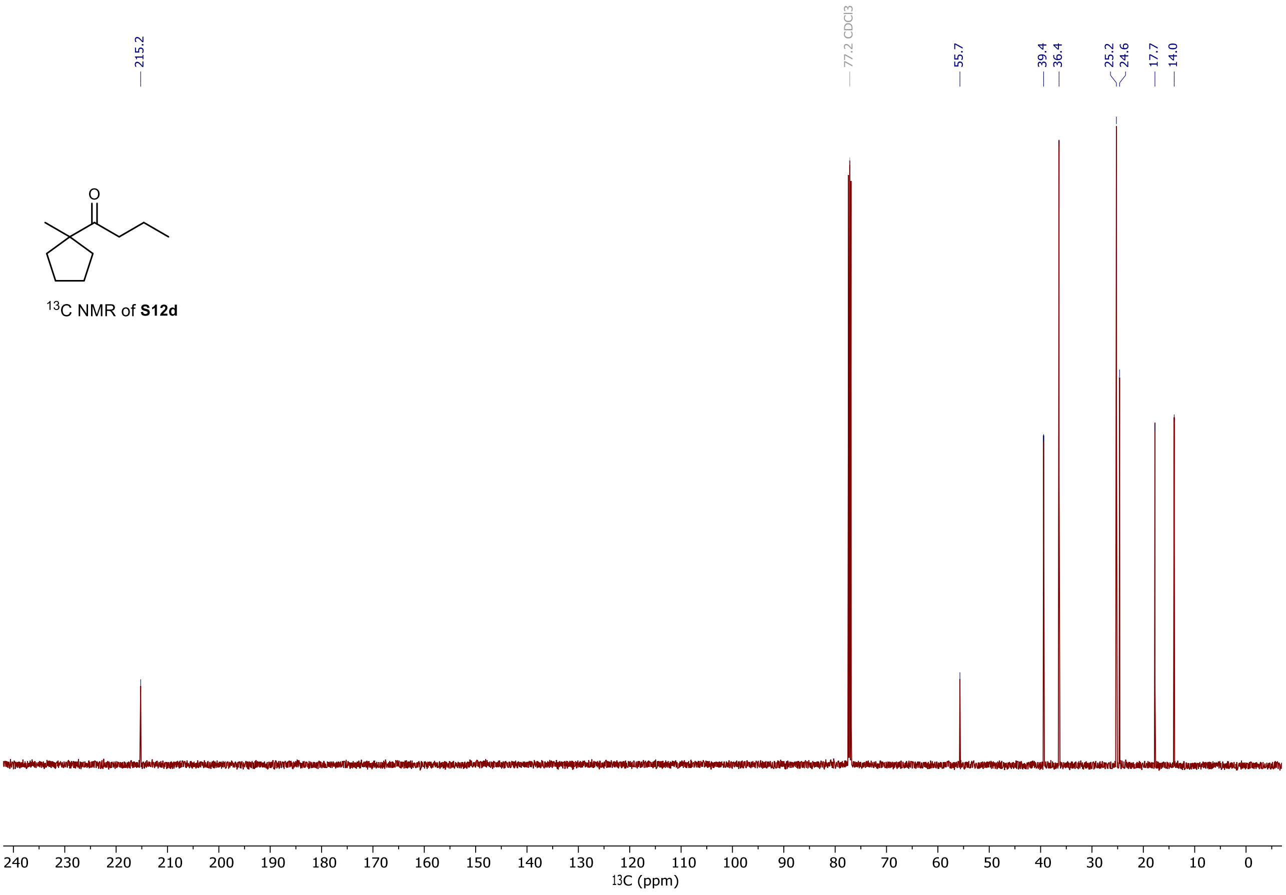

^{13}C NMR of **S12d**

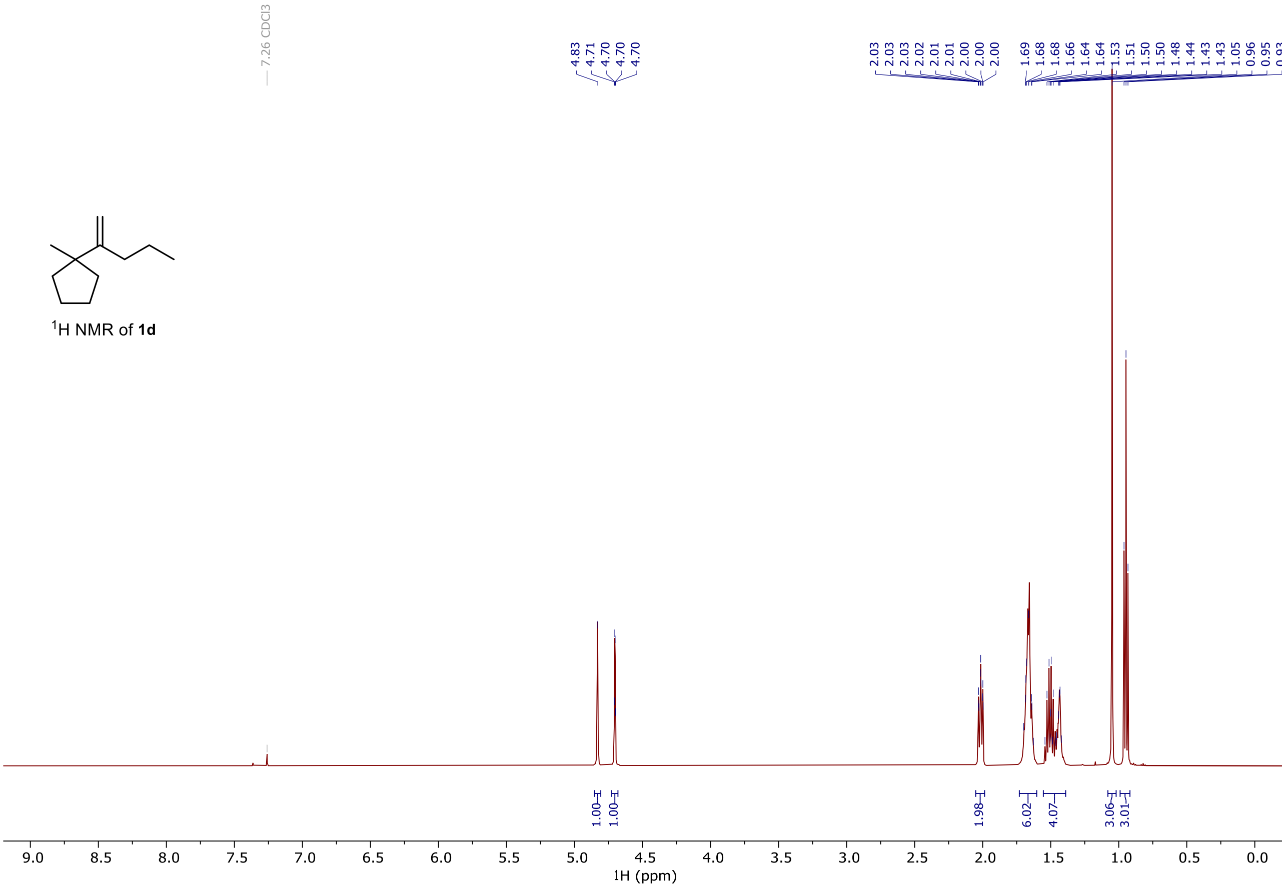

^{1}H NMR of **1d**

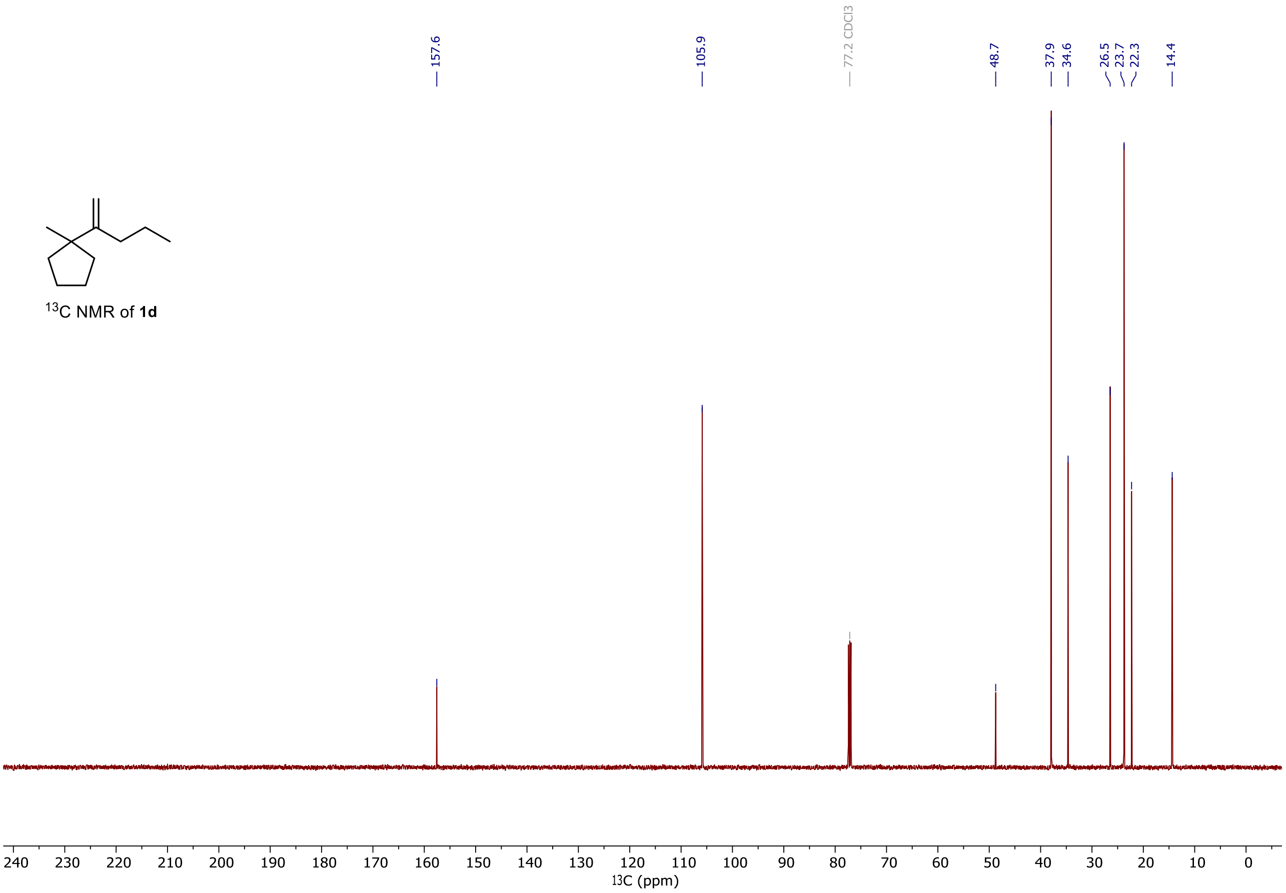

^{13}C NMR of **1d**

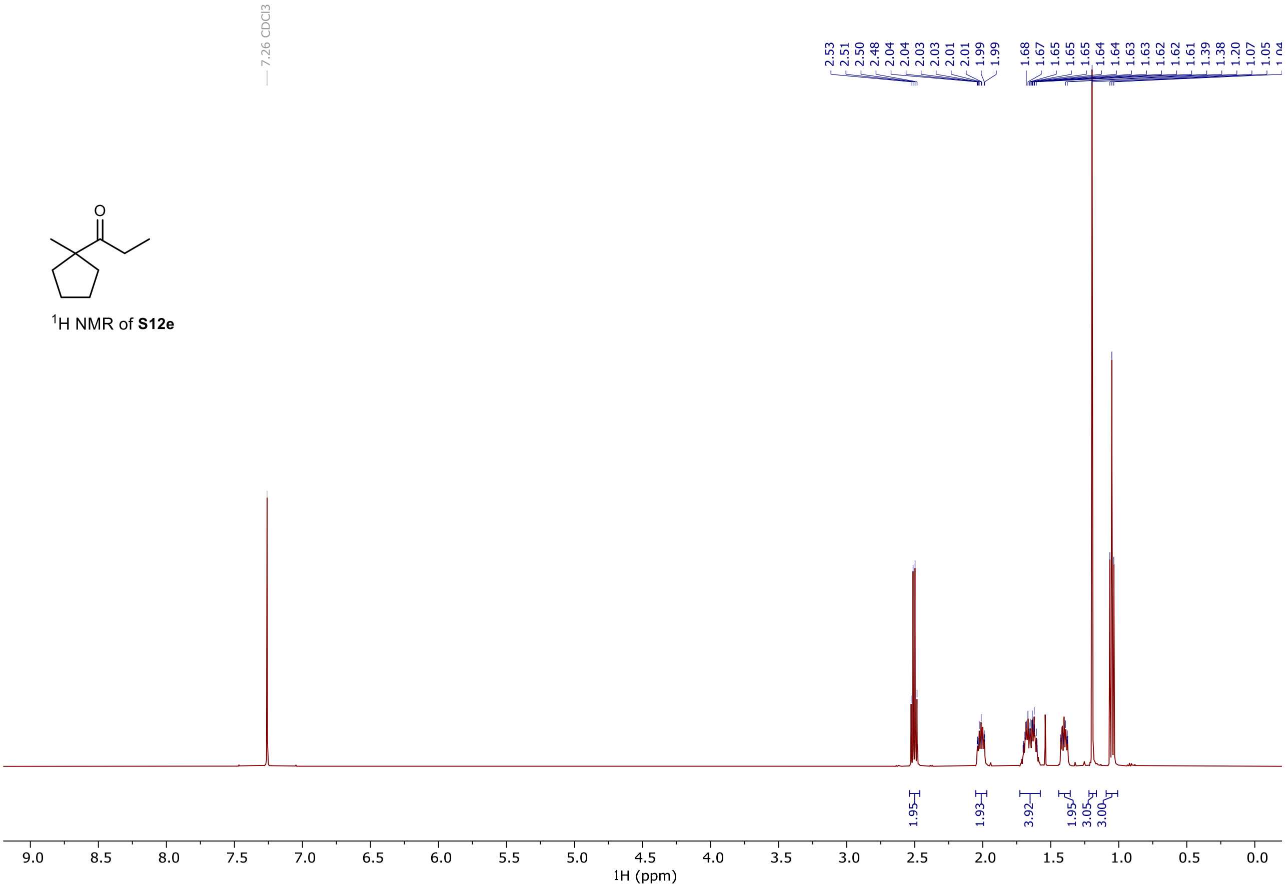

^{1}H NMR of **S12e**

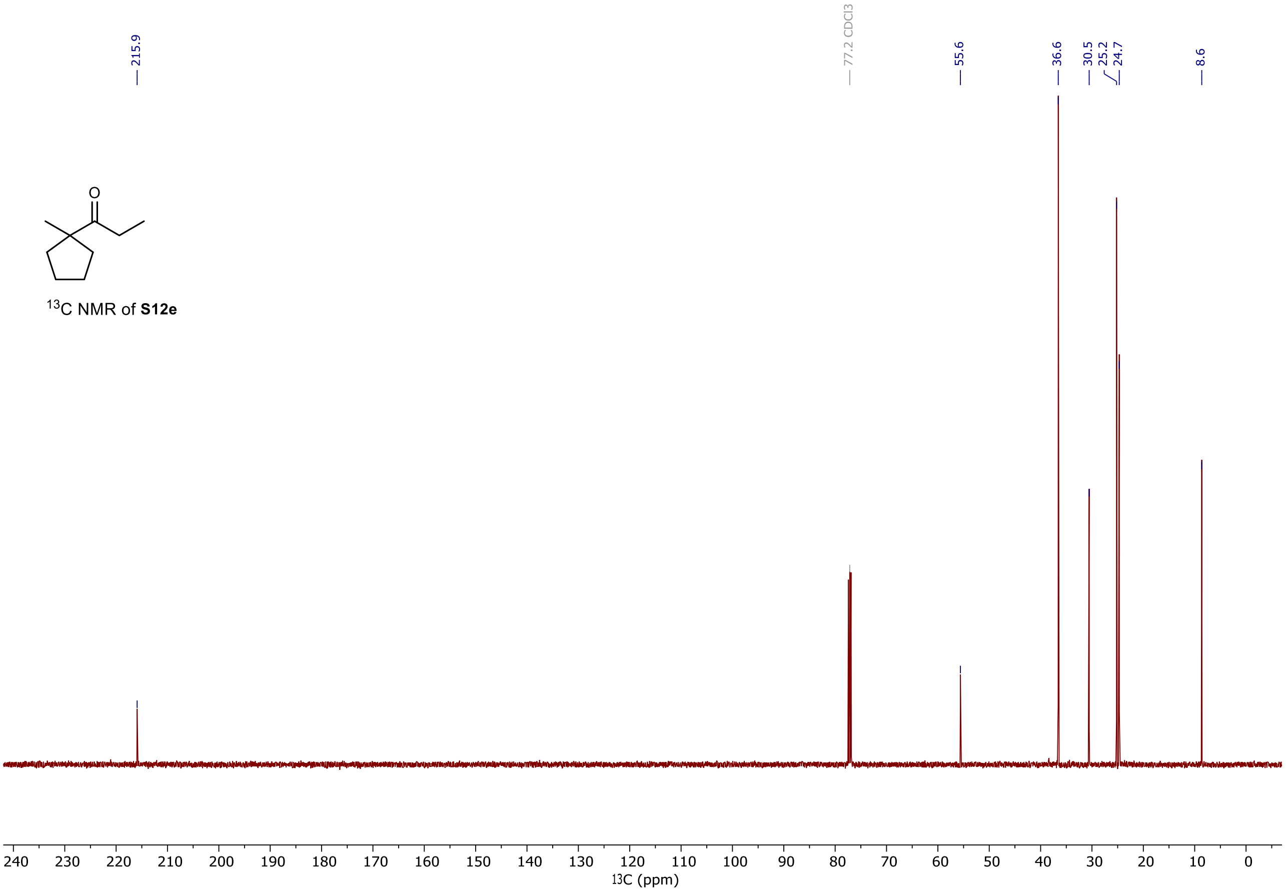

^{13}C NMR of **S12e**

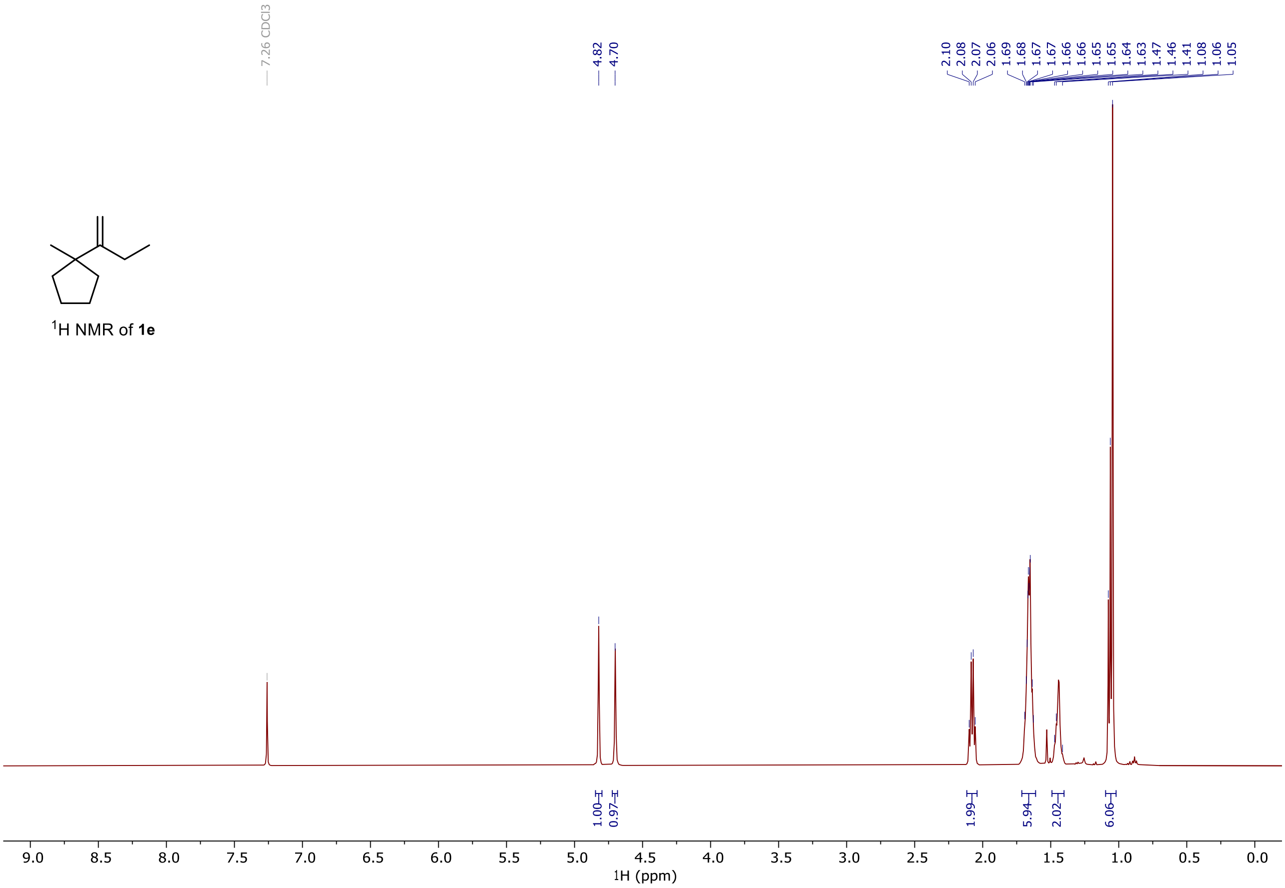

^{1}H NMR of **1e**

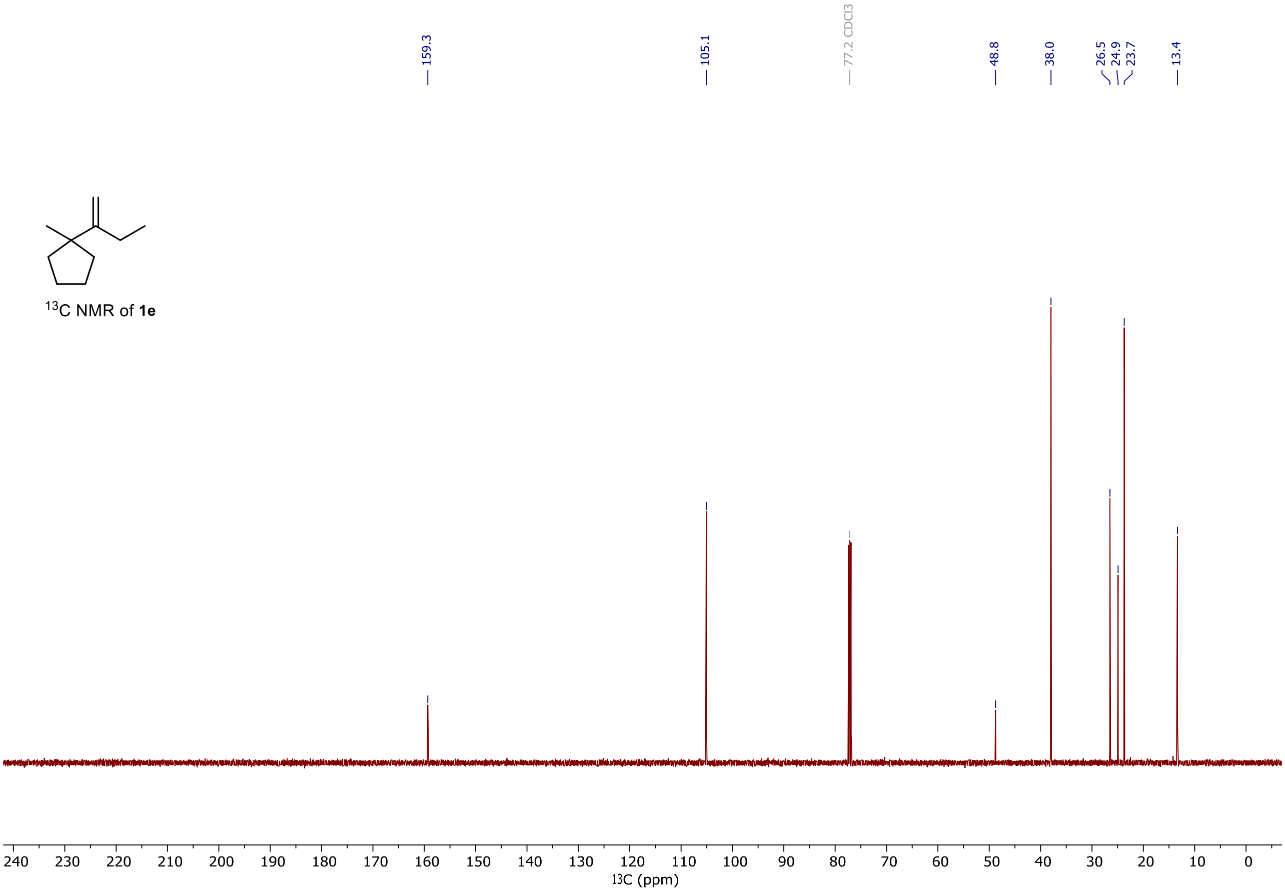

^{13}C NMR of **1e**

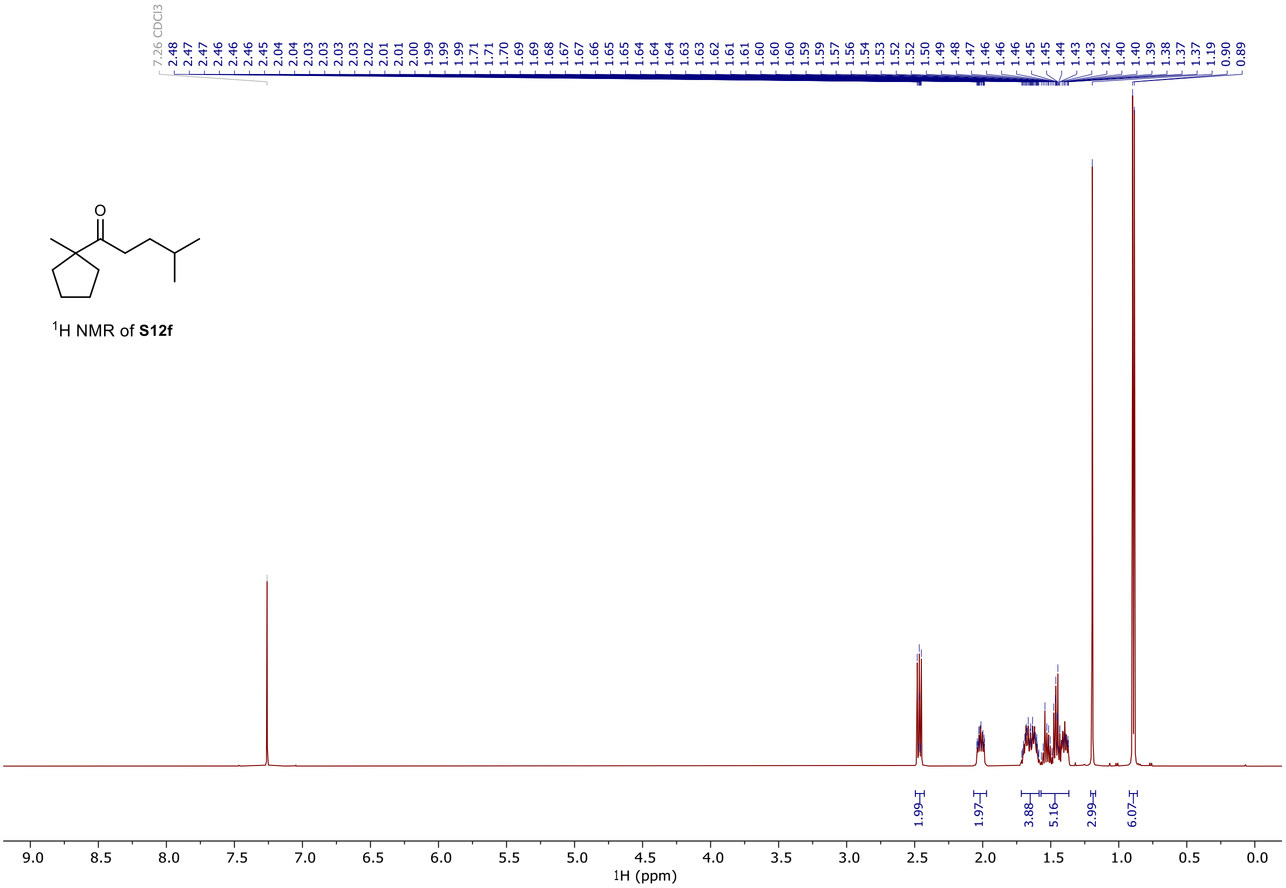
7.26 CDCl3
2.48 2.47 2.46 2.46 2.46 2.45 2.04 2.04 2.03 2.03 2.03 2.02 2.01 2.01 2.00 1.99 1.99 1.99 1.71 1.71 1.70 1.69 1.69 1.68 1.67 1.67 1.66 1.65 1.65 1.64 1.64 1.63 1.63 1.62 1.61 1.61 1.60 1.60 1.59 1.59 1.57 1.56 1.54 1.53 1.52 1.52 1.50 1.49 1.48 1.47 1.46 1.46 1.46 1.45 1.45 1.44 1.43 1.43 1.42 1.40 1.40 1.39 1.38 1.37 1.37 1.19 0.90 0.89
O
^{1}H NMR of **S12f**
1.99
1.97
3.88
5.16
2.99
6.07
9.0 8.5 8.0 7.5 7.0 6.5 6.0 5.5 5.0 4.5 4.0 3.5 3.0 2.5 2.0 1.5 1.0 0.5 0.0
1H (ppm)

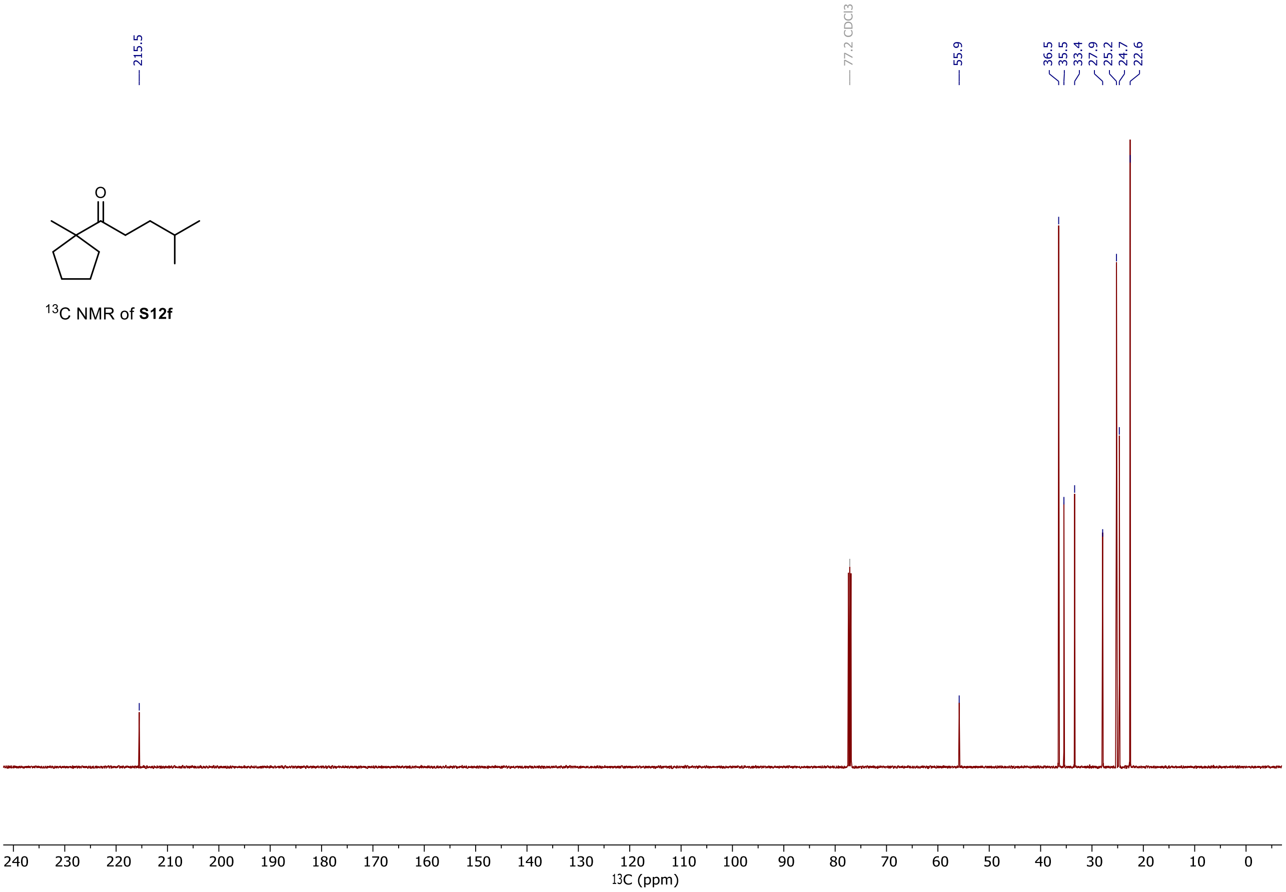
215.5
77.2 CDCl3
55.9
36.5 35.5 33.4 27.9 25.2 24.7 22.6
O
^{13}C NMR of **S12f**
240 230 220 210 200 190 180 170 160 150 140 130 120 110 100 90 80 70 60 50 40 30 20 10 0
13C (ppm)

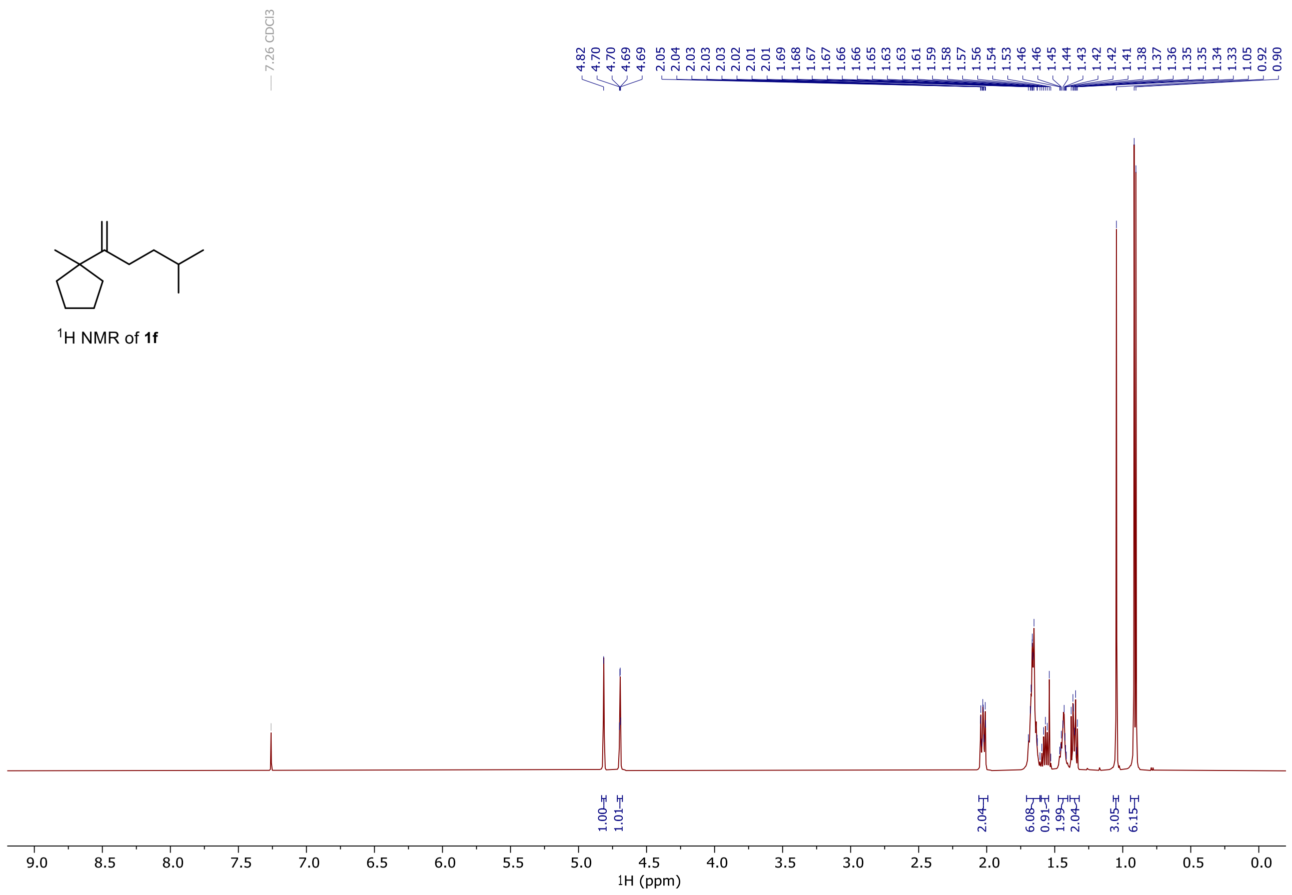
7.26 CDCl3
4.82
4.70
4.70
4.69
4.69
2.05
2.04
2.03
2.03
2.03
2.02
2.01
2.01
1.69
1.68
1.67
1.67
1.66
1.66
1.65
1.63
1.63
1.61
1.59
1.58
1.57
1.56
1.54
1.53
1.46
1.46
1.45
1.44
1.43
1.42
1.42
1.41
1.38
1.37
1.36
1.35
1.35
1.34
1.33
1.05
0.92
0.90
^{1}H NMR of **1f**
1.00
1.01
2.04
6.08
0.91
1.99
2.04
3.05
6.15
1H (ppm)

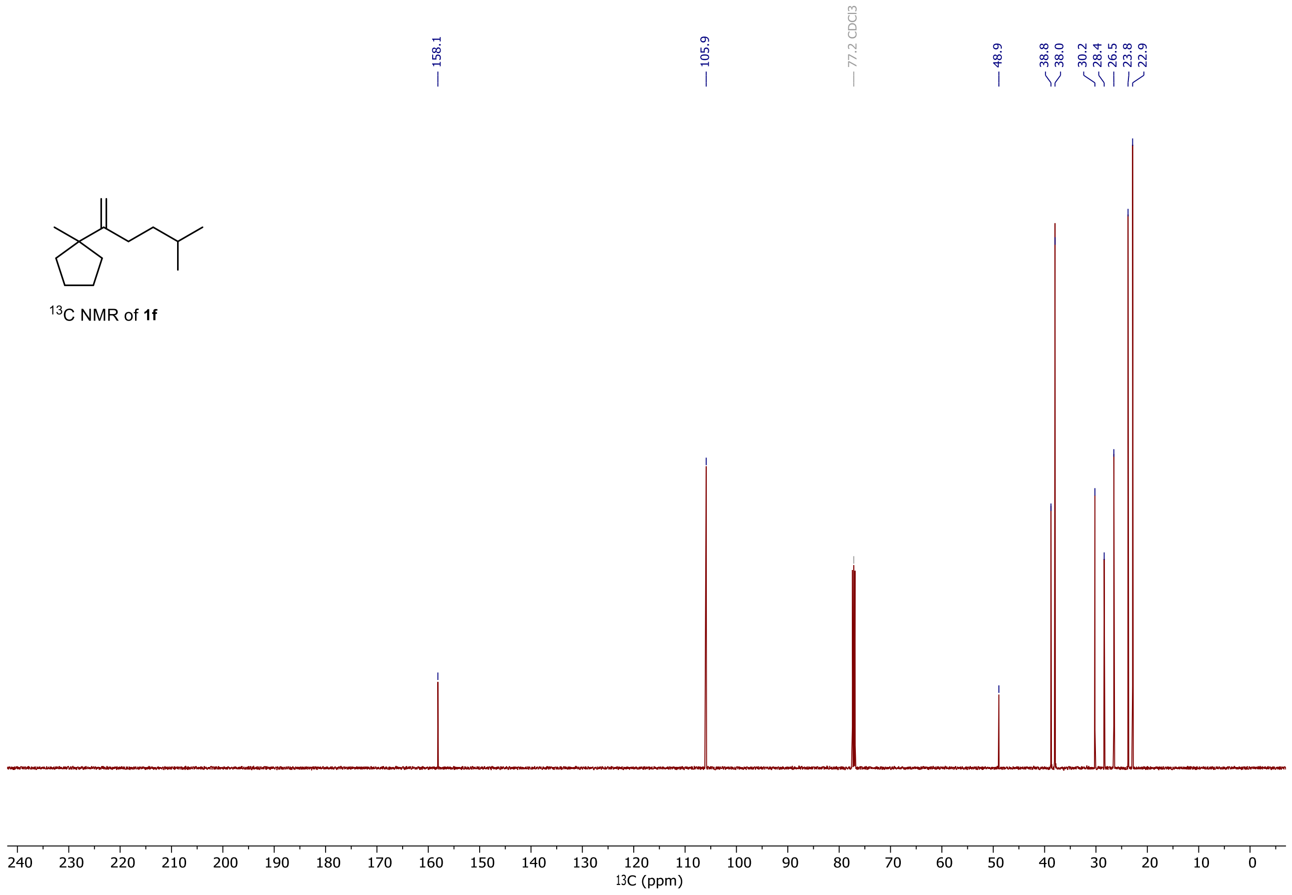
158.1
105.9
77.2 CDCl3
48.9
38.8
38.0
30.2
28.4
26.5
23.8
22.9
^{13}C NMR of **1f**
13C (ppm)

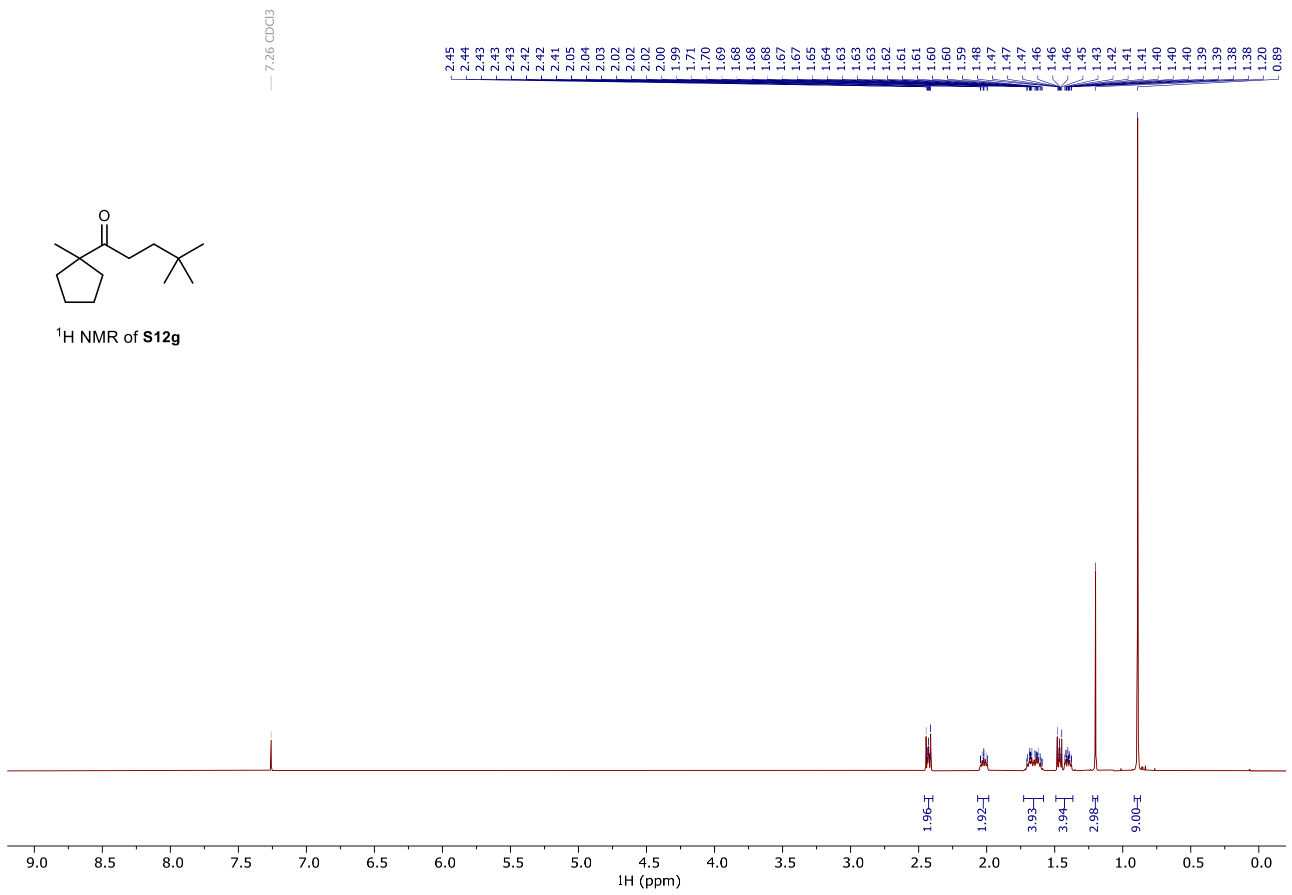
7.26 CDCl3
2.45 2.44 2.43 2.43 2.43 2.42 2.42 2.41 2.05 2.04 2.03 2.02 2.02 2.02 2.00 1.99 1.71 1.70 1.69 1.68 1.68 1.67 1.67 1.65 1.64 1.63 1.63 1.63 1.62 1.61 1.61 1.60 1.60 1.59 1.48 1.47 1.47 1.47 1.46 1.46 1.46 1.45 1.43 1.42 1.41 1.41 1.40 1.40 1.40 1.39 1.39 1.38 1.38 1.20 0.89
O
1H NMR of S12g
1.96
1.92
3.93
3.94
2.98
9.00
9.0 8.5 8.0 7.5 7.0 6.5 6.0 5.5 5.0 4.5 4.0 3.5 3.0 2.5 2.0 1.5 1.0 0.5 0.0
1H (ppm)

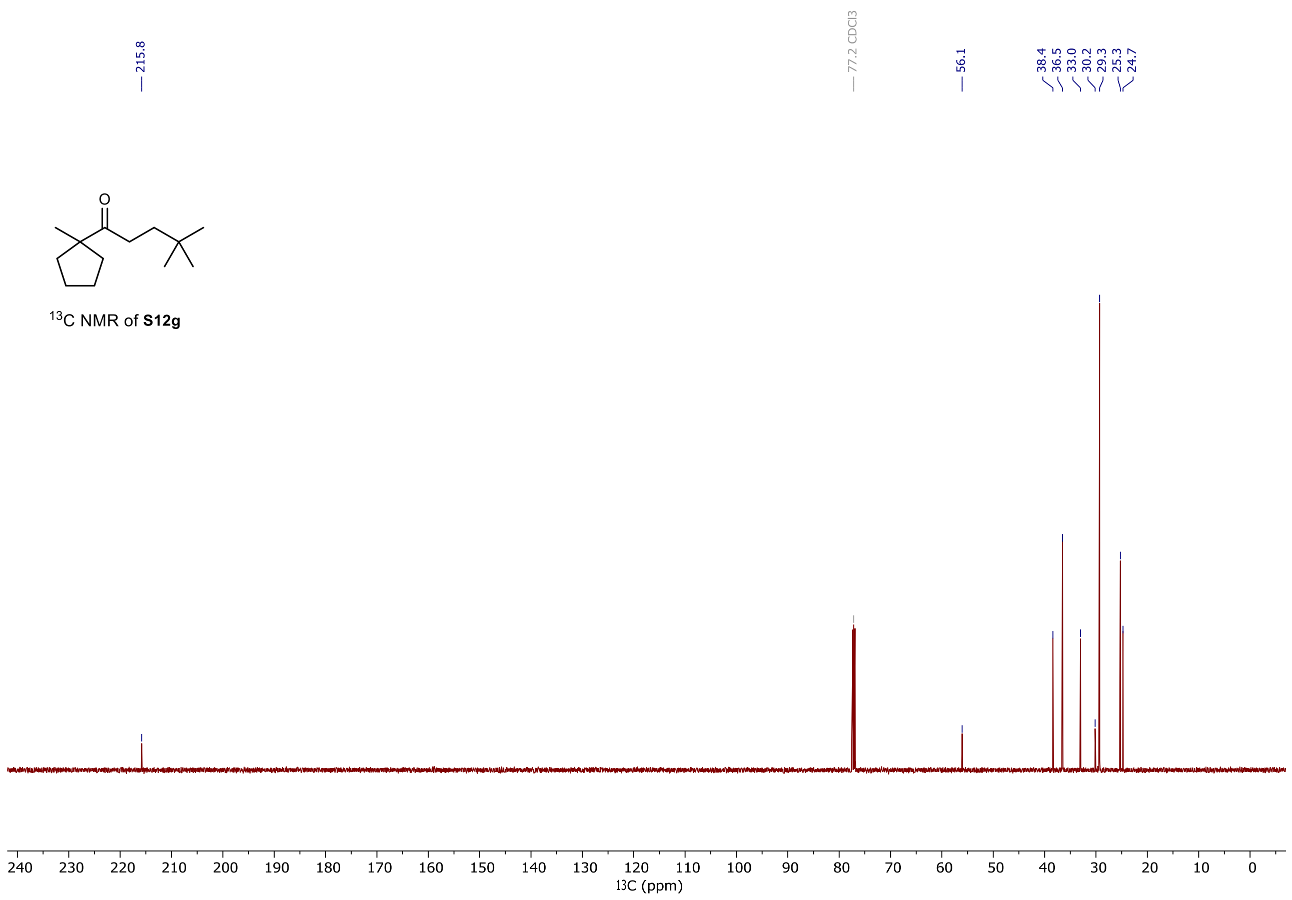
215.8
77.2 CDCl3
56.1
38.4 36.5 33.0 30.2 29.3 25.3 24.7
O
13C NMR of S12g
240 230 220 210 200 190 180 170 160 150 140 130 120 110 100 90 80 70 60 50 40 30 20 10 0
13C (ppm)

^{1}H NMR of **1g**

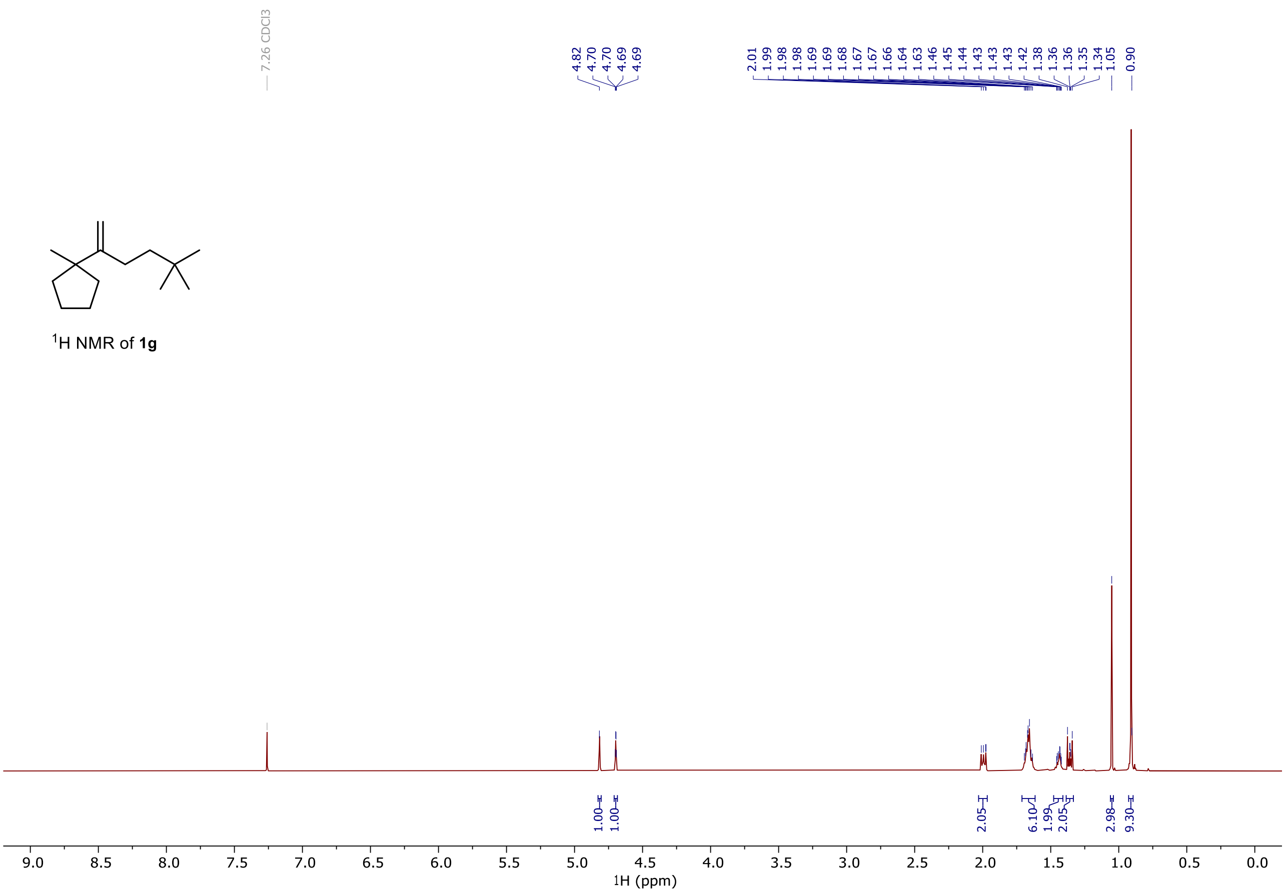

^{13}C NMR of **1g**

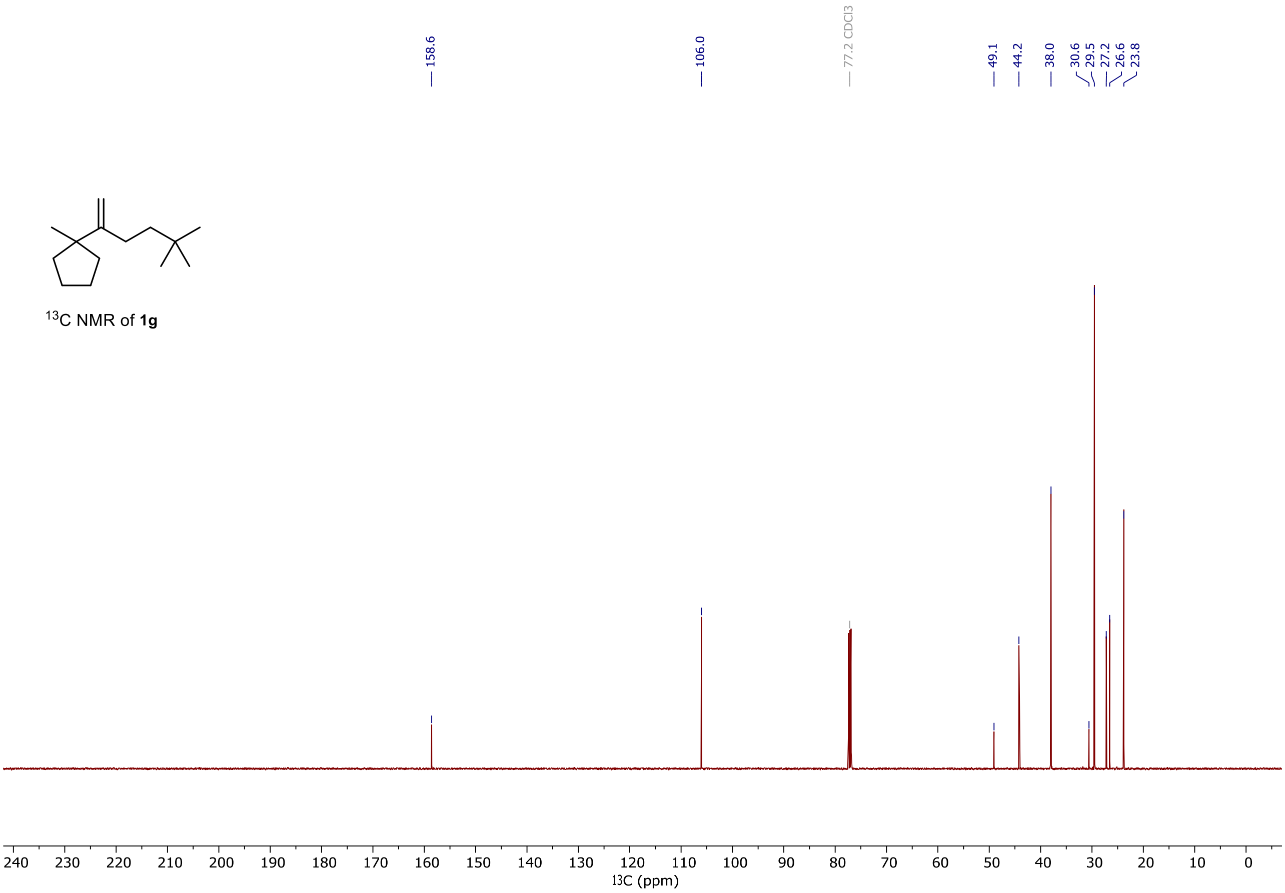

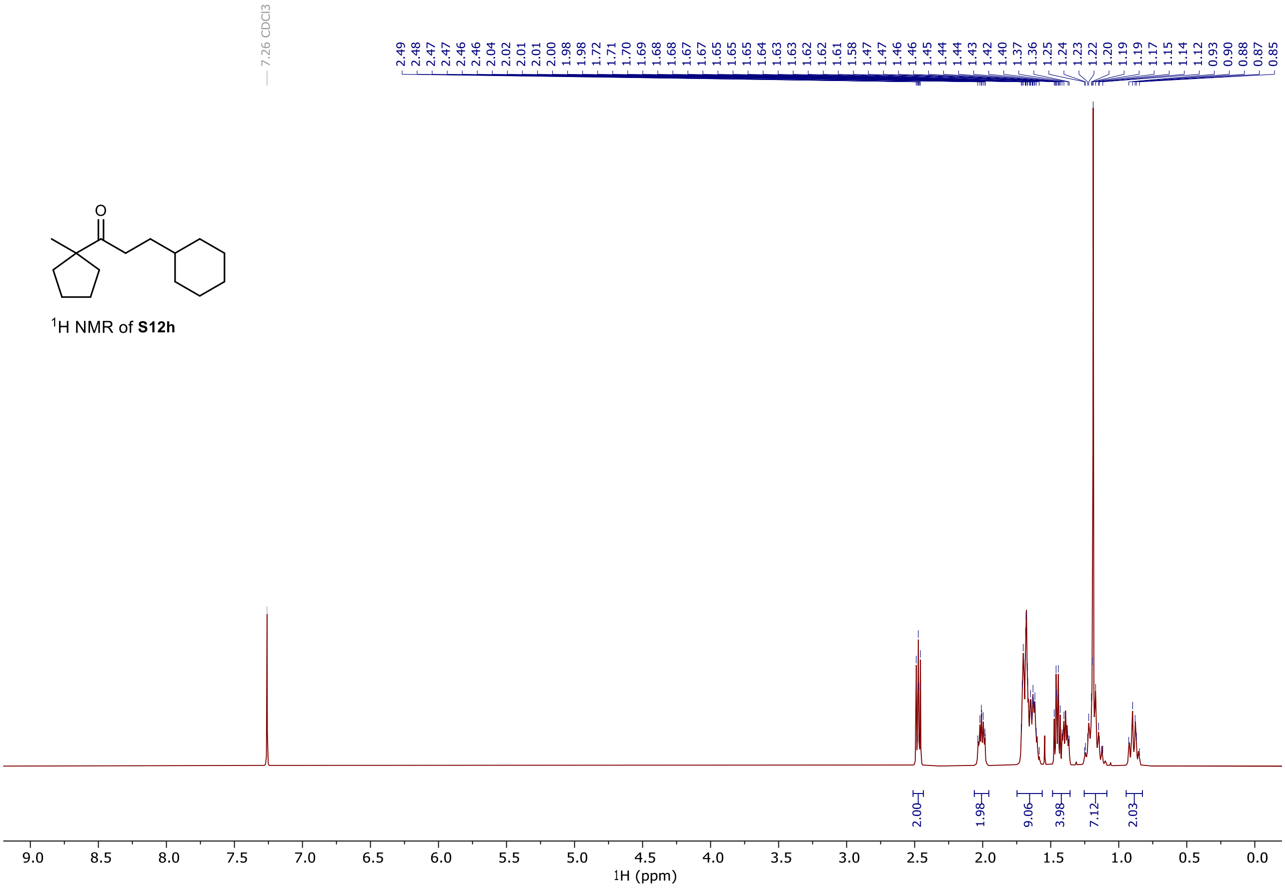

1H NMR of S12h
7.26 CDCl3
2.00
1.98
9.06
3.98
7.12
2.03
1H (ppm)

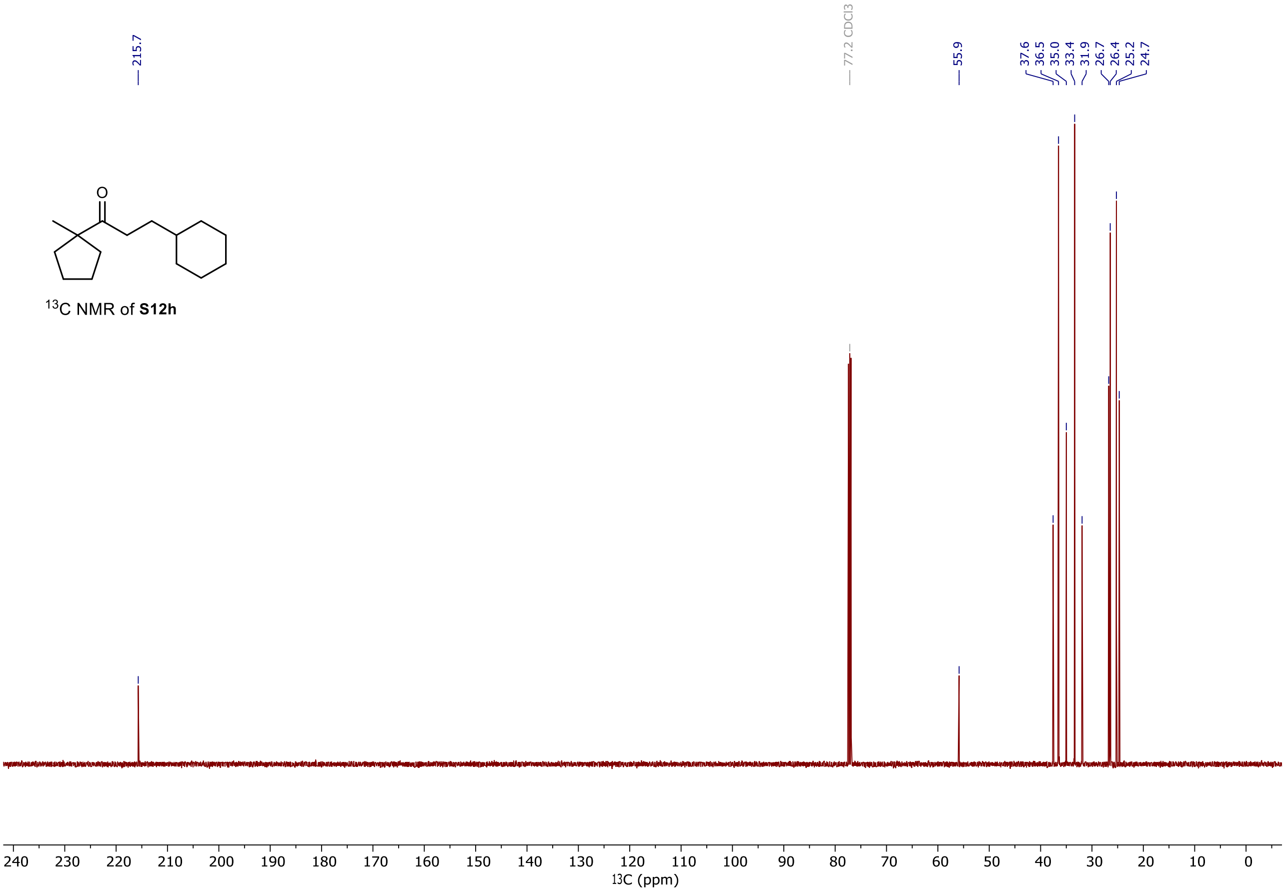

13C NMR of S12h
215.7
77.2 CDCl3
55.9
37.6
36.5
35.0
33.4
31.9
26.7
26.4
25.2
24.7
13C (ppm)

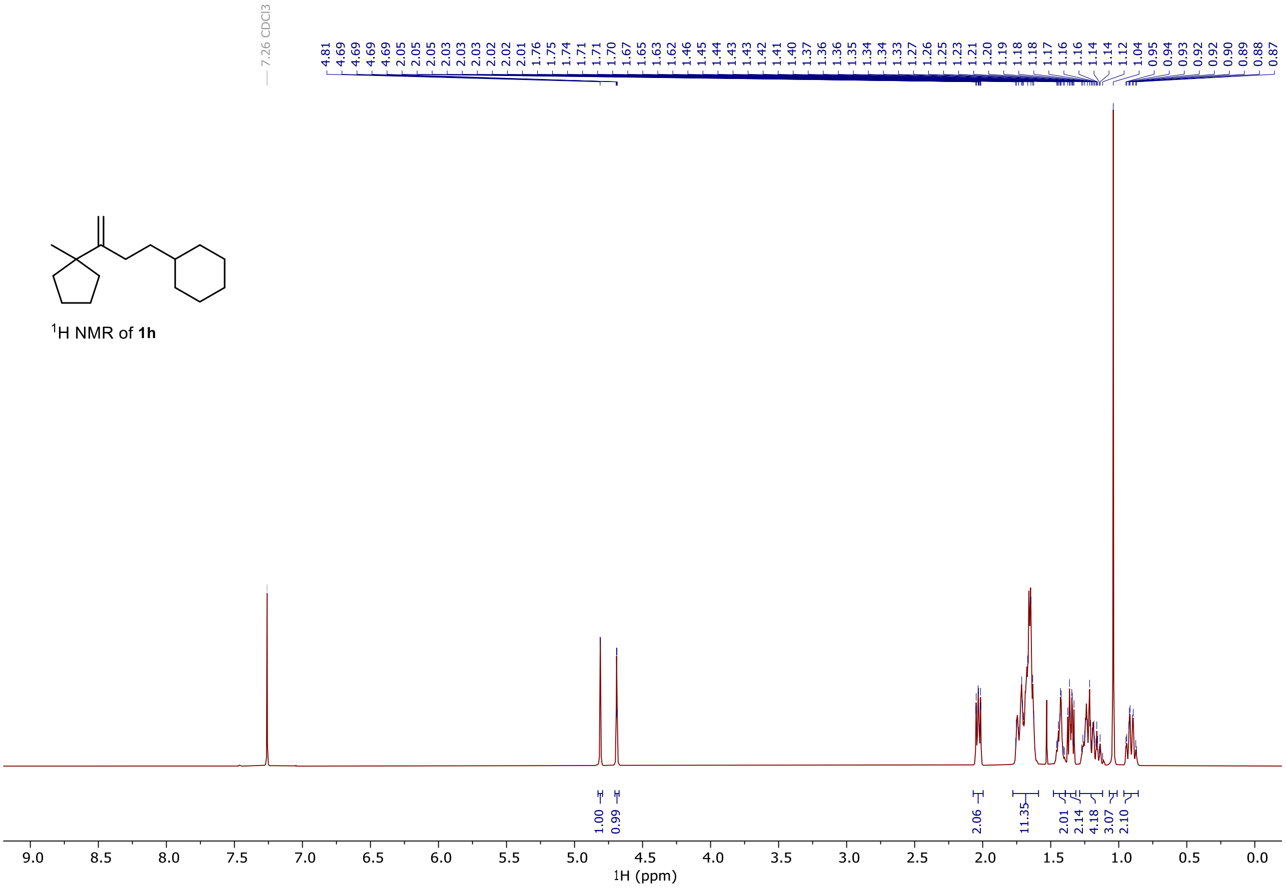

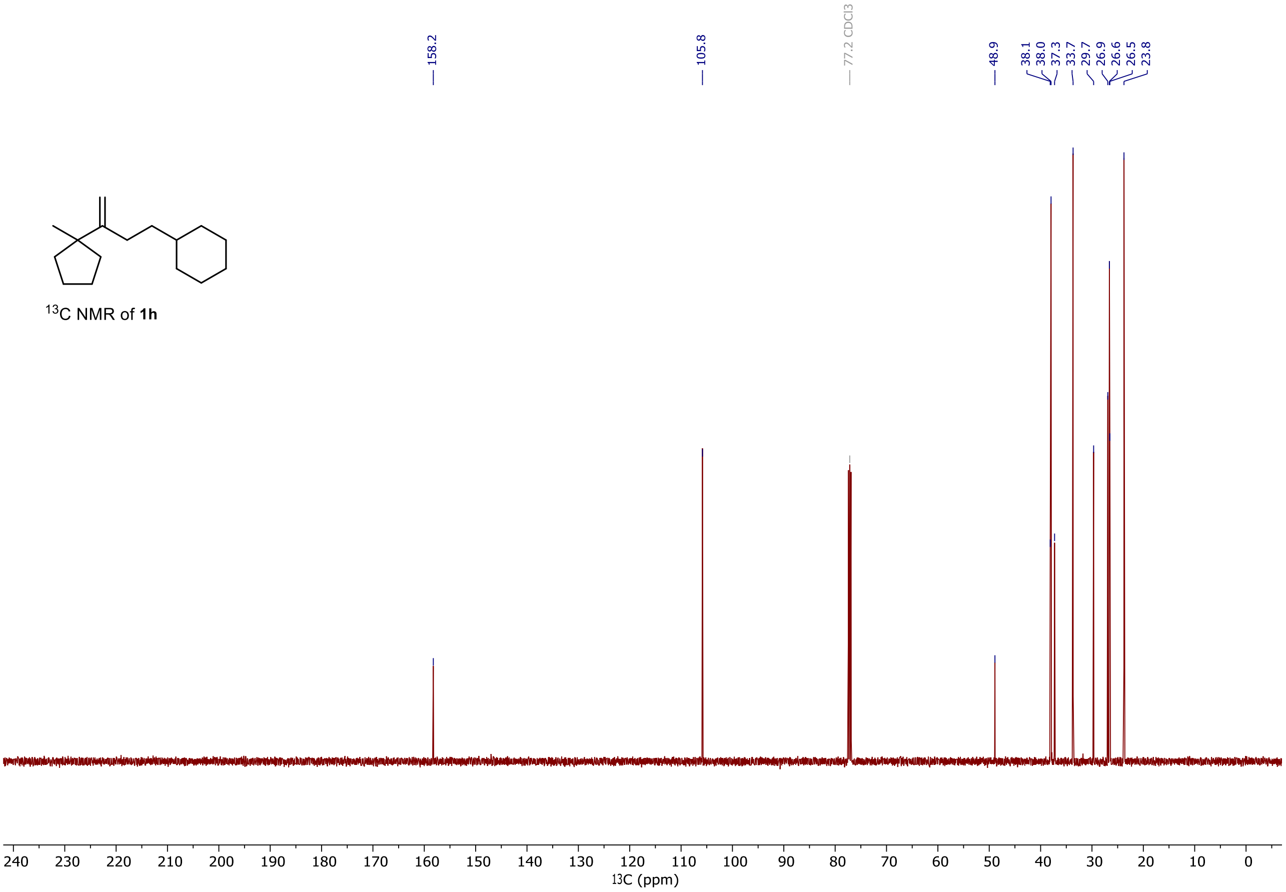

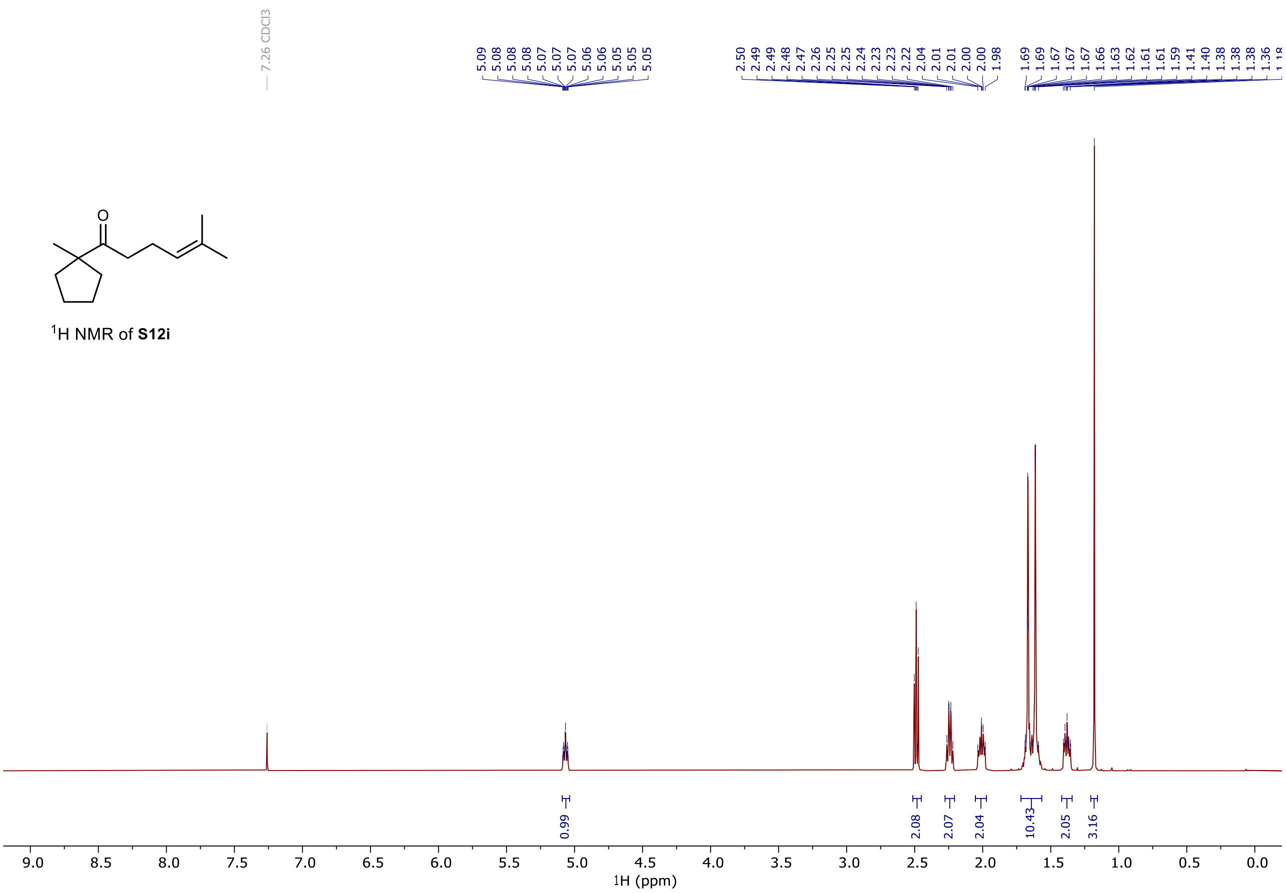

^{1}H NMR of **S12i**
7.26 CDCl3
1H (ppm)

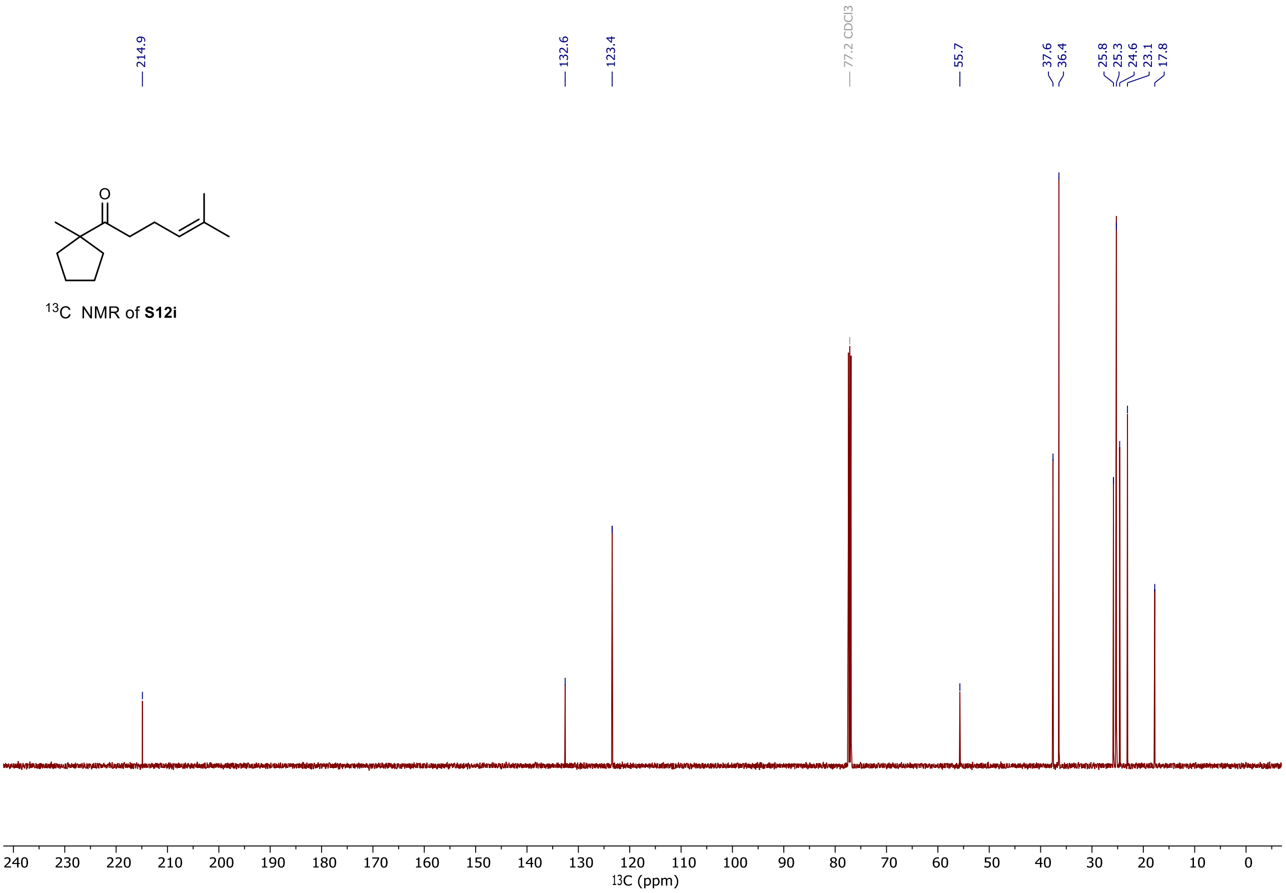

^{13}C NMR of **S12i**
214.9
132.6
123.4
77.2 CDCl3
55.7
37.6
36.4
25.8
25.3
24.6
23.1
17.8
13C (ppm)

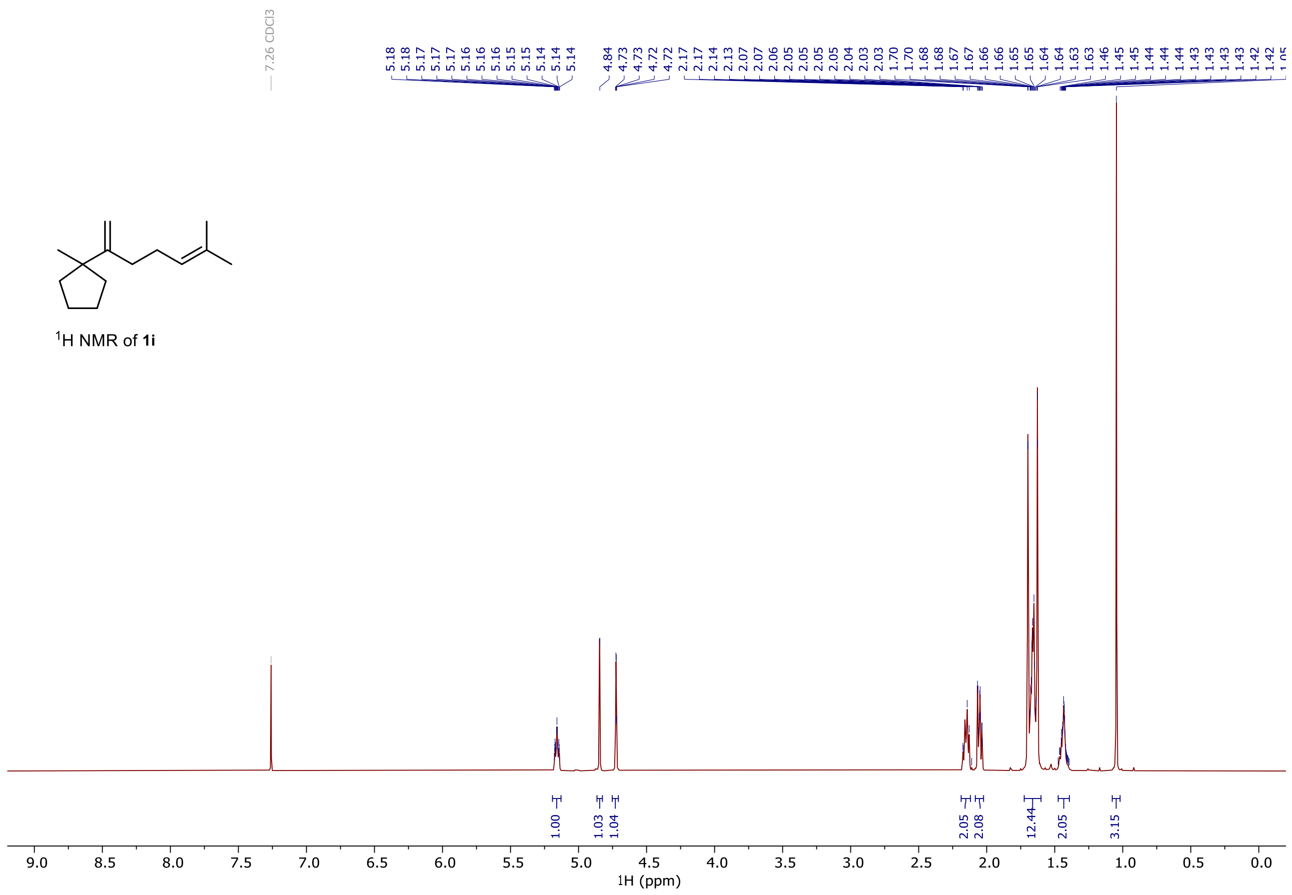

^{1}H NMR of **1i**

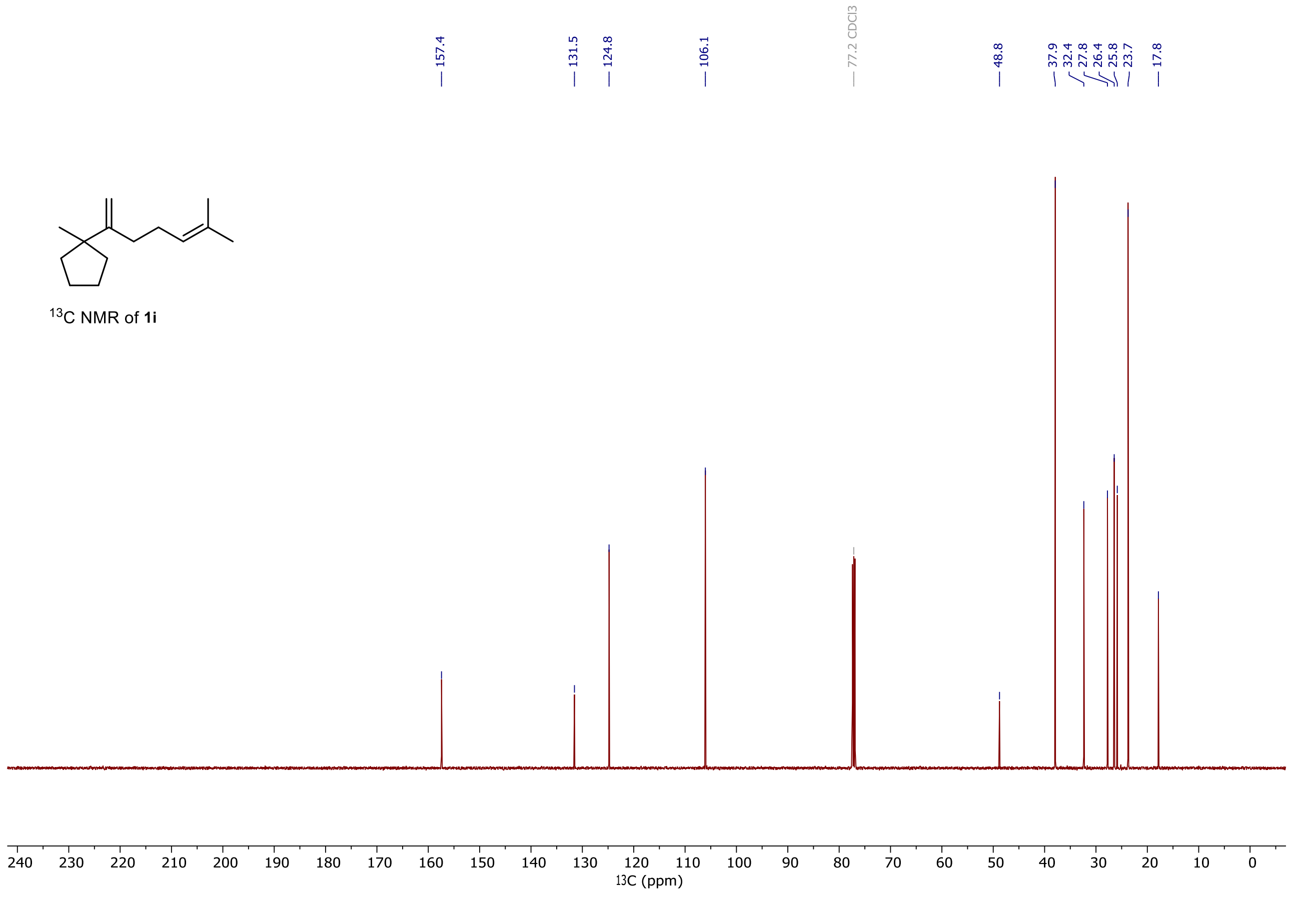

^{13}C NMR of **1i**

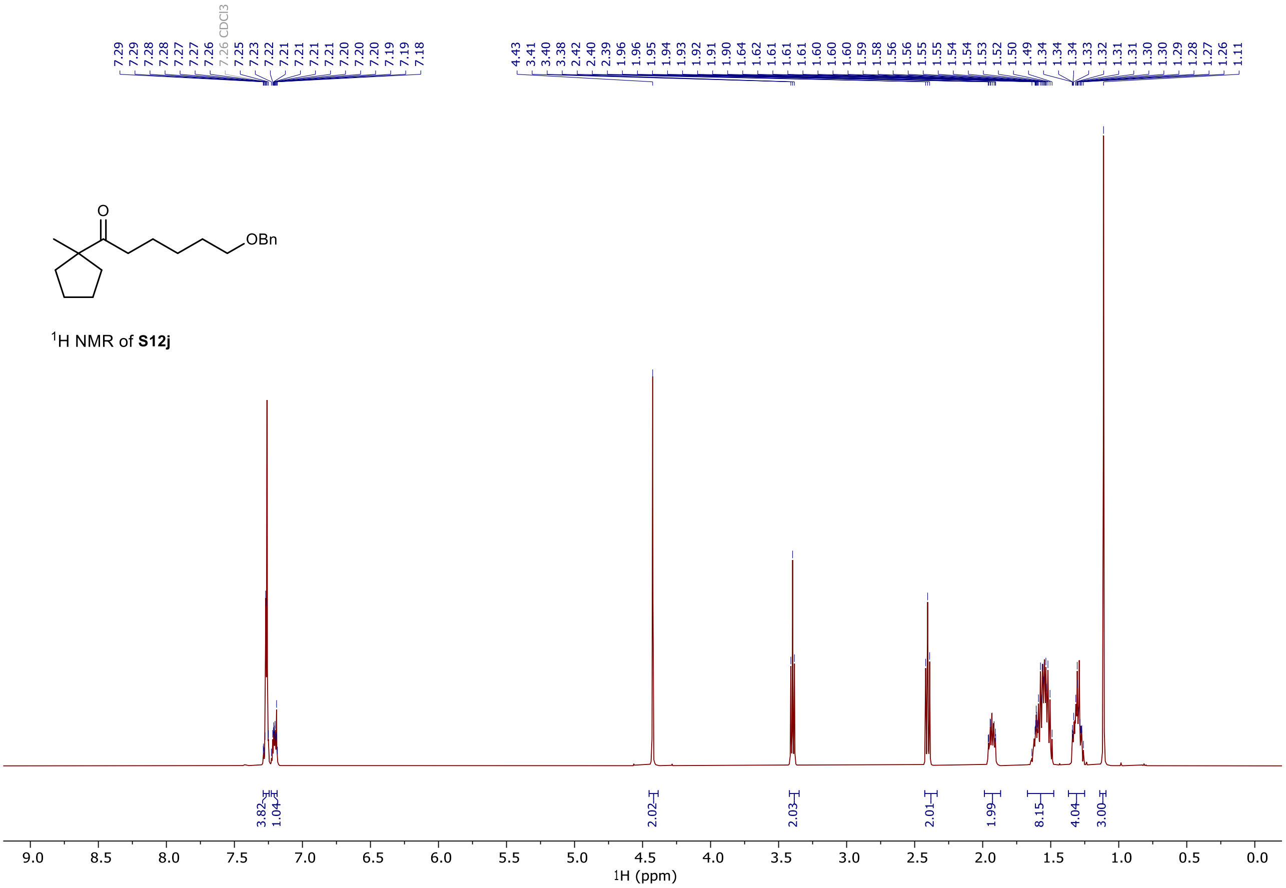
OBn
O
1H NMR of S12j
1H (ppm)
13C (ppm)

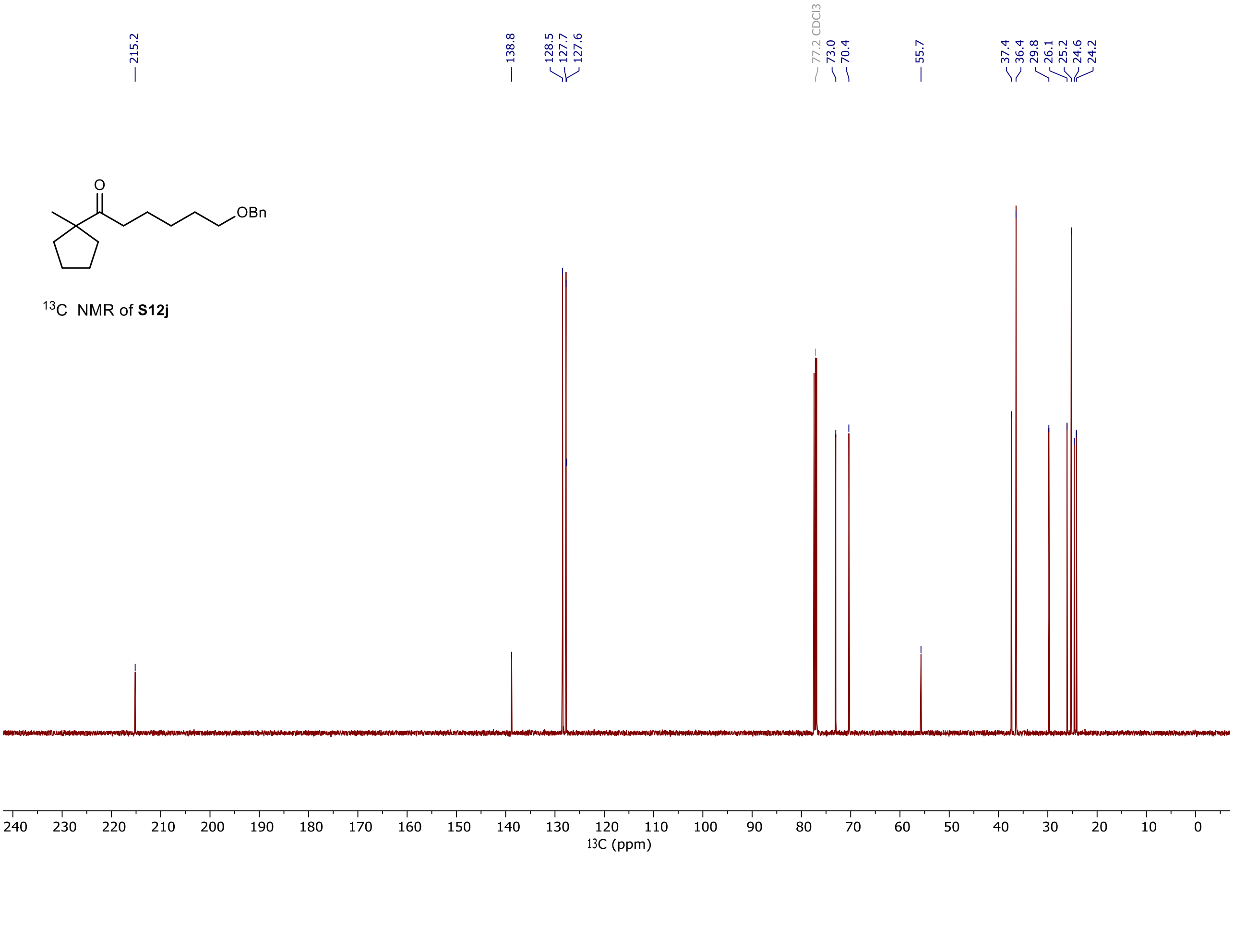
OBn
O
13C NMR of S12j
13C (ppm)

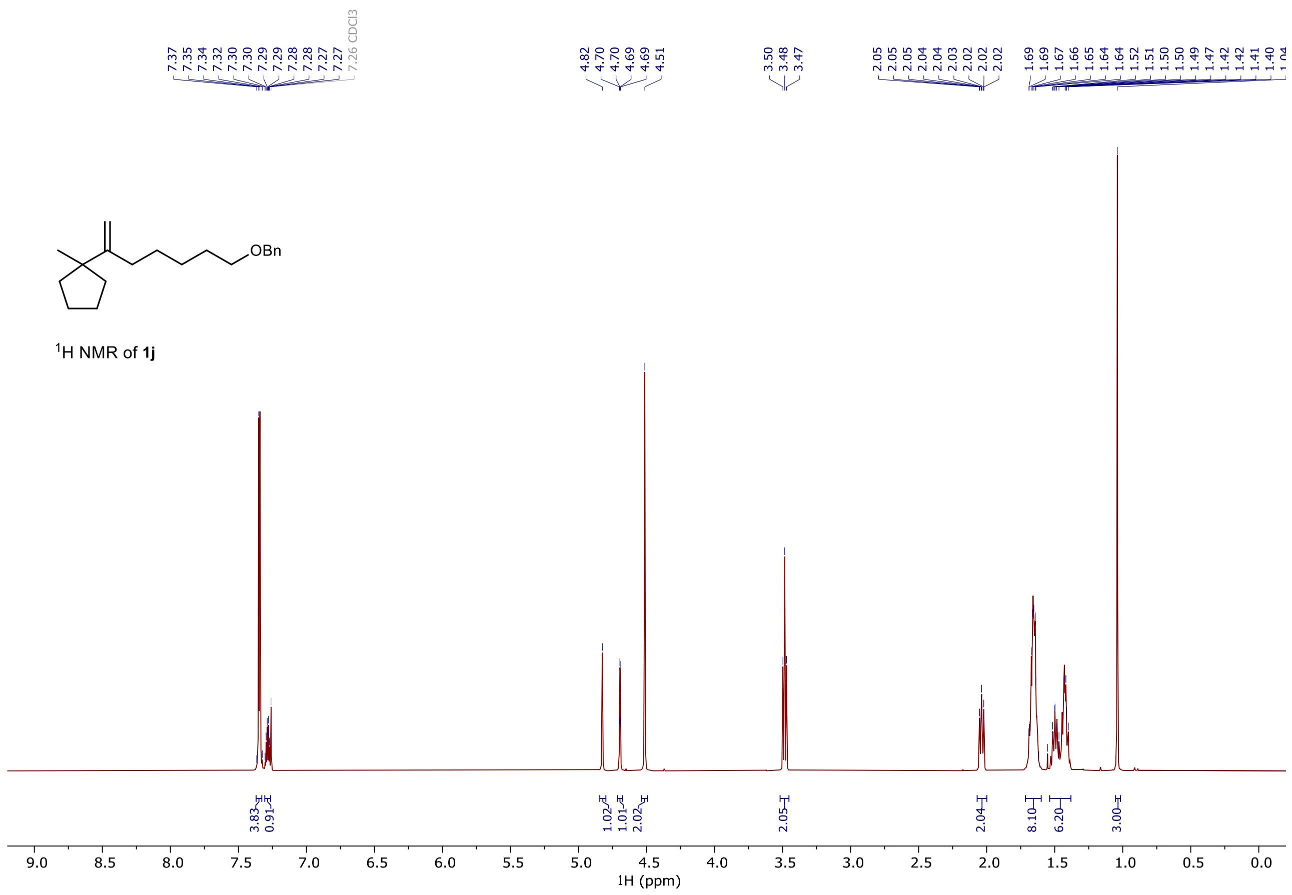
OBn
1H NMR of 1j
7.37
7.35
7.34
7.32
7.30
7.29
7.29
7.28
7.28
7.27
7.27
7.26 CDCl3
4.82
4.70
4.70
4.69
4.69
4.51
3.50
3.48
3.47
2.05
2.05
2.05
2.04
2.04
2.03
2.02
2.02
2.02
1.69
1.69
1.67
1.66
1.65
1.64
1.64
1.52
1.51
1.50
1.50
1.49
1.47
1.42
1.42
1.41
1.40
3.83
0.91
1.02
1.01
2.02
2.05
2.04
8.10
6.20
3.00
9.0
8.5
8.0
7.5
7.0
6.5
6.0
5.5
5.0
4.5
4.0
3.5
3.0
2.5
2.0
1.5
1.0
0.5
0.0
1H (ppm)

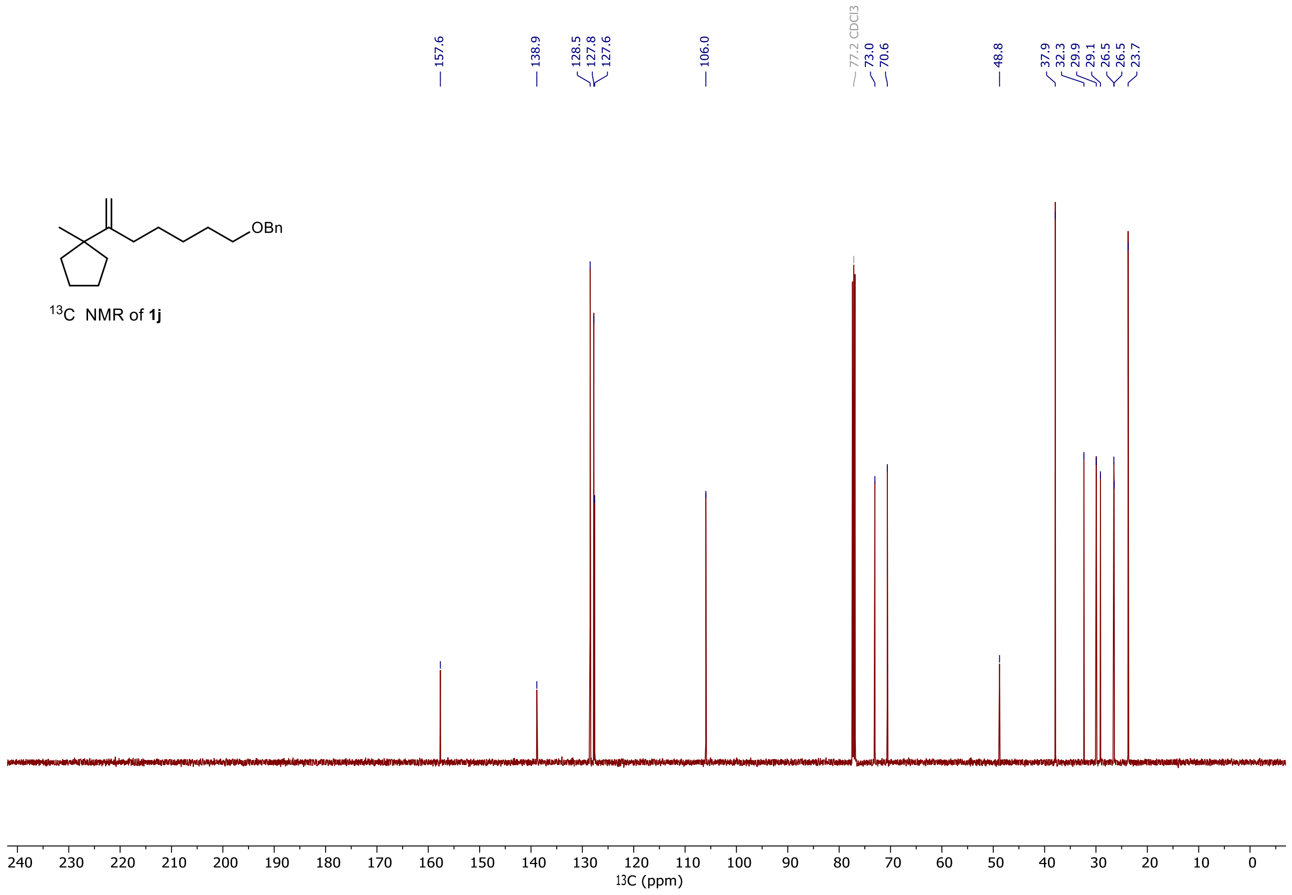
OBn
13C NMR of 1j
157.6
138.9
128.5
127.8
127.6
106.0
77.2 CDCl3
73.0
70.6
48.8
37.9
32.3
29.9
29.1
26.5
26.5
23.7
240
230
220
210
200
190
180
170
160
150
140
130
120
110
100
90
80
70
60
50
40
30
20
10
0
13C (ppm)

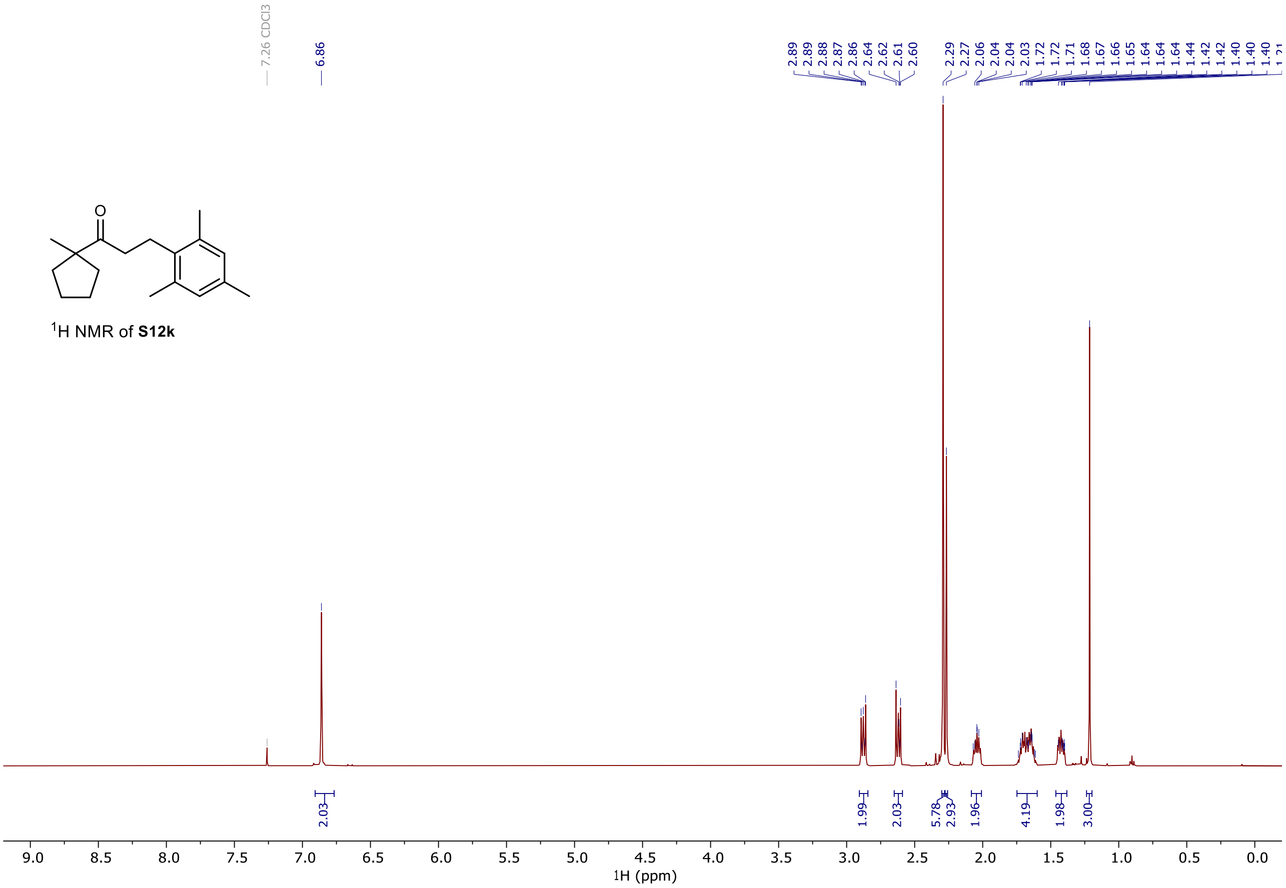

7.26 CDCl3
6.86
2.89
2.89
2.88
2.87
2.86
2.64
2.62
2.61
2.60
2.29
2.27
2.06
2.04
2.04
2.03
1.72
1.72
1.71
1.68
1.67
1.66
1.65
1.64
1.64
1.64
1.44
1.42
1.42
1.40
1.40
1.40
O
1H NMR of S12k
2.03
1.99
2.03
5.78
2.93
1.96
4.19
1.98
3.00
9.0
8.5
8.0
7.5
7.0
6.5
6.0
5.5
5.0
4.5
4.0
3.5
3.0
2.5
2.0
1.5
1.0
0.5
0.0
1H (ppm)

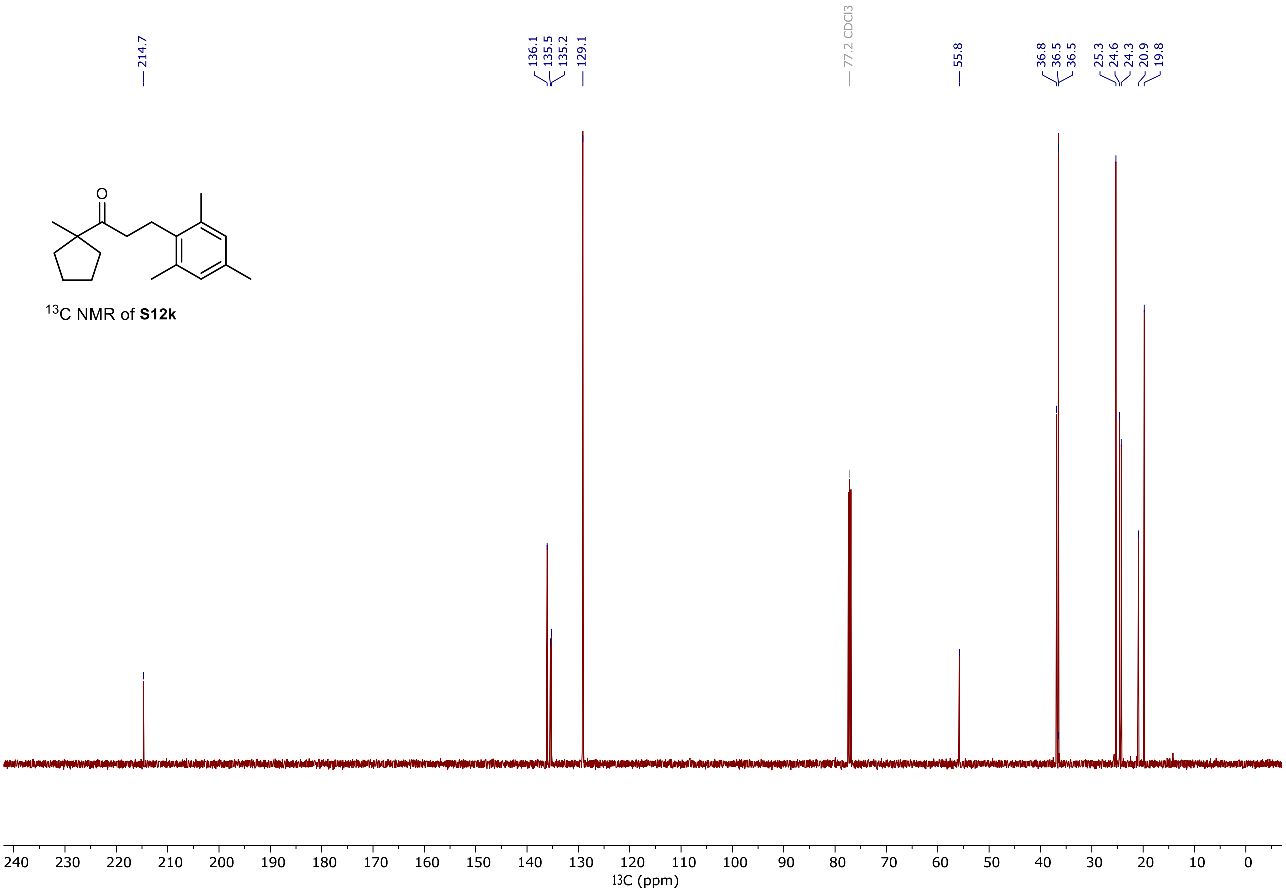

214.7
136.1
135.5
135.2
129.1
77.2 CDCl3
55.8
36.8
36.5
36.5
25.3
24.6
24.3
20.9
19.8
O
13C NMR of S12k
240
230
220
210
200
190
180
170
160
150
140
130
120
110
100
90
80
70
60
50
40
30
20
10
0
13C (ppm)

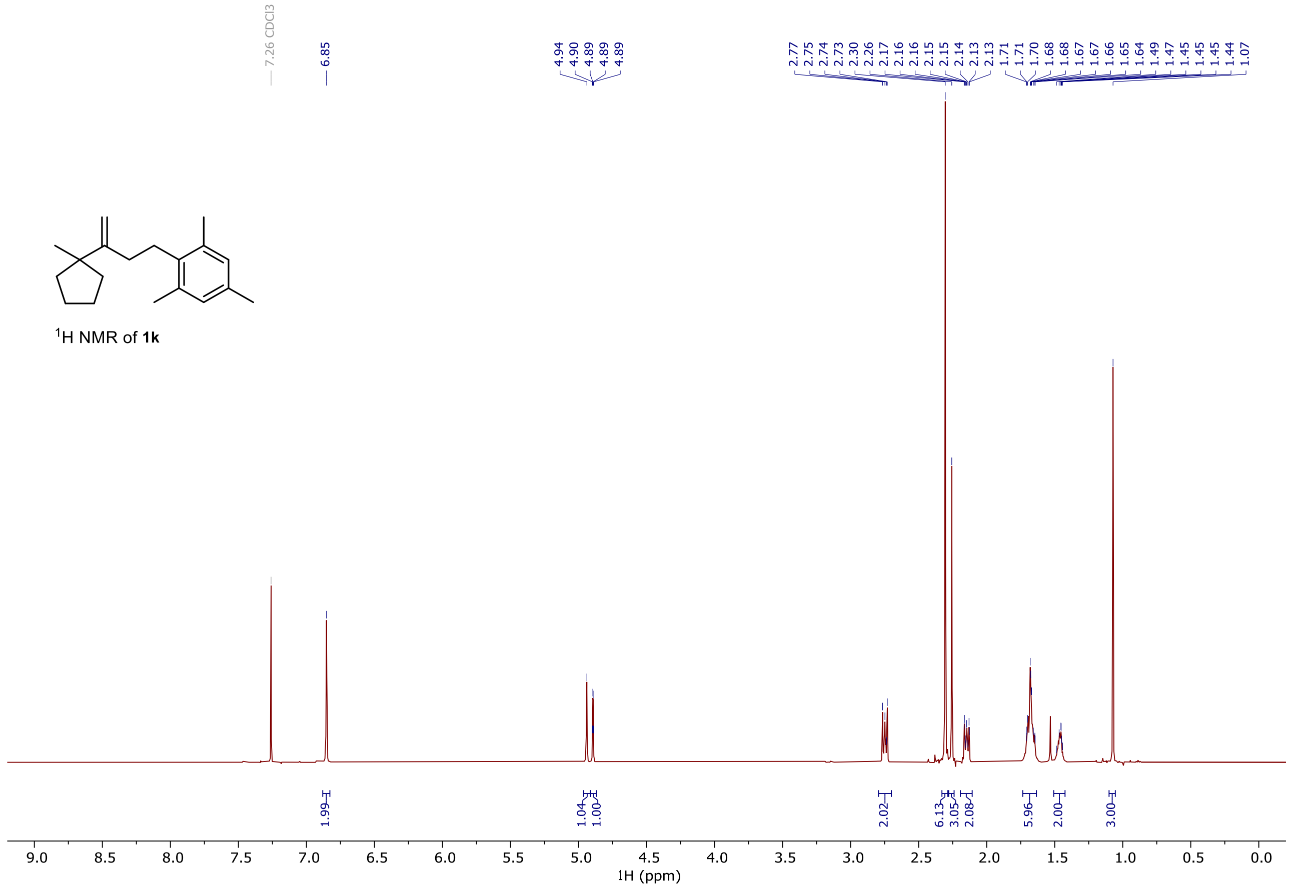
7.26 CDCl3
6.85
4.94 4.90 4.89 4.89 4.89
2.77 2.75 2.74 2.73 2.30 2.26 2.17 2.16 2.16 2.15 2.15 2.14 2.13 2.13 1.71 1.71 1.70 1.68 1.68 1.67 1.67 1.66 1.65 1.64 1.49 1.47 1.45 1.45 1.45 1.44 1.07
1H NMR of 1k
1.99
1.04
1.00
2.02
6.13
3.05
2.08
5.96
2.00
3.00
9.0 8.5 8.0 7.5 7.0 6.5 6.0 5.5 5.0 4.5 4.0 3.5 3.0 2.5 2.0 1.5 1.0 0.5 0.0
1H (ppm)

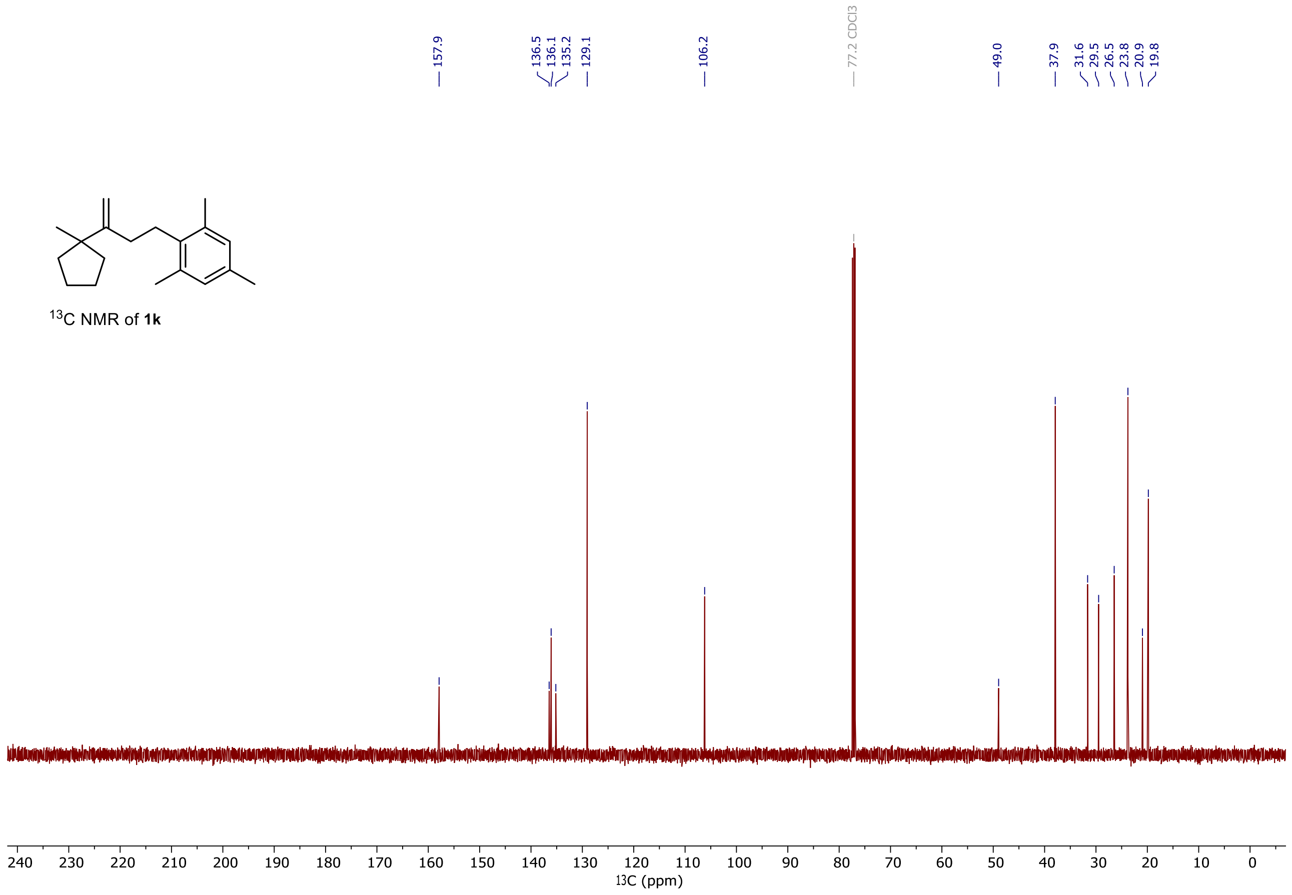
157.9
136.5 136.1 135.2 129.1
106.2
77.2 CDCl3
49.0
37.9
31.6 29.5 26.5 23.8 20.9 19.8
13C NMR of 1k
240 230 220 210 200 190 180 170 160 150 140 130 120 110 100 90 80 70 60 50 40 30 20 10 0
13C (ppm)

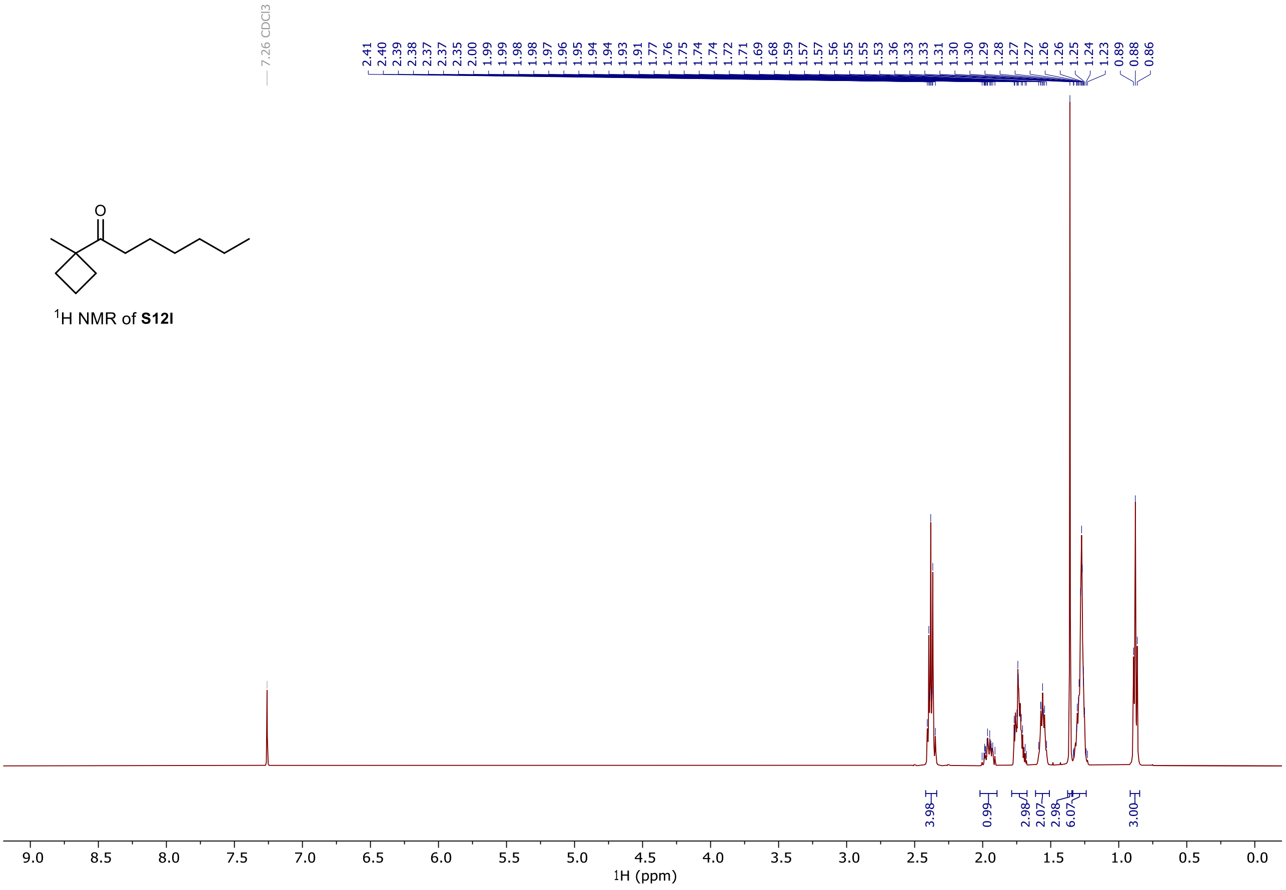

^{1}H NMR of **S12l**

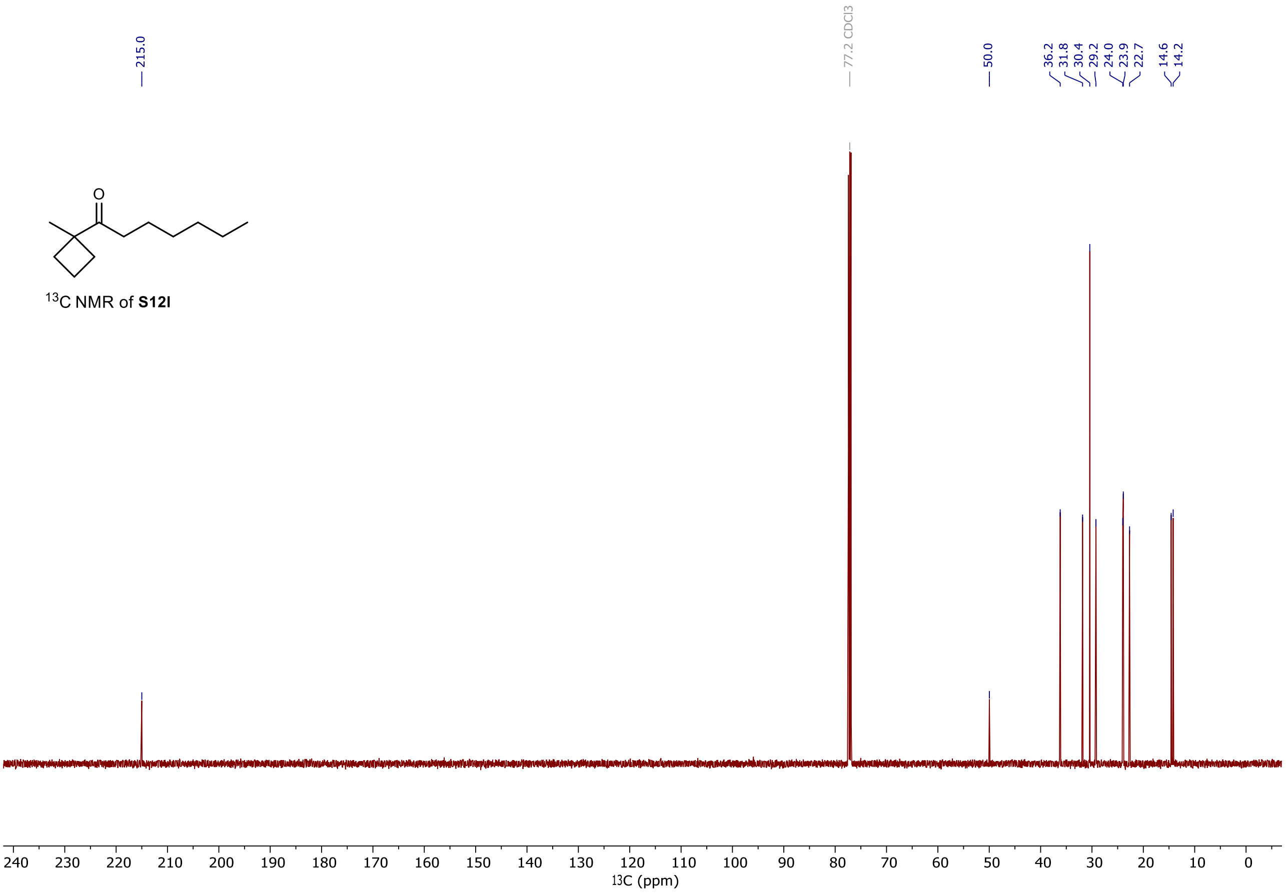

^{13}C NMR of **S12l**

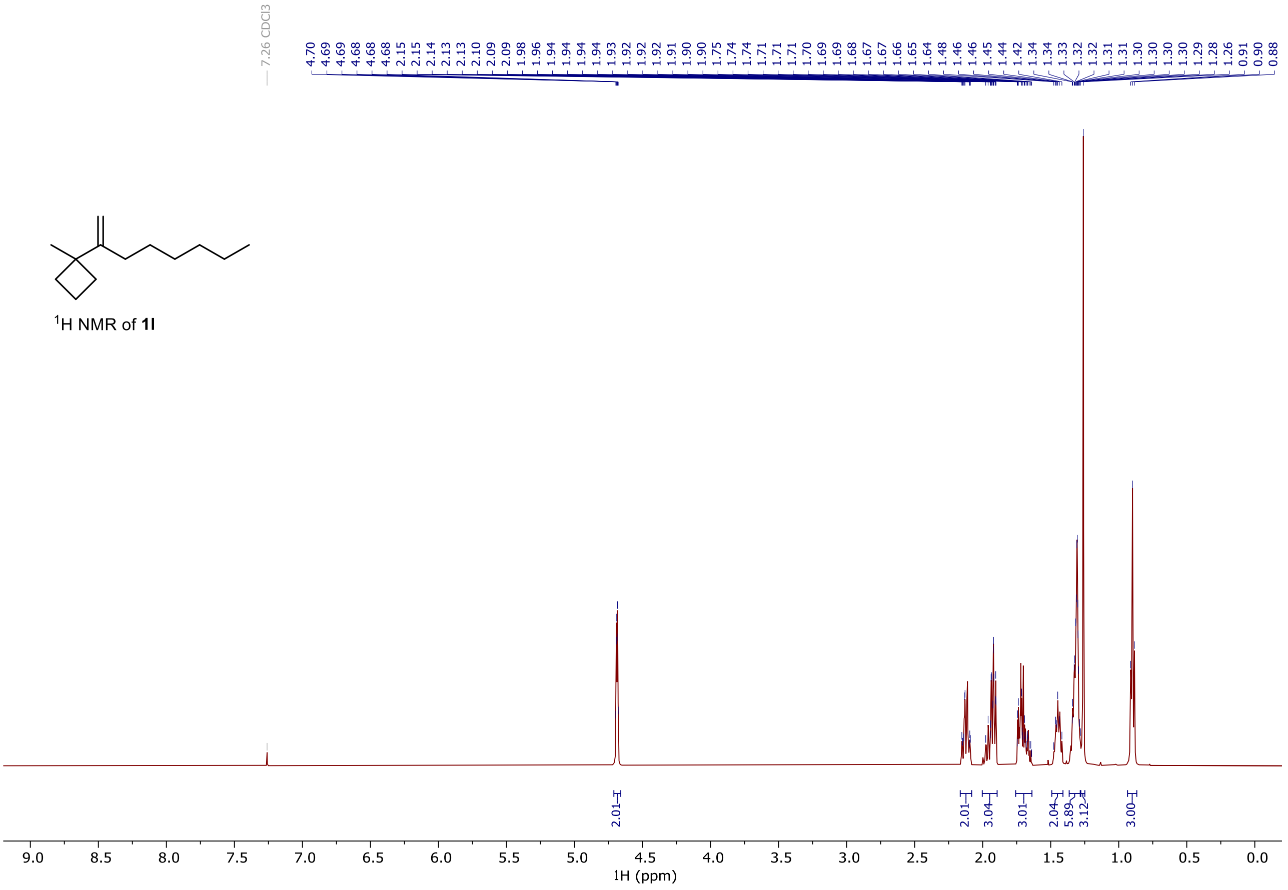
1H NMR of 1l
1H (ppm)

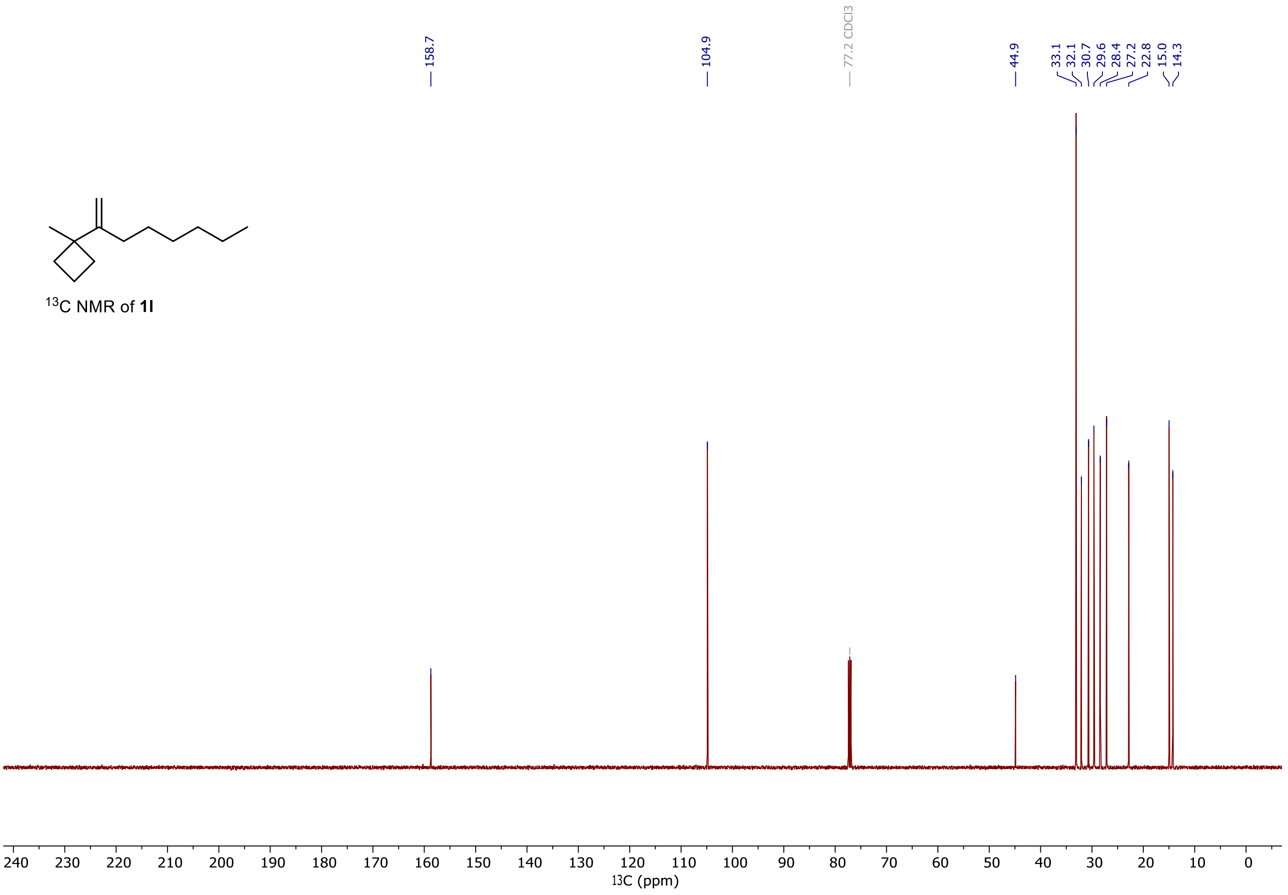
13C NMR of 1l
158.7
104.9
77.2 CDCl3
44.9
33.1
32.1
30.7
29.6
28.4
27.2
22.8
15.0
14.3
13C (ppm)

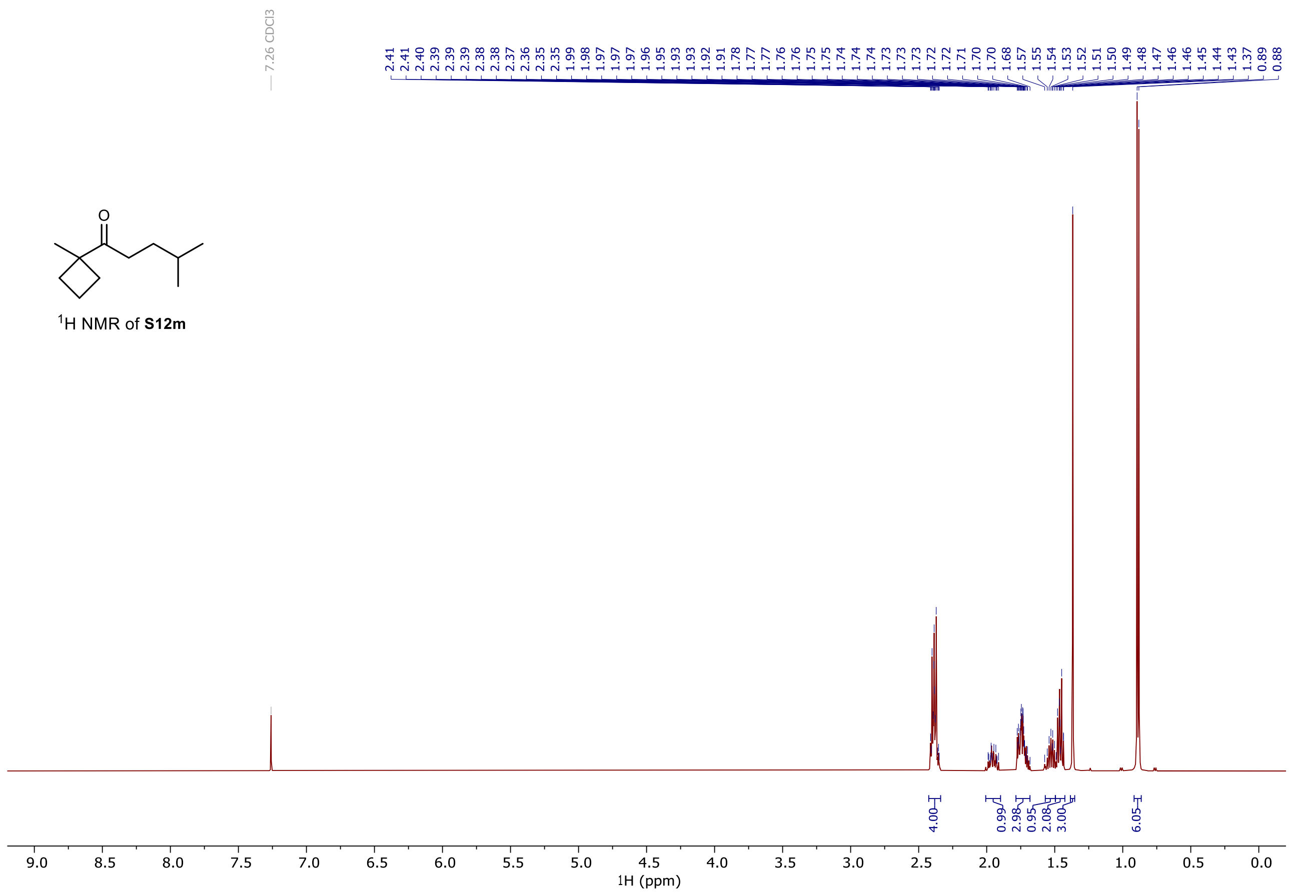

^{1}H NMR of **S12m**

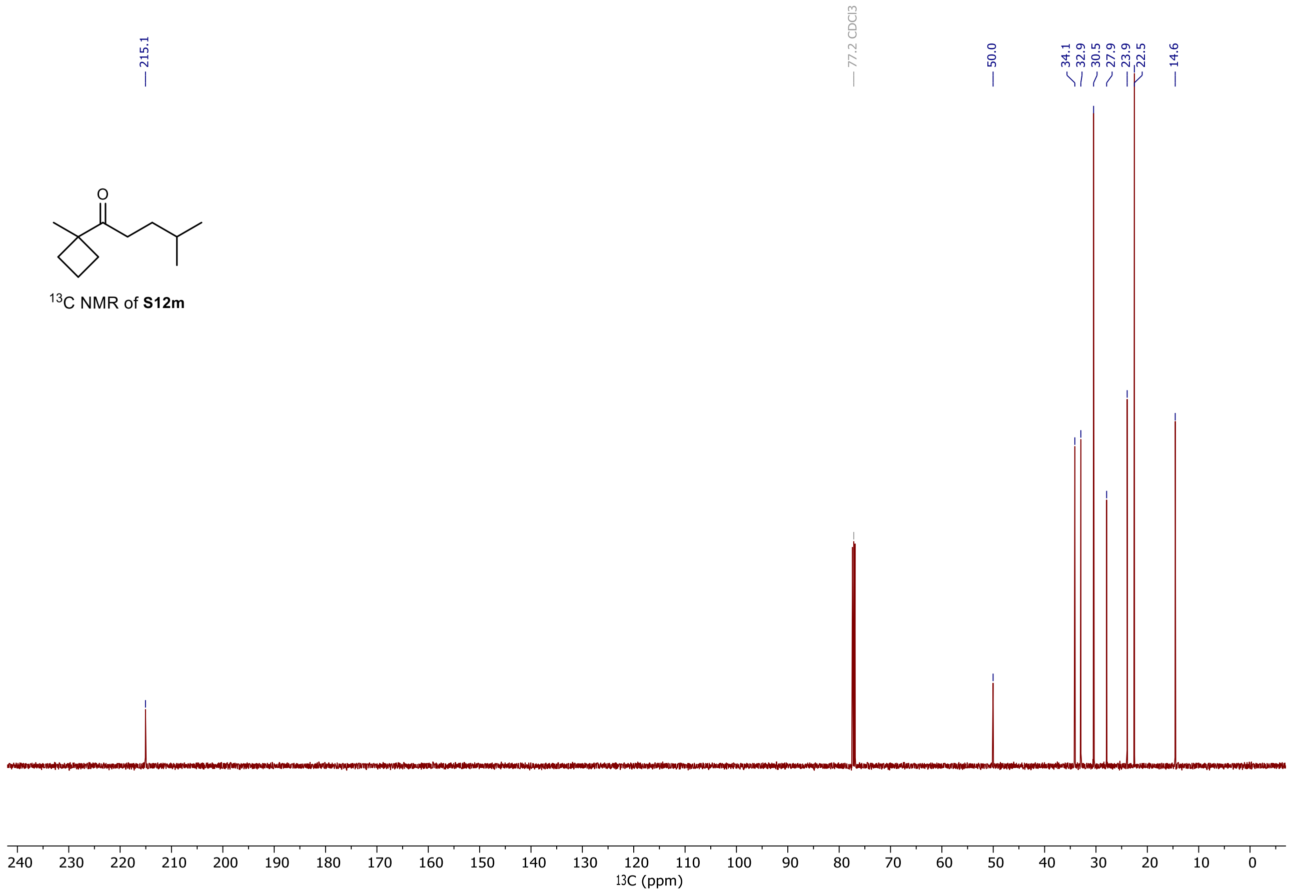

^{13}C NMR of **S12m**

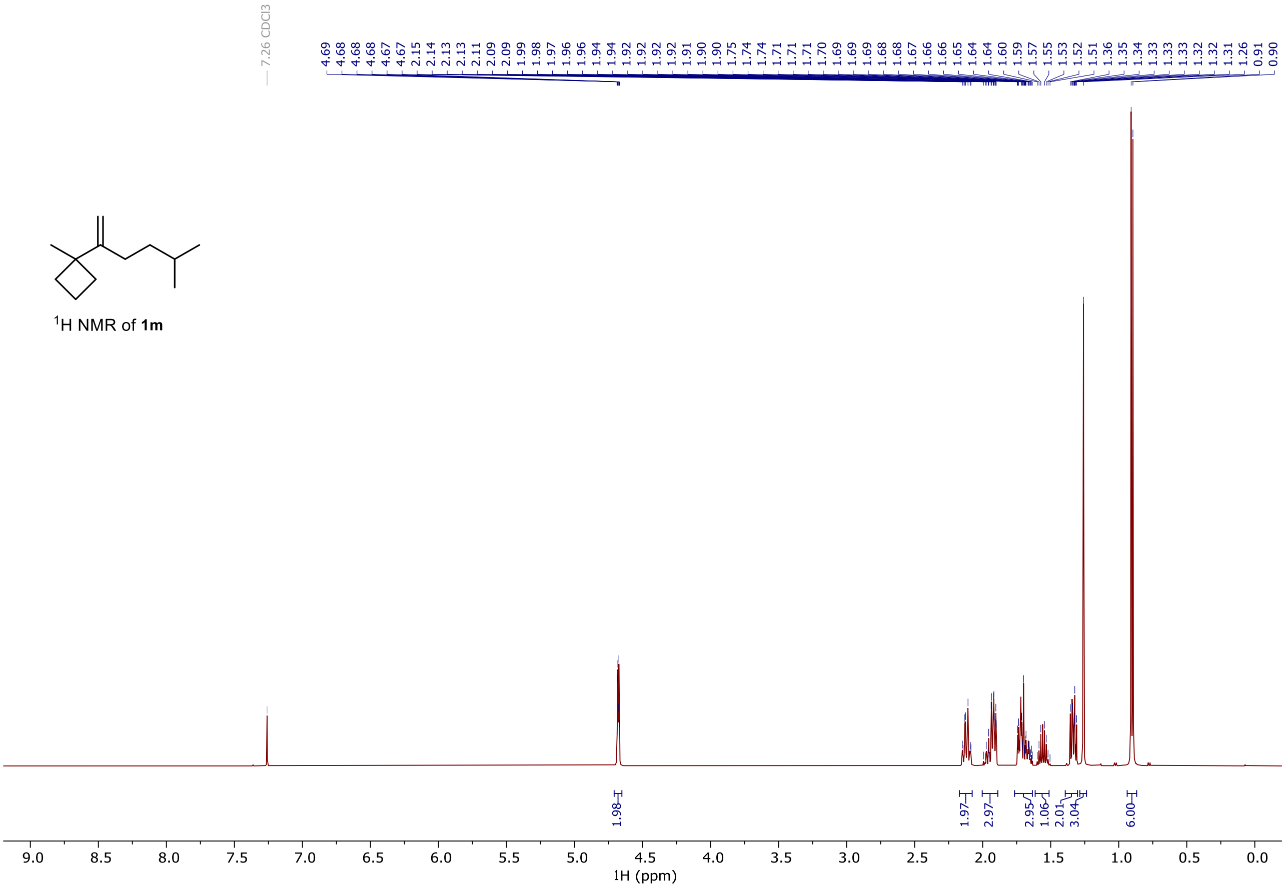

^{1}H NMR of **1m**

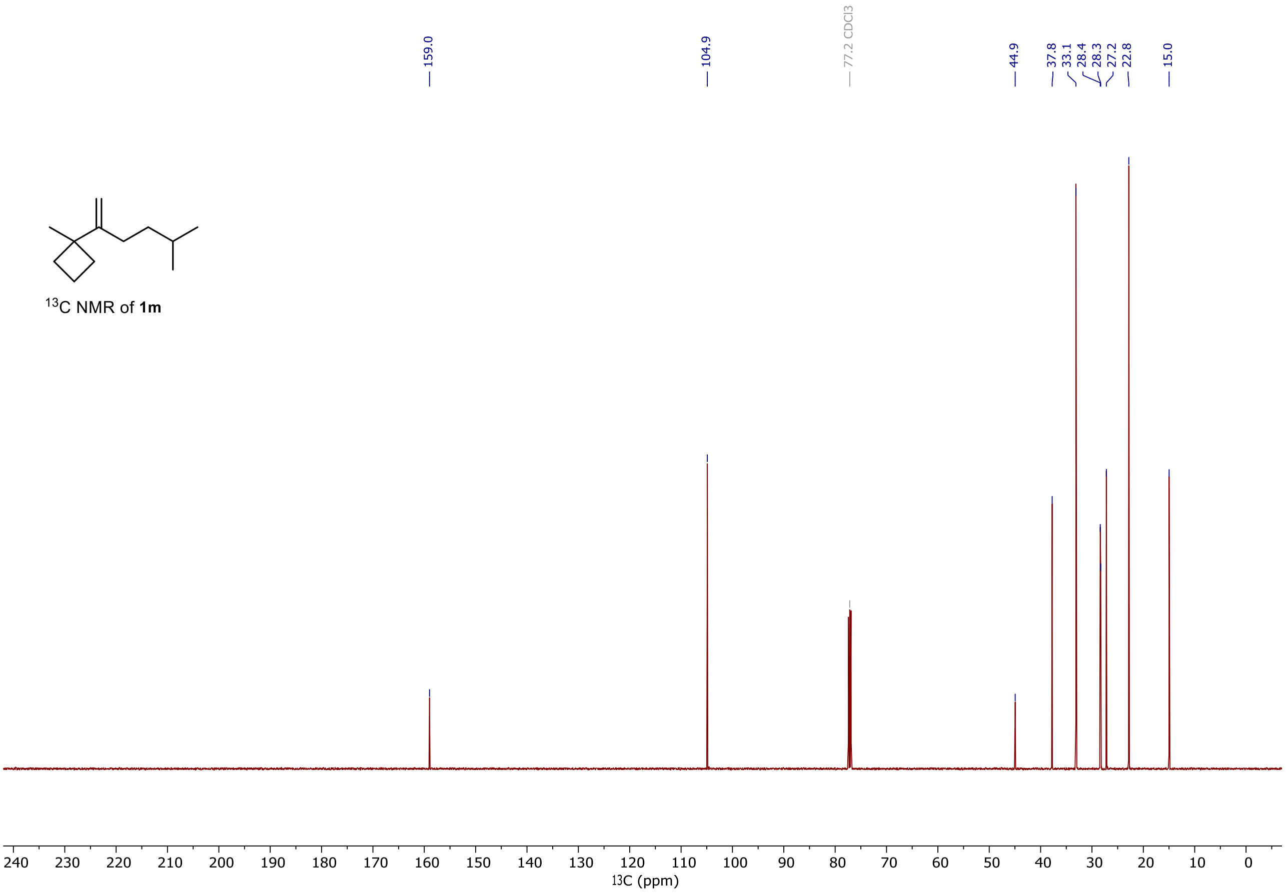

^{13}C NMR of **1m**

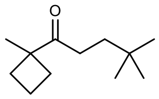

¹H NMR of **S12n**

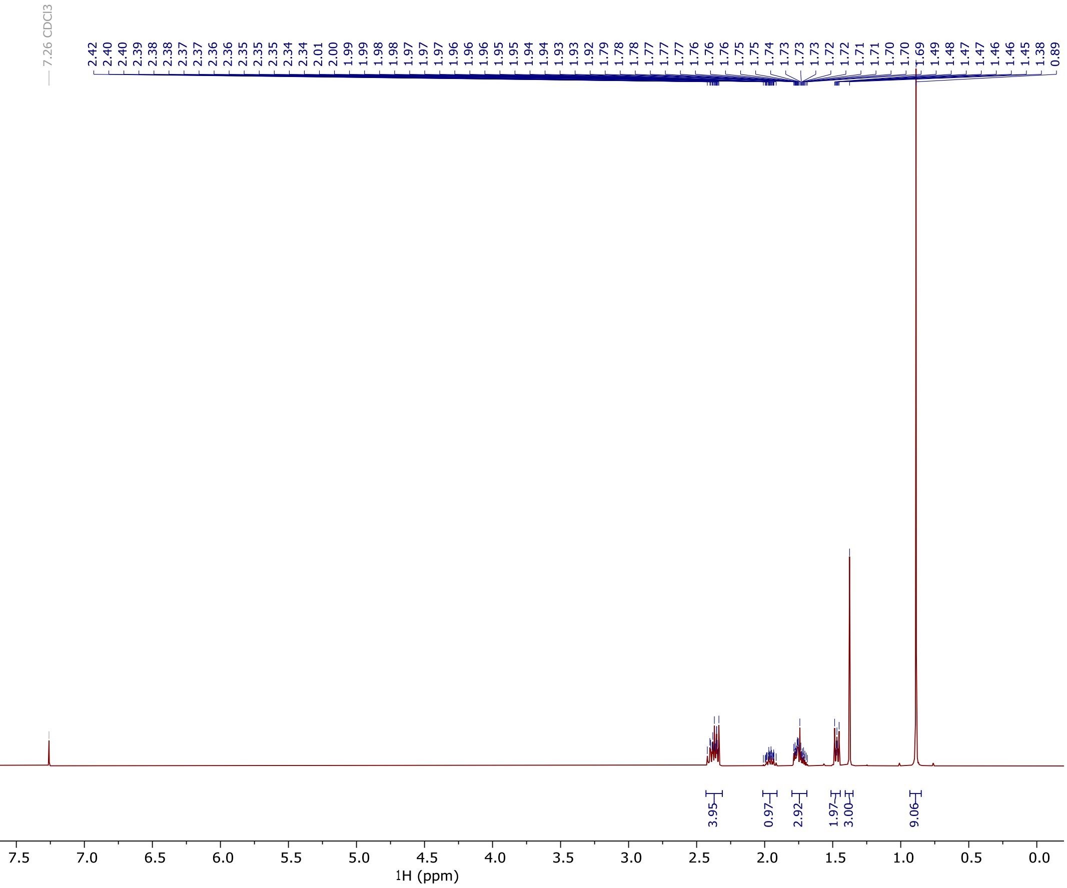

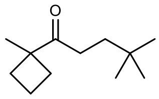

¹³C NMR of **S12n**

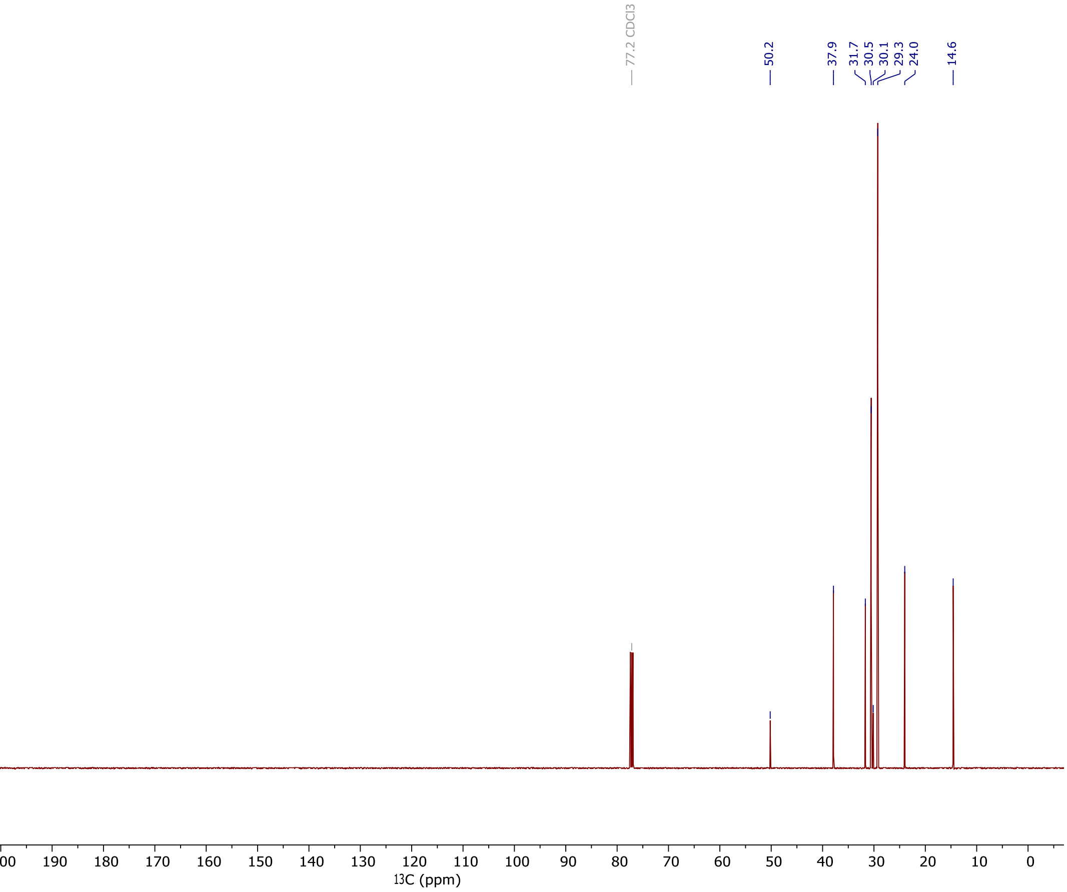

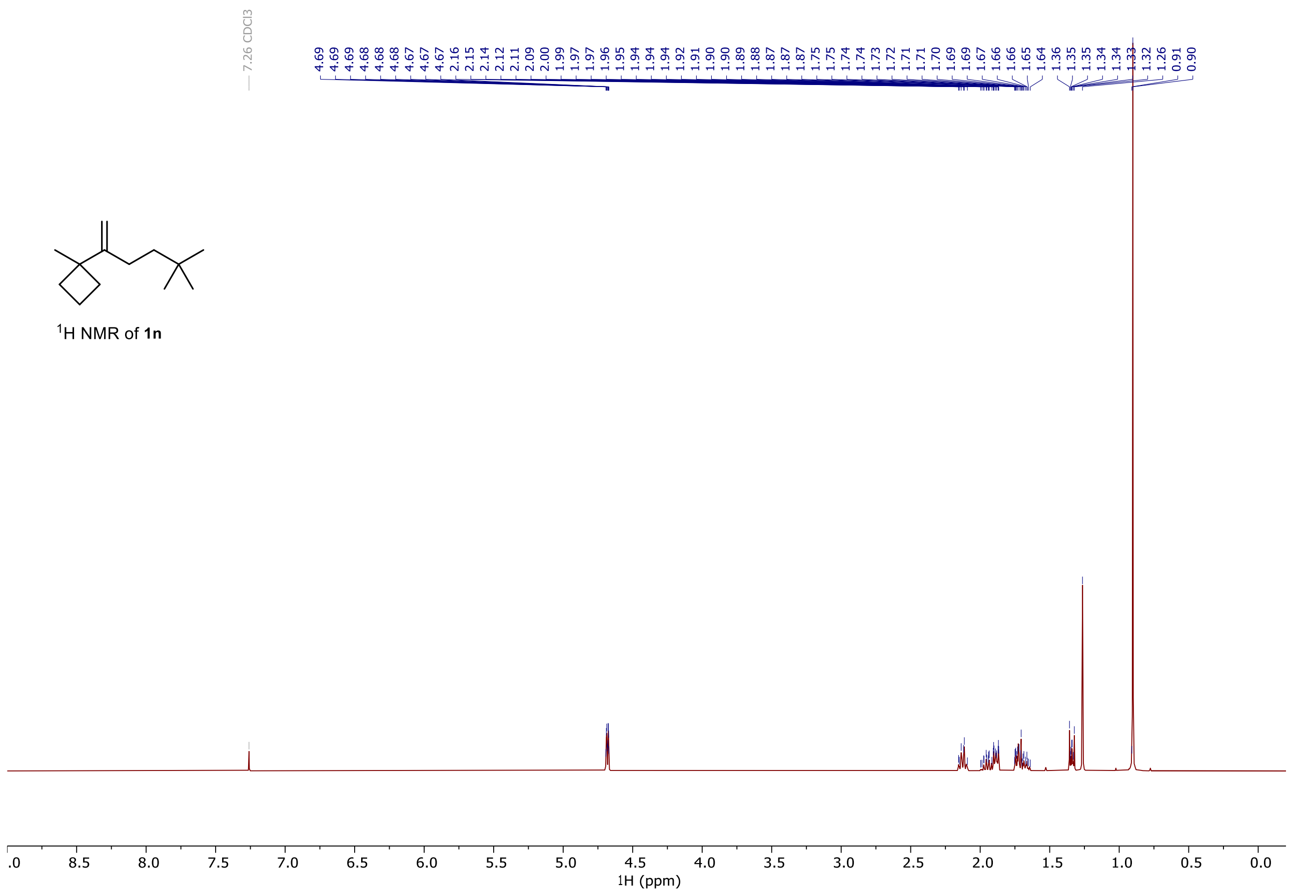
7.26 CDCl3
4.69
4.69
4.69
4.68
4.68
4.67
4.67
4.67
2.16
2.15
2.14
2.12
2.11
2.09
2.00
1.99
1.97
1.97
1.96
1.95
1.94
1.94
1.94
1.92
1.91
1.90
1.90
1.89
1.88
1.87
1.87
1.87
1.75
1.75
1.74
1.74
1.73
1.72
1.71
1.71
1.70
1.69
1.69
1.67
1.66
1.66
1.65
1.64
1.36
1.35
1.35
1.34
1.34
1.33
1.32
1.26
0.91
0.90
^{1}H NMR of **1n**
1H (ppm)

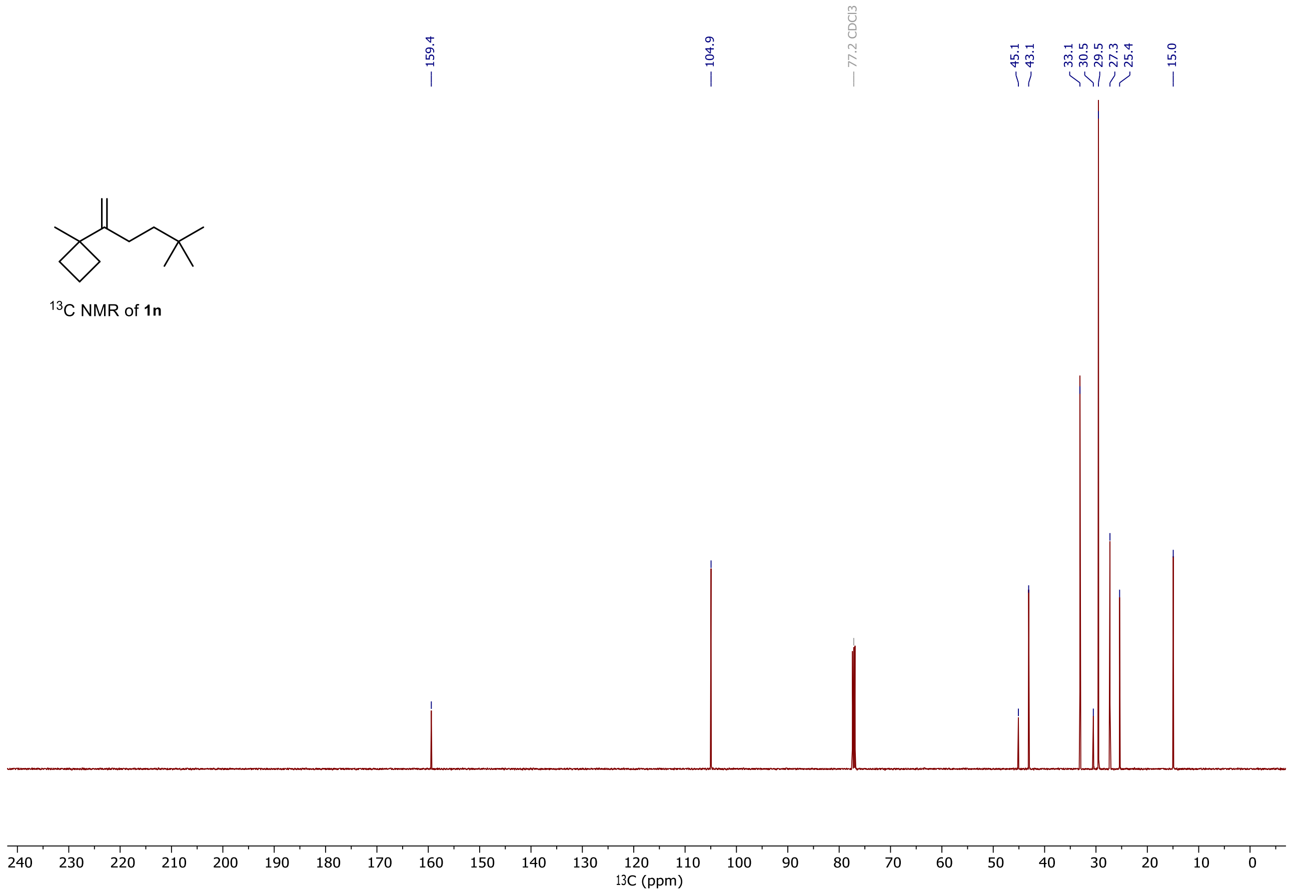
159.4
104.9
77.2 CDCl3
45.1
43.1
33.1
30.5
29.5
27.3
25.4
15.0
^{13}C NMR of **1n**
13C (ppm)

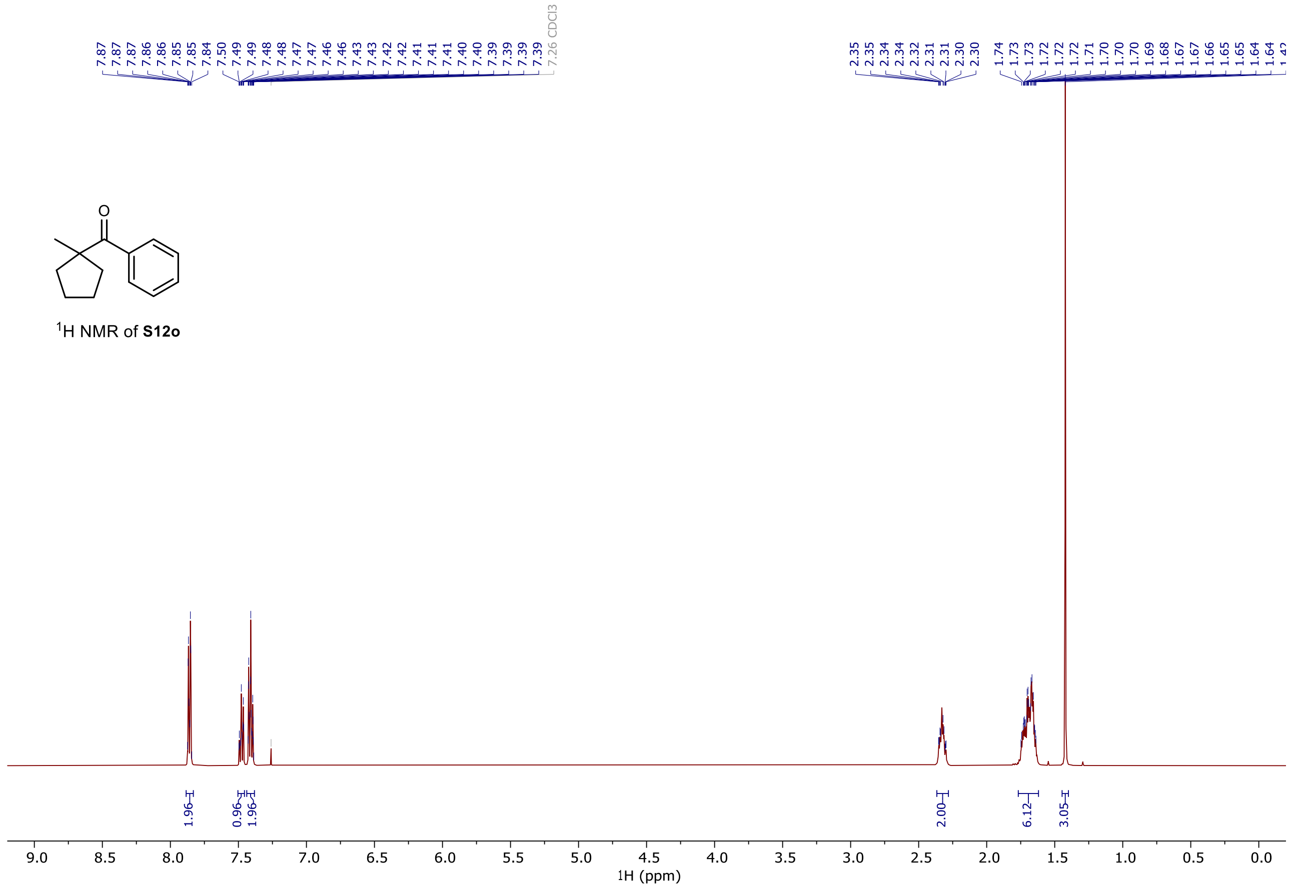
1H NMR of S12o
1H (ppm)

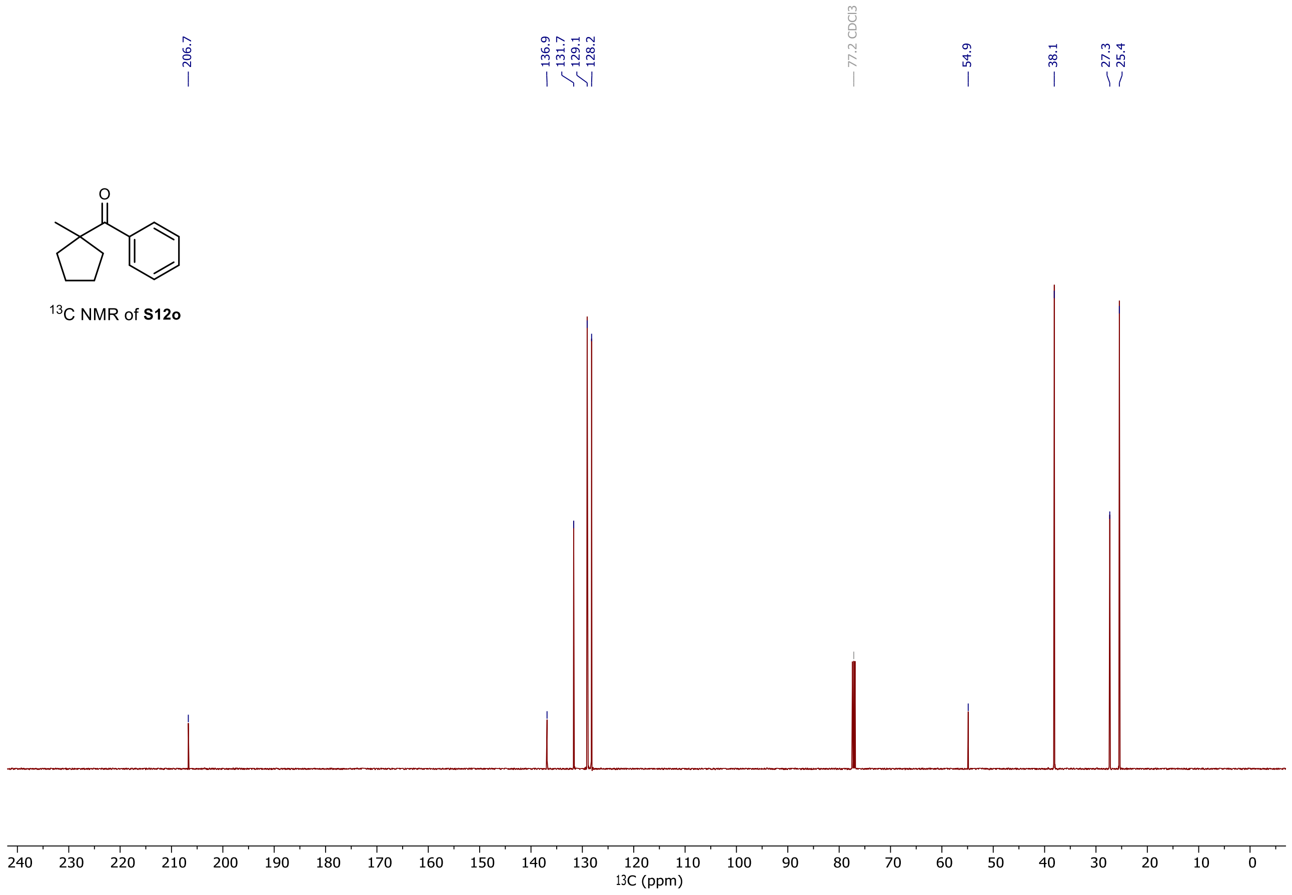
13C NMR of S12o
13C (ppm)

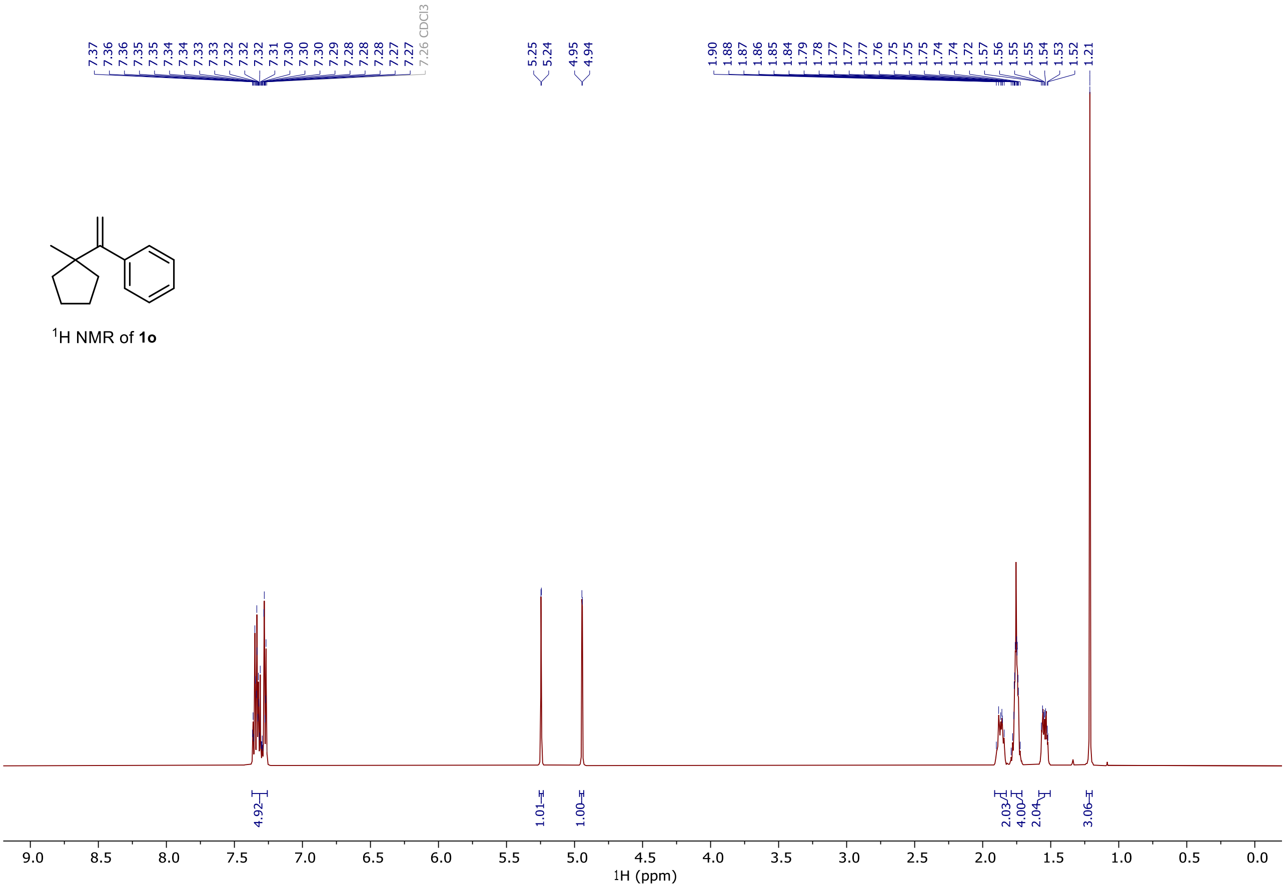

^{1}H NMR of **1o**

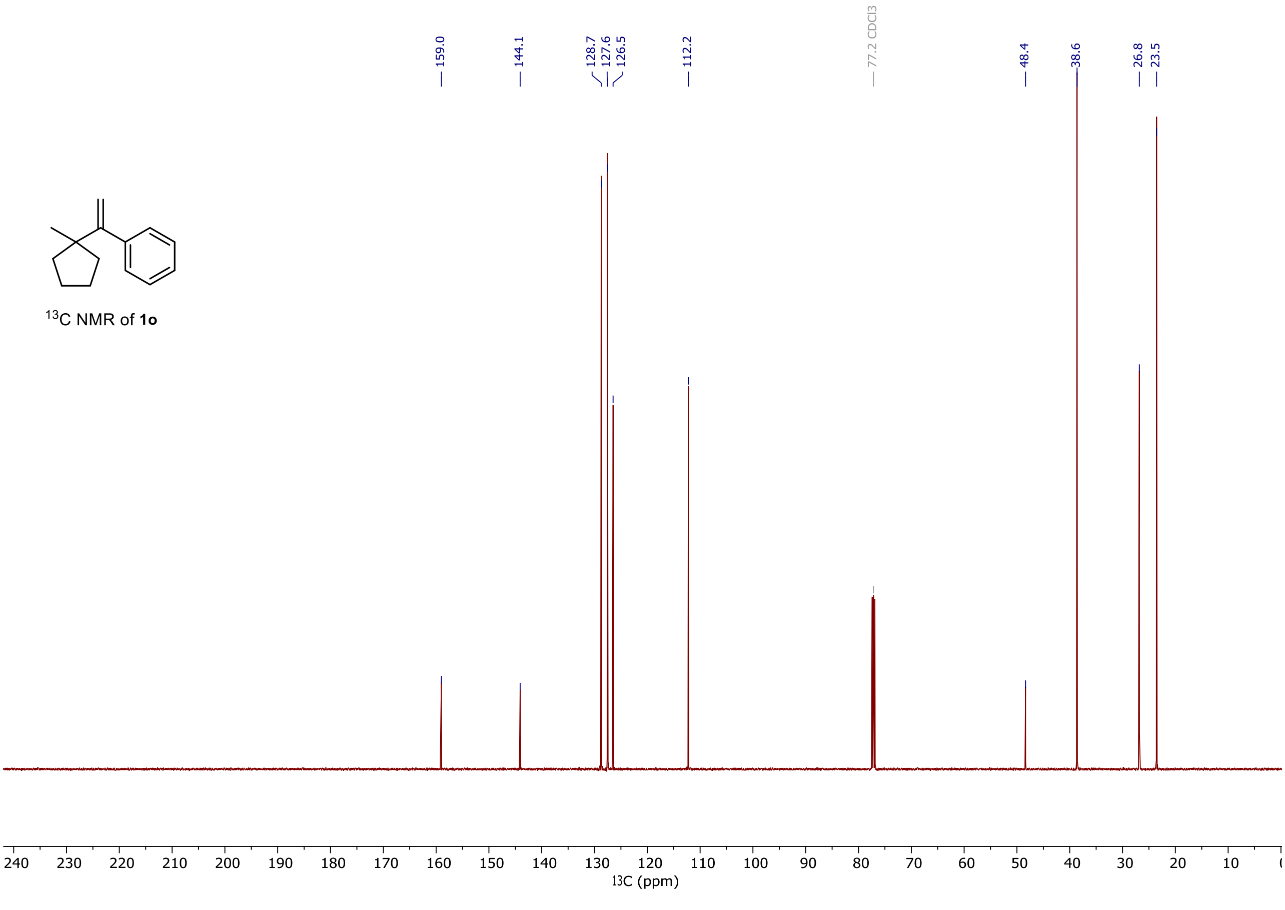

^{13}C NMR of **1o**

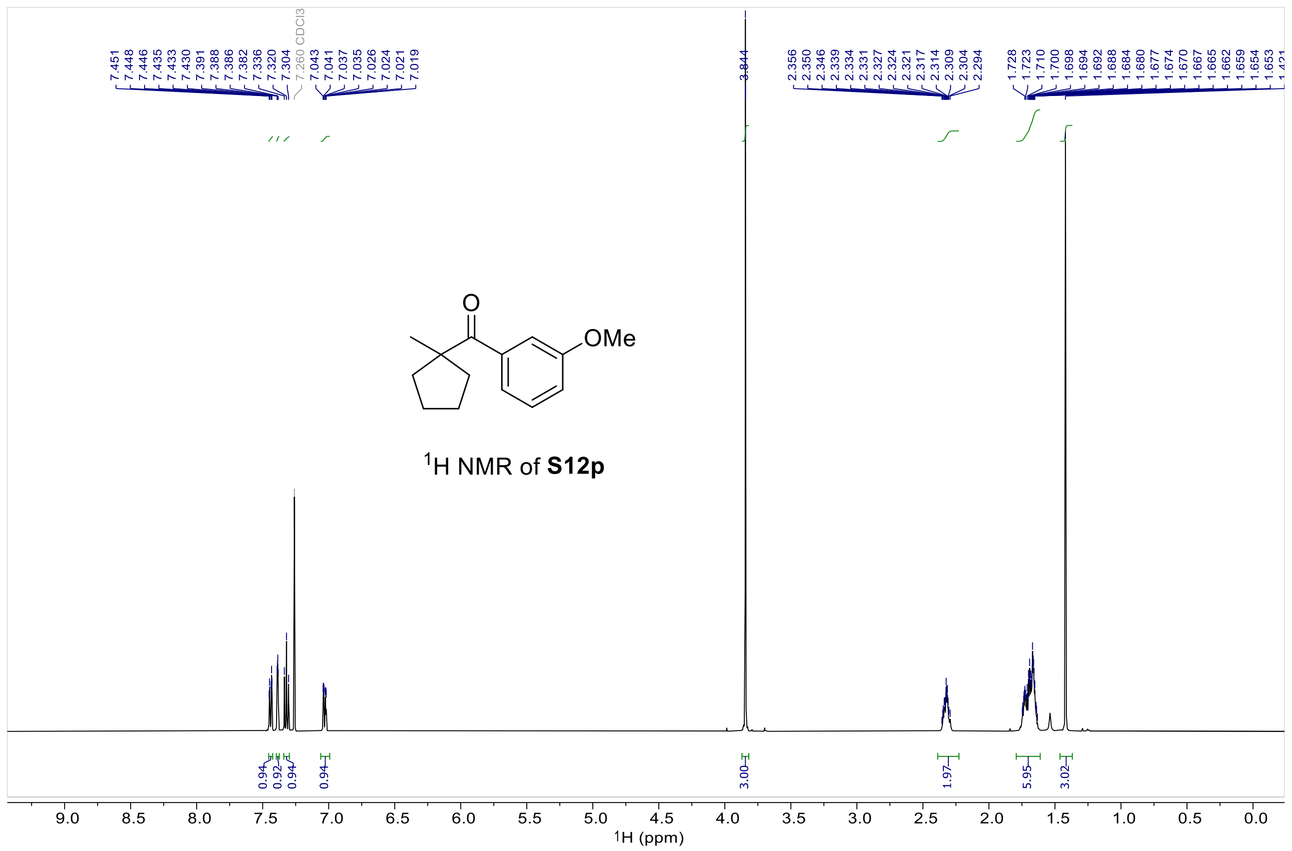
OMe
1H NMR of S12p
1H (ppm)

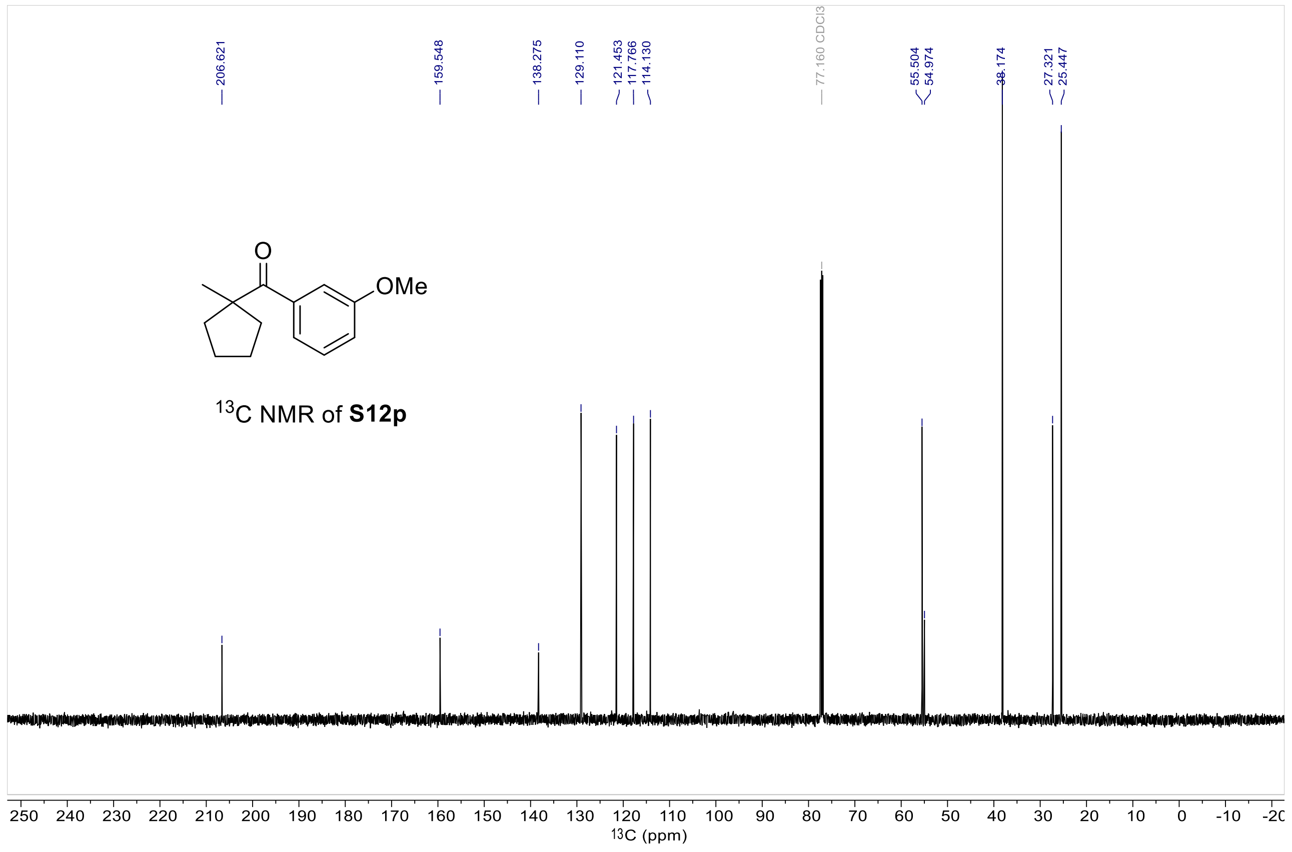
OMe
13C NMR of S12p
13C (ppm)

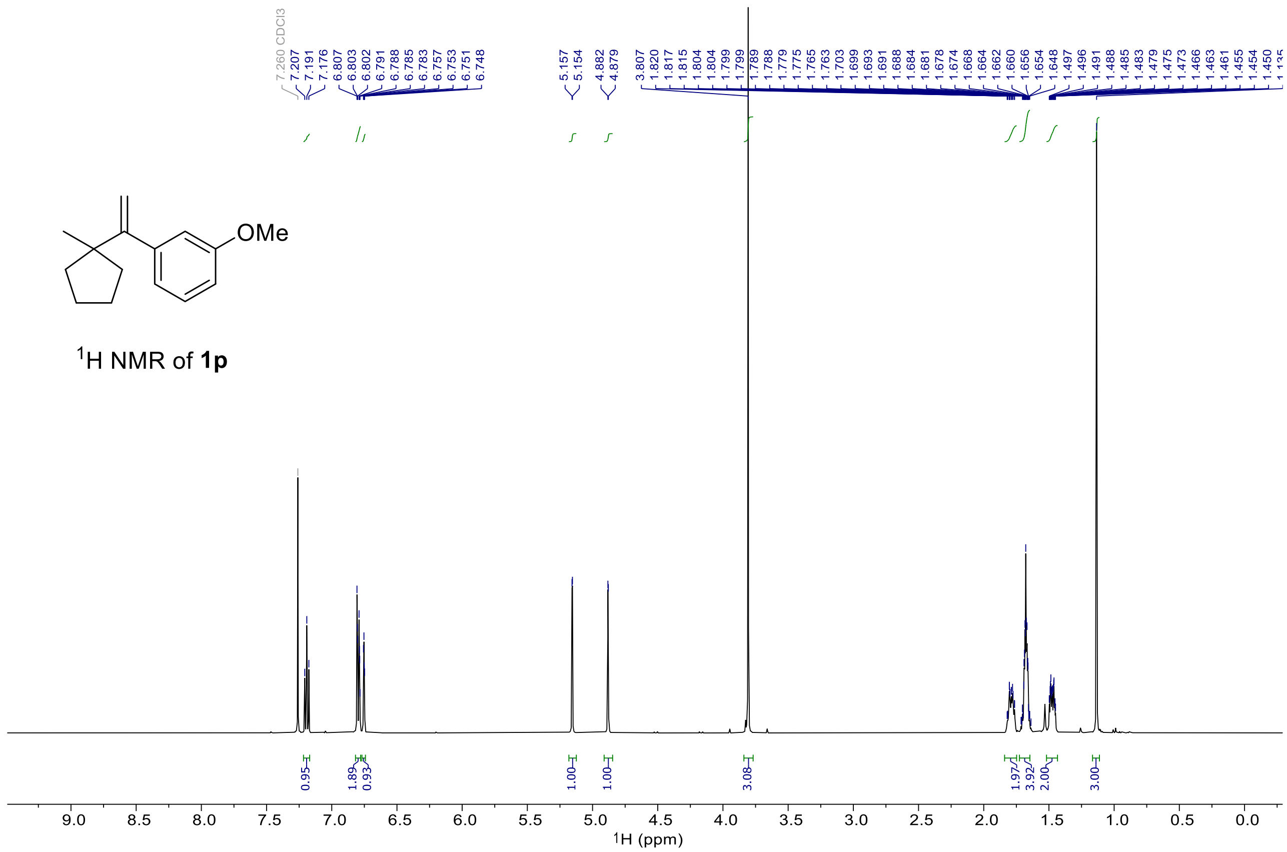
OMe
1H NMR of 1p
1H (ppm)

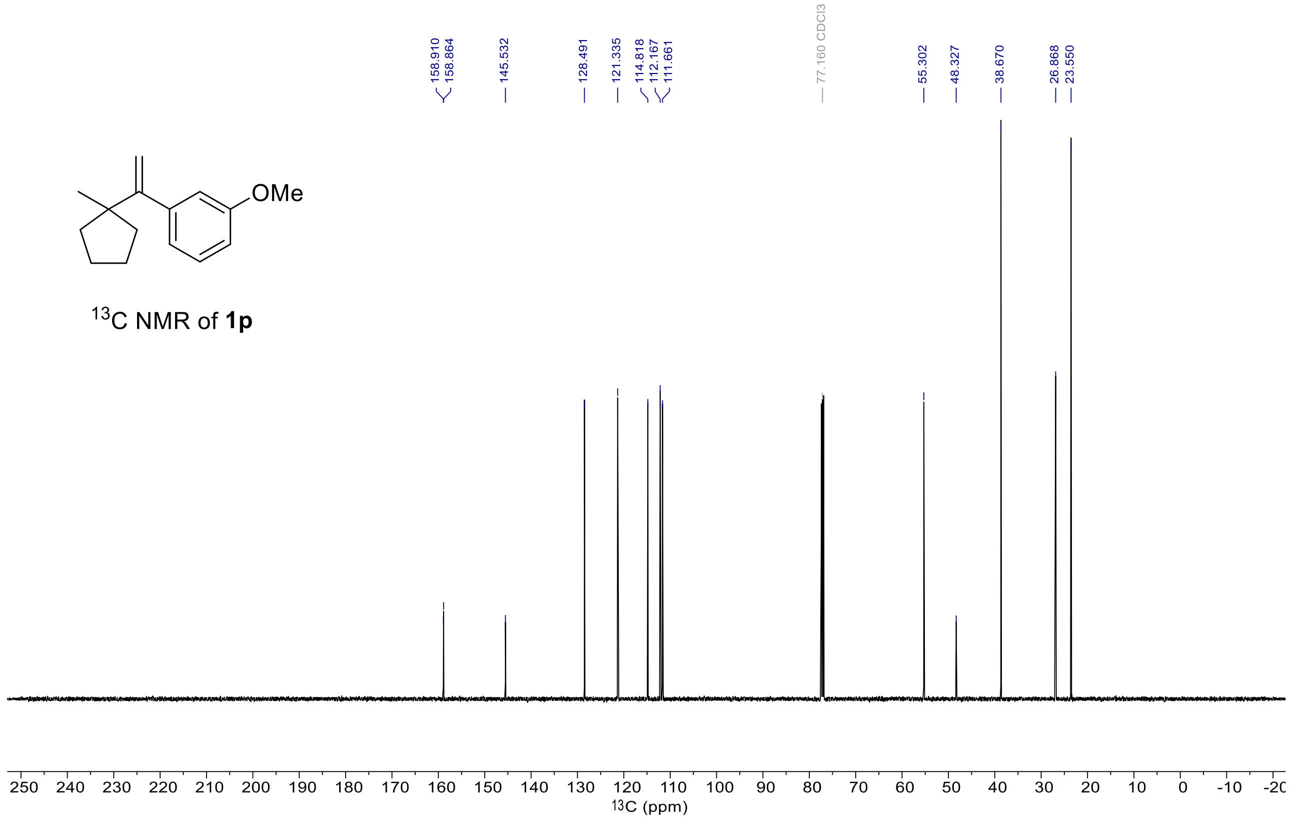
OMe
13C NMR of 1p
158.910
158.864
145.532
128.491
121.335
114.818
112.167
111.661
77.160 CDCl3
55.302
48.327
38.670
26.868
23.550
13C (ppm)

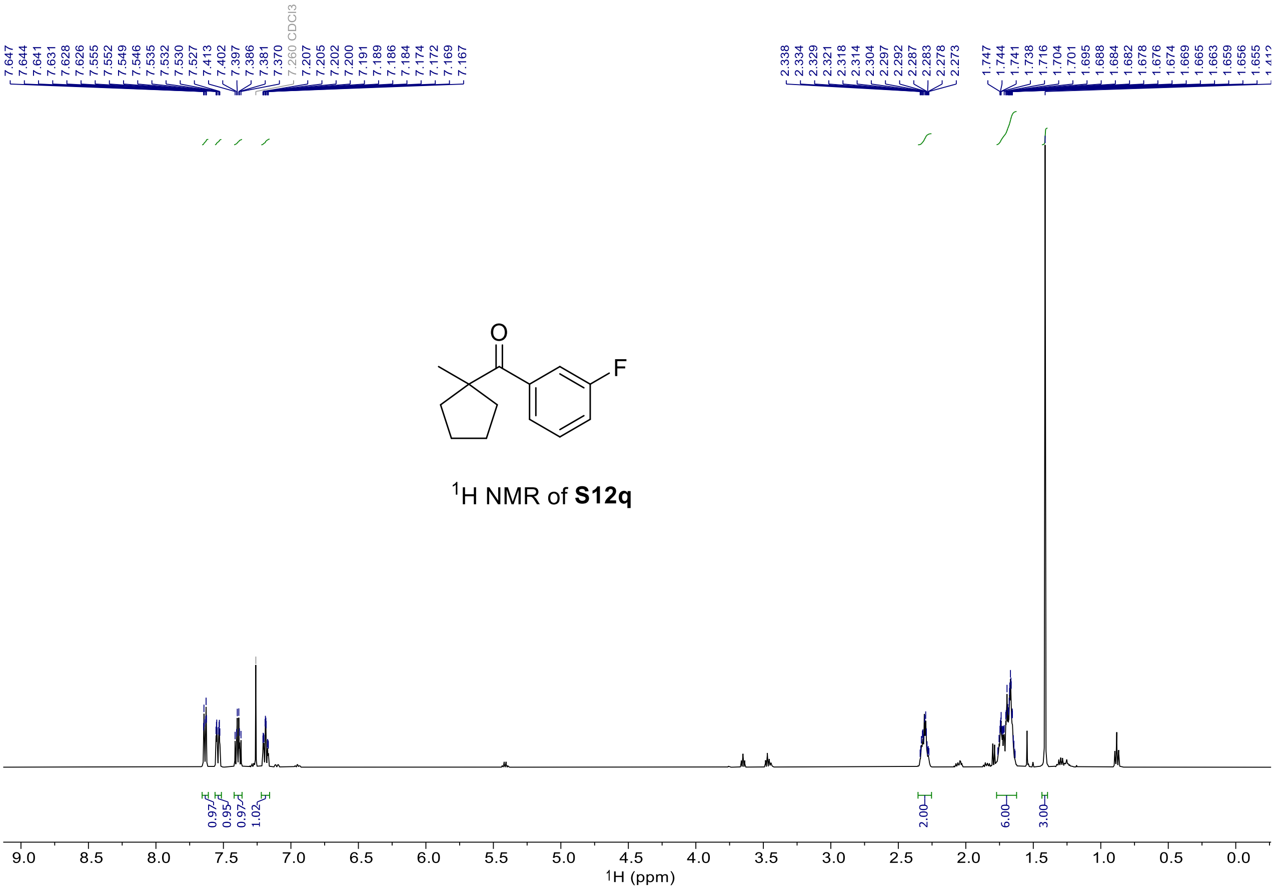
7.647 7.644 7.641 7.631 7.628 7.626 7.555 7.552 7.549 7.546 7.535 7.532 7.530 7.527 7.413 7.402 7.397 7.386 7.381 7.370 7.260 CDCl3 7.207 7.205 7.202 7.200 7.191 7.189 7.186 7.184 7.174 7.172 7.169 7.167
2.338 2.334 2.329 2.321 2.318 2.314 2.304 2.297 2.292 2.287 2.283 2.278 2.273
1.747 1.744 1.741 1.738 1.716 1.704 1.701 1.695 1.688 1.684 1.682 1.678 1.676 1.674 1.669 1.665 1.663 1.659 1.656 1.655
O
F
1H NMR of S12q
0.97 0.95 0.97 1.02 2.00 6.00 3.00
9.0 8.5 8.0 7.5 7.0 6.5 6.0 5.5 5.0 4.5 4.0 3.5 3.0 2.5 2.0 1.5 1.0 0.5 0.0
1H (ppm)

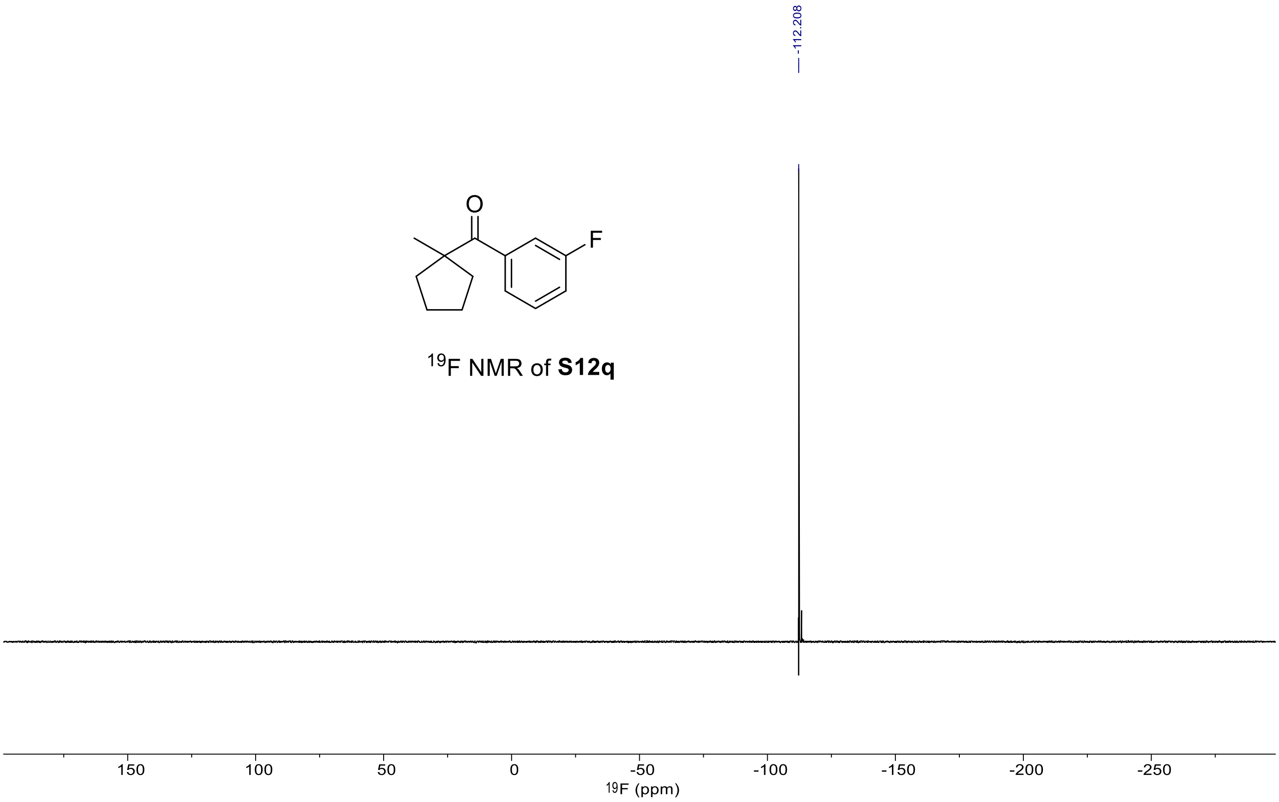
-112.208
O
F
19F NMR of S12q
150 100 50 0 -50 -100 -150 -200 -250
19F (ppm)

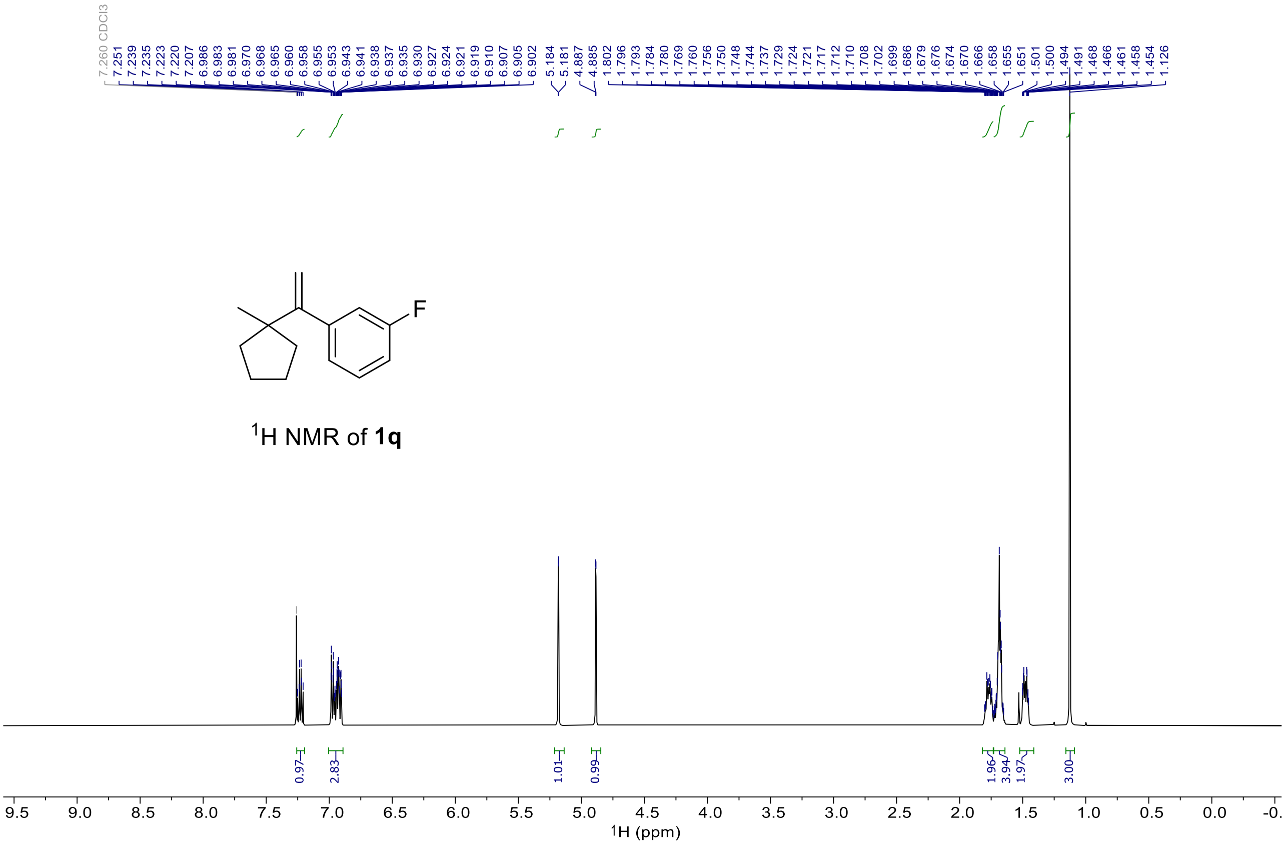

^{1}H NMR of **1q**

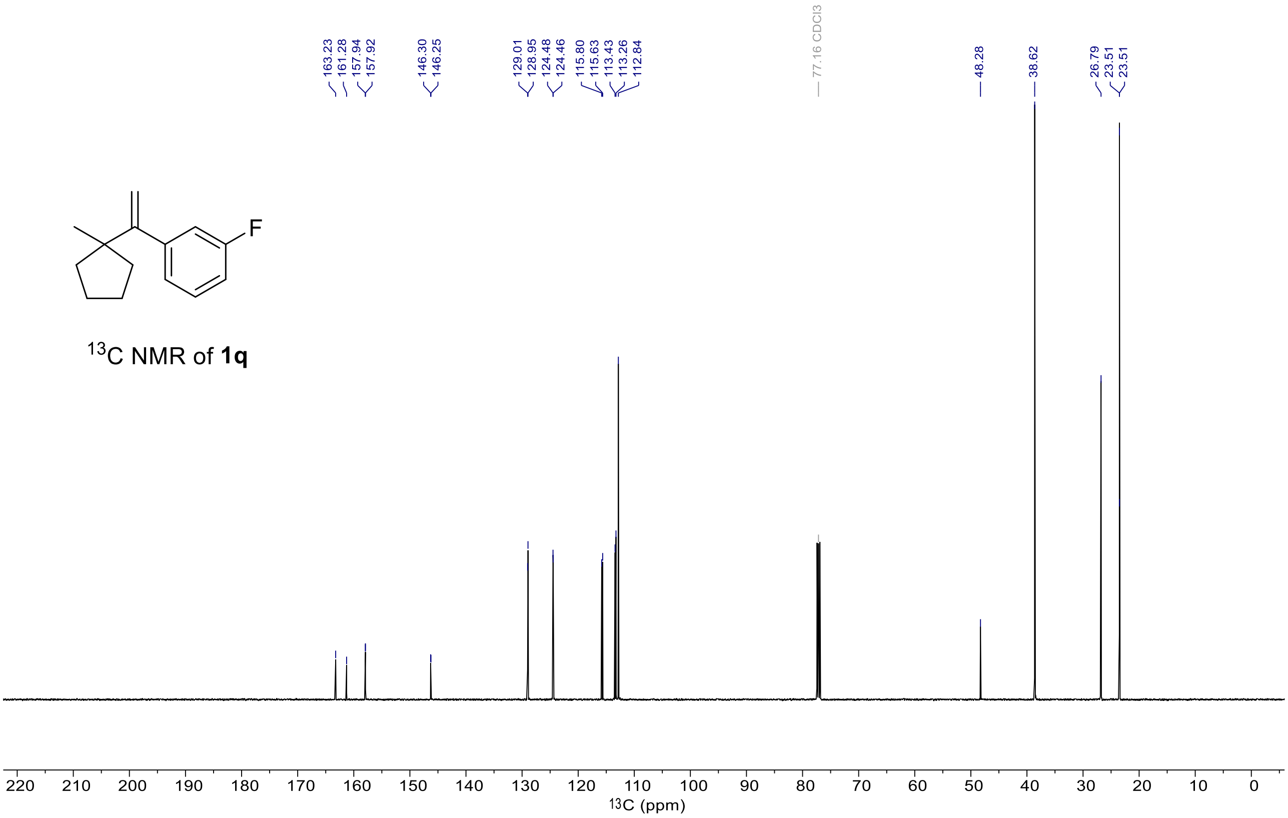

^{13}C NMR of **1q**

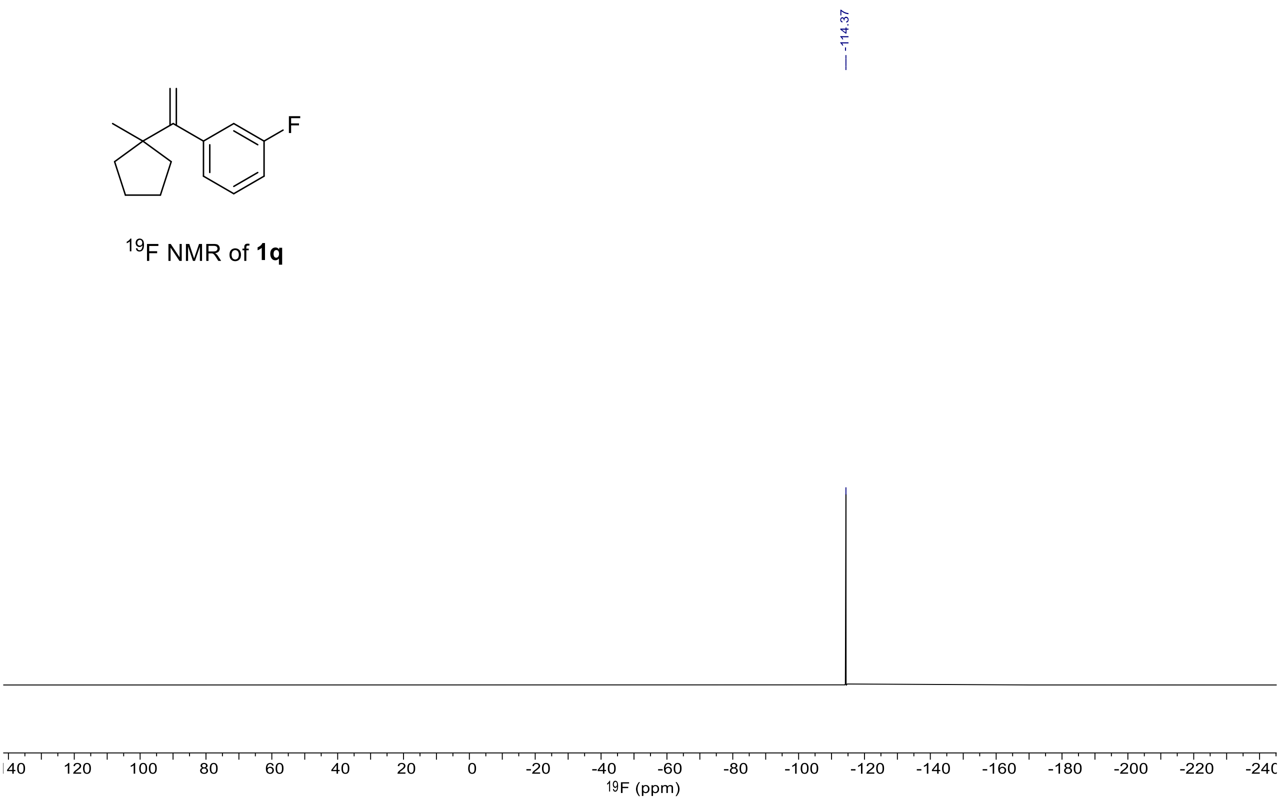

F
19F NMR of 1q
-114.37
19F (ppm)

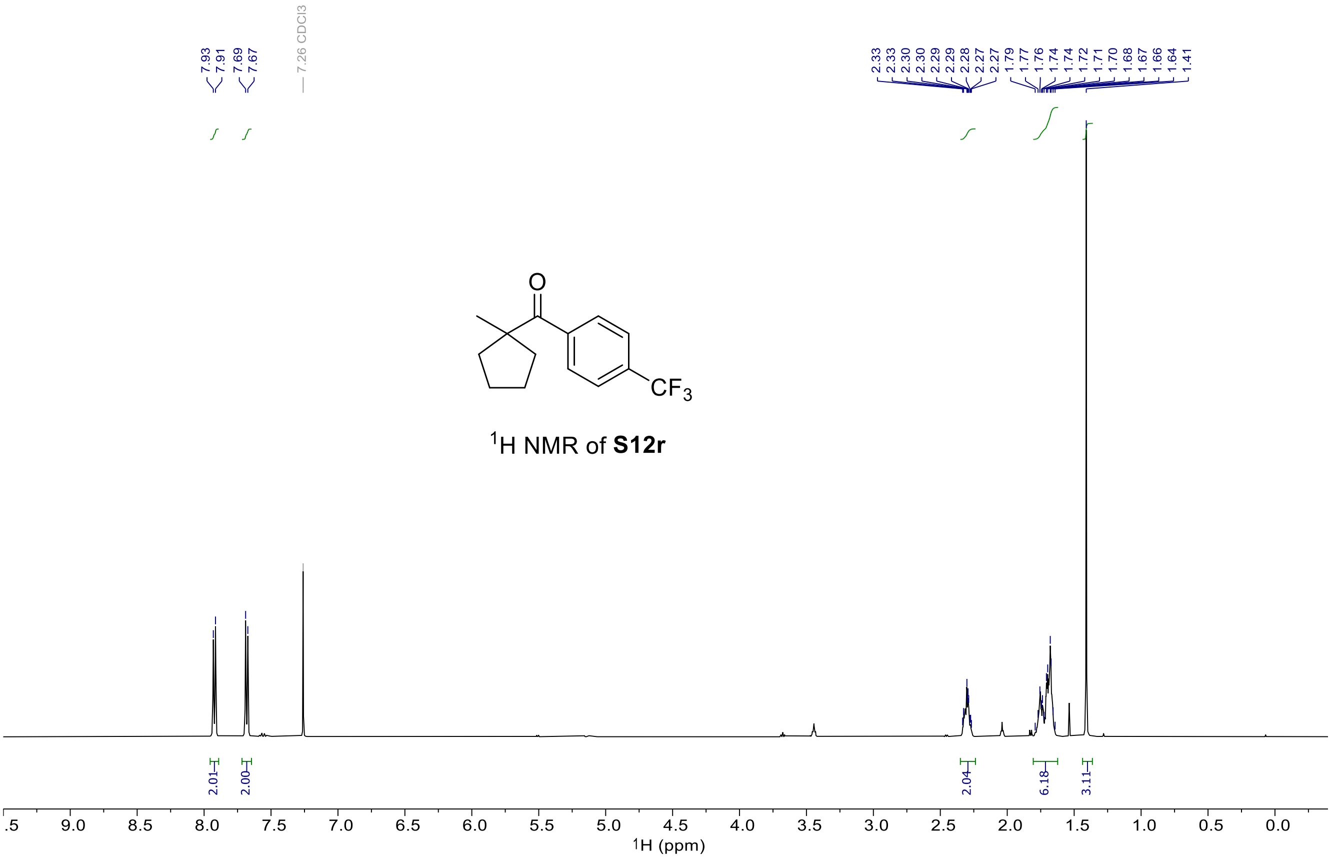

7.93
7.91
7.69
7.67
7.26 CDCl3
2.33
2.33
2.30
2.30
2.29
2.29
2.28
2.27
2.27
1.79
1.77
1.76
1.74
1.74
1.72
1.71
1.70
1.68
1.67
1.66
1.64
1.41
O
CF3
1H NMR of S12r
2.01
2.00
2.04
6.18
3.11
1H (ppm)

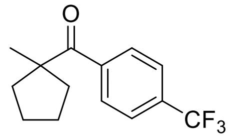

^{19}F NMR of **S12r**

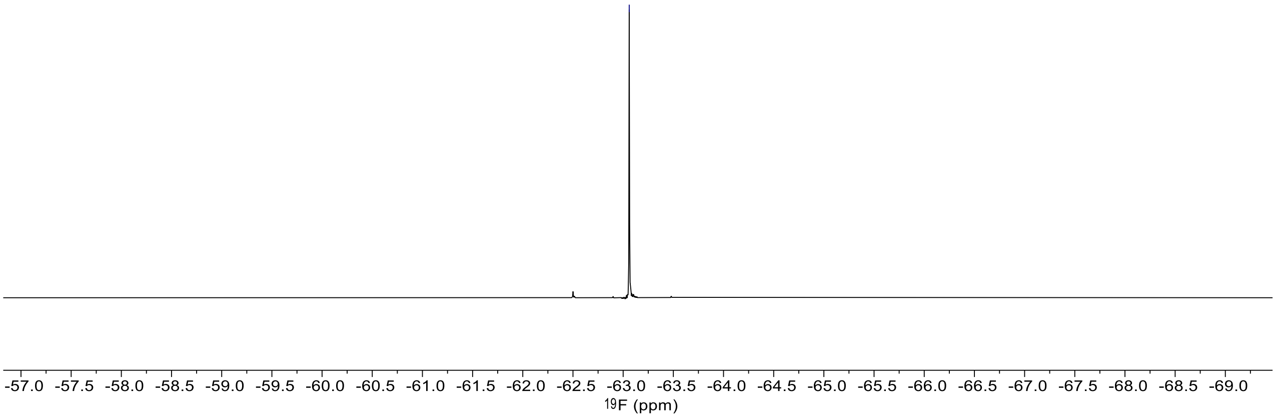

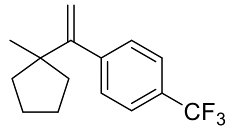

^{1}H NMR of **1r**

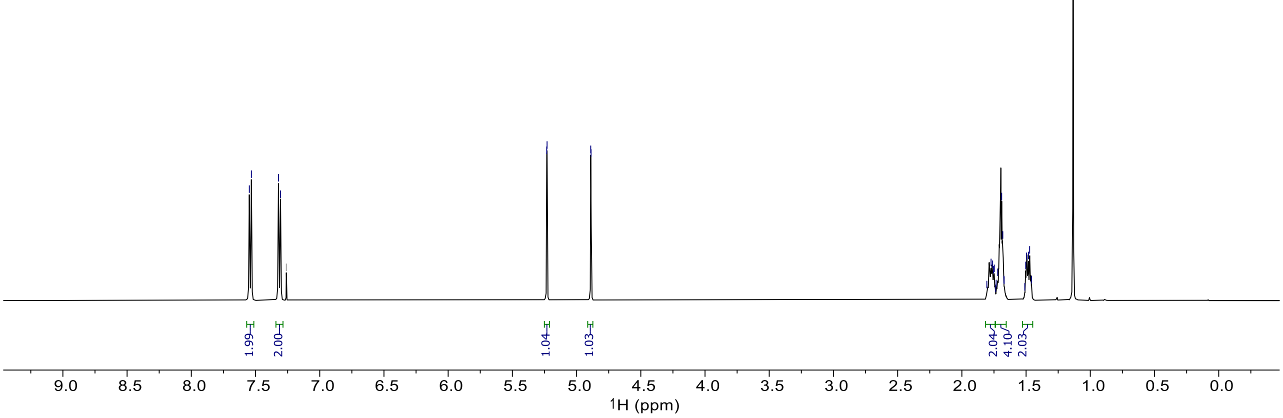

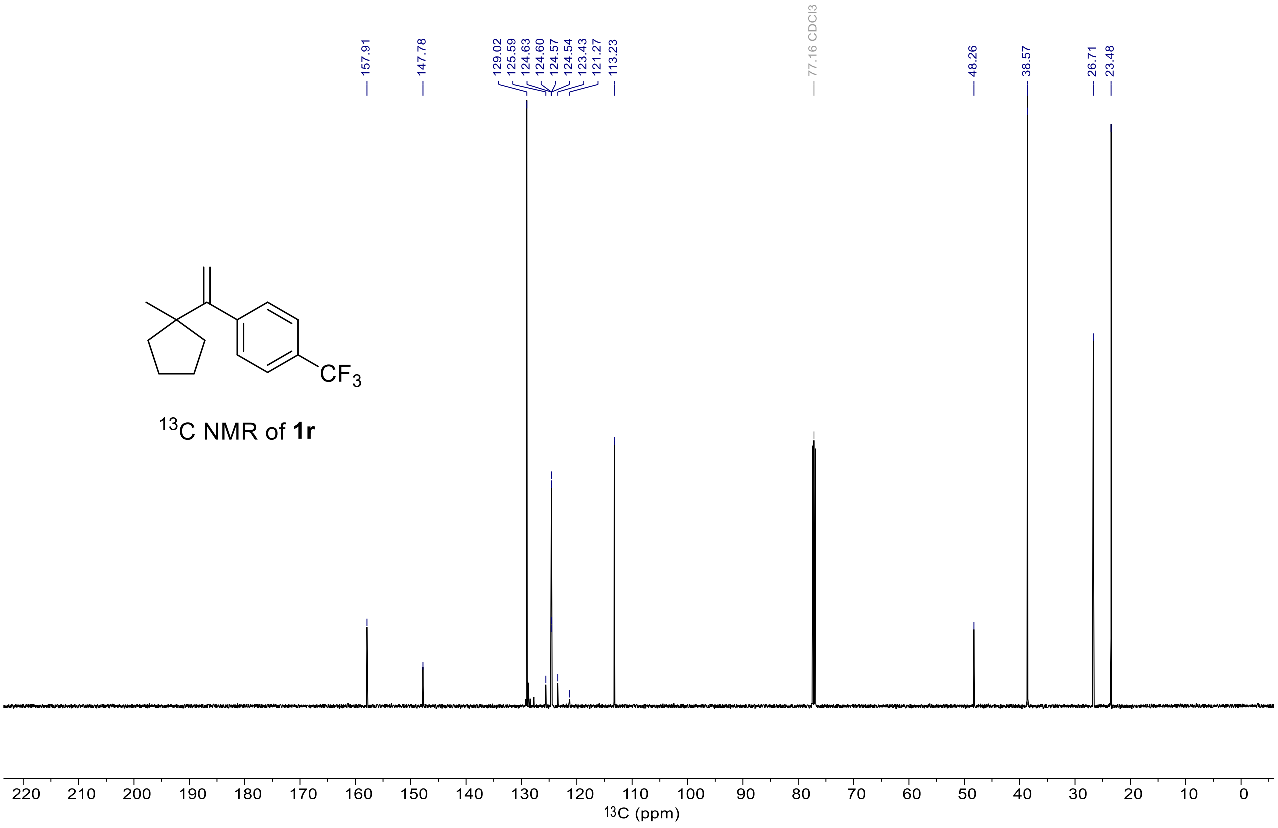
157.91
147.78
129.02
125.59
124.63
124.60
124.57
124.54
123.43
121.27
113.23
77.16 CDCl3
48.26
38.57
26.71
23.48
CF3
13C NMR of 1r
220 210 200 190 180 170 160 150 140 130 120 110 100 90 80 70 60 50 40 30 20 10 0
13C (ppm)

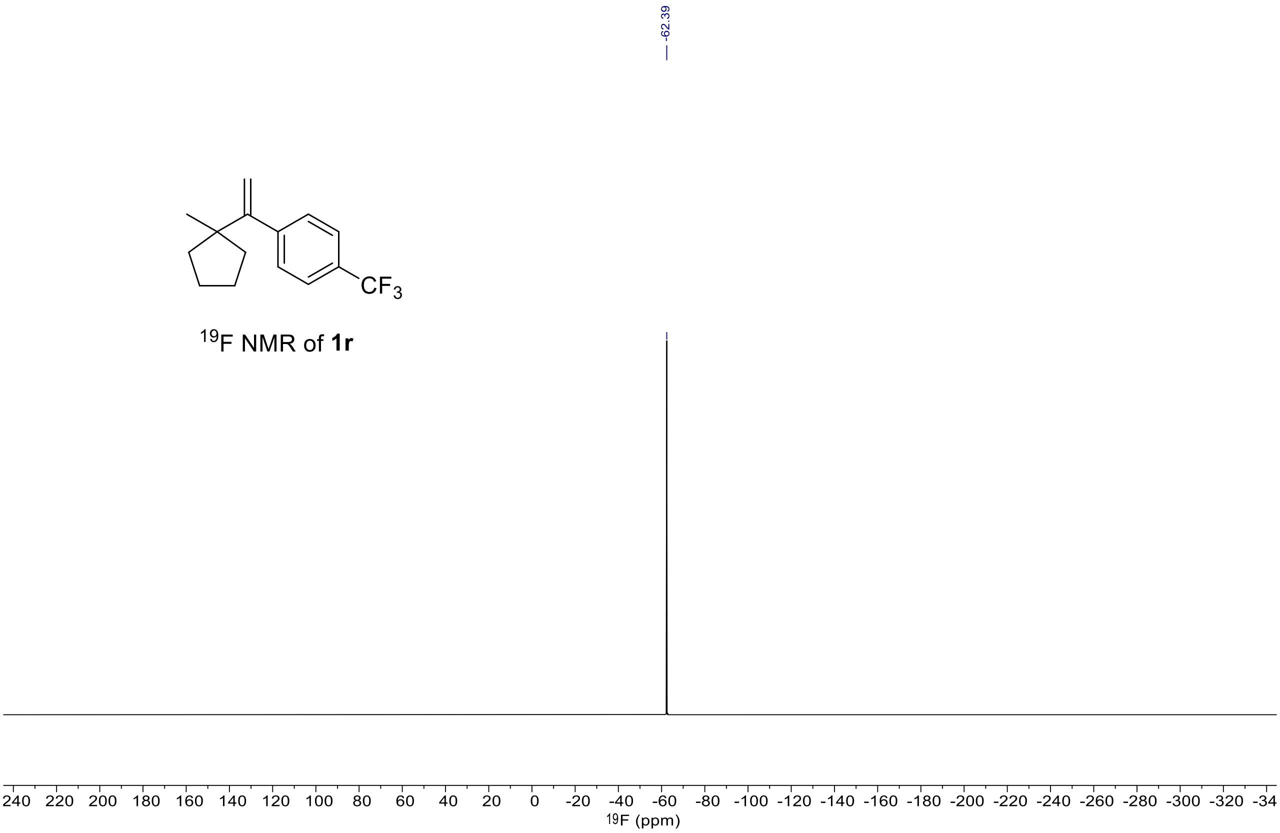
-62.39
CF3
19F NMR of 1r
240 220 200 180 160 140 120 100 80 60 40 20 0 -20 -40 -60 -80 -100 -120 -140 -160 -180 -200 -220 -240 -260 -280 -300 -320 -34
19F (ppm)

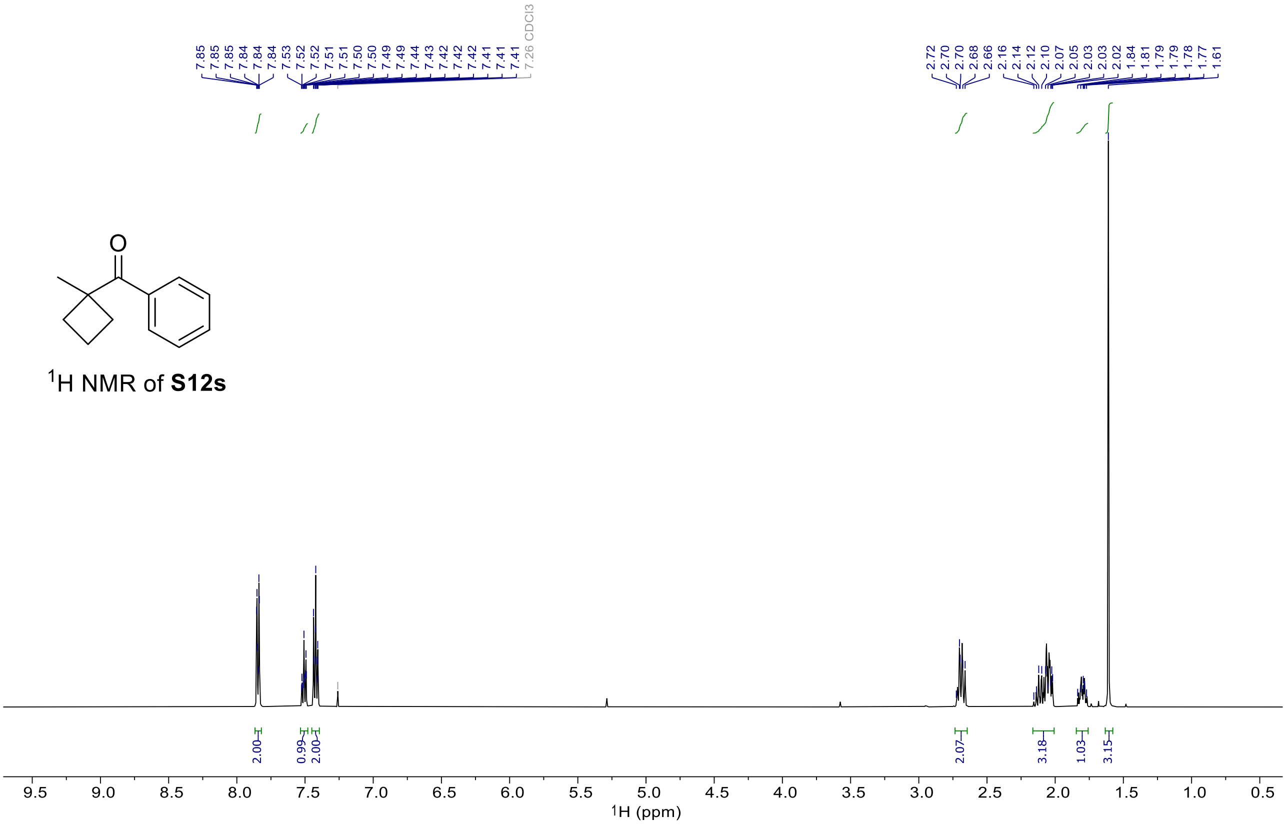
7.85
7.85
7.84
7.84
7.53
7.52
7.52
7.51
7.51
7.50
7.49
7.49
7.44
7.43
7.42
7.42
7.41
7.41
7.26 CDCl3
2.72
2.70
2.70
2.68
2.66
2.16
2.14
2.12
2.10
2.07
2.05
2.03
2.03
2.02
1.84
1.81
1.79
1.78
1.77
1.61
O
1H NMR of S12s
2.00
0.99
2.00
2.07
3.18
1.03
3.15
9.5 9.0 8.5 8.0 7.5 7.0 6.5 6.0 5.5 5.0 4.5 4.0 3.5 3.0 2.5 2.0 1.5 1.0 0.5
1H (ppm)

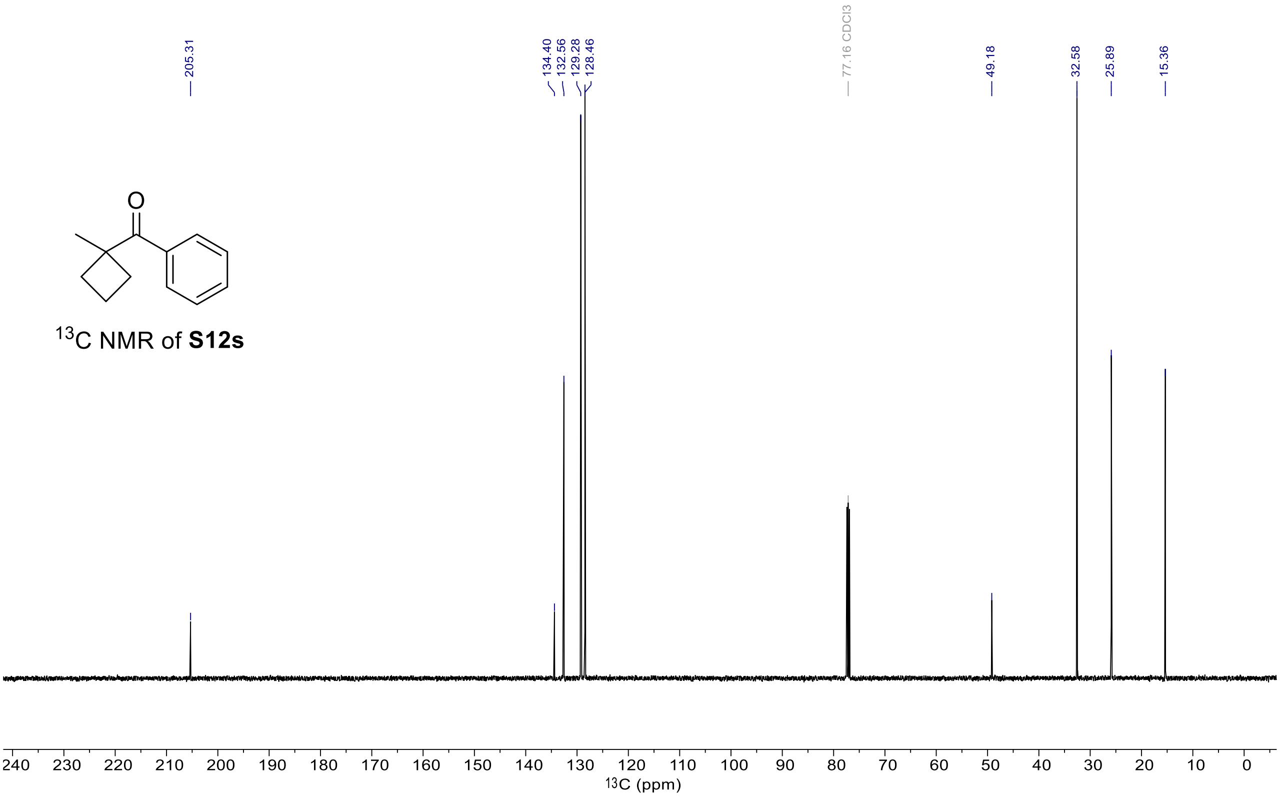
205.31
134.40
132.56
129.28
128.46
77.16 CDCl3
49.18
32.58
25.89
15.36
O
13C NMR of S12s
240 230 220 210 200 190 180 170 160 150 140 130 120 110 100 90 80 70 60 50 40 30 20 10 0
13C (ppm)

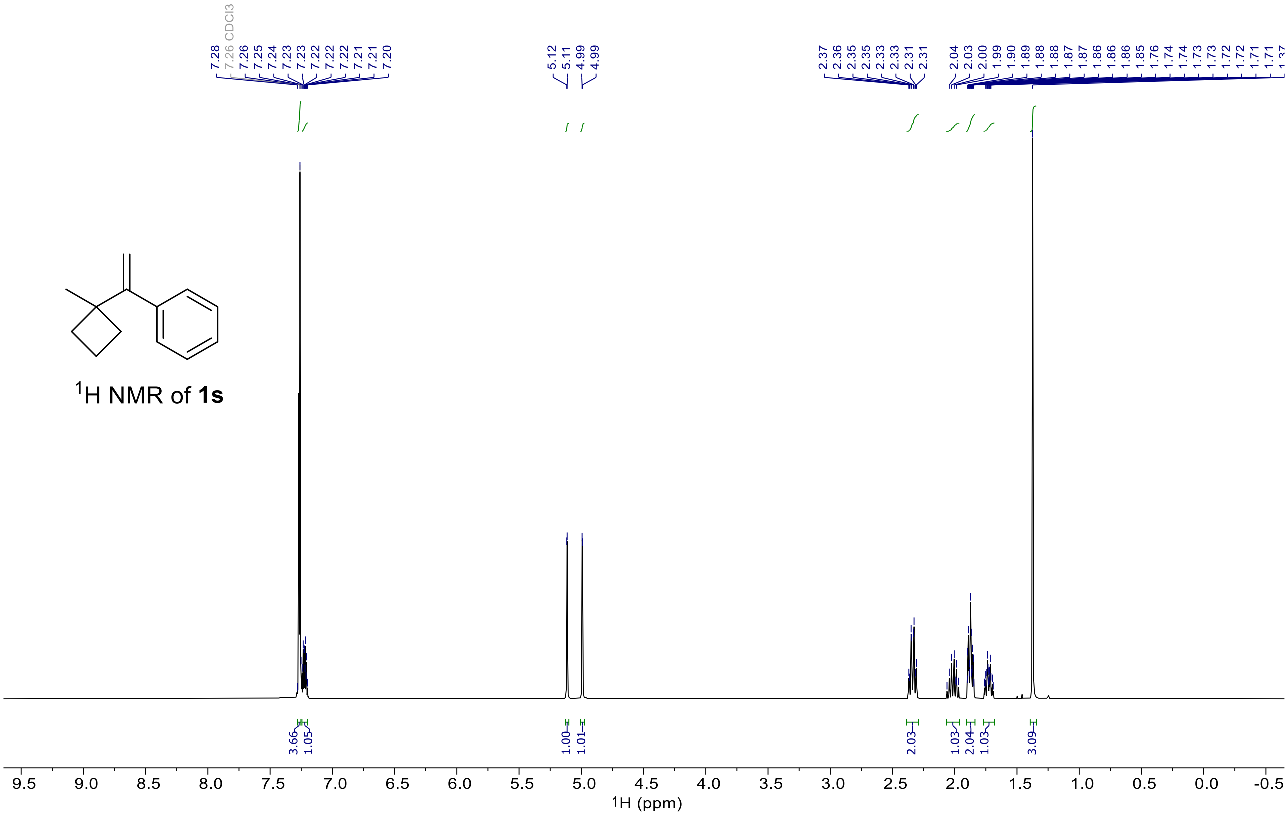

^{1}H NMR of **1s**

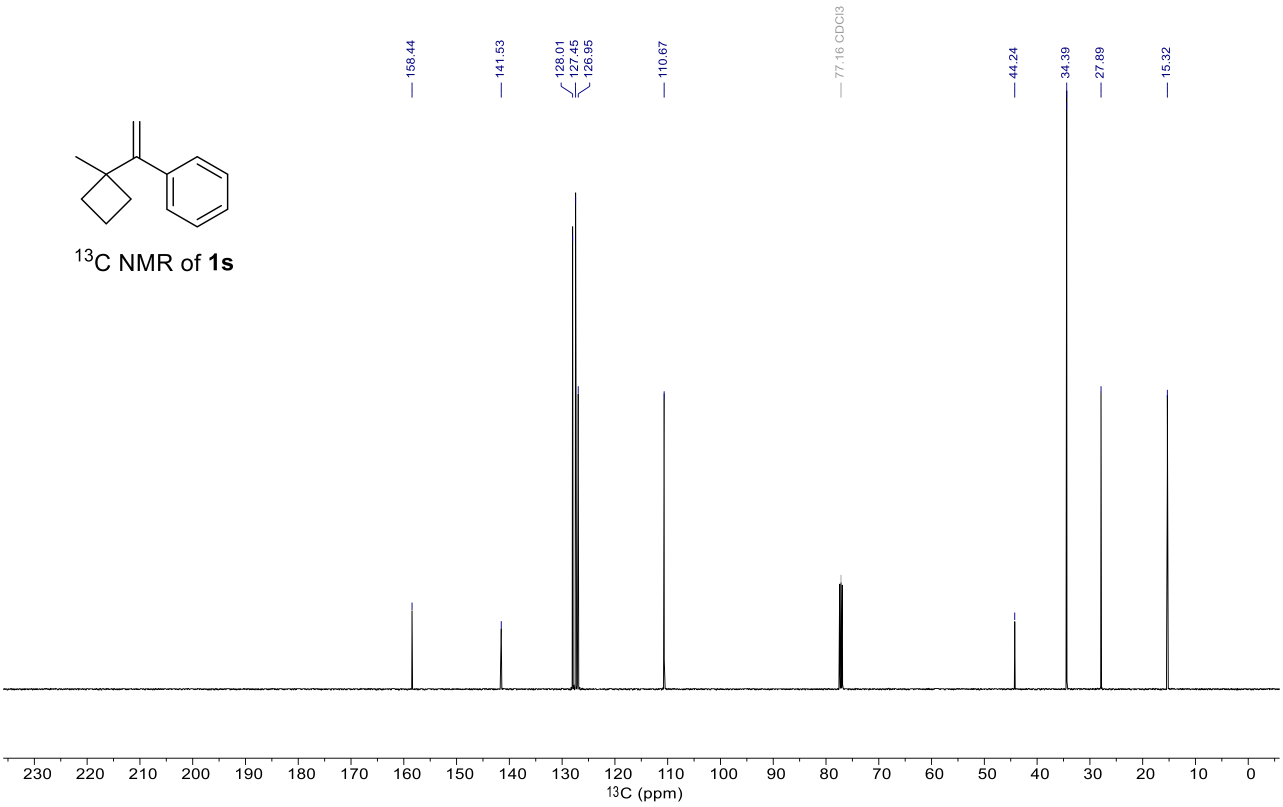

^{13}C NMR of **1s**

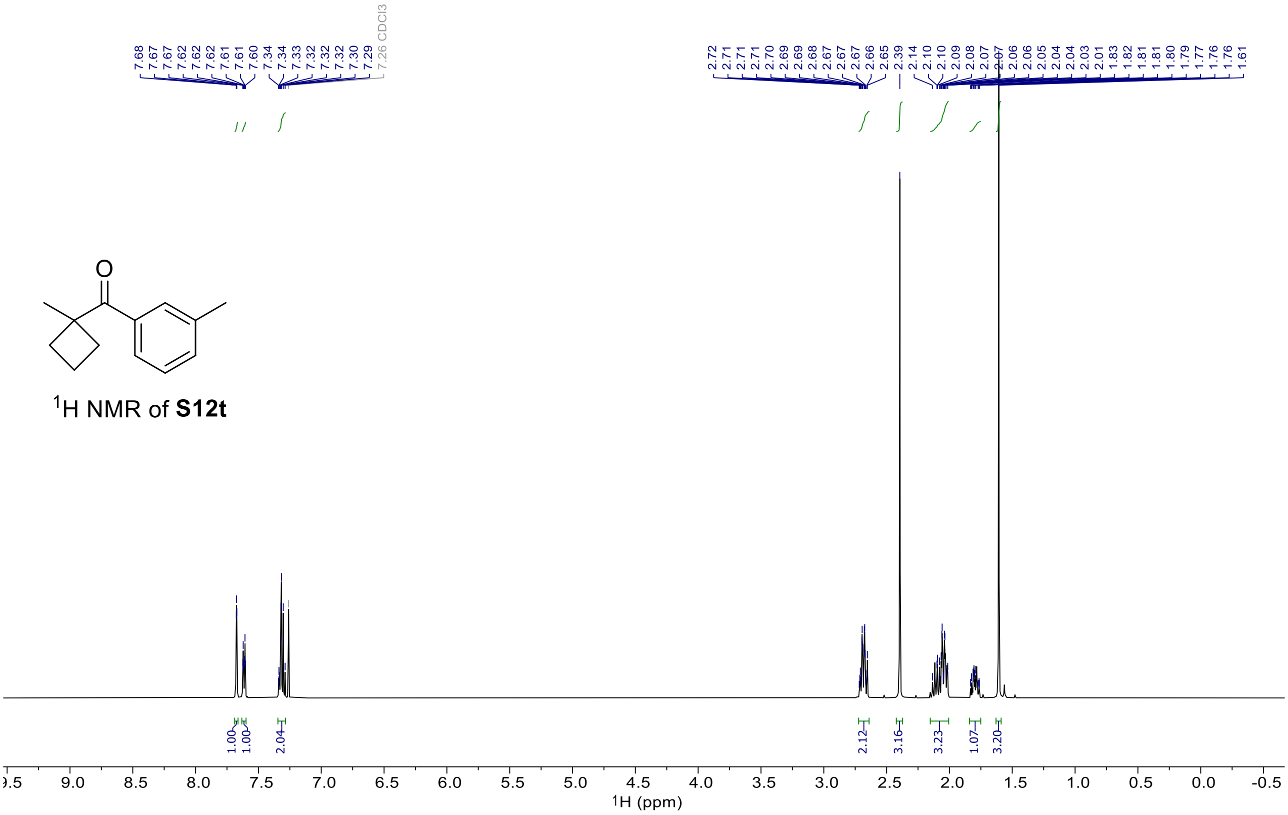

^{1}H NMR of **S12t**

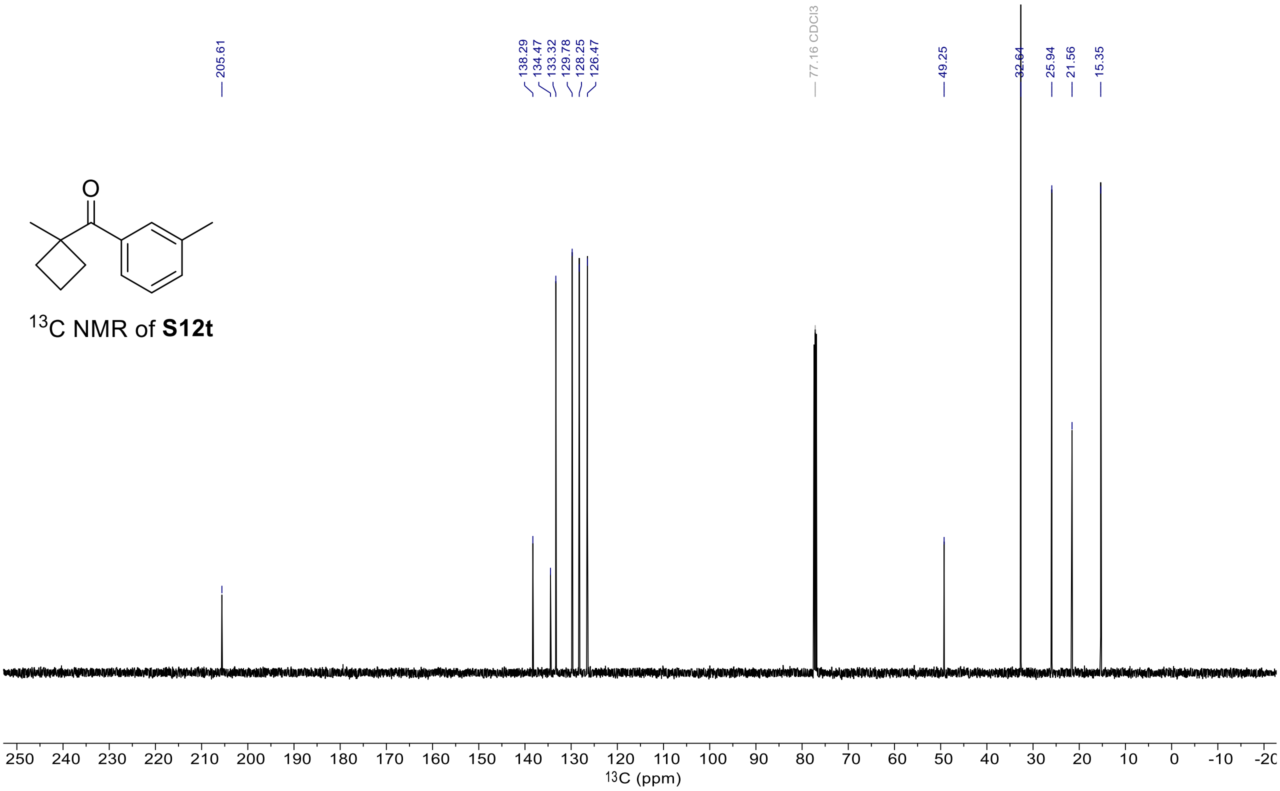

^{13}C NMR of **S12t**

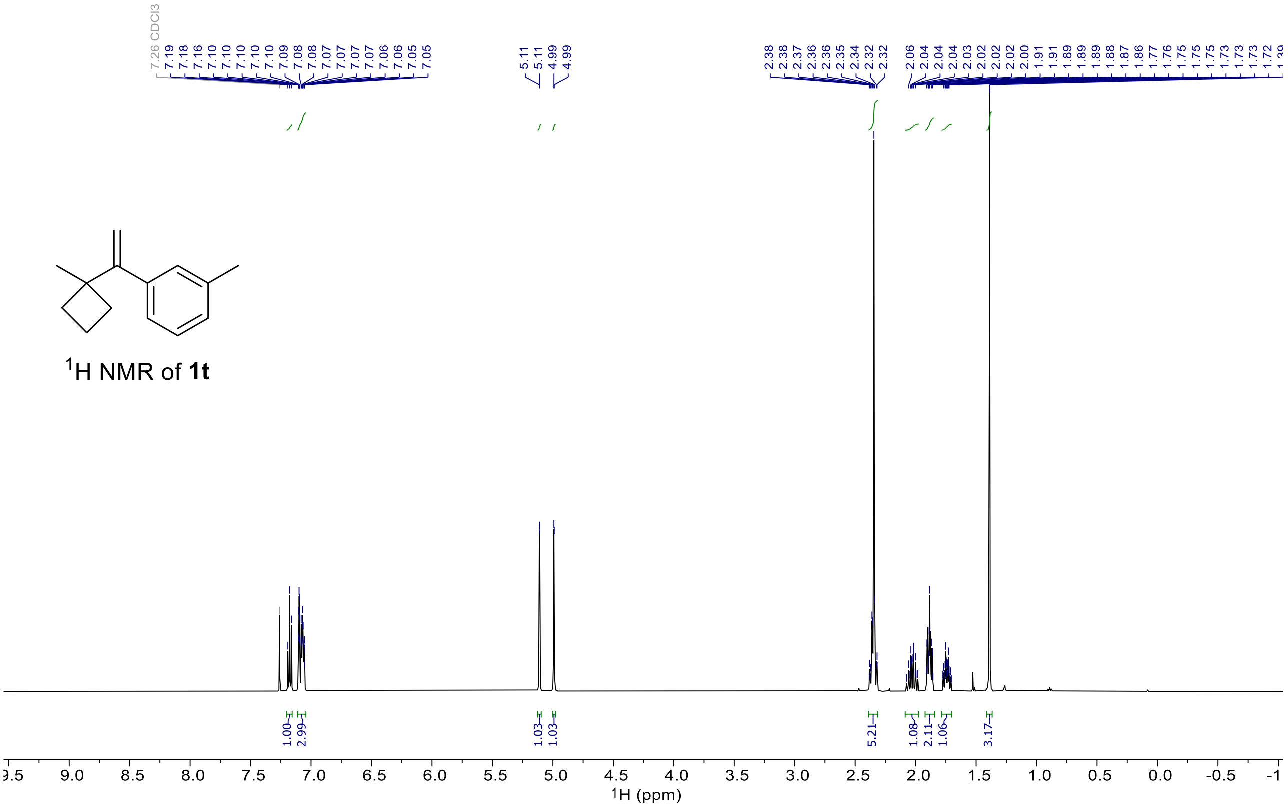
7.26 CDCl3
7.19 7.18 7.16 7.10 7.10 7.10 7.10 7.09 7.08 7.08 7.07 7.07 7.07 7.06 7.06 7.05 7.05
5.11 5.11 4.99 4.99
2.38 2.38 2.37 2.36 2.36 2.35 2.34 2.32 2.32 2.32
2.06 2.04 2.04 2.04 2.03 2.02 2.02 2.02 2.00 1.91 1.91 1.89 1.89 1.89 1.88 1.87 1.86 1.77 1.76 1.75 1.75 1.73 1.73 1.73 1.72
1.00 2.99 1.03 1.03 5.21 1.08 2.11 1.06 3.17
¹H NMR of **1t**
9.5 9.0 8.5 8.0 7.5 7.0 6.5 6.0 5.5 5.0 4.5 4.0 3.5 3.0 2.5 2.0 1.5 1.0 0.5 0.0 -0.5 -1
1H (ppm)

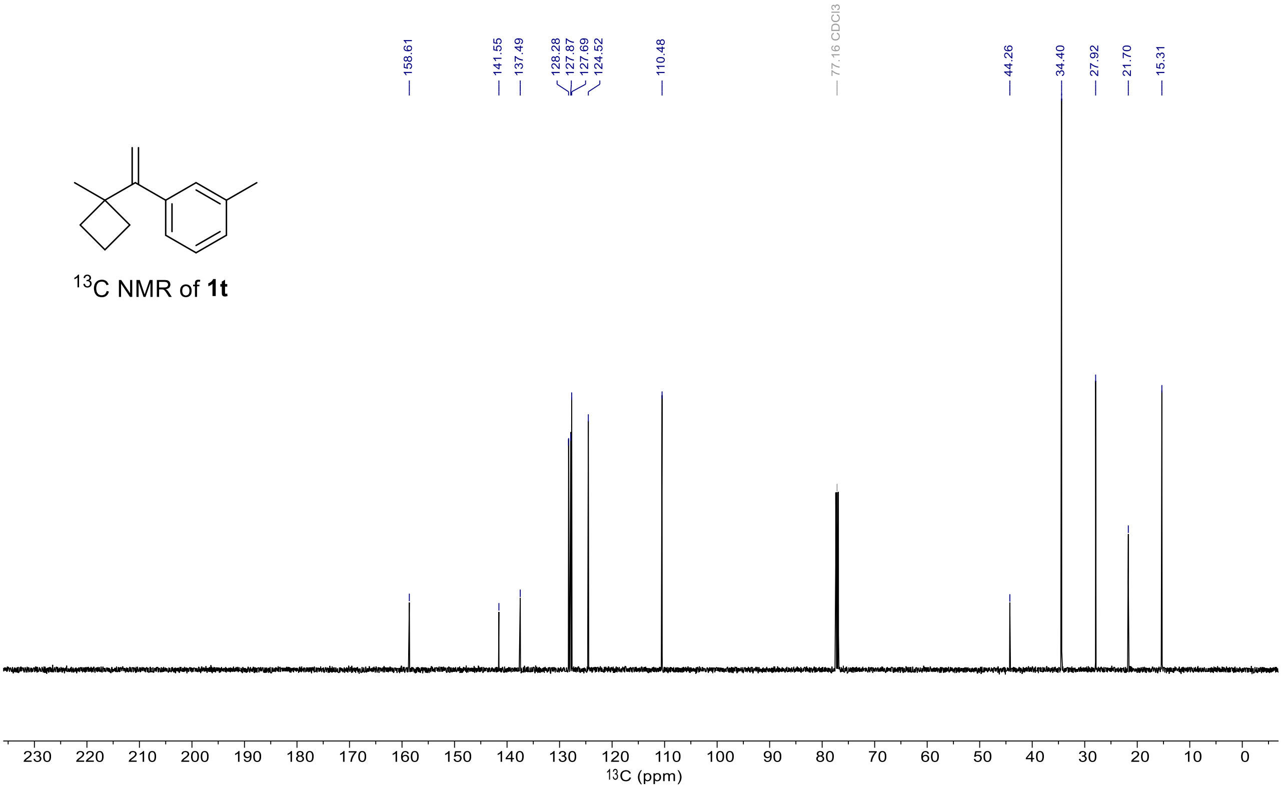
158.61
141.55
137.49
128.28 127.87 127.69 124.52
110.48
77.16 CDCl3
44.26
34.40
27.92
21.70
15.31
13C NMR of 1t
230 220 210 200 190 180 170 160 150 140 130 120 110 100 90 80 70 60 50 40 30 20 10 0
13C (ppm)

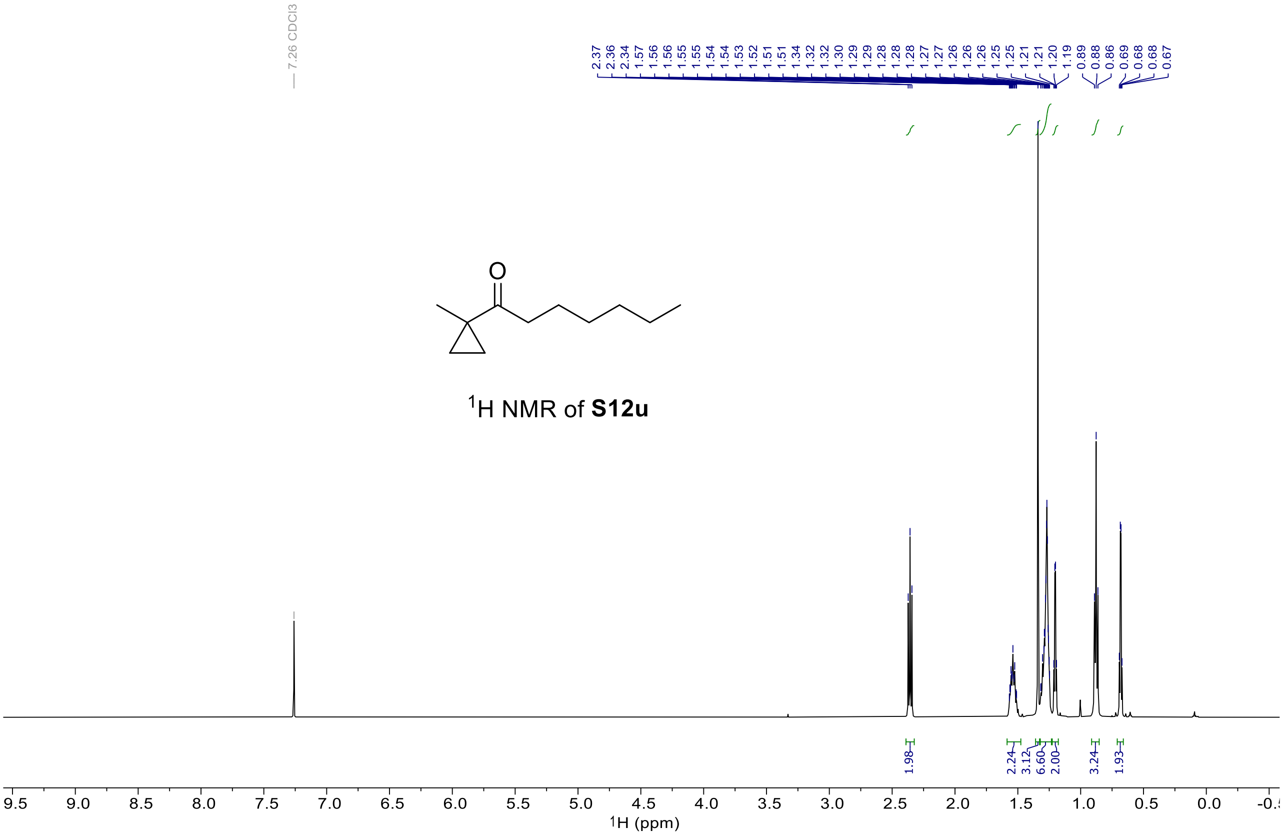

^{1}H NMR of **S12u**

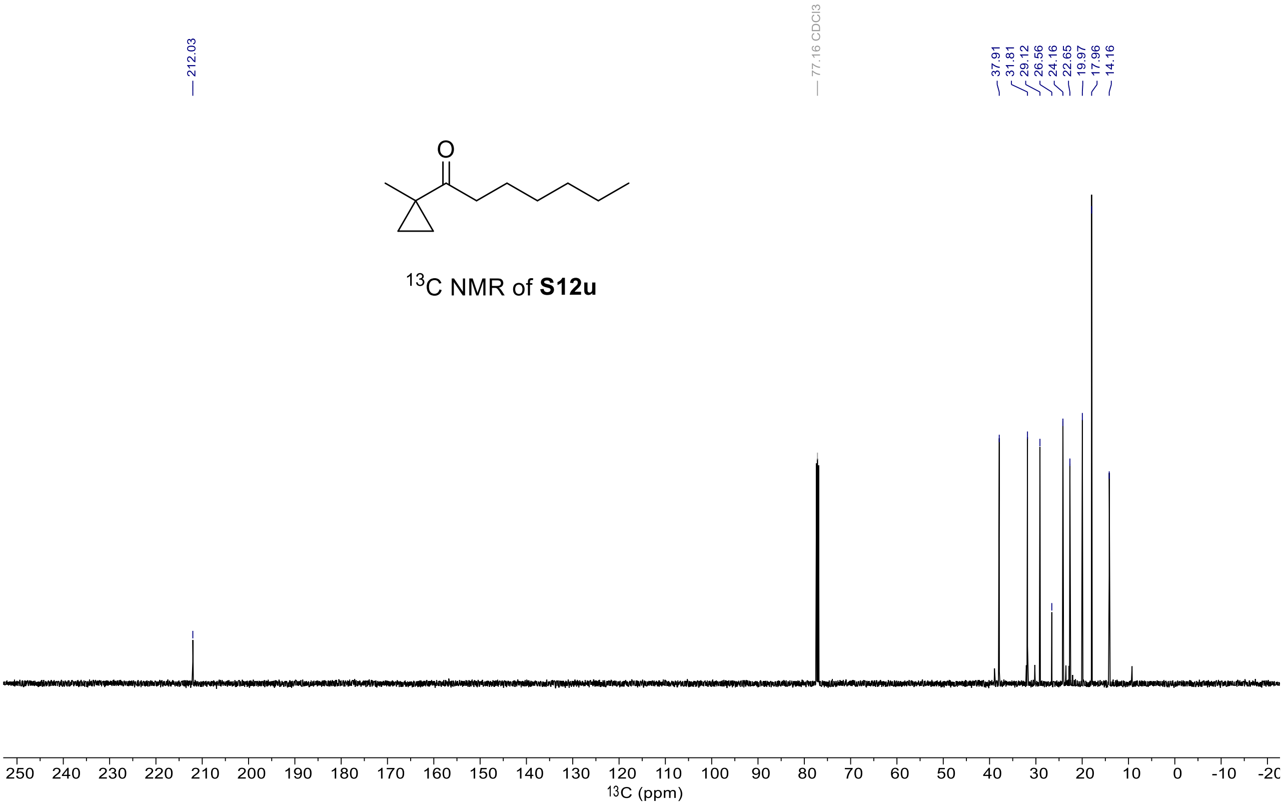

^{13}C NMR of **S12u**

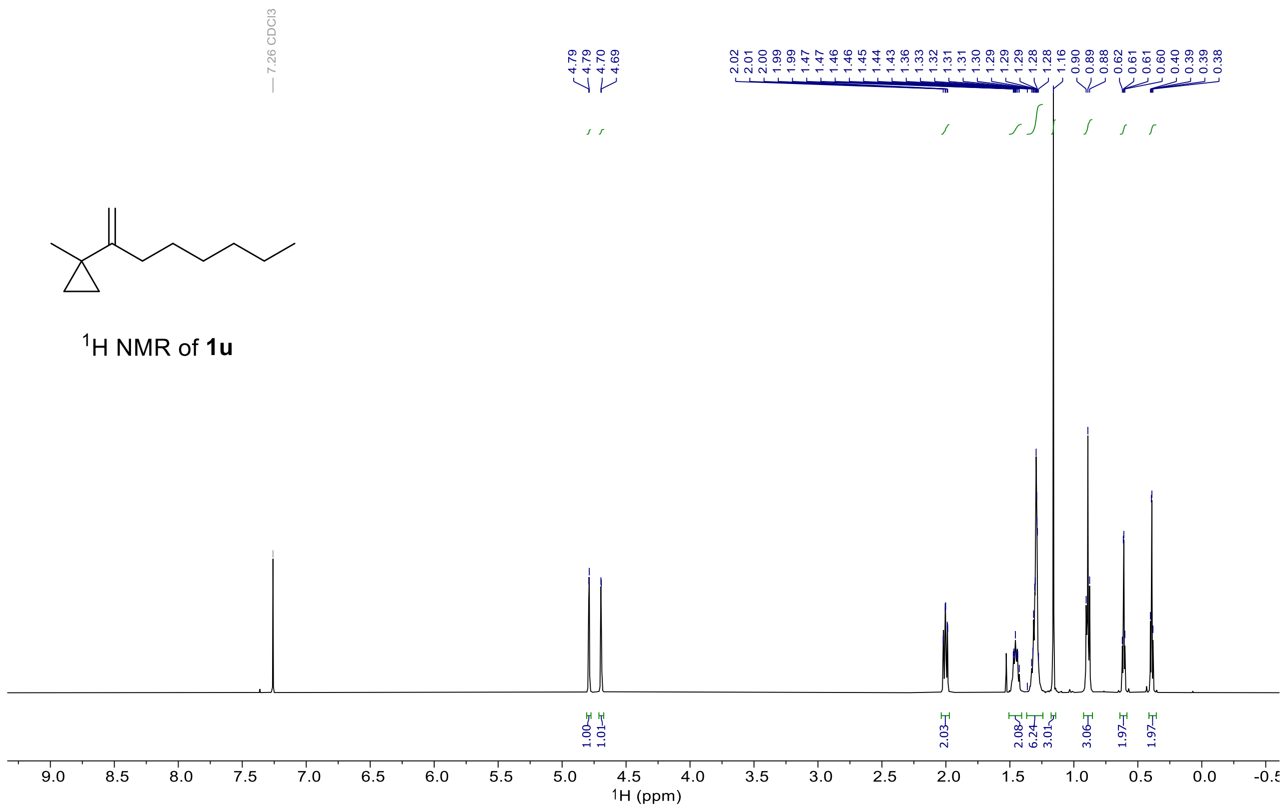
7.26 CDCl3
4.79
4.79
4.70
4.69
2.02
2.01
2.00
1.99
1.99
1.47
1.47
1.46
1.46
1.45
1.44
1.43
1.36
1.33
1.32
1.31
1.31
1.30
1.29
1.29
1.29
1.28
1.28
1.16
0.90
0.89
0.88
0.62
0.61
0.61
0.60
0.40
0.39
0.39
0.38
^{1}H NMR of **1u**
1.00
1.01
2.03
2.08
6.24
3.01
3.06
1.97
1.97
9.0 8.5 8.0 7.5 7.0 6.5 6.0 5.5 5.0 4.5 4.0 3.5 3.0 2.5 2.0 1.5 1.0 0.5 0.0 -0.5
^{1}H (ppm)

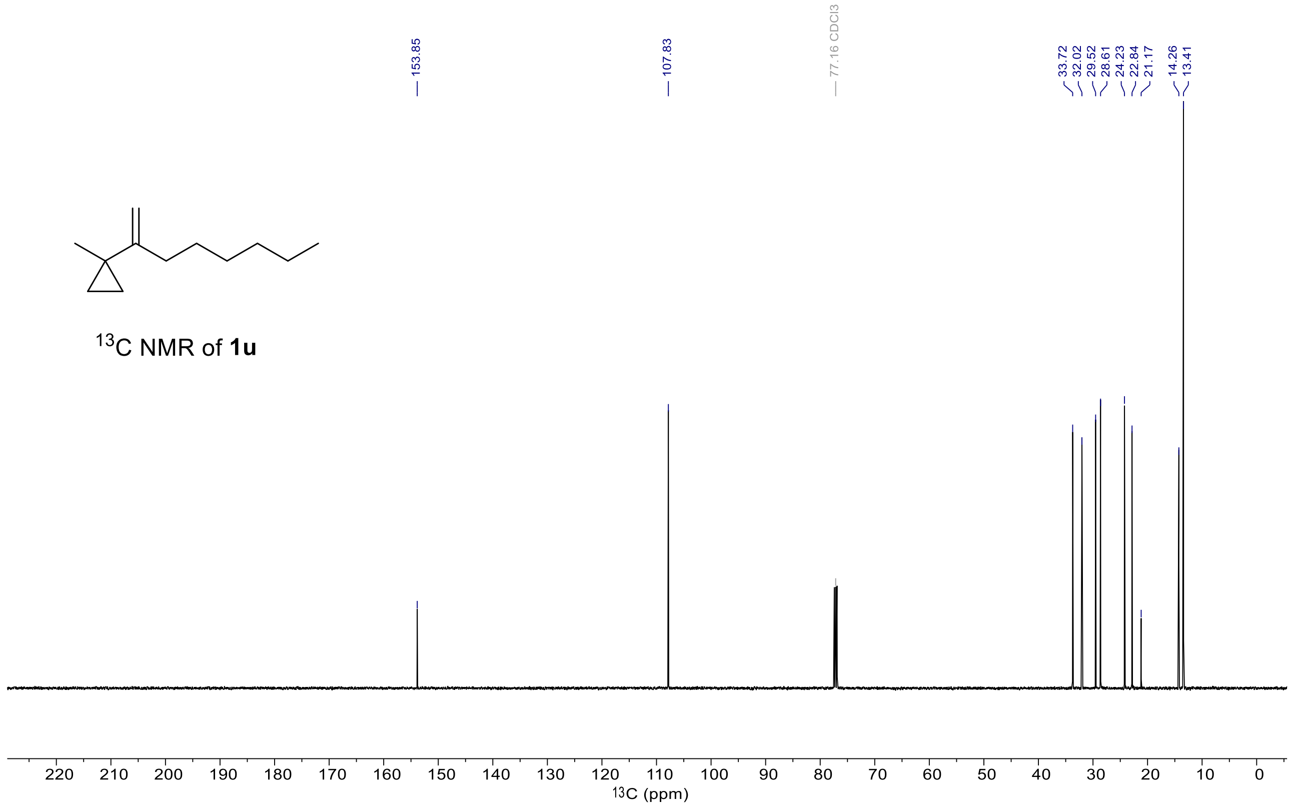
153.85
107.83
77.16 CDCl3
33.72
32.02
29.52
28.61
24.23
22.84
21.17
14.26
13.41
^{13}C NMR of **1u**
220 210 200 190 180 170 160 150 140 130 120 110 100 90 80 70 60 50 40 30 20 10 0
^{13}C (ppm)

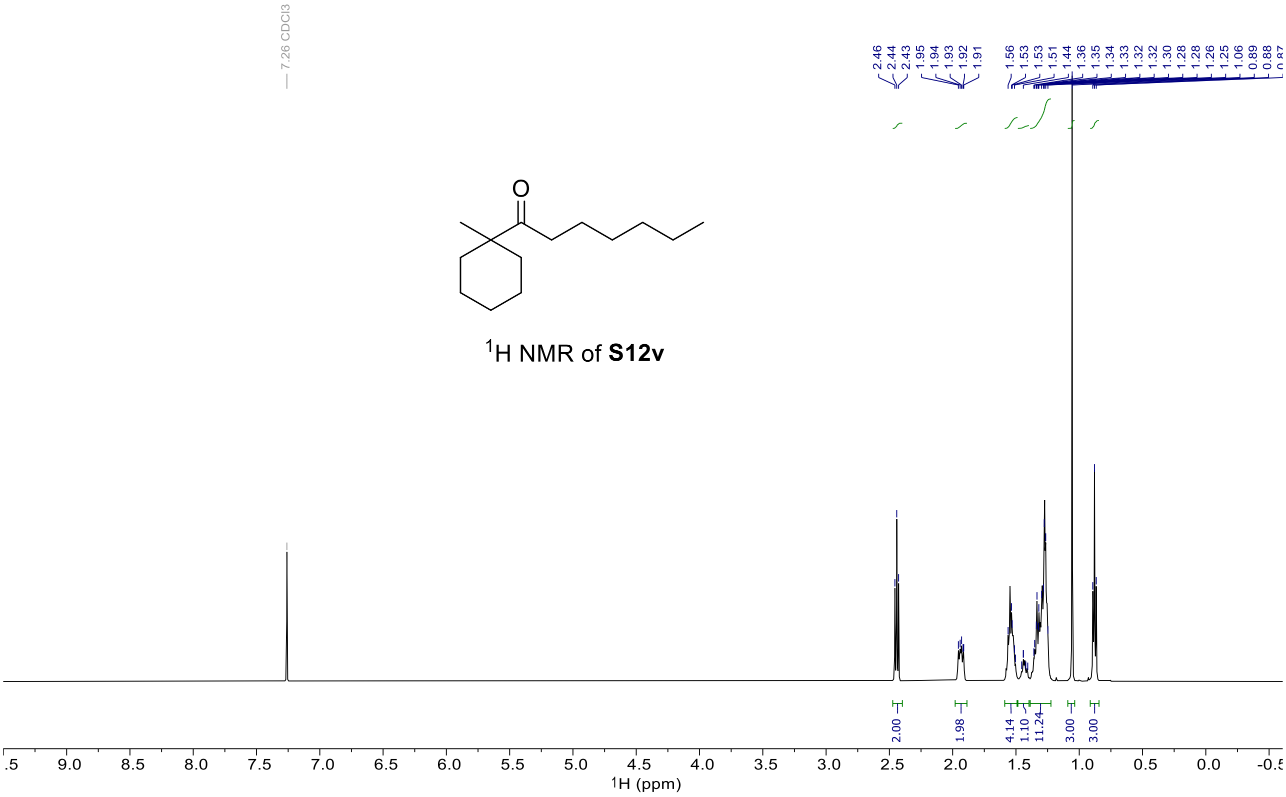
7.26 CDCl3
2.46
2.44
2.43
1.95
1.94
1.93
1.92
1.91
1.56
1.53
1.53
1.51
1.44
1.36
1.35
1.34
1.33
1.32
1.32
1.30
1.28
1.28
1.26
1.25
1.06
0.89
0.88
O
1H NMR of S12v
2.00
1.98
4.14
1.10
11.24
3.00
3.00
1H (ppm)

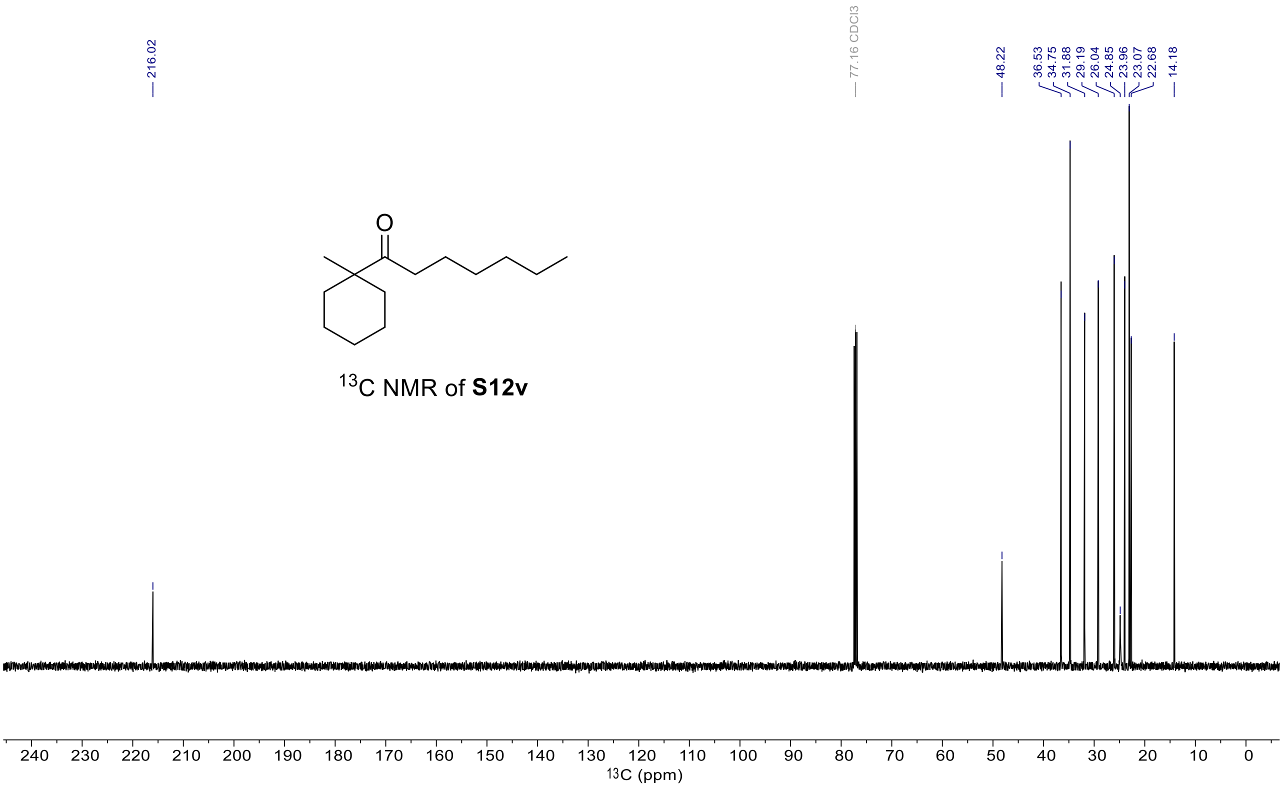
216.02
77.16 CDCl3
48.22
36.53
34.75
31.88
29.19
26.04
24.85
23.96
23.07
22.68
14.18
O
13C NMR of S12v
13C (ppm)

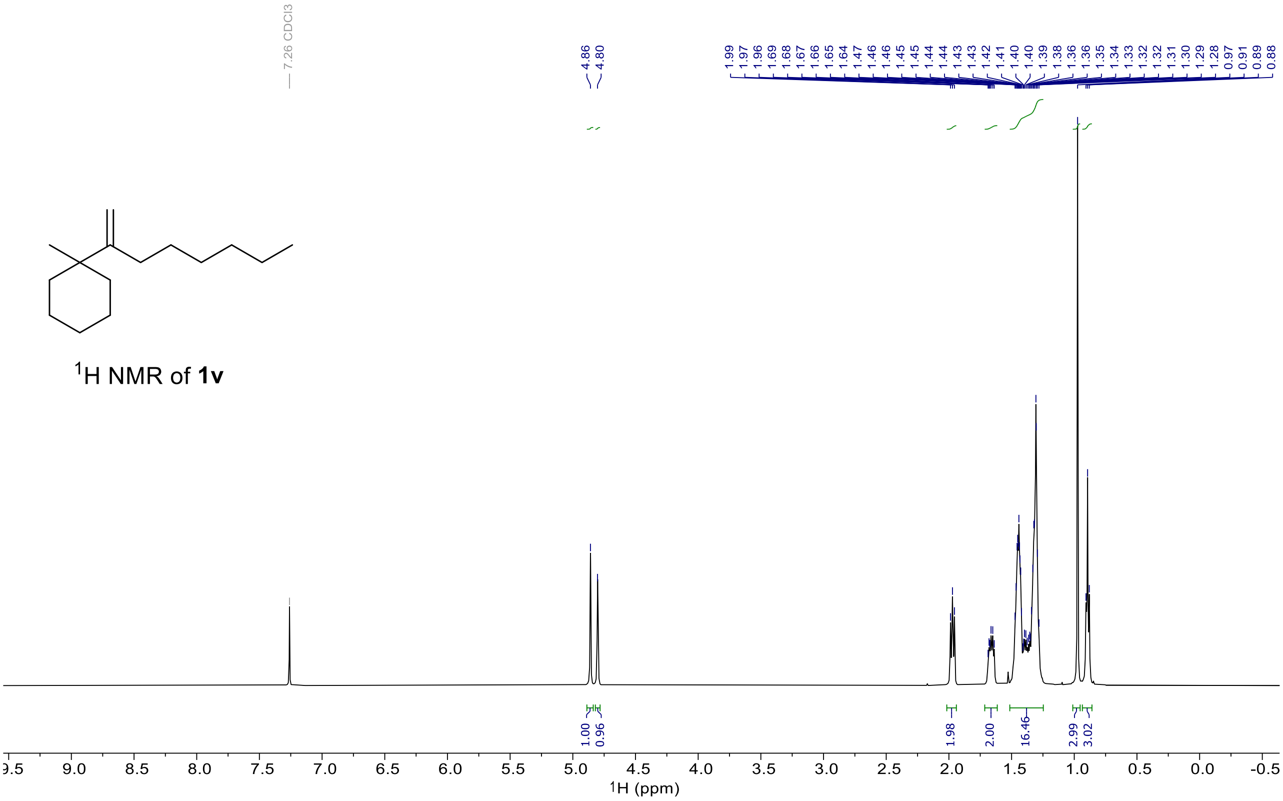

7.26 CDCl3
4.86
4.80
1.99
1.97
1.96
1.69
1.68
1.67
1.66
1.65
1.64
1.47
1.46
1.46
1.45
1.45
1.44
1.44
1.43
1.43
1.42
1.41
1.40
1.40
1.40
1.39
1.38
1.36
1.36
1.36
1.35
1.34
1.33
1.32
1.32
1.31
1.30
1.29
1.28
0.97
0.91
0.89
0.88
^{1}H NMR of **1v**
1.00
0.96
1.98
2.00
16.46
2.99
3.02
9.5 9.0 8.5 8.0 7.5 7.0 6.5 6.0 5.5 5.0 4.5 4.0 3.5 3.0 2.5 2.0 1.5 1.0 0.5 0.0 -0.5
^{1}H (ppm)

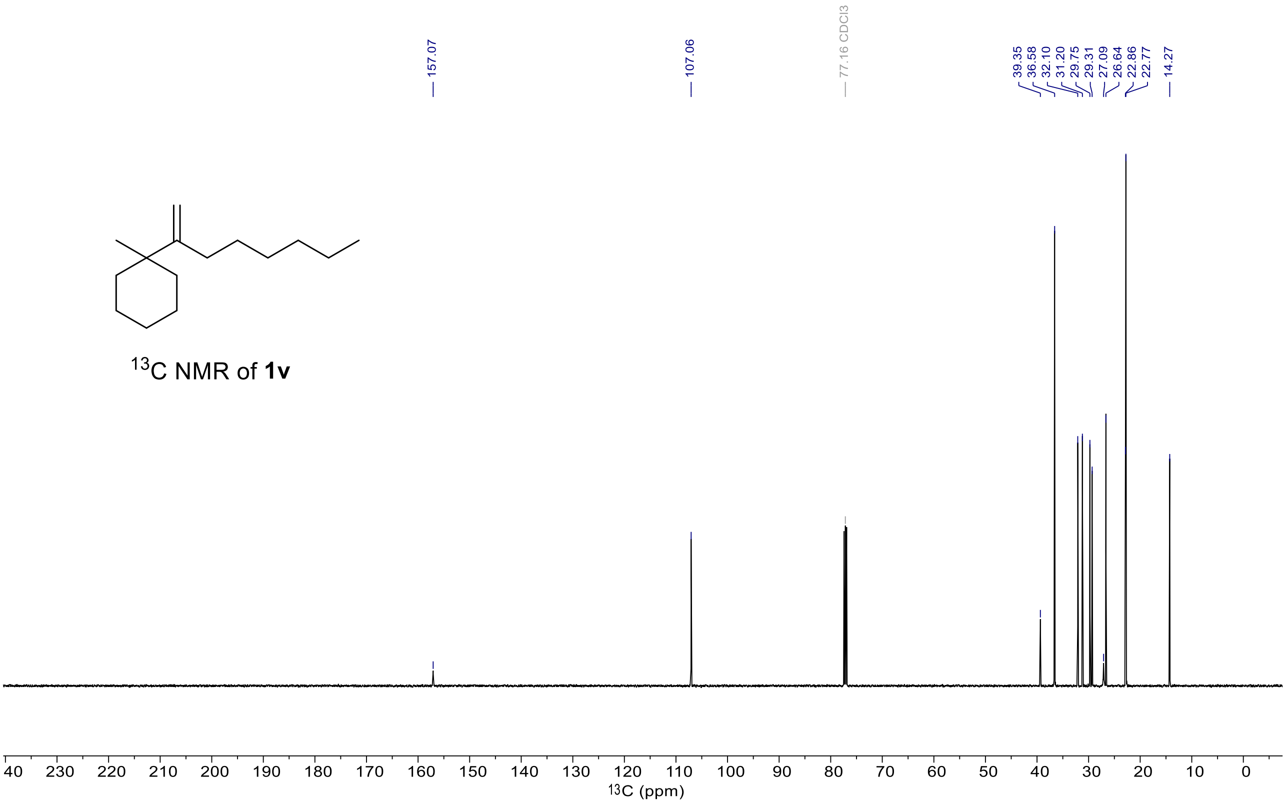

157.07
107.06
77.16 CDCl3
39.35
36.58
32.10
31.20
29.75
29.31
27.09
26.64
22.86
22.77
14.27
^{13}C NMR of **1v**
240 230 220 210 200 190 180 170 160 150 140 130 120 110 100 90 80 70 60 50 40 30 20 10 0
^{13}C (ppm)

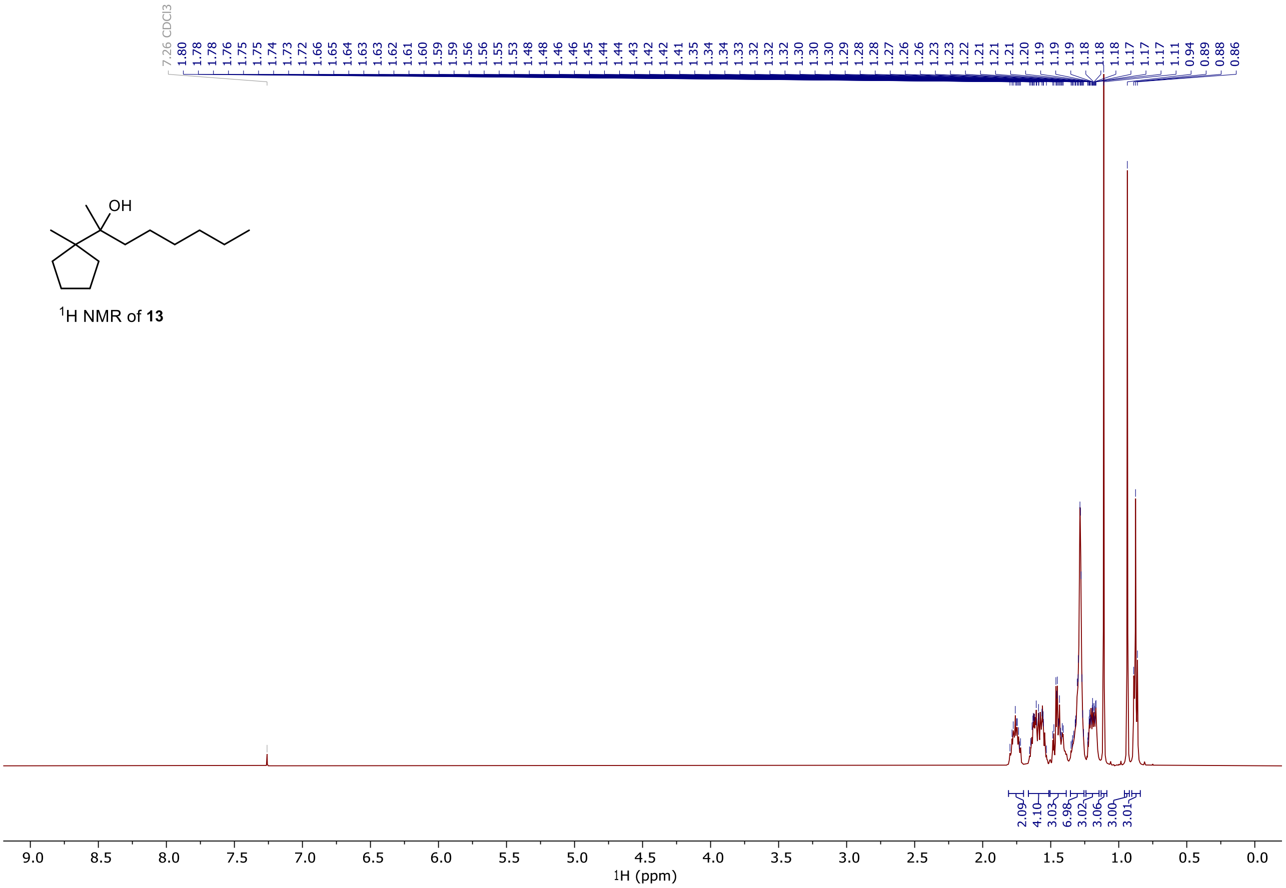

^{1}H NMR of **13**

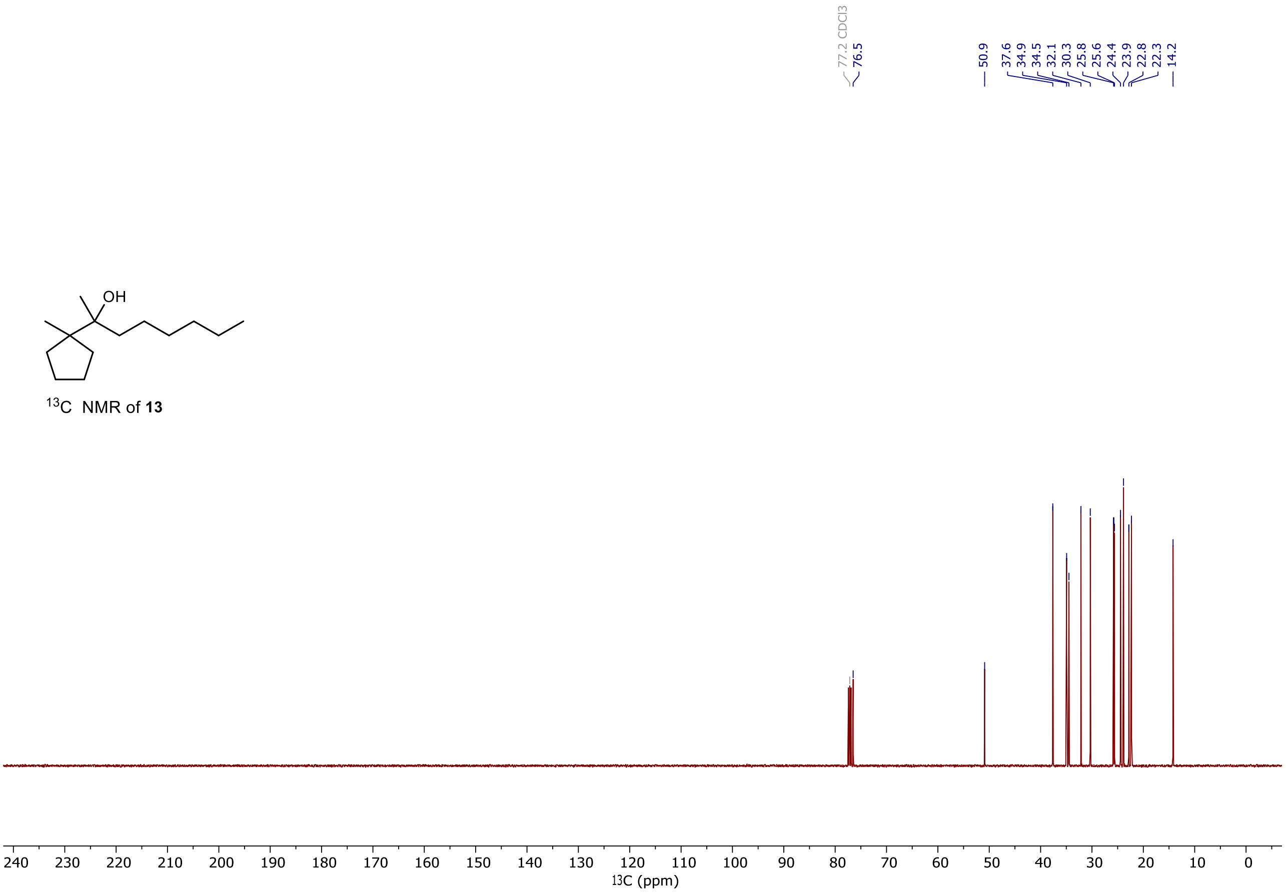

^{13}C NMR of **13**

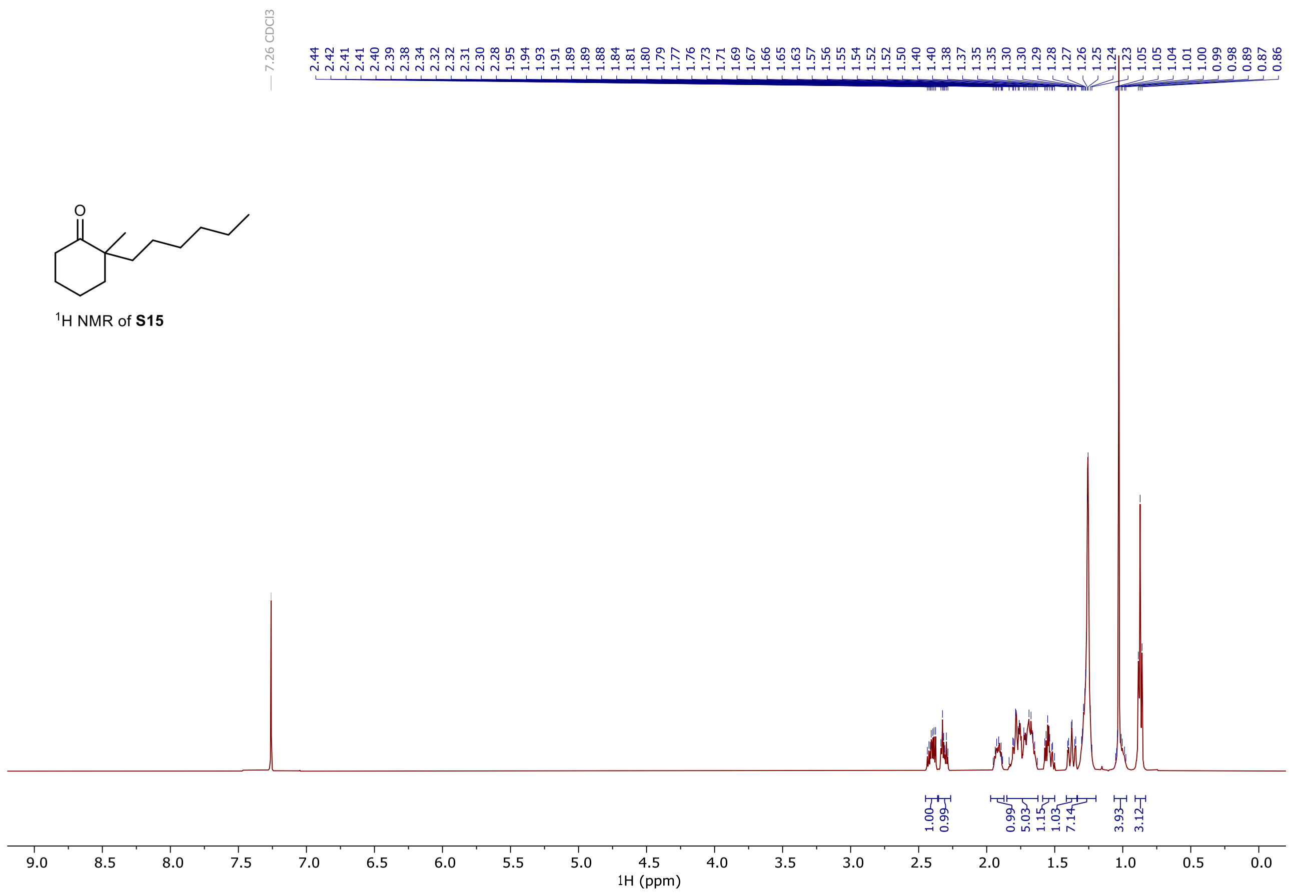
^{1}H NMR of **S15**
1H (ppm)

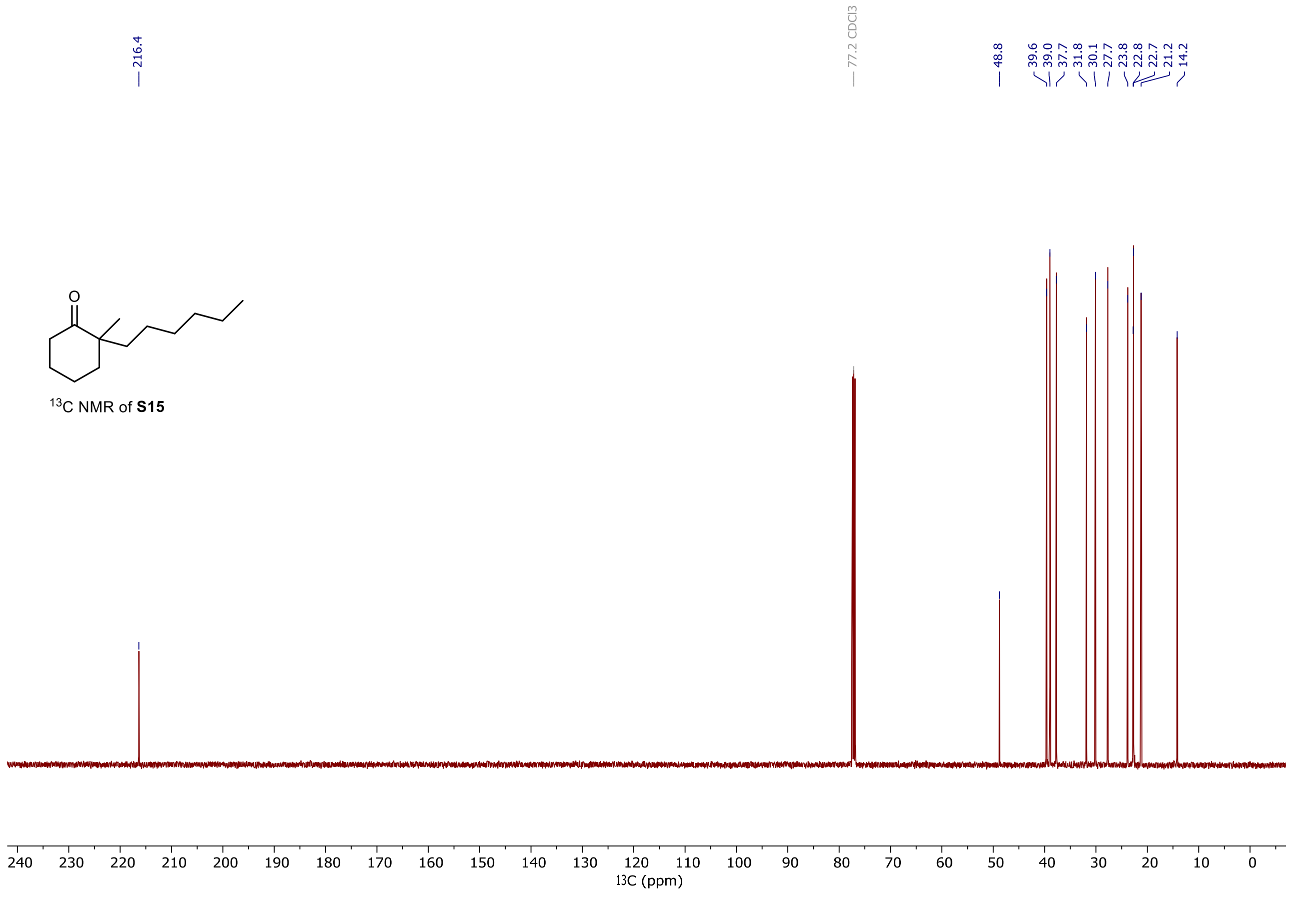
^{13}C NMR of **S15**
13C (ppm)

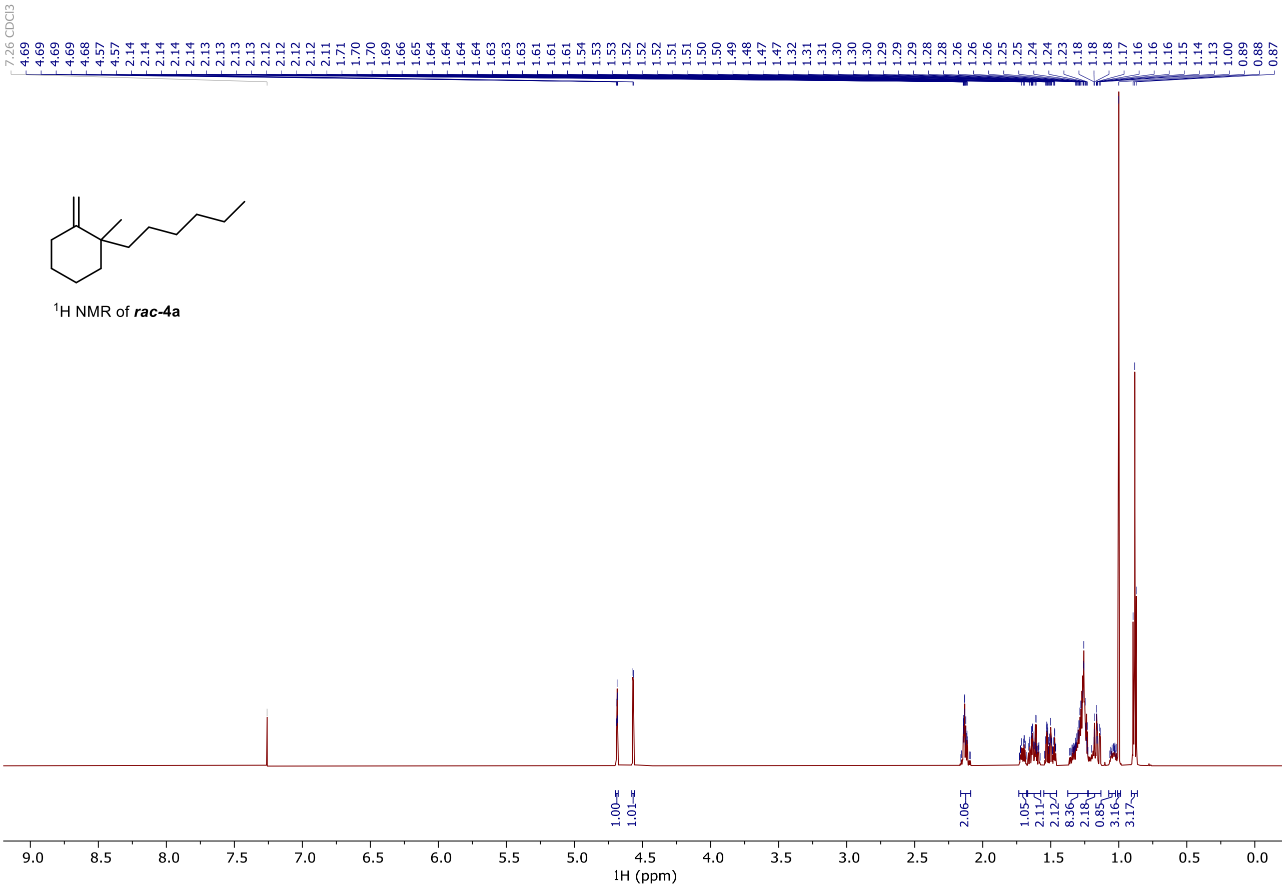

7.26 CDCl3
4.69 4.69 4.69 4.69 4.68 4.57 4.57 2.14 2.14 2.14 2.14 2.14 2.13 2.13 2.13 2.13 2.12 2.12 2.12 2.11 1.71 1.70 1.70 1.69 1.66 1.65 1.64 1.64 1.64 1.63 1.63 1.63 1.61 1.61 1.61 1.54 1.53 1.53 1.52 1.52 1.51 1.51 1.50 1.50 1.49 1.48 1.47 1.32 1.31 1.31 1.30 1.30 1.30 1.29 1.29 1.28 1.28 1.26 1.26 1.25 1.25 1.24 1.24 1.23 1.18 1.18 1.17 1.16 1.16 1.15 1.14 1.13 1.00 0.89 0.88 0.87
1H NMR of rac-4a
1.00 1.01 2.06 1.05 2.11 2.12 8.36 2.18 0.85 3.16 3.17
9.0 8.5 8.0 7.5 7.0 6.5 6.0 5.5 5.0 4.5 4.0 3.5 3.0 2.5 2.0 1.5 1.0 0.5 0.0
1H (ppm)

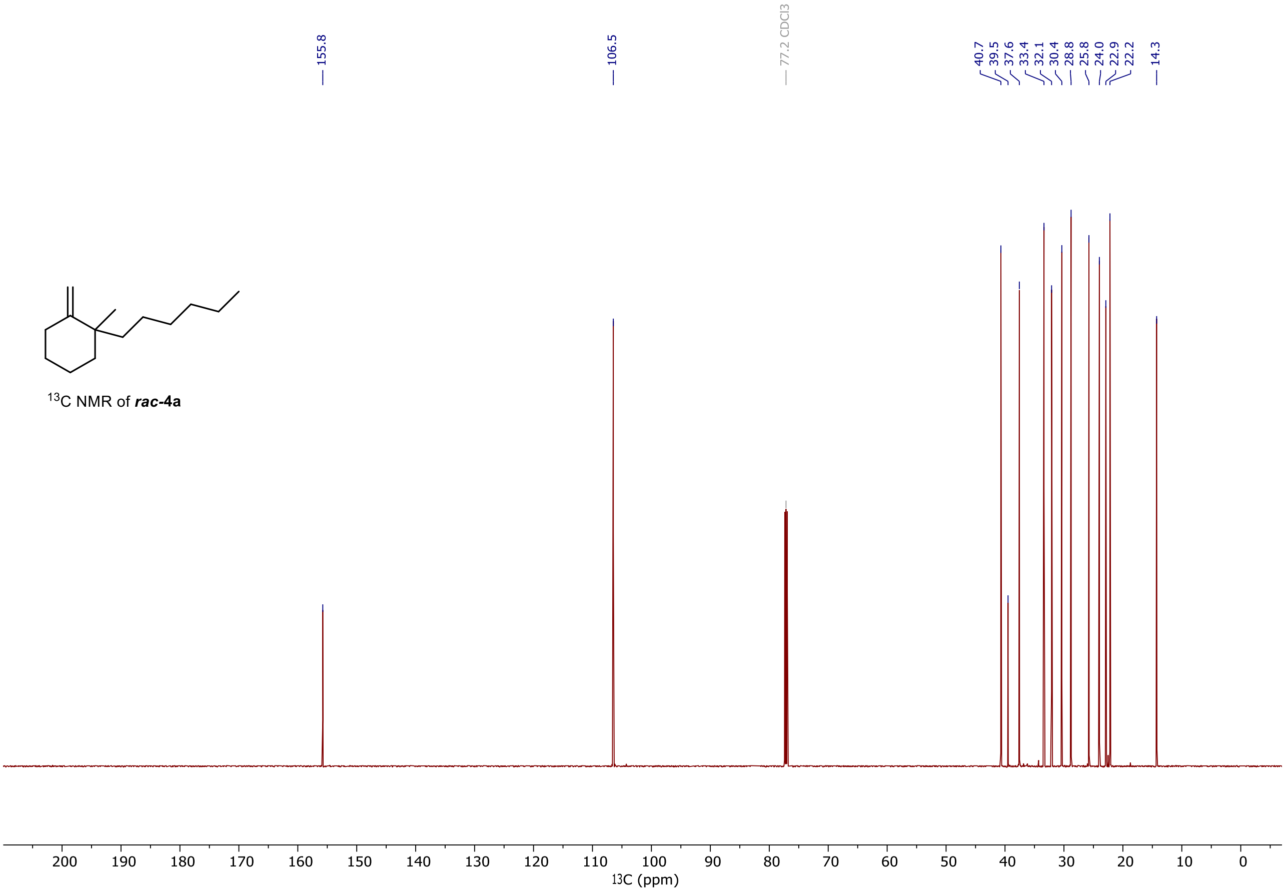

155.8
106.5
77.2 CDCl3
40.7 39.5 37.6 33.4 32.1 30.4 28.8 25.8 24.0 22.9 22.2
14.3
13C NMR of rac-4a
200 190 180 170 160 150 140 130 120 110 100 90 80 70 60 50 40 30 20 10 0
13C (ppm)

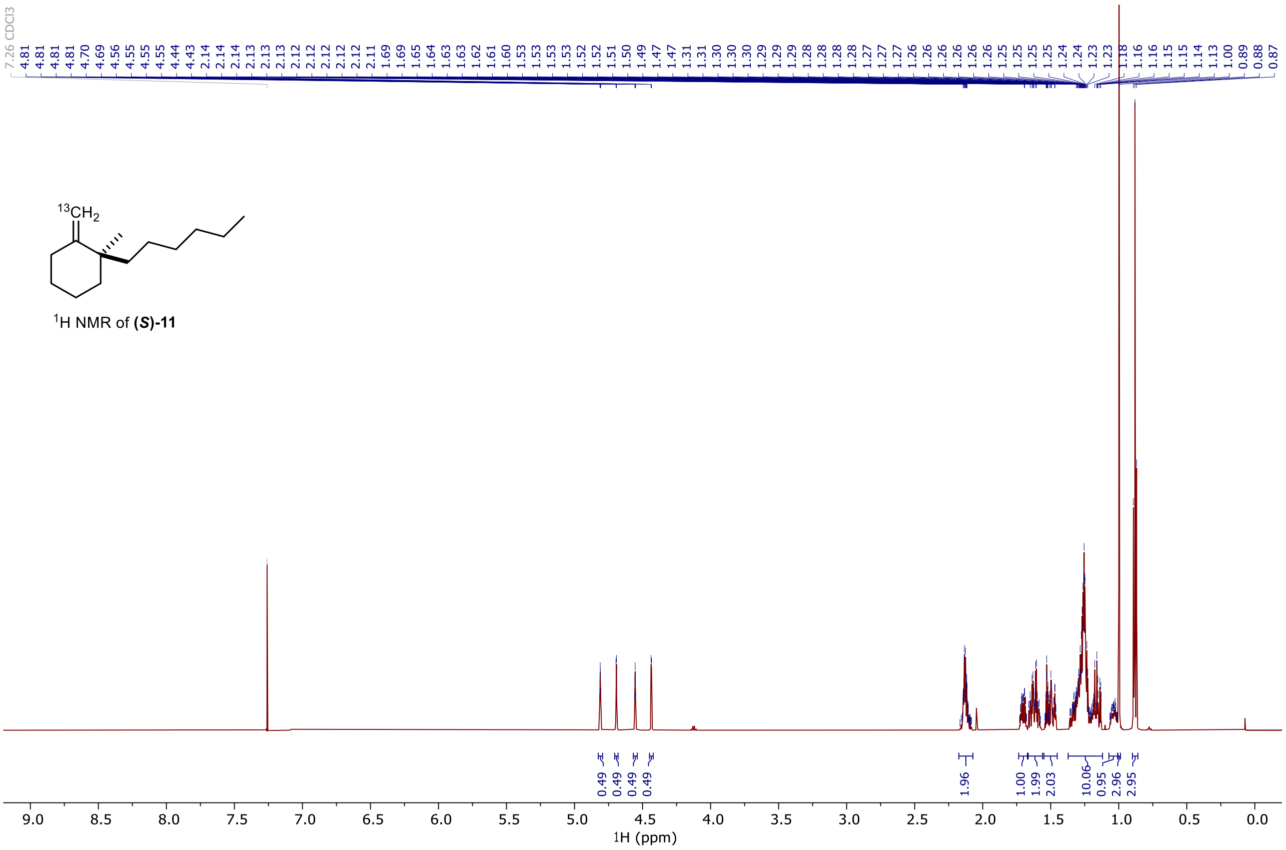
^{13}CH$_2$
^{1}H NMR of (S)-11
7.26 CDCl3
0.49 0.49 0.49 0.49 1.96 1.00 1.99 2.03 10.06 0.95 2.96 2.95
1H (ppm)

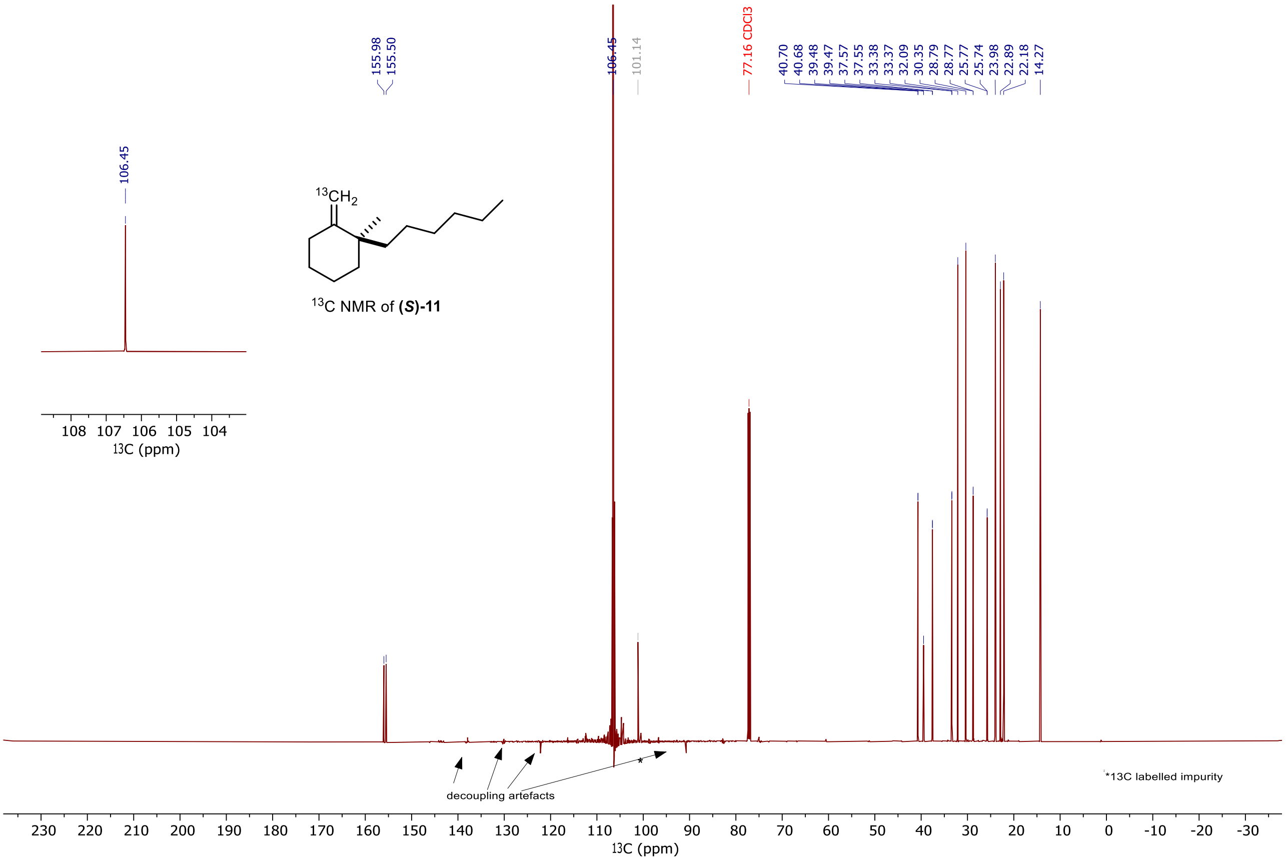
155.98
155.50
106.45
101.14
77.16 CDCl3
40.70 40.68 39.48 39.47 37.57 37.55 33.38 33.37 32.09 30.35 28.79 28.77 25.77 25.74 23.98 22.89 22.18 14.27
^{13}CH$_2$
^{13}C NMR of (S)-11
13C (ppm)
decoupling artefacts
*13C labelled impurity
13C (ppm)

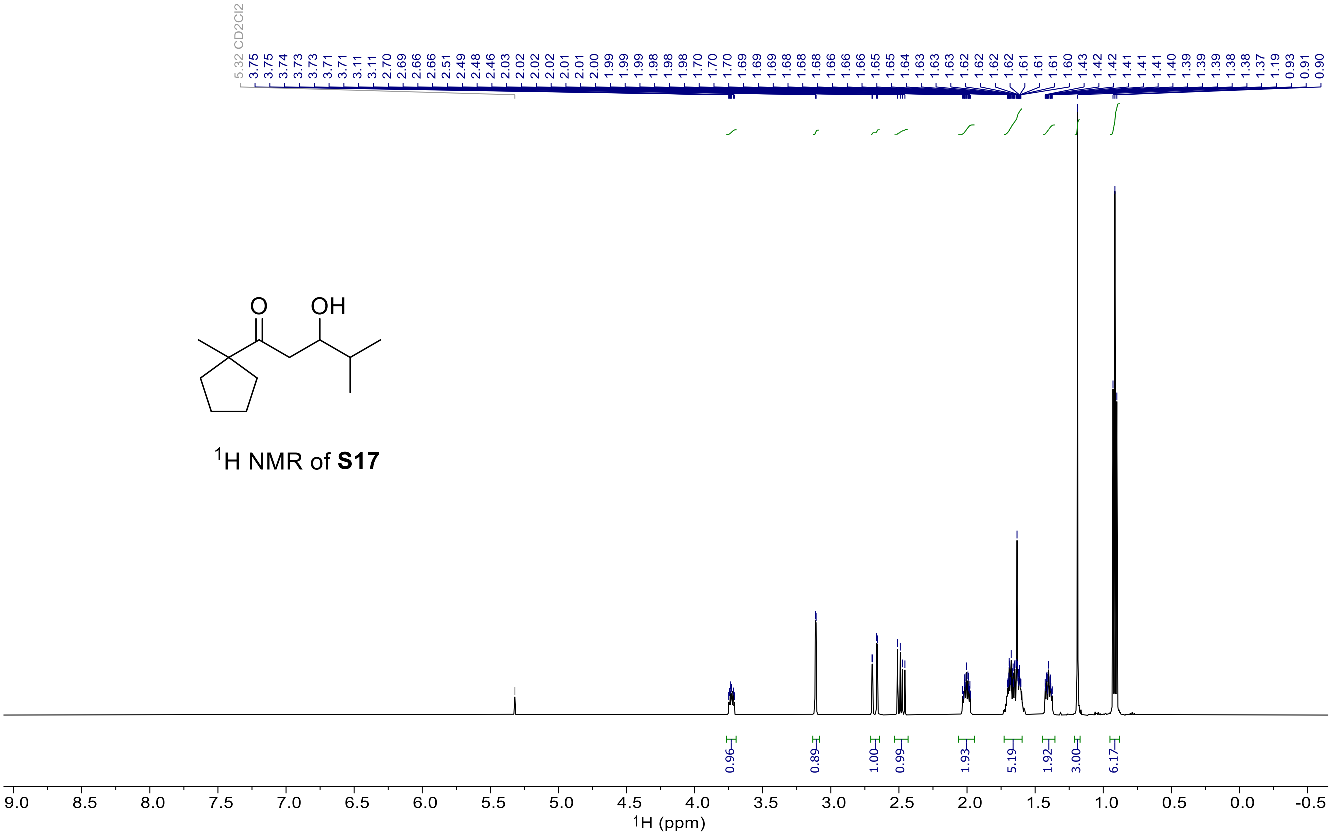
5.32 CD2Cl2
3.75 3.75 3.74 3.73 3.73 3.71 3.71 3.11 3.11 2.70 2.69 2.66 2.66 2.51 2.49 2.48 2.46 2.03 2.02 2.02 2.02 2.01 2.01 2.00 1.99 1.99 1.99 1.98 1.98 1.98 1.70 1.70 1.70 1.69 1.69 1.69 1.68 1.68 1.68 1.66 1.66 1.65 1.65 1.64 1.63 1.63 1.63 1.62 1.62 1.62 1.61 1.61 1.61 1.60 1.43 1.42 1.42 1.41 1.41 1.40 1.39 1.39 1.38 1.38 1.37 1.19 0.93 0.91 0.90
O
OH
1H NMR of S17
0.96 0.89 1.00 0.99 1.93 5.19 1.92 3.00 6.17
9.0 8.5 8.0 7.5 7.0 6.5 6.0 5.5 5.0 4.5 4.0 3.5 3.0 2.5 2.0 1.5 1.0 0.5 0.0 -0.5
1H (ppm)

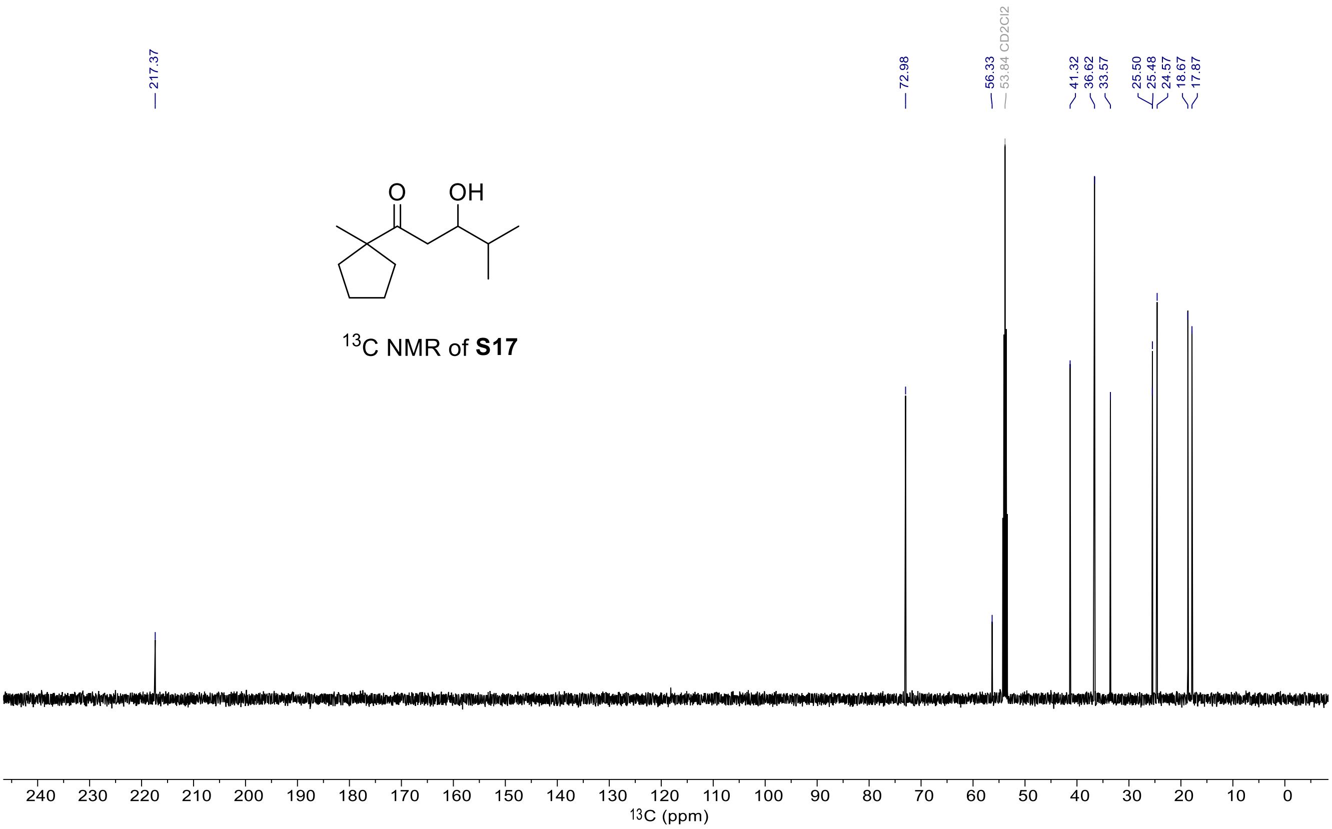
217.37
72.98
56.33
53.84 CD2Cl2
41.32 36.62 33.57
25.50 25.48 24.57 18.67 17.87
O
OH
13C NMR of S17
240 230 220 210 200 190 180 170 160 150 140 130 120 110 100 90 80 70 60 50 40 30 20 10 0
13C (ppm)

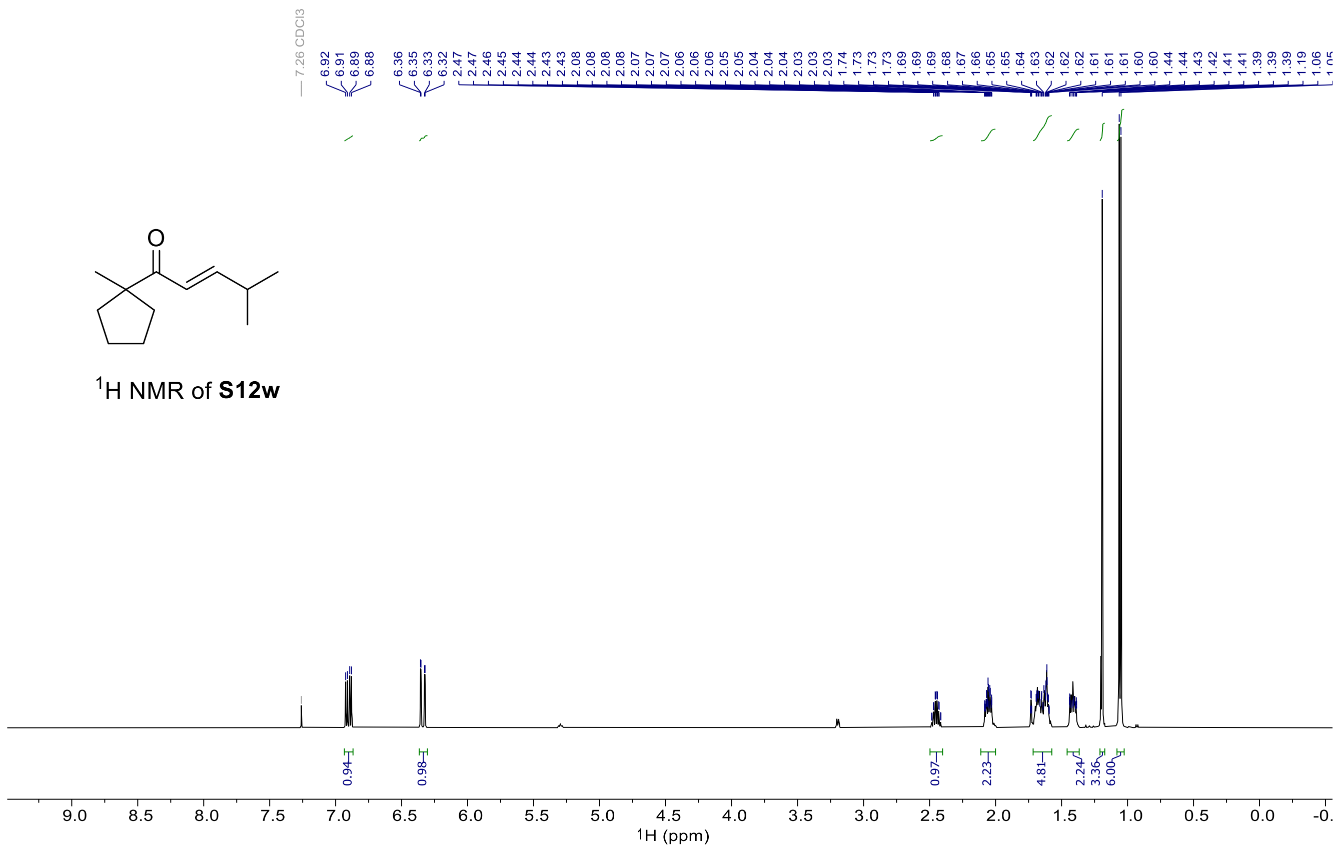

^{1}H NMR of **S12w**

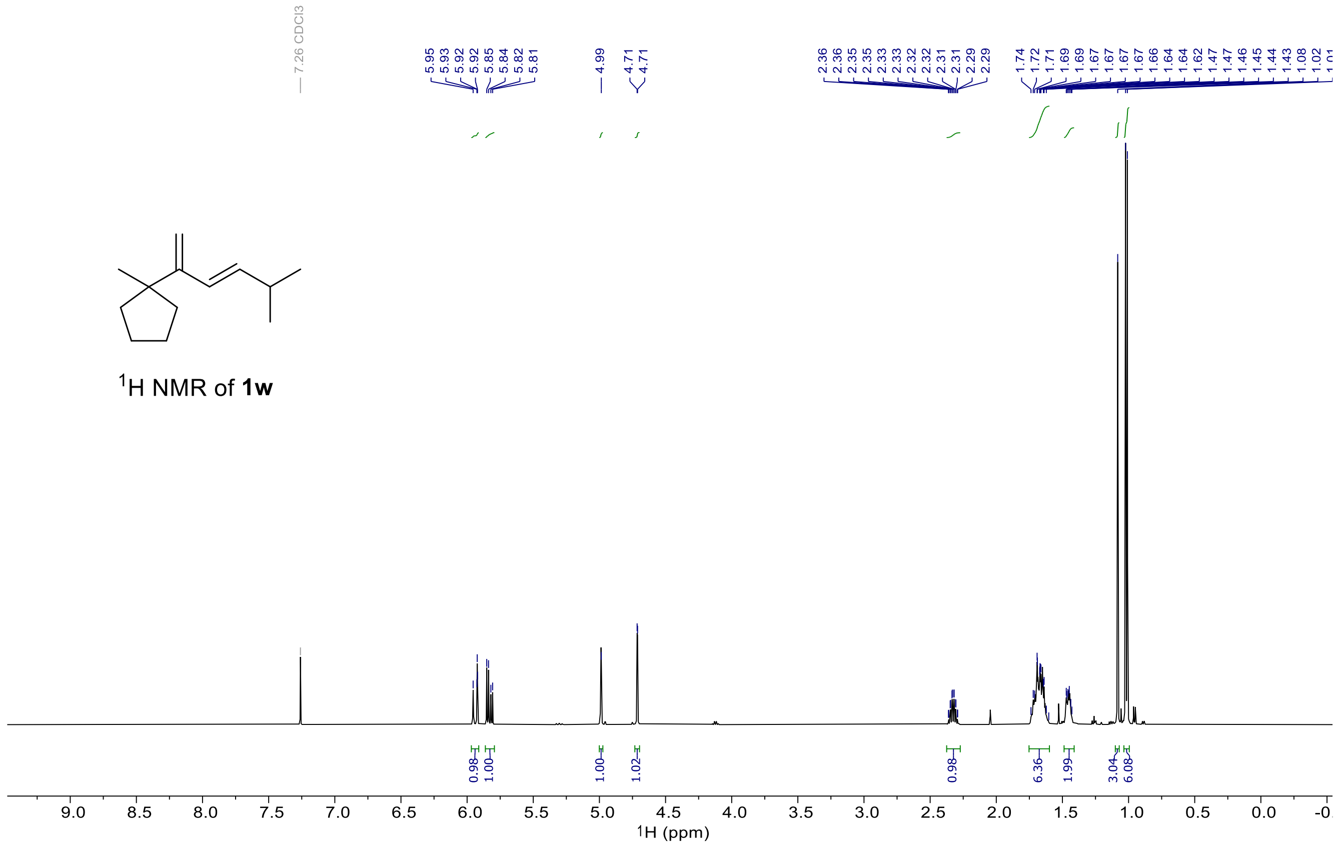

^{1}H NMR of **1w**

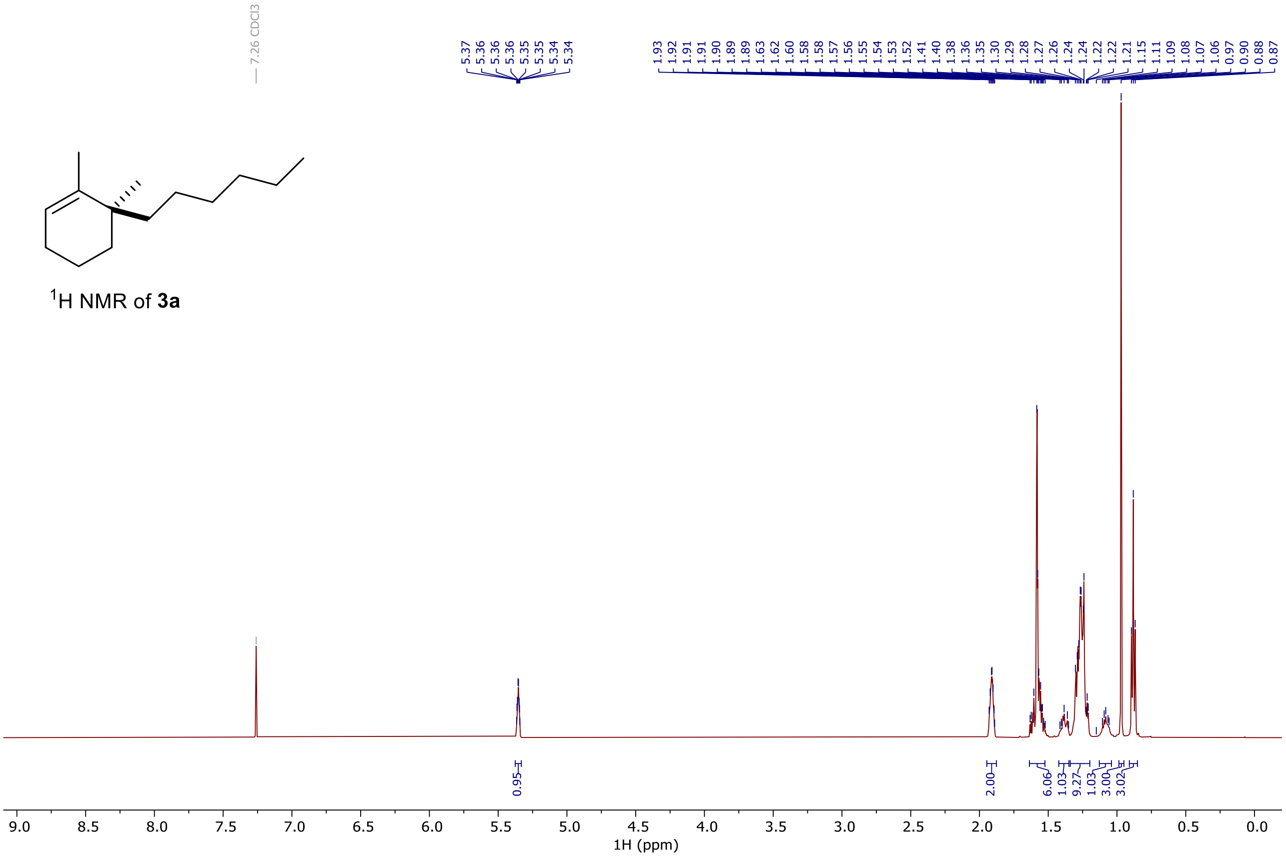

^{1}H NMR of **3a**

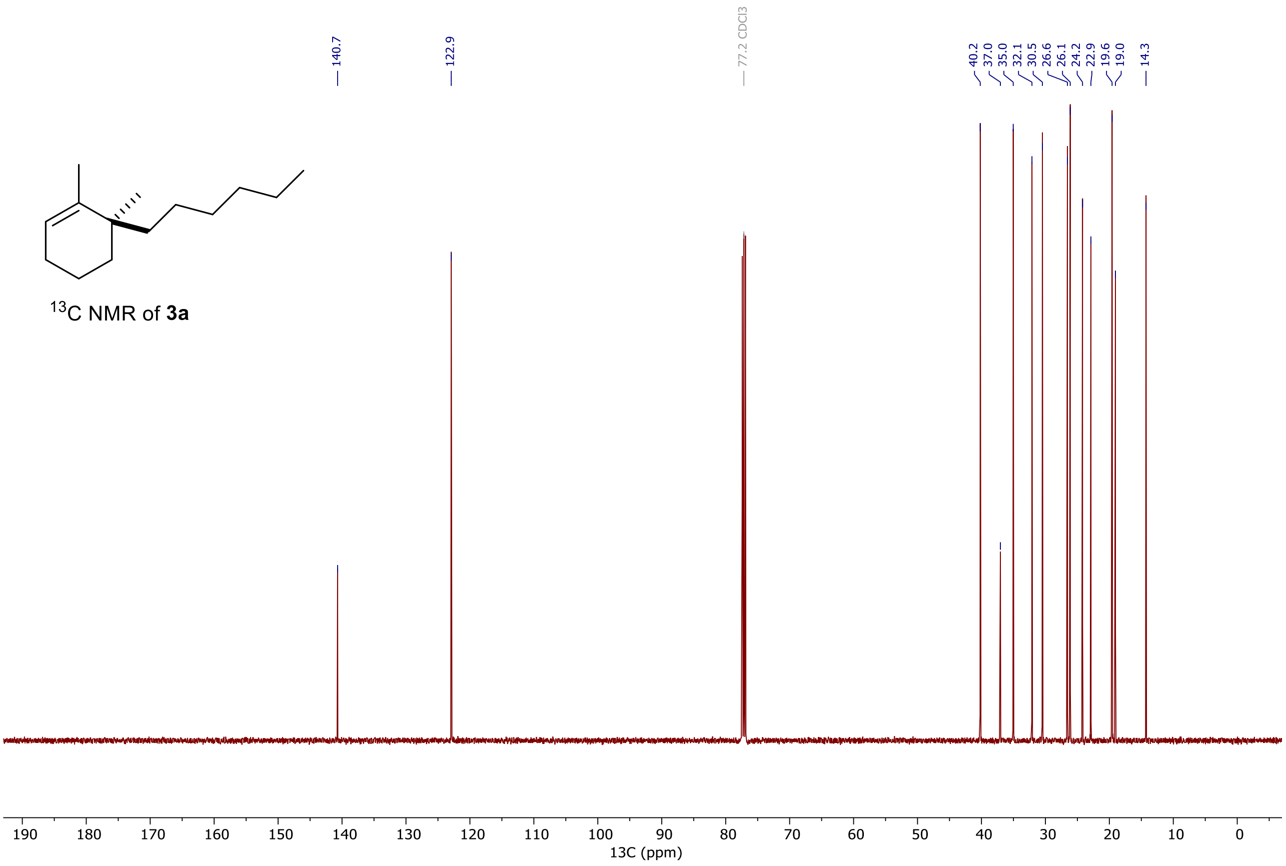

^{13}C NMR of **3a**

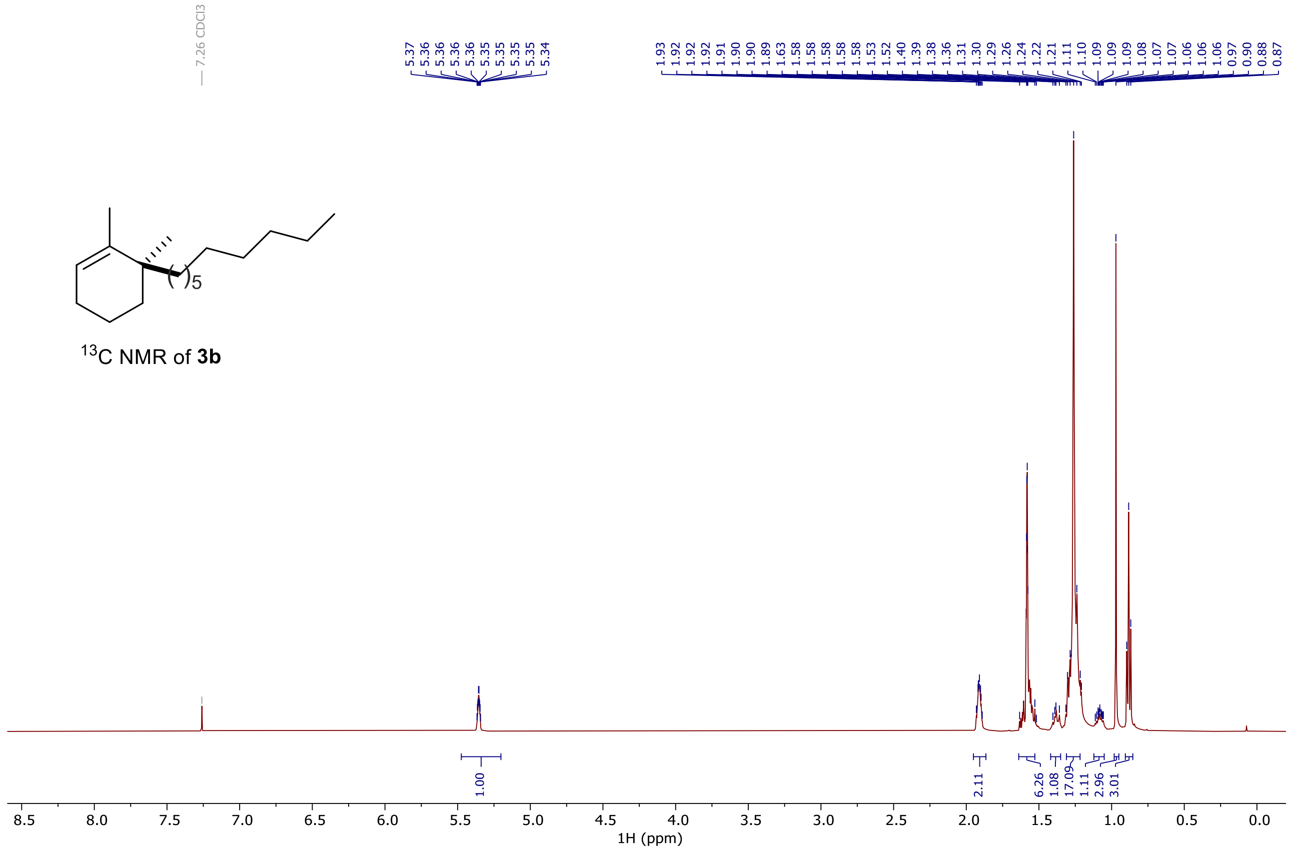
7.26 CDCl3
5.37 5.36 5.36 5.36 5.35 5.35 5.35 5.35 5.34
1.93 1.92 1.92 1.92 1.91 1.90 1.90 1.89 1.63 1.58 1.58 1.58 1.58 1.58 1.53 1.52 1.52 1.40 1.39 1.38 1.36 1.31 1.30 1.29 1.26 1.24 1.22 1.21 1.11 1.10 1.09 1.09 1.09 1.08 1.07 1.07 1.06 1.06 0.97 0.90 0.88 0.87
5
^{13}C NMR of **3b**
1.00
2.11
6.26
1.08
17.09
1.11
2.96
3.01
1H (ppm)

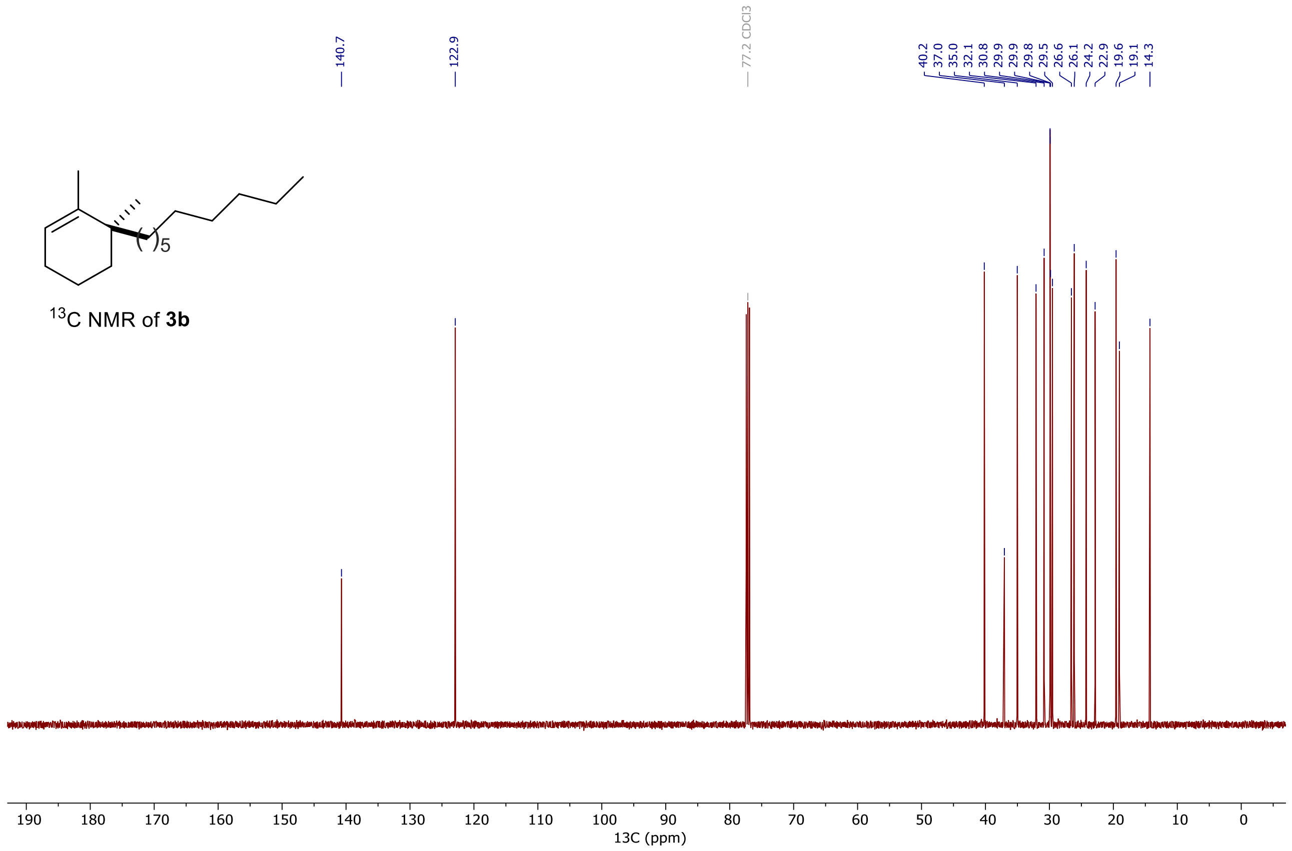
140.7
122.9
77.2 CDCl3
40.2 37.0 35.0 32.1 30.8 29.9 29.9 29.8 29.5 26.6 26.1 24.2 22.9 19.6 19.1 14.3
5
^{13}C NMR of **3b**
13C (ppm)

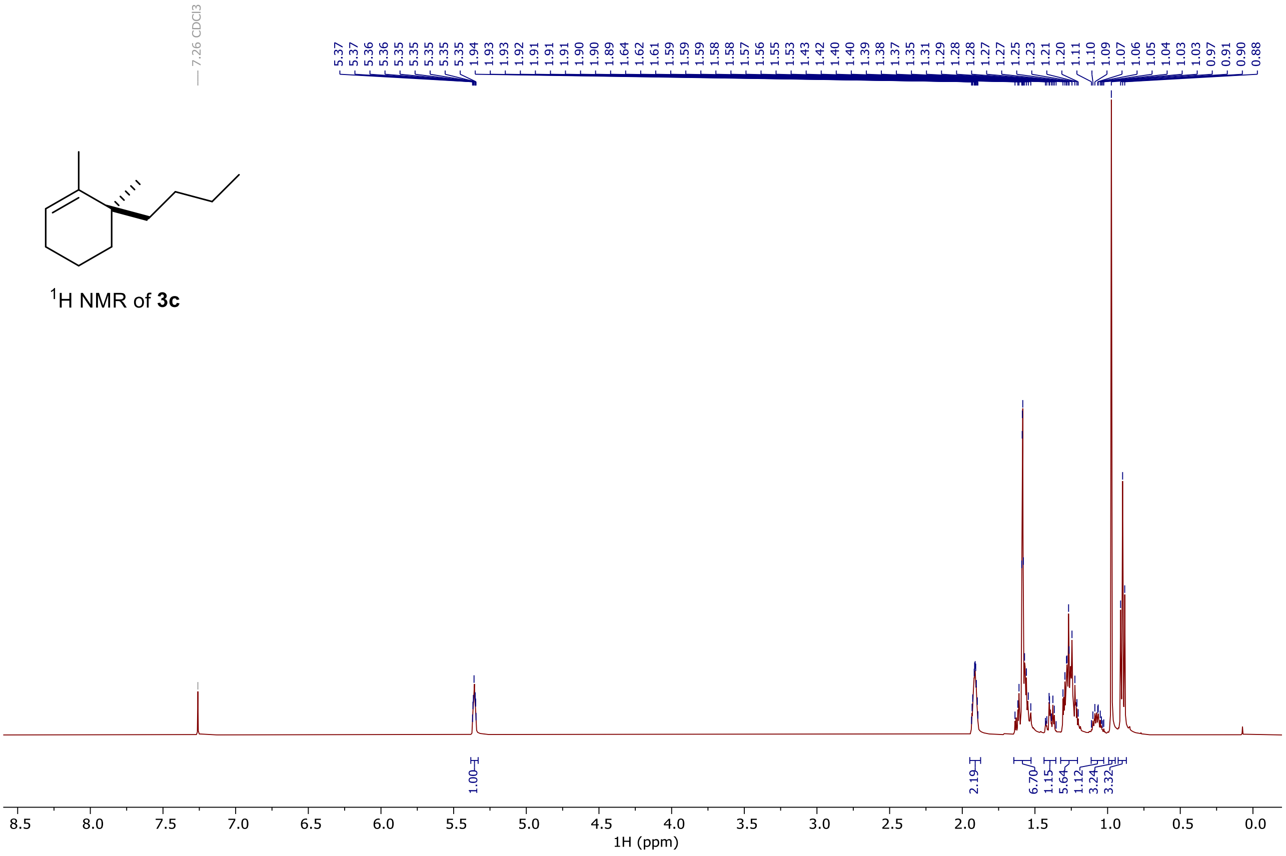
7.26 CDCl3
5.37 5.37 5.36 5.36 5.35 5.35 5.35 5.35 1.94 1.93 1.93 1.92 1.91 1.91 1.91 1.90 1.89 1.64 1.62 1.61 1.59 1.59 1.59 1.58 1.58 1.57 1.56 1.55 1.53 1.43 1.42 1.40 1.40 1.39 1.38 1.37 1.35 1.31 1.29 1.28 1.28 1.27 1.27 1.25 1.23 1.21 1.20 1.11 1.10 1.09 1.07 1.06 1.05 1.04 1.03 1.03 0.97 0.91 0.90 0.88
1H NMR of 3c
1.00
2.19
6.70
1.15
5.64
1.12
3.24
3.32
8.5 8.0 7.5 7.0 6.5 6.0 5.5 5.0 4.5 4.0 3.5 3.0 2.5 2.0 1.5 1.0 0.5 0.0
1H (ppm)

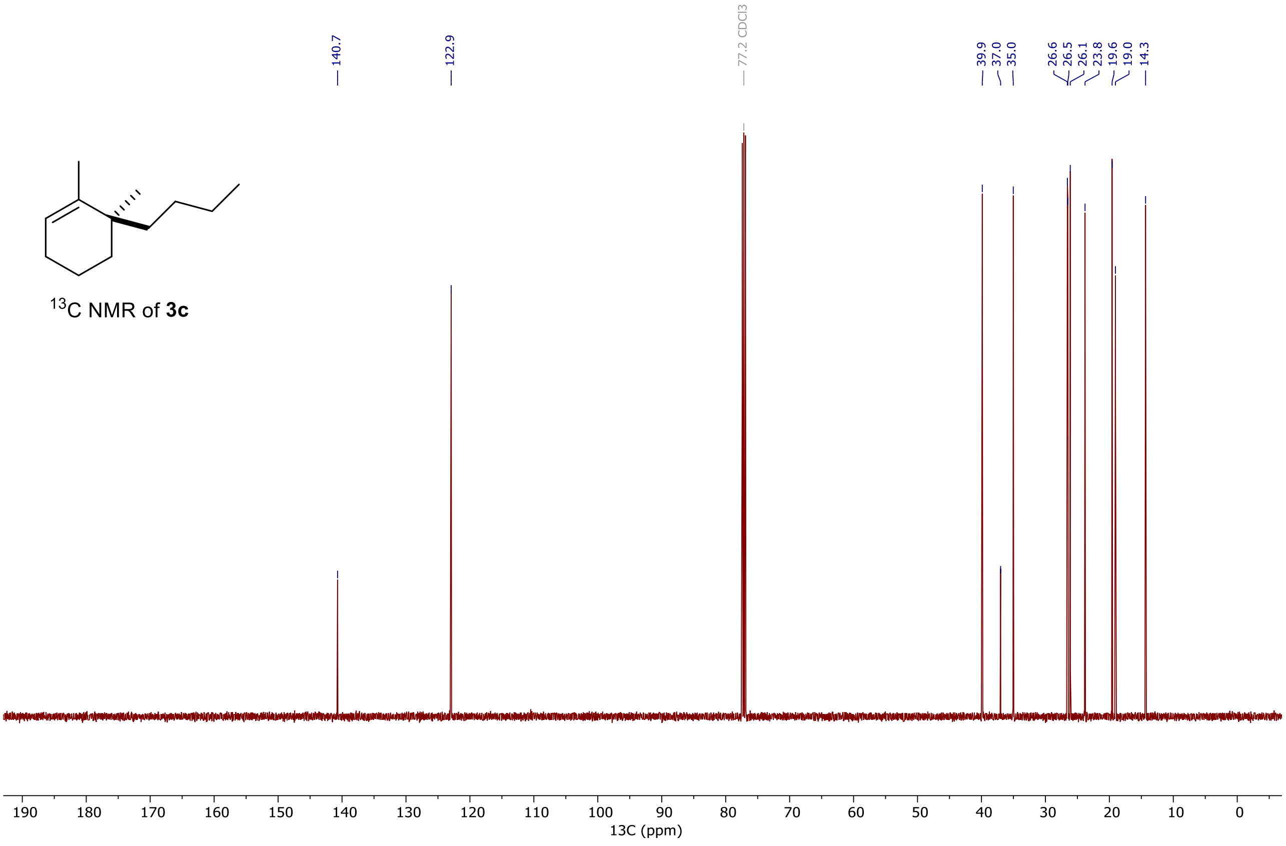
140.7
122.9
77.2 CDCl3
39.9 37.0 35.0 26.6 26.5 26.1 23.8 19.6 19.0 14.3
13C NMR of 3c
190 180 170 160 150 140 130 120 110 100 90 80 70 60 50 40 30 20 10 0
13C (ppm)

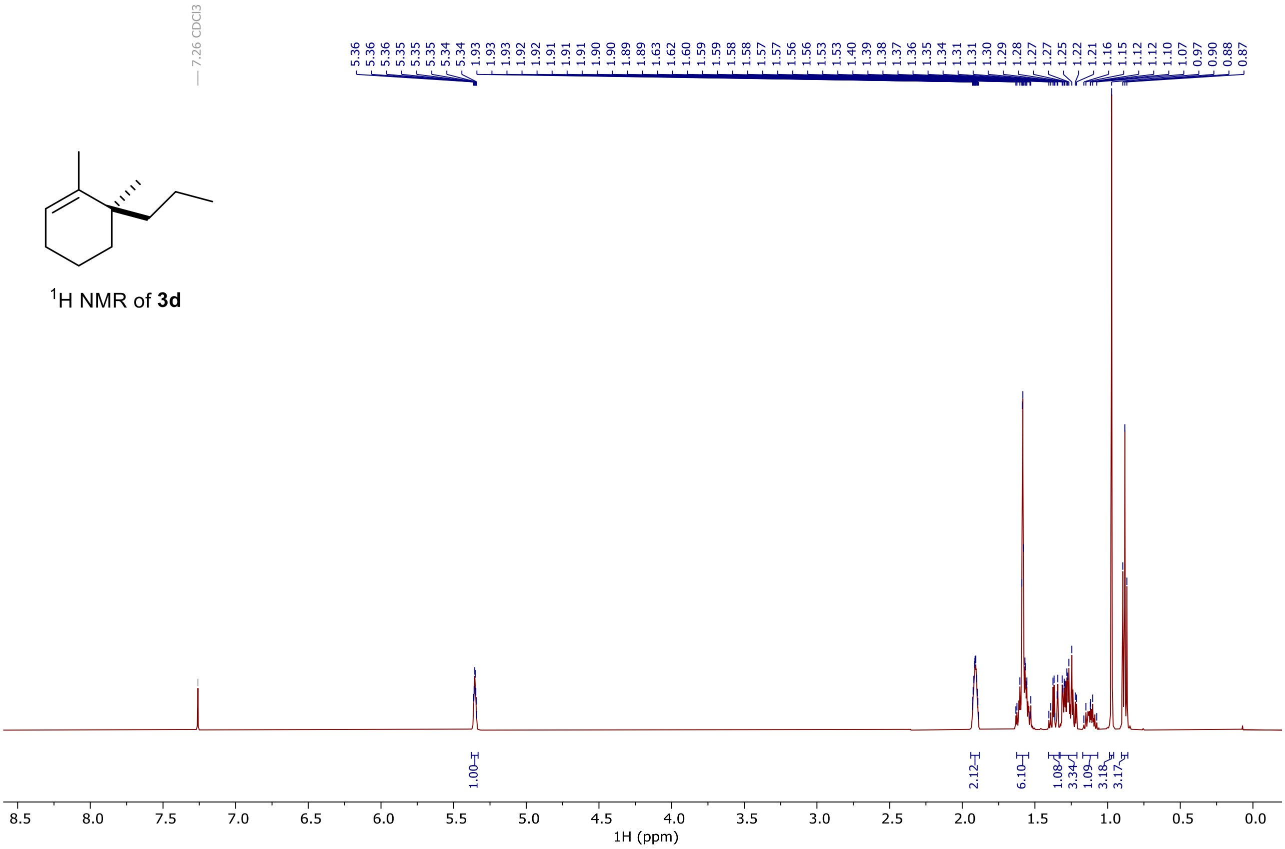

1H NMR of 3d
7.26 CDCl3
1H (ppm)

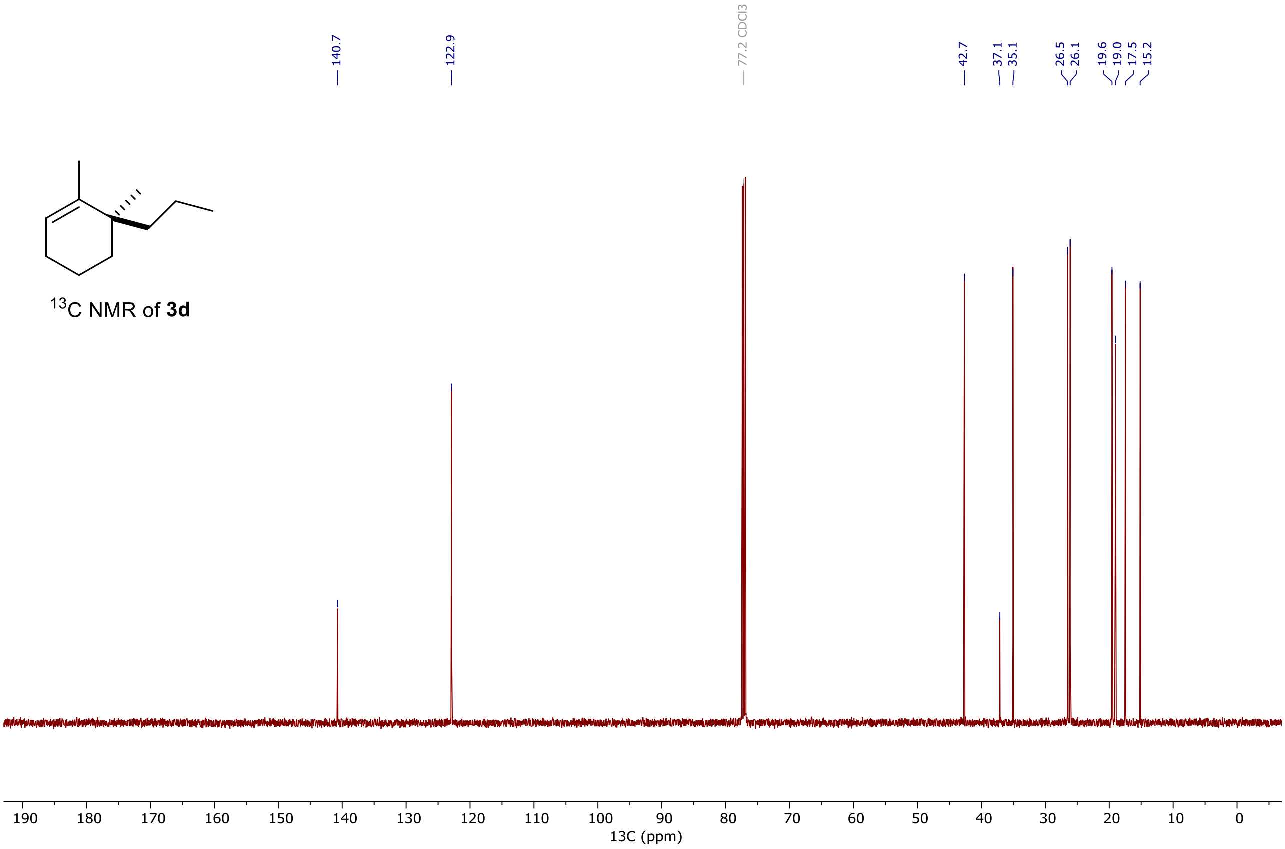

13C NMR of 3d
140.7
122.9
77.2 CDCl3
42.7
37.1
35.1
26.5
26.1
19.6
19.0
17.5
15.2
13C (ppm)

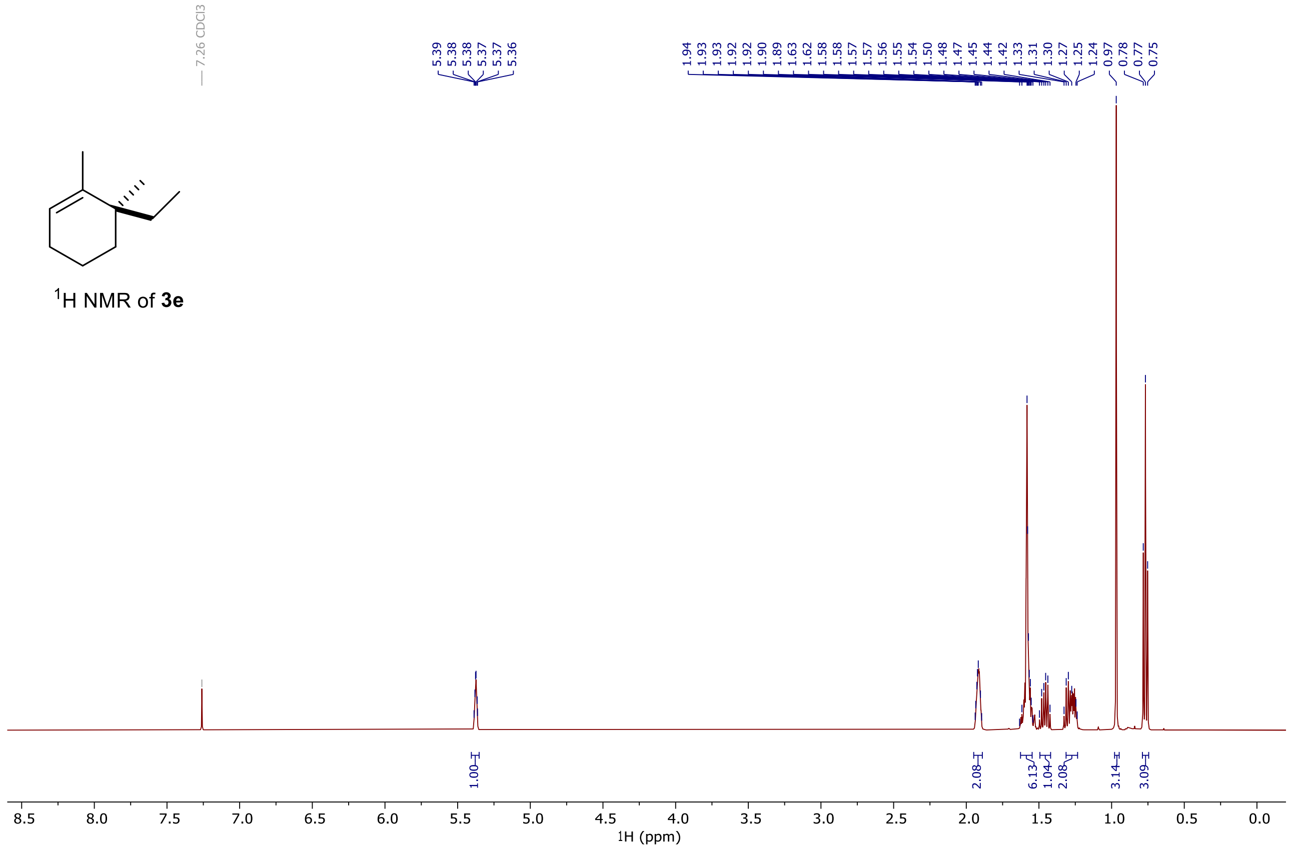
7.26 CDCl3
5.39
5.38
5.38
5.37
5.37
5.36
1.94
1.93
1.93
1.92
1.92
1.90
1.89
1.63
1.62
1.58
1.58
1.57
1.57
1.56
1.55
1.54
1.50
1.48
1.47
1.45
1.44
1.42
1.33
1.31
1.30
1.27
1.25
1.24
0.97
0.78
0.77
0.75
1H NMR of 3e
1.00
2.08
6.13
1.04
2.08
3.14
3.09
1H (ppm)

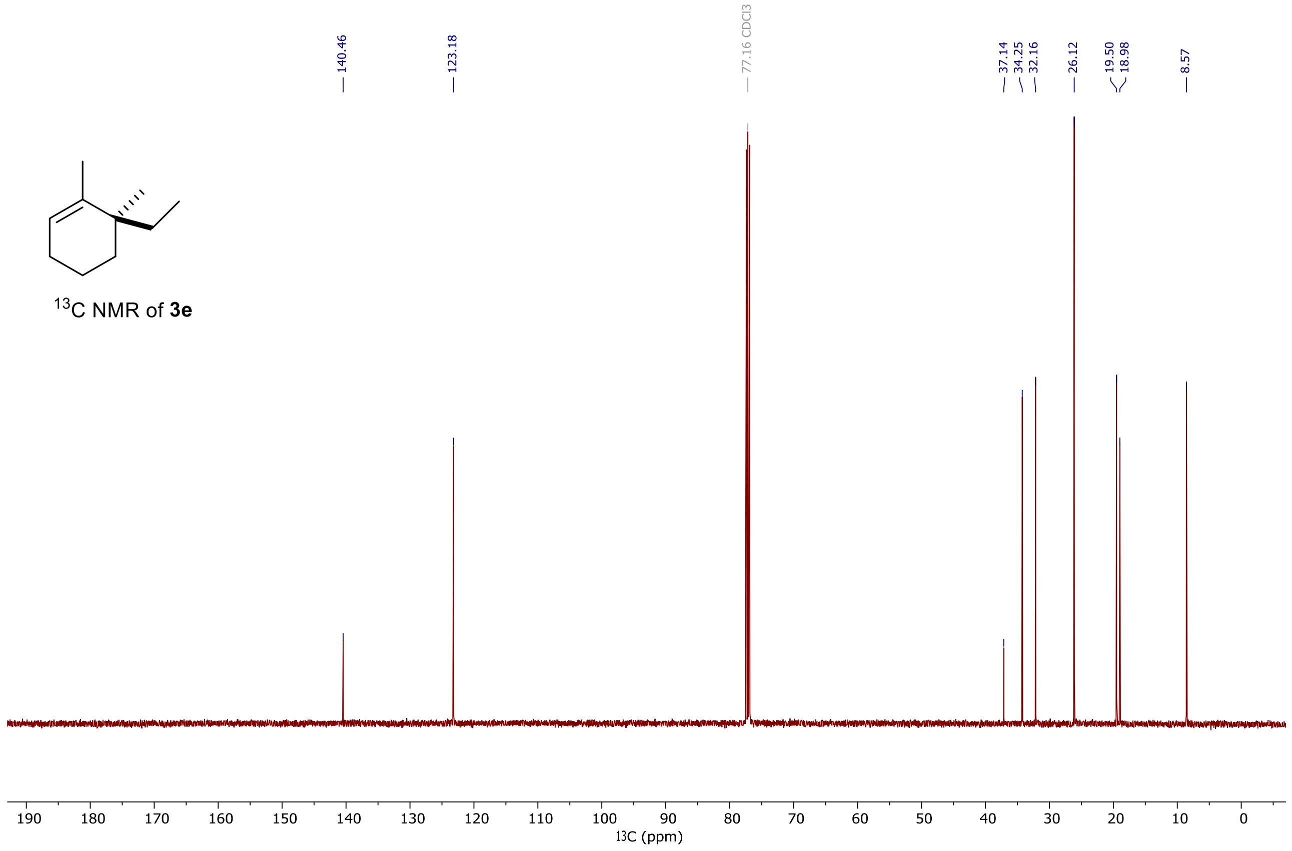
140.46
123.18
77.16 CDCl3
37.14
34.25
32.16
26.12
19.50
18.98
8.57
13C NMR of 3e
13C (ppm)

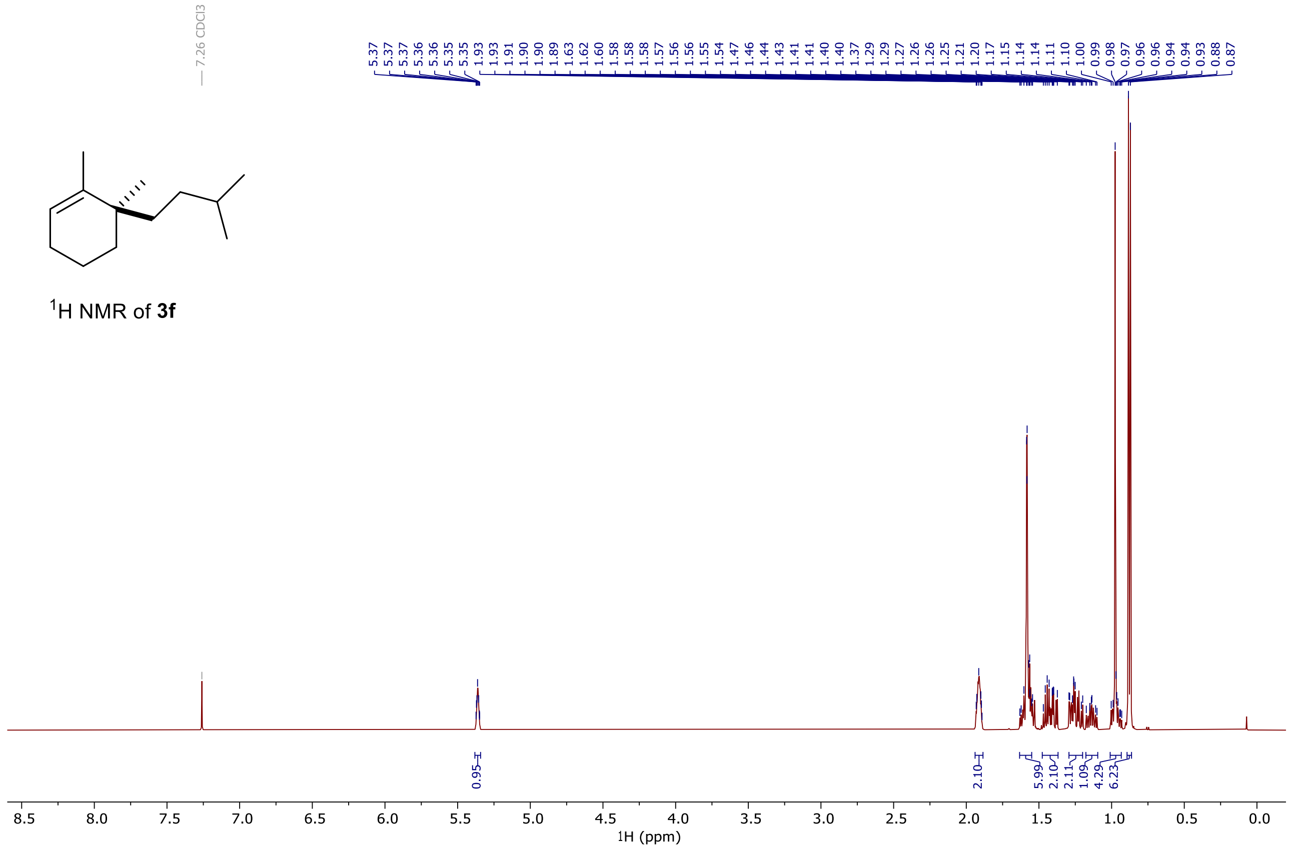
7.26 CDCl3
5.37 5.37 5.37 5.36 5.36 5.35 5.35 1.93 1.93 1.91 1.90 1.90 1.89 1.63 1.62 1.60 1.58 1.58 1.57 1.56 1.56 1.55 1.54 1.47 1.46 1.44 1.43 1.41 1.41 1.40 1.40 1.37 1.29 1.29 1.27 1.26 1.26 1.25 1.21 1.20 1.17 1.15 1.14 1.14 1.11 1.10 1.00 0.99 0.98 0.97 0.96 0.96 0.94 0.94 0.93 0.88 0.87
1H NMR of 3f
0.95
2.10
5.99
2.10
2.11
1.09
4.29
6.23
1H (ppm)

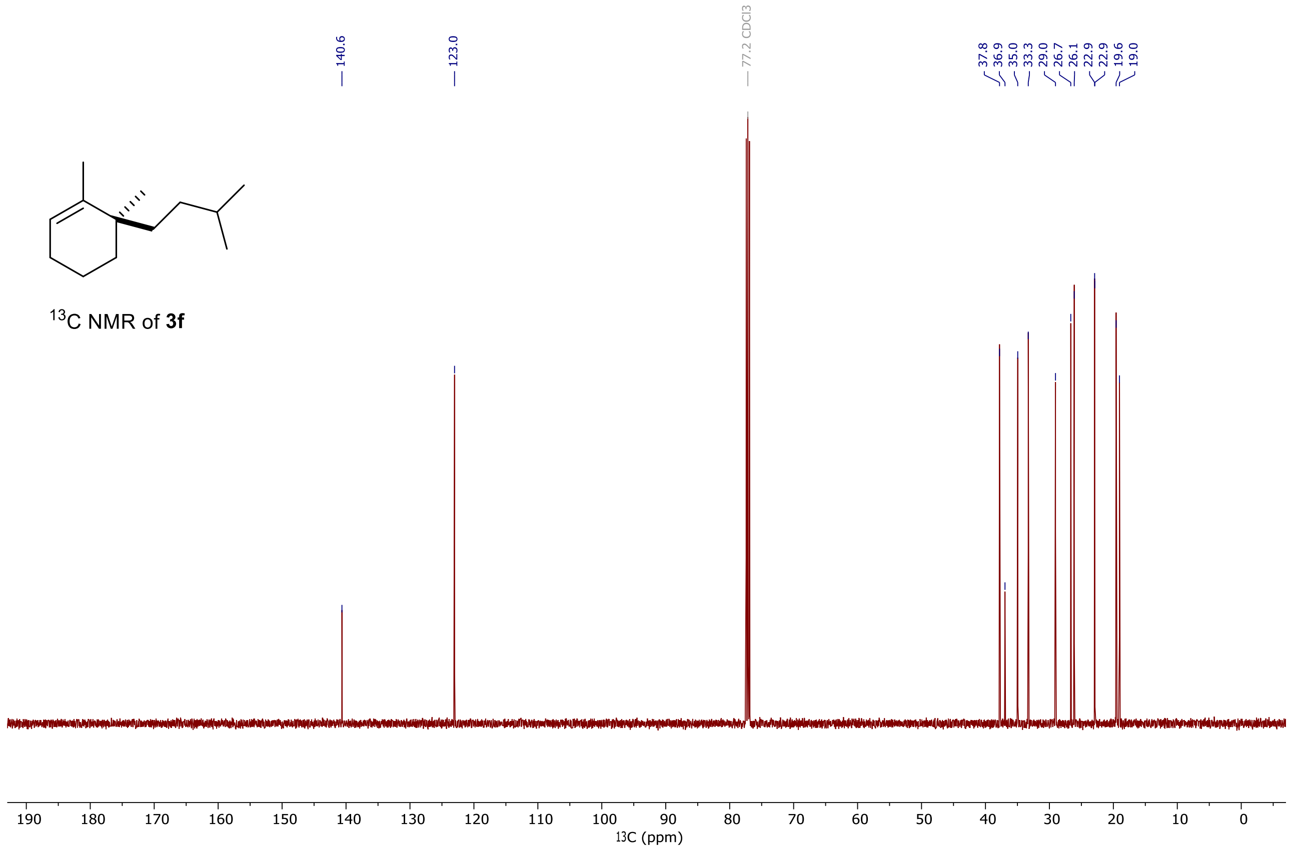
140.6
123.0
77.2 CDCl3
37.8 36.9 35.0 33.3 29.0 26.7 26.1 22.9 22.9 19.6 19.0
13C NMR of 3f
13C (ppm)

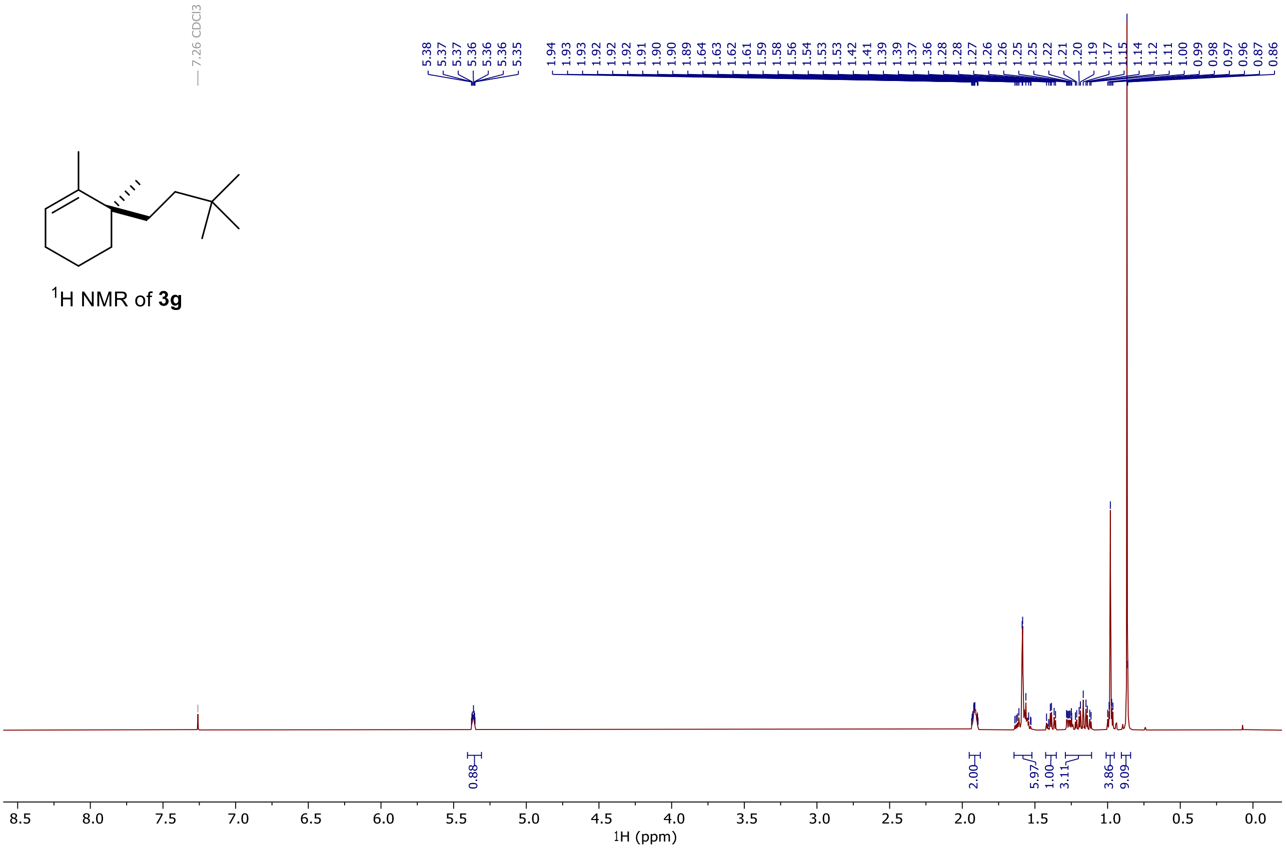

1H NMR of 3g
7.26 CDCl3
1H (ppm)

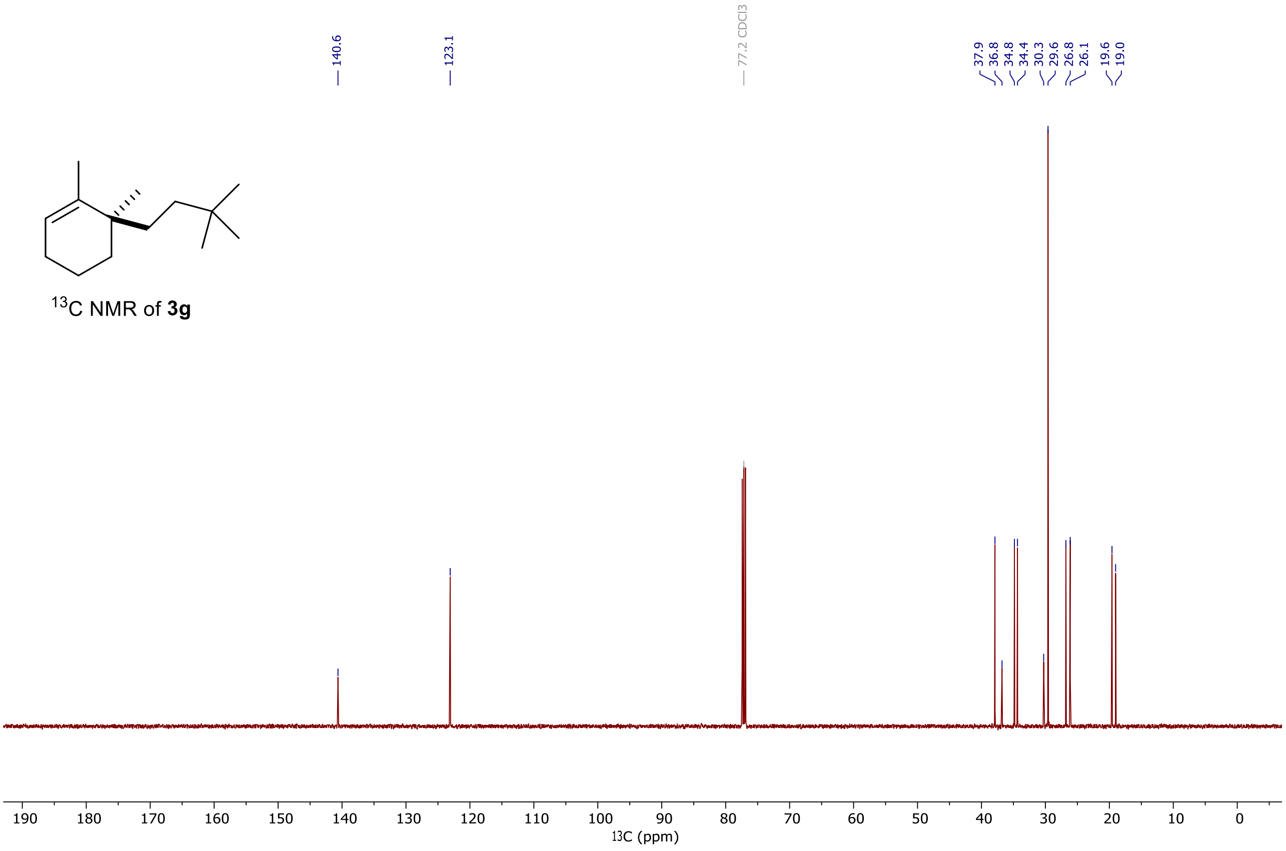

13C NMR of 3g
140.6
123.1
77.2 CDCl3
37.9
36.8
34.8
34.4
30.3
29.6
26.8
26.1
19.6
19.0
13C (ppm)

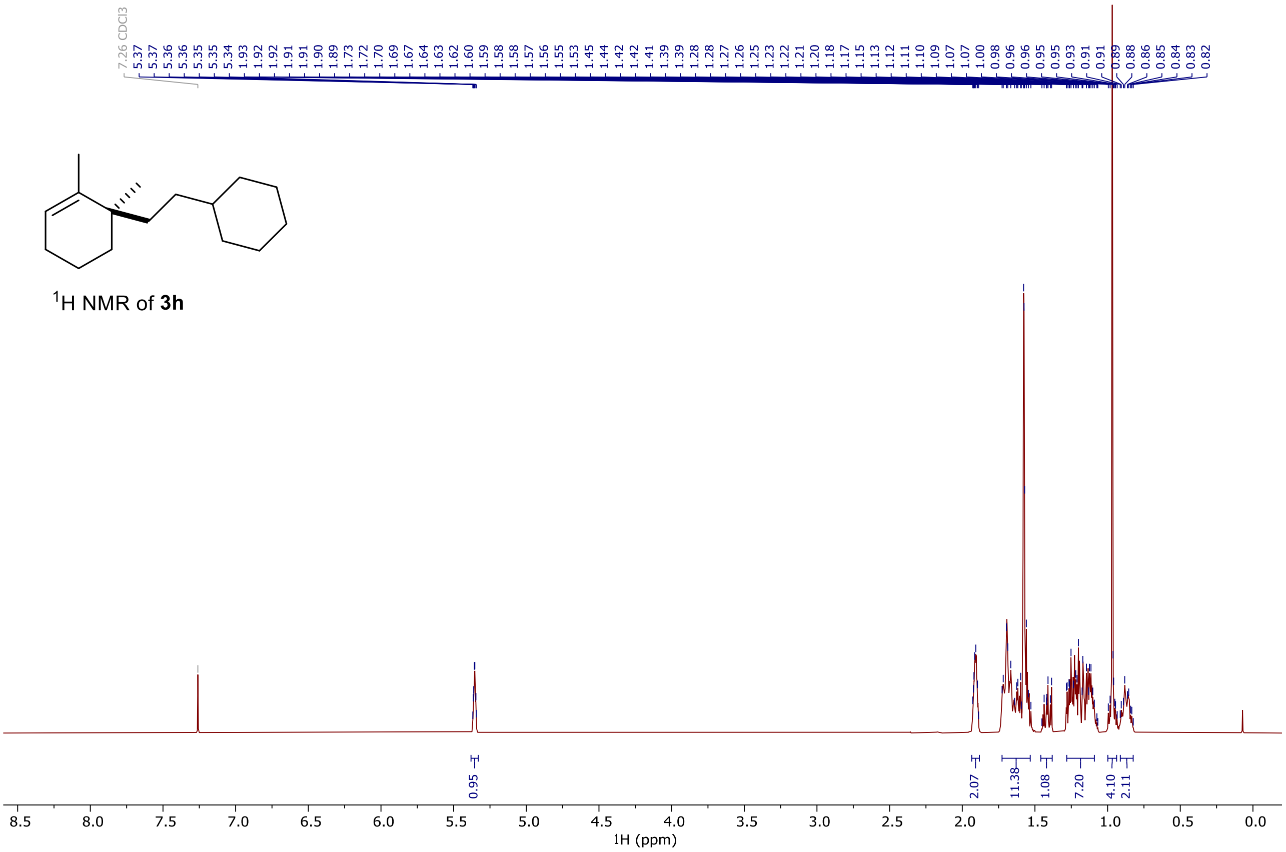

^{1}H NMR of **3h**

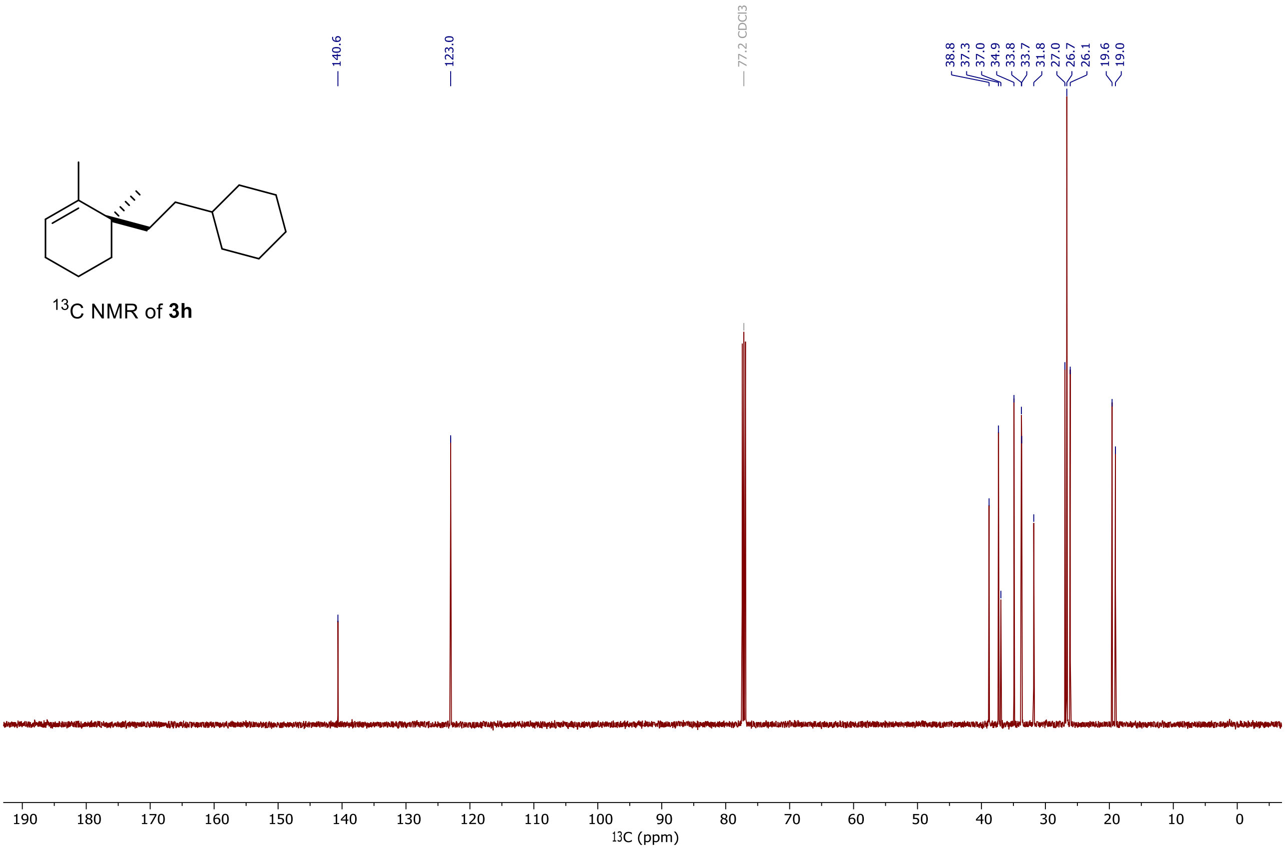

^{13}C NMR of **3h**

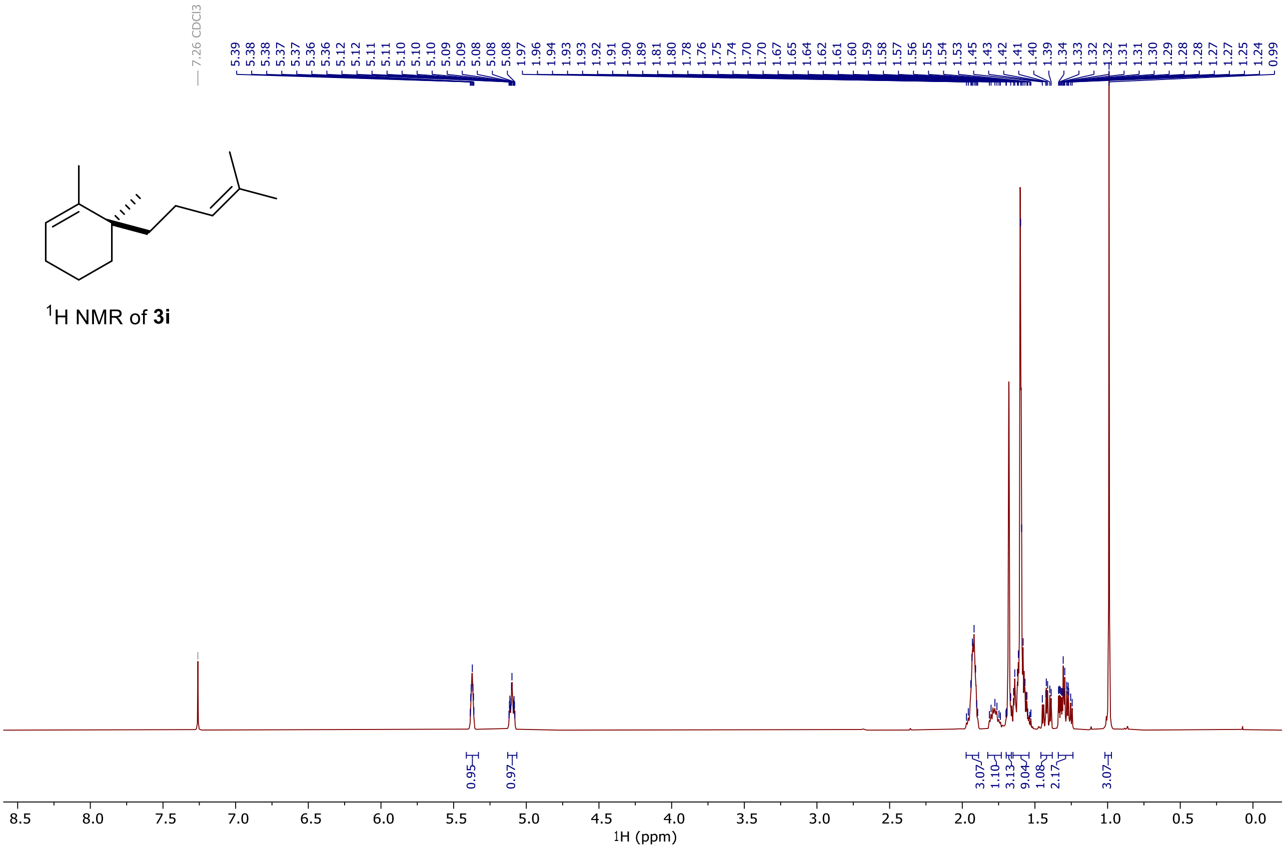
^{1}H NMR of **3i**

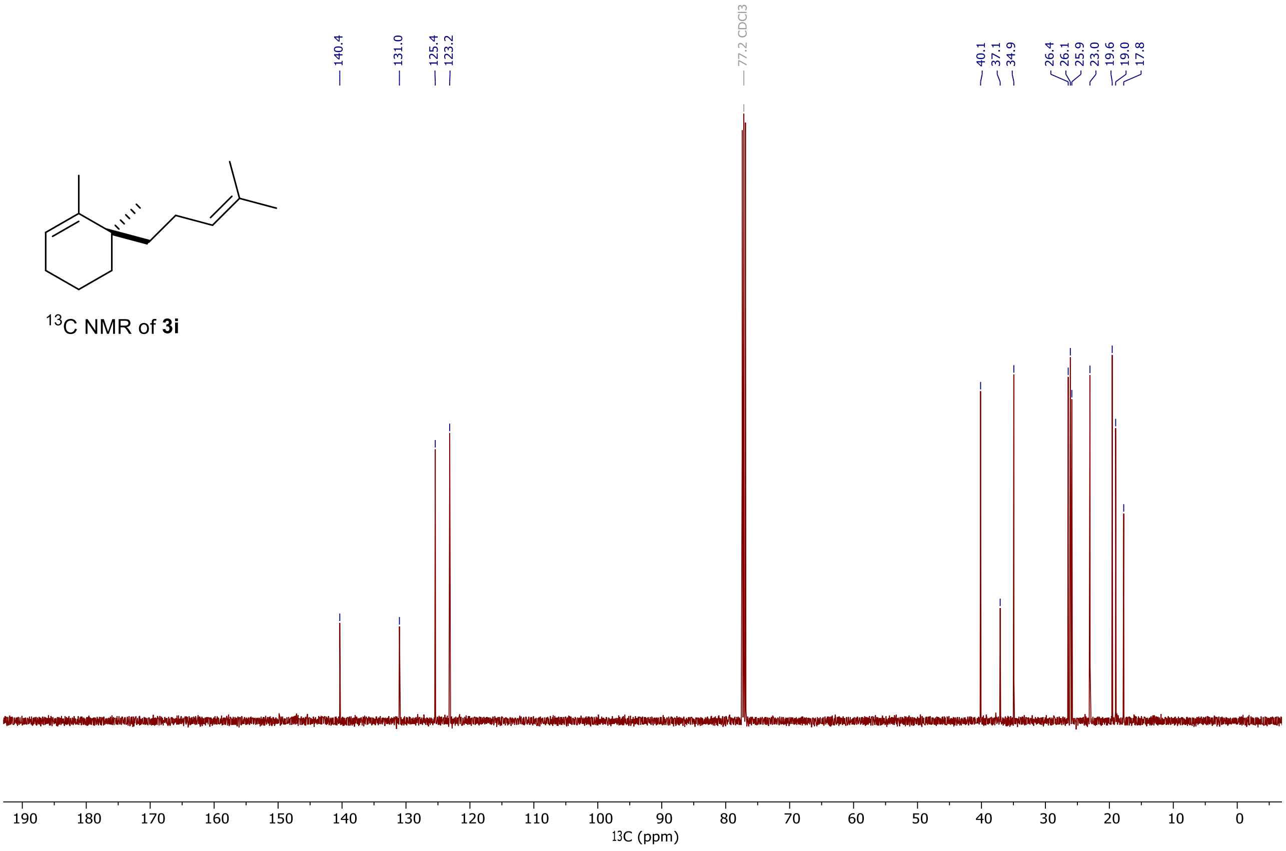
^{13}C NMR of **3i**

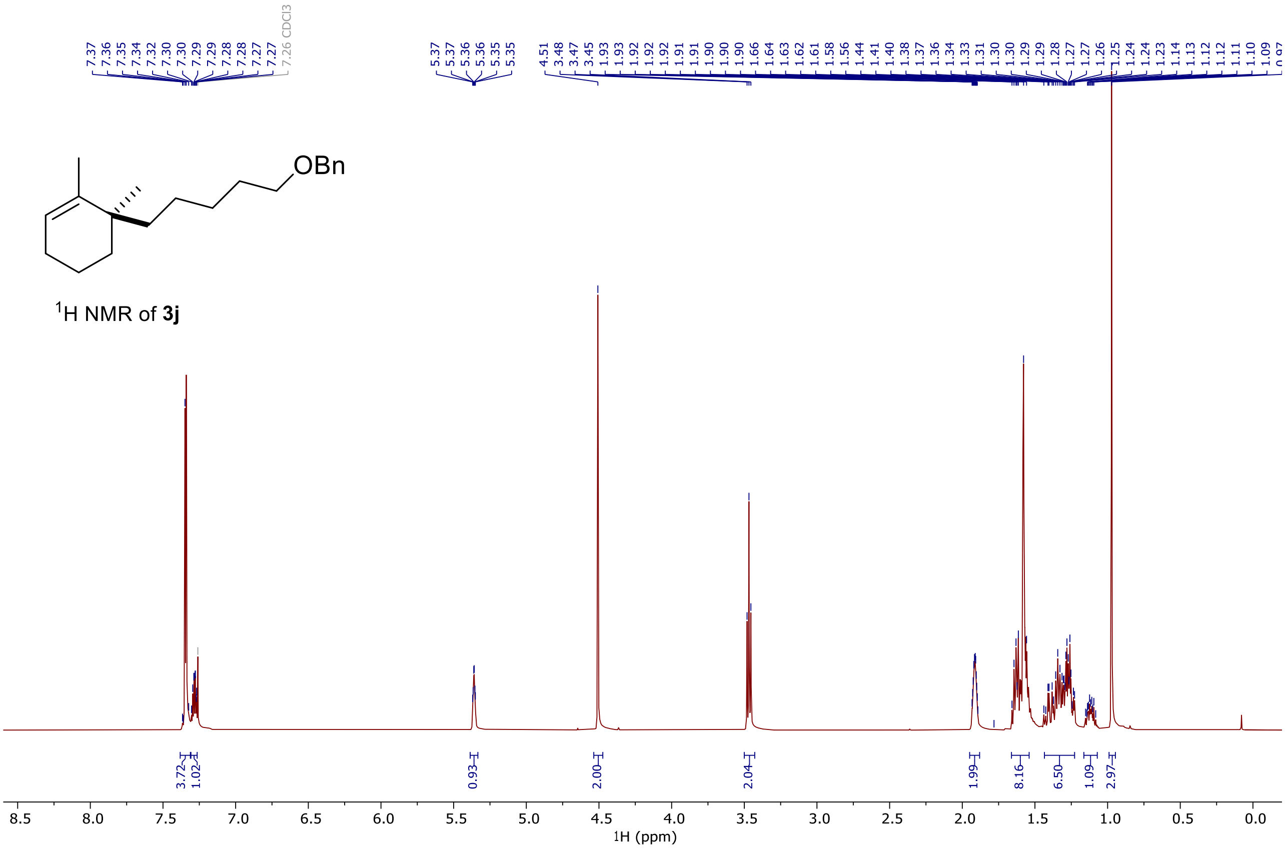

^{1}H NMR of **3j**

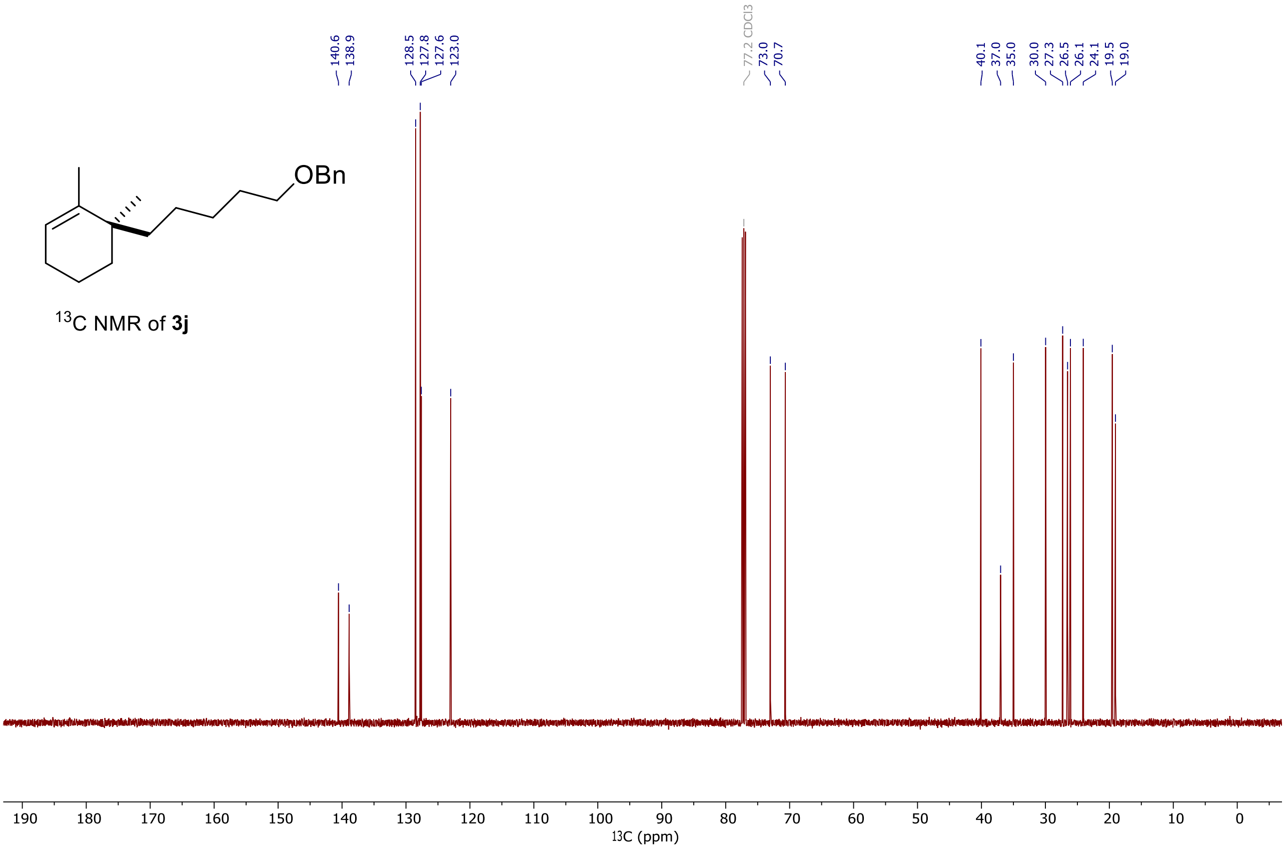

^{13}C NMR of **3j**

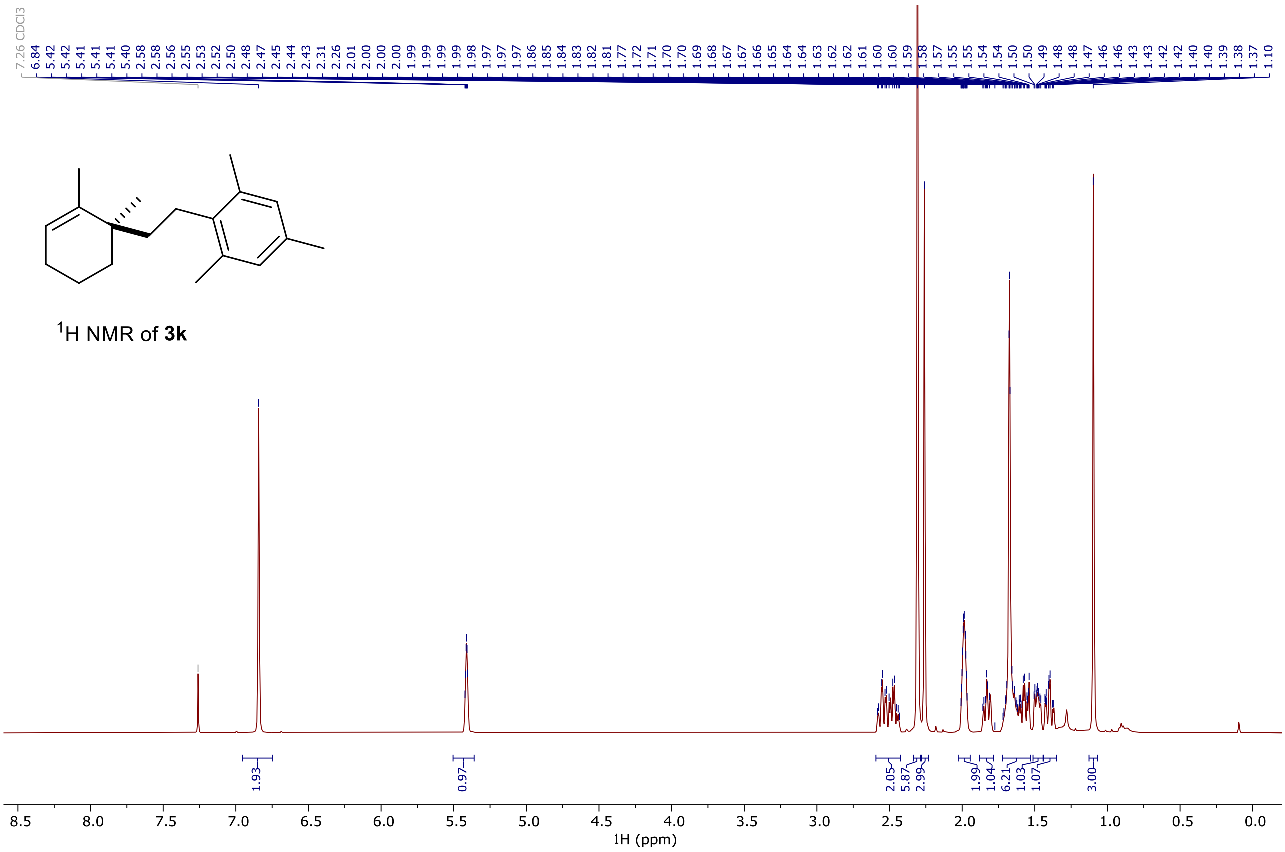

^{1}H NMR of **3k**

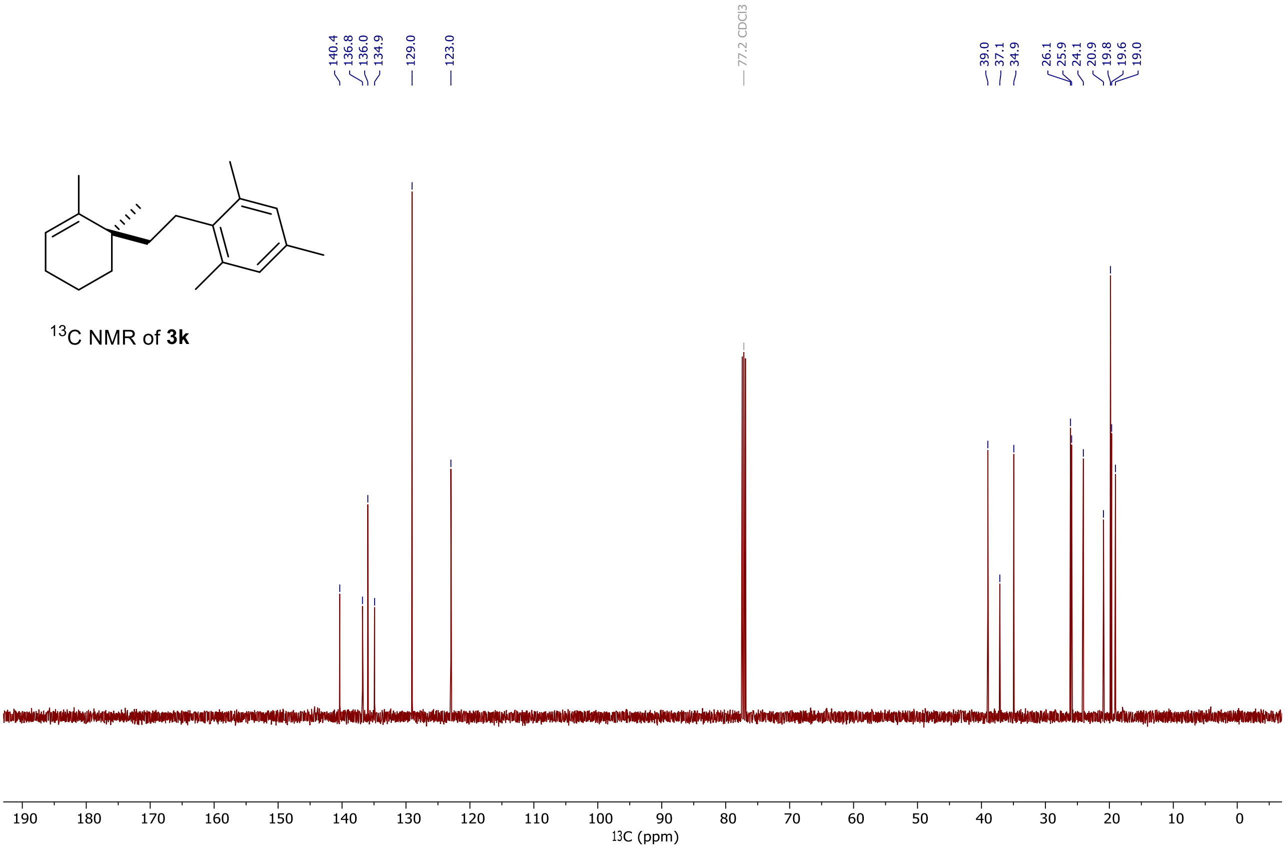

^{13}C NMR of **3k**

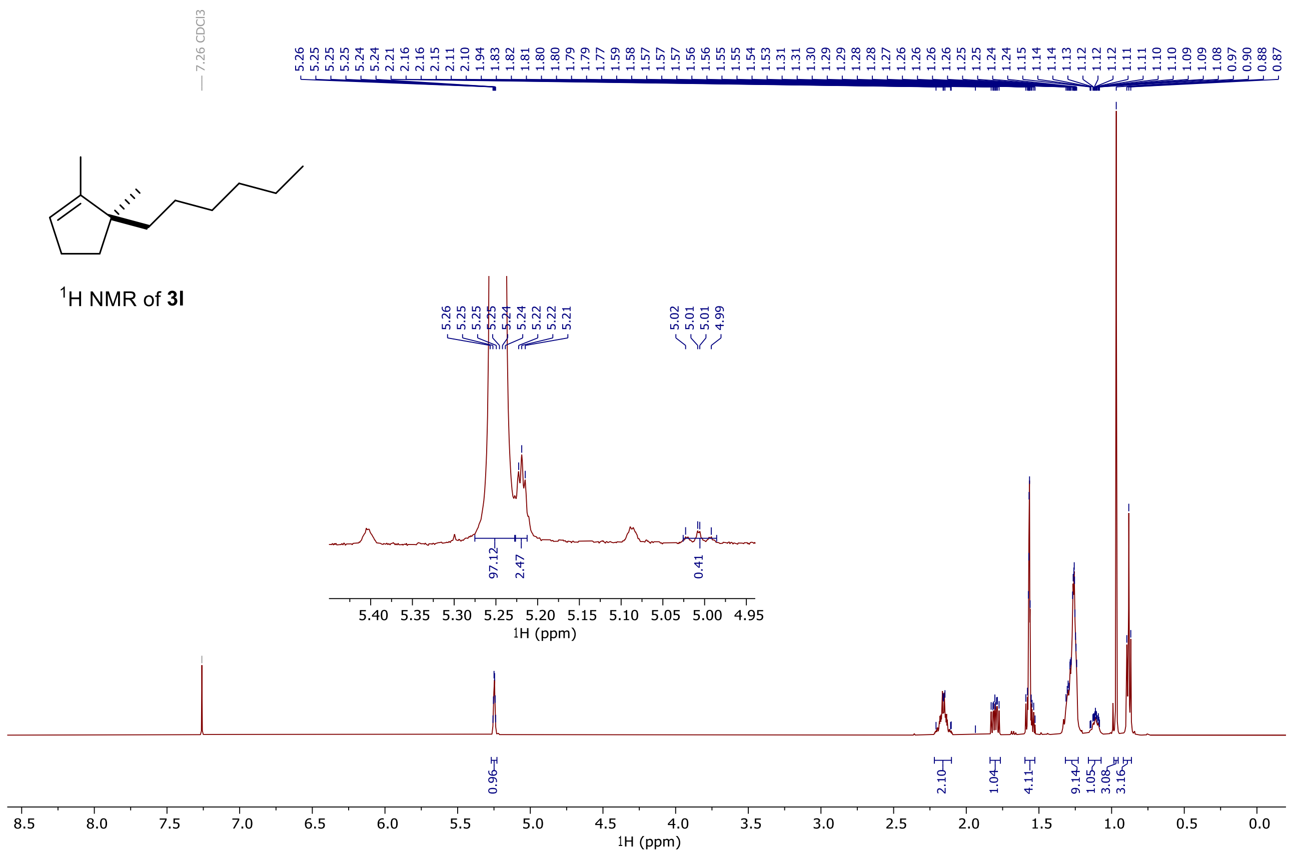

1H NMR of 3l
7.26 CDCl3
97.12
2.47
0.41
0.96
2.10
1.04
4.11
9.14
1.05
3.08
3.16
1H (ppm)

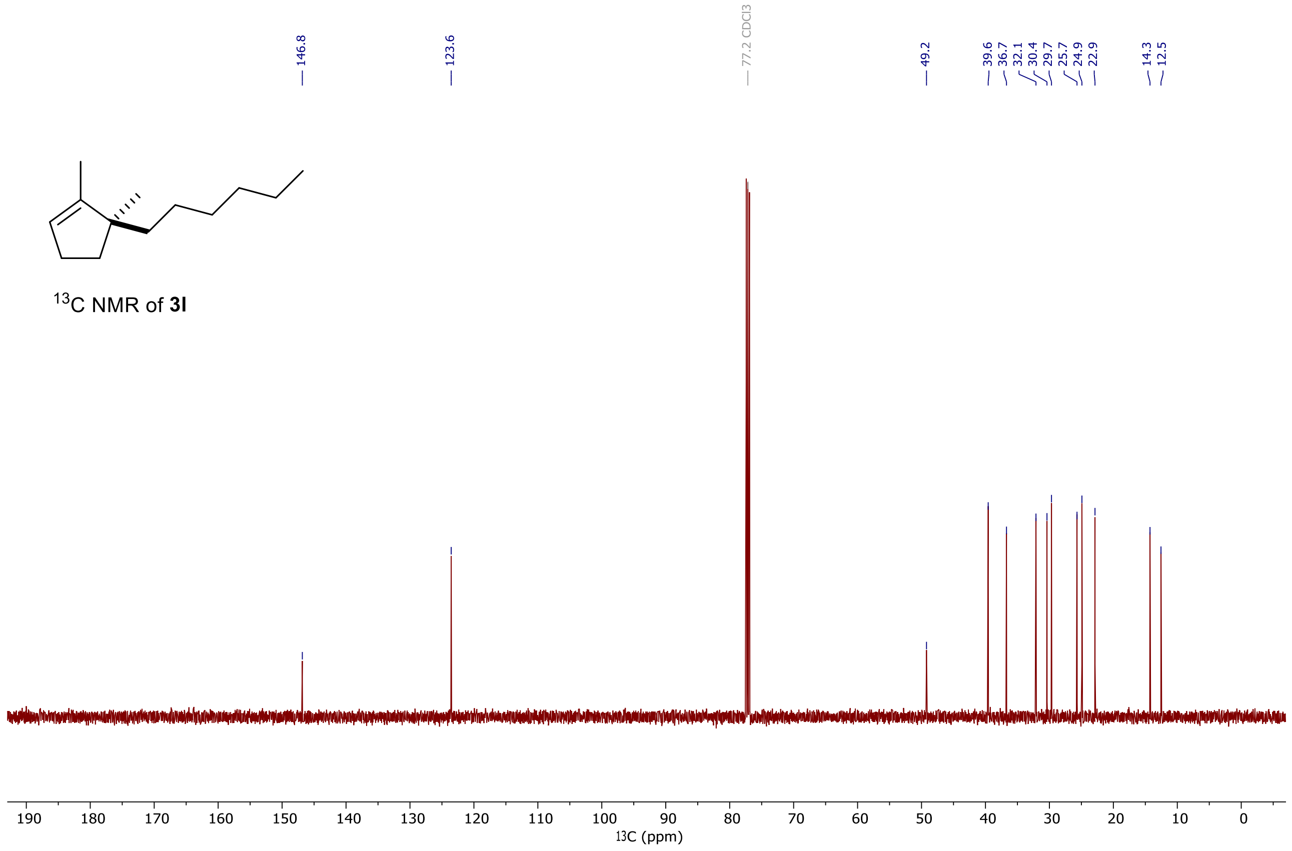

13C NMR of 3l
146.8
123.6
77.2 CDCl3
49.2
39.6
36.7
32.1
30.4
29.7
25.7
24.9
22.9
14.3
12.5
13C (ppm)

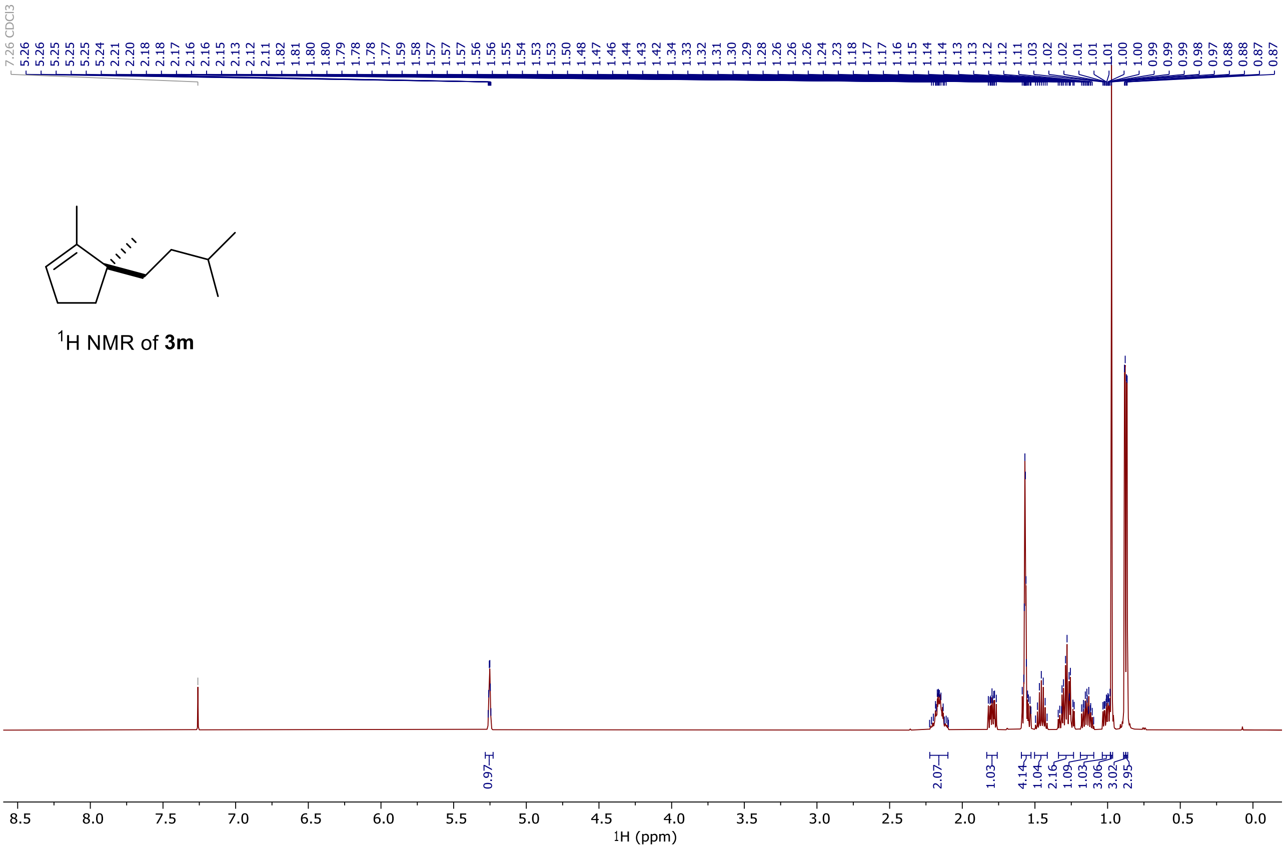
1H NMR of 3m
7.26 CDCl3
0.97
2.07
1.03
4.14
1.04
2.16
1.09
1.03
3.06
3.02
2.95
1H (ppm)

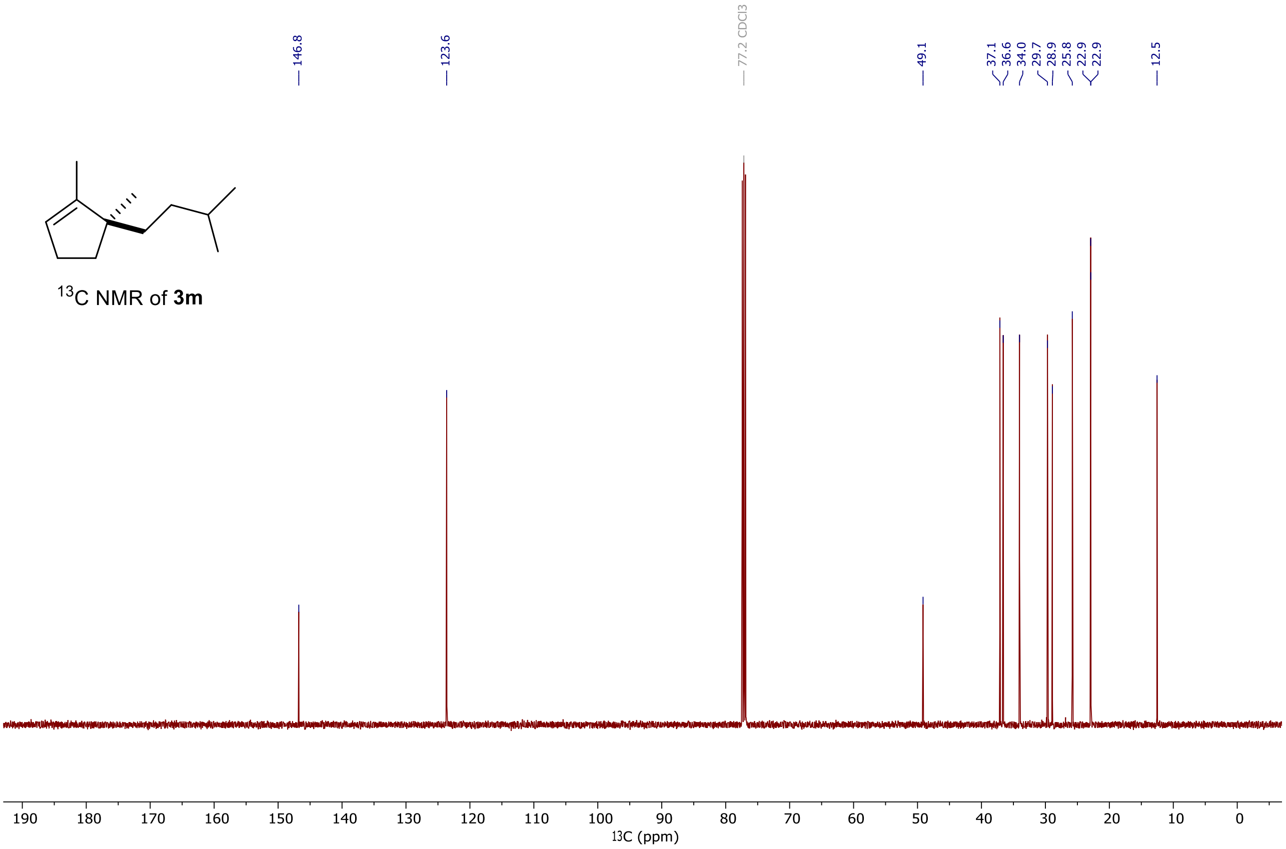
13C NMR of 3m
146.8
123.6
77.2 CDCl3
49.1
37.1
36.6
34.0
29.7
28.9
25.8
22.9
22.9
12.5
13C (ppm)

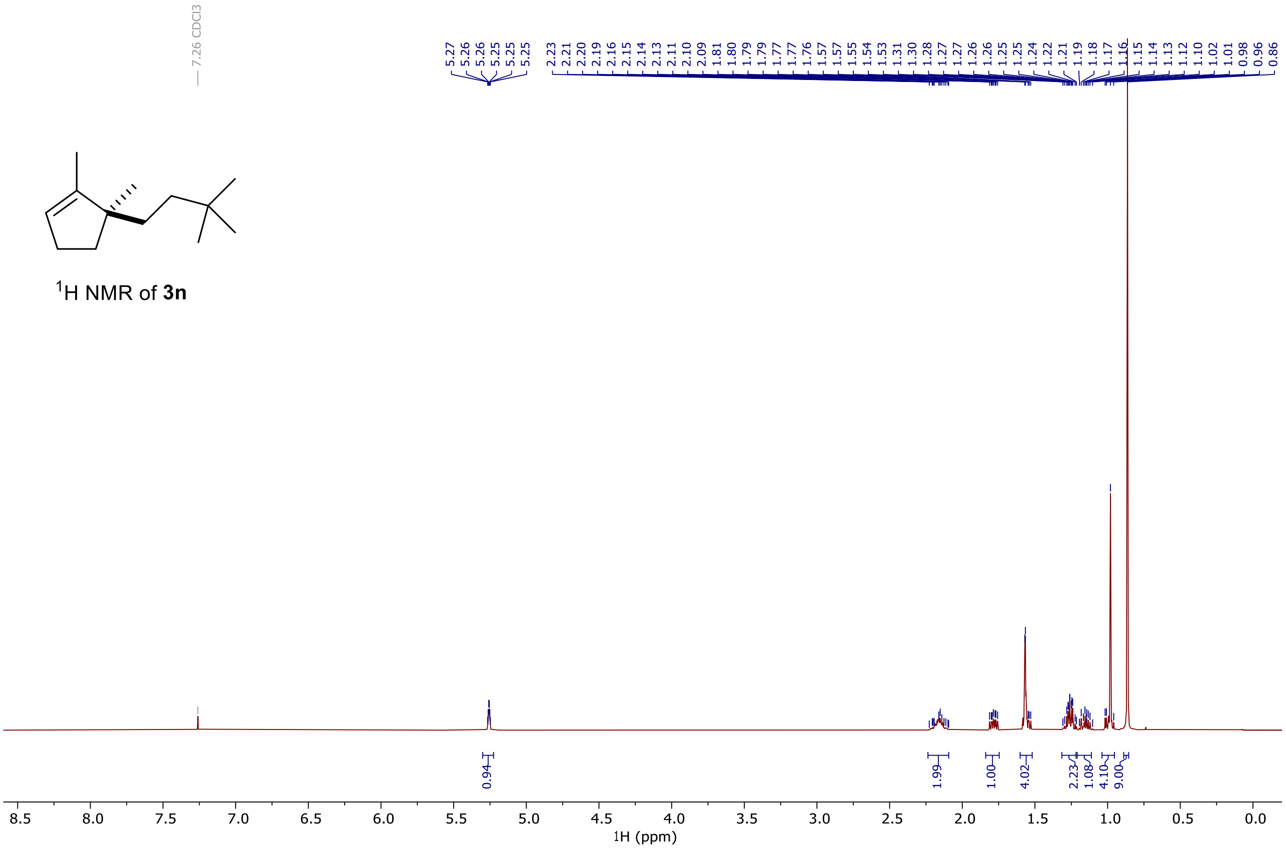

^{1}H NMR of **3n**
7.26 CDCl3
1H (ppm)

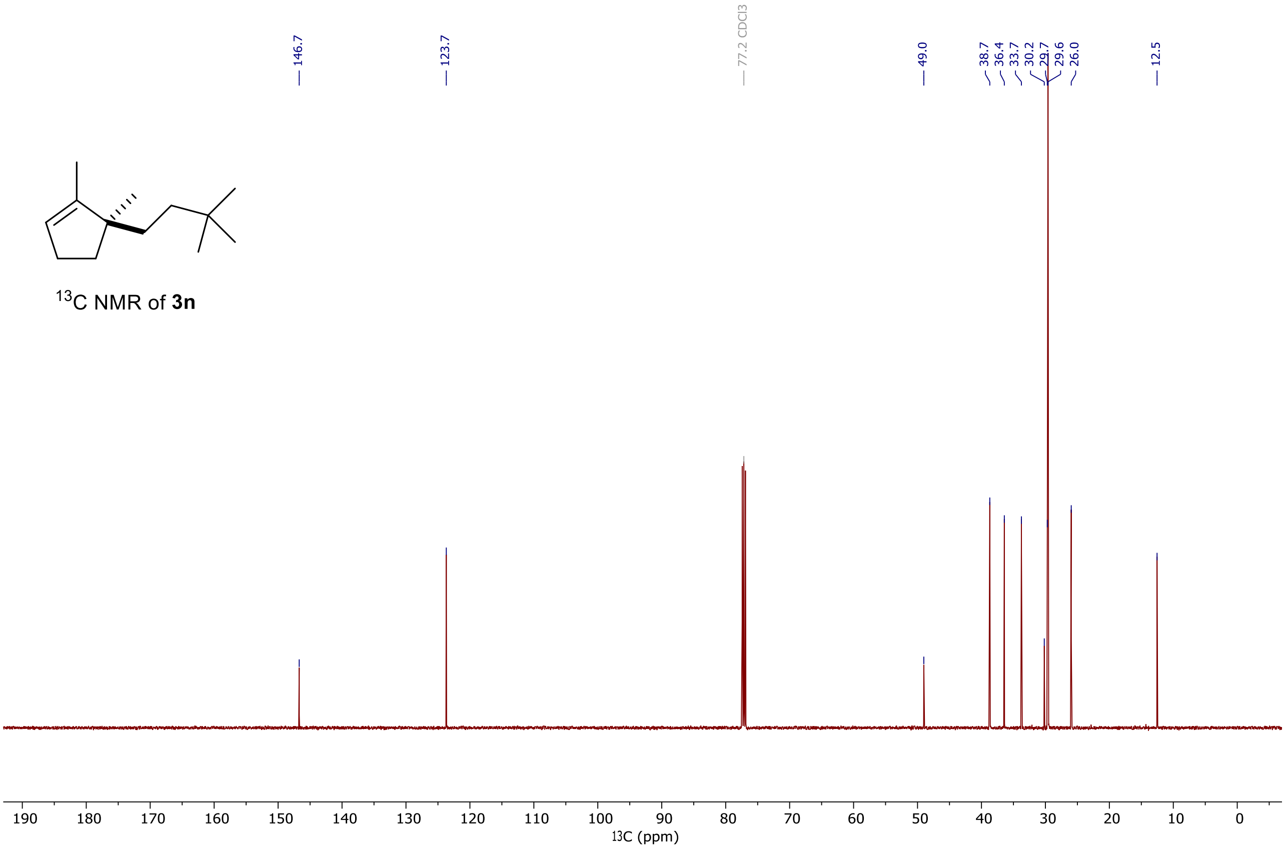

^{13}C NMR of **3n**
146.7
123.7
77.2 CDCl3
49.0
38.7
36.4
33.7
30.2
29.7
29.6
26.0
12.5
13C (ppm)

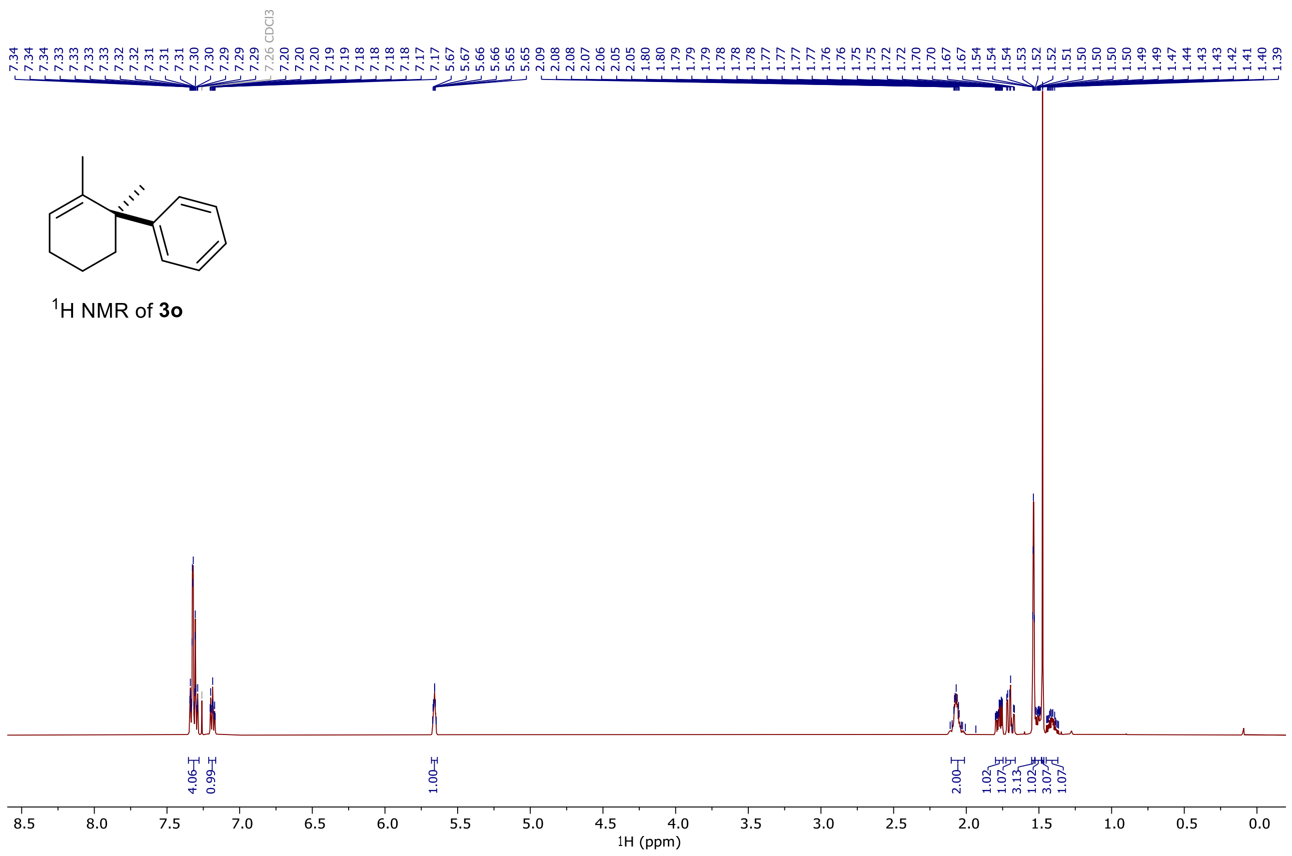

1H NMR of 3o
4.06
0.99
1.00
2.00
1.02
1.07
3.13
1.02
3.07
1.07
8.5 8.0 7.5 7.0 6.5 6.0 5.5 5.0 4.5 4.0 3.5 3.0 2.5 2.0 1.5 1.0 0.5 0.0
1H (ppm)

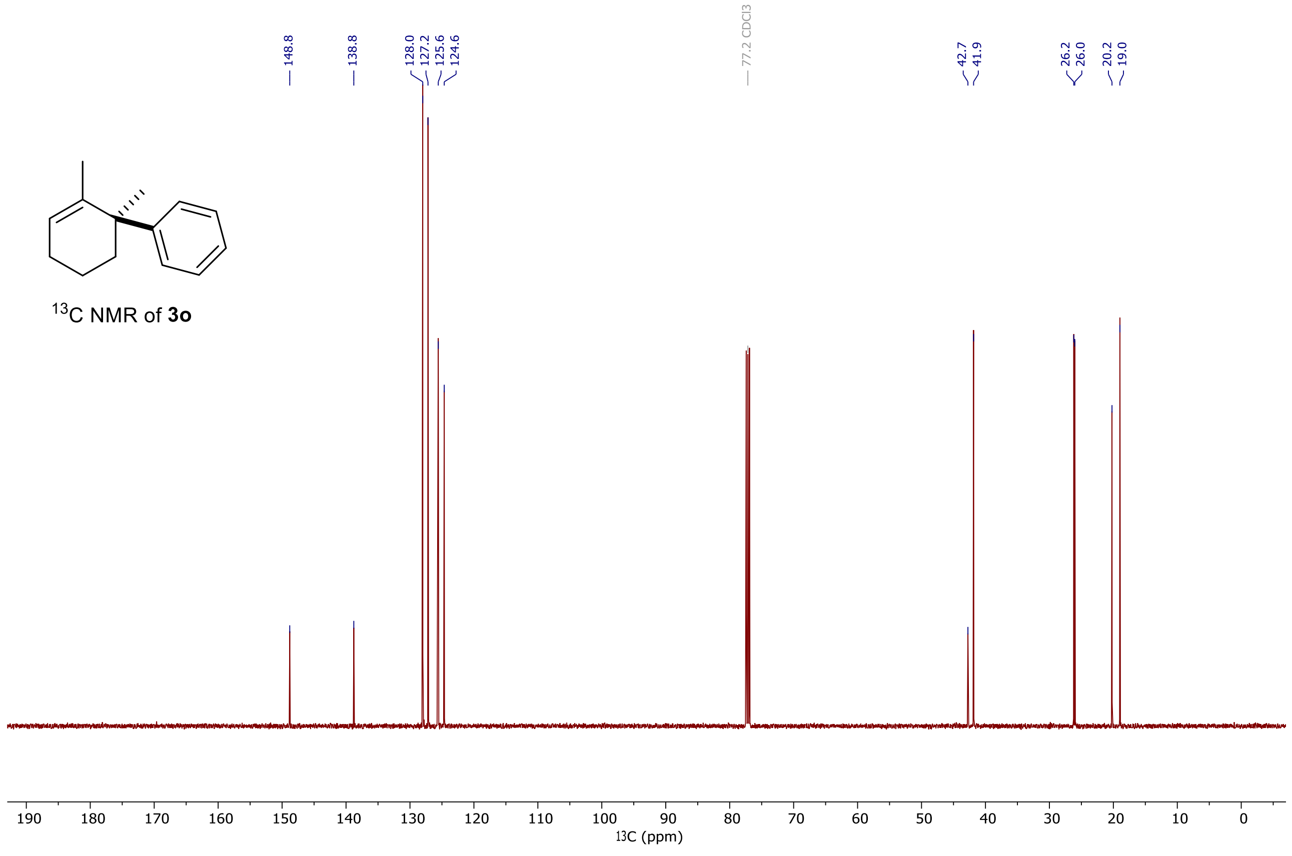

13C NMR of 3o
148.8
138.8
128.0
127.2
125.6
124.6
77.2 CDCl3
42.7
41.9
26.2
26.0
20.2
19.0
190 180 170 160 150 140 130 120 110 100 90 80 70 60 50 40 30 20 10 0
13C (ppm)

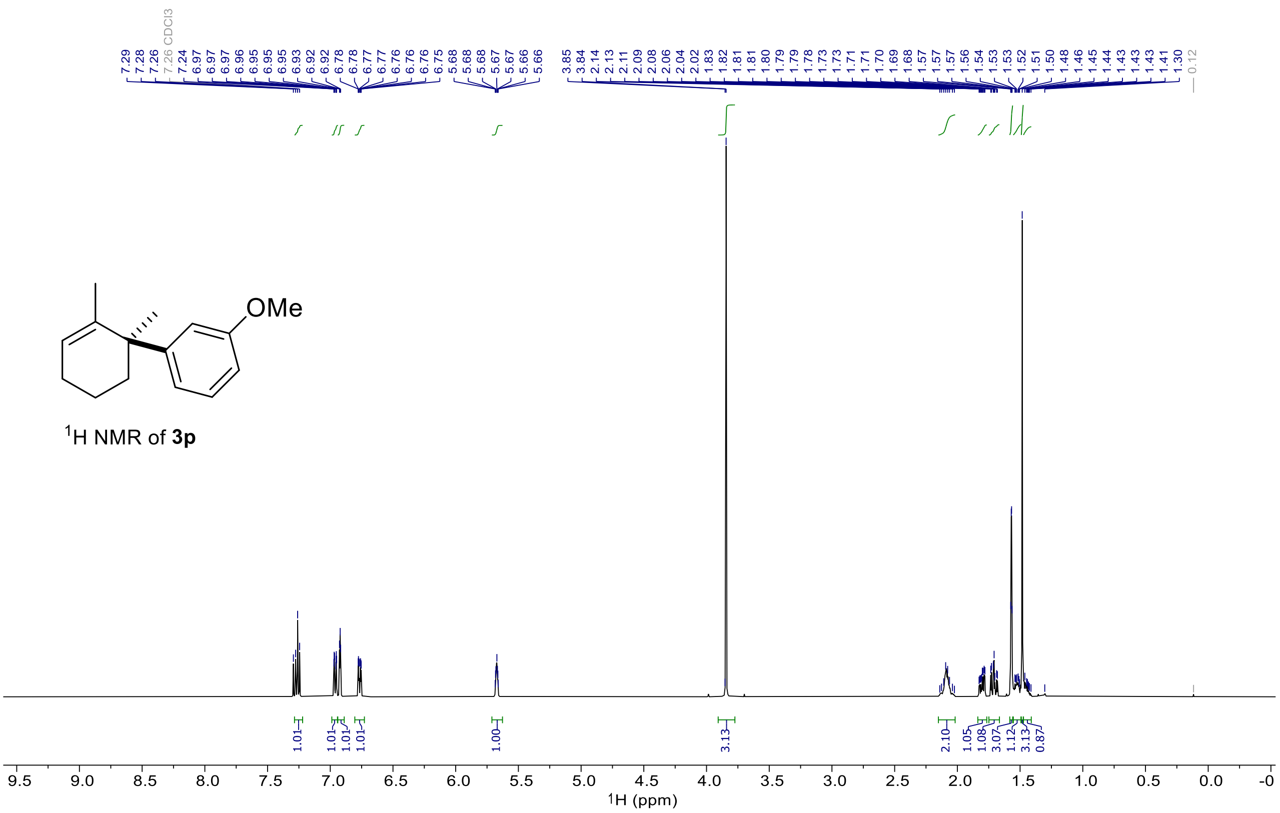

^{1}H NMR of **3p**

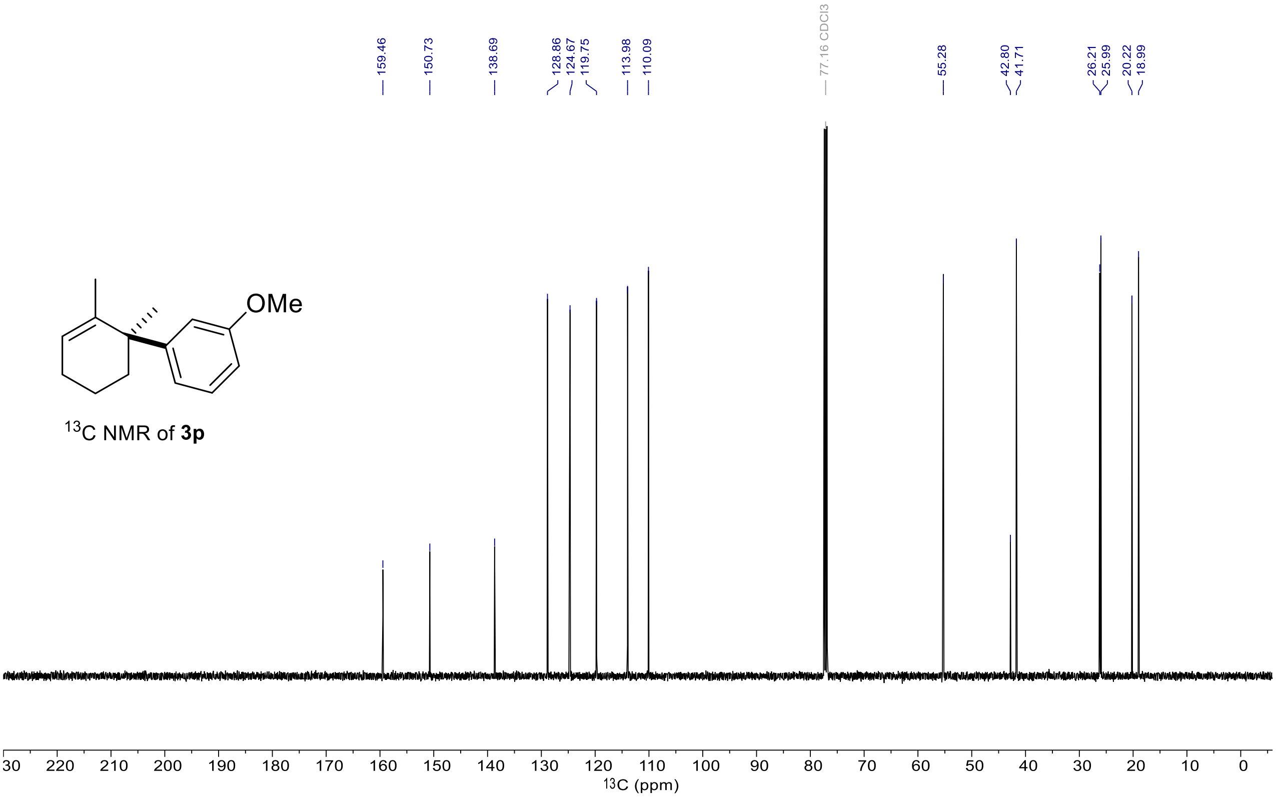

^{13}C NMR of **3p**

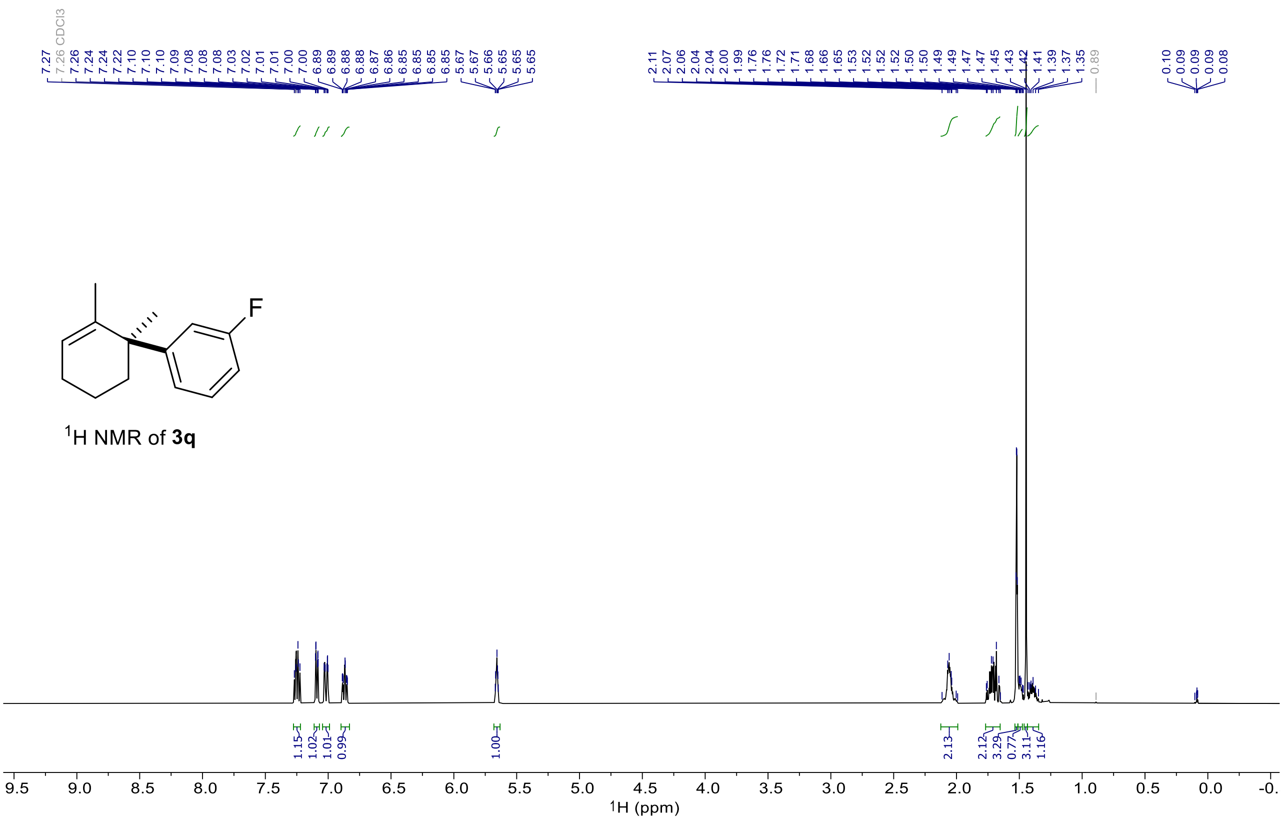

^{1}H NMR of **3q**

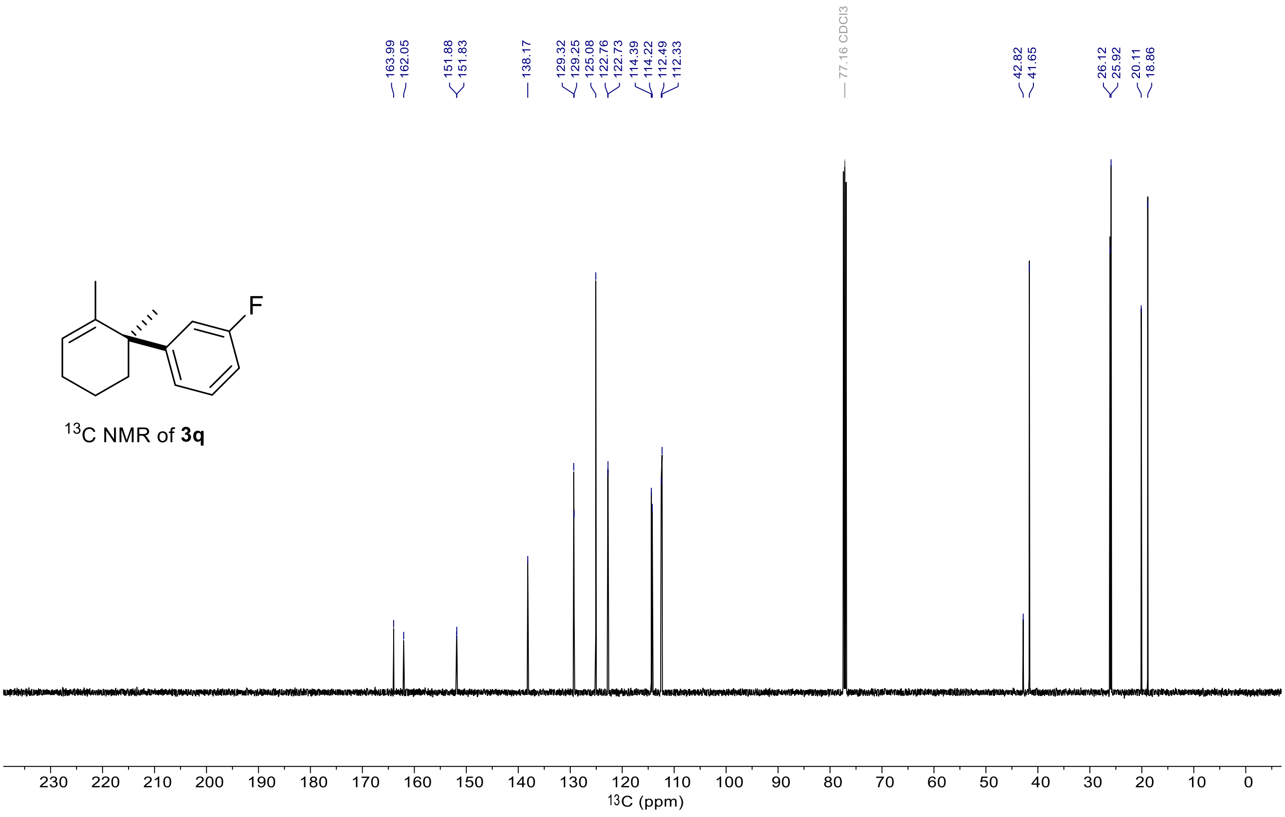

^{13}C NMR of **3q**

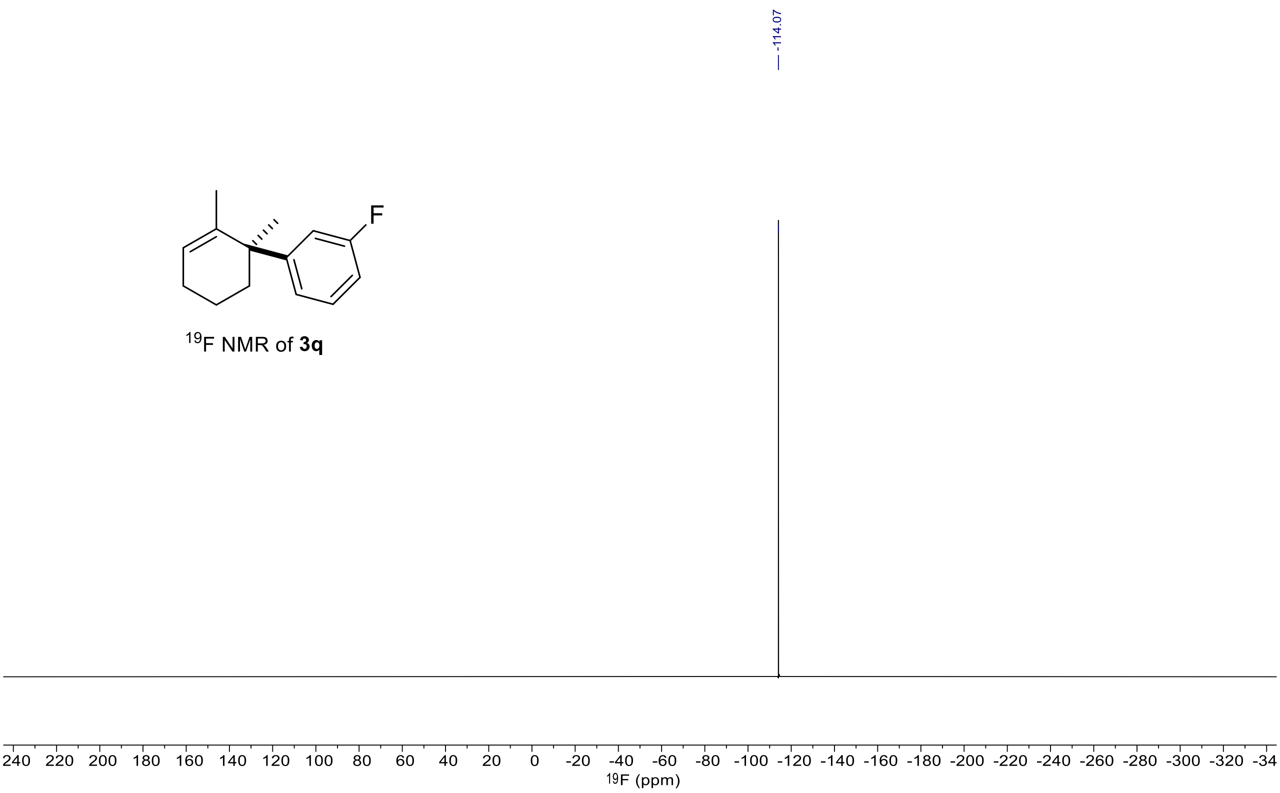

19F NMR of **3q**

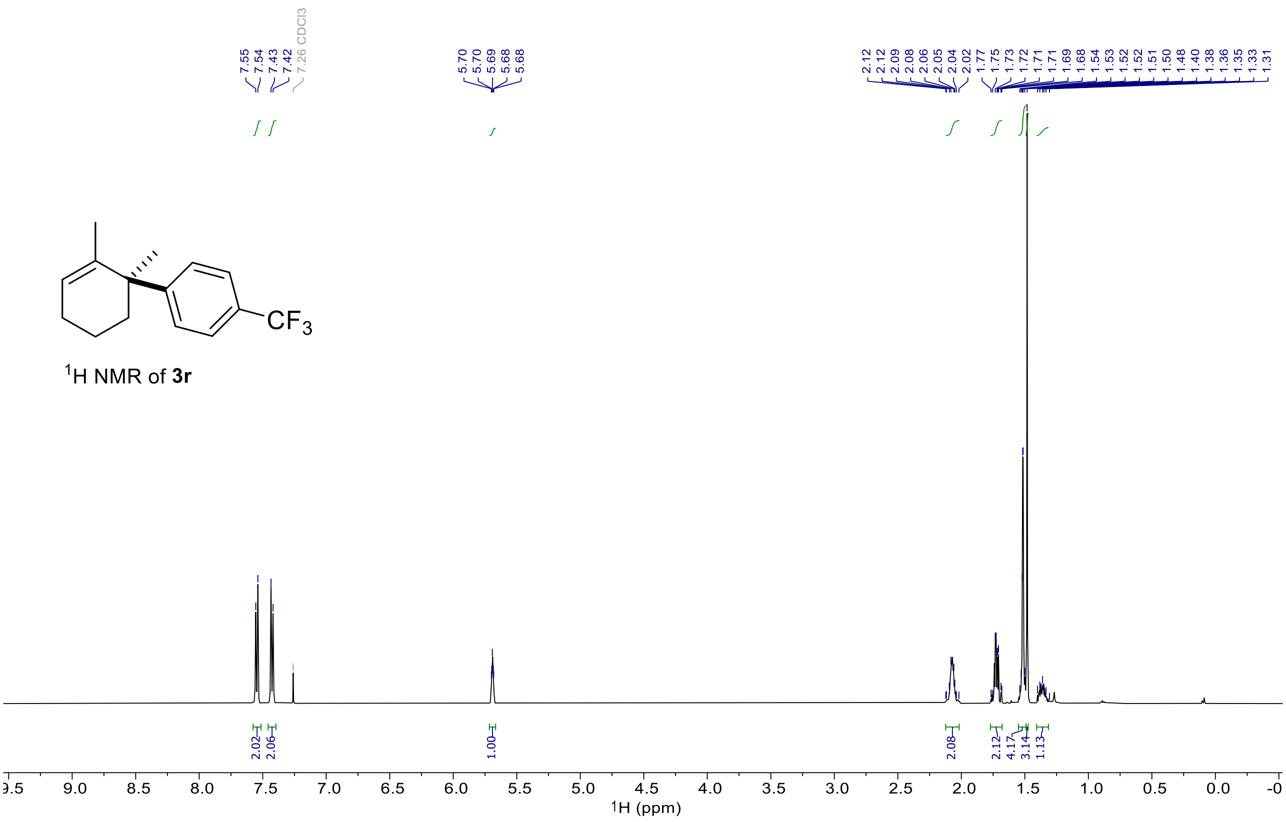

1H NMR of **3r**

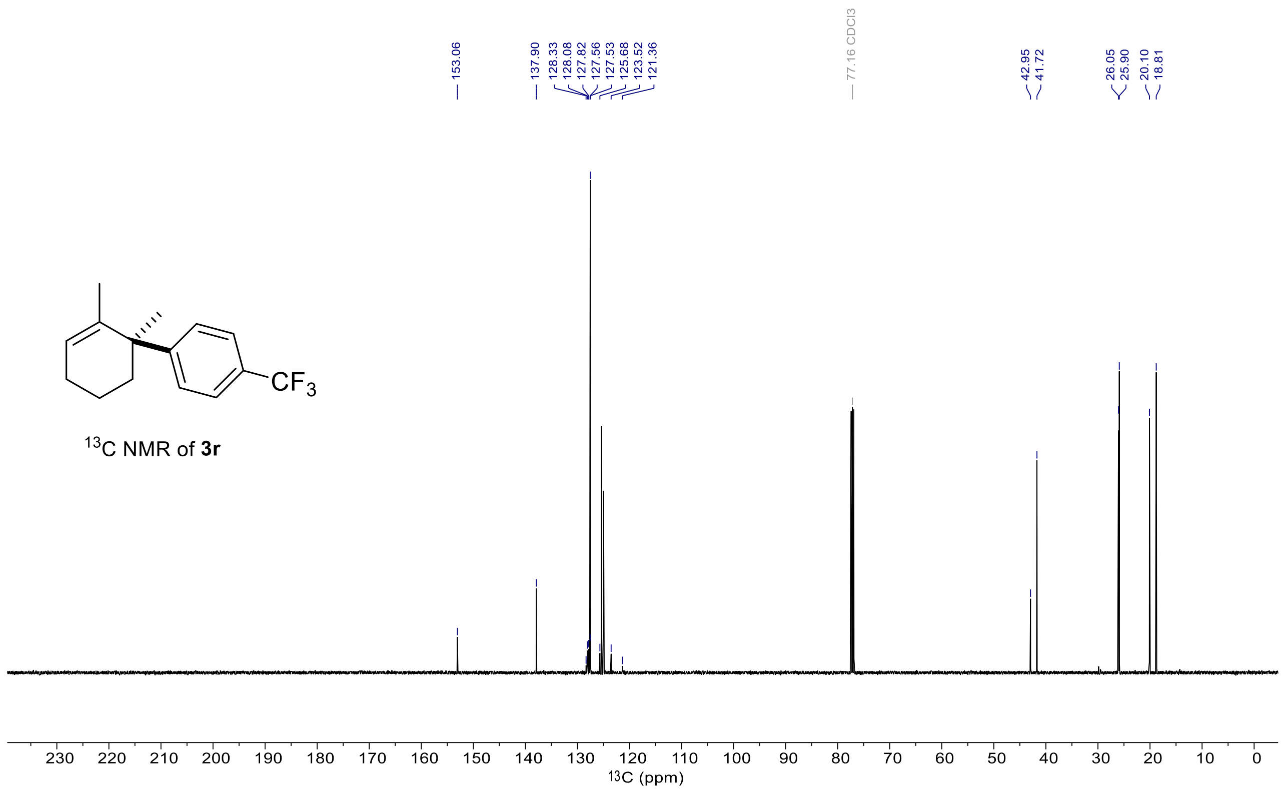
153.06
137.90
128.33
128.08
127.82
127.56
127.53
125.68
123.52
121.36
77.16 CDCl3
42.95
41.72
26.05
25.90
20.10
18.81
CF3
13C NMR of 3r
230 220 210 200 190 180 170 160 150 140 130 120 110 100 90 80 70 60 50 40 30 20 10 0
13C (ppm)

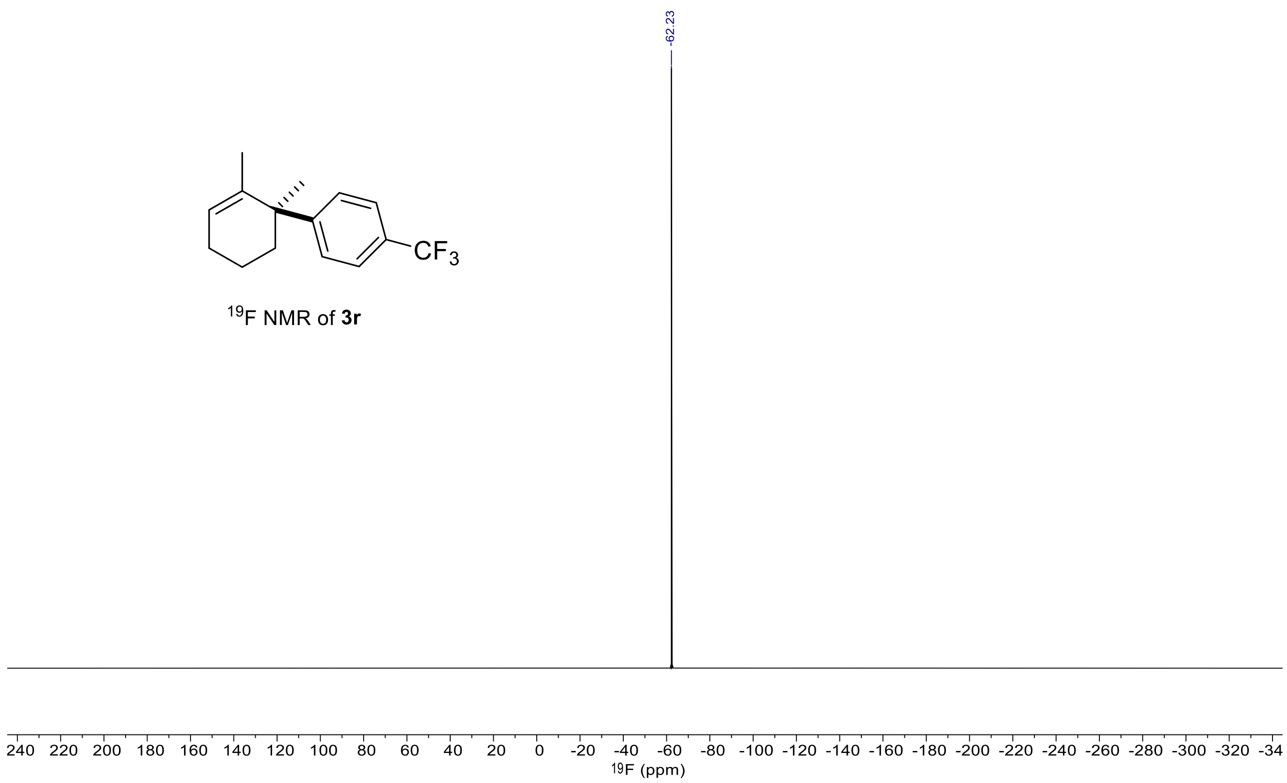
-62.23
CF3
19F NMR of 3r
240 220 200 180 160 140 120 100 80 60 40 20 0 -20 -40 -60 -80 -100 -120 -140 -160 -180 -200 -220 -240 -260 -280 -300 -320 -34
19F (ppm)

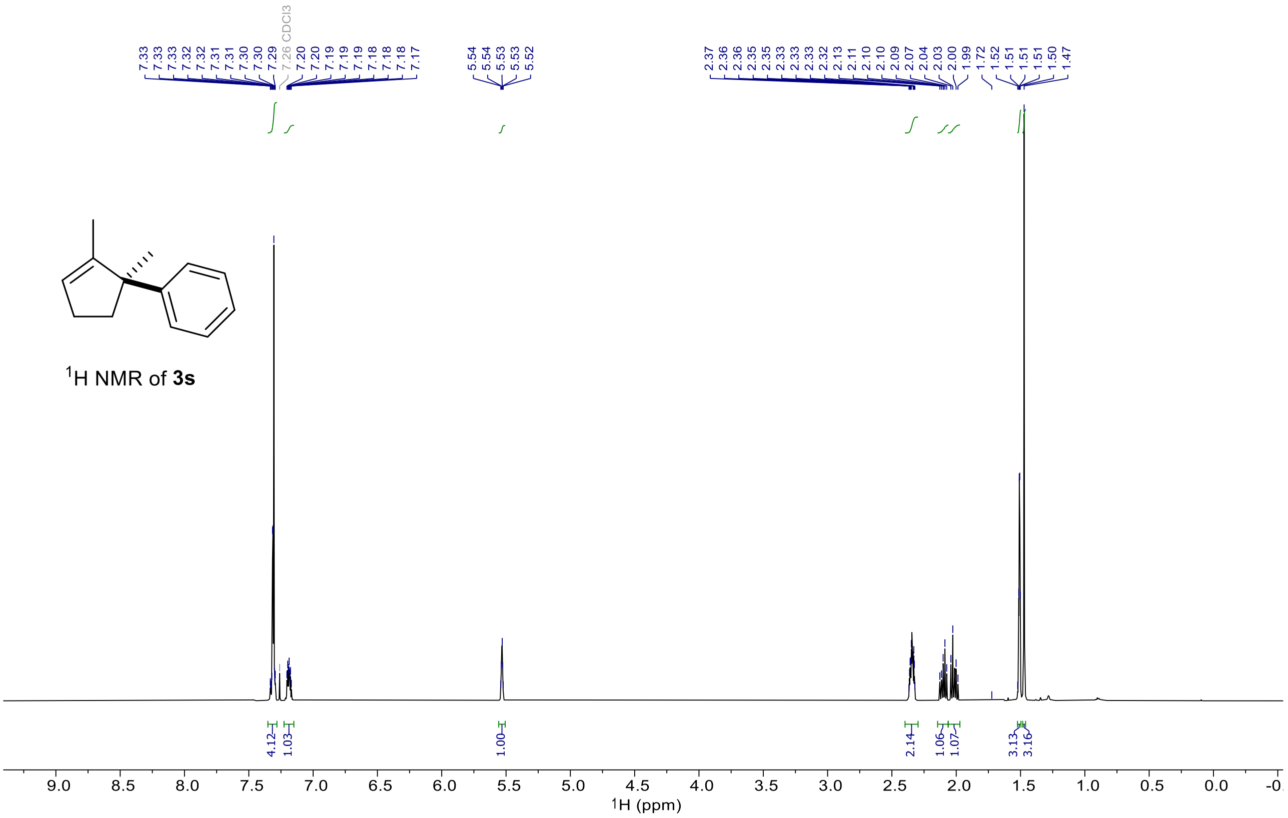

1H NMR of 3s
1H (ppm)

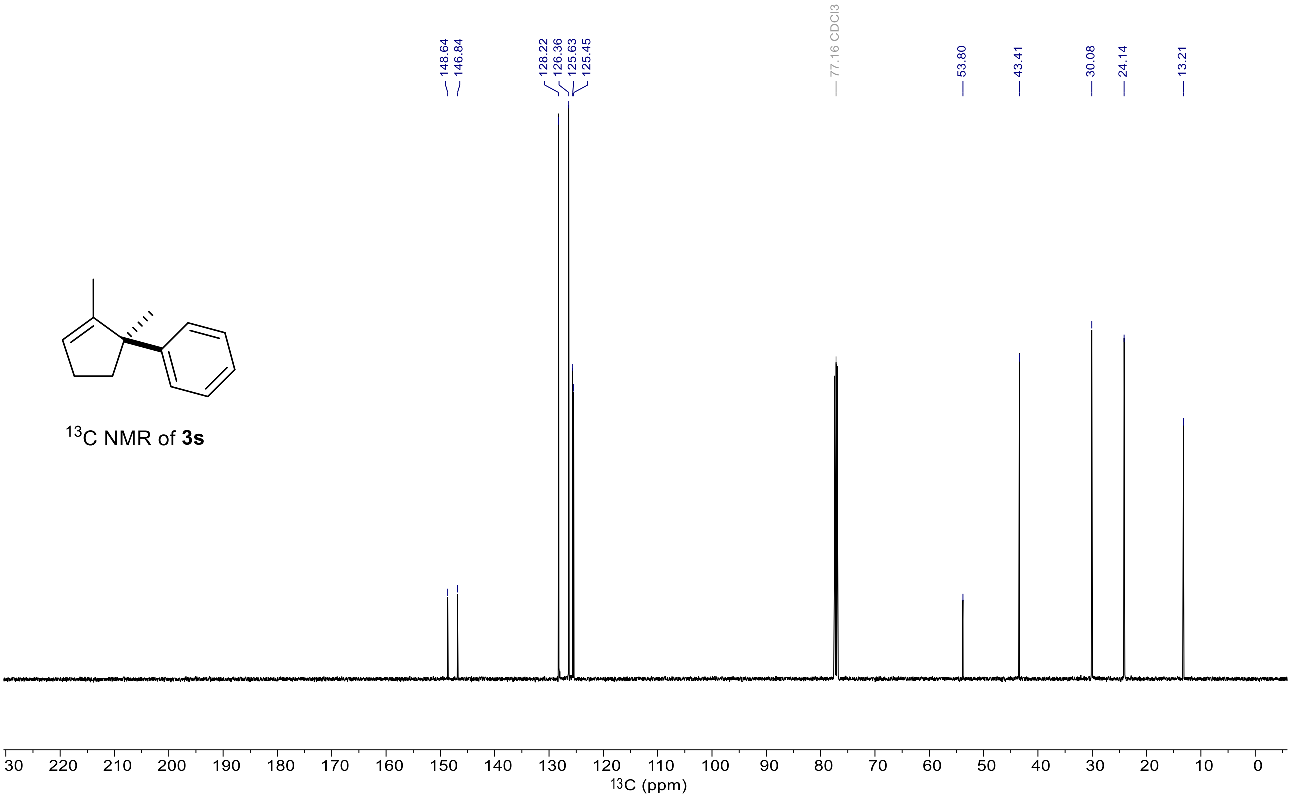

13C NMR of 3s
13C (ppm)

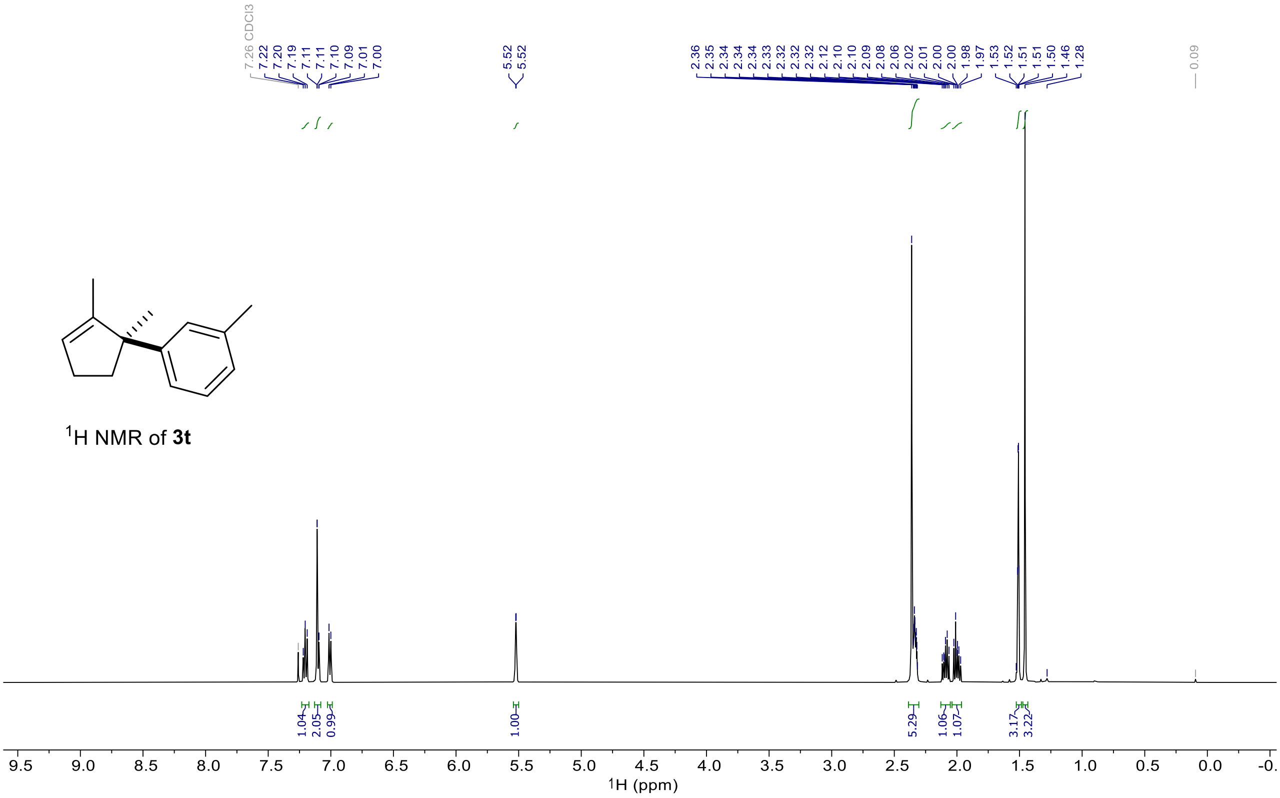
7.26 CDCl3
7.22 7.20 7.19 7.11 7.11 7.10 7.09 7.01 7.00
5.52 5.52
2.36 2.35 2.34 2.34 2.33 2.32 2.32 2.32 2.32 2.12 2.10 2.10 2.09 2.08 2.06 2.02 2.01 2.00 2.00 1.98 1.97 1.53 1.52 1.51 1.51 1.50 1.46 1.28
0.09
1H NMR of 3t
1.04 2.05 0.99 1.00 5.29 1.06 1.07 3.17 3.22
1H (ppm)

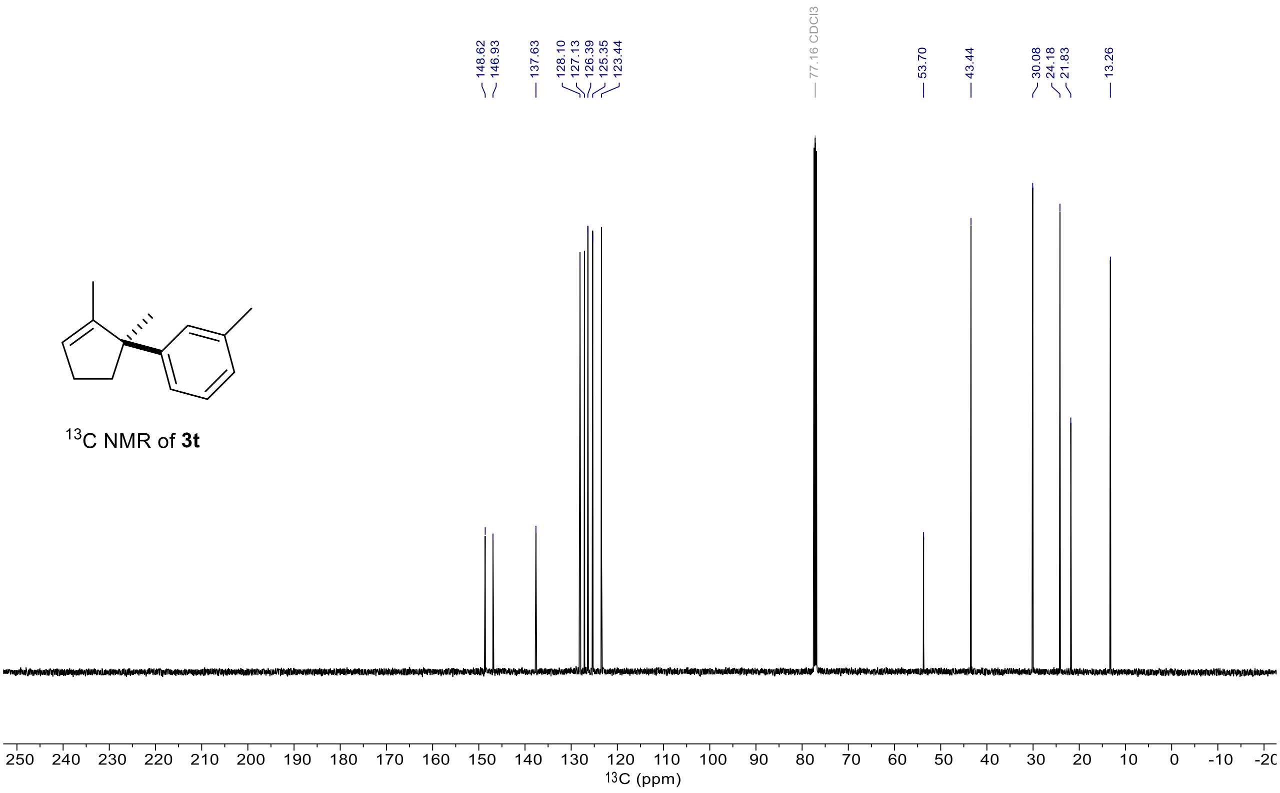
148.62 146.93 137.63 128.10 127.13 126.39 125.35 123.44
77.16 CDCl3
53.70 43.44 30.08 24.18 21.83 13.26
13C NMR of 3t
13C (ppm)

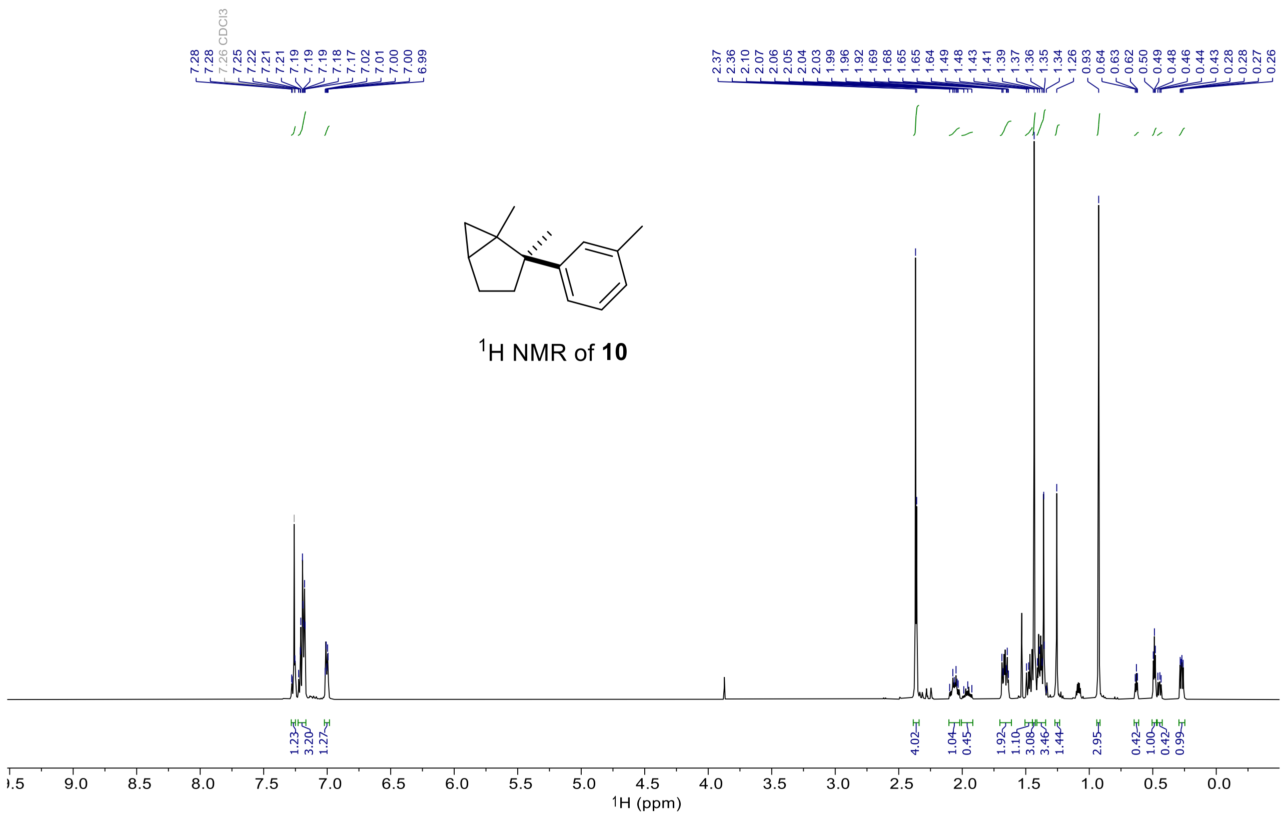

^{1}H NMR of **10**

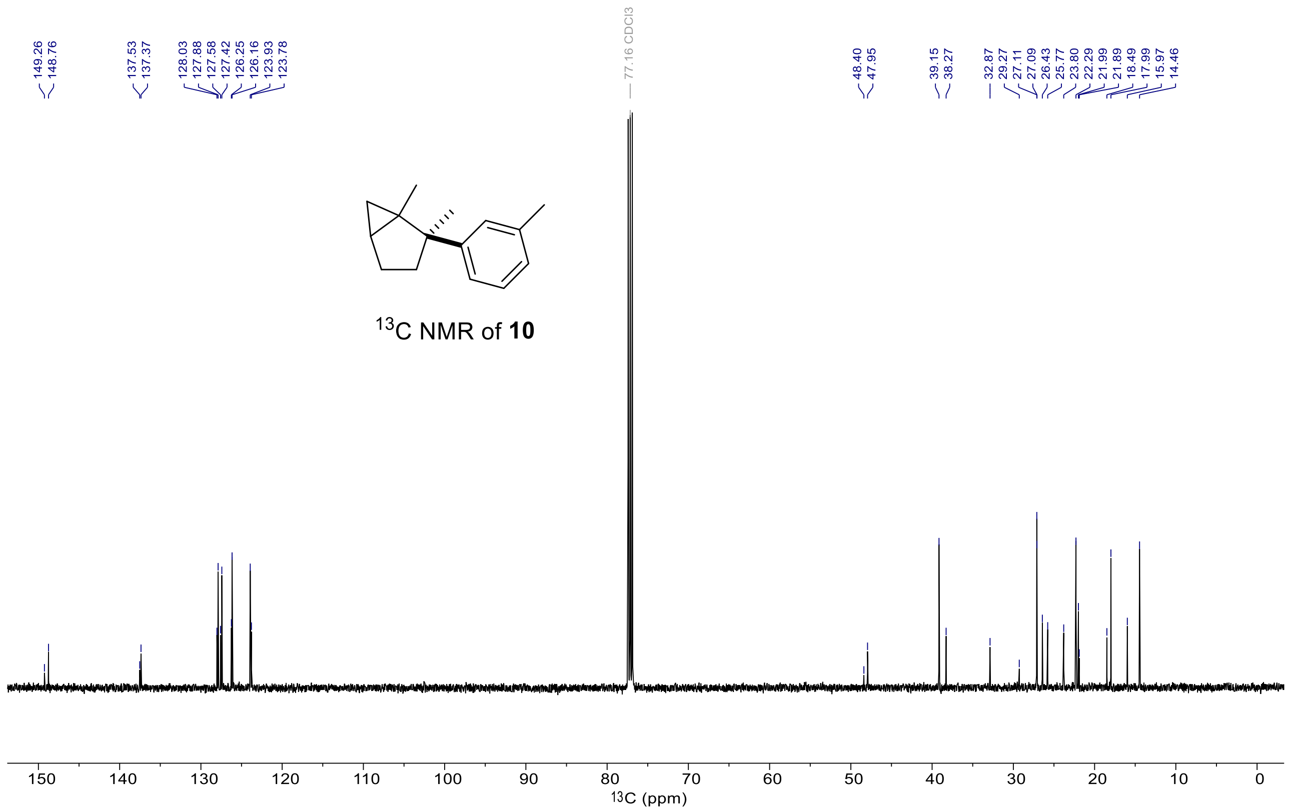

^{13}C NMR of **10**

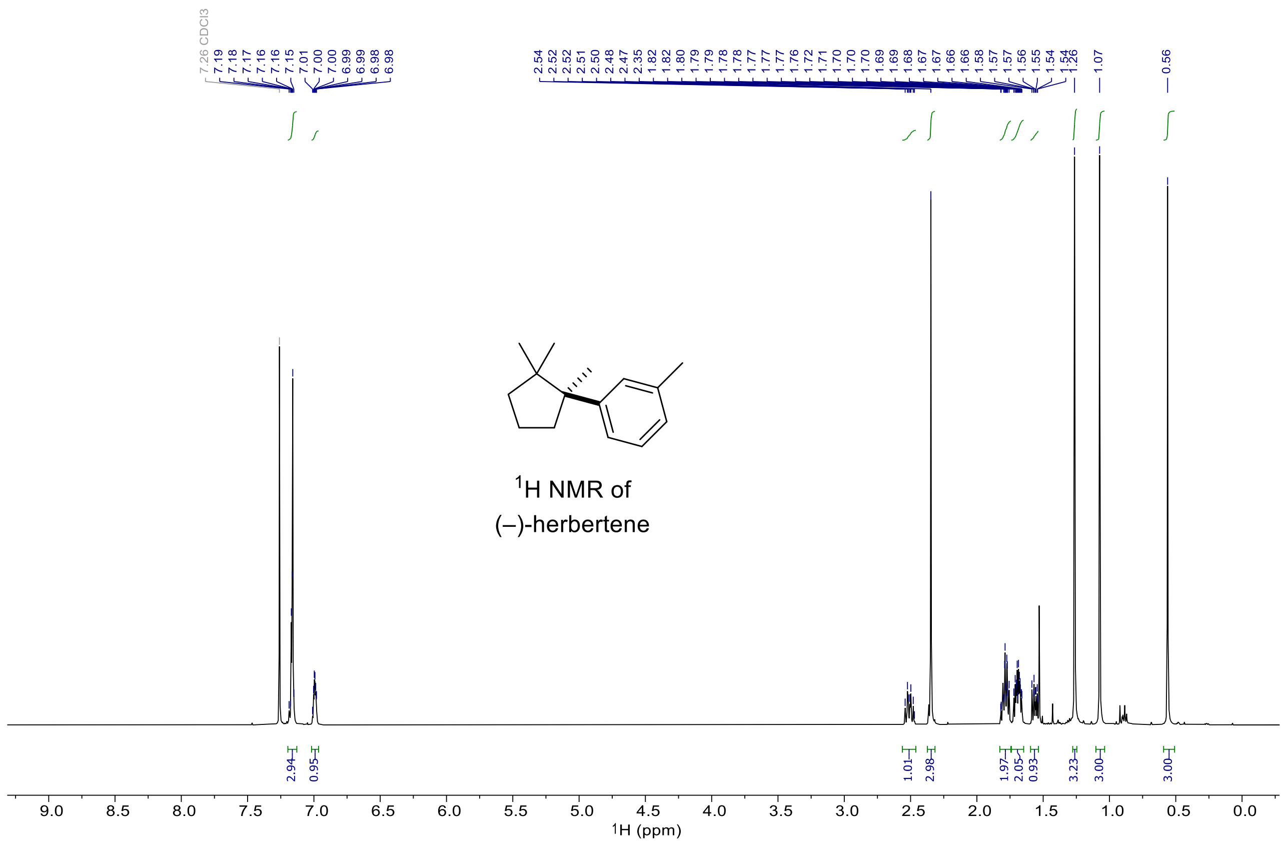
7.26 CDCl3
7.19 7.18 7.17 7.16 7.15 7.01 7.00 7.00 6.99 6.99 6.98 6.98
2.54 2.52 2.52 2.51 2.50 2.48 2.47 2.35 1.82 1.82 1.80 1.79 1.79 1.78 1.78 1.77 1.77 1.76 1.72 1.71 1.70 1.70 1.69 1.69 1.68 1.67 1.67 1.66 1.66 1.58 1.57 1.57 1.56 1.55 1.54 1.54 1.26 1.07 0.56
¹H NMR of
(–)-herbertene
2.94 0.95 1.01 2.98 1.97 2.05 0.93 3.23 3.00 3.00
1H (ppm)

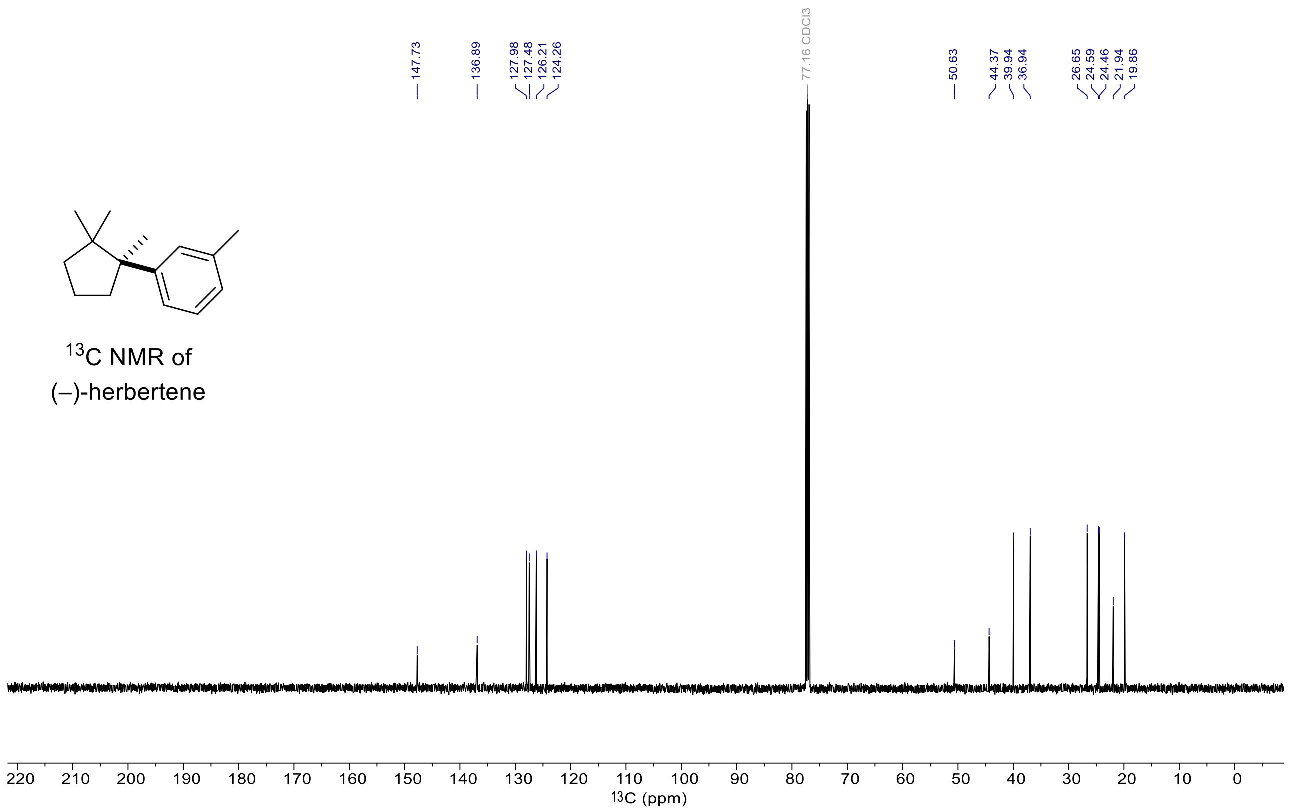
147.73 136.89 127.98 127.48 126.21 124.26
77.16 CDCl3
50.63 44.37 39.94 36.94 26.65 24.59 24.46 21.94 19.86
¹³C NMR of
(–)-herbertene
13C (ppm)

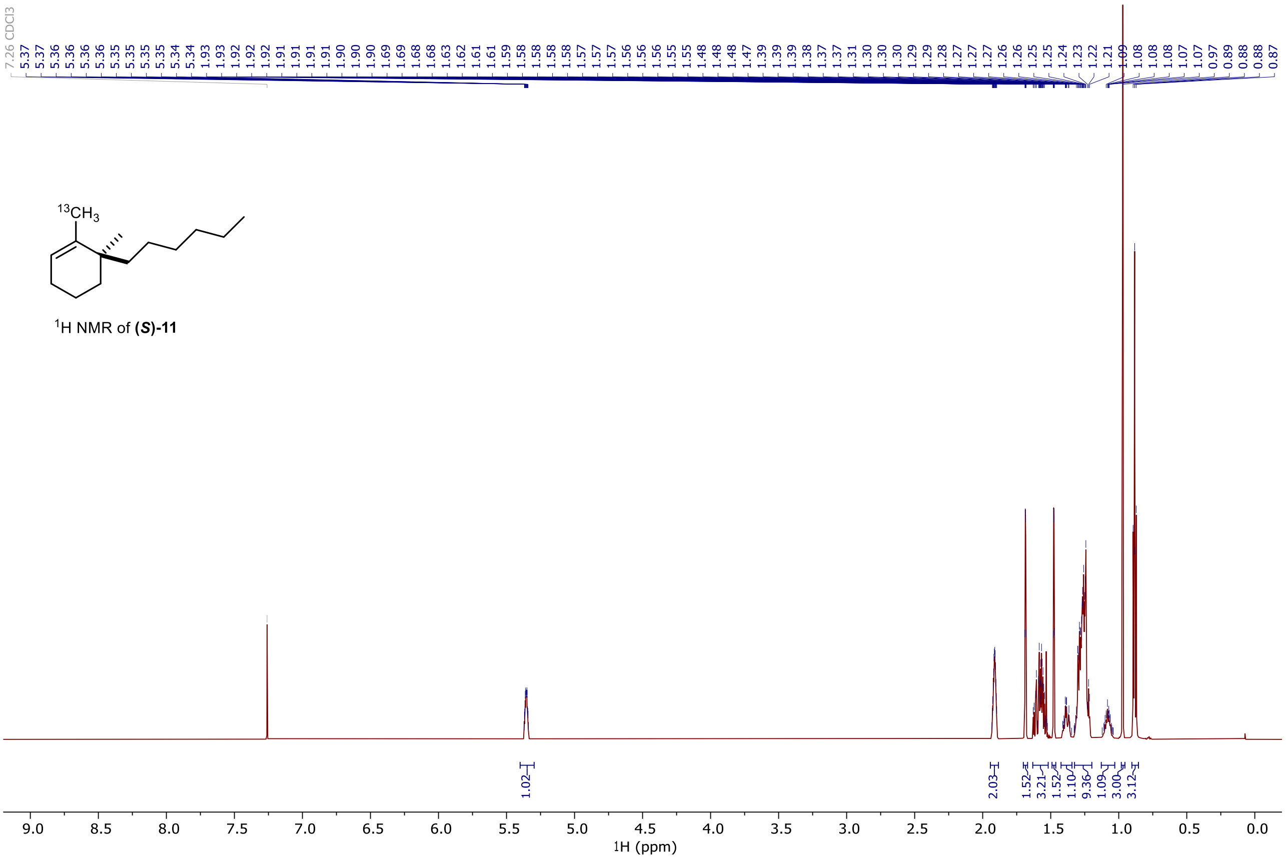
7.26 CDCl3
5.37 5.37 5.36 5.36 5.36 5.36 5.35 5.35 5.35 5.35 5.34 5.34 1.93 1.93 1.92 1.92 1.92 1.91 1.91 1.91 1.91 1.90 1.90 1.90 1.90 1.69 1.69 1.68 1.68 1.63 1.62 1.61 1.61 1.59 1.58 1.58 1.58 1.58 1.58 1.57 1.57 1.57 1.56 1.56 1.56 1.55 1.55 1.48 1.48 1.48 1.47 1.39 1.39 1.39 1.38 1.37 1.37 1.31 1.30 1.30 1.30 1.29 1.29 1.28 1.27 1.27 1.27 1.26 1.26 1.25 1.25 1.24 1.23 1.22 1.21 1.09 1.08 1.08 1.08 1.08 1.07 1.07 0.97 0.89 0.88 0.88 0.87
$^{13}CH_3$
^{1}H NMR of **(*S*)-11**
1.02
2.03
1.52
3.21
1.52
1.10
9.36
1.09
3.00
3.12
9.0 8.5 8.0 7.5 7.0 6.5 6.0 5.5 5.0 4.5 4.0 3.5 3.0 2.5 2.0 1.5 1.0 0.5 0.0
1H (ppm)

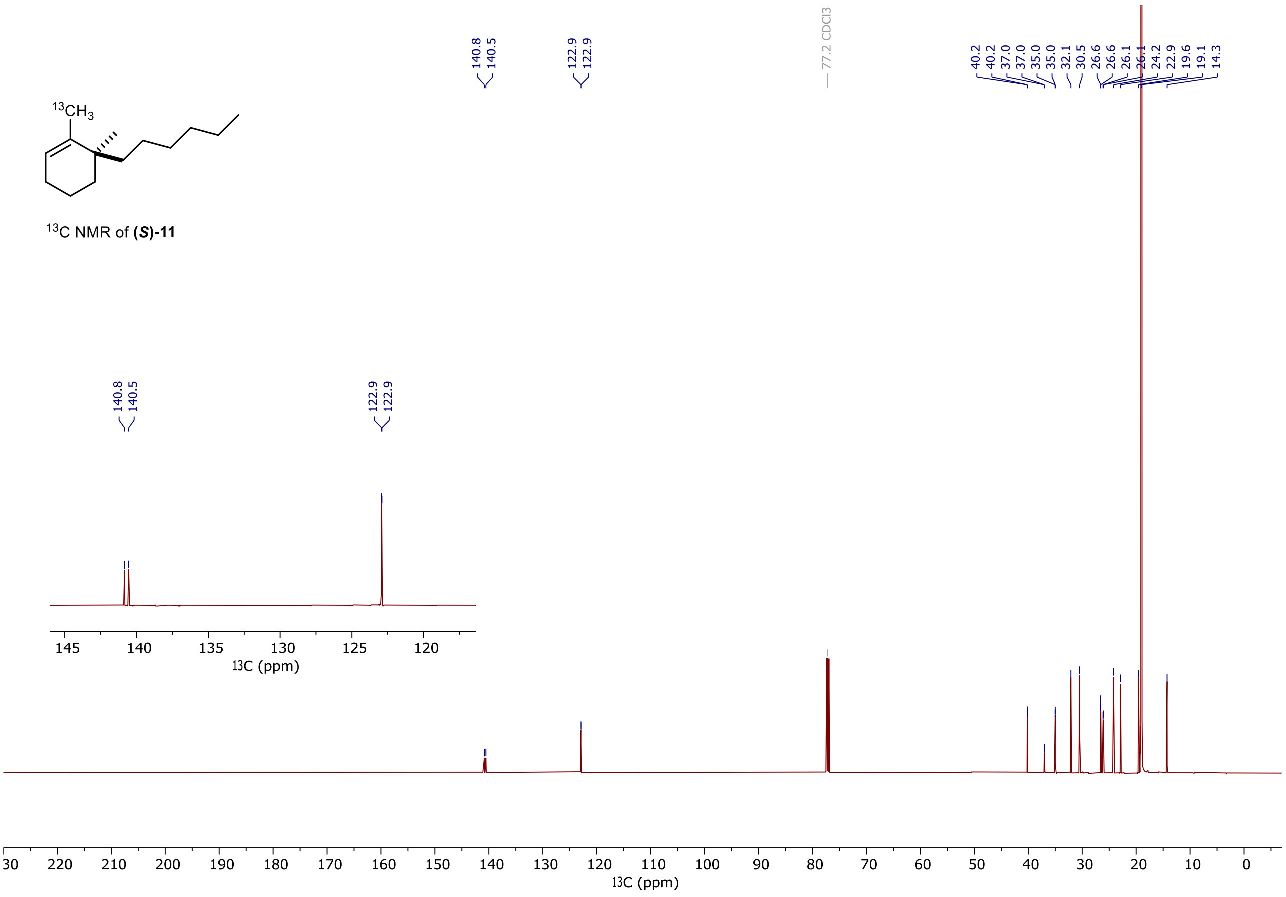
140.8 140.5
122.9 122.9
77.2 CDCl3
40.2 40.2 37.0 37.0 35.0 35.0 32.1 30.5 26.6 26.6 26.1 26.1 24.2 22.9 19.6 19.1 14.3
$^{13}CH_3$
^{13}C NMR of **(*S*)-11**
145 140 135 130 125 120
13C (ppm)
30 220 210 200 190 180 170 160 150 140 130 120 110 100 90 80 70 60 50 40 30 20 10 0
13C (ppm)

User Report (WEV-WF-002-25)

NMR data supports the following structure as major component:

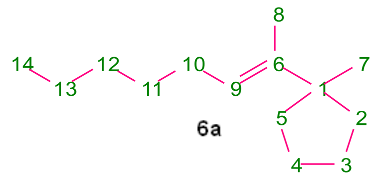

6a

Atom	δ (ppm)	Predicted Shift	COSY	HSQC	HMBC	NOESY
1 C	48.780	45.24			7, 8, 9	
2 C	37.781	38.98		2', 2''	5', 7	
H'	1.611		2''	2	3, 4, 5	9
H''	1.397		2'	2		9
3 C	23.760	25.20		3	2', 5'	
H2	1.635			3		9
4 C	23.760	25.20		4	2', 5'	
H2	1.635			4		9
5 C	37.781	38.98		5', 5''	2', 7	
H'	1.611			5	2, 3, 4	9
H''	1.397			5		9
6 C	142.291	138.84			7, 8, 10	
7 C	26.227	23.41		7		
H3	1.006			7	1, 2, 5, 6	8, 9
8 C	13.827	16.56		8	9	
H3	1.611		9	8	1, 6, 9	7, 10
9 C	121.710	123.05		9	8, 10, 11	
H	5.157		8, 10	9	1, 8, 10, 11	2', 2'', 3, 4, 5', 5'', 7, 10, 11, 12
10 C	28.158	28.72		10	9, 11, 12, 13	
H2	1.972		9, 11	10	6, 9, 11, 12	8, 9
11 C	29.756	28.42		11	9, 10, 12, 13	
H2	1.335		10	11	9, 10, 12, 13	9
12 C	31.792	31.24		12	10, 11, 13, 14	
H2	1.270			12	10, 11, 13, 14	9
13 C	22.794	22.40		13	11, 12, 14	
H2	1.298		14	13	10, 11, 12	
14 C	14.275	14.06		14	12	
H3	0.887		13	14	12, 13	

Minor component (3%)

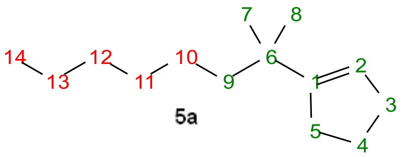

5a

Atom	δ (ppm)	Predicted Shift	COSY	HSQC	HMBC	NOESY
1 C	152.707	150.62			5, 7, 8, 9	
2 C	122.023	121.24		2	3	
H	5.295		3, 5	2		3, 7, 8
3 C	32.492	30.50		3		
H2	2.275		2, 4	3	2	2
4 C	23.981	24.71		4		
H2	1.817		3, 5	4		
5 C	31.575	31.01		5		
H2	2.204		2, 4	5	1	
6 C	35.964	38.22			7, 8, 9	
7 C	27.466	27.04		7	8, 9	
H3	1.009			7	1, 6, 8, 9	2, 9
8 C	27.466	27.04		8	7, 8, 9	
H3	1.009			8	1, 6, 7, 8	2, 9
9 C	41.552	42.26		9	7	
H2	1.293			9	1, 6, 7, 8	7, 8
10 C		25.16				
H2						
11 C		29.41				
H2						
12 C		31.29				
H2						
13 C		22.13				
H2						
14 C		14.09				
H3						

P-ID: ML00xxx
Measured on: 25/01/2022
CHIFFRE: WEV-WF-002-25
ELNA#: 7577
Client: Vijay Wakchaure
Group: List
Spectroscopist: Leutzsch
Analysed on: 27/01/2022
Analysed by: Leutzsch
Amount: 6.0 mg
Solvent: CDCl3
Reference: 1H+13C on solvent,other nuclei w/ xiref
Temperature: 298 K
Spectrometer: av600neo
Probe: cryoBBO
Experiments: 1H-zg30, 13C-zgpg30, [13C, 1H]-hsqcedetgpsisp2.3, [13C, 1H]-hmbcetgpl3nd, [1H, 1H]-cosygpppqf, [1H, 1H]-noesygpphpp

^{1}H NMR of **6a** and **5a**

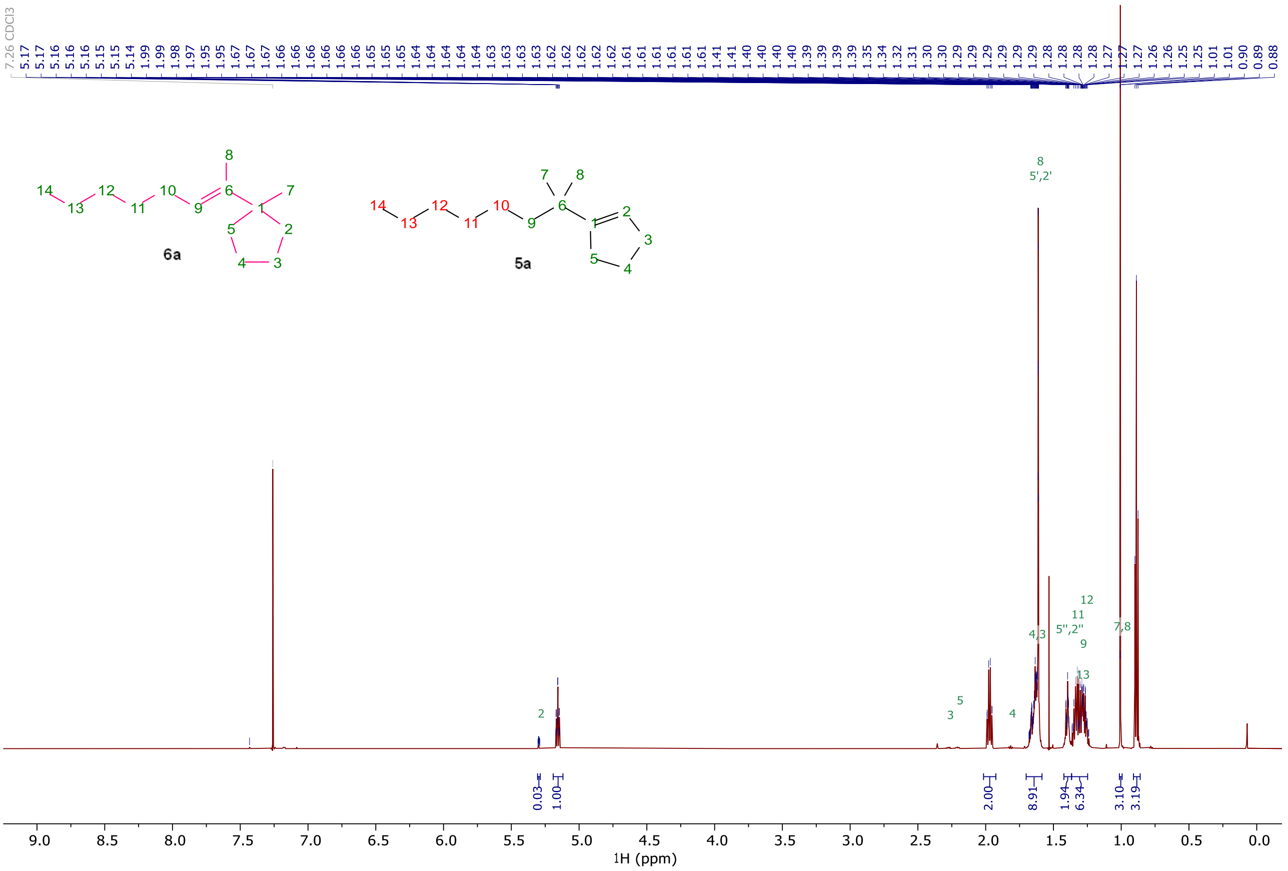

^{13}C NMR of **6a** and **5a**

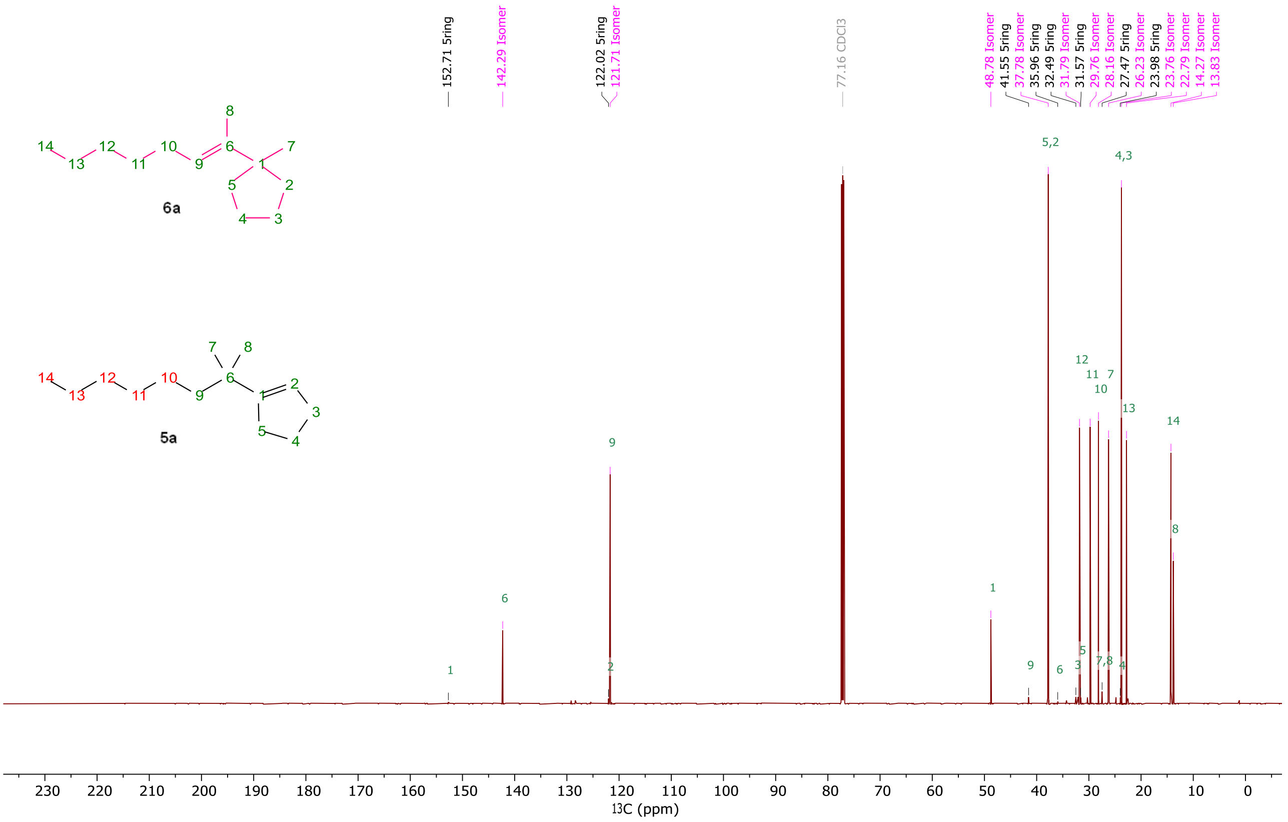

1H{13C},HSQC-EDITED, 600.20 MHz,CDCl3,298.0K, pulse sequence: hsqcedetgpsisp2.3

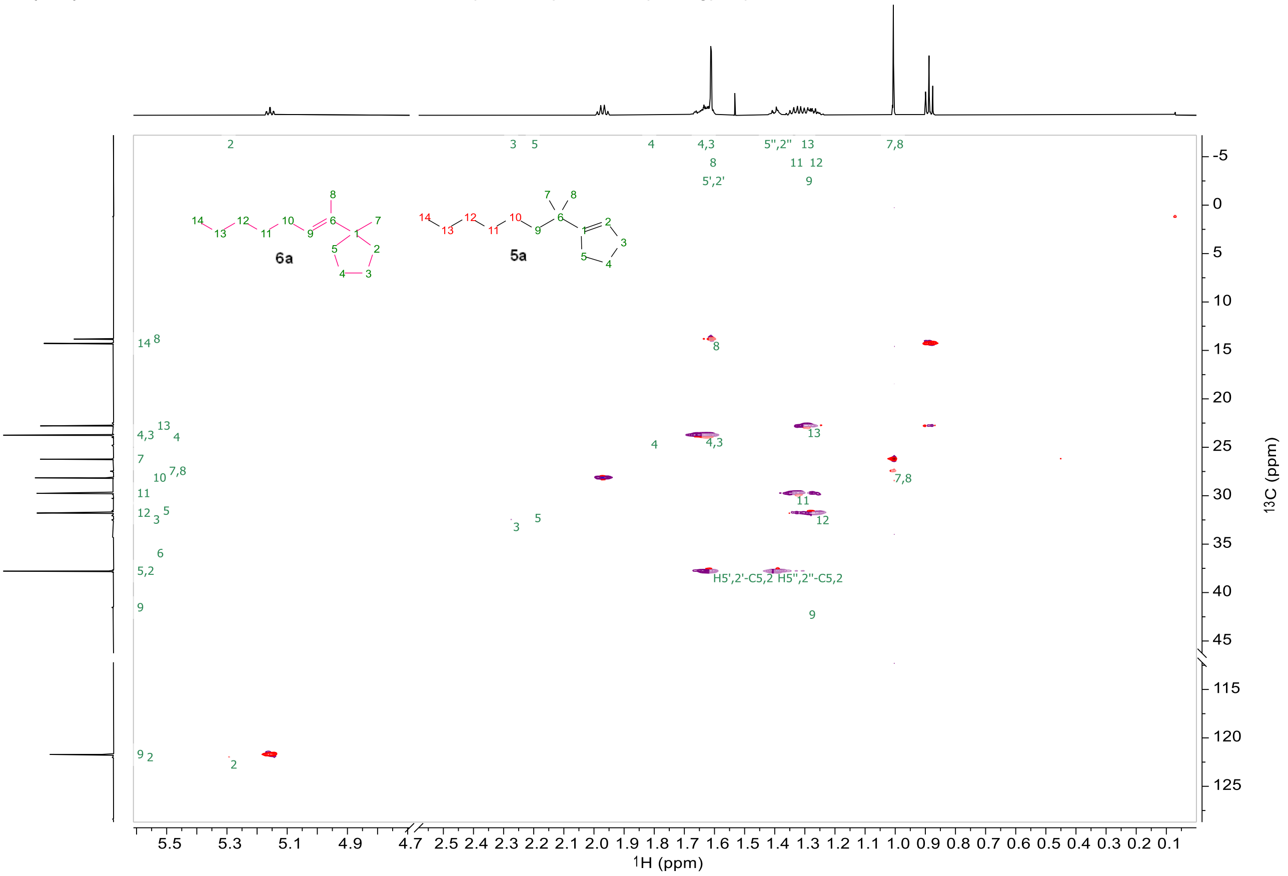

1H{13C},HMBC, 600.20 MHz,CDCl3,298.0K, pulse sequence: hmbcetgpl3nd

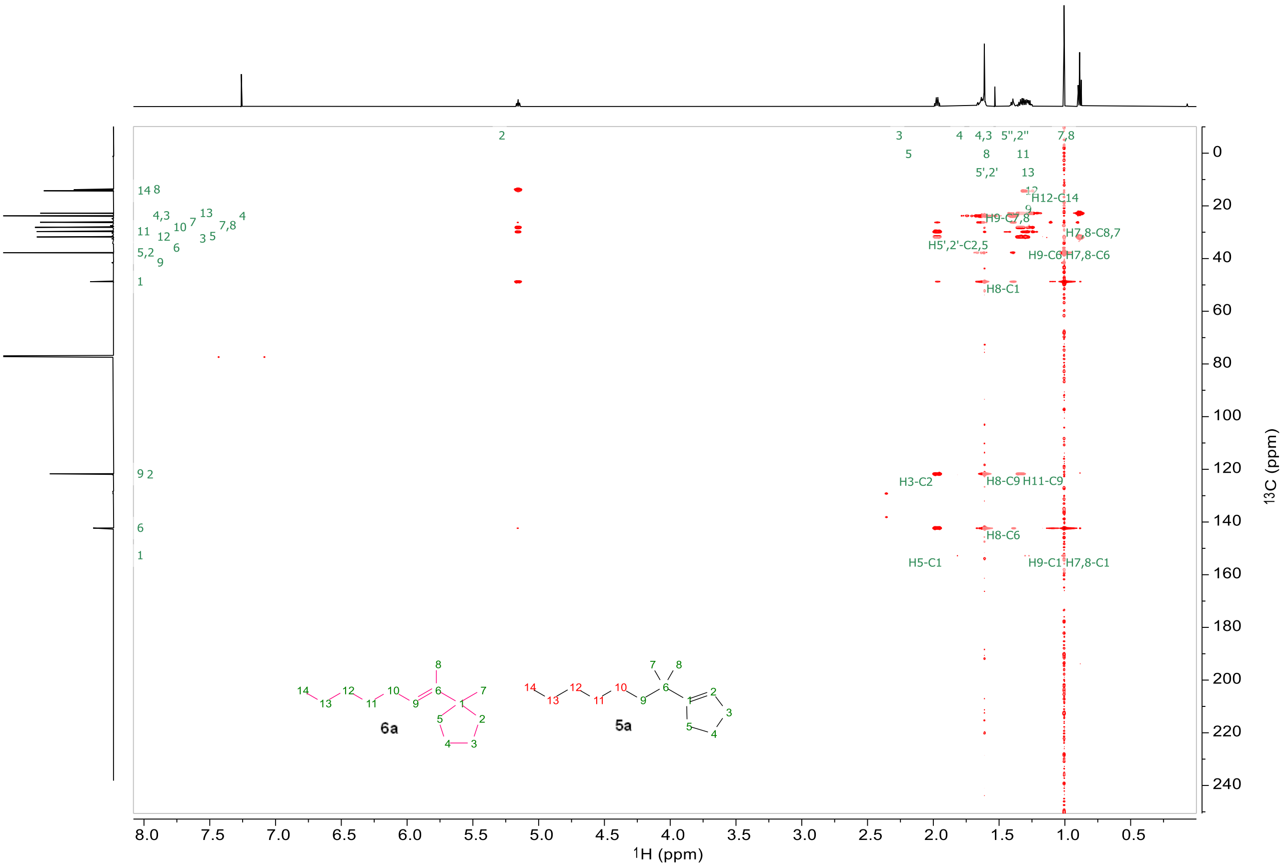

1H{off},COSY, 600.20 MHz,CDCl3,298.0K, pulse sequence: cosygpppqf

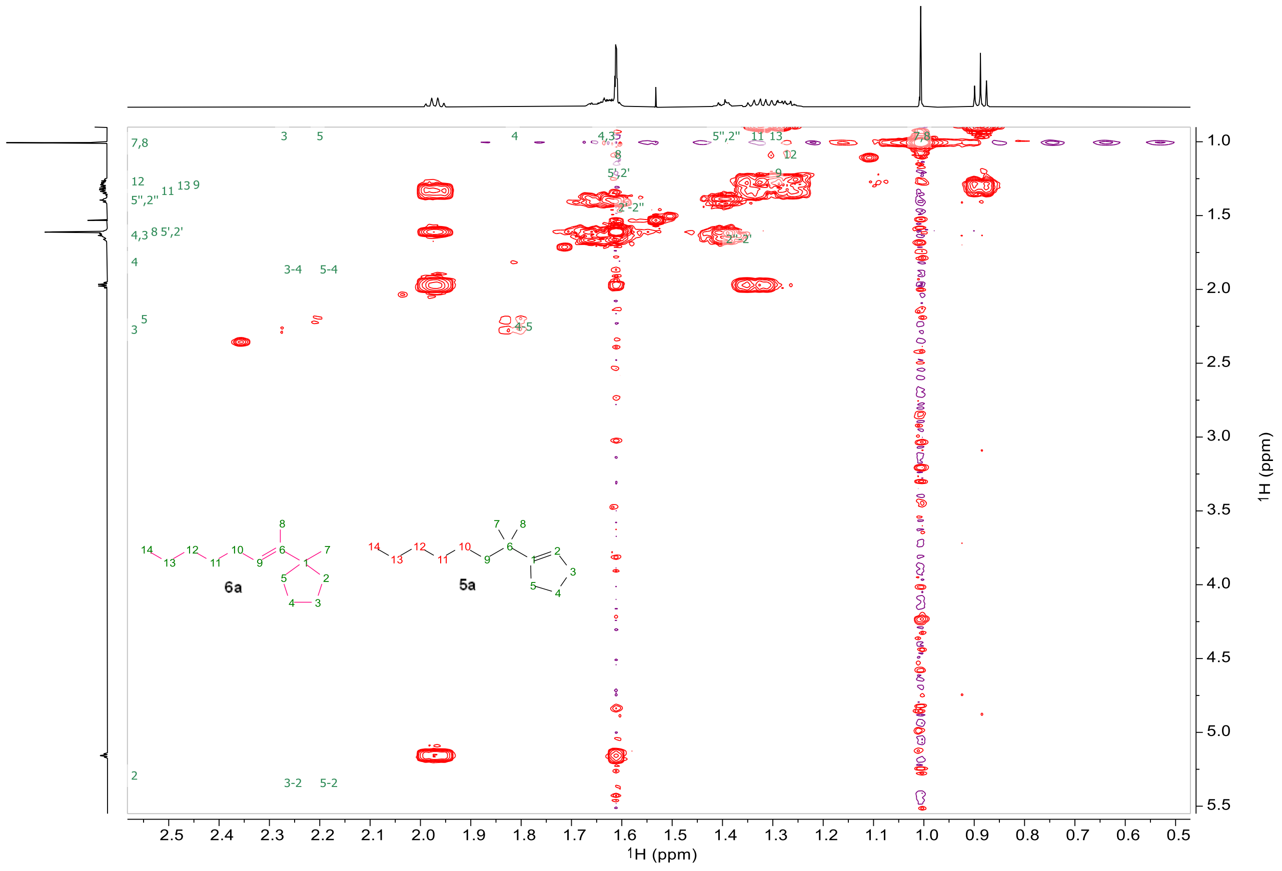

1H{off},NOESY, 600.20 MHz,CDCl3,298.0K, pulse sequence: noesygpphpp

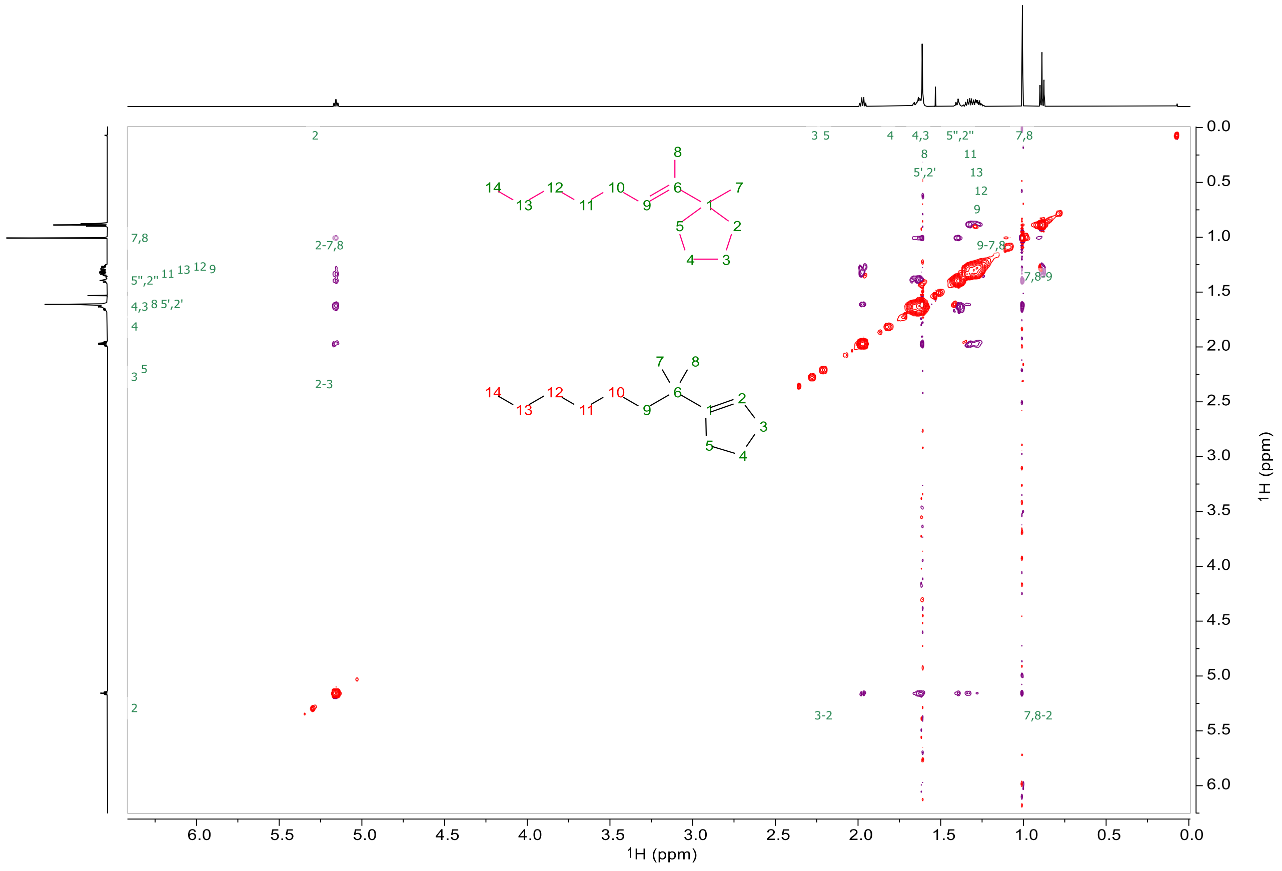

NMR data supports the following structure

User Report
WEV-WF-314-01

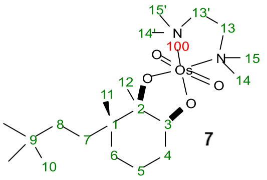

Observed NOEs are in good agreement with the X-ray structure

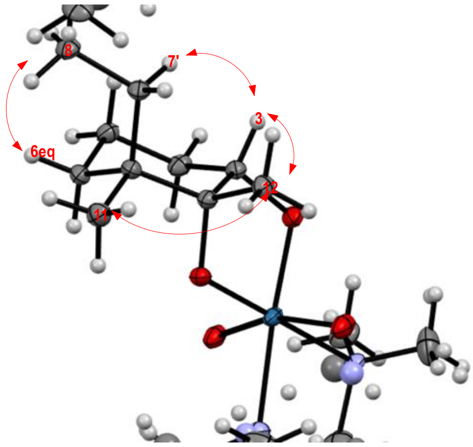

Atom	J	δ (ppm)	HSQC	COSY	HMBC	NOESY
1 C		39.927			7', 11, 12	
2 C		91.838			11, 12	
3 C		89.277	3		4ax, 12	
H	10.2(4ax), 7.0(4eq)	3.761	3	4ax, 4eq	12	4eq, 5'', 7', 12
4 C		27.331	4ax, 4eq			
Hax	10.2(3)	1.973	4	3, 4eq, 5', 5'', 6eq	3, 5	4eq, 5', 6ax
Heq	7.0(3)	1.615	4	3, 4ax, 5', 5''		3, 4ax
5 C		19.139	5', 5''		4ax, 6ax	
H'		1.488	5	4ax, 4eq, 5''		4ax, 5''
H''		1.380	5	4ax, 4eq, 5'		3, 5'
6 C		30.794	6ax, 6eq		11	
Hax		1.385	6	6eq	5	4ax, 6eq, 11
Heq		1.205	6	4ax, 6ax		6ax, 8, 11
7 C		32.167	7', 7''		11	
H'		1.493	7	7'', 8	1, 8, 11	3, 10
H''		1.358	7	7', 8	11	10
8 C		37.576	8		7'	
H2		1.126	8	7', 7''	9, 10	6eq, 10, 11
9 C		30.282			8, 10	

Atom	J	δ (ppm)	HSQC	COSY	HMBC	NOESY
10 C		29.650	10		8, 10	
H3		0.897	10		9, 10	7', 7'', 8
11 C		20.681	11		7', 7''	
H3		0.991	11		1, 2, 6, 7	6ax, 6eq, 8, 12
12 C		18.149	12		3	
H3		1.230	12		1, 2, 3	3, 11
13 C		64.118	13		13', 14, 15	
H2		3.079	13		13', 14, 15	
13' C		64.028	13'		13, 14', 15'	
H2		3.036	13'		13, 14', 15'	
14 C		51.641	14		13, 15	
H3		2.777	14		13, 15	
14' C		50.939	14'		13', 15'	
H3		2.784	14'		13', 15'	
15 C		51.467	15		13, 14	
H3		2.772	15		13, 14	
15' C		50.693	15'		13', 14'	
H3		2.789	15'		13', 14'	
100 N						

P-ID: ML00xxx
Measured on: 17/01/2023
CHIFFRE: WEV-WF-114-01
ELNA#: 9597
Client: Vijay Wakchaure
Group: List
Spectroscopist: Leutzsch
Analysed on: 27/04/2023
Analysed by: Leutzsch
Amount: 8.0 mg
Solvent: CDCl3
Reference: 1H+13C on solvent,other nuclei w/ [illegible]
Temperature: 298 K
Spectrometer: av600neo
Probe: cryoBBO
Experiments: 1H-zg30, 1H-zg30, 13C-zgpg30, 1H-13C-hsqcedetgpsisp2.3, 1H-13C-hmbcetgpl3nd, 1H-1H-cosygpppqf, 1H-1H-no[illegible]

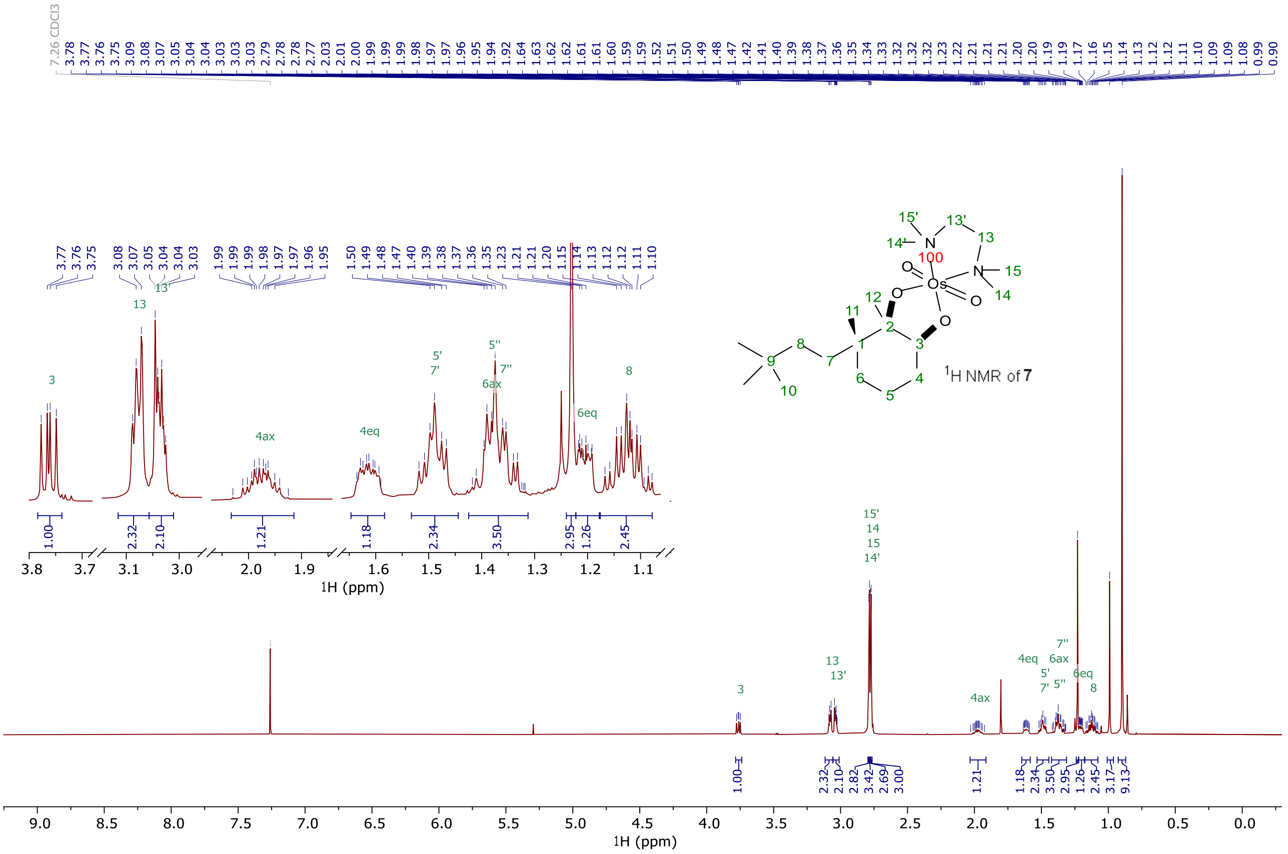

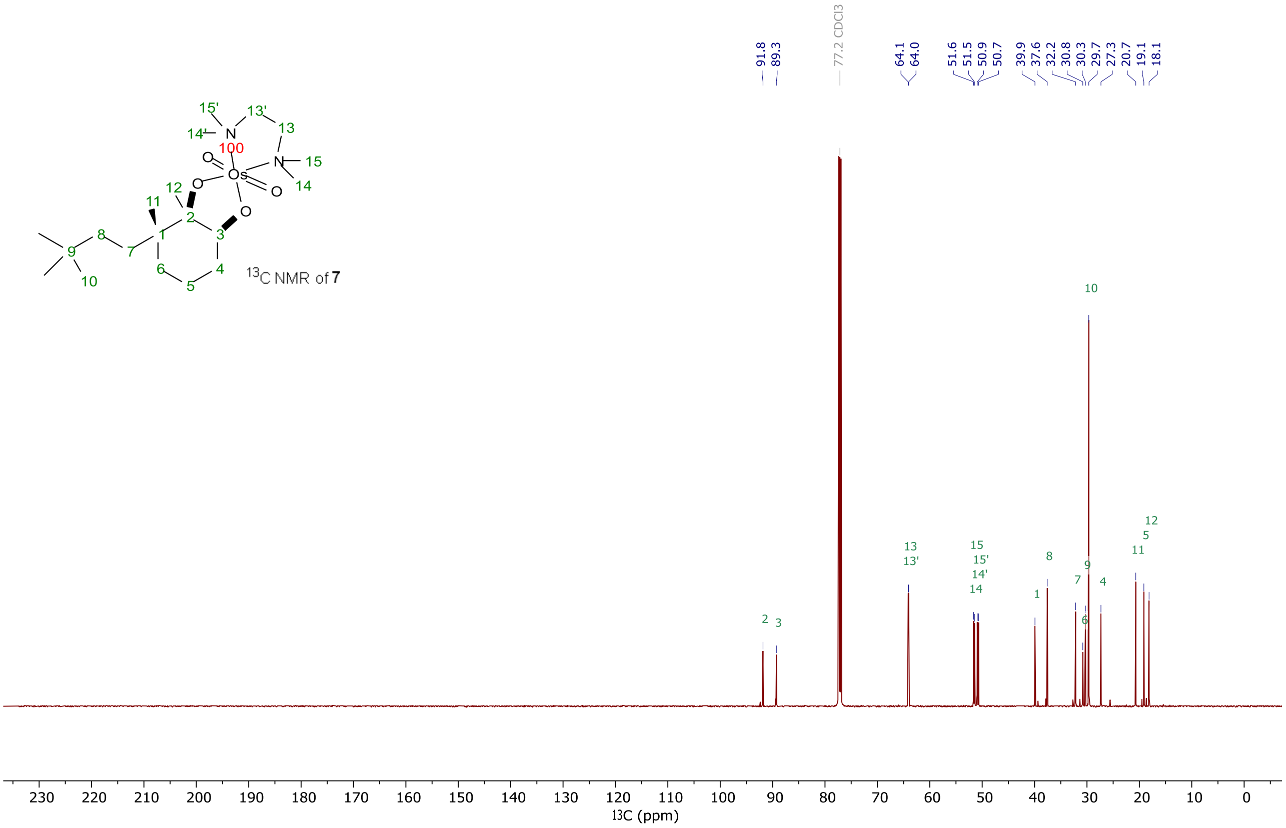

1H{13C},HSQC-EDITED, 600.20 MHz,CDCl3,298.0K, pulse sequence: hsqcedetgpsisp2.3

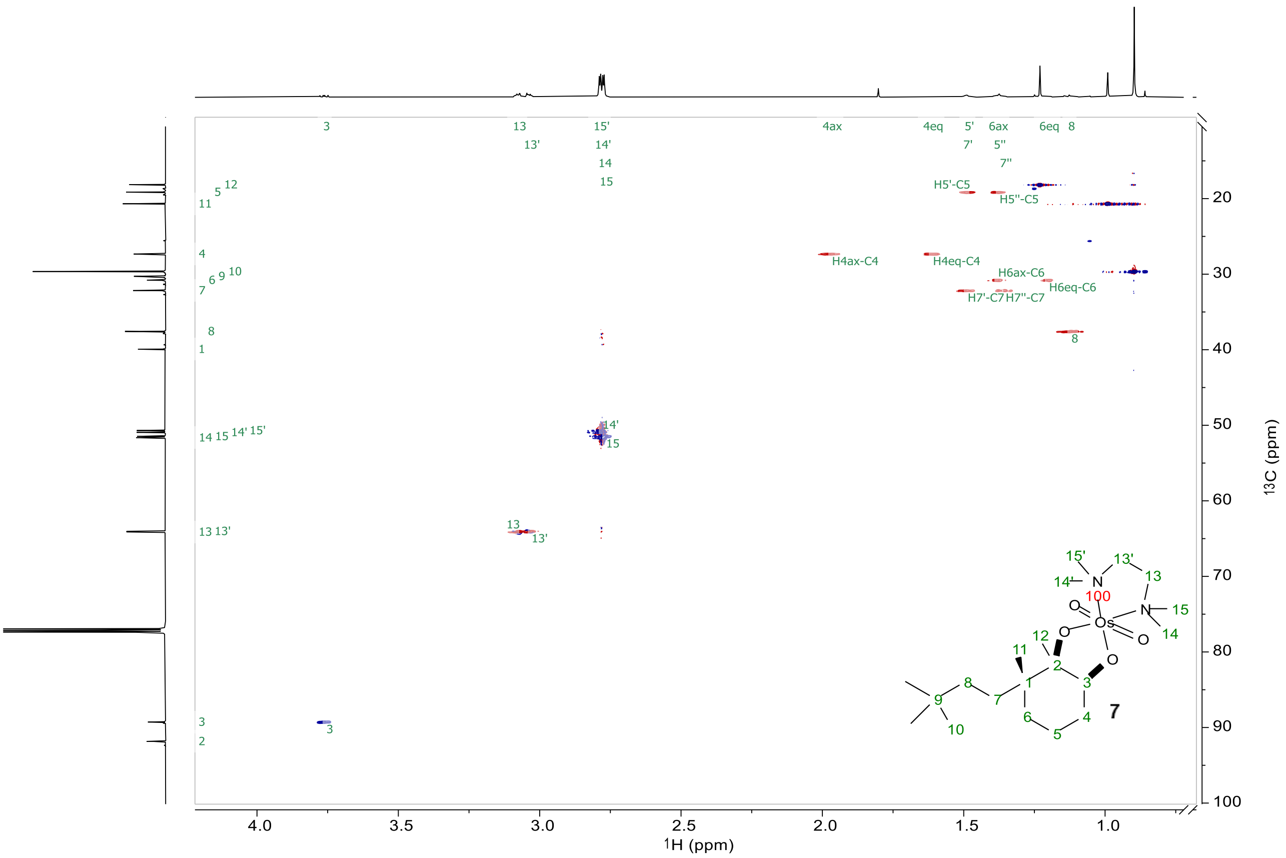

1H{13C},HMBC, 600.20 MHz,CDCl3,298.0K, pulse sequence: hmbcetgpl3nd

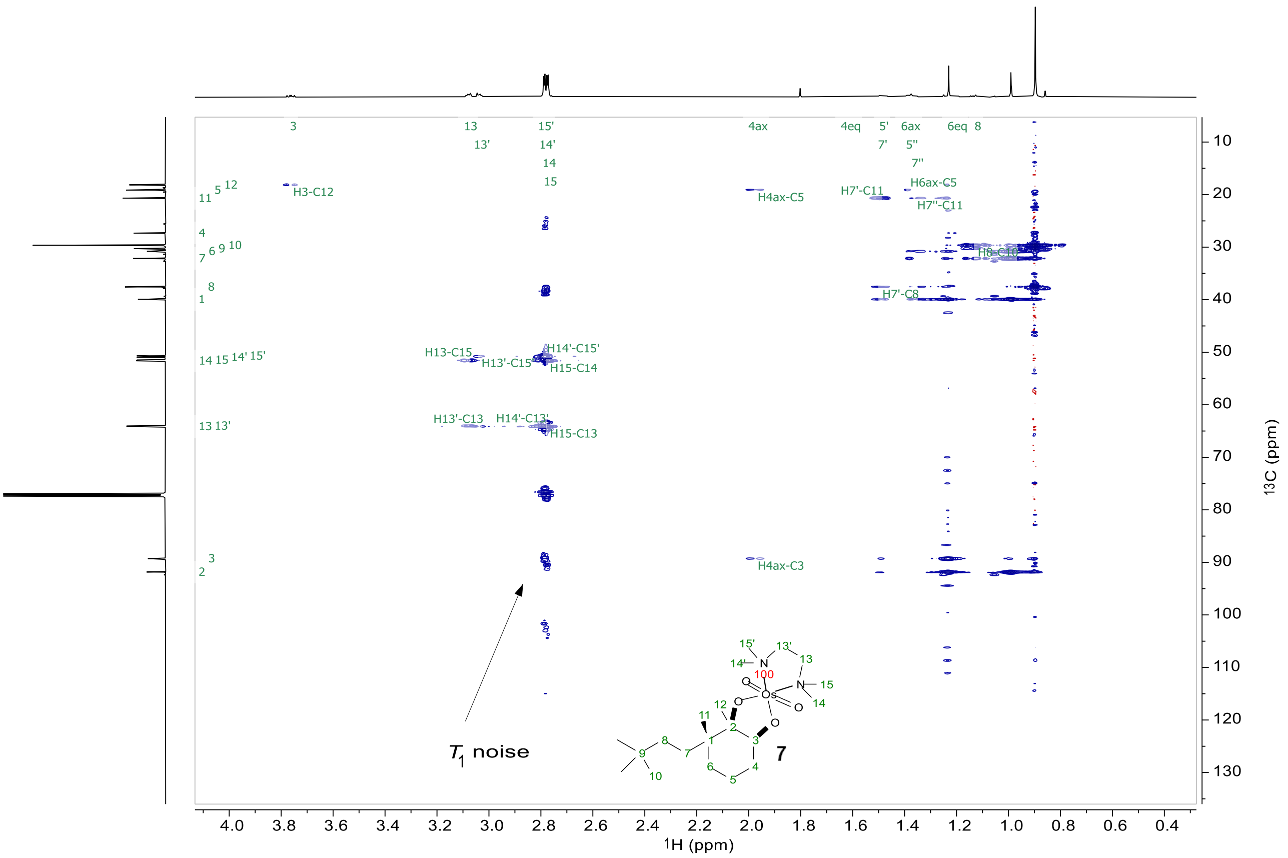

1H{off},COSY, 600.20 MHz,CDCl3,298.0K, pulse sequence: cosygpppqf

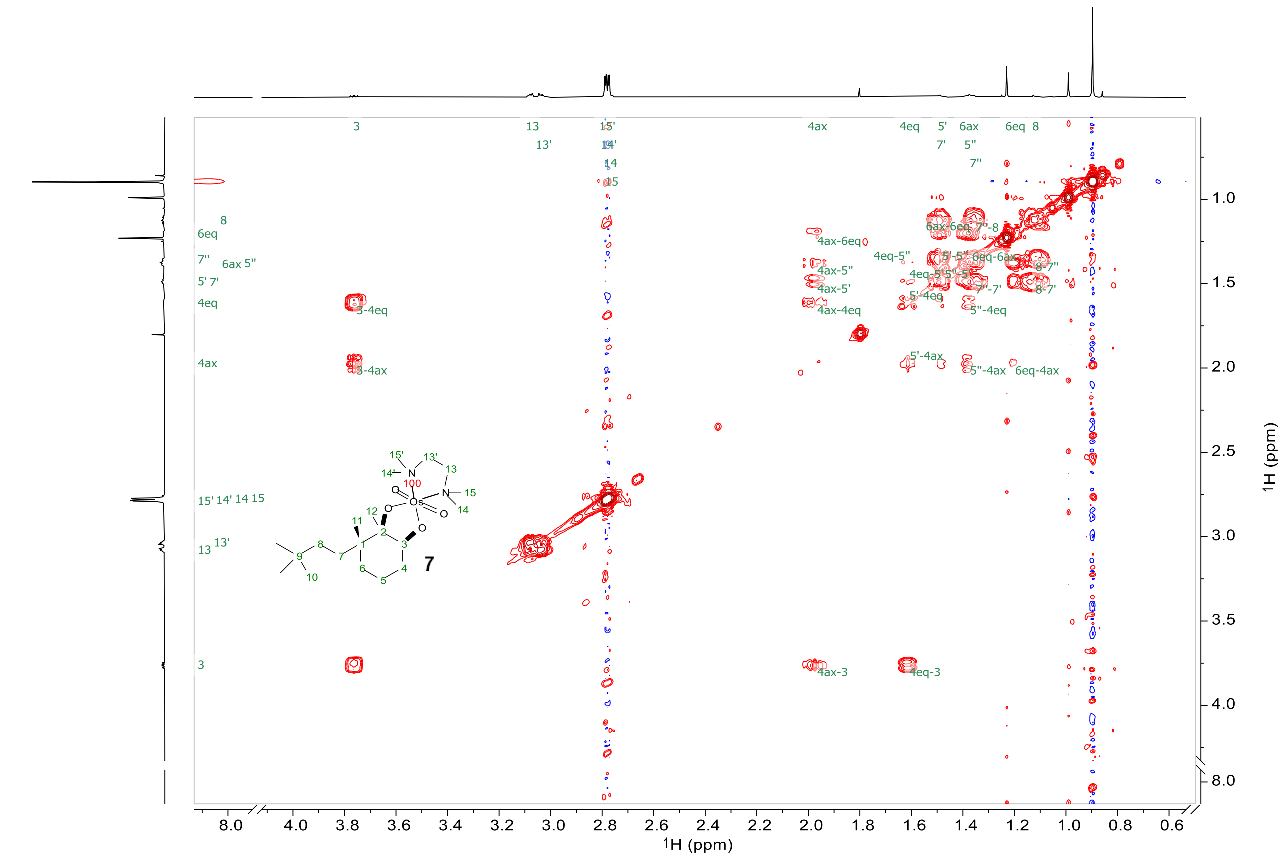

1H{off},NOESY, 600.20 MHz,CDCl3,298.0K, pulse sequence: noesygpphpp

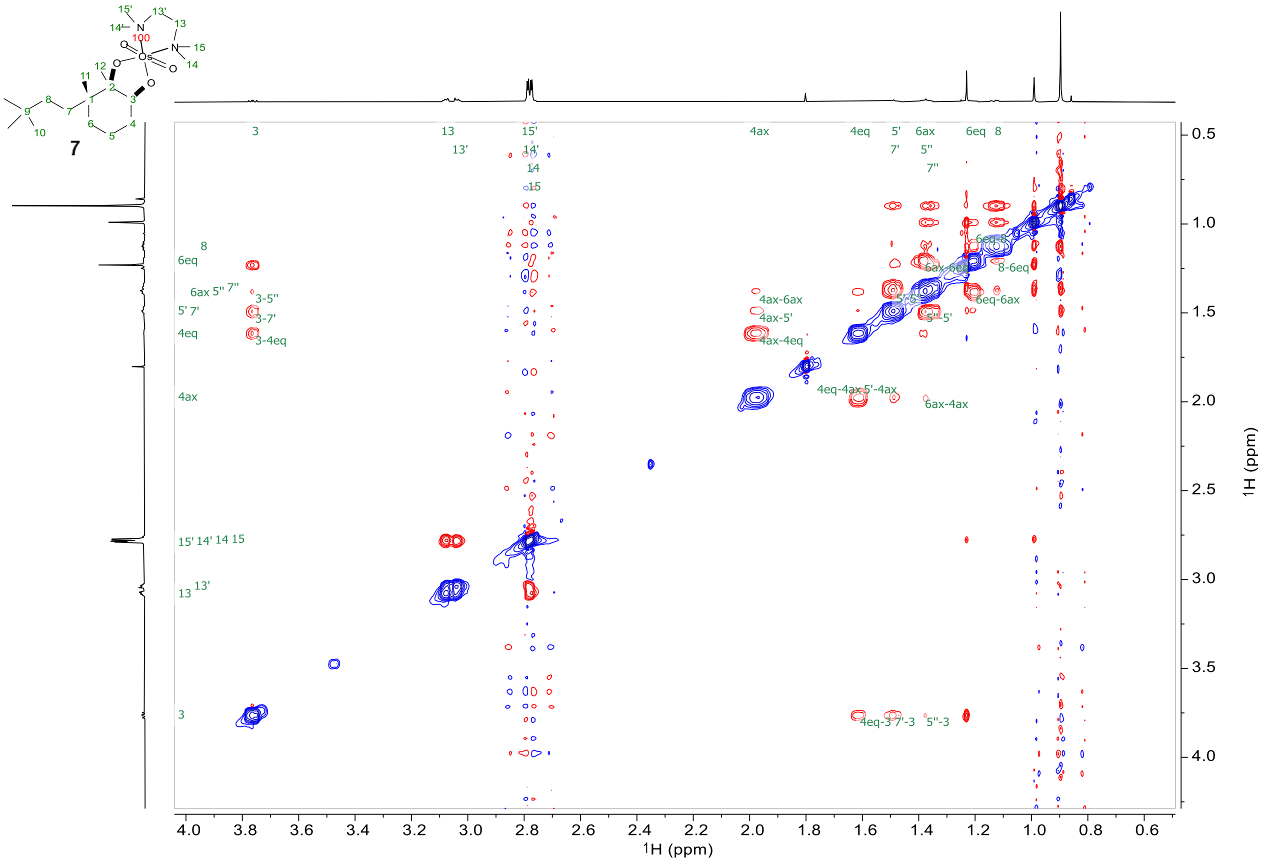

1H{15N},HMBC, 600.20 MHz,CDCl3,298.0K, pulse sequence: hmbcgpndqf

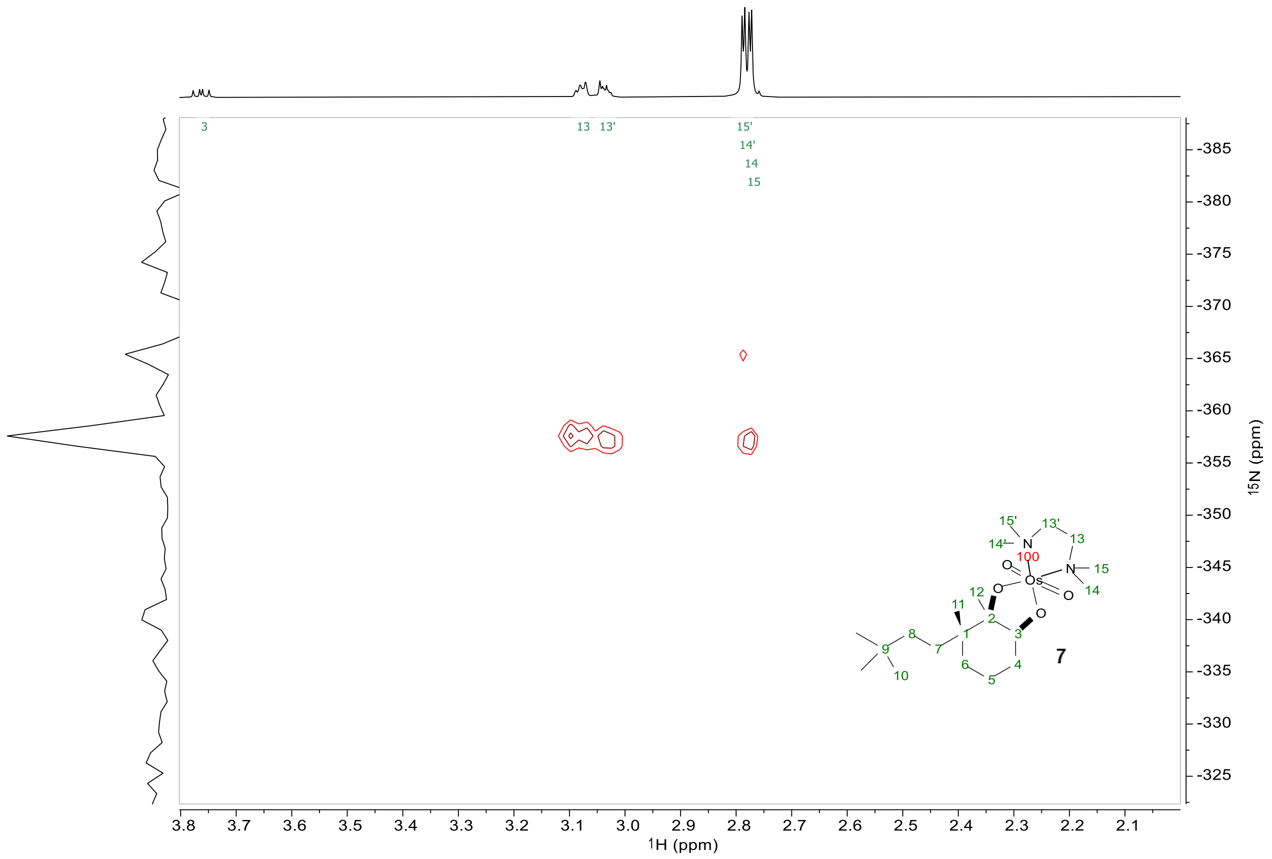

The sample is a ~3:1 mixture of 2 diasteremers.

NMR data supports the following structure of the major diastereomer:

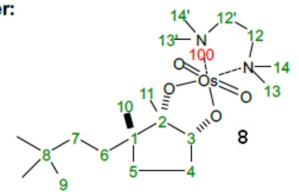

8

Observed **NOEs** are in good agreement with the X-ray structure

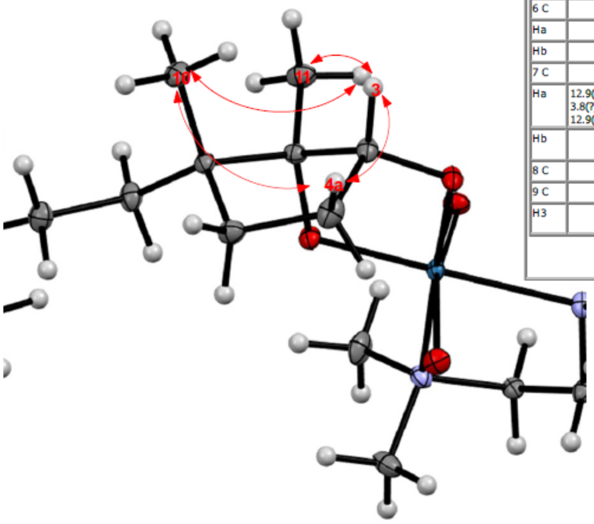

User Report
WEV-WF-316-01

Atom	J	δ (ppm)	HSQC	COSY	HMBC	NOESY
1 C		48.619			4b, 6a, 10, 11	
2 C		100.429			10, 11	
3 C		98.748	3		4a, 11	
H	9.3(?), 2.9(?)	4.462	3	3, 4a, 4b	5, 11	3, 4a, 10, 11
4 C		29.860	3, 4a			
Ha		1.921	4	3, 4b, 5a, 5b	3	3, 4b
Hb		1.455	4	3, 4a, 5a	1	4a
5 C		36.822	5a, 5b		3, 6b, 10	
Ha		1.644	5	4a, 4b, 5b		5b, 7a
Hb		1.216	5	4a, 5a		5a, 7b, 10
6 C		29.400	6a, 6b		10	
Ha		1.648	6	6b, 7a, 7b	1, 10	9
Hb		1.203	6	6a, 7a, 7b	5, 10	9
7 C		38.519	7a, 7b			
Ha	12.9(?), 3.8(?), 12.9(?)	0.977	7	6a, 6b	8, 9	5a, 7b, 9, 10
Hb		1.141	7	6a, 6b	8, 9	5b, 7a, 9, 10
8 C		30.443			7a, 7b, 9	
9 C		29.581	9		7a, 7b, 9	
H3		0.761	9		8, 9	6a, 6b, 7a, 7b

Atom	J	δ (ppm)	HSQC	COSY	HMBC	NOESY
10 C		24.573	10		6a, 6b	
H3		0.884	10		1, 2, 5, 6	3, 5b, 7a, 7b, 11
11 C		16.887	11		3	
H3		1.160	11		1, 2, 3	3, 10
12 C		64.173	12a, 12b		12'a, 12'b, 13, 14	
Ha		3.026	12	12'a, 12'b, 12b	12', 13, 14	
Hb		2.914	12	12'a, 12'b, 12a	12', 13, 14	
12' C		64.028	12'a, 12'b		12a, 12b, 13', 14'	
Ha		3.010	12'	12'b, 12a, 12b	12, 13', 14'	
Hb		2.878	12'	12'a, 12a, 12b	12, 13', 14'	
13 C		52.006	13		12a, 12b, 14	
H3		2.699	13		12, 14	
13' C		51.342	13'		12'a, 12'b, 14'	
H3		2.744	13'		12', 14'	
14 C		51.384	14		12a, 12b, 13	
H3		2.680	14		12, 13	
14' C		50.603	14'		12'a, 12'b, 13'	
H3		2.673	14'		12', 13'	
100 N						

Remarks: The minor diastereomer was not assigned in detail. In the 2D NOESY H-3 of the minor has not cross peak to H-10, but to an additional CH2 group. This is in line with the expectation for the other diastereomer.

P-ID: ML00xxx
Measured on: 27/04/2023
CHIFFRE: WEV-WF-116-01
ELNA#: 9599
Client: Vijay Wakchaure
Group: List
Spectroscopist: Leutzsch
Analysed on: 27/04/2023
Analysed by: Leutzsch
Amount: 9.0 mg
Solvent: CDCl3
Reference: 1H+13C on solvent,other nuclei w/ xiref
Temperature: 298 K
Spectrometer: av600neo
Probe: cryoBBO
Experiments: 1H-zg30, 13C-zgpg30, 1H-13C-hsqcedetgpsisp2.3, 1H-13C-hmbcetgpl3nd, 1H-1H-cosygpppqf, 1H-1H-noesygpphpp

1H{off,off},1D, 600.20 MHz,CDCl3,298.0K, pulse sequence: zg30

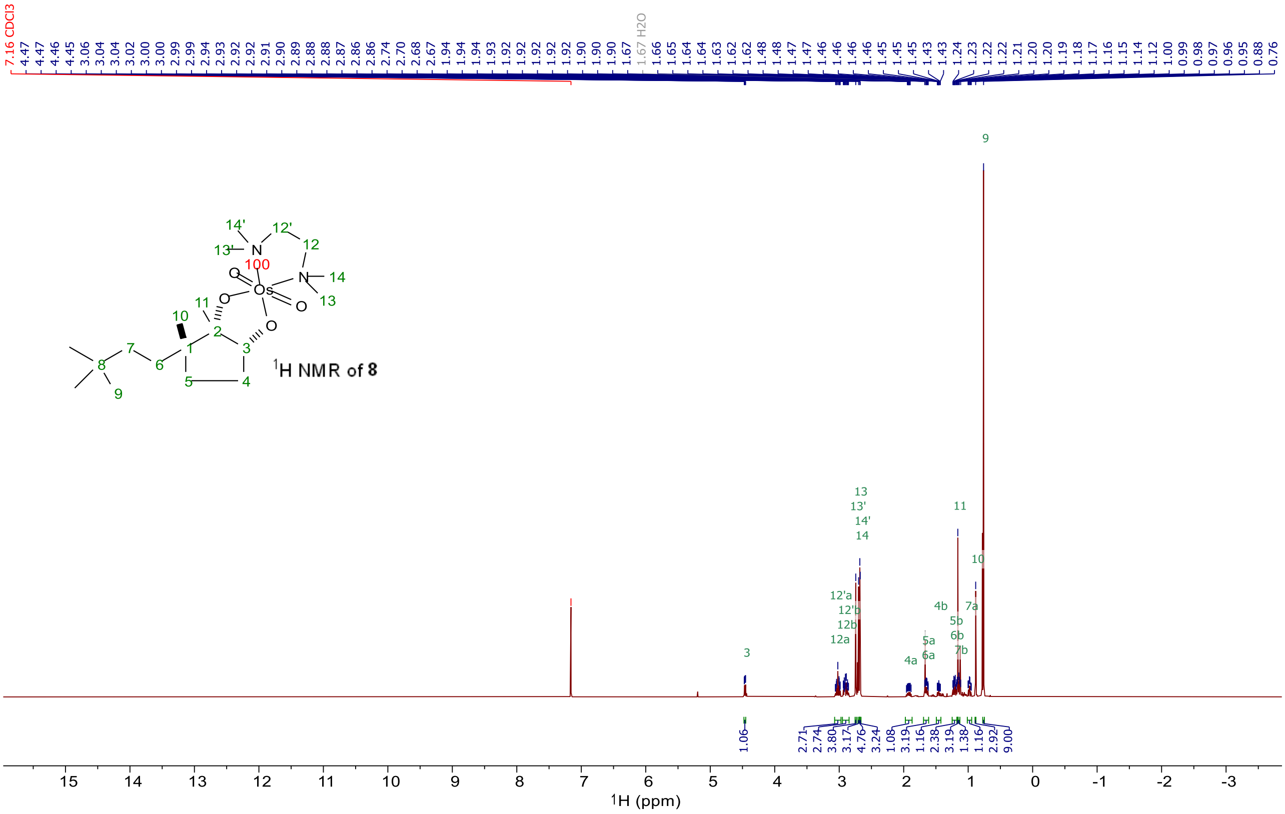

13C{1H,off},1D, 150.94 MHz,CDCl3,298.0K, pulse sequence: zgpg30

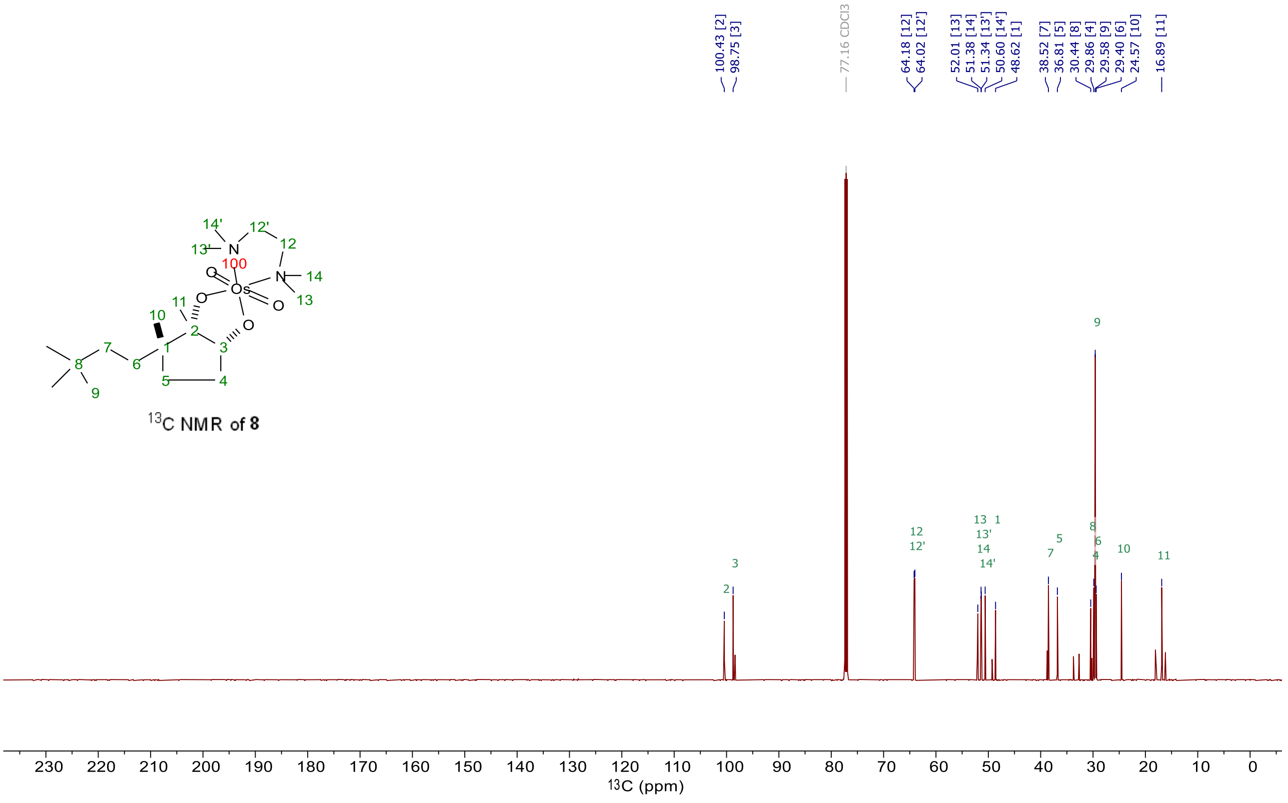

1H{13C,off},HSQC-EDITED, 600.20 MHz,CDCl3,298.0K, pulse sequence: hsqcedetgpsisp2.3

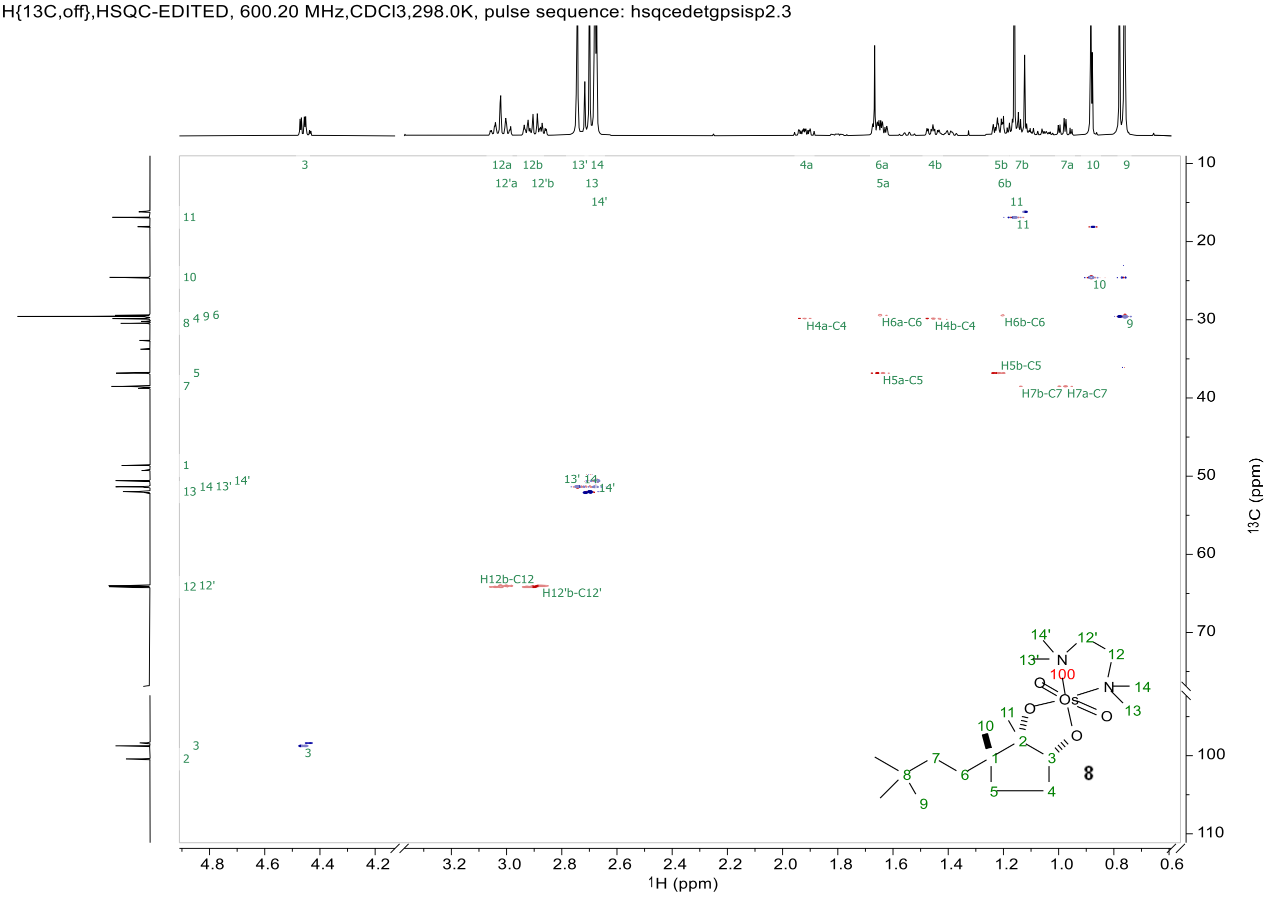

1H{13C,off},HMBC, 600.20 MHz,CDCl3,298.0K, pulse sequence: hmbcetgpl3nd

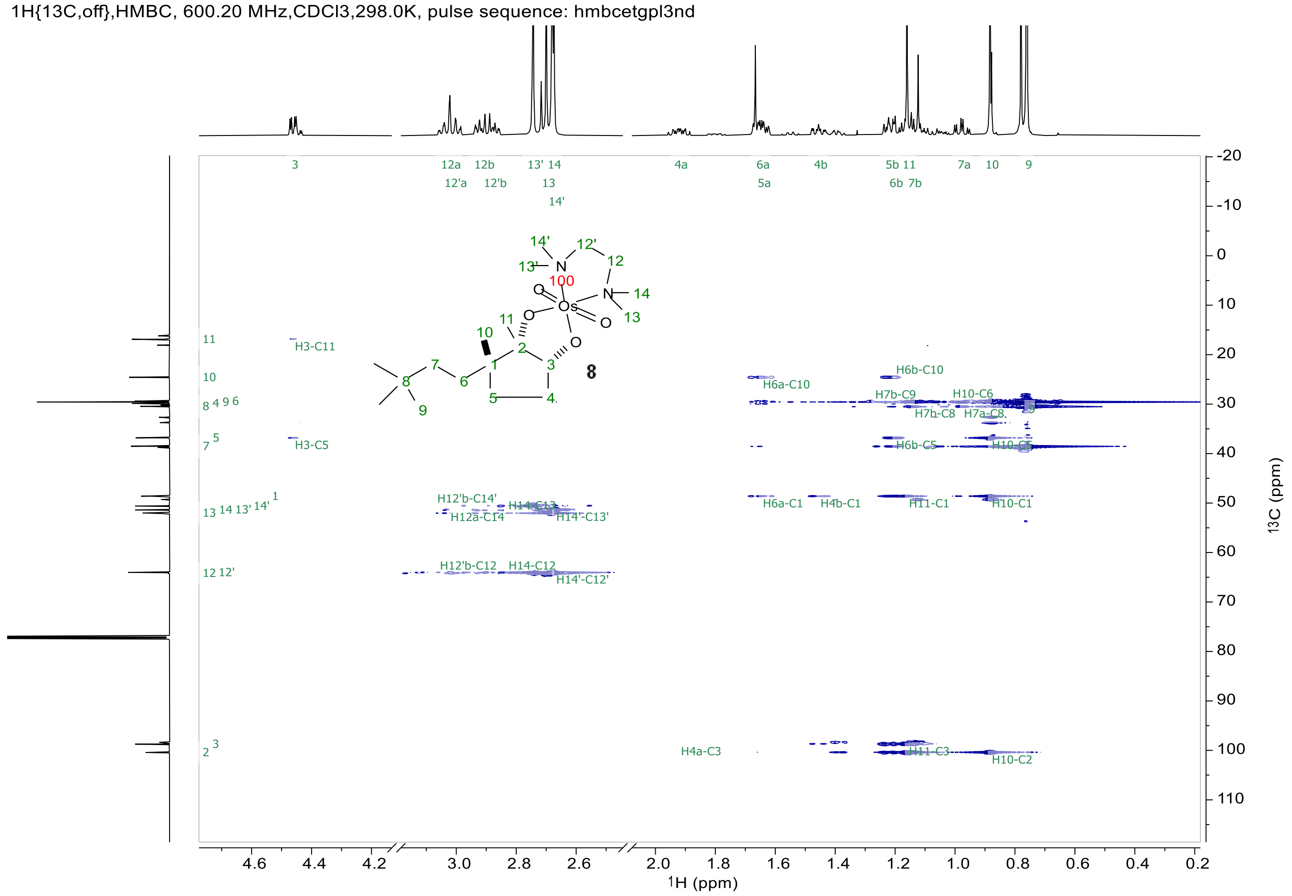

1H{off,off},COSY, 600.20 MHz,CDCl3,298.0K, pulse sequence: cosygpppqf

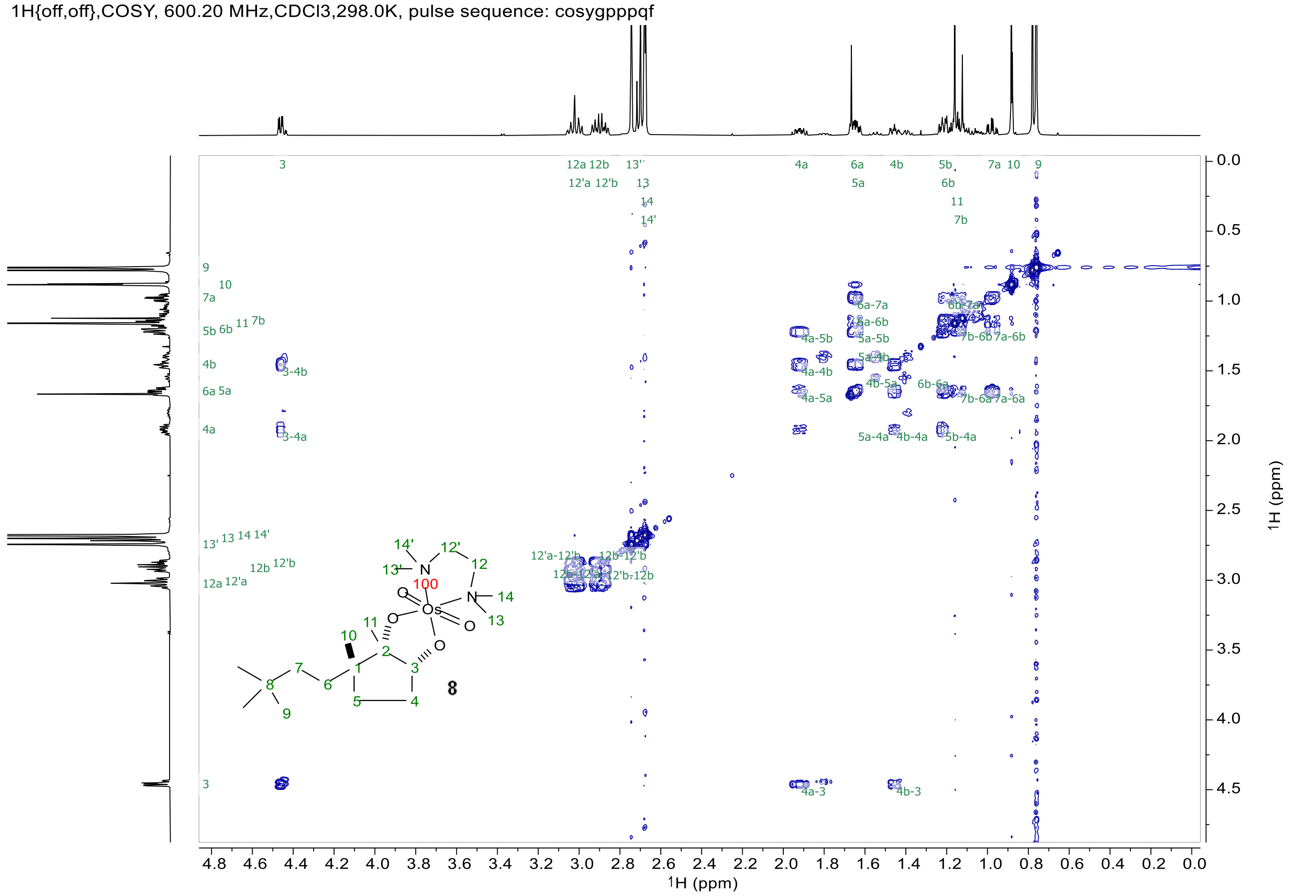

1H{off,off},NOESY, 600.20 MHz,CDCl3,298.0K, pulse sequence: noesygpphpp

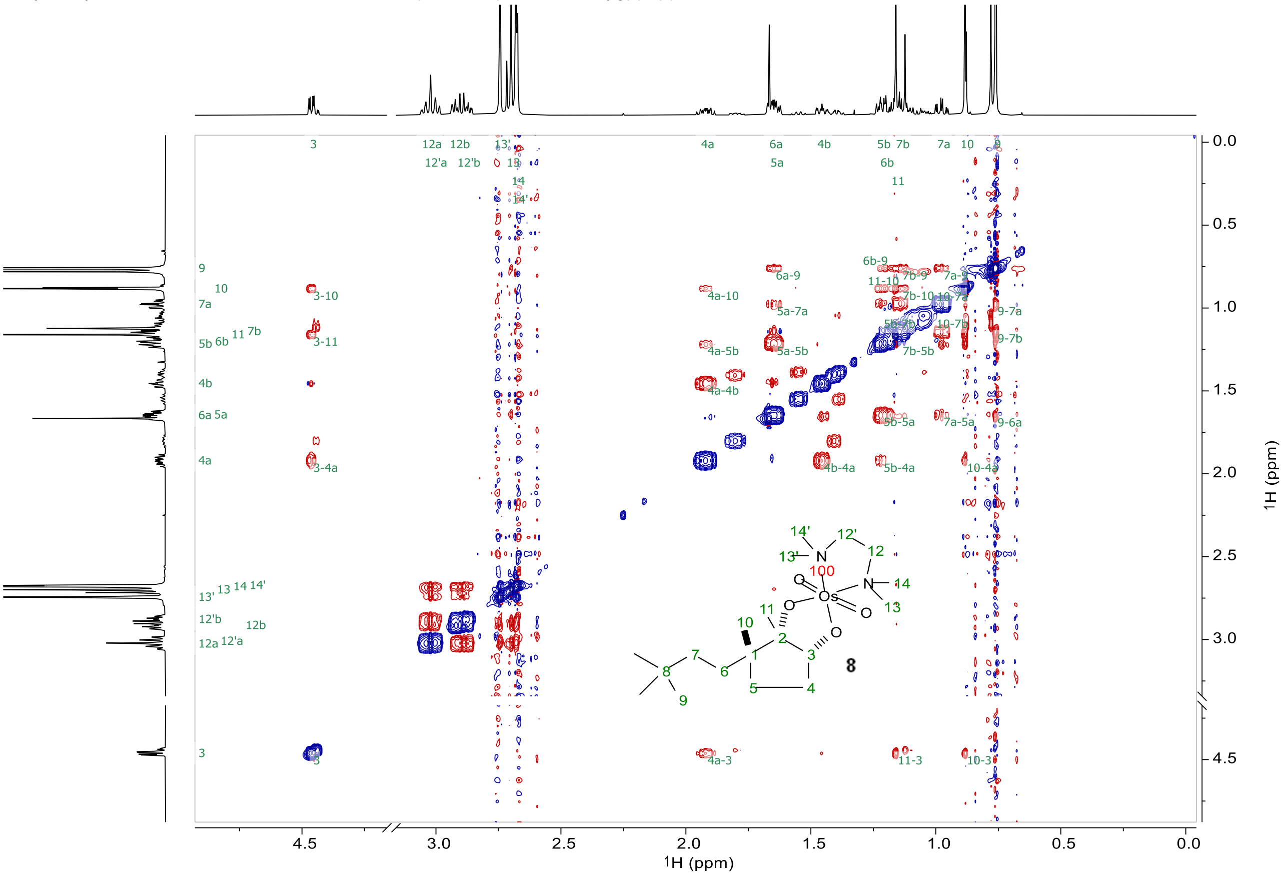

1H{15N,off},HMBC, 600.20 MHz,CDCl3,298.0K, pulse sequence: hmbcgpndqf

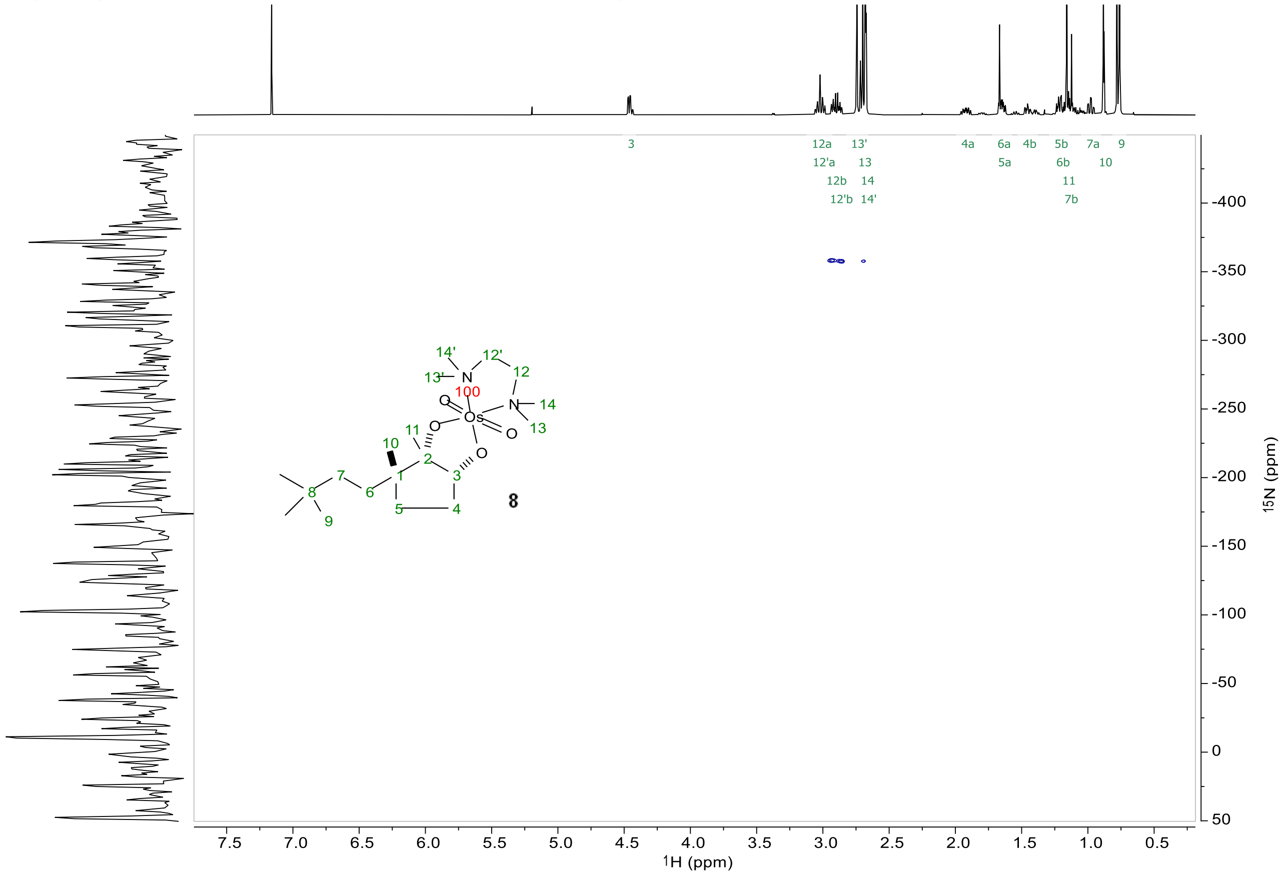

NMR data supports the following structure

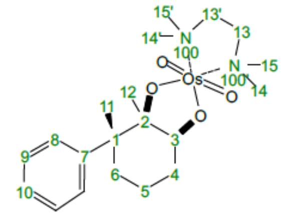

Observed NOEs are mostly good agreement with the X-ray structure.

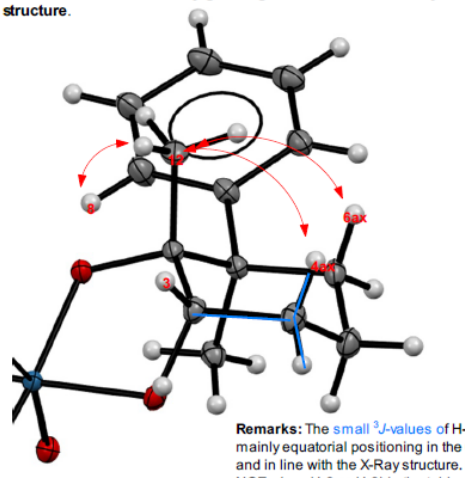

Remarks: The small ^3J-values of H-3 hint for the mainly equatorial positioning in the 6-membered ring and in line with the X-Ray structure. However, some NOEs (e.g. H-8<->H-3) in the table cannot be explained by the conformation observed in the X-Ray, but can be explained by contributations from a different ring conformer (Ph and H-3 in axial position) fo the average NMR signal. This NOE does not match to the other diastereomer.

User Report
WEV-WF-315-01

Atom	J	δ (ppm)	HSQC	COSY	HMBC	NOESY
1 C		47.841			3, 5ax, 5eq, 6ax, 6eq, 8, 11, 12	
2 C		87.580			3, 4ax, 4eq, 6ax, 6eq, 11, 12	
3 C		98.068	3		4ax, 4eq, 5ax, 5eq, 12	
H	4.6(4ax), 2.9(4eq)	4.282	3	4ax, 4eq	1, 2, 4, 5, 12	4ax, 4eq, 8, 12
4 C		31.643	4ax, 4eq		3, 5ax, 5eq, 6ax, 6eq	
Hax	12.9(5ax), 4.6(3), 14.6(?), 4.8(?)	1.908	4	3, 4eq, 5ax, 5eq, 6eq	2, 3, 5, 6	3, 4eq, 5eq, 12
Heq	2.9(3)	2.387	4	3, 4ax	2, 3	3, 4ax, 5ax
5 C		18.332	5ax, 5eq		3, 4ax, 6ax	
Hax	12.9(4ax), 12.9(6ax)	2.091	5	4ax, 5eq	1, 3, 4, 6	4eq, 5eq, 6eq
Heq	3.1(6ax)	1.617	5	4ax, 5ax	1, 3, 4, 6	4ax, 5ax
6 C		37.236	6ax, 6eq		4ax, 5ax, 5eq, 11	
Hax	12.2(6eq), 12.9(5ax), 3.1(5eq)	2.016	6	6eq	1, 2, 4, 5, 11	6eq, 8, 11, 12
Heq	12.2(6ax)	1.840	6	4ax, 6ax	1, 2, 4	5ax, 6ax, 8, 11
7 C		151.156			9, 11	
8 C		127.821	8		8, 10	
H	7.7(9)	7.635	8	9	1, 8, 10	3, 6ax, 6eq, 11, 12, 14
9 C		127.327			9	
H	7.7(8)	7.273		8, 10	7, 9	
10 C		125.140			8	
H		7.161		9	8	

Atom	J	δ (ppm)	HSQC	COSY	HMBC	NOESY
11 C		25.149	11		6ax, 7''	
H3		1.602	11		1, 2, 6, 7	6ax, 6eq, 8, 12
12 C		24.044	12		3	
H3		0.903	12		1, 2, 3	3, 4ax, 6ax, 8, 11
13 C		64.342	13a, 13b		13'a, 13'b, 14, 15	
Ha		2.979	13	13'a, 13'b, 13b	13', 14, 15	
Hb		3.244	13	13'a, 13'b, 13a	13', 14, 15	
13' C		64.366	13'a, 13'b		13a, 13b, 14', 15'	
Ha		2.942	13'	13'b, 13a, 13b	13, 14', 15'	
Hb		3.186	13'	13'a, 13a, 13b	13, 14', 15'	
14 C		53.317	14		13a, 13b, 15	
H3		3.071	14		13, 15, 100	8
14' C		51.627	14'		13'a, 13'b, 15'	
H3		2.905	14'		13', 15', 100'	
15 C		50.863	15		13a, 13b, 14	
H3		2.763	15		13, 14, 100	
15' C		50.460	15'		13'a, 13'b, 14'	
H3		2.820	15'		13', 14', 100'	
100 N		-358.816			14, 15	
100' N		-358.571			14', 15'	

P-ID: ML00xxx
Measured on: 27/04/2023
CHIFFRE: WEV-WF-115-01
ELNA#: 9598
Client: Vijay Wakchaure
Group: List
Spectroscopist: Leutzsch
Analysed on: 27/04/2023
Analysed by: Leutzsch
Amount: 8.0 mg
Solvent: CDCl3
Reference: 1H+13C on solvent,other nuclei w/ xiref
Temperature: 298 K
Spectrometer: av600neo
Probe: cryoBBO
Experiments: 1H-zg30, 13C-zgpg30, 1H-13C-hsqcedetgpsisp2.3, 1H-13C-hmbcetgpl3nd, 1H-1H-cosygpppqf, 1H-1H-noesygpphpp, 1H-15N-hmbcgpndqf

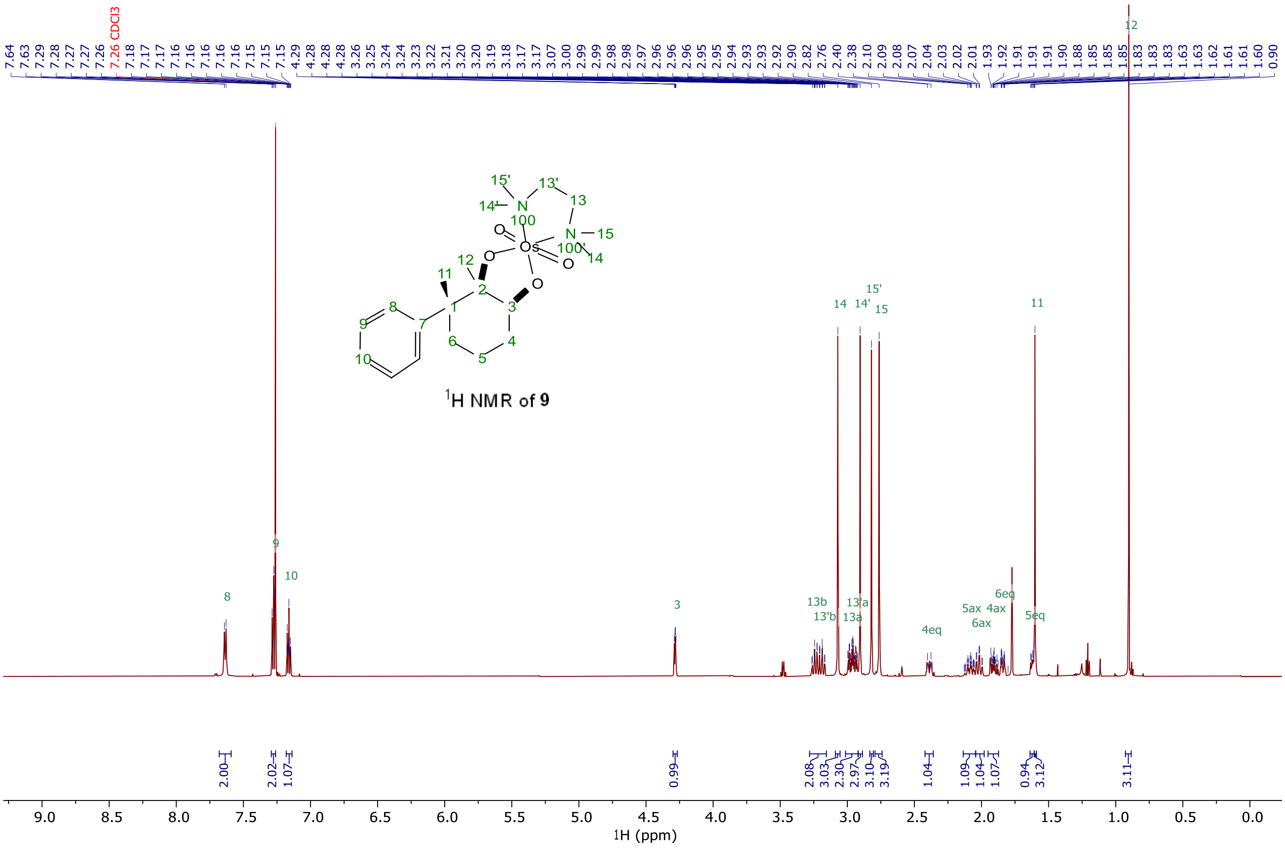

^{1}H NMR of **9**

13C{1H,off},1D, 150.94 MHz,CDCl3,298.0K, pulse sequence: zgpg30

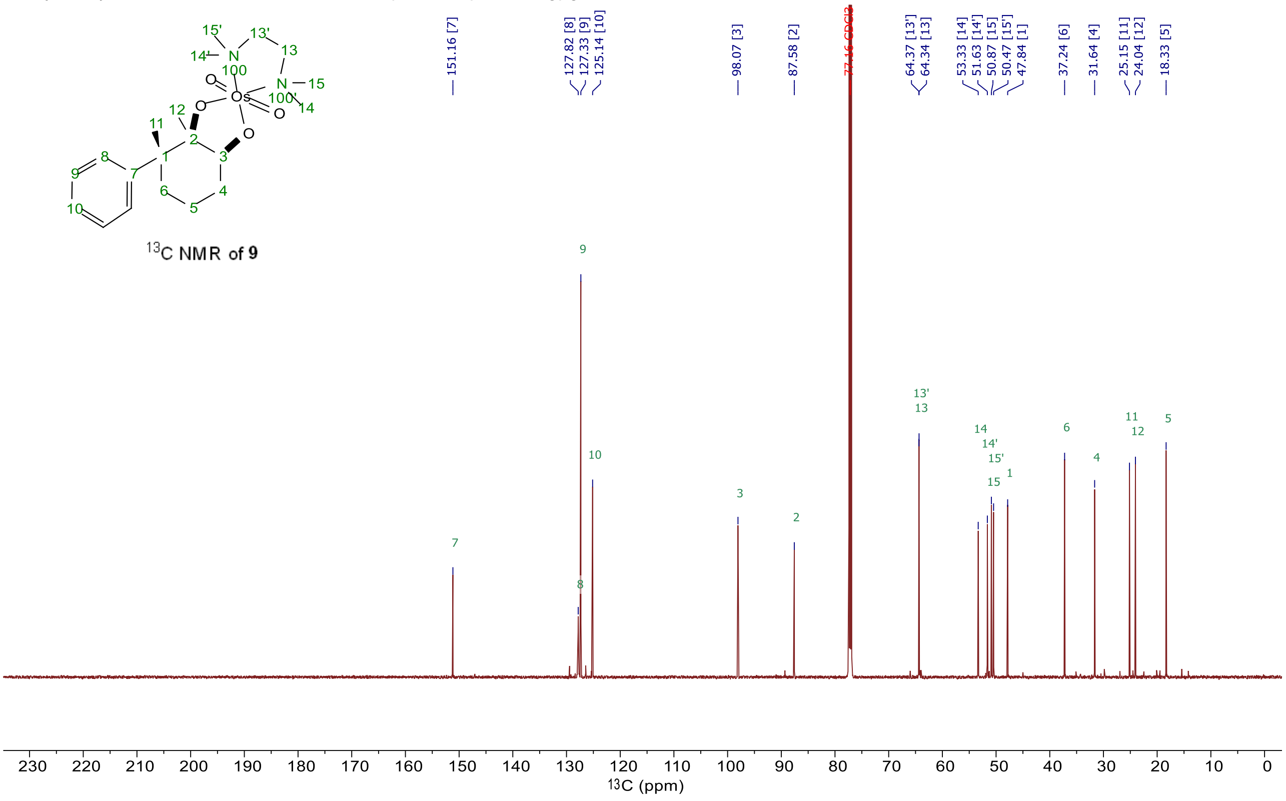

1H{13C,off},HSQC-EDITED, 600.20 MHz,CDCl3,298.0K, pulse sequence: hsqcedetgpsisp2.3

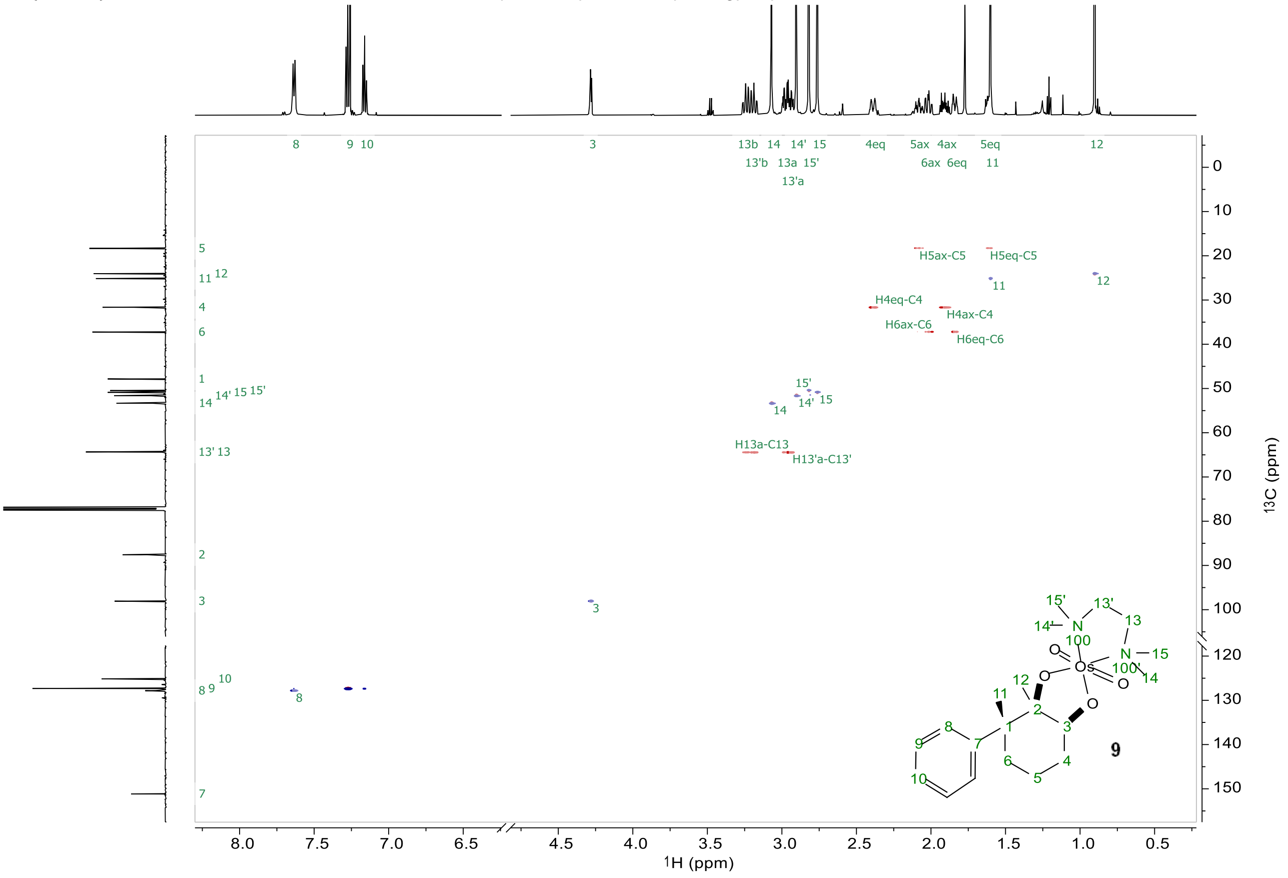

1H{13C,off},HMBC, 600.20 MHz,CDCl3,298.0K, pulse sequence: hmbcetgpl3nd

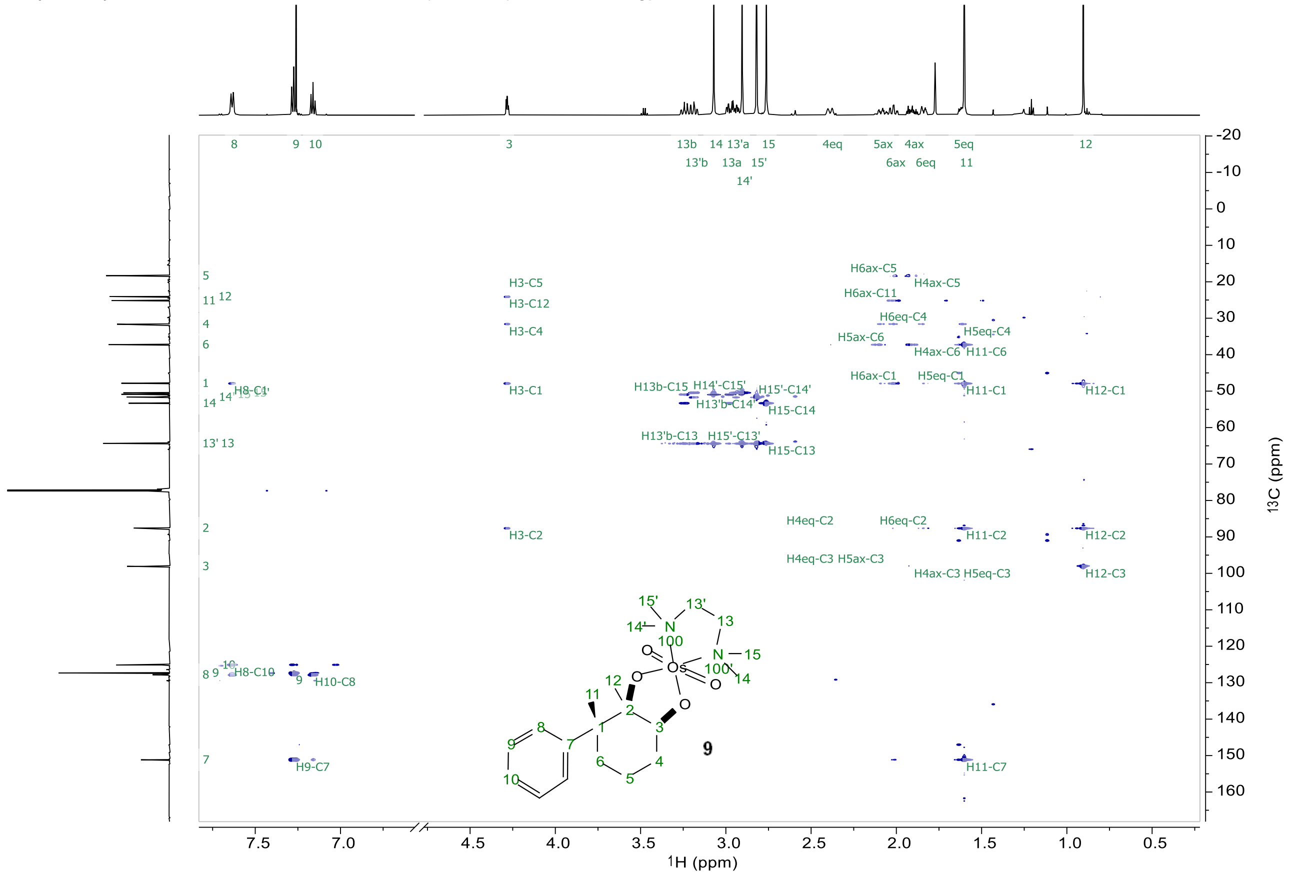

1H{off,off},COSY, 600.20 MHz,CDCl3,298.0K, pulse sequence: cosygpppqf

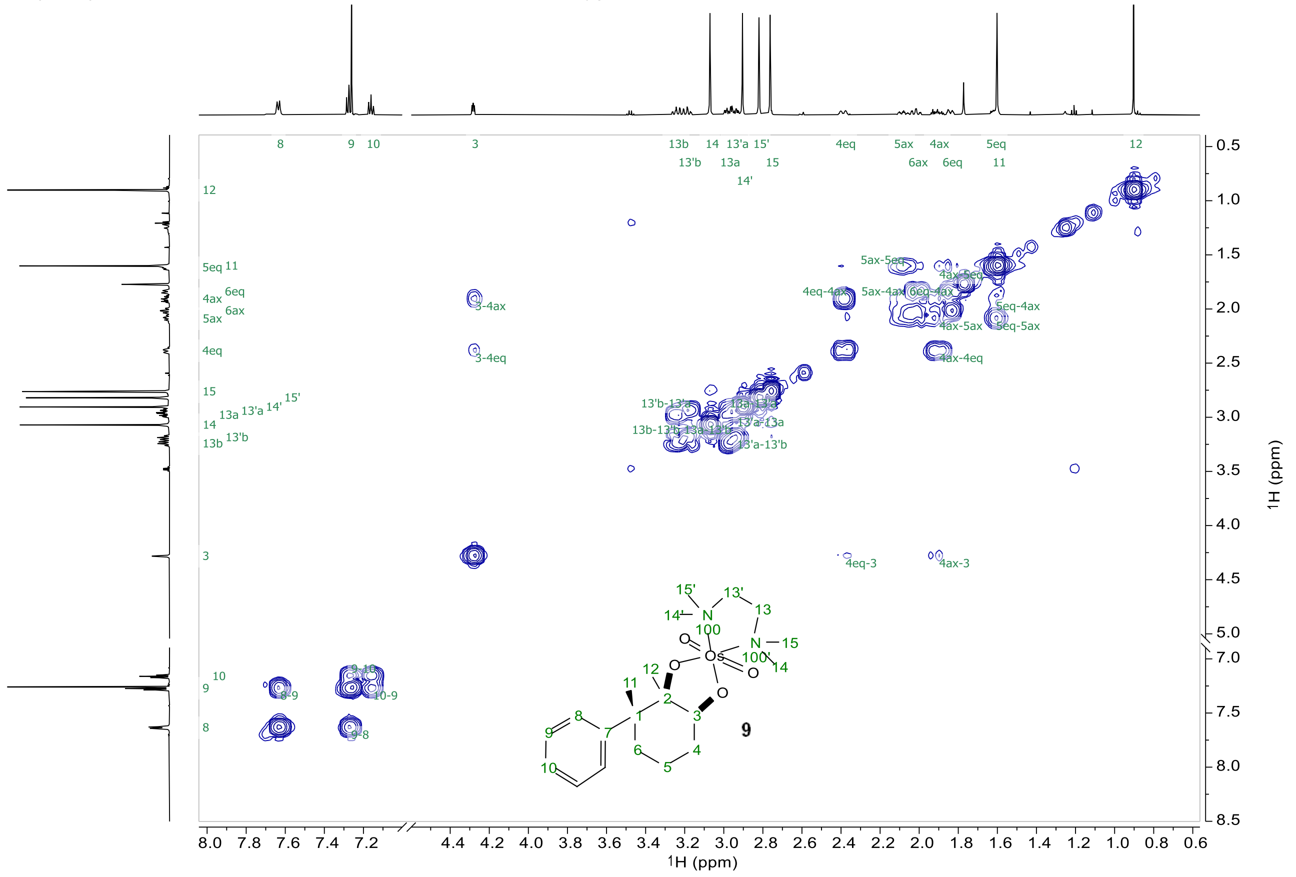

1H{off,off},NOESY, 600.20 MHz,CDCl3,298.0K, pulse sequence: noesygpphpp

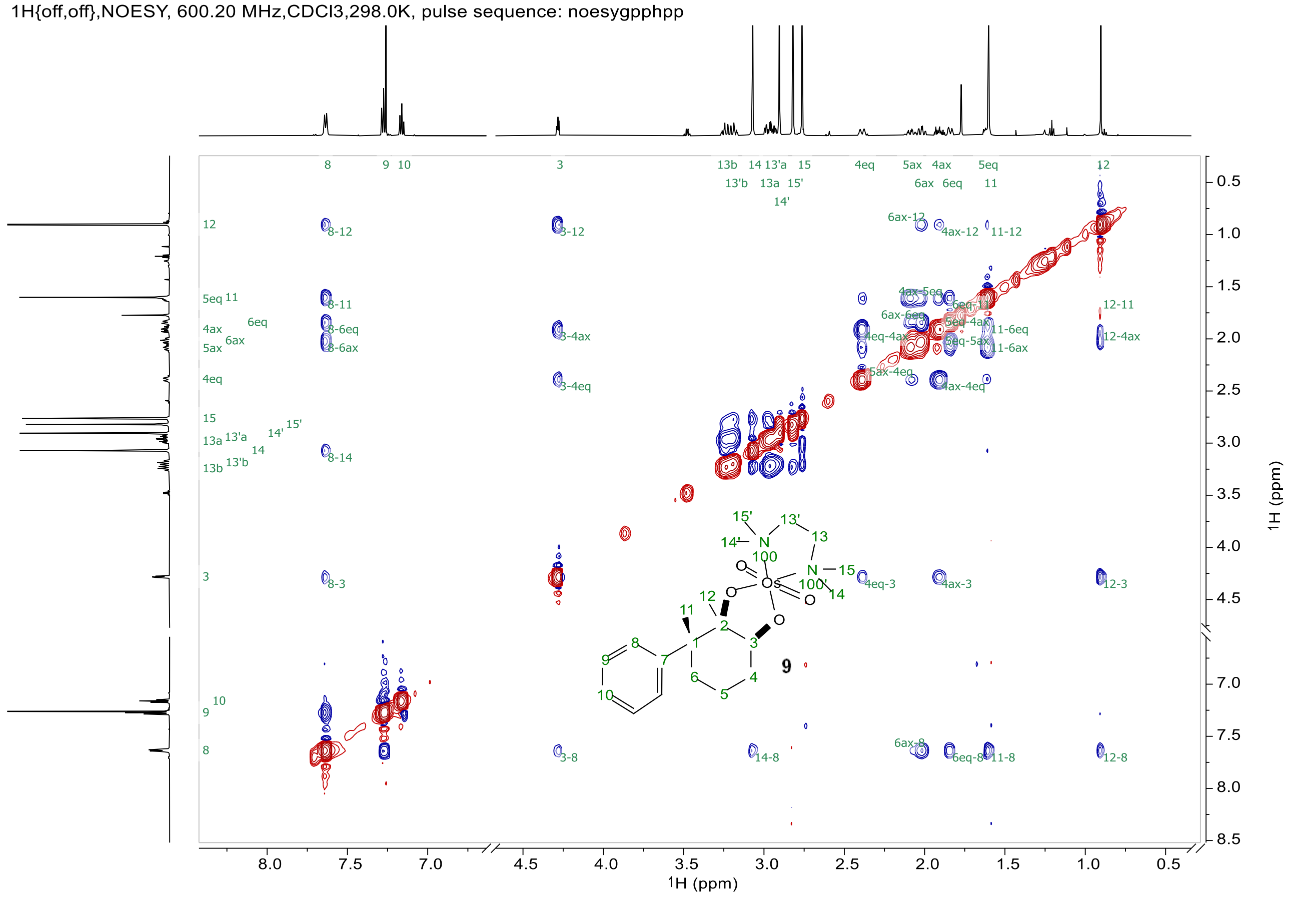

1H{15N,off},HMBC, 600.20 MHz,CDCl3,298.0K, pulse sequence: hmbcgpndqf

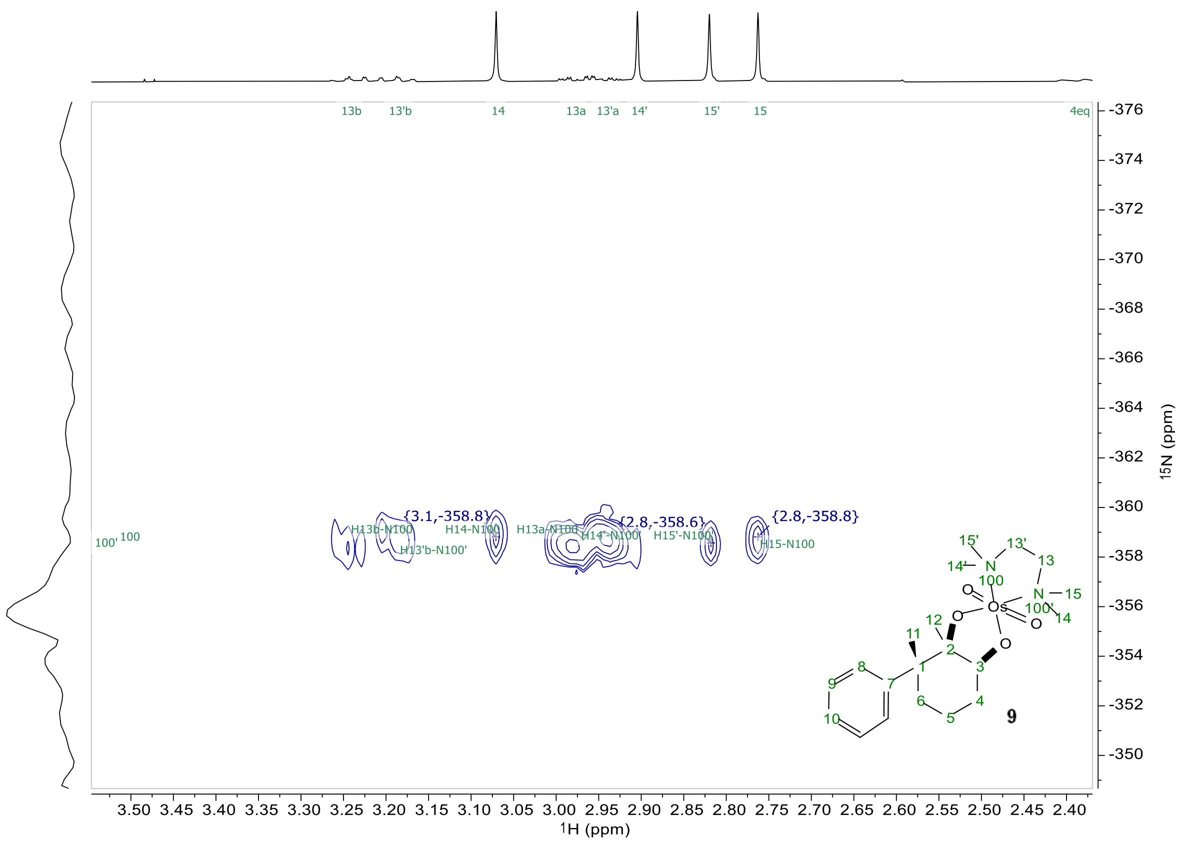

10. Copies of GC traces

GC (30.0 m BGB-176, injection temperature: 220 °C, 100 °C iso 60 min, 20 °C/min, 220 °C iso 10 min, 0.5 bar He)

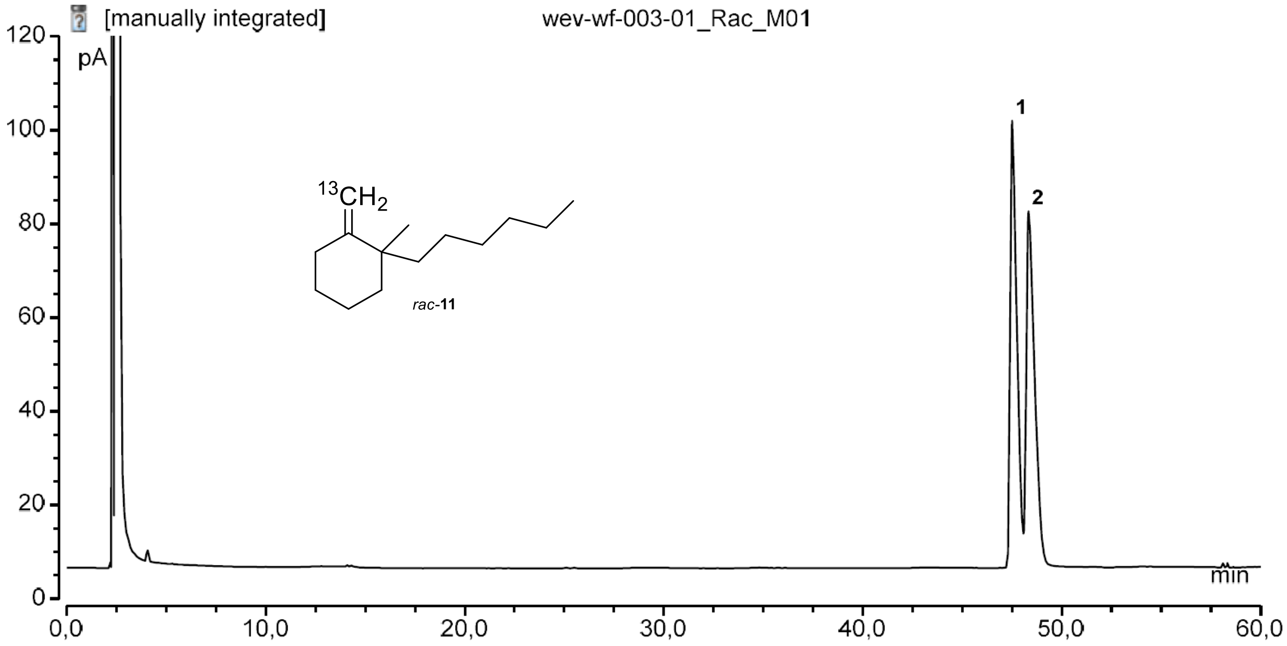

Peak #	t_R (min)	Area (%)
1	47.5	50.61
2	48.3	49,39
Total		100

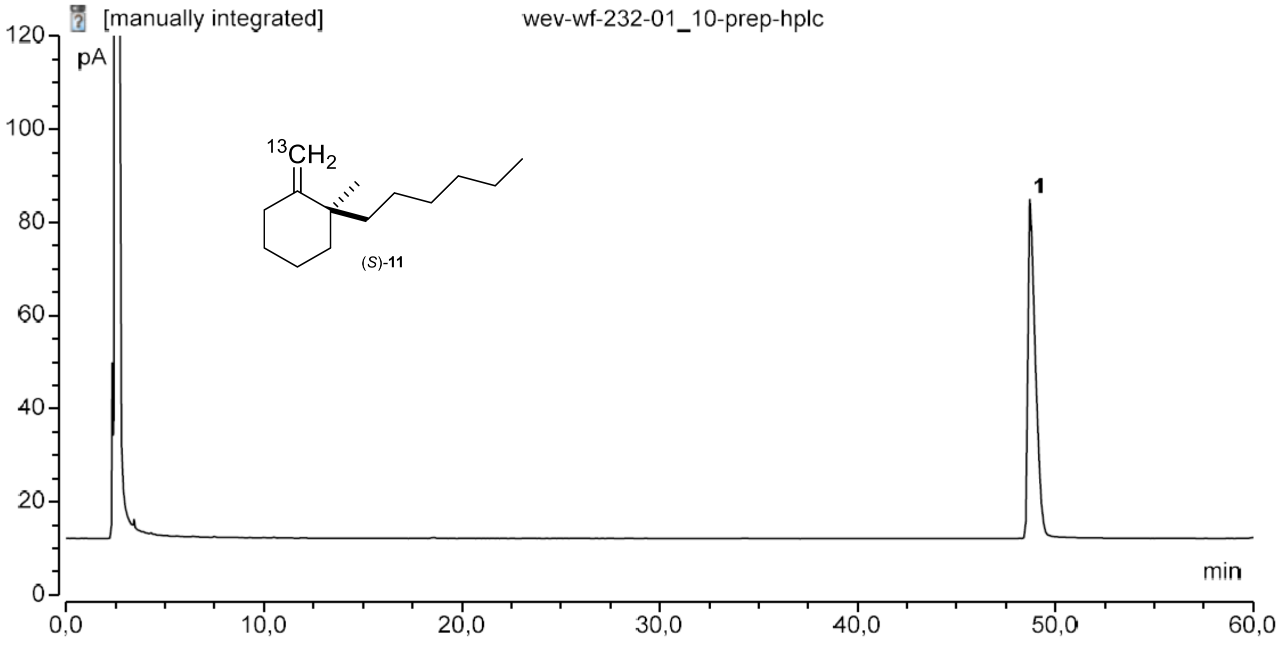

Peak #	t_R (min)	Area (%)
1	48.71	100

GC (30.0 m BGB-176, injection temperature: 220 °C, 100 °C iso 60 min, 20 °C/min, 220 °C iso 10 min, 0.5 bar He)

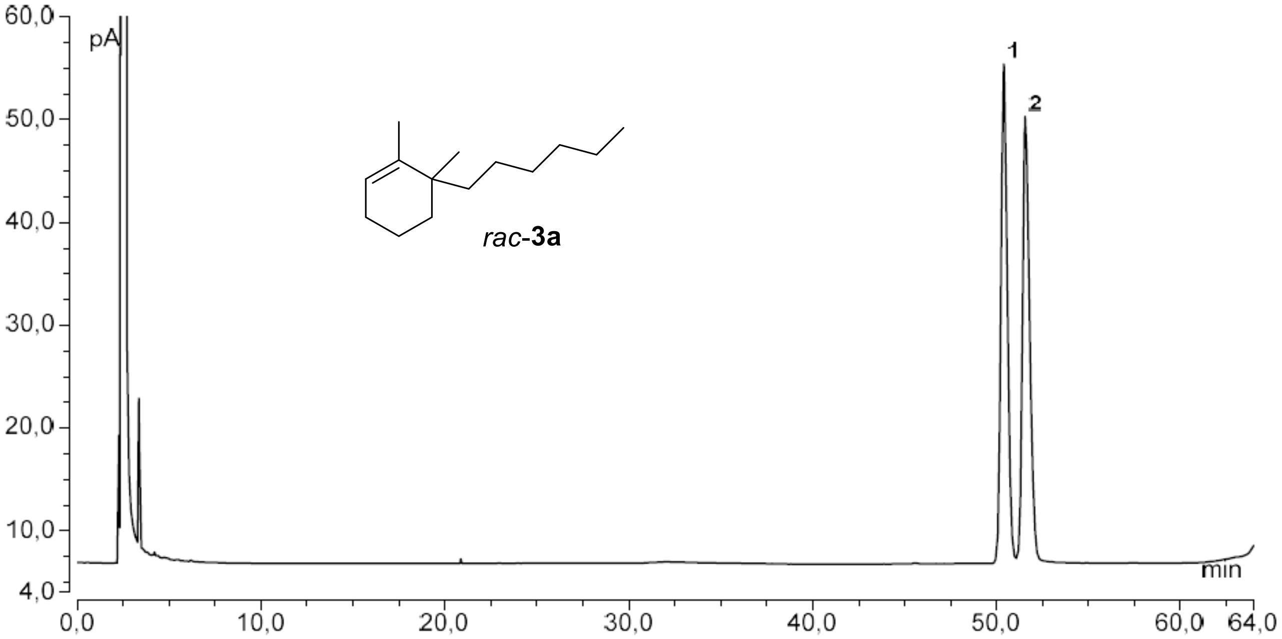

Peak #	t_R (min)	Area (%)
1	50.4	49.54
2	51.6	50.46
Total		100

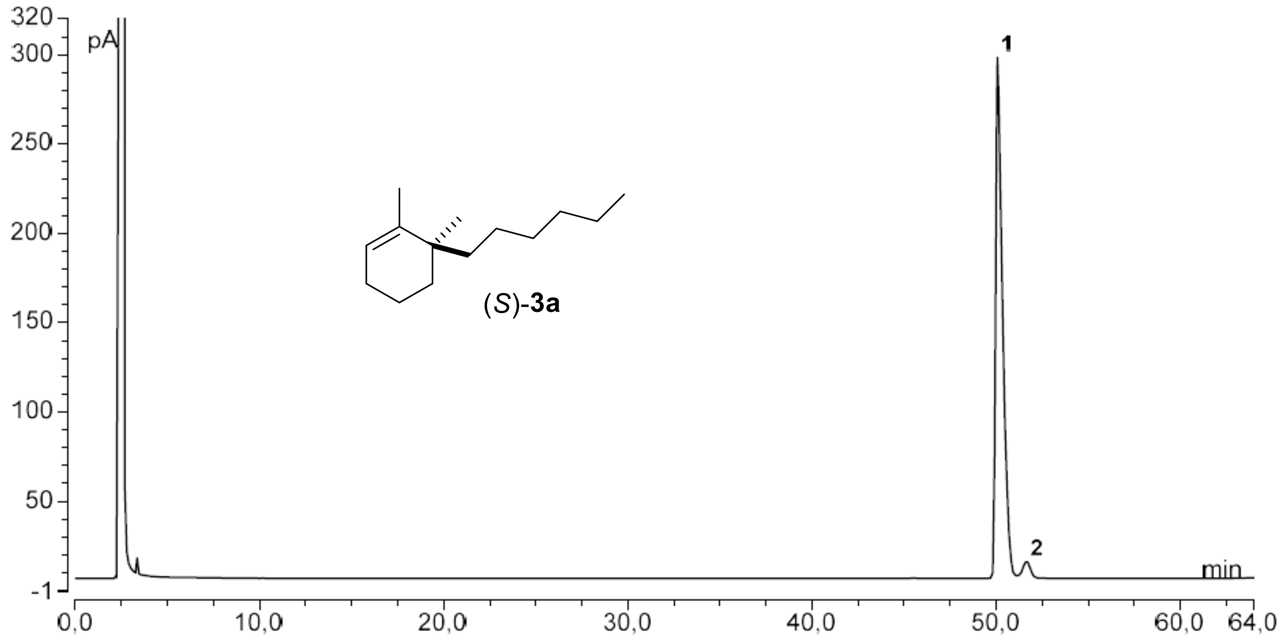

Peak #	t_R (min)	Area (%)
1	50.1	97
2	51.6	3
Total		100

GC (30.0 m BGB-176, injection temperature: 220 °C, 100 °C iso 600 min, 8 °C/min, 240 °C iso 3 min, 0.6 bar H_2)

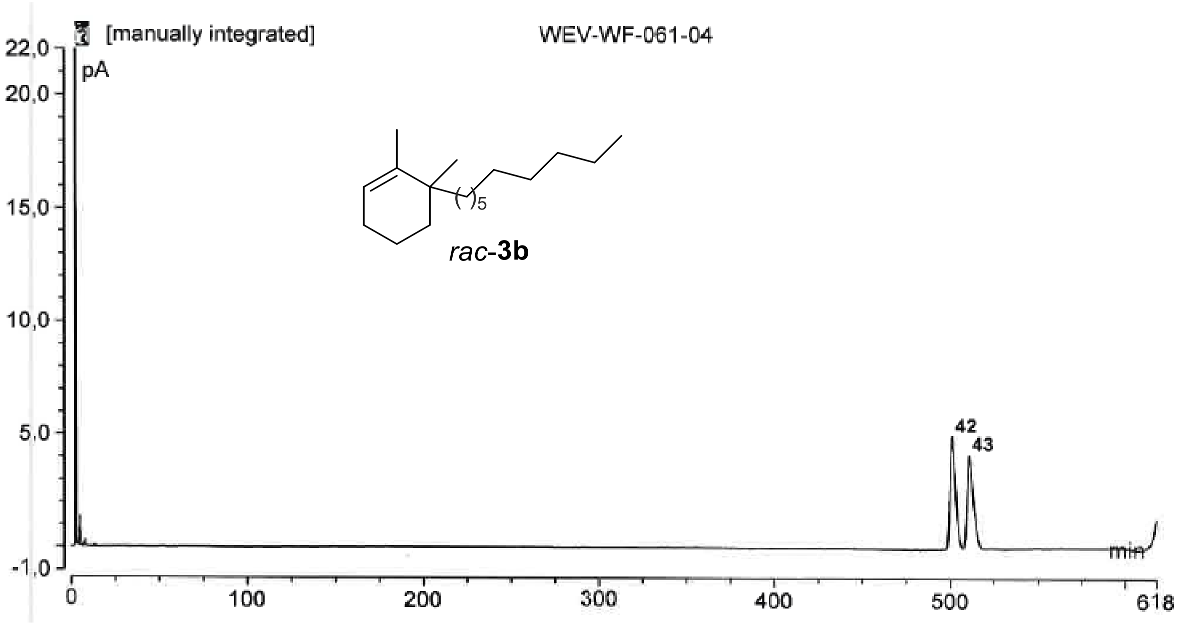

Peak #	t_R (min)	Area (%)
1	501.1	50.17
2	510.6	49.83
Total		100

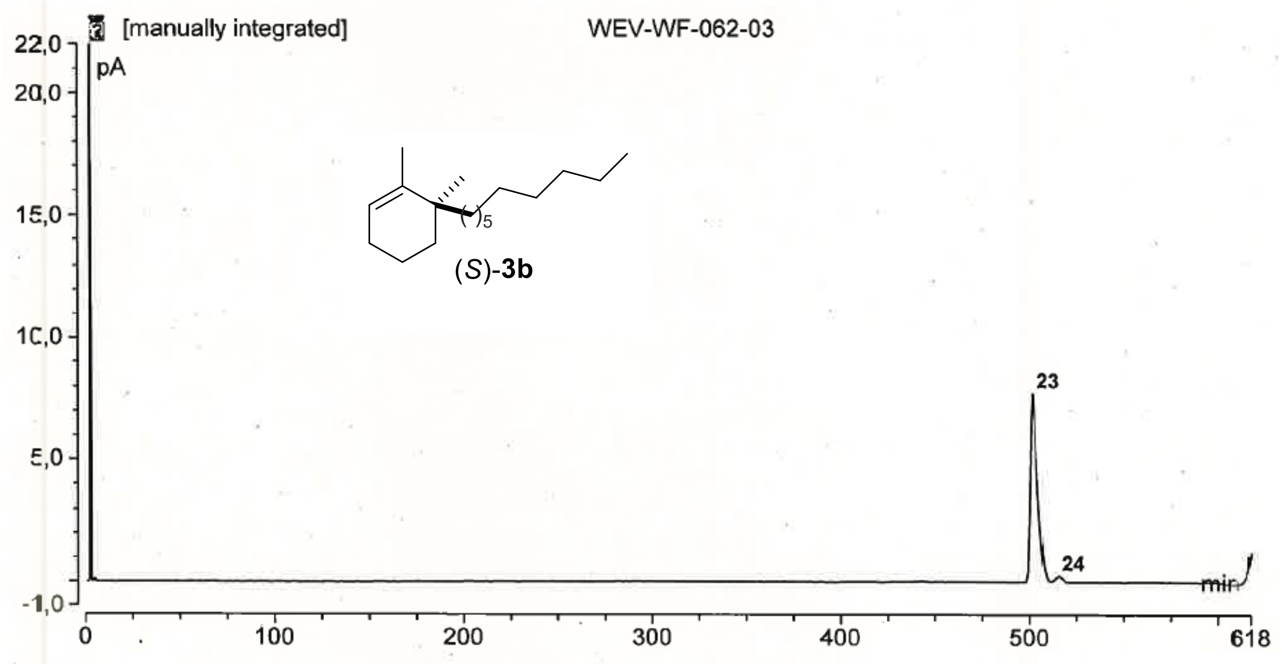

Peak #	t_R (min)	Area (%)
1	501.6	97.5
2	515.2	2.5
Total		100

GC (30.0 m CycloSil-B, injection temperature: 220 °C, 85 °C iso 43 min, 20 °C/min, 220 °C iso 5 min, 0.5 bar He)

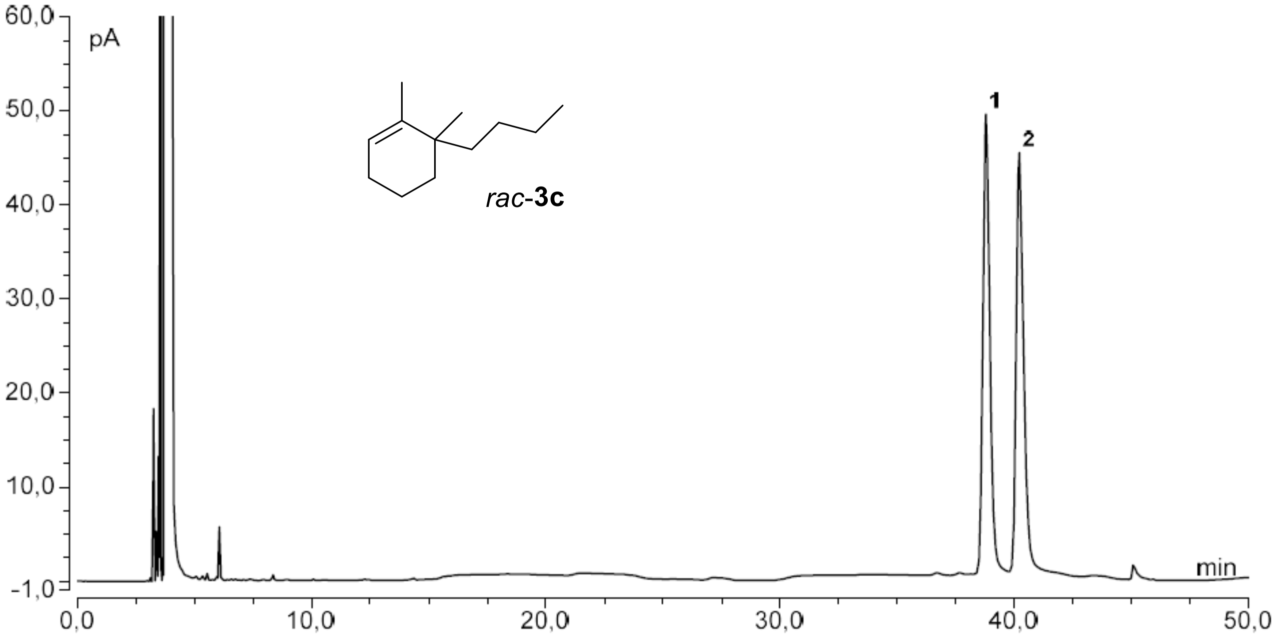

Peak #	t_R (min)	Area (%)
1	38.9	50.17
2	40.2	49.83
Total		100

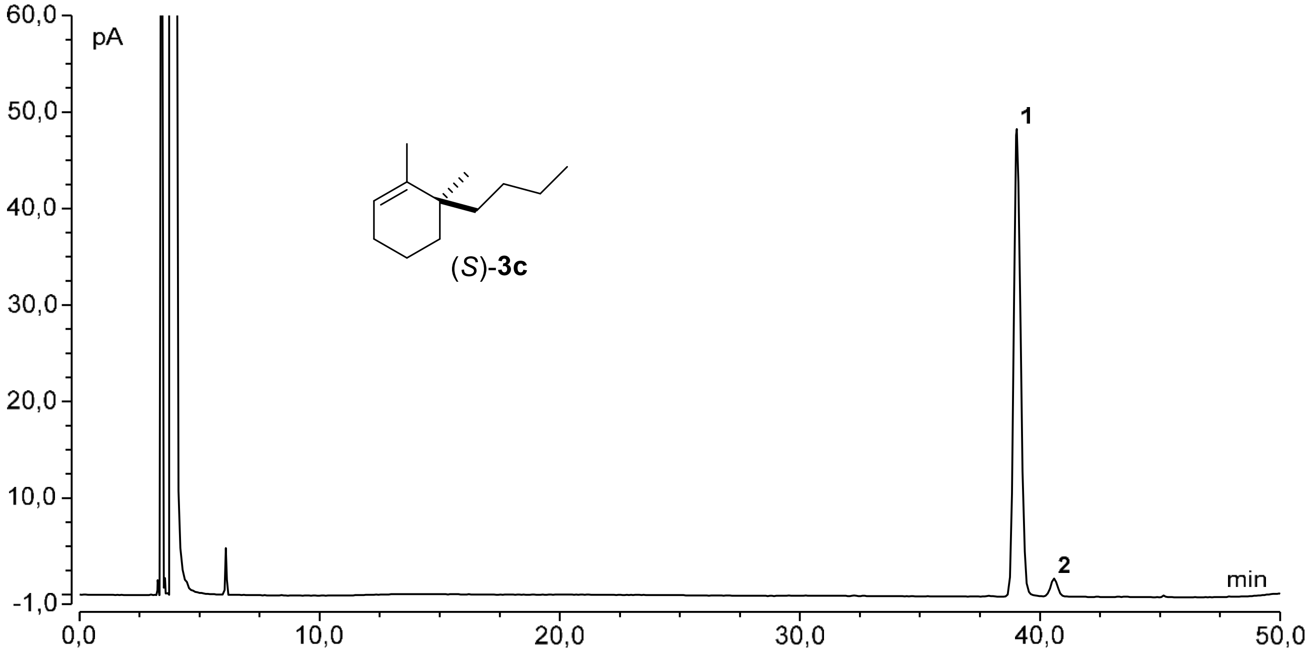

Peak #	t_R (min)	Area (%)
1	39.0	96.20
2	40.6	3.80
Total		100

GC (30.0 m CycloSil-B, injection temperature: 220 °C, 75 °C iso 44 min, 20 °C/min, 220 °C iso 5 min, 0.5 bar He)

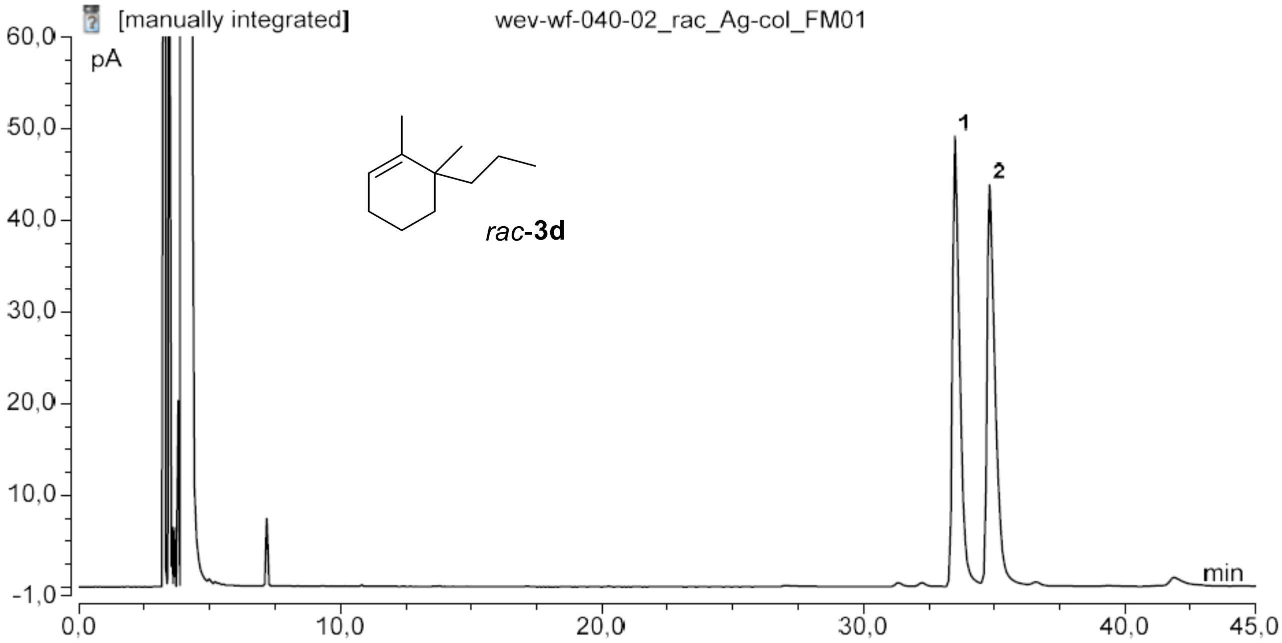

Peak #	t_R (min)	Area (%)
1	33.5	49.92
2	34.8	50.08
Total		100

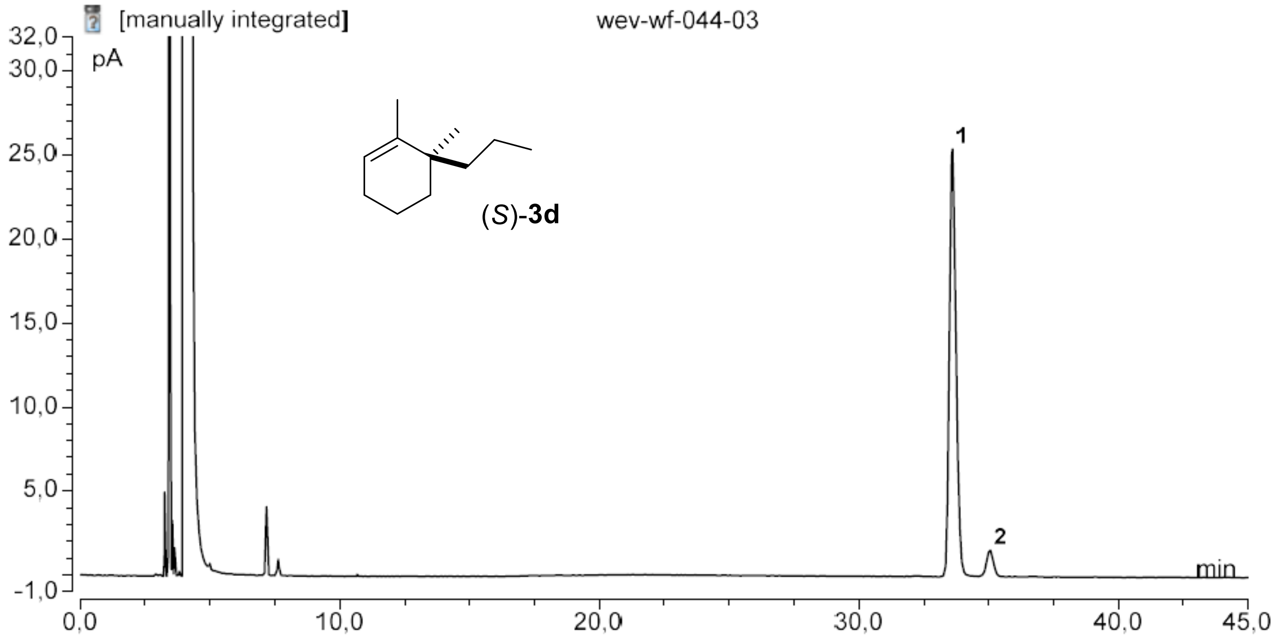

Peak #	t_R (min)	Area (%)
1	33.6	94.04
2	35.0	5.96
Total		100

GC (30.0 m Ivadex-1, injection temperature: 220 °C, 30–50 °C (0.5 °C/min) iso 10 min, 20 °C/min, 220 °C iso 5 min, 0.5 bar He)

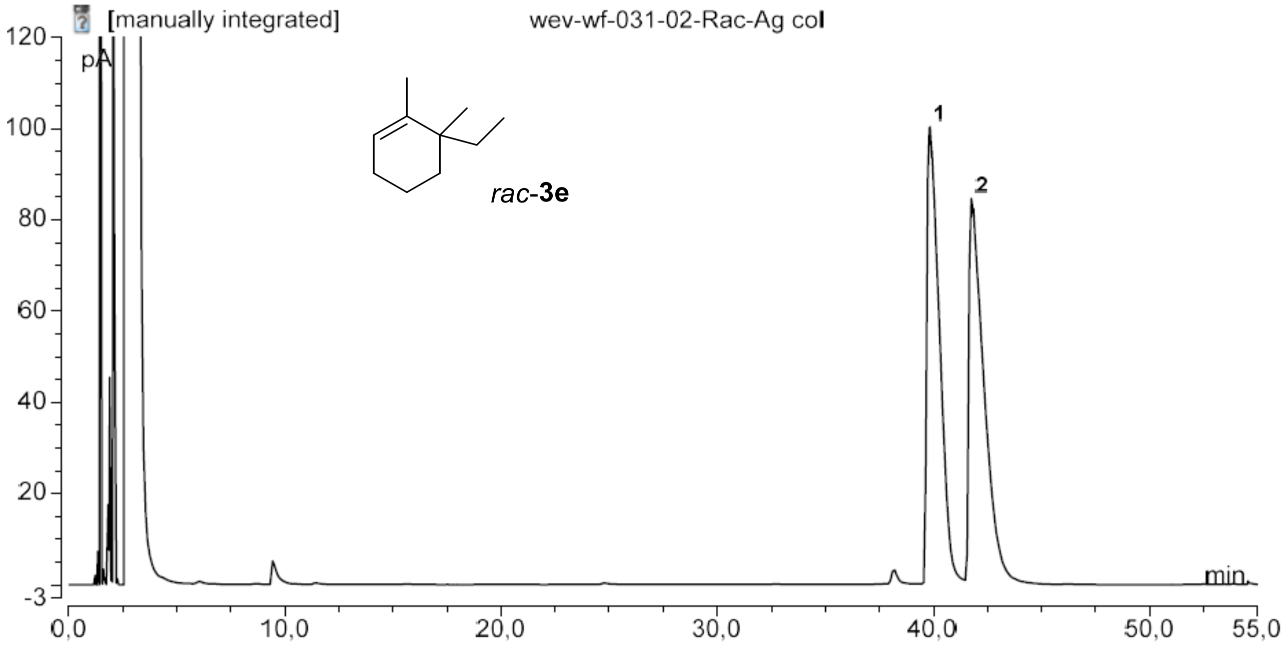

Peak #	t_R (min)	Area (%)
1	39.8	50.18
2	41.8	49.82
Total		100

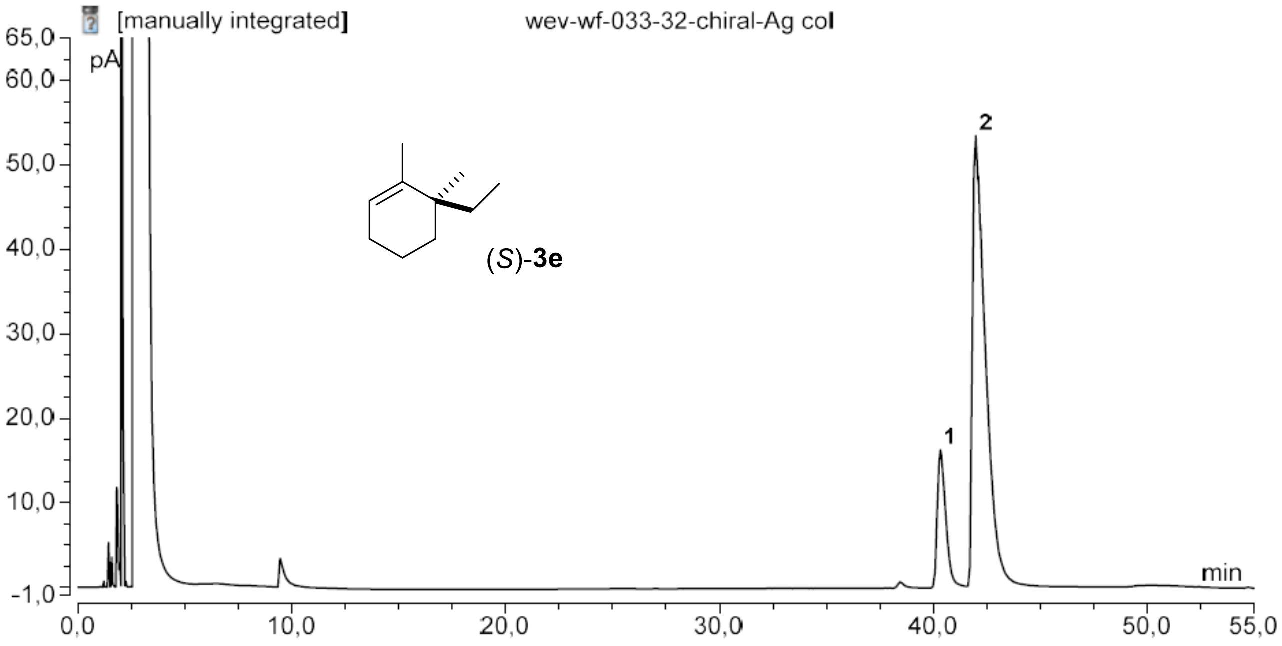

Peak #	t_R (min)	Area (%)
1	40.3	15.90
2	42.0	84.10
Total		100

GC (30.0 m CycloSil-B, injection temperature: 220 °C, 90 °C iso 50 min, 20 °C/min, 220 °C iso 5 min, 0.5 bar He)

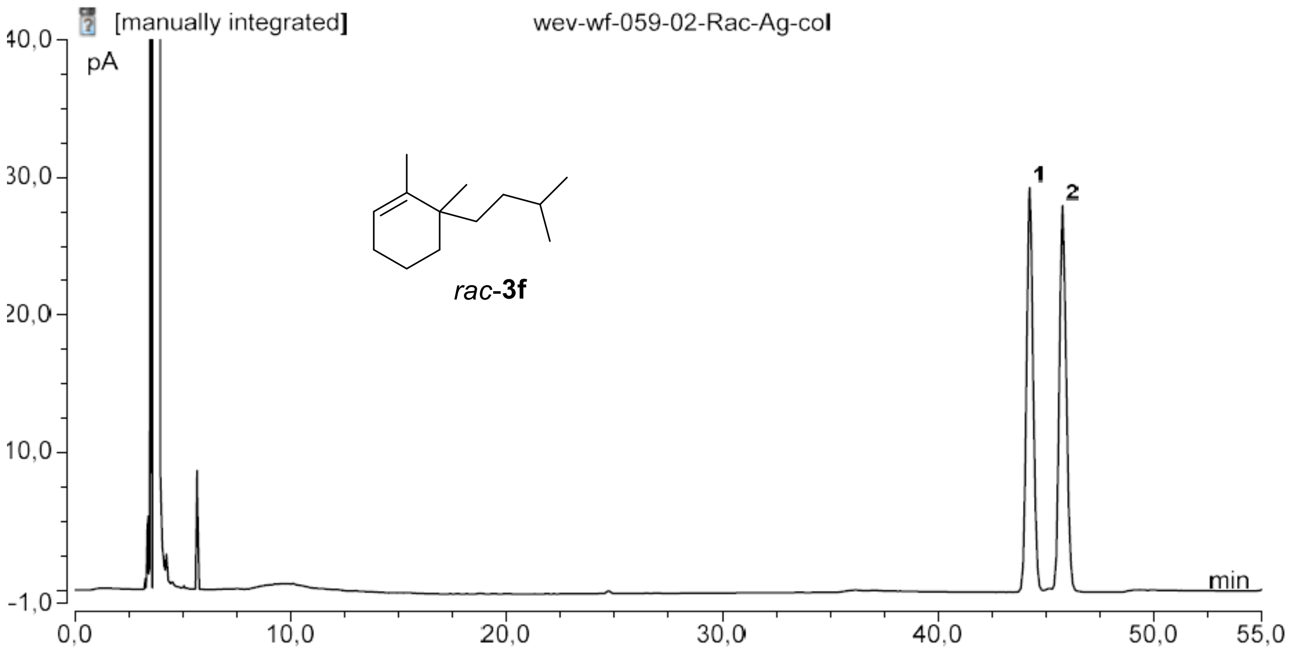

Peak #	t_R (min)	Area (%)
1	44.2	50.17
2	45.8	49.83
Total		100

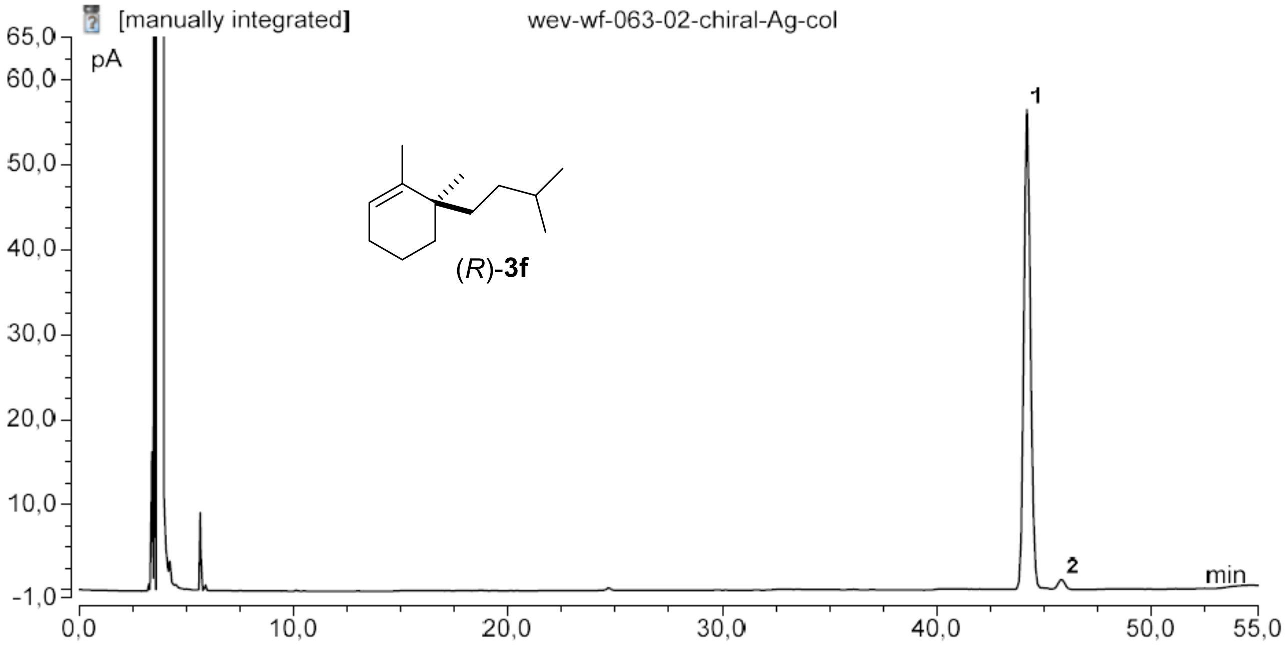

Peak #	t_R (min)	Area (%)
1	44.2	97.98
2	45.9	2.02
Total		100

GC (30.0 m BGB-176, injection temperature: 220 °C, 100 °C iso 30 min, 20 °C/min, 220 °C iso 5 min, 0.5 bar He)

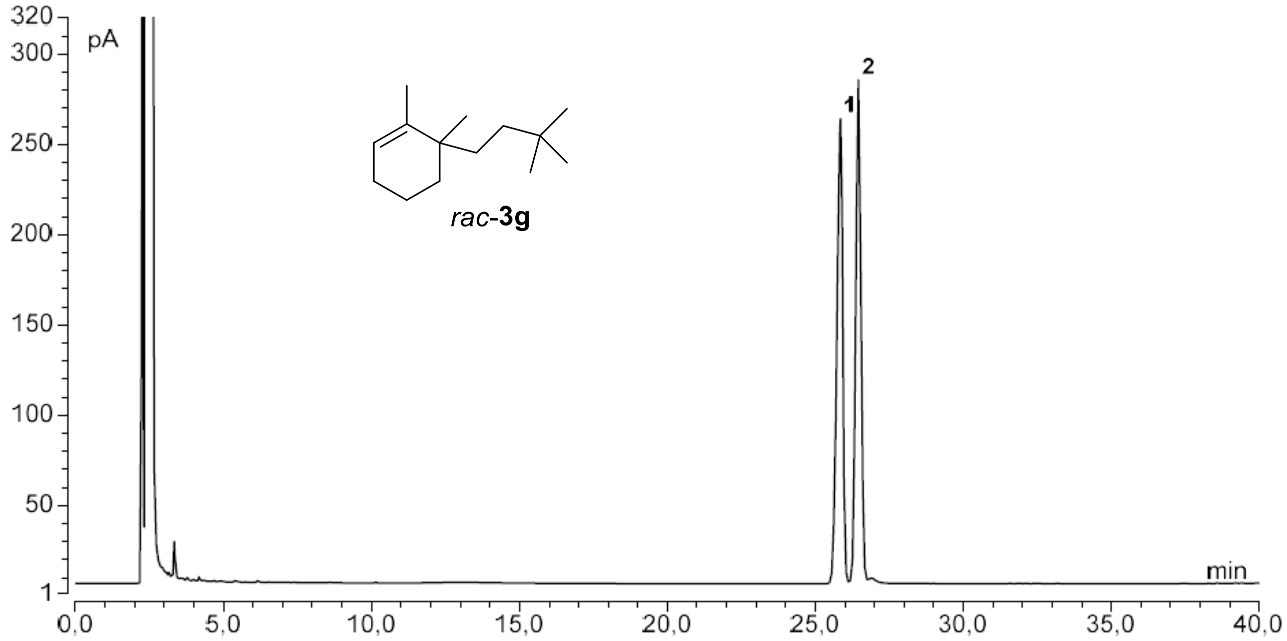

Peak #	t_R (min)	Area (%)
1	25.8	50.46
2	26.4	49.54
Total		100

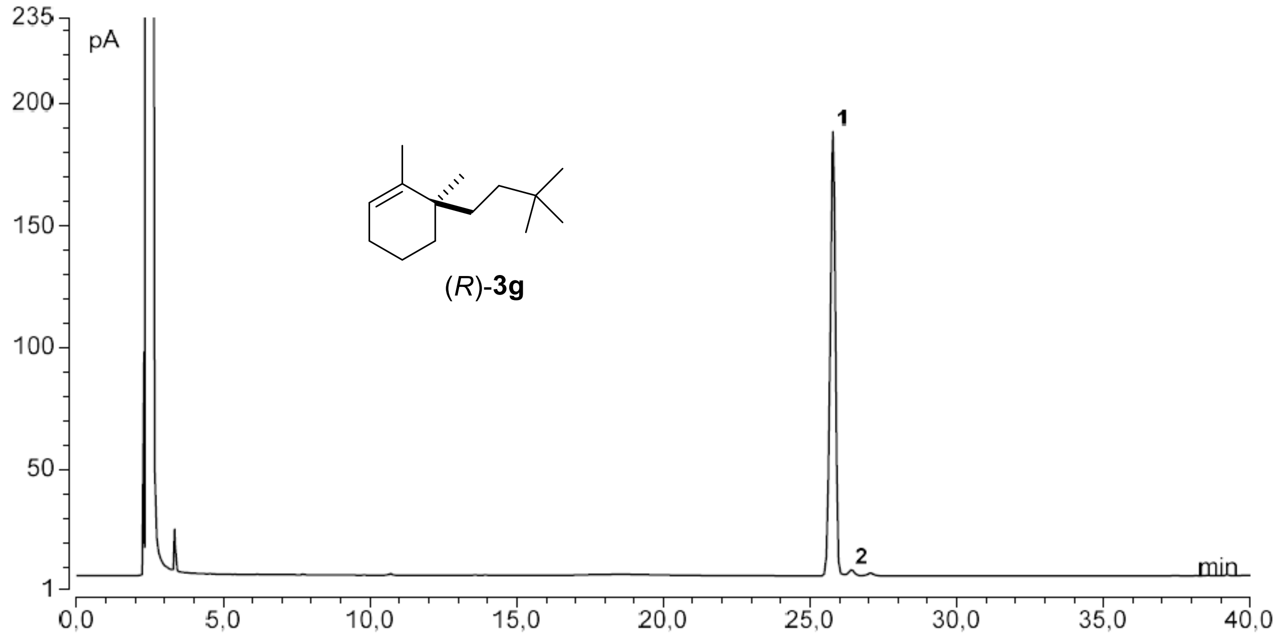

Peak #	t_R (min)	Area (%)
1	25.8	98.90
2	26.4	1.10
Total		100

GC (25.0 m Ivadex-1, injection temperature: 220 °C, 80 °C iso 325 min, 8 °C/min, 220 °C iso 3 min, 0.5 bar H_2)

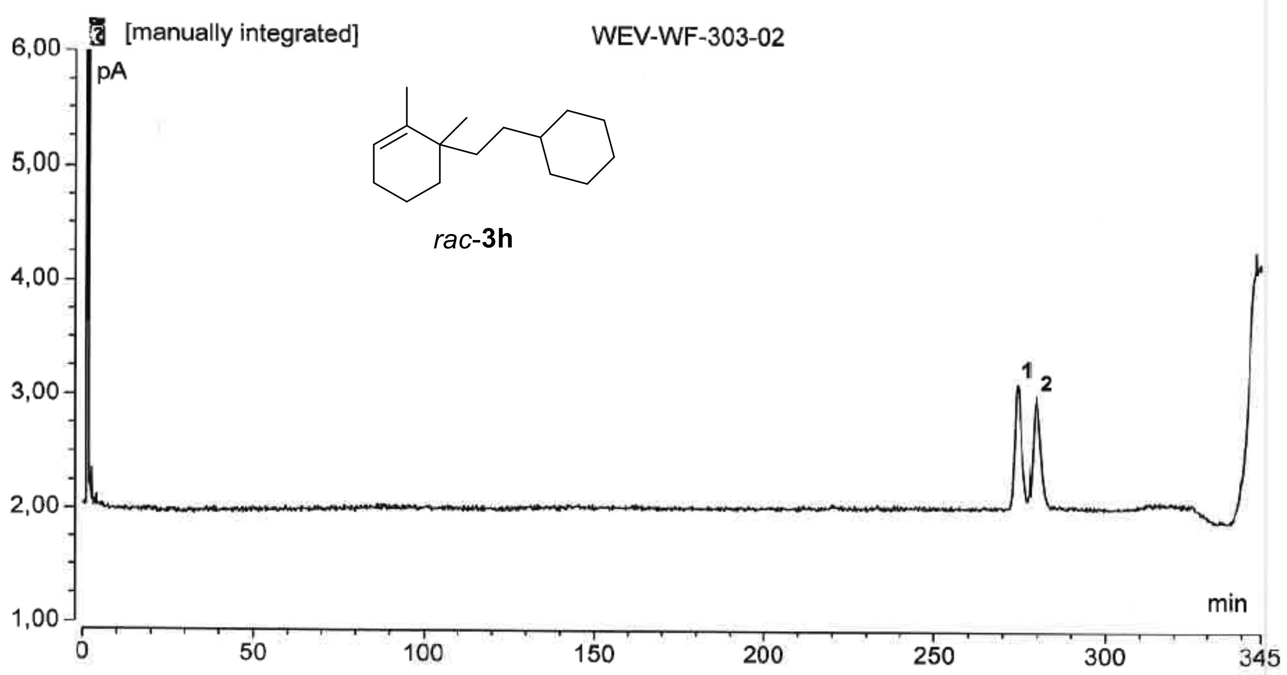

Peak #	t_R (min)	Area (%)
1	274.5	49.91
2	279.6	50.09
Total		100

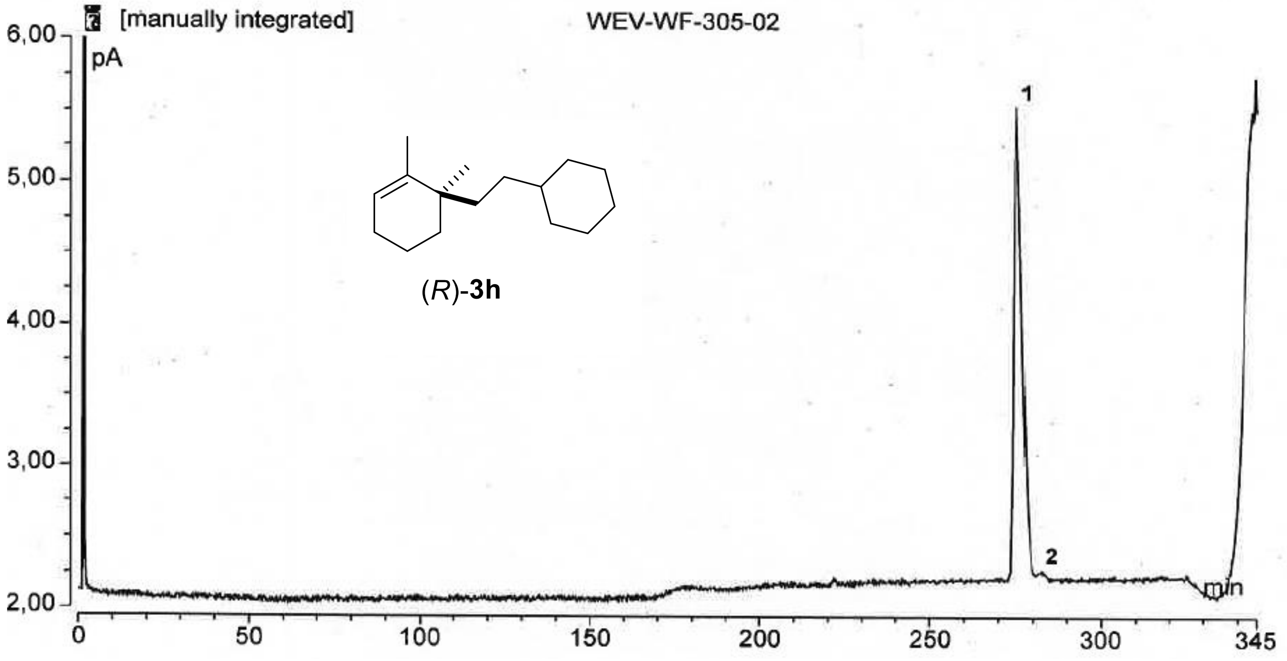

Peak #	t_R (min)	Area (%)
1	275.2	98.40
2	282.4	1.60
Total		100

GC (30.0 m CycloSil-B, injection temperature: 220 °C, 100 °C iso 100 min, 20 °C/min, 220 °C iso 5 min, 0.5 bar He)

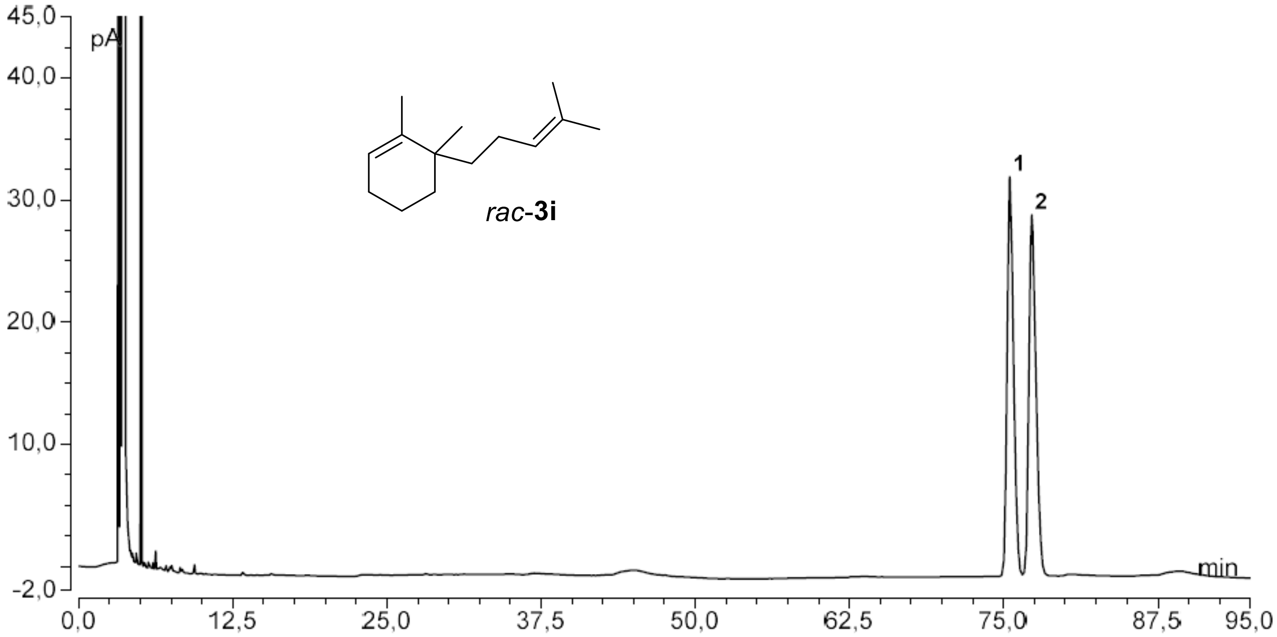

Peak #	t_R (min)	Area (%)
1	75.5	50.37
2	77.3	49.63
Total		100

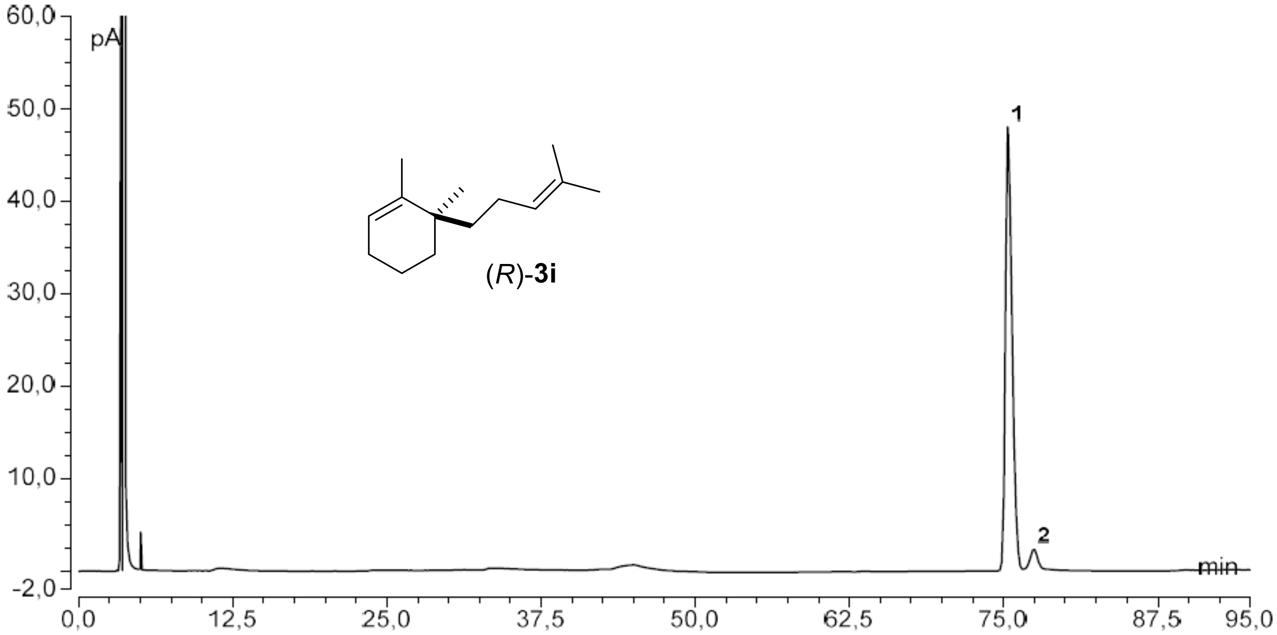

Peak #	t_R (min)	Area (%)
1	75.3	95.35
2	77.4	4.65
Total		100

GC (21.5 m Ivadex-5, injection temperature: 220 °C, 120 °C iso 900 min, 8 °C/min, 220 °C iso 3 min, 0.4 bar H_2)

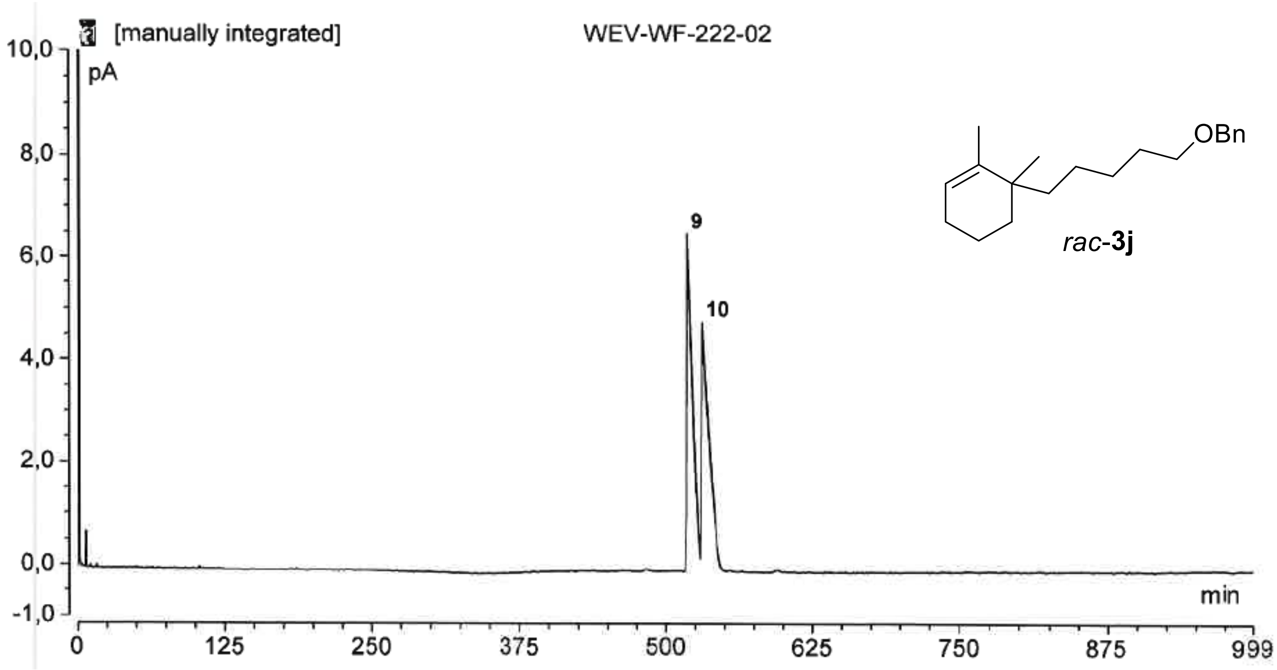

Peak #	t_R (min)	Area (%)
9	518.9	49.94
10	531.6	50.06
Total		100

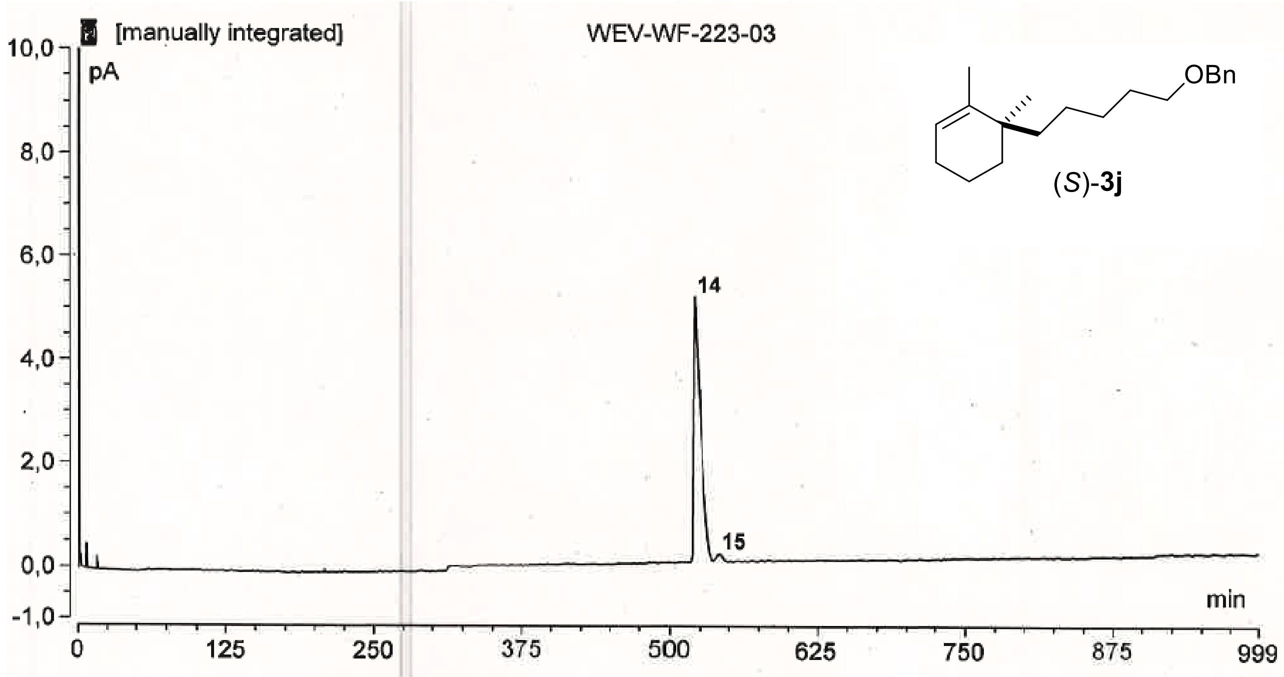

Peak #	t_R (min)	Area (%)
14	521.2	97.95
15	541.7	2.05
Total		100

GC (25.0 m Ivadex-1, injection temperature: 220 °C, 100 °C iso 710 min, 8 °C/min, 220 °C iso 3 min, 0.5 bar H_2)

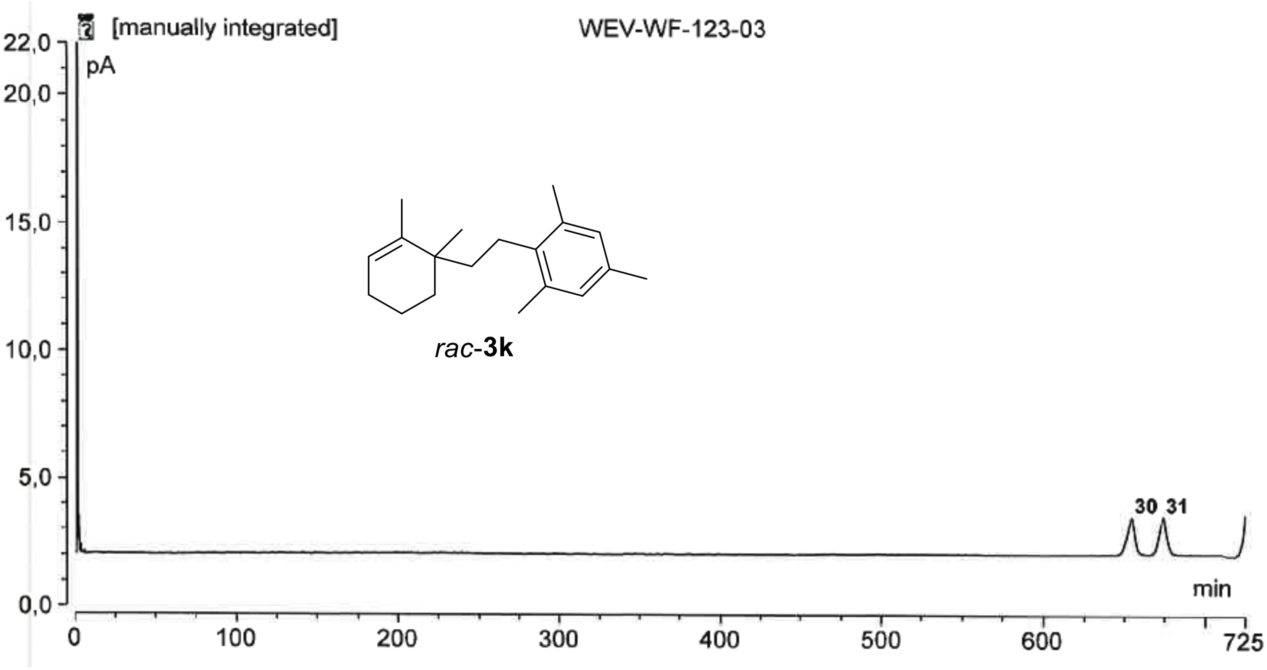

Peak #	t_R (min)	Area (%)
30	654.4	50.05
31	673.9	49.95
Total		100

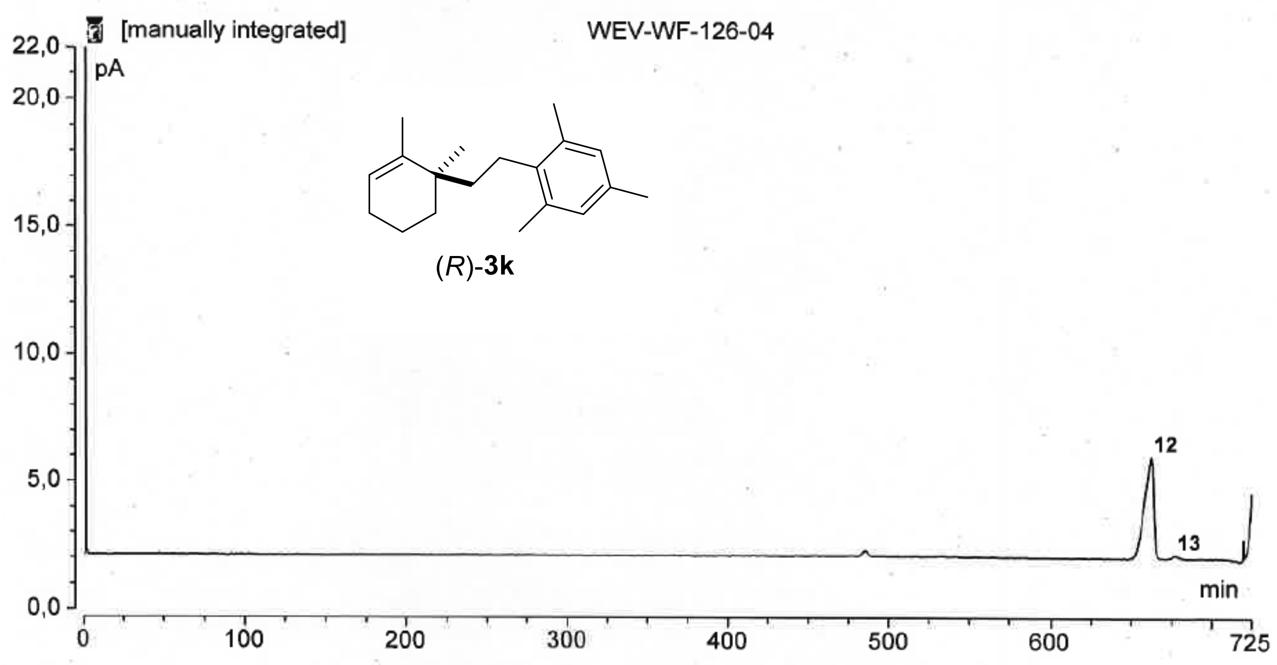

Peak #	t_R (min)	Area (%)
12	662.3	97.18
13	676.9	2.82
Total		100

GC (30.0 m CycloSil-B, injection temperature: 220 °C, 90 °C iso 65 min, 20 °C/min, 220 °C iso 10 min, 0.5 bar He)

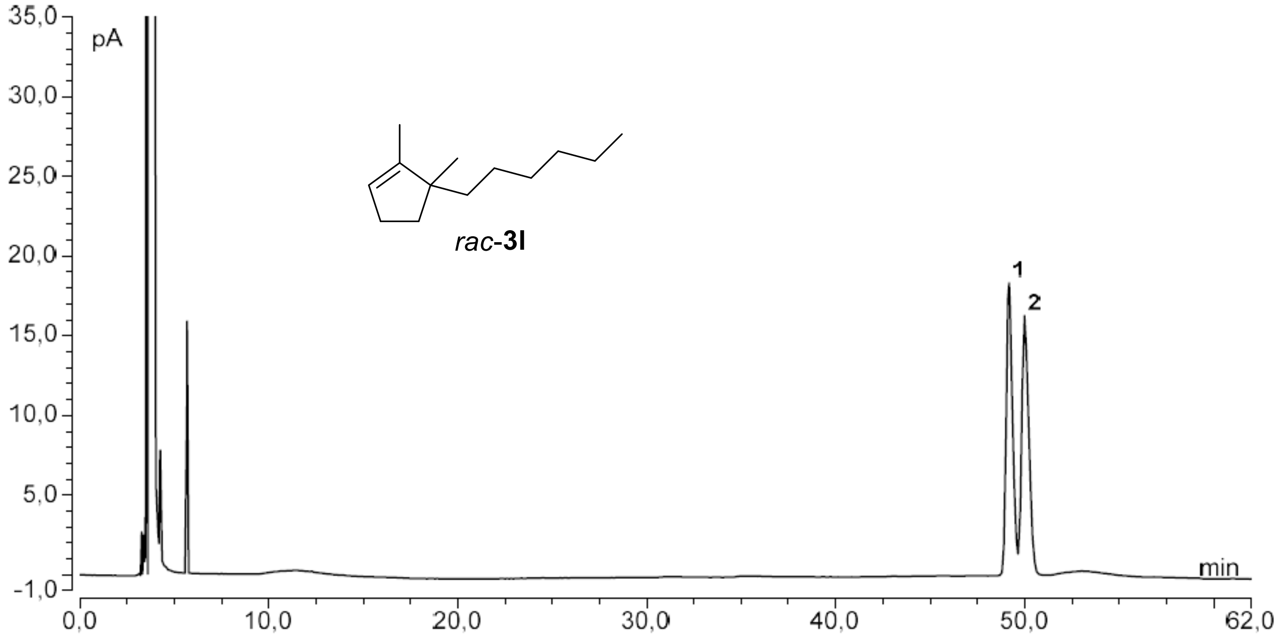

Peak #	t_R (min)	Area (%)
1	49.2	50.01
2	50.0	49.99
Total		100

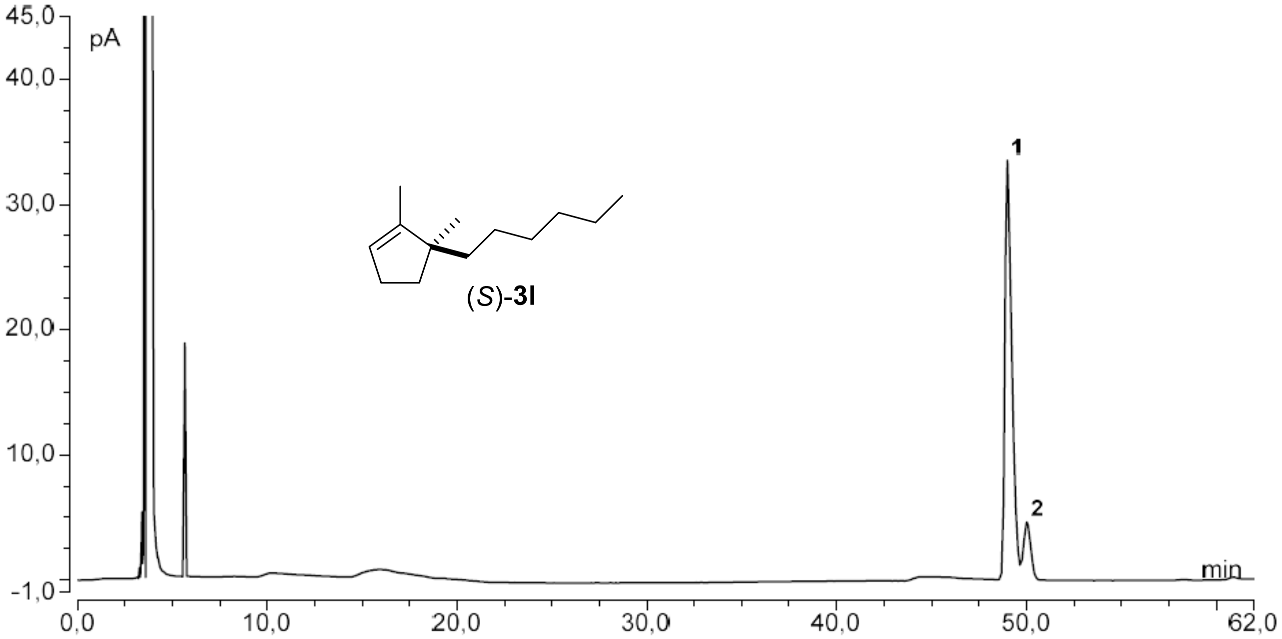

Peak #	t_R (min)	Area (%)
1	49.0	90.07
2	50.0	9.93
Total		100

GC (30.0 m CycloSil-B, injection temperature: 220 °C, 70 °C iso 65 min, 20 °C/min, 220 °C iso 10 min, 0.5 bar He)

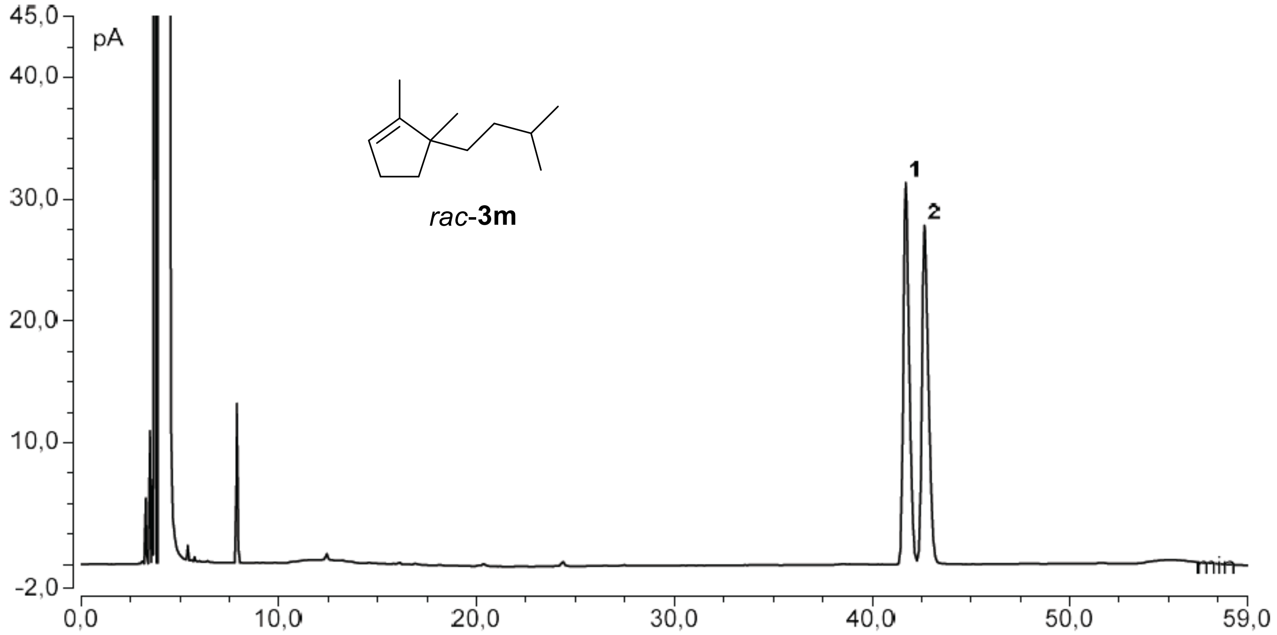

Peak #	t_R (min)	Area (%)
1	41.7	50.91
2	42.6	49.09
Total		100

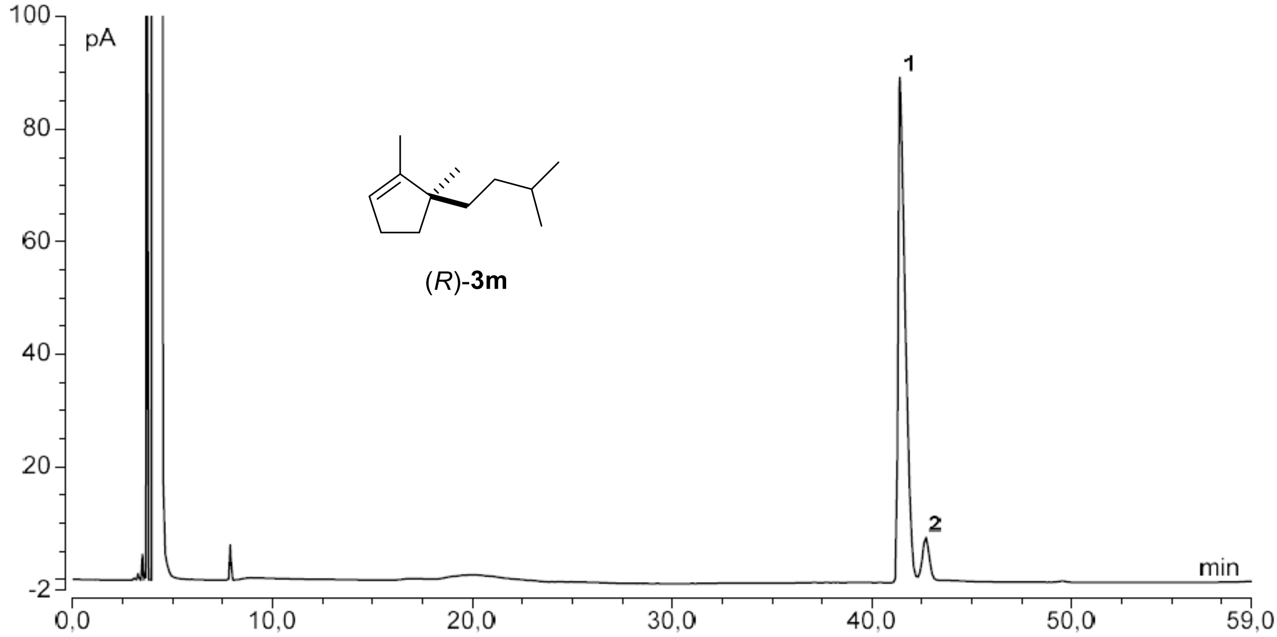

Peak #	t_R (min)	Area (%)
1	41.4	92.82
2	42.7	7.18
Total		100

GC (30.0 m CycloSil-B, injection temperature: 220 °C, 70 °C iso 75 min, 20 °C/min, 220 °C iso 10 min, 0.5 bar He)

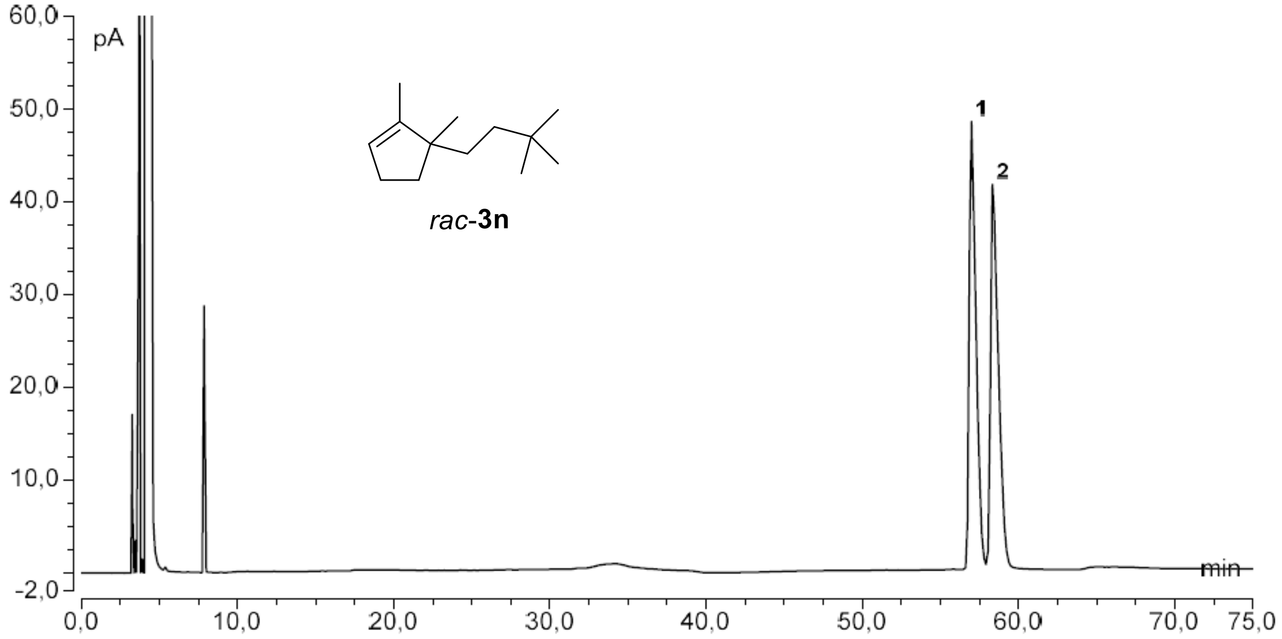

Peak #	t_R (min)	Area (%)
1	57.0	50.31
2	58.3	49.69
Total		100

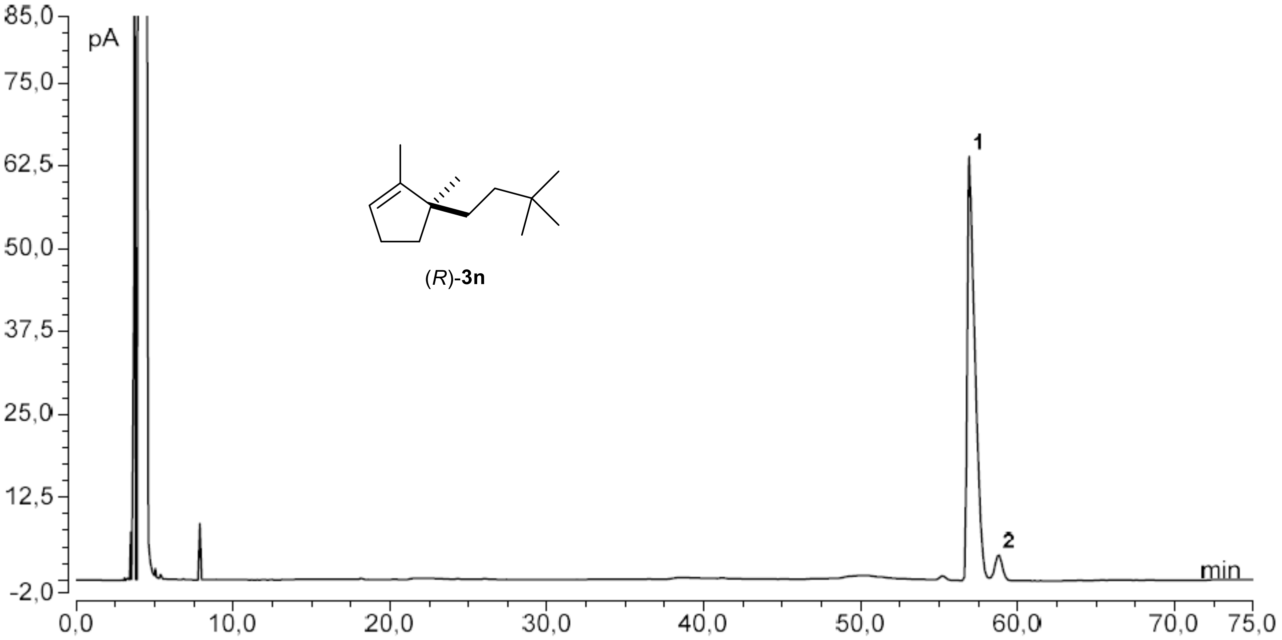

Peak #	t_R (min)	Area (%)
1	56.9	94.92
2	58.8	5.08
Total		100

GC (30.0 m BGB-176, injection temperature: 220 °C, 80–125°C (0.7 °C/min) iso 20 min, 20 °C/min, 220 °C iso 5 min,, 0.5 bar He)

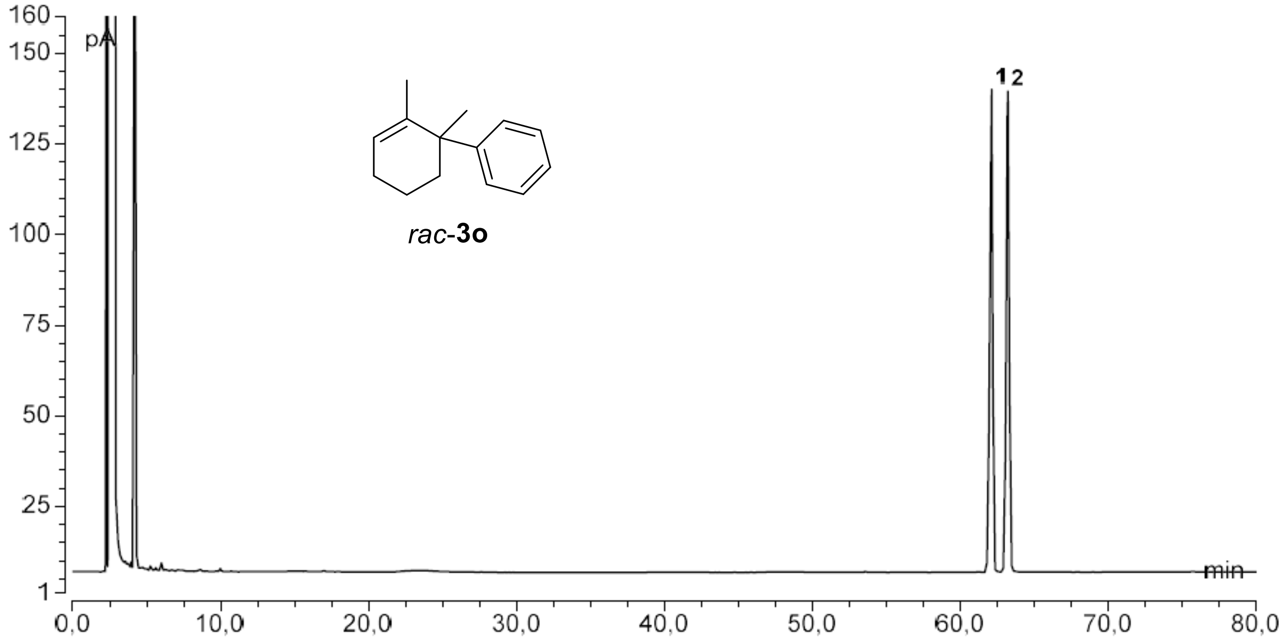

Peak #	t_R (min)	Area (%)
1	62.1	50.42
2	63.2	49.58
Total		100

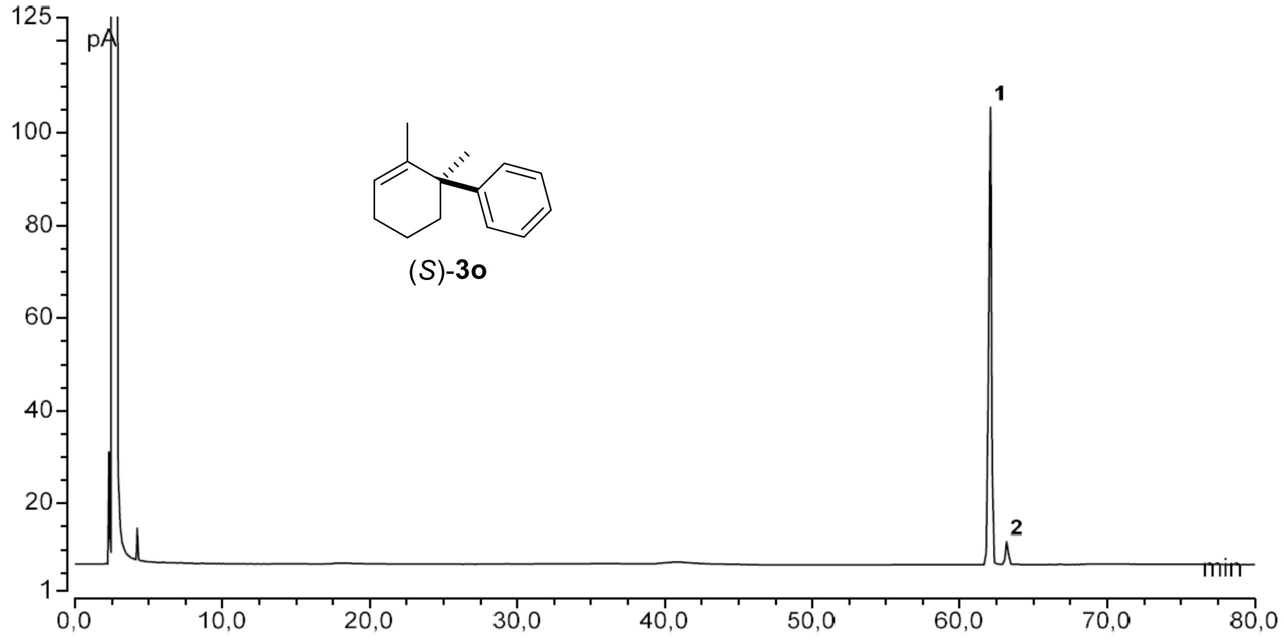

Peak #	t_R (min)	Area (%)
1	62.1	95.25
2	63.2	4.75
Total		100

GC (30.0 m BGB-176, injection temperature: 220 °C, 130 °C iso 90 min, 20 °C/min, 220 °C iso 10 min, 0.5 bar He).

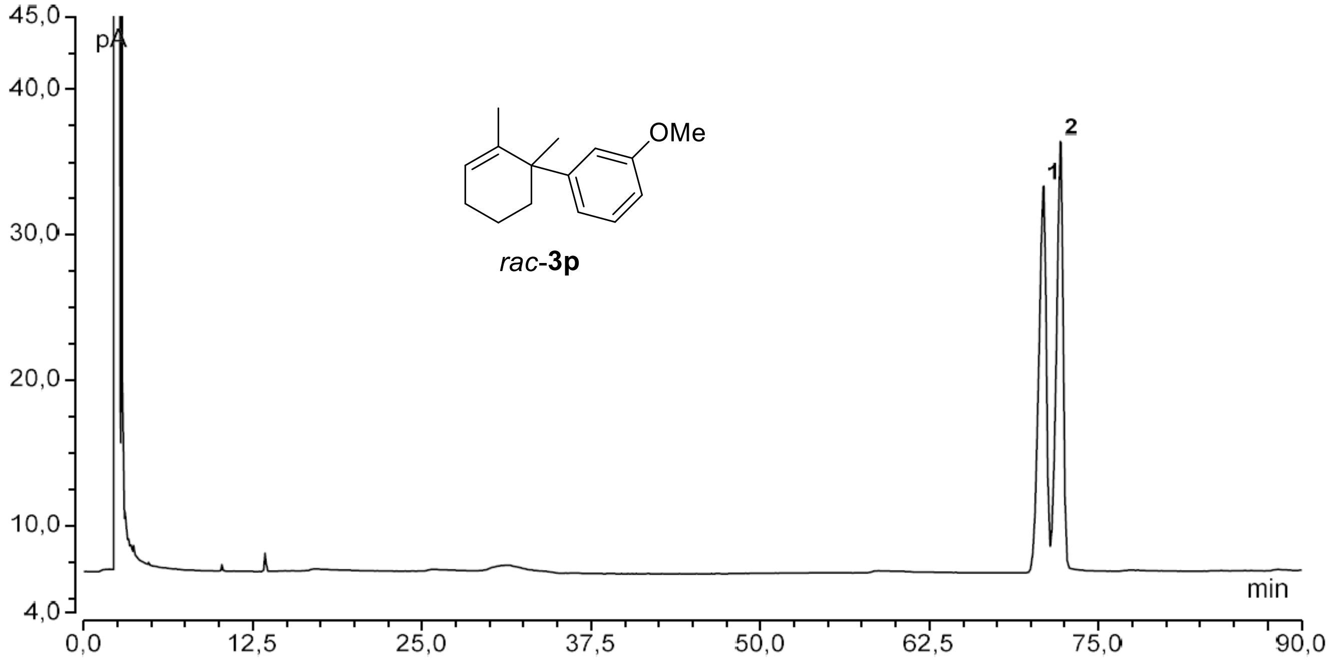

Peak #	t_R (min)	Area (%)
1	70.94	50.08
2	72.19	49.92
Total		100

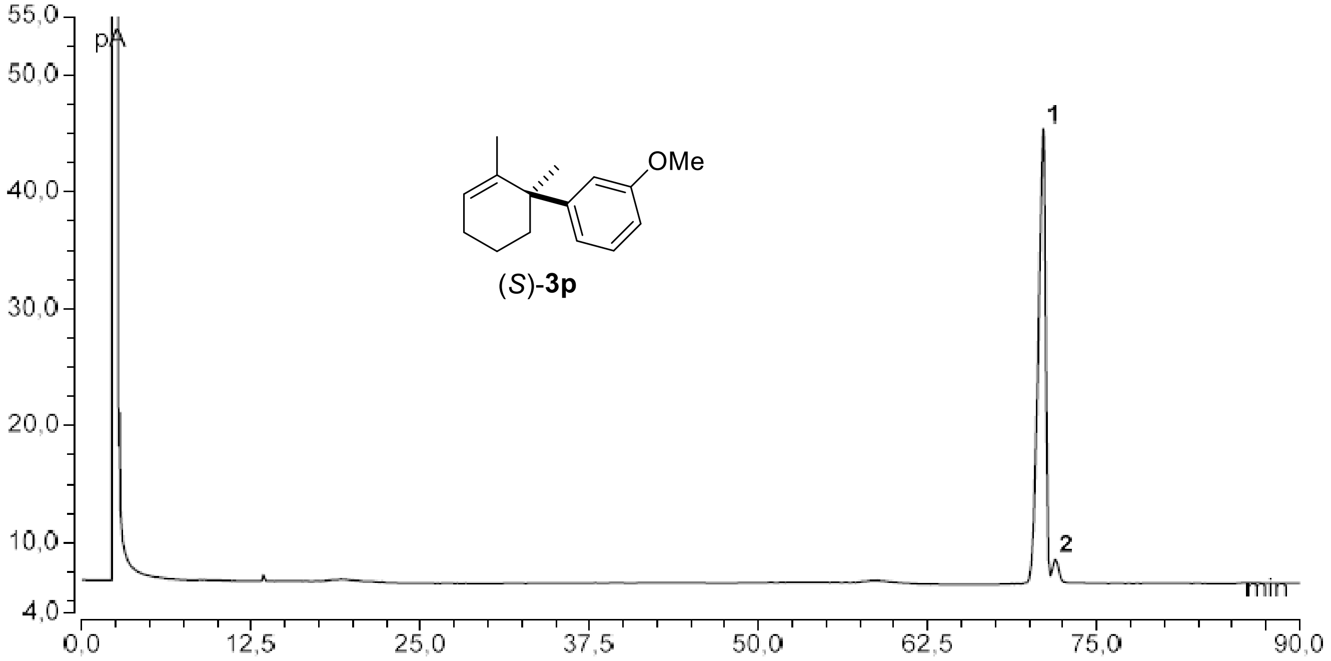

Peak #	t_R (min)	Area (%)
1	71.04	97.03
2	71.94	2.97
Total		100

GC (30.0 m BGB-176, injection temperature: 220 °C, 115 °C iso 55 min, 20 °C/min, 220 °C iso 10 min, 0.5 bar He).

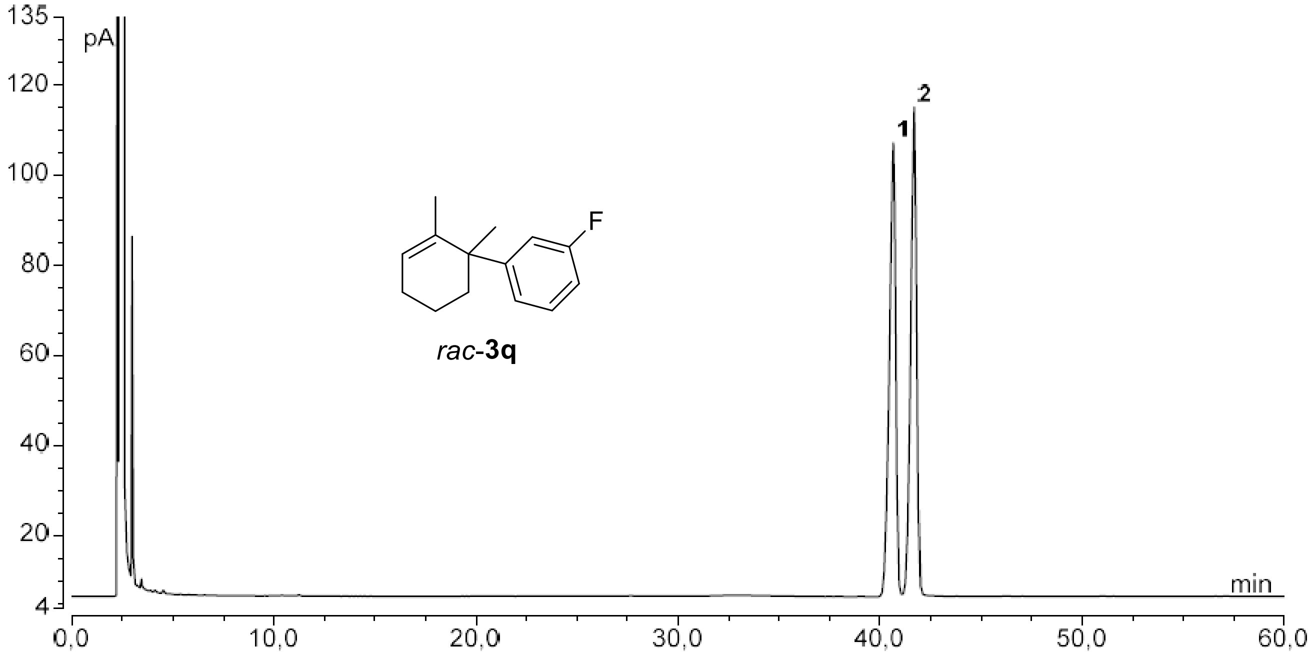

Peak #	t_R (min)	Area (%)
1	40.68	50.29
2	41.70	49.71
Total		100

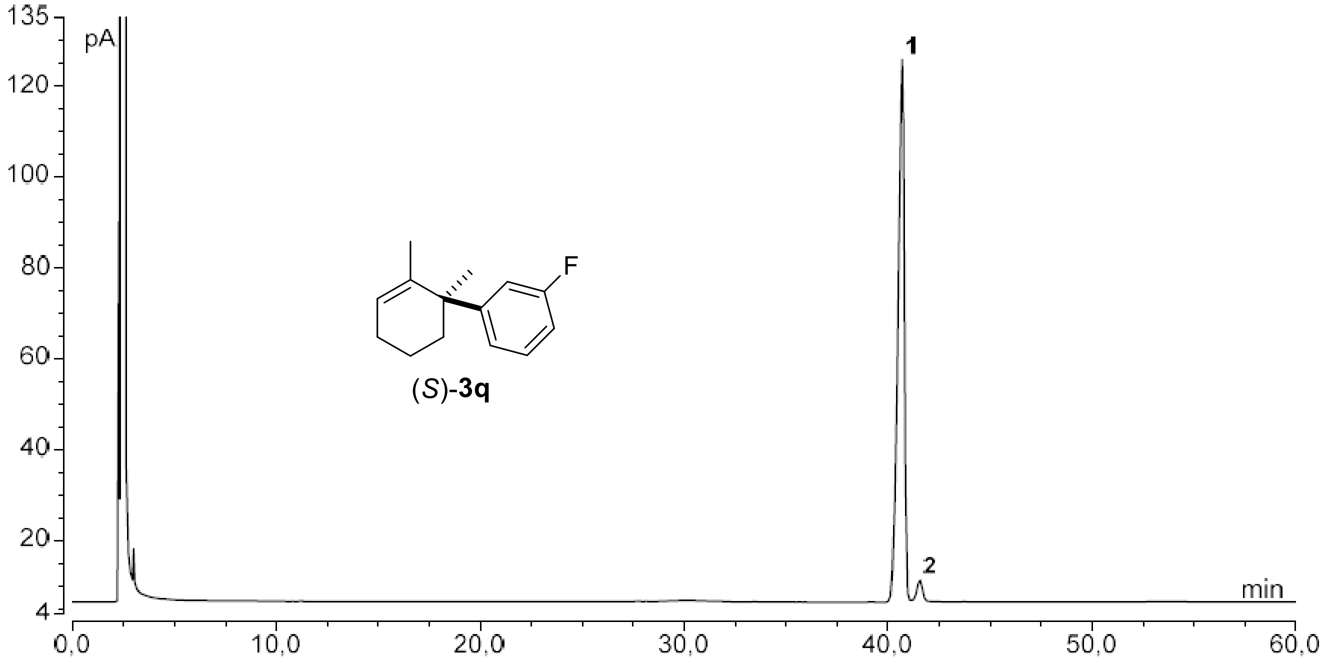

Peak #	t_R (min)	Area (%)
1	40.69	96.85
2	41.55	3.15
Total		100

GC (30.0 m BGB-176, injection temperature: 220 °C, 130 °C iso 45 min, 20 °C/min, 220 °C iso 10 min, 0.5 bar He).

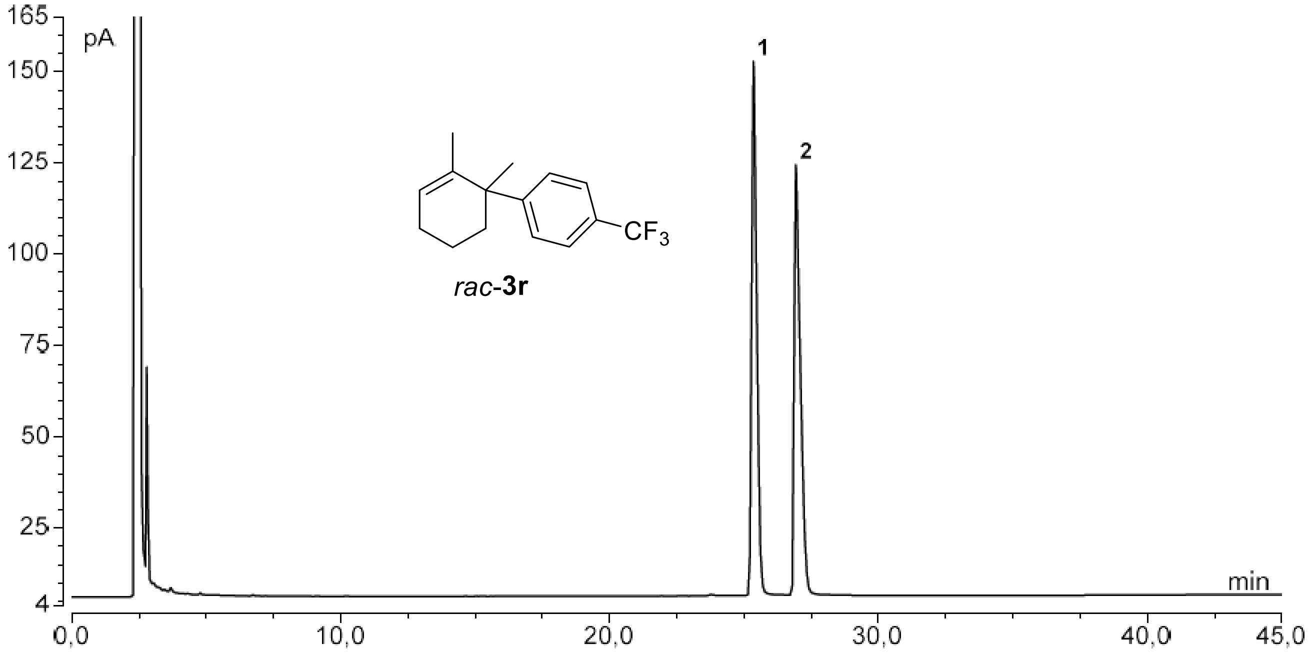

Peak #	t_R (min)	Area (%)
1	25.36	50.21
2	26.93	49.79
Total		100

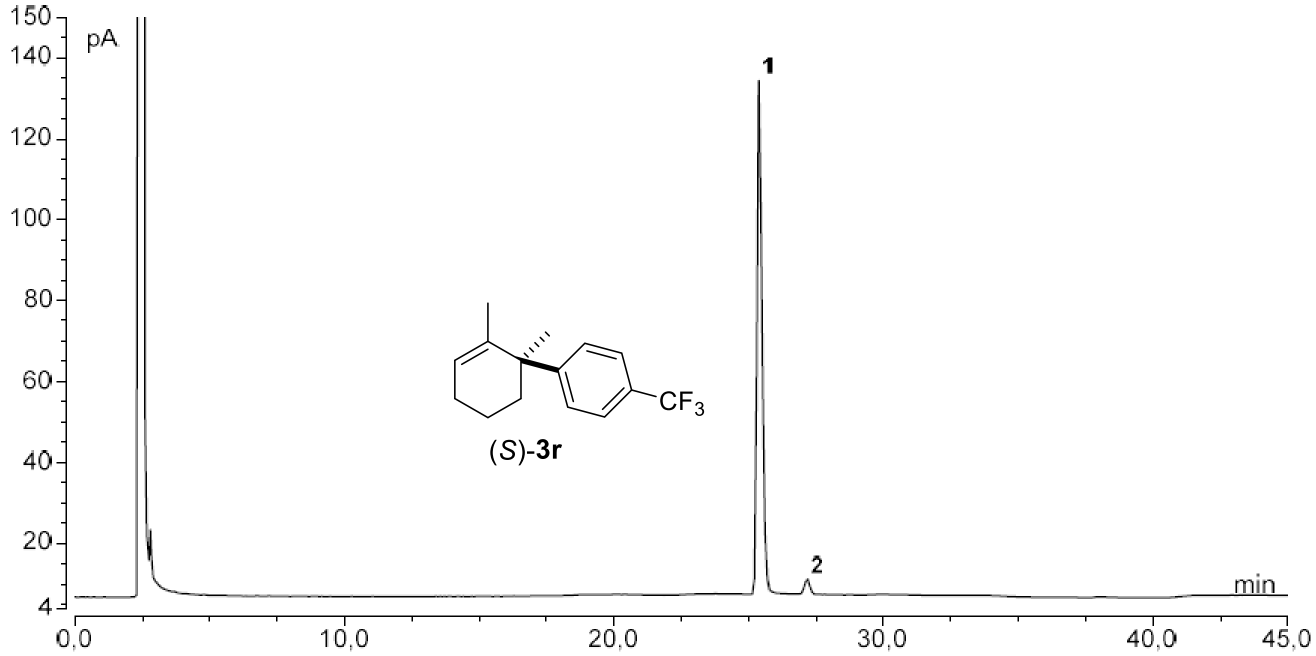

Peak #	t_R (min)	Area (%)
1	25.39	96.95
2	27.16	3.05
Total		100

GC (30.0 m CycloSil-B, injection temperature: 220 °C, 110 °C iso 50 min, 20 °C/min, 220 °C iso 10 min, 0.5 bar H_2).

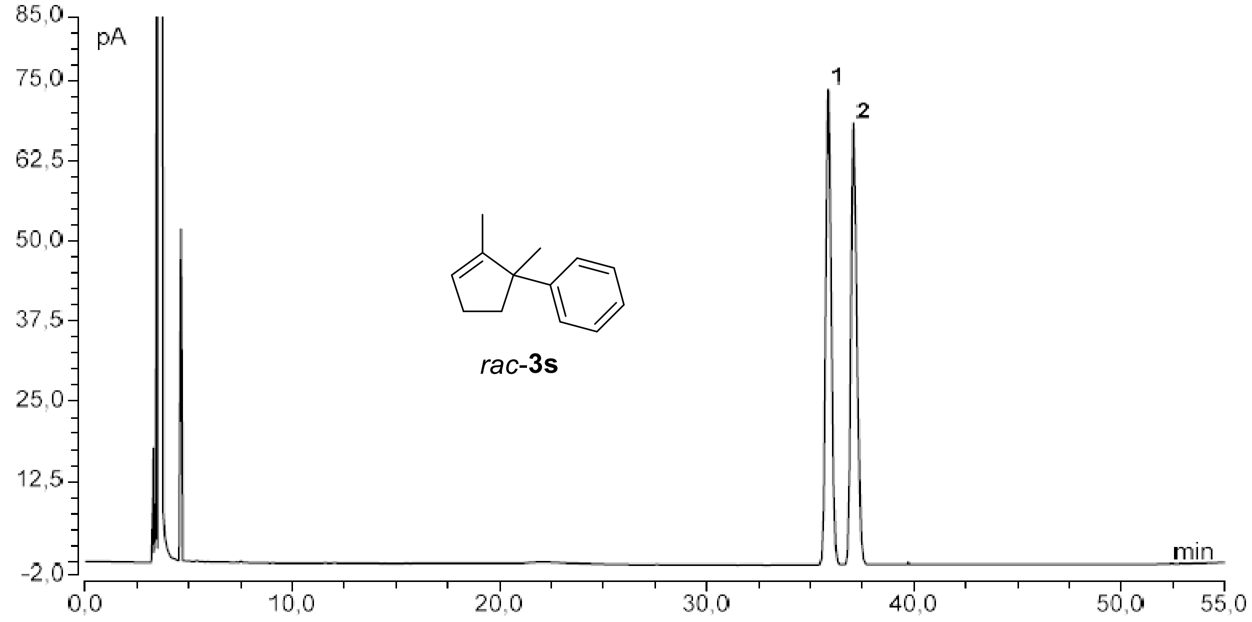

Peak #	t_R (min)	Area (%)
1	35.87	50.03
2	37.07	49.97
Total		100

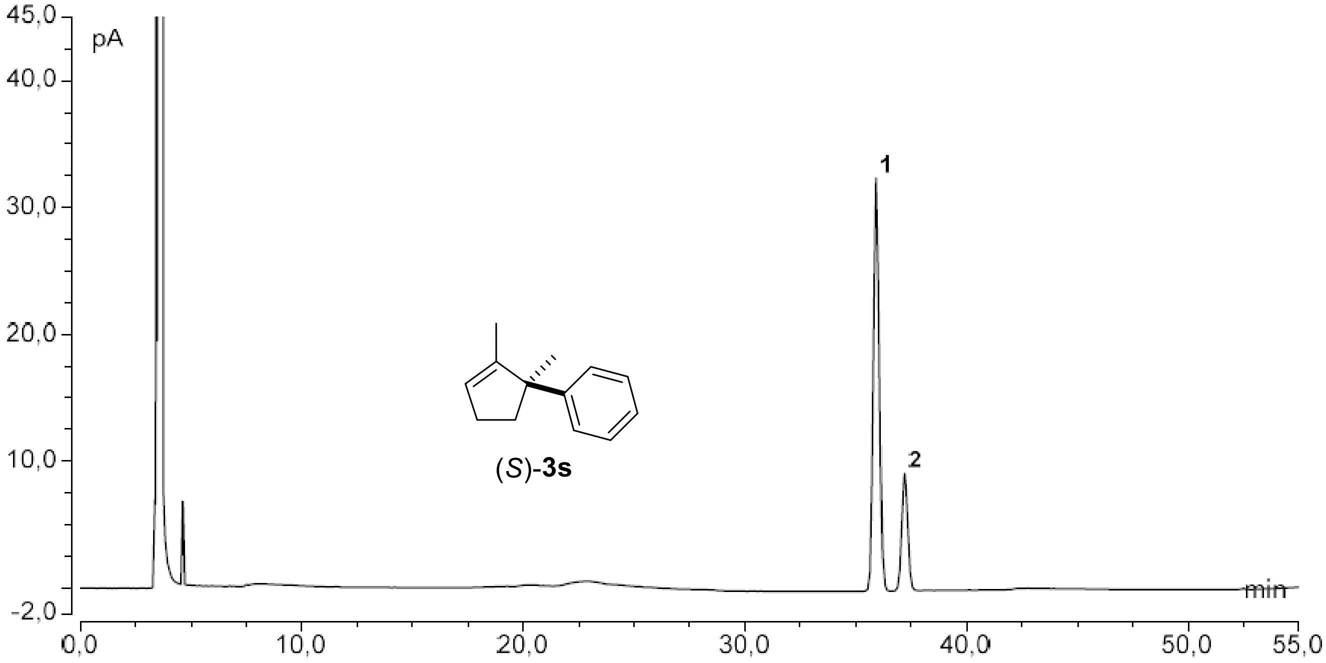

Peak #	t_R (min)	Area (%)
1	35.93	77.18
2	37.22	22.82
Total		100

GC (30.0 m CycloSil-B, injection temperature: 220 °C, 115 °C iso 60 min, 20 °C/min, 220 °C iso 10 min, 0.5 bar H_2).

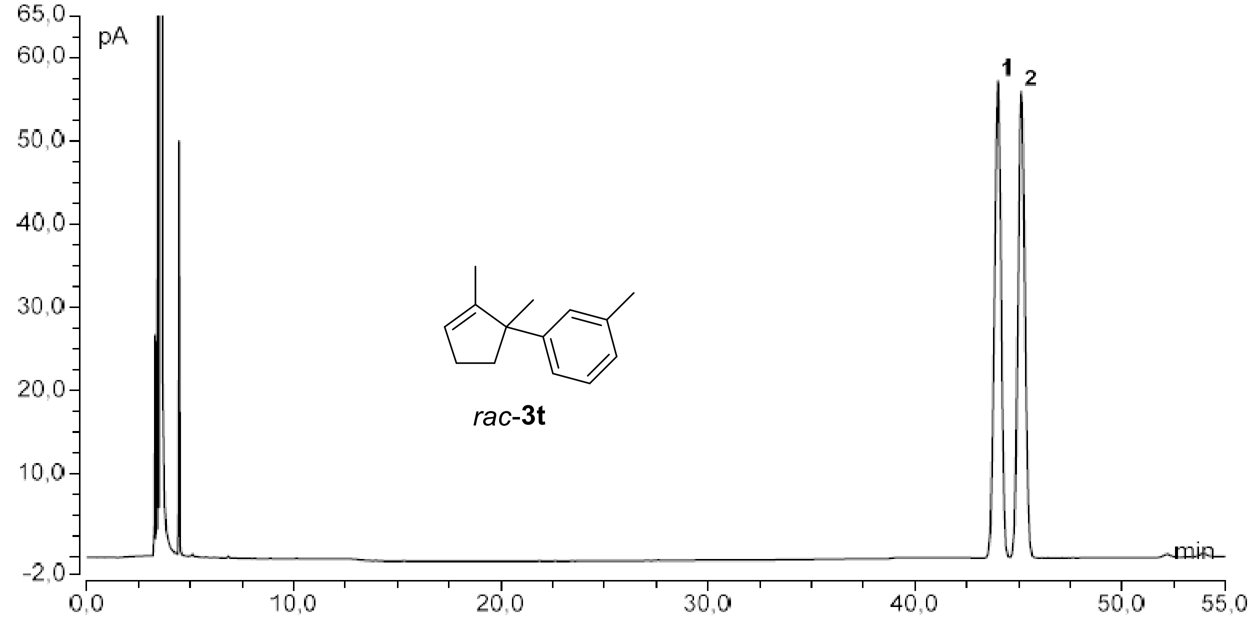

Peak #	t_R (min)	Area (%)
1	44.01	49.98
2	45.13	50.02
Total		100

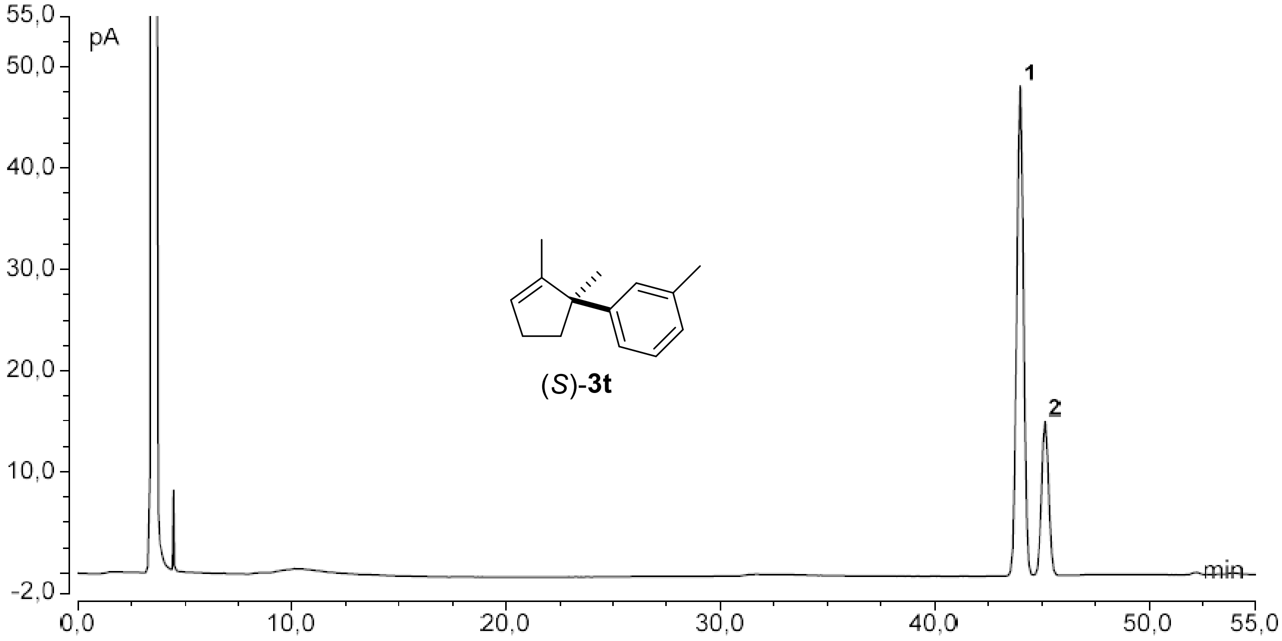

Peak #	t_R (min)	Area (%)
1	44.01	75.77
2	45.16	24.23
Total		100

GC (24.0 m Cyclodextrin-H, injection temperature: 220 °C, 90 °C iso 35 min, 8 °C/min, 180 °C iso 10 min, 0.5 bar H_2).

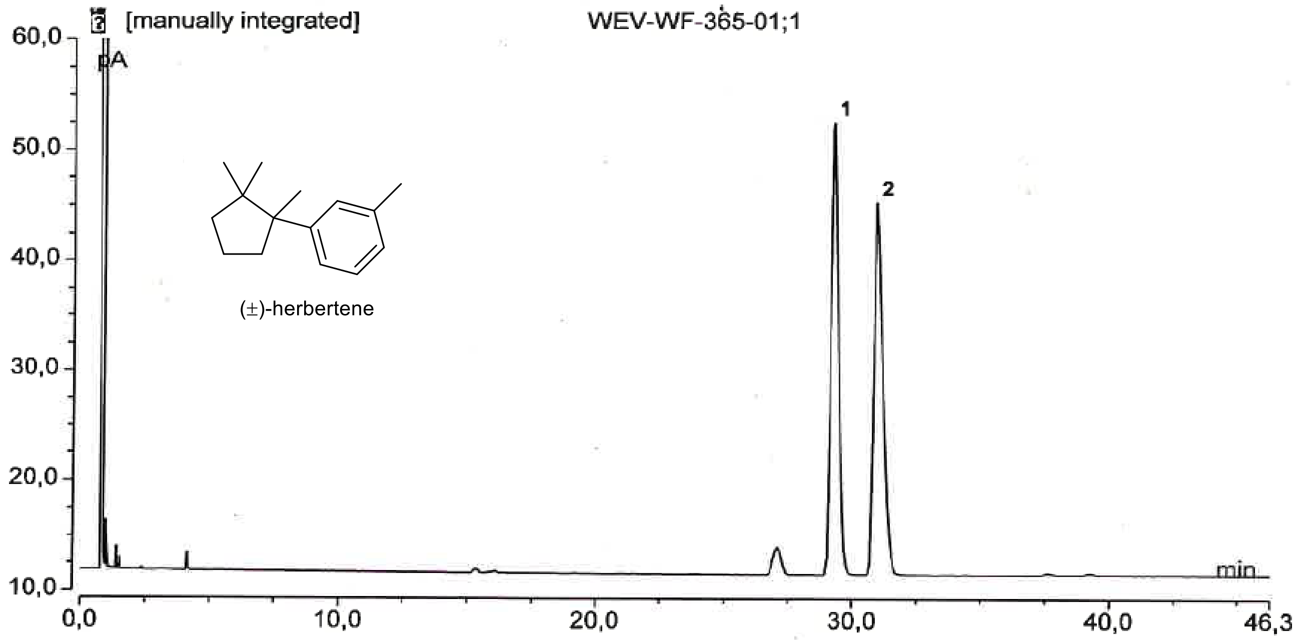

Peak #	t_R (min)	Area (%)
1	29,20	50.02
2	30.85	49.98
Total		100

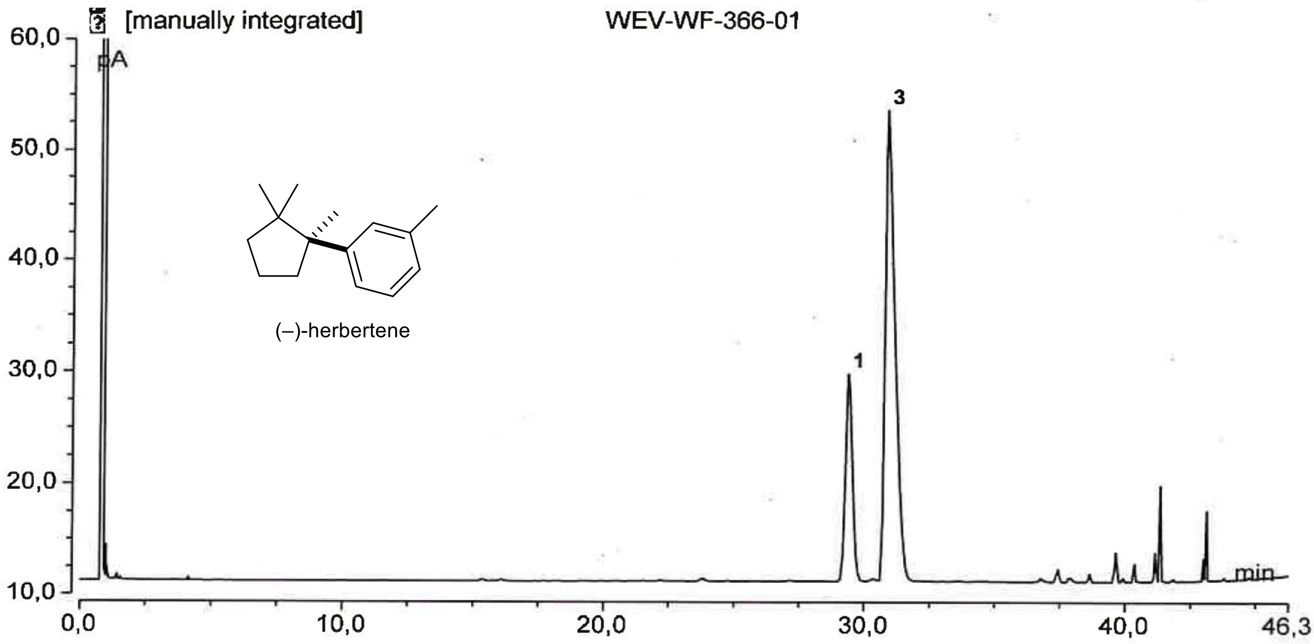

Peak #	t_R (min)	Area (%)
1	29.36	23.86
2	30.80	76.14
Total		100

11. References

1 Schwengers, S. A. et al. Unified Approach to Imidodiphosphate-Type Brønsted Acids with Tunable Confinement and Acidity. *J. Am. Chem. Soc.* **143**, 14835–14844 (2021).

2 Liu, Y., Peng, X., She, R., Zhou, X. & Peng, Y. Catalytic Asymmetric (3 + 3) Cycloaddition of Oxyallyl Zwitterions with α-Diazomethylphosphonates. *Org. Lett.* **23**, 7295–7300 (2021).

3 Lee, S., Bae, H. Y. & List, B. Can a Ketone Be More Reactive than an Aldehyde? Catalytic Asymmetric Synthesis of Substituted Tetrahydrofurans. *Angew. Chem., Int. Ed.* **57**, 12162–12166 (2018).

4 Zhou, H. et al. Organocatalytic DYKAT of Si-Stereogenic Silanes. *J. Am. Chem. Soc.* **145**, 4994–5000 (2023).

5 Maji, R. et al. A Catalytic Asymmetric Hydrolactonization. *J. Am. Chem. Soc.* **145**, 8788–8793 (2023).

6 Lee, S., Kaib, P. S. J. & List, B. Asymmetric Catalysis via Cyclic, Aliphatic Oxocarbenium Ions. *J. Am. Chem. Soc.* **139**, 2156–2159 (2017).

7 Ye, B., Zhao, J., Zhao, K., McKenna, J. M. & Toste, F. D. Chiral Diaryliodonium Phosphate Enables Light Driven Diastereoselective α-C(sp3)–H Acetalization. *J. Am. Chem. Soc.* **140**, 8350–8356 (2018).

8 Yue, Y., Turlington, M., Yu, X.-Q. & Pu, L. 3,3′-Anisyl-Substituted BINOL, H4BINOL, and H8BINOL Ligands: Asymmetric Synthesis of Diverse Propargylic Alcohols and Their Ring-Closing Metathesis to Chiral Cycloalkenes. *J. Org. Chem.* **74**, 8681–8689 (2009).

9 Properzi, R. et al. Catalytic enantiocontrol over a non-classical carbocation. *Nat. Chem.* **12**, 1174–1179 (2020).

10 Yan, J.-X. et al. Palladium-Catalyzed C(sp^3)-H Activation: A Facile Method for the Synthesis of 3,4-Dihydroquinolinone Derivatives. *Angew. Chem., Int. Ed.* **53**, 4945–4949 (2014).

11 Houston, S. D. et al. Cyclooctatetraenes through Valence Isomerization of Cubanes: Scope and Limitations. *Chem. Eur. J.* **25**, 2735–2739 (2019).

12 Ranu, B. C. & Jana, U. A New Redundant Rearrangement of Aromatic Ring Fused Cyclic α-Hydroxydithiane Derivatives. Synthesis of Aromatic Ring Fused Cyclic 1,2-Diketones with One-Carbon Ring Expansion. *J. Org. Chem.* **64**, 6380–6386 (1999).

13 Ikeda, S. & Shintani, R. Rhodium-Catalyzed Stitching Polymerization of 1,5-Hexadiynes and Related Oligoalkynes. *Angew. Chem., Int. Ed.* **58**, 5734–5738 (2019).

14 Albright, H. et al. Catalytic Carbonyl-Olefin Metathesis of Aliphatic Ketones: Iron(III) Homo-Dimers as Lewis Acidic Superelectrophiles. *J. Am. Chem. Soc.* **141**, 1690–1700 (2019).

15 Xie, Y.-Y. et al. Lewis Base/Brønsted Acid Co-catalyzed Enantioselective Sulfenylation/Semipinacol Rearrangement of Di- and Trisubstituted Allylic Alcohols. *Angew. Chem., Int. Ed.* **58**, 12491–12496 (2019).

16 Burés, J. A Simple Graphical Method to Determine the Order in Catalyst. *Angew. Chem., Int. Ed.* **55**, 2028–2031 (2016).

17 Burns, A. S., Dooley, C., III, Carlson, P. R., Ziller, J. W. & Rychnovsky, S. D. Relative and Absolute Structure Assignments of Alkenes Using Crystalline Osmate Derivatives for X-ray Analysis. *Org. Lett.* **21**, 10125–10129 (2019).

18 Pracht, P., Bohle, F. & Grimme, S. Automated exploration of the low-energy chemical space with fast quantum chemical methods. *Phys. Chem. Chem. Phys.* **22**, 7169–7192 (2020).

19 Bannwarth, C., Ehlert, S. & Grimme, S. GFN2-xTB—An Accurate and Broadly Parametrized Self-Consistent Tight-Binding Quantum Chemical Method with Multipole Electrostatics and Density-Dependent Dispersion Contributions. *J. Chem. Theory Comput.* **15**, 1652–1671 (2019).

20 Lu, T. *Molclus program, Version 1.9.9.9*, <http://www.keinsci.com/research/molclus.html > (accessed February 5, 2022).

21 Gaussian 16 Rev. A.03 (Wallingford, CT, 2016).

22 Becke, A. D. Density-functional thermochemistry. III. The role of exact exchange. *J. Chem. Phys.* **98**, 5648–5652 (1993).

23 Stephens, P. J., Devlin, F. J., Chabalowski, C. F. & Frisch, M. J. Ab Initio Calculation of Vibrational Absorption and Circular Dichroism Spectra Using Density Functional Force Fields. *J. Phys. Chem.* **98**, 11623–11627 (1994).

24 Lee, C., Yang, W. & Parr, R. G. Development of the Colle-Salvetti correlation-energy formula into a functional of the electron density. *Phys. Rev. B* **37**, 785–789 (1988).

25 Weigend, F. & Ahlrichs, R. Balanced basis sets of split valence, triple zeta valence and quadruple zeta valence quality for H to Rn: Design and assessment of accuracy. *Phys. Chem. Chem. Phys.* **7**, 3297–3305 (2005).

26 Grimme, S., Antony, J., Ehrlich, S. & Krieg, H. A consistent and accurate ab initio parametrization of density functional dispersion correction (DFT-D) for the 94 elements H-Pu. *J. Chem. Phys.* **132**, 154104 (2010).

27 Grimme, S., Ehrlich, S. & Goerigk, L. Effect of the damping function in dispersion corrected density functional theory. *J. Comput. Chem.* **32**, 1456–1465 (2011).

28 Barone, V. & Cossi, M. Quantum Calculation of Molecular Energies and Energy Gradients in Solution by a Conductor Solvent Model. *J. Phys. Chem. A* **102**, 1995–2001 (1998).

29 Cossi, M., Rega, N., Scalmani, G. & Barone, V. Energies, structures, and electronic properties of molecules in solution with the C-PCM solvation model. *J. Comput. Chem.* **24**, 669–681 (2003).

30 Zhao, Y. & Truhlar, D. G. The M06 suite of density functionals for main group thermochemistry, thermochemical kinetics, noncovalent interactions, excited states, and transition elements: two new functionals and systematic testing of four M06-class functionals and 12 other functionals. *Theor. Chem. Acc.* **120**, 215–241 (2008).

31 Chai, J.-D. & Head-Gordon, M. Long-range corrected hybrid density functionals with damped atom–atom dispersion corrections. *Phys. Chem. Chem. Phys.* **10**, 6615–6620 (2008).

32 Caldeweyher, E. et al. A generally applicable atomic-charge dependent London dispersion correction. *J. Chem. Phys.* **150**, 154122 (2019).

33 Neese, F., Wennmohs, F., Becker, U. & Riplinger, C. The ORCA quantum chemistry program package. *J. Chem. Phys.* **152**, 224108 (2020).

34 Gallarati, S. et al. OSCAR: an extensive repository of chemically and functionally diverse organocatalysts. *Chem. Sci.* **13**, 13782–13794 (2022).

35 Nistanaki, S. K. et al. Catalytic asymmetric C–H insertion reactions of vinyl carbocations. *Science* **378**, 1085–1091 (2022).

36 Legault, C. Y. CYLview; Université de Sherbrooke, 2009 (http://www.cylview.org).

37 Johnson, E. R. et al. Revealing Noncovalent Interactions. *J. Am. Chem. Soc.* **132**, 6498–6506 (2010).

38 Lu, T. & Chen, F. Multiwfn: A multifunctional wavefunction analyzer. *J. Comput. Chem.* **33**, 580–592 (2012).

39 Álvarez-Moreno, M. et al. Managing the Computational Chemistry Big Data Problem: The ioChem-BD Platform. *J. Chem. Inf. Model.* **55**, 95–103 (2015).

40 Emamian, S., Lu, T., Kruse, H. & Emamian, H. Exploring Nature and Predicting Strength of Hydrogen Bonds: A Correlation Analysis Between Atoms-in-Molecules Descriptors, Binding Energies, and Energy Components of Symmetry-Adapted Perturbation Theory. *J. Comput. Chem.* **40**, 2868–2881 (2019).

41 Maeda, S., Harabuchi, Y., Takagi, M., Taketsugu, T. & Morokuma, K. Artificial Force Induced Reaction (AFIR) Method for Exploring Quantum Chemical Potential Energy Surfaces. *Chem. Rec.* **16**, 2232–2248 (2016).

42 Maeda, S., Ohno, K. & Morokuma, K. Systematic exploration of the mechanism of chemical reactions: the global reaction route mapping (GRRM) strategy using the ADDF and AFIR methods. *Phys. Chem. Chem. Phys.* **15**, 3683–3701 (2013).

43 Weigend, F. Accurate Coulomb-fitting basis sets for H to Rn. *Phys. Chem. Chem. Phys.* **8**, 1057–1065 (2006).

44 Falivene, L. et al. Towards the online computer-aided design of catalytic pockets. *Nat. Chem.* **11**, 872–879 (2019).
